# Osteolytic cancer cells induce vascular/axon guidance processes in the bone/bone marrow stroma

**DOI:** 10.18632/oncotarget.25608

**Published:** 2018-06-22

**Authors:** Janine Hensel, Antoinette Wetterwald, Ramzi Temanni, Irene Keller, Carsten Riether, Gabri van der Pluijm, Marco G. Cecchini, George N. Thalmann

**Affiliations:** ^1^ Urology, Department for BioMedical Research, University of Bern, Bern, Switzerland; ^2^ Department of Urology, Inselspital, Bern University Hospital, Bern, Switzerland; ^3^ Biomedical Informatics Division, Research Branch, Sidra Medical and Research Center, Doha, Qatar; ^4^ Department for Biomedical Research, University of Bern, Bern, Switzerland; ^5^ Swiss Institute of Bioinformatics, University of Bern, Bern, Switzerland; ^6^ Tumor Immunology, Department for BioMedical Research, University of Bern, Bern, Switzerland; ^7^ Department of Medical Oncology, Inselspital, Bern University Hospital, Bern, Switzerland; ^8^ Department of Urology, Leiden University Medical Centre, Leiden, Netherlands

**Keywords:** prostate and breast cancer, osteolytic bone metastasis, stroma, angiogenesis, axon guidance

## Abstract

Prostate and breast cancers frequently metastasize to bone. The physiological bone homeostasis is perturbed once cancer cells proliferate at the bone metastatic site. Tumors are complex structures consisting of cancer cells and numerous stroma cells.

In this study, we show that osteolytic cancer cells (PC-3 and MDA-MB231) induce transcriptome changes in the bone/bone marrow microenvironment (stroma). This stroma transcriptome differs from the previously reported stroma transcriptome of osteoinductive cancer cells (VCaP). While the biological process “angiogenesis/vasculogenesis” is enriched in both transcriptomes, the “vascular/axon guidance” process is a unique process that characterizes the osteolytic stroma. In osteolytic bone metastasis, angiogenesis is denoted by vessel morphology and marker expression specific for arteries/arterioles. Interestingly, intra-tumoral neurite-like structures were in proximity to arteries. Additionally, we found that increased numbers of mesenchymal stem cells and vascular smooth muscle cells, expressing osteolytic cytokines and inhibitors of bone formation, contribute to the osteolytic bone phenotype.

Osteoinductive and osteolytic cancer cells induce different types of vessels, representing functionally different hematopoietic stem cell niches. This finding suggests different growth requirements of osteolytic and osteoinductive cancer cells and the need for a differential anti-angiogenic strategy to inhibit tumor growth in osteolytic and osteoblastic bone metastasis.

## INTRODUCTION

Prostate and breast cancers are frequently diagnosed solid cancers that often metastasize to bone [1, 2]. Bone metastases are diagnosed in approximately 70% of advanced prostate and breast cancer patients [3]. Proliferation of cancer cells at the bone metastatic site causes a number of skeletal-related events, such as severe pain, fractures, spinal cord/intervertebral nerve compression and hypercalcemia. Even today, bone metastases remain incurable and therapies are limited to prevent skeletal-related events and to control pain.

Nowadays, it is widely accepted that the tumor-associated microenvironment supports cancer cell growth at the primary and metastatic site. Once disseminated cancer cells proliferate at the bone metastatic site, normal bone physiology, characterized by balanced bone formation and resorption, is perturbed. Cancer cells either stimulate an excess of bone formation (osteoblastic or osteosclerotic bone metastasis) or stimulate an excess of bone resorption with concomitant block of bone formation (osteolytic bone metastasis) [4]. Prostate cancer cells preferentially induce osteoblastic bone metastasis, whereas breast cancer cells predominantly display an osteolytic bone phenotype. These two distinct stroma responses suggest that cancer cells have different growth requirements.

Osteolytic cancer cell-derived factors, such as parathyroid hormone like hormone (PTHLH/PTHrP), interleukin (IL)1, IL6, IL8, IL10, receptor activator of nuclear factor kappa B ligand (RANKL), colony stimulating factor 1 (CSF1), tumor necrosis factor α (TNFα) and Jagged1, directly or indirectly stimulate osteoclastogenesis [5, 6]. Additionally, osteolytic prostate cancer cells secrete the bone morphogenetic protein (BMP) antagonist, noggin, which blocks bone formation, contributing to the progression of the osteolytic lesion [7].

Few studies addressed the stromal changes occurring in osteolytic bone metastasis. According to the “vicious cycle” hypothesis the major relevant biological processes are increased osteoclastogenesis and activated osteoclastic bone resorption. Bone resorption releases bone matrix embedded growth factors, such as transforming growth factor beta (TGFβ), insulin-like growth factor 1 (IGF1) and calcium ions, which further fuel cancer cell growth and secretion of PTHLH, thus amplifying and perpetuating the process [4, 8]. Other studies highlighted that stroma cell derived factors are fundamental to release soluble RANKL and Tgfβ from their latent forms [9–11]. One of the early events, both in primary tumors and the metastatic site, is the stimulation of angiogenesis by cancer cell-derived factors [12]. However, the role of angiogenesis in bone metastasis in general [13, 14] and specifically in osteolytic bone metastasis [15] is almost unexplored.

In the clinics the strategy to inhibit bone metastatic tumor growth is based on the vicious cycle hypothesis and therefore, inhibition of bone resorption remains the treatment of choice. In the clinical setting bisphosphonate (BP)-mediated inhibition of bone resorption is an effective strategy to reduce skeletal-related events [16], despite the lack of proof for a direct negative impact of BPs on cancer cell growth and tumor mass. In animal models of bone metastasis, BP treatment effectively abolishes bone resorption, however, with no effect on total tumor burden as cancer cell proliferation persists [17, 18]. This suggests that mechanisms other than bone resorption support cancer cell growth at the bone metastatic site. Our current study is designed to systematically analyze osteolytic cancer cell induced molecular changes in the bone/bone marrow stroma.

## RESULTS

### Osteolytic prostate and breast cancer cells alter the bone/bone marrow stroma transcriptome

**Figure 1:**
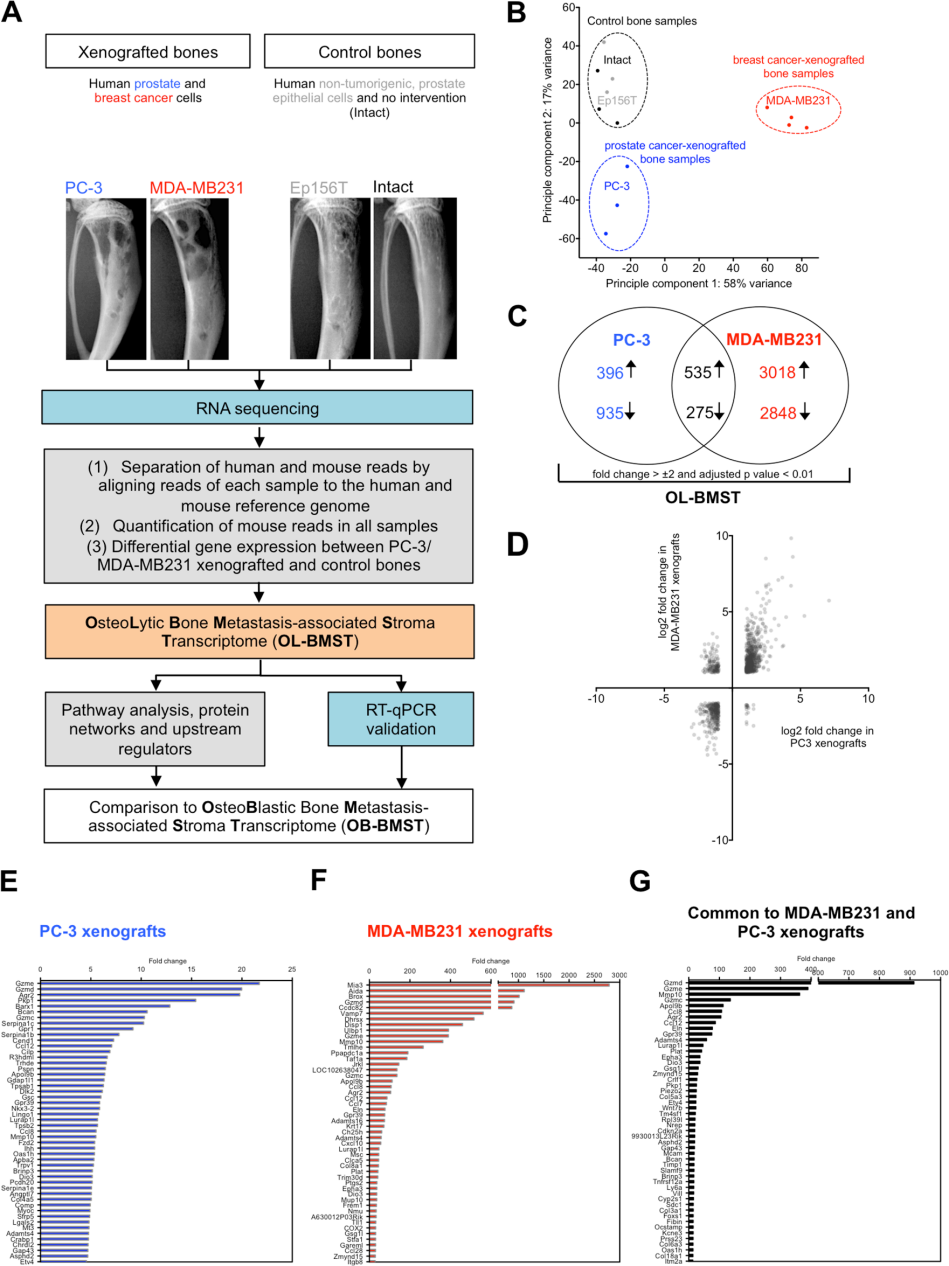
Bones xenografted with osteolytic prostate and breast cancer cells alter the gene expression profile of the bone/bone marrow stroma **(A)** Flow chart outlining experimental (blue) and bioinformatic (grey) steps used to define the stroma response signature in osteolytic bone metastasis (OL-BMST) (orange). **(B)** Principle component analysis showing the sample distribution of prostate (blue - PC-3 cell line) and breast (red - MDA-MB231 cell line) cancer cell line xenografted bones, Ep156T xenografted bones (grey) and intact bones (black). Each dot represents one mouse. **(C)** Venn diagram showing the number of overlapping and unique genes differentially expressed in PC-3 (*p value* < 0.01) and MDA-MB231 (*p value* < 0.01) xenografted bones *versus* controls. The sum of differentially expressed genes is referred to as the OL-BMST. **(D)** Scatter plot showing log2 fold change of differentially expressed genes in PC-3 and MDA-MB231 xenografts. **(E)** Top 50 annotated up-regulated genes in the PC-3 xenografts. **(F)** Top 50 annotated up-regulated genes in the MDA-MB231 xenografts. **(G)** Top 50 annotated up-regulated genes common to both, PC-3 and MDA-MB231 xenografts.

We took advantage of bone metastasis xenograft models to identify the molecular changes in the bone/bone marrow stroma in response to osteolytic cancer cells. In this study, osteolytic human prostate (PC-3) or breast (MDA-MB231) cancer cells were intra-osseously inoculated into the bone marrow cavity of tibial bones of immunocompromised mice. Figure 1A outlines the experimental strategy to define the molecular changes in the stroma of osteolytic bone metastasis. RNA sequencing data were explored by using a principal component analysis (PCA). The PCA plot showed a distinct separation of control bone samples from the cancer cell-xenografted samples, showing that osteolytic cancer cells alter the bone/bone marrow stroma transcriptome (Figure 1B). Interestingly, prostate and breast cancer cell-xenografted bone samples also separated from each other in the PCA plot. This observation shows that despite the capacity of osteolytic prostate and breast cancer cell in inducing an osteolytic bone phenotype, the regulation of genes is not identical. When compared to control bones, 2141 and 6676 stroma genes were differentially expressed in PC-3 and MDA-MB231 xenografts, respectively (Supplementary Tables 1 and 2). In total, 810 stroma genes were differentially expressed in both, PC-3 and MDA-MB231 xenografts (fold change > ±2, adjusted *p value* < 0.01) (Figure 1C, Supplementary Table 3). The VENN diagram illustrates that the osteolytic stroma response consists of two components, (1) a shared response component independent of cancer cell origin and (2) a specific response component depending on cancer cell origin. The majority of differentially expressed stromal genes were up- or down-regulated consistently in both xenografts, which was illustrated by the scatter plot displaying the log2 fold change in PC-3 *versus* MDA-MB231 xenografts (Figure 1D). Subsequently, our analysis is focused on overlapping differentially expressed genes showing a concordant gene regulation in both xenograft models. It is likely that those are important genes determining the osteolytic phenotype. The bar graphs in Figure 1E-1G display the top 50 annotated, up-regulated stroma genes and their fold change in PC-3 xenografts (Figure 1E), MDA-MB231 xenografts (Figure 1F) and genes common to both, PC-3 and MDA-MB231 xenografts (Figure 1G).

**Table 1:** Activated upstream regulators common to PC-3 and MDA-MB231 xenografts

		PC-3 xenografts		MDA-MB231 xenografts	
Upstream Regulator	Molecule Type	Activation z-score	p-value of overlap	Activation z-score	p-value of overlap
TGFB1	growth factor	4.8	2.31E-17	8.8	6.24E-69
TP53	transcription regulator	3.8	5.13E-13	4.8	4.40E-47
ERBB2	kinase	2.5	1.56E-10	3.2	4.55E-29
BNIP3L	other	4.4	4.04E-08	3.3	2.43E-13
TWIST1	transcription regulator	2.2	9.07E-08	3.6	1.19E-10
thioacetamide	chemical toxicant	3.2	9.69E-07	2.5	1.15E-16
camptothecin	chemical reagent	3.5	1.27E-06	3.4	2.38E-17
WNT3A	cytokine	2.9	3.90E-06	4.3	3.08E-08
NUPR1	transcription regulator	5.1	8.03E-06	3.2	2.44E-10
CTNNB1	transcription regulator	2.8	1.10E-05	3.8	2.75E-22
SPARC	other	2.4	1.14E-05	2.7	8.81E-09
bleomycin	chemical drug	3.0	1.88E-05	4.5	8.35E-18
GDF2	growth factor	2.5	1.91E-05	3.3	7.59E-08
BMP2	growth factor	3.1	2.46E-05	3.4	1.63E-04
HTT	transcription regulator	2.4	3.09E-05	2.3	6.26E-15
SMAD3	transcription regulator	3.3	3.24E-05	4.4	2.96E-15
SMARCA4	transcription regulator	2.2	3.79E-05	2.9	6.92E-14
Tgf beta	group	3.4	5.14E-05	5.4	1.11E-19
CDKN2A	transcription regulator	3.7	7.18E-05	5.0	1.37E-18
RB1	transcription regulator	2.9	7.61E-04	2.7	2.38E-10
CTGF	growth factor	2.8	1.22E-03	2.6	1.19E-04
RETNLB	other	2.9	1.48E-03	3.5	1.14E-11
TGFB3	growth factor	2.8	1.97E-03	5.2	7.68E-09
SMARCB1	transcription regulator	2.3	2.22E-03	3.0	4.39E-12
IL13	cytokine	2.0	2.30E-03	2.4	3.11E-13
Cg	complex	2.2	2.88E-03	6.3	1.33E-19
Am 580	chemical toxicant	2.2	3.82E-03	2.2	6.18E-03
tretinoin	chemical - endogenous mammalian	3.7	7.93E-03	4.3	1.15E-36
TNFSF11	cytokine	2.5	7.94E-03	3.3	4.94E-20
let-7	microrna	2.1	7.96E-03	2.6	8.69E-17
JUN	transcription regulator	2.2	1.08E-02	2.9	2.64E-16
KEAP1	transcription regulator	2.4	1.45E-02	2.1	4.45E-03
ochratoxin A	chemical toxicant	2.2	1.45E-02	3.4	4.45E-03
IL1A	cytokine	2.6	1.52E-02	5.5	6.60E-10
PDGFB	growth factor	2.4	2.32E-02	3.0	2.95E-05
MAP2K1	kinase	2.0	2.90E-02	2.7	2.02E-07
ERK	group	2.7	4.48E-02	4.3	2.16E-16

The table lists upstream regulators with their corresponding molecule type, activation z-score and *p-value* for both, PC-3 and MDA-MB231 xenografts.

**Table 2:** Genes of Acta2 (αSMA) dataset (GSE15062) shared with up-regulated genes common to both, PC-3 and MDA-MB231 xenografts

category	gene symbols					
Angiogenesis	Angptl2	Cd248	Ecm1	Eln	Tnfrsf12a	
**Adipocyte proliferation**	Aebp1					
**Blood brain barrier**	Mxra8					
**Blood coagulation**	Procr					
**Bone turn-over**	Pkdcc	Tcta	Wisp2			
**Cell adhesion**	Ddr1	Itga3	Itgbl1	Scarf2	Wtip	
**Cell mortility**	Acta2	Actg2	Bcar1	Marcksl1		
**Collagen-related**	Adamts2	Cthrc1	Loxl3	Pcolce		
**ECM**	Col18a1	Col4a5	Col6a2	Fbln2		
**Fibroblasts**	Nrep	Prrx2	S100a4			
**Growth factor**	Efemp2					
**Metabolism**	Cbr2	Ckb				
**Neurogenesis**	Chac1	Crlf1	Dbn1	Ncs1	Nrep	Serpinf1
	Thy1	Unc5b				
**Niche**	Gas1					
**Regulation of growth**	Gadd45b	Wtip				
**Smooth muscle cell proliferation**	Csrp2					
**Wnt signaling**	Dact3	Dkk3	Frzb	Fzd2	Fzd8	Trabd2b
**Others**	Ahnak2	Akr1b8	Apoe	Arhgef5	Ccdc12	Chst14
	Crtap	Dbndd2	Ddah2	Eva1b	Fhl2	Gpsm1
	Hspb2	Lurap1l	Ly6a	Meg3	Nnmt	Pcbp4
	Pfdn1	Phlda3	Pkp1	Pxdc1	Rabac1	Sod3
	Tagln	Tmed1	Tmem109	Tmem184b	Trim47	Tubb6

Taken together, these findings indicate that osteolytic cancer cells of different origin elicit a bone/bone marrow stroma response consisting of a (1) shared and (2) specific component.

### In the bone/bone marrow stroma osteolytic cancer cells induce pathways linked to angiogenesis and axon guidance

We analyzed pathways, biological processes (gene ontology (GO) terms), protein interactions and upstream regulators represented in the transcriptome to identify changes occurring in the bone/bone marrow stroma in response to osteolytic cancer cells.

**Figure 2:**
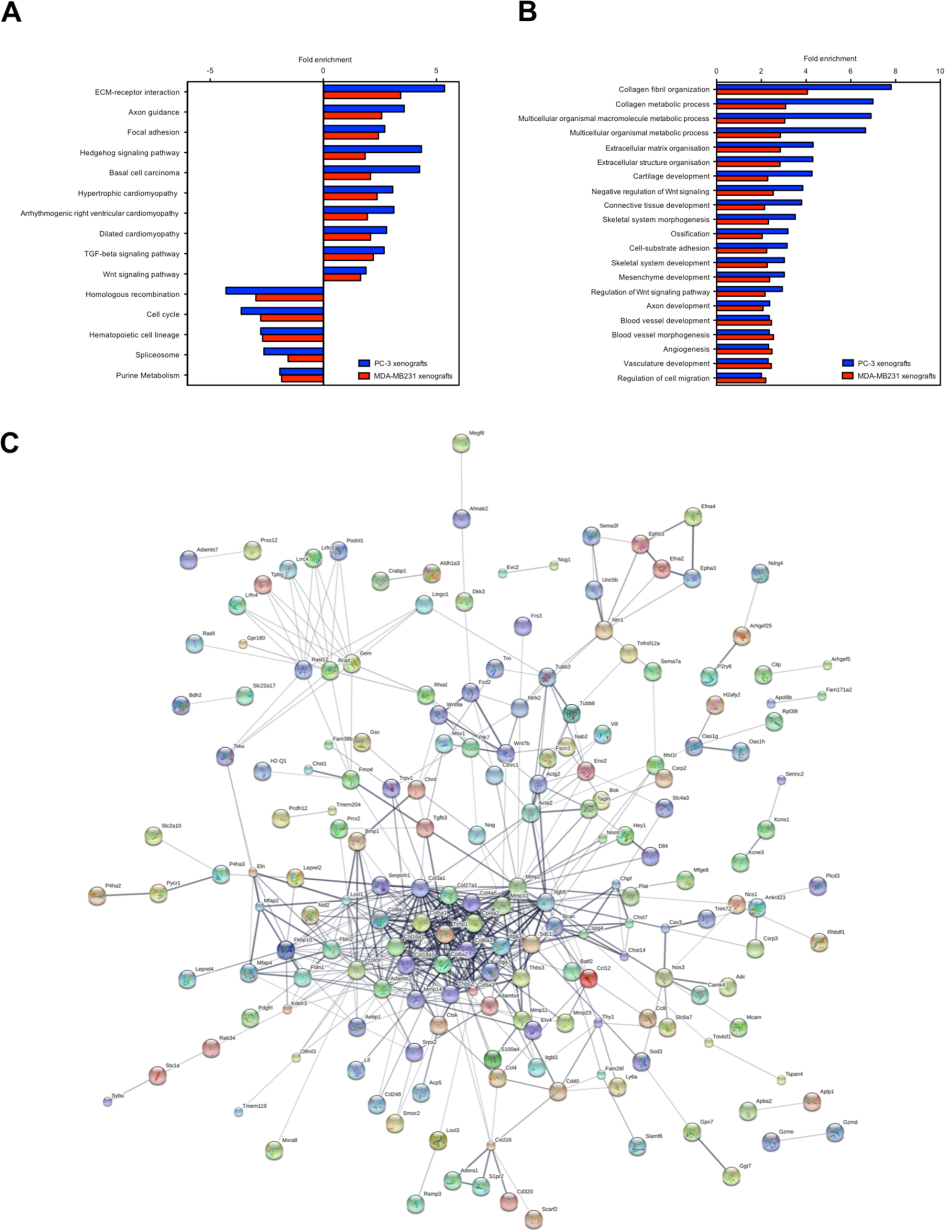
KEGG pathways, GO terms and protein interaction network of the osteolytic stroma response **(A)** Enriched pathways common to both, PC-3 and MDA-MB231 xenografts. **(B)** Enriched biological processes common to both, PC-3 and MDA-MB231 xenografts. **(C)** Protein interaction network generated from differentially expressed genes common to both, PC-3 and MDA-MB231 xenografts. The line thickness positively correlates with the confidence score that was obtained for each protein interaction.

ECM-receptor interaction, axon guidance, focal adhesion, hedgehog/Tgfβ/Wnt signaling pathways and cardiomyopathy were significantly enriched pathways (*p value* ≤ 0.05) in the up-regulated stroma genes common to PC-3 and MDA-MB231 xenografts (Figure 2A). The down-regulated stroma genes were significantly enriched for pathways (*p value* ≤ 0.05) associated to homologous recombination, cell cycle, hematopoietic cell lineage, spliceosome metabolism and purine metabolism (Figure 2A). Prominent significantly enriched biological processes were collagen metabolic process, ECM organization, blood vessel development, bone development and axon development (FDR ≤ 0.001) (Figure 2B). Accordingly, the protein network analysis of the osteolytic stroma transcriptome revealed collagens (Col3a1, Cold5a1, Col6a2), matrix metalloprotease 2 (Mmp2) and Elastin as the central protein nodes with most interaction partners (Figure 2C). We performed an upstream molecule analysis to predict molecules inducing the stroma response in osteolytic bone metastasis. Thirty-seven shared activated upstream regulators were identified for the PC-3 and MDA-MB231 xenografts (Table 1). The upstream regulators consist of 8 growth factors (Bmp2/4, Ctgf, Gdf2, Igf1, Pdgfb, Tgfβ1/3), 4 cytokines (Il1a, Il13, Tnfsf11, Wnt3a), transcription regulators (Cdkn2a, Ctnnb1, Htt, Jun, Keap1, Nupr1, Rb1, Smad3, Smarca4, Smarcb1, Tp53, Twist1) and one miRNA (let-7) and kinase (Map2k1). Tgfβ1 had the highest activation score (4.8 and 8.8 in PC-3 and MDA-MB231 xenografts, respectively) and stringency (2.3E-17 and 6.2E-69 in PC-3 and MDA-MB231 xenografts, respectively).

Taken together, osteolytic and osteoinductive cancer cells both induce angiogenesis in the bone/bone marrow stroma. However, only osteolytic cancer cells concomitantly induce axon guidance.

### Genes of the axon guidance pathway are specifically induced in the stroma of osteolytic bone metastasis

**Figure 3:**
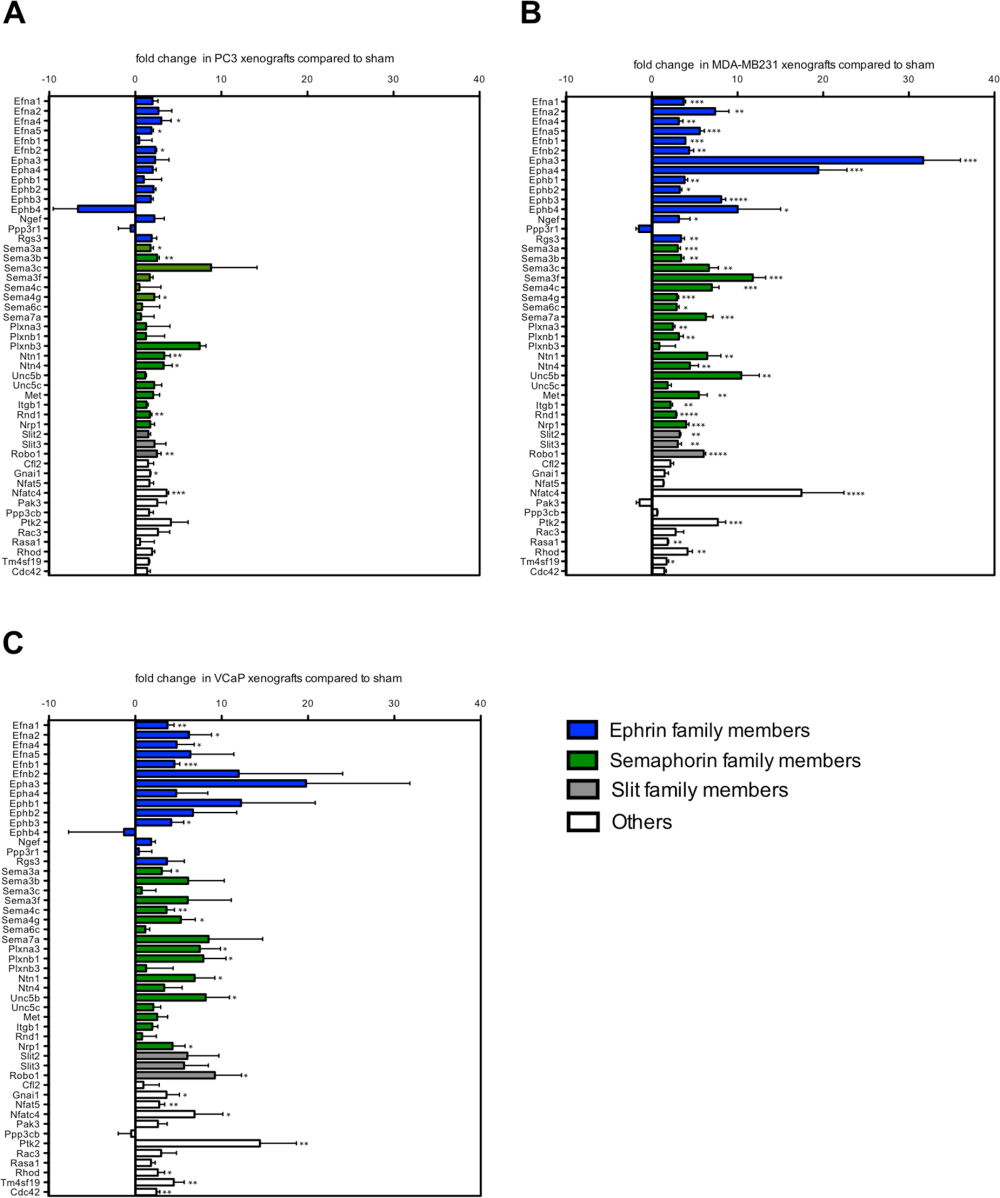
A subset of axon guidance molecules is exclusively induced in osteolytic bone metastasis Fold change expression levels of axon guidance genes in osteolytic (PC-3 and MDA-MB231 xenografts) and osteoblastic (VCaP xenografts) bone metastasis compared to sham-operated animals. Expression levels of ephrin family members (blue bars), semaphorin family members (green bars), Slit family members (grey bars) and others (white bars) in **(A)** PC-3, **(B)** MDA-MB231 and **(C)** VCaP xenografts. Values are shown as fold change (mean ± SD) relative to sham-operated animals. ^*^, *P* < 0.01; ^**^, *P* < 0.001; ^***^, *P* < 0.0001; ^****^, *P* < 0.0001.

The axon guidance pathway was one of the most significantly enriched pathways in the stroma of osteolytic bone metastasis. To validate the differential gene expression of axon guidance pathway family members, we measured their expression in the stroma of osteolytic bone metastasis using mouse-specific real time PCR primers (Figure 3A, 3B). Additionally, we investigated the same gene panel in the stroma of osteoblastic bone metastasis (Figure 3C) to determine whether these genes are specific to the osteolytic stroma response. The genes of the axon guidance pathways were either grouped to ephrin (ephrin ligands, ephrin receptors, ephrin signaling), semaphorin (semaphorin ligands, semaphorin receptors) or slit (slit ligands, slit receptor) family members. Genes that could not be assigned to any of these category were grouped to the panel “others”.

Out of the 49 measured genes, the following genes were significantly induced in the stroma of one or both models of osteolytic bone metastasis, but not in the stroma of osteoblastic bone metastasis: ephrin ligands (Efna5, Efnb2) ephrin receptors (Epha3, Epha4, Ephb1, Ephb2, Ephb4) ephrin signaling molecules (Ngef, Rsg3), semaphorin ligands (Sema3b, Sema3c, Sema3f, Sema6c, Sema7a), semaphorin receptors (Ntn4, Met, Itgb1, Rnd1), slit ligands (Slit2, Slit3) and one gene (Rasa1) out of the category “others”. Two significantly induced genes, Nfat5 and Cdc42, were uniquely induced in the stroma of osteoblastic bone metastasis, however, not in the stroma of osteolytic bone metastasis.

These findings suggest that the gene regulation of numerous members of the axon guidance pathway can be specifically attributed to the osteolytic stroma response.

### Markers associated with arteries are significantly upregulated in the stroma of osteolytic bone metastasis

The comparison of the biological processes of the osteolytic stroma response with the previously published biological processes in the osteoblastic bone metastasis-associated stroma (OB-BMST)[19], showed that most biological processes were shared. Processes related to angiogenesis, ECM remodeling, cell adhesion and skeletal system development were enriched in both osteolytic and osteoblastic stroma transcriptomes.

**Figure 4:**
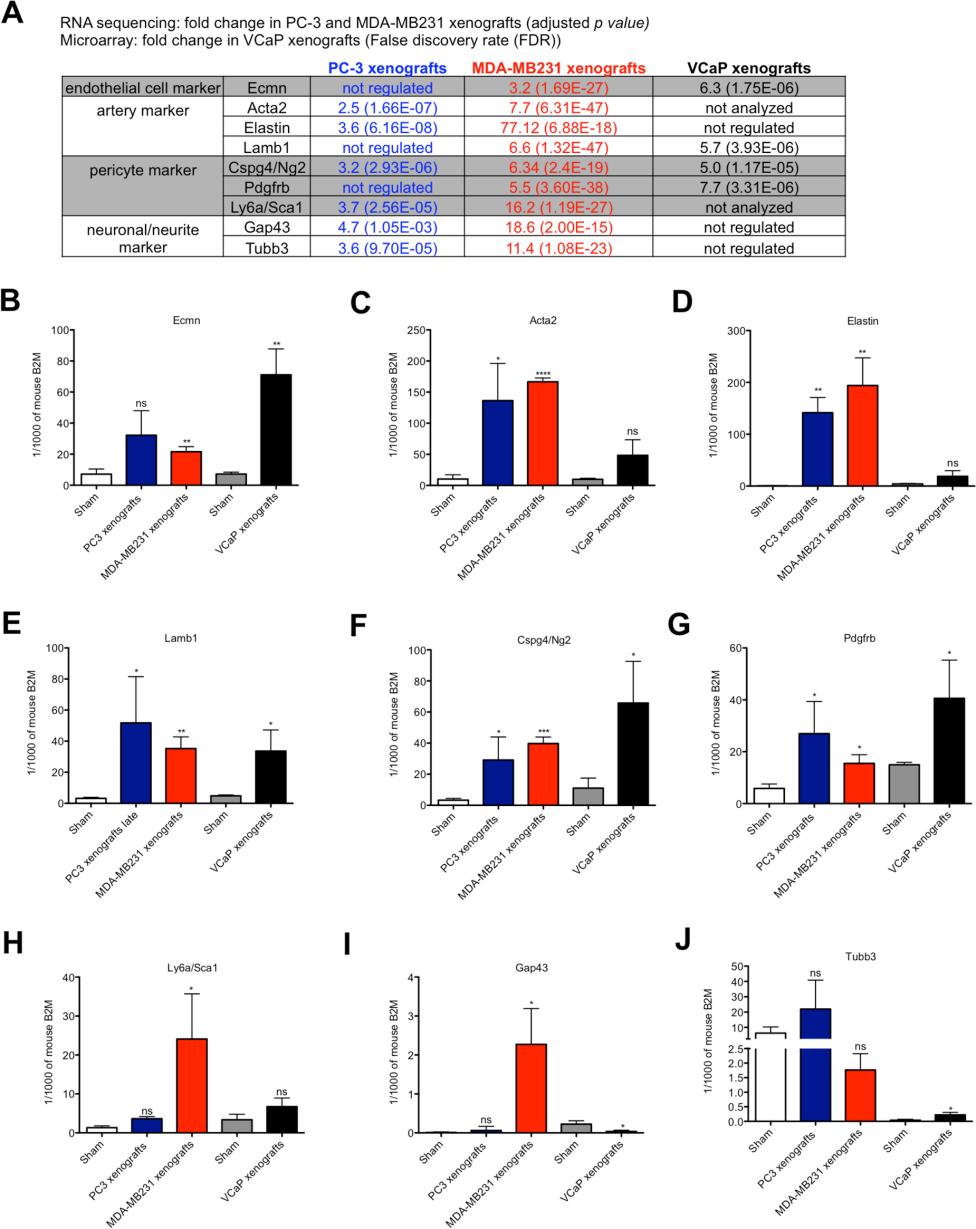
Expression analysis of endothelial cell, artery, pericyte and neuronal/neurite markers in osteolytic and osteoblastic bone metastasis **(A)** Fold change of genes, associated to endothelial cells, arteries, pericytes and neurons/neurites, measured by RNA sequencing (for PC-3 and MDA-MB231 xenografts *versus* controls) or microarray data (for VCaP xenografts *versus* controls). B-J. Relative expression levels of endothelial cell **(B)**, artery **(C-E)**, pericyte **(F-H)** and neuron/neurite **(I** and **J)** markers in osteolytic (PC-3 and MDA-MB231 xenografts) and osteoblastic (VCaP xenografts) bone metastasis and corresponding sham-operated animals. Expression levels are shown for PC-3 (blue bar), MDA-MB231 (red bar), VCaP (black bar) xenografts and corresponding sham-operated animals (white bar: sham-operated animals of PC3 and MDA-MB231 xenografts models; grey bar: sham-operated animals of VCaP xenografts). Values for each gene is shown as relative expression to mouse B2M (mean ± SD). ^*^, *P* < 0.01; ^**^, *P* < 0.001; ^***^, *P* < 0.0001; ^****^, *P* < 0.0001, ns - not significant.

In the RNA sequencing data genes linked to arteries (Elastin, Lamb1, Acta2), pericytes (Cspg4/Ng2, Pdgfrb, Sca1 (Ly6a)) and neurons/neurites (Gap43, Tubb3) were up-regulated in the stroma of osteolytic bone metastasis, (Figure 4A). First, we validated the differential gene expression of these markers in the stroma of osteolytic and osteoblastic bone metastasis using mouse-specific real time PCR primers (Figure 4B-4J). Endomucin mRNA was up-regulated in both, osteolytic and osteoblastic, stroma transcriptomes; however, the extent of induction was higher in osteoblastic bone metastasis as compared to osteolytic bone metastasis (Figure 4B). The artery markers, Acta2 and Elastin, were significantly up-regulated in response to osteolytic cancer cells, however, not in response to osteoinductive cancer cells (Figure 4C and 4D). Lamb1, another artery marker, was significantly up-regulated in both, osteolytic and osteoblastic, stroma transcriptomes (Figure 4E). Cspg4/Ng2 and Pdgfrb were significantly up-regulated in the stroma of both osteolytic and osteoblastic bone metastasis and also the induction level was not significantly different (Figure 4F and 4G). Sca1 (Ly6a) was only significantly up-regulated in MDA-MB231 xenografts, however, not in PC-3 xenografts and VCaP-xenografts (Figure 4H). In the literature, it has been reported that the vascular and neuronal networks are tightly linked, which is also confirmed by our pathway analysis. In our RNA sequencing data, we found that the neurite/neuronal marker, Gap43 and β3 tubulin, were significantly up-regulated in the stroma of osteolytic bone metastasis (Figure 4A). Only the up-regulation of Gap43 was confirmed using mouse-specific real time PCR probes (Figure 4I).

Taken together, these findings suggest that markers associated with arteries are specifically up-regulated in the stroma of osteolytic bone metastasis.

### The stroma of osteolytic bone metastasis is comprised of an arterial network

**Figure 5:**
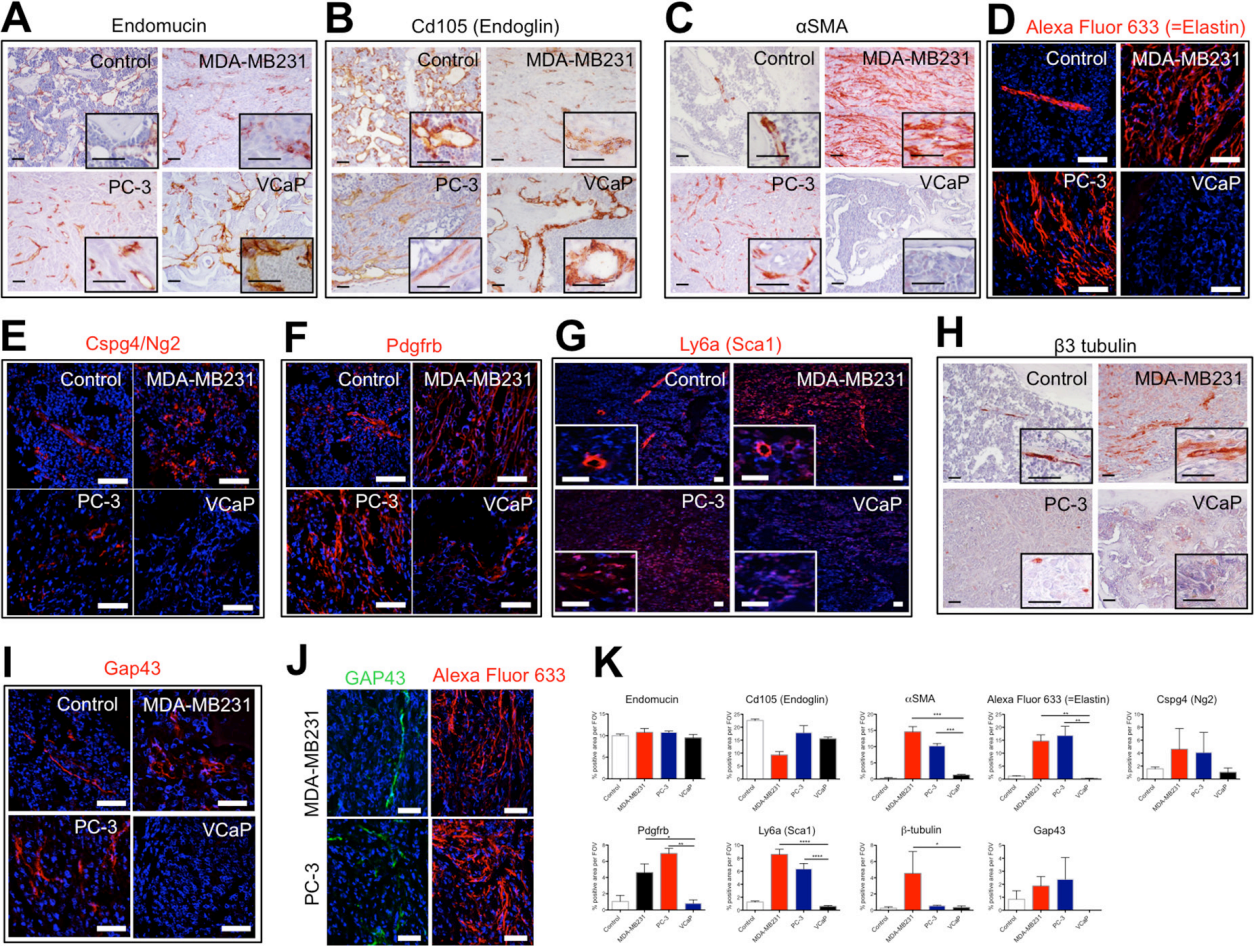
The stroma of osteolytic bone metastasis is comprised of an arterial network Immunohistochemical or immunofluorescent detection of endothelial cell markers (Endomucin **(A)** and Cd105 (Endoglin) **(B)**), artery markers (αSMA **(C)** and Elastin **(D)**), pericyte markers (Cspg4/Ng2 **(E)**, Pdgfrb **(F)** and Sca1 **(G)**) and neuronal/neurite markers (β3 tubulin **(H)** and Gap43 **(I)** in control bones, MDA-MB231 xenografts, PC-3 xenografts and VCaP xenografts. **(J)** Immunofluorescent detection of Gap43 positive cells in proximity to Elastin positive cells in PC-3 and MDA-MB231 xenografts. Scale bar = 50 *μ*m. **(K)** Quantification of percent positive staining area per field of view (FOV) for endomucin, Cd105 (Endoglin), αSMA, Alexa Fluor 633 (=Elastin), Cspg4/Ng2, Pdgfrb, Sca1, β3 tubulin and Gap43.

To characterize the vessels present in osteolytic and osteoblastic bone metastases, we analyzed the protein expression of endothelial cells markers (endomucin and Cd105), artery markers (αSMA and Elastin), pericyte markers (Cspg4/Ng2, Pdgfrb, Sca1) and neurite/neuronal marker (Gap43 and β3 tubulin) (Figure 5).

Endomucin immunoreactivity was found in the normal bone marrow and in the stroma of osteolytic and osteoblastic bone metastasis (Figure 5A). In the stroma of osteolytic bone metastasis, endomucin positive cells were detected as elongated, intra-tumoral vessel-like structures, whereas in the osteoblastic bone metastasis endomucin-labeled structures were similar to the sinusoidal vessel morphology of the control bone marrow. Interestingly, in VCaP tumors, endomucin-positive cells surround tumor areas and associated to the bone-forming surfaces, whereas the intra-tumoral regions were devoid of endomucin immunoreactivity. Cd105 immunoreactivity showed a similar expression patterns as endomucin (Figure 5B).

Alpha SMA protein expression in normal bone marrow marked arteries/arterioles. Interestingly, the stroma of osteoblastic bone metastasis was devoid of αSMA immunoreactivity (Figure 5C). In the stroma of osteolytic bone metastasis αSMA positive cells were distributed in vessel-like structures throughout the entire stroma. When we stained for an epithelial marker (marking cancer cells) and αSMA in PC-3 and MDA-MB231 xenografts in consecutive sections, αSMA positive cells did not express the epithelial marker, Epcam and Pan-cytokeratin, respectively (Supplementary Figure 1). To confirm that αSMA positive cells were of stromal origin and not cancer cells expressing a mesenchymal marker, we performed laser-captured micro-dissection of αSMA positive cells from MDA-MB231 xenografts. In RNA samples of αSMA positive cells only mouse Acta2 was detected, whereas human Acta2 was absent (data not shown). This analysis substantiates the stroma origin of αSMA positive cells. We used a model of systemic bone metastasis, in which we injected MDA-MB231 cells into the left ventricle of the heart and analyzed the protein expression of αSMA in bone metastasis specimens. The accumulation of αSMA positive cells was observed in the same way as in the intra-osseous inoculated xenograft model (Supplementary Figure 2A). Furthermore, we demonstrated that αSMA positive cells also accumulated in immune-competent animals (syngeneic mouse model, in which 4T1.2 cells were injected intra-osseous) (Supplementary Figure 2B).

To further characterize the marker expression of the intra-tumoral vessels, animals bearing intra-osseous xenografts of PC-3, MDA-MB231 and VCaP cancer cells and sham-operated animals were intra-venously injected with Alexa Fluor 633, a dye that specifically labels arteries by binding to elastin [20]. In control bone marrow, the central artery and arterioles were labeled with Alexa Fluor 633, whereas in PC-3 and MDA-MB231 xenografts, intra-tumoral vessel-like structures were Alexa Fluor 633 labeled (Figure 5D). In contrast, osteoinductive VCaP xenografts were devoid of any Alexa Fluor 633 staining after *in vivo* labeling.

The protein expression analysis of pericyte markers, namely Cspg4 (Ng2), Pdgfrb and Ly6a (Sca1), showed that Cspg4 (Ng2) and Ly6a (Sca1) positive cells are associated to arteries/arterioles in normal bone marrow. Pdgfrb marked pericytes and also scattered cells in the normal bone marrow. There are numerous Cspg4 (Ng2) positive cells found intra-tumoral in osteolytic bone metastasis. In VCaP xenografts few Cspg4 (Ng2) positive cells were found around the tumor but not within the tumor. A similar expression pattern was found for Ly6a (Sca1) positive cells. In osteolytic bone metastasis Pdgfrb positive, stripe-like structures were found in the tumor, whereas in VCaP xenografts only few Pdgfrb positive cells were found in proximity to the bone surface (Figure 5E-5G).

In normal bone marrow β3 tubulin immunoreactivity is detected in cells close to the central artery. In contrast, in the stroma of osteolytic bone metastasis, β3 tubulin positive cells were scattered throughout the stroma. A higher frequency of β3 tubulin positive cells with different morphology (elongated, stripe-like) was seen in MDA-MB231 xenografts as compared to PC-3 xenografts. The immunoreactivity in PC-3 xenografts was less pronounced. The stroma of VCaP xenografts showed rare β3 tubulin positive cells (Figure 5H).

Additionally, we analyzed the protein expression of the early neurite marker Gap43. Gap43 expression was detected in cells close to the central artery and arterioles in normal bone marrow. Gap43 protein expression was found only in osteolytic bone metastasis but not in osteoblastic bone metastasis (Figure 5I). Gap43 expression was detected in close proximity to Alexa Fluor 633 labeled cells (Figure 5J).

In summary, osteolytic cancer cells induce an accumulation of cells staining positive for αSMA, Elastin, Pdgfrb and Ly6a (Sca1) (Figure 5K).

### Osteolytic cytokines and BMP/Wnt antagonists are expressed by mesenchymal stem cells and αSMA positive cells

Osteolytic bone metastases are characterized by increased bone resorption and decreased bone formation. Previously, we reported that prostate cancer cells secrete the BMP antagonist, noggin, and, thereby, bone formation is inhibited [7]. Interestingly, our RNA sequencing data pinpointed that the stroma serves as an additional source of BMP (Chordin, Noggin, Sclerostin) and Wnt antagonists (Dkk1, Dkk2, Dkk3, Frzb) (see supplementary results).

The osteolytic bone metastasis stroma contained an accumulation of αSMA positive cells and increased numbers of mesenchymal stem cells (MSCs) (see supplementary results).

To identify whether these two cell types express genes encoding osteolytic cytokines (Rankl and Csf1) and/or BMP/Wnt antagonists (Dkk1-3, Sost, Chordin, Noggin, Frzb), we analyzed mRNA expression of FACS-sorted MSCs and αSMA positive cells isolated from MDA-MB231 intra-osseous xenografts and control bones.

**Figure 6:**
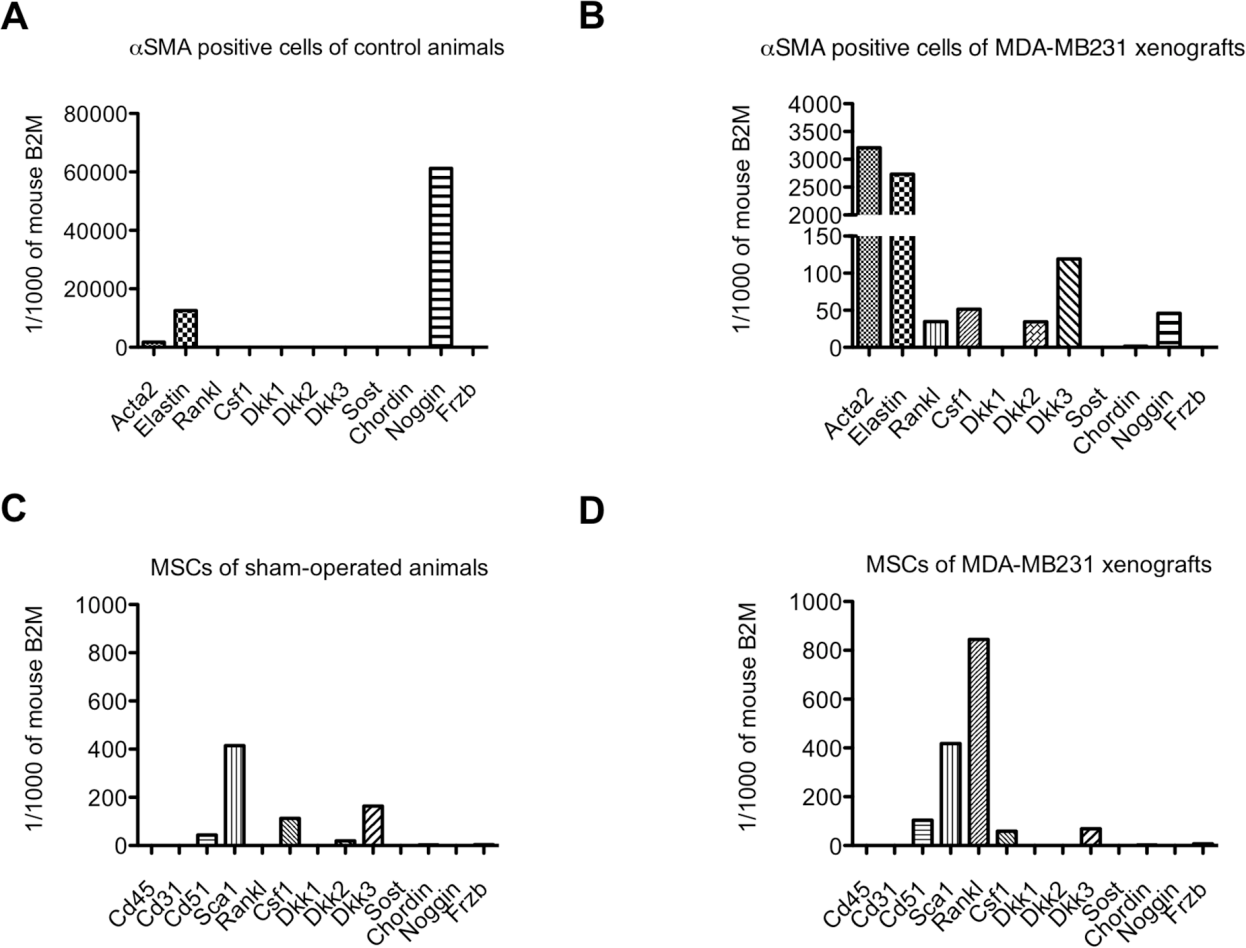
Osteolytic cytokine and Wnt/BMP antagonist expression in αSMA positive cells and mesenchymal stem cells (MSCs) isolated from control bones and MDA-MB231 xenografts Relative expression levels of osteolytic cytokines (*Rankl*, *Csf1*) and Wnt/BMP antagonists (*Dkk1*, *Dkk2*, *Dkk3*, *Sost*, *Chordin*, *Noggin*, *Frzb*) in αSMA positive cells **(A** and **B)** and mesenchymal stem cells (MSCs) **(C** and **D)** isolated from control bones (A and C) and MDA-MB231 xenografts (B and D).

Alpha SMA positive cells isolated from the bone/bone marrow of control animal express Noggin, which was also expressed by αSMA positive cells isolated from MDA-MB231 xenografts. Interestingly, αSMA positive cells from tumors additionally expressed Rankl, Csf1, Dkk2 and Dkk3 (Figure 6A and 6B).

MSCs from sham-operated animals expressed Csf1, Dkk2 and Dkk3. The expression pattern in MSCs isolated from MDA-MB231 xenografts was mostly retained, however, MSCs additionally expressed Rankl and Frzb (Figure 6C and 6D). Noteworthy was also the high expression level of Rankl in MSCs isolated from MDA-MB231 xenografts compared to αSMA positive cells.

In osteolytic bone metastasis, the source of osteolytic cytokines can be attributed to MSCs and αSMA positive cells. However, the major source of Rankl in osteolytic tumors are MSCs. Interestingly, the major source of BMP/Wnt antagonists are αSMA positive cells.

### Alpha SMA positive cells express genes involved in bone turn-over, cell adhesion, neurogenesis and Wnt signaling

Alpha SMA positive cells were the predominant cell type in the stroma of osteolytic bone metastasis. We analyzed which differentially expressed stromal genes in osteolytic bone metastasis can be attributed to αSMA positive cells. We utilized a published mRNA expression dataset (GSE15062), in which smooth muscle cells isolated from aortae of 8- to 12-week-old C57BL/6 mice were profiled [21]. We compared up-regulated genes in PC-3, MDA-MB231, VCaP xenografts and the dataset of αSMA positive cells. We focused on genes shared between the stroma response of osteolytic bone metastasis and the dataset characterizing αSMA positive cells and not overlapping with VCaP xenografts. Interestingly, we identified 80 genes (Table 2), among which various genes are involved in bone turnover (Pkdcc, Tcta, Wisp2), cell adhesion (Ddr1, Itga3, Itgbl1, Scarf2, Wtip), neurogenesis (Chac1, Crlf1, Dbn1, Ncs1, Nrep, Serpinf1, Thy1, Unc5b) and Wnt signaling (Dact3, Dkk3, Frzb, Fzd2, Trabd2b).

The growth support of αSMA positive cells towards cancer cells might be diverse, suppression of bone formation, on one hand, direct secretion of growth factors (Efemp2) on the other hand. In future it would be interesting to study the depletion of αSMA positive cells in osteolytic bone metastasis and whether this would alter growth of osteolytic cancer cells.

### Human bone metastasis are characterized by distinct vessel morphology

**Figure 7:**
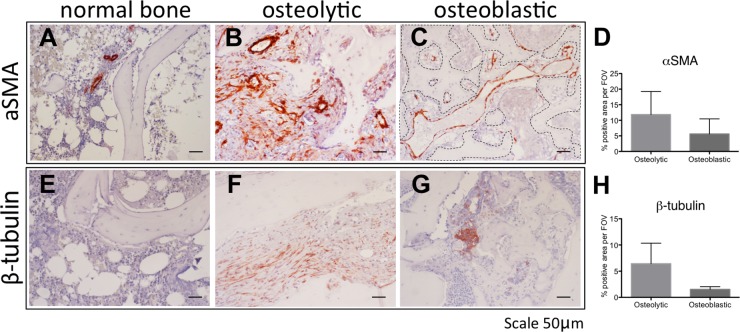
The stroma of human osteolytic bone metastases consist of arteries Immunohistochemical detection of αSMA **(A-C)** and β3 tubulin **(E-G)** in normal bone (A, E), in osteolytic bone metastasis (B, F) and in osteoblastic bone metastasis (C, G). Scale bar = 50 *μ*m. Positive staining area of αSMA **(D)** and β3 tubulin **(H)** was quantified in 2 specimens of osteoblastic bone metastasis and 3 specimens of osteolytic bone metastasis. Dotted line in panel C highlights the bone surface.

To translate our finding to human bone metastatic disease, we next investigated the protein expression of αSMA and β3 tubulin in normal bone and bone metastasis (Figure 7).

In normal bone αSMA protein expression was seen in arteries/arterioles in the hematopoietic marrow (Figure 7A). In osteolytic bone metastases arteries were labeled and also various scattered stroma cells stained positive for αSMA (Figure 7B). In osteoblastic bone metastasis, αSMA positive vessels, in proximity to the bone surface, are reminiscent of sinusoidal vessels (Figure 7C). In osteolytic bone metastasis a trend for more aSMA positive staining area was found, however, this was statistically not significant (Figure 7D). Normal bone marrow was devoid of β3 tubulin immunoreactivitiy (Figure 7E), however, in osteolytic bone metastasis elongated scattered β3 tubulin positive cells were found (Figure 7F). In contrast, in osteoblastic bone metastasis, immunoreactivity was attributed to cancer cells (Figure 7G). In osteolytic bone metastasis, β3 tubulin positive staining area trend to be higher, however, this was statistically not significant (Figure 7H).

Our findings indicate the cell types staining positive for aSMA and β3 tubulin at different in osteolytic and osteoblastic bone metastasis. Our findings suggest similarly to the mouse model, that osteolytic bone metastasis consist of arteries, whereas osteoblastic bone metastasis are characterized by sinusoidal vessel structures.

## DISCUSSION

This is the first systematic transcriptome analysis defining the bone/bone marrow stroma reaction in xenograft models of prostate and breast cancer-induced osteolytic bone metastasis. The stroma specificity of this transcriptome has been achieved by using bioinformatic tools discriminating tumor stroma (mouse transcripts) from cancer cell (human transcripts) transcriptomes.

Despite their distinct primary tumor origin, osteolytic prostate and breast cancer cells induce the same phenotypic stroma response in radiography images. Consistently, these cancer cells induce similar gene expression changes in the bone/bone marrow stroma.

One of the most enriched pathways induced in the bone/bone marrow stroma in response to osteolytic cancer cells is the axon guidance pathway. Genes within the axon guidance pathway have been shown to be involved in tumor progression and metastasis [22–24]. It has been also shown that in primary prostate and breast tumors autonomic nerve development contributes to cancer progression [25, 26]. Interestingly, elongated (neurite-like) and scattered cells can be found in the stroma of osteolytic bone metastasis. These cells express class 3 β tubulin and Gap43. Initially, class 3 β tubulin was commonly used as a neuron-specific marker, however, as a component of the mitotic spindle, its expression can be found in multiple cell types, including fibroblasts and cancer cells [27, 28]. Gap43 expression is associated with neurite outgrowth [29] and interestingly a component of the hematopoietic stem cell niche [30]. In osteolytic bone metastasis neurite-like structures are found intra-tumoral, therefore, Gap43 positive cells likely re-present an osteolytic cancer cell niche component. Previously, nerve growth factor induced Gap43 positive nerve fiber sprouting and re-organisation has been reported in a mouse breast cancer bone metastasis model [31]. Inhibition of nerve growth factor diminished the effect and attenuated bone pain.

In normal physiology, the central and peripheral nervous system controls bone remodelling [32]. Systemic activation of the sympathetic nervous system using a β1/2 adrenergic receptor non-selective agonist (isoproterenol) or chronic stress stimulates bone resorption and inhibits bone formation [33]. Isoproterenol stimulates Rankl secretion in osteoblasts leading to increased osteoclastogenesis and migration of cancer cells. In a breast cancer bone metastasis mouse model, betablocker, propranolol, prevented bone metastases [33].

Nerve fibers and larger blood vessels, such as arteries, align into two parallel structures [34]. Vessel patterning occurs in response to nerve-derived signals, and *vice versa*, vascular cell types can produce guidance cues for axons. Vascular patterning and growth processes are regulated by axon guidance cues [34]. In our RNA sequencing data, we found some axon guidance genes specifically up-regulated in the stroma of osteolytic bone metastasis suggesting that osteolytic cancer cells use axon guidance cues to generate an arterial network in the bone/bone marrow stroma.

Members of the semaphorin family are also part of the axon guidance pathway. Besides an involvement in neurite growth, these axon guidance molecules have also various other functions. Under physiological conditions, Semaphorin 3B and 7A are known to stimulate osteolysis and fusion of osteoclast progenitors, respectively [35, 36]. The observation that osteolytic cancer cells also induce expression of semaphorin family members in the bone/bone marrow compartment is novel and might be an additional mechanism to stimulate bone resorption. The function of most induced semaphorin family members on osteoclasts is not yet investigated, it would be worthwhile to study their functions in future experiments.

Osteolytic lesions are also the result of a decrease in osteoblast-mediated bone formation [7]. Previously, it was reported that osteolytic cancer cell growth is inhibited by the presence of osteoblasts [37]. Our data indicate that osteolytic breast and prostate cancer cells *in vivo* instruct the stroma to express Bmp/Wnt antagonists, which further suppress bone formation.

Another enriched process in the stroma of bone metastasis is angiogenesis. Angiogenesis is tightly coupled to osteoclastogenesis and bone formation in physiology and pathophysiology [38, 39], therefore, it was not entirely surprising that angiogenesis is an enriched pathway in both, osteolytic and osteoblastic bone metastasis. However, the vasculature was markedly different in osteolytic and osteoblastic bone metastasis. The sinusoidal morphology in osteoblastic metastasis resembles the physiological bone marrow vasculature, whereas in osteolytic bone metastasis the tumor vasculature was remodelled. Osteolytic cancer cells induce an arterial network in the stroma. Interestingly, in normal bone physiology, an arterial network is associated with hematopoietic stem cell (HSC) quiescence [40]. Our data shows that osteolytic cancer cells induce an arterial network, which might create a cancer cell niche beneficial to support cancer cell growth. The dormancy niche of HSC consist also of non-myelinating Schwann cells [41]. Schwann cells express various markers, among them Ng2 [42], and process latent TGF-β into active TGF-β in the bone marrow. Interestingly, we found Ng2 positive cells in osteolytic bone metastasis. These Ng2 positive cells could be Schwann cells providing active TGF-β to support cancer cell growth. Schwann cells also direct neurite outgrowth, and these might be the cells directing neurite ingrowth seen in osteolytic bone metastasis [43]. One key molecule in physiological artery specification is Sonic hedgehog (Shh), which stimulates Notch signaling and thereby inducing the arterial program [44]. In future it would be interesting to study the effects on osteolytic cancer growth upon disruption of the arterial network.

The predominant accumulation of smooth muscle cells/cancer-associated fibroblasts was interesting and evident only in osteolytic bone metastasis. The origin of these cells remains unclear. We have proven that these cells are of stromal origin and not cancer cells with a mesenchymal phenotype. Previously, it has been reported that in fibrosis fibroblasts can originate from endothelial cells [45]. Therefore, it is tempting to speculate that in the context of bone metastasis, cancer-associated fibroblasts are also derived from endothelial cells undergoing a mesenchymal transition. Interestingly, the upstream activator of the osteolytic stroma response with the highest activation score is Tgfβ, a potent inducer of endothelial to mesenchymal transition [45]. The growth support provided by mesenchymal cells to cancer cells beyond osteolytic cytokines and the expression of BMP/Wnt antagonists remains unclear. The overlap with an expression profile of αSMA positive cells yielded a list of interesting target genes that could be investigated as targets to interfere with osteolytic cancer cell growth at the bone/bone marrow site. Our study is in line with a previous study reporting that a cancer-associated fibroblast signature is overrepresented in bone metastases, as compared to lung, liver and brain metastases [46]. We observed cancer-associated fibroblasts (αSMA positive cells) in samples of human osteolytic bone metastasis. Fibroblast-like cells were previously described to mediate osteoclast-independent resorption, which could explain why these cells are present in osteolytic bone metastasis [47]. In human osteolytic bone metastasis cancer-associated fibroblasts and arteries are αSMA positive. Arteries were exclusively observed in osteolytic bone metastasis, whereas in osteoblastic bone metastasis vessels displayed a sinusoidal morphology.

Taken together, this study provides a systematic analysis of upstream regulators and altered pathways associated with the stroma response in osteolytic bone metastasis.

## MATERIALS AND METHODS

### Ethics statement

The Committee for Animal Experimentation and the Veterinary Authorities of the Canton of Bern, Switzerland approved the experimental animal protocols, anesthesia, surgical procedures and post-surgical analgesia (Permit Number: 15/07 and 6/10). Mice were housed in individual ventilated cages in strict accordance to the Swiss Guidelines for the Care and Use of Laboratory Animals. Autoclaved water and sterile mouse chow were provided *ad libitum*. For surgical manipulation, mice were anesthetized with a cocktail of medetomidin (1 mg/kg body weight), midazolam (10 mg/kg) and fentanyl (0.1 mg/kg). Post-operative analgesia with buprenorphine (0.1 mg/kg) was performed for 3 days following surgical intervention. Animals xenografted with human cancer cells were monitored for signs of pain, distress and loss of body weight. Development of bone lesions was followed by radiography at weekly intervals for PC-3, MDA-MB231, VCaP and 4T1.2 cells. At the experimental endpoint mice were sacrificed by CO_2_ euthanasia.

The Ethical Committee of the Canton of Bern, Switzerland, approved the overall study protocol and tissue collection from patients (Nr 06/03). A written informed consent was obtained from each patient.

### Cell culture

The osteolytic prostate cancer cell line PC-3 (ATCC CRL1435) was grown in DMEM and the prostate epithelial cell line Ep156T (kindly donated by V. Rotter, Department of Molecular Cell Biology, Weizmann Institute of Science, Rehovot, Israel) in modified MCDB-153 medium (WKS Diagnostics, Frankfurt am Main, Germany) supplemented with 5 ng/ml epidermal growth factor (Invitrogen), 10 nM R1881 and 50 ug/ml bovine pituitary extract (Gibco). The breast cancer cell line MDA-MB231 (ATCC-HTB-26) was grown in RPMI 1640 (Biochrom AG, Berlin, Germany) supplemented with sodium pyruvate. The mouse breast cancer cell line 4T1.2 (kindly donated by CR. Anderson, University of Melbourne, Melbourne) was grown in RPMI 1640 (Biochrom AG, Berlin, Germany). The human osteoinductive prostate cancer cell line VCaP (kindly donated by K. Pienta, University of Michigan, Ann Arbor) was grown in RPMI 1640 medium (Biochrom AG, Berlin, Germany).

All media were supplemented with 10% heat-inactivated fetal bovine serum gold (PAA, The Cell Culture Company).

### Titration of osteolytic cancer cells

A major difficulty in working with xenografts of osteolytic cancer cells is the rapidity by which bone lesions develop. The bone marrow ablation procedure, necessary to create space for implanting cancer cells in the marrow cavity, induces a wave of bone formation. The wave of bone formation normalizes within 2 weeks [48, 49]. It was necessary to determine the minimal inoculum of PC-3 cells able to generate an osteolytic reaction while allowing a sufficient lag-phase for the animals to recover to ensure that the stroma gene expression profile induced by the osteolytic cancer cells would not be influenced by the response due to marrow ablation. Constitutively firefly luciferase-expressing PC-3 cells were used to determine the onset of osteolytic lesions using different cell numbers. Single-cell suspensions of 2.500 (n=2), 5.000 (n=2), 10.000 (n=2), 20.000 (n=2), 50.000 (n=2) cells per 10ul phosphate-buffered saline (PBS) or 10ul PBS (n=2)(sham) were injected into the bone marrow cavity of the left tibia of mice. Intra-osseous tumor growth was monitored non-invasively in the living animals by bioluminescent imaging (BLI) at weekly intervals as previously described [50] using an ultrasensitive charge-coupled device (CCD) camera (NightOWL LB, Berthold Technologies, Bad Wilbad, Germany). The inoculation of decreasing numbers of cancer cells resulted in a delayed tumor onset but did not modify growth rate and tumor take. Based on these results, the *in vivo* experiment for RNA sequencing was performed using 2500 PC-3 cells (shown in Supplementary File).

### Bone metastasis mouse models

For intra-osseous xenografts, human prostate (PC-3 cell line) and breast (MDA-MB231 cell line) cancer cells with the capacity to induce an osteoclast reaction (osteolytic cancer cells) and human prostate cancer cells with capacity to induce an osteoblastic response (VCaP cell line) and immortalized, non-tumorigenic human prostate epithelial Ep156T cells were inoculated in the bone marrow cavity of the left tibia of male CB17 SCID mice as previously described [51]. Sham-operated animals (sham) and animals not subjected to surgery (intact) were used as controls. Development of bone lesions was monitored by radiography (MX-20, Faxitron X-Ray Cooperation, Edimex, Le Plessis, France). PC-3 and MDA-MB231 xenografts and Ep156T control animals were sacrificed after 33 days. Mice xenografted with VCaP and sham-operated were sacrificed after 72 days. Sham and intact animals were sacrificed at day 33 for PC-3 and MDA-MB231 xenografts and at day 72 for VCaP xenografts. Xenografted and control tibiae were used either for RNA isolation, immunohistochemistry, immunofluorescence or FACS.

As a systemic bone metastasis model, human osteolytic breast cancer cells (MDA-A12luc cells) were injected into the left cardiac ventricle and tumor growth was monitored using bioluminescent imaging (NightOWL LB, Berthold Technologies, Bad Wilbad, Germany) and radiography (MX-20, Faxitron X-Ray Cooperation, Edimex, Le Plessis, France). As a syngeneic bone metastasis model, mouse osteolytic breast cancer cells (4T1.2 cells) were intra-osseously injected in Balb/c mice. Sham-operated animals were used as controls.

### RNA extraction

Total RNA was isolated using TRIzol (Invitrogen) according to the manufacturer's instructions. RNA was quantified using the NanoDrop spectrophotometer (NanoDrop Technologies, Wilmington, Delaware, USA). The RNA quality was determined using RNA 6000 Nano Kit (Agilent Technologies), all RNA samples had a RNA integrity number (RIN score) more than 7. Total RNA without any further DNase treatment was used for RNA sequencing. The ratio of human to mouse RNA in the xenograft samples was determined by measuring 18S and both mouse and human β2-microglobulin, hypoxanthine phosphoribosyltransferase 1 and actin beta expression with RT-qPCR. For subsequent cDNA synthesis Trizol extracted RNA was cleaned up using a RNeasy Mini cleanup kit (Qiagen). The protocol also consisted of a DNase digestion step (Kit from Qiagen).

For FACS experiments, cells were directly sorted into lysis buffer. RNA was isolated using a RNA mini kit (Qiagen). Subsequently, RNA samples were amplified using a REPLI-g Single Cell RNA Library Kit.

For laser-captured micro-dissection analysis, RNA was isolated from specimens using a RNA mini kit (Qiagen).

### RNA sequencing and data analysis

For the identification of differentially expressed genes between the physiological bone/bone marrow stroma and that in established osteolytic bone metastasis, samples from 13 mice were analyzed. Tibiae from mice xenografted with osteolytic prostate (PC-3 cells (n=3)) and breast (MDA-MB231 cells (n=4)) cancer cells were analyzed. As controls, we analyzed tibiae xenografted with prostate epithelial non-tumorigenic cells (Ep156T)(n=3) and tibiae from control mice (intact tibiae with no intervention) (n=3). Previously, we have shown that all control samples - namely, intact bones, sham-operated bones and Ep156T xenografted bones - show a similar gene expression profile based on hierarchical clustering of our microarray data [19]. Only a single gene, namely MMP12, was differentially expressed between intact bones and sham-operated/Ep156T-xenografted bones. Therefore, in this study we included as controls intact bones and Ep156T xenografted bones.

As the stromal response could be tumor size-dependent, we selected RNA samples derived from a group of mice with tumors of comparable size for each group (PC3 and MDA-MB231 xenografts). We determined the percentage of human and mouse RNA by real-time PCR using species-specific primers of housekeeping genes. This measurement estimates the percentage of tumor (human transcripts) *versus* stroma (mouse transcripts). The selected samples for RNA sequencing had an average of 93% mouse transcripts (stroma) in PC-3 xenografts and 73% mouse transcripts (stroma) in MDA-MB231 xenografts (Supplementary File). RNA samples of (a) tibiae xenografted with PC-3 and MDA-MB231 cells, (b) intact bones and (c) tibiae xenografted with human non-tumorigenic prostate epithelial cells (Ep156T) were subsequently sequenced using an Illumina platform. First, we analyzed whether all reads can be unequivocally assigned to either human or mouse transcripts. In total, reads from 245 genes could not be unequivocally assigned to either human or mouse (Supplementary Table 4) and were therefore excluded from subsequent analyses. Furthermore, we analyzed whether genes were differentially expressed between Ep156T xenografted bones and intact bones. In total, 73 genes were differentially expressed, among which 68 genes were up-regulated and 5 genes were down-regulated (fold change > ±2, adjusted *p value* < 0.01) (Supplementary Table 5). The latter result indicated that the surgical manipulation with inoculation of human epithelial cells did not considerably alter the stroma gene expression.

### PC-3 xenografts

Paired-end high-throughput RNA sequencing of PC-3 xenografts, Ep156T xenografts and intact samples was performed on the Illumina/Solexa GAII platform (Illumina Inc, San Diego, CA, USA) by the ServiceXS Sequencing Facility (Leiden, Netherlands).

The Illumina mRNA sequencing sample preparation kit was used to process all RNA samples. In brief, after isolation of mRNA from total RNA using poly-T-oligo-attached magnetic beads, mRNA was fragmented (using divalent cations), which yielded an average size of 200 nucleotides (fragment library). Following cDNA synthesis, cDNA was ligated with adapters and amplified by PCR. The quality and yield was evaluated using a DNA 1000 kit (Agilent Technologies). cDNA fragments were hybridized onto an Illumina glass flow cell, fragments were amplified into a cluster using bridge amplification. 8pmol of DNA per sample was sequenced. Two sequencing reads of 75 cycles each using the Read 1 sequencing and Read 2 sequencing primers were performed using a glass flow cell. Sequencing files were generated in FastQ format.

#### Pre-processing

Image analysis, base calling, and quality check was performed with the Illumina Genome Analyzer data analysis pipeline RTA v1.8.80.0 and/or OLB v1.8 and CASACA v1.7.0.

FastQ files were filtered by removing reads with more than 20% of nucleotides having Phred quality scores less than 20 (probability of incorrect base call 1%).

#### Alignment of human reads to reference genome

Reads were mapped to the human genome hg19 (GCA_000001405.1) using the Bowtie aligner version 0.12.7 (http://bowtie.cbcb.umd.edu/).

#### Alignment of mouse reads to reference genome

Sequenced fragments that did not map to the human reference genome were paired by matching forward and reverse reads based on their IDs and then aligned to the Mus musculus reference genome (Mus_musculus.NCBIM37.60.dn) using TopHat version 1.4.0. Per gene (ftp://ftp.ensembl.org/pub/release-60/gtf/mus_musculus/) the amount of aligned reads was counted (https://htseq.readthedocs.io/en/release_0.9.1/history.html#version-0-4-5) and stored in count files.

### MDA-MB231 xenografts

The paired-end RNA sequencing was performed using an Illumina HiSeq 2000 sequencer. The paired-end mRNA-Seq reads were filtered using a Q22 Phred score as a minimum. Bases with Phred scores below this score were removed, only reads longer than 36 bases were retained. The reads were aligned to a combined transcriptome reference derived from draft versions mm9 as mouse reference and hg19 as human reference. This combined reference approach separates the dataset by mapping paired-end reads to genes of a specific organism using the aligner (BWA version 0.5.9). The approach used data from both human and mouse reads from the sequence read archieve (SRA), resulting in a mapping mismatch rate of 0.005% and 0.8% respectively.

Tophat version 1.4.0 and Bowtie version 0.12.7 align the separated reads to the mm9 (Mus_musculus.NCBIM37.60.dn) and hg19 (GCA_000001405.1) genome references. Per gene (ftp://ftp.ensembl.org/pub/release-60/gtf/mus_musculus/) the amount of aligned reads was counted (https://htseq.readthedocs.io/en/release_0.9.1/history.html#version-0-4-5) and stored in count files.

In PC-3 xenografts 2141 genes are differentially expressed compared to control bones, as opposed to 6476 differentially expressed genes in MDA-MB231 xenografts. This higher number of differentially expressed genes could be due to different sequencing depth. The different sequencing depths for PC-3 and MDA-MD231 samples occur due to different sequencing technology. In PC-3 samples (n=3) 6'730'384, 9'524'486 and 9'503'449 total reads overlapped with annotated genes, whereas in MDA-MB231 samples (n=4) 26'422'805, 12'982'791, 20'323'365 and 20'897'481 total reads overlapped with annotated genes. Furthermore, the statistical power to detect differentially expressed genes in MDA-MB231 xenografts compared to controls is higher due to 4 replicates as compared to three replicates for PC-3 xenografts and therefore, it is also likely that more differentially expressed genes are identified. Finally, MDA-MB231 tumors were more advanced (based on the measurement of the human to mouse ratio) when we isolated the RNA for sequencing. This could also explain why more stromal genes are differentially expressed as compared to PC-3 xenografts.

### Quantification of gene expression and identification of differentially expressed genes (DESeq2)

Differential gene expression was calculated from raw read counts using Deseq2 v. 1.6.1. (adjusted pvalue of <0.01). Gene expression differences were measured using log2 fold change. A gene was considered to be differentially expressed if it had a Benjamini-Hochberg adjusted P-value < 0.01.

### Principal component analysis (PCA) plot

The first two axes from a PCA plot in DESeq2 v. 1.6.1. were plotted to visualize the similarity of gene expression profiles among samples. A regularized log transformation was applied to the counts before PCA.

### Reverse transcription quantitative PCR (RT-qPCR)

First strand cDNA was synthesized using 2 ug of total RNA, 8U/μl MMLV Reverse Transcriptase, 10 ng/μl random primer, 0.4mM dNTPs and 1.6U/μl RNase inhibitor (all Promega, Wallisellen, Switzerland).

For laser-captured microdissection samples, cDNA was synthesized using SuperScript™ II Reverse Transcriptase (ThermoFisher Scientific).

mRNA expression was measured by real-time-qPCR using species-specific TaqMan gene expression assays. The mouse and human specific gene expression assays are listed in Supplementary Table 6. ABI-Prism 7500 Sequence Detection System (Applied Biosystems, Rotkreuz, Switzerland) was used to analyze the samples. Gene expression was either normalized to a panel of housekeeping genes (ACTB, B2M, HRPT1, 18S) to dermine the mouse *versus* human ratio or to β-2-microglobulin to validate differentially expressed target genes. RNA expression data were analyzed with two-tailed, unpaired t-test using GraphPad Prism version 6.0 (GraphPad Software Inc., La Jolla, USA).

### Immunohistochemistry and immunofluorescence

Immunohistochemical staining was performed on deparaffinized tissue sections with the primary antibodies listed in Supplementary Table 7. Antibodies were detected using horseradish peroxidase-conjugated biotin-streptavidin (GE Healthcare, Glattbrugg, Switzerland) or EnVision (Dako, Baar, Switzerland) systems. 3-Amino-9-ethyl-carbazole (AEC, Sigma) was used as a chromogen. Sections were counterstained with hematoxilin.

Immunofluorescence staining was performed on undecalcified tissue sections [52] with the primary and secondary antibodies listed in Supplementary Table 7. Sections were counter-stained with Dapi.

Samples of normal bone were obtained from patients with coxarthrosis. Prostate cancer bone metastasis samples were derived from iliac crest and femur, while breast cancer bone metastasis samples were from the humerus.

Quantification of stainings were performed using ImageJ.

### Labeling of arteries *in vivo*

Alexa Fluor 633 hydrazide (Invitrogen) was intra-venously injected in mice 5 minutes before euthanasia to label arteries [20]. Undecalcified frozen bone sections were prepared from Alexa Fluor 633 injected animals.

### FACS

We analyzed intact tibiae (n=5), sham-operated tibiae (n=5) and MDA-MB231 xenografted tibiae (n=5) to determine the number of MSCs. Tibiae were crushed and digested for 15min at 37 degrees at 110 revolutions per minute. The digestion solution was prepared in 1xPBS/0.5% BSA containing 1.25 mg/ml Collagenase II (Worthington), 1.25 mg/ml Collagenase IV (Sigma C5138) and 0.25 mg/ml deoxyribonuclease (Roche, 11284932001). To deplete hematopoietic cells, the cell suspensions were stained with Cd45- and Ter119-biotinylated antibodies and subsequently stained with anti-biotin beads to deplete Cd45 and Ter119 positive cells using MACS separation columns (130-042-401). Remaining cells were stained for Cd31, Sca1, Cd51, Cd140a and HLA-A2 (labels human cells). Cells were then analyzed using FACS Aria. MSCs were characterized by the following marker expression: Cd45-, Ter119-, Cd31- and Sca-1+, Cd51+, Cd140a+.

For the RNA expression analysis, we pooled MSCs sorted from either sham-operated tibiae or MDA-MB231 xenografted tibiae. Alpha SMA positive cells from intact (n=3) and MDA-MB231 xenografted (n=3) tibiae were sorted after depletion of hematopoietic cells. We pooled αSMA positive cells from either intact or MDA-MB231 xenografted tibiae and then analyzed the expression profile.

### Identification of key biological processes, pathways, up-stream regulators

Differentially expressed genes were analyzed for enriched gene ontology (GO) terms, enriched KEGG pathways (DAVID 6.7) and to create a functional protein network (STRING, 9.05; confidence score = 0.4). The upstream regulators were predicted by using Ingenuity Pathway Analysis software.

## SUPPLEMENTARY MATERIALS FIGURES AND TABLES














