# Supplementary material for: Osteolytic cancer cells induce vascular/axon guidance processes in the bone/bone marrow stroma
Source: Oncotarget. 2018 Jun 22;9(48):28877–96. doi: 10.18632/oncotarget.25608 (PMC6034746; doi:10.18632/oncotarget.25608)
Supplement: Supplementary file 1 [file oncotarget-09-28877-s001.pdf]

# Osteolytic cancer cells induce vascular/axon guidance processes in the bone/bone marrow stroma

## SUPPLEMENTARY MATERIALS

### Osteolytic cancer cells induce BMP and Wnt antagonist expression in the stroma of osteolytic bone metastasis

We used mouse-specific real time PCR primers to validate the differential expression of Wnt and Bmp antagonists in the stroma of osteolytic bone metastasis (Supplementary Figure 3A). In PC-3 xenografts, all Wnt antagonists highlighted by RNA sequencing, were confirmed by real time PCR to be significantly up-regulated in the stroma of osteolytic bone metastasis. The Bmp antagonists, except Sclerostin, were also significantly up-regulated in PC-3 xenografts. In MDA-MB231 xenografts, Wnt antagonists, Dkk2 and Dkk3, were significantly up-regulated, whereas Wnt antagonist, Dkk1, and Bmp antagonist, Sclerostin, were significantly down-regulated. The Wnt antagonist Frzb, and Bmp antagonists, Chordin and Noggin, remained unchanged. In the stroma of osteoblastic bone metastasis, all Bmp and Wnt antagonists remained unchanged (Supplementary Figure 3B).

### The stroma of osteolytic bone metastasis contains a higher number of MSCs than control bones

We quantified the MSC population in the stroma of one model of osteolytic bone metastasis, namely MDA-MB231 xenografted bones, by FACS analysis. In all analyses, we depleted Cd45 and Ter119 positive cells and then quantified MSCs ($Cd31^-$, $Sca1^+$, $Cd51^+$, $Cd140a^+$). Interestingly, the stroma of MDA-MB231 xenografted bones contained 5.7 times more MSCs than intact bones and 2.2 times more MSCs than sham-operated bones.

### In osteolytic bone metastasis blood vessels are distributed throughout the tumor

We stained osteolytic bone metastasis (PC-3 and MDA-MB231 xenografts), osteoblastic bone metastasis (VCaP xenografts) and control bones for various other endothelial cell markers (Cdh5, Cd31, Tie2 and Vegfr3) and for laminin (constituent of basement membranes) (Supplementary Figure 4). Cdh5, Cd31, laminin, Tie2 and Vegfr3 positive endothelial cells are found in the normal bone marrow, but also in the osteolytic and osteoblastic bone metastasis. The difference between osteolytic and osteoblastic bone metastasis was the different spatial distribution of Cdh5, Cd31, laminin, Tie2 and Vegfr3-labeled blood vessels; intra-tumoral in osteolytic bone metastasis and surrounding tumor areas and in close proximity to the bone surface in osteoblastic bone metastasis. Interestingly, the frequency of laminin positive cells was higher in MDA-MB231 and PC-3 xenografts as compared to VCaP xenografts. Also, there were more Tie2 positive cells in MDA-MD231 xenografts as compared to VCaP xenografts.

### Human osteolytic bone metastasis contain an arterial network as opposed to osteoblastic bone metastasis

We stained consecutive specimens from human osteolytic and osteoblastic bone metastasis for αSMA, Epcam and β3 tubulin (Supplementary Figure 5). In osteolytic bone metastasis αSMA positive stain arteries and scattered stromal cells. β3 tubulin positive cells are preferentially found in osteolytic bone metastasis in the stromal compartment.

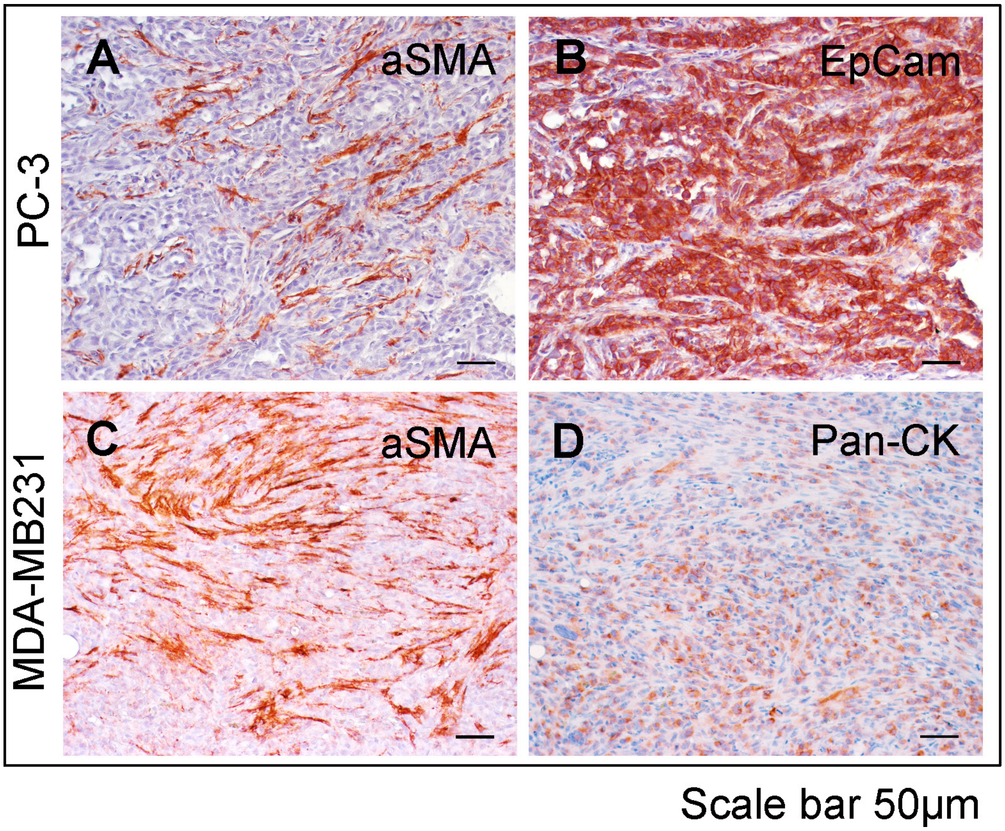


**Supplementary Figure 1: The smooth muscle marker (αSMA) protein expression is detected in non-epithelial elongated cells in the stroma of osteolytic bone metastasis.** Immunohistochemical detection of αSMA **(A)** and Epcam **(B)** in consecutive sections of PC-3 xenografts and αSMA **(C)** and Pan-cytokeratin **(D)** in consecutive sections of MDA-MB231 xenografts. Scale bar = 50 µm.

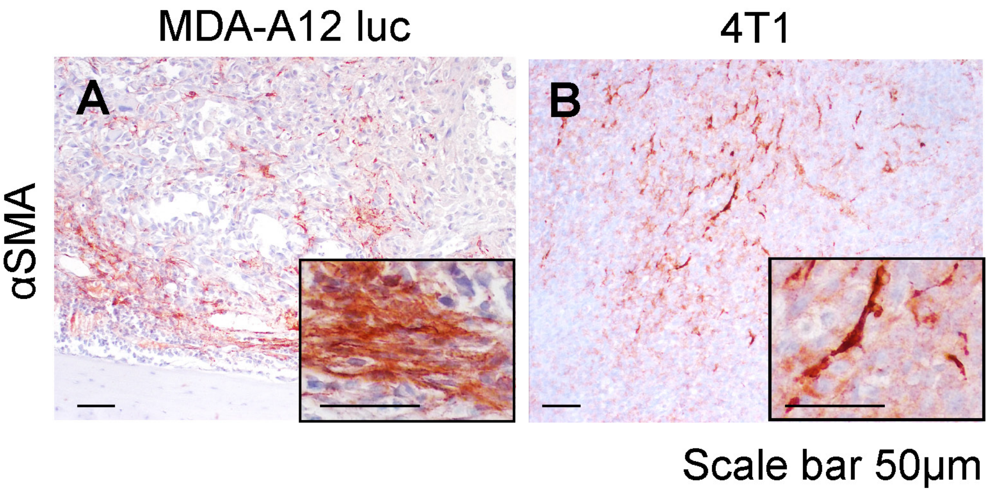


**Supplementary Figure 2: The smooth muscle marker (αSMA) is expressed in a systemic and syngeneic bone metastasis model.** Immunohistochemical detection of αSMA (**A** and **B)** in systemic (MDA-A12 cells injected intra-cardiac) and syngeneic (4T1.2 intra-osseous xenograft) bone metastasis model. Insets represent a higher magnification of selected areas. Scale bar = 50 μm.

**A**

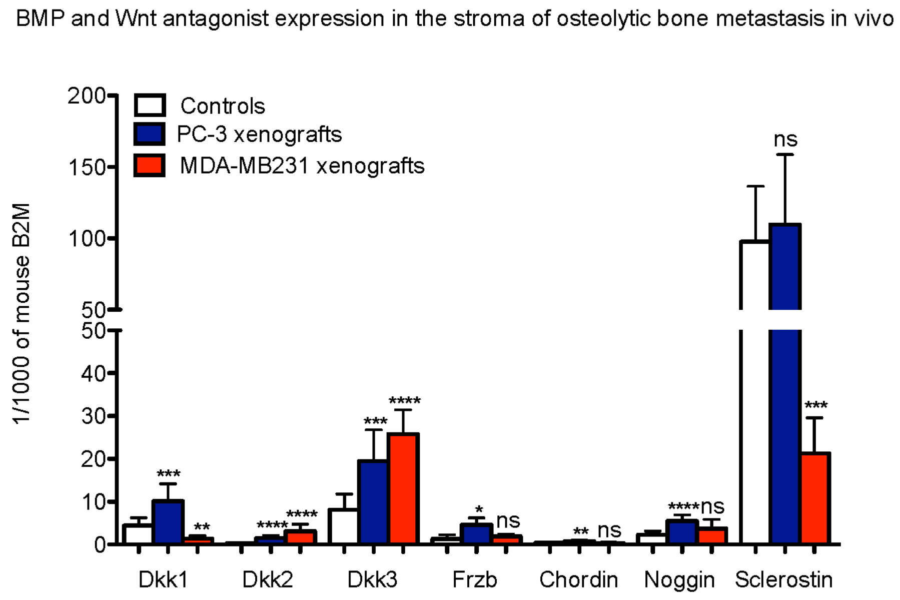


**B**

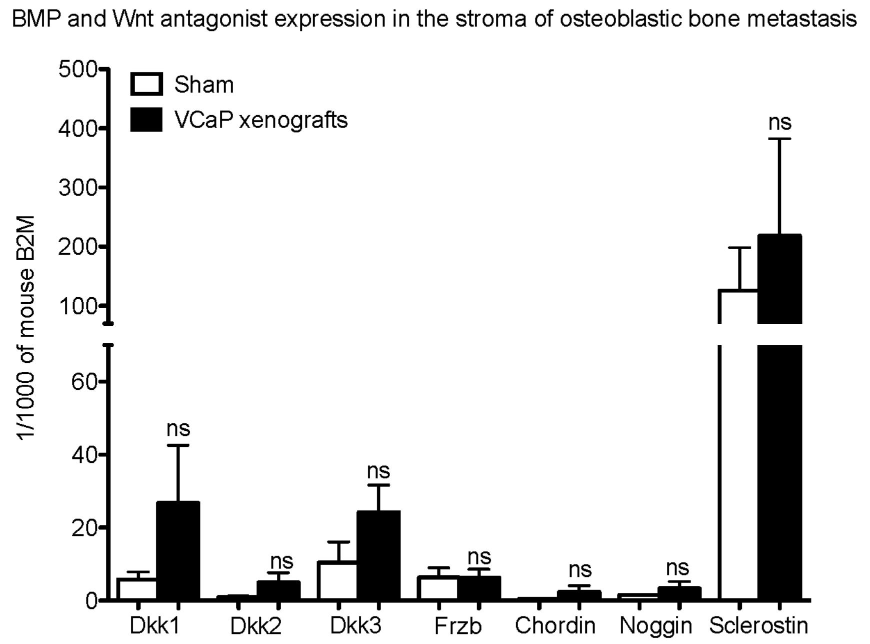


**Supplementary Figure 3: Bmp and Wnt antagonists are specifically expressed in osteolytic bone metastasis.** Fold change expression levels of *Dkk1*, *Dkk2*, *Dkk3*, *Frzb*, *Chordin*, *Noggin* and *Sclerostin* in **(A)** osteolytic (PC-3 and MDA-MB231 xenografts) and **(B)** osteoblastic (VCaP xenografts) bone metastasis compared to sham-operated animals. Values are shown as fold change (mean ± SD). $^{*}$, $P < 0.01$; $^{**}$, $P < 0.001$; $^{***}$, $P < 0.0001$; $^{****}$, $P < 0.0001$, ns - not significant.

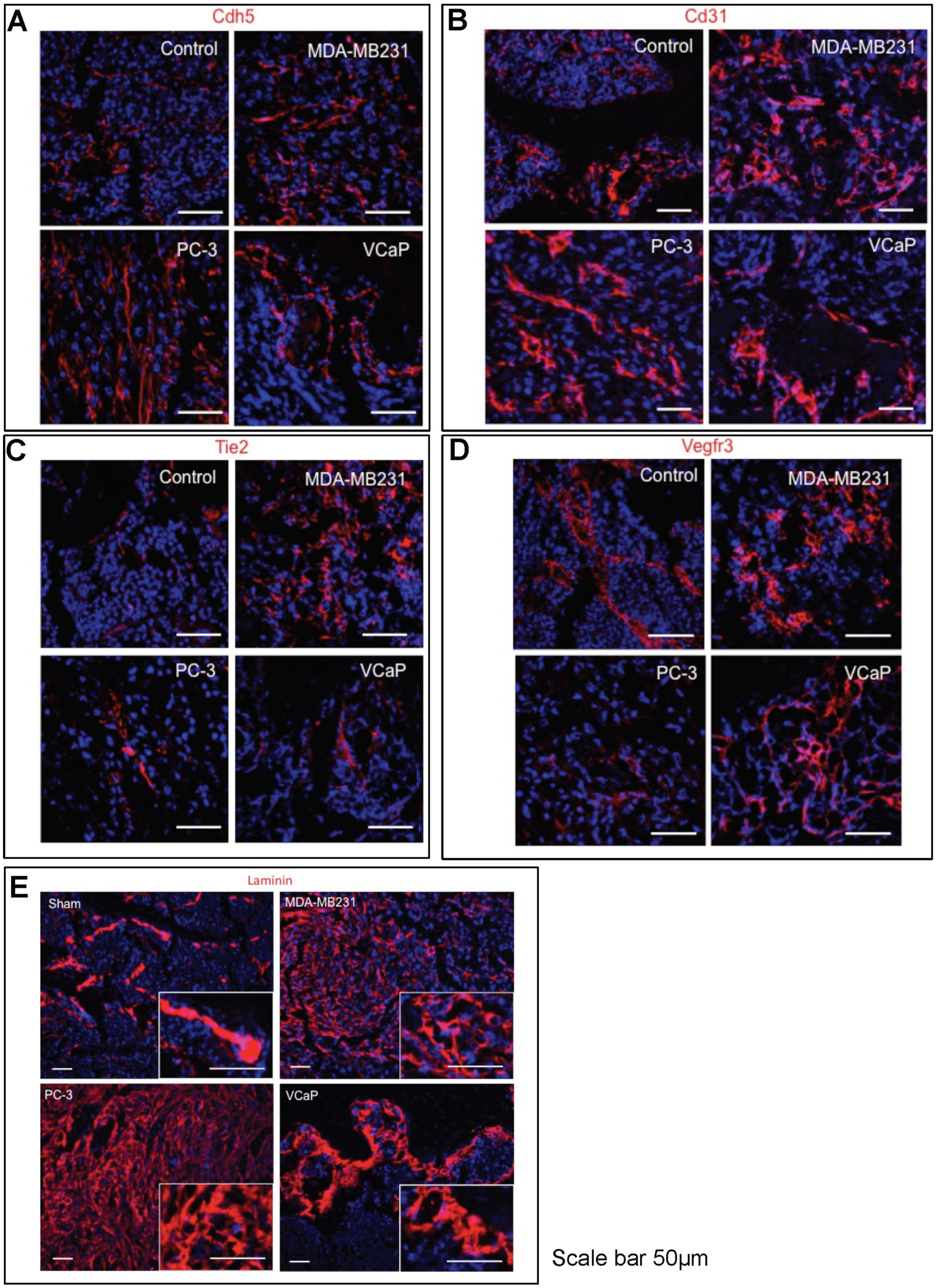


**Supplementary Figure 4: In osteolytic bone metastasis blood vessels are distributed throughout the tumor, in osteoblastic bone metastasis blood vessels are linked to bone forming surfaces.** Immunofluorescent detection of endothelial cell markers (Cdh5 **(A)**, Cd31 **(B)**, Tie2 **(C)** and Vegfr3 **(D)**) and for laminin (constituent of basement membranes) **(E)** in control bones, MDA-MB231 xenografts, PC-3 xenografts and VCaP xenografts. Scale bar 50 µm.

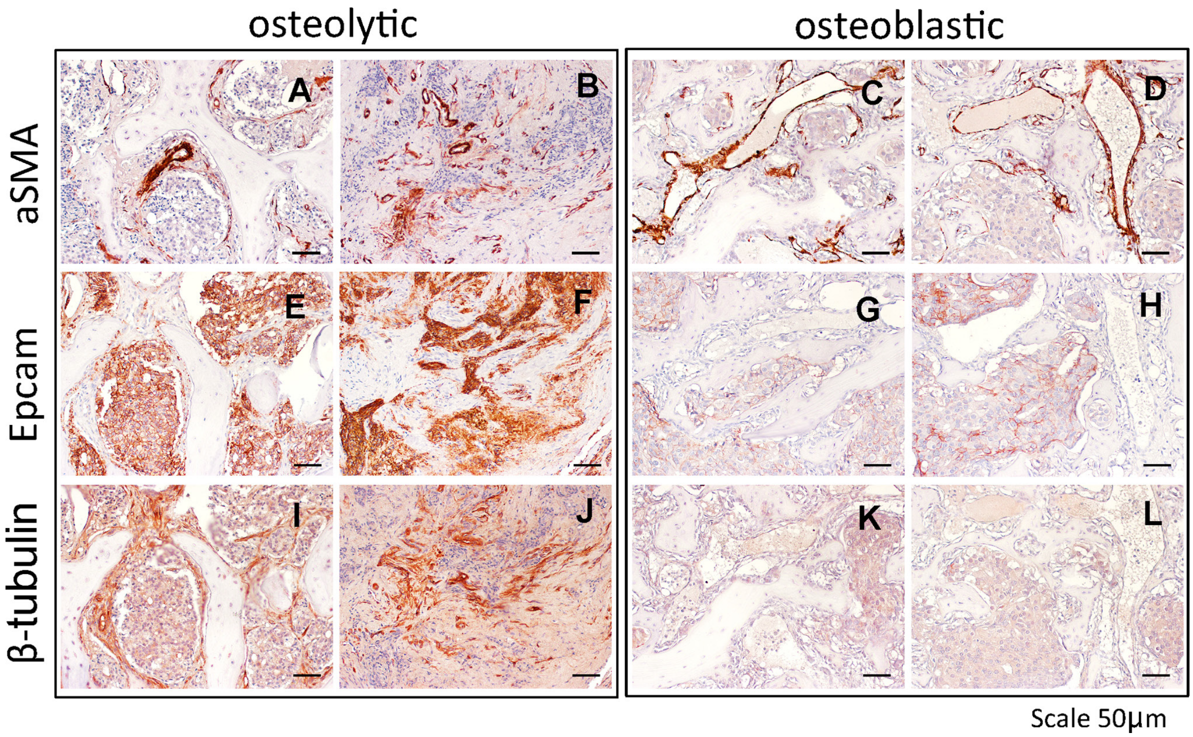


**Supplementary Figure 5: The stroma of human osteolytic bone metastases consist of an arterial network.** Immunohistochemical detection of αSMA **(A-D)**, Epcam **(E-H)** and β3 tubulin **(I-L)** in consecutive osteolytic (A, B, E, F, I and J) and osteoblastic bone metastasis (C, D, G, H, K and L) sections. Scale bar = 50 µm.

**Supplementary Table 1: List of differentially expressed genes in PC-3 xenografts**

See supplementary File 1

**Supplementary Table 2: List of differentially expressed genes in MDA-MB231 xenografts**

See supplementary File 2

**Supplementary Table 3: Differentially expressed genes common to both PC-3 and MDA-MB231 xenografts**

See supplementary File 3

**Supplementary Table 4: List of excluded genes showing species homology and therefore, cannot be clearly assigned to either human or mouse**

See supplementary File 4

**Supplementary Table 5: List of differentially expressed genes in Ep156T xenografted bones compared to intact bones**

See supplementary File 5

**Supplementary Table 6: List of TaqMan probes**

See supplementary File 6

**Supplementary Table 7: List of antibodies**

| *Immunohistochemistry/Immunofluorescence* | | | |
|---|---|---|---|
| **primary antibodies** | **company** | **cat. no.** | **clone** |
| Alexa Fluor 633 | Invitrogen | A10266 | |
| alpha SMA | Sigma Aldrich | A 2547 | 1A4 |
| Cd105 | R&D Systems | AF1320 | |
| Cd31 | BD Pharmingen | 553370 | MEC 13.3 |
| Cdh5 | Biolegend | 138005 | |
| Cy3 anti-ms | Jackson | 115-165-003 | |
| Cy3 anti-rat | Abcam | Ab6953 | |
| Cy3 anti-rbt | Jackson | 111-165-003 | |
| Endomucin | Sigma | HPA 005928 | |
| EpCAM | DAKO | M3525 | MOC-31 |
| Gap-43 | Millipore | AB5220 | |
| Laminin | Sigma Aldrich | L9393 | |
| Ng2 | Abcam | AB81104 | |
| panCK | DAKO | M0821 | MNF 116 |
| Pdgfrb | Abcam | Ab325570 | |
| Sca-1 | Biolegend | 122502 | E13-161.7 |
| Tie2 | Santa Cruz | SC-324 | |
| Tubulin beta III | Millipore | MAB1637 | Tu-20 |
| Vegfr3 | RD systems | AF743 | |

| *FACS* | | | |
|---|---|---|---|
| **primary antibodies** | **company** | **cat. no.** | **clone** |
| aSMA | Abcam | Ab197240 | E184 |
| Anti-biotin beads | Miltenyi | 130-090-489 | |
| Cd31-FITC | Biolegend | 102405 | 390 |
| Cd45 biotin/Tert119 biotin | Miltenyi | | |
| Cd51 | AbD Serotec | MCA2461A647T | |
| HLA-A2 | Biolegend | 343310 | BB7.2 |
| rbt IgG Isotype Ctrl. | Abcam | Ab199091 | |
| Sca1-PerCP | Biolegend | 108-124 | |
| V500 Streptavidin | eBioscience | 12-1401-81 | |

**Radiographs, RIN numbers of RNA samples and human/mouse ratio**

PC-3 xenografts at day 33

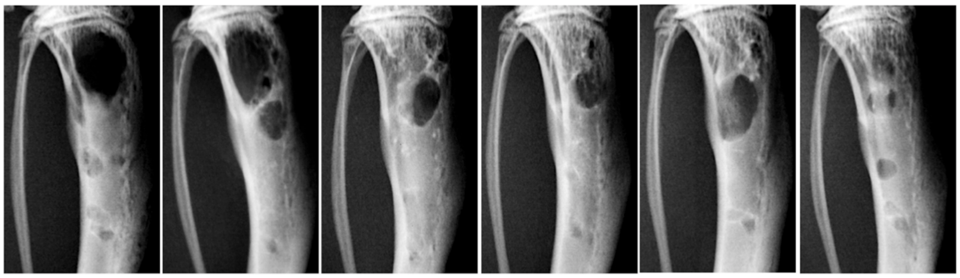

| m1 | m2 | m3 | m4 | m5 | m6 | **mouse number** |
|---|---|---|---|---|---|---|
| 8.7 | 7.8 | 8.6 | 8.7 | 7.3 | 6.1 | **RIN number** |
| 33 | 21 | 6 | 3 | 7 | 8 | **human RNA in %** |
| 67 | 79 | 94 | 97 | 93 | 92 | **mouse RNA in %** |
| no | no | yes | no | yes | yes | **RNA sequencing** |

MDA-MB231 xenografts at day 31

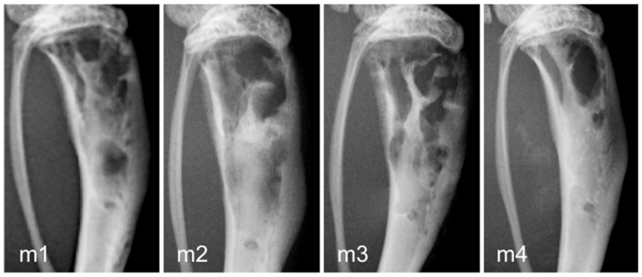


| m1 | m2 | m3 | m4 | **mouse number** |
|---|---|---|---|---|
| 8.6 | 9 | 8.6 | 8.7 | **RIN number** |
| 28 | 31 | 28 | 21 | **human RNA in %** |
| 72 | 69 | 72 | 79 | **mouse RNA in %** |
| yes | yes | yes | yes | **RNA sequencing** |

(*Continued*)

## Radiographs and RIN numbers of RNA samples from control groups at the experimental endpoint

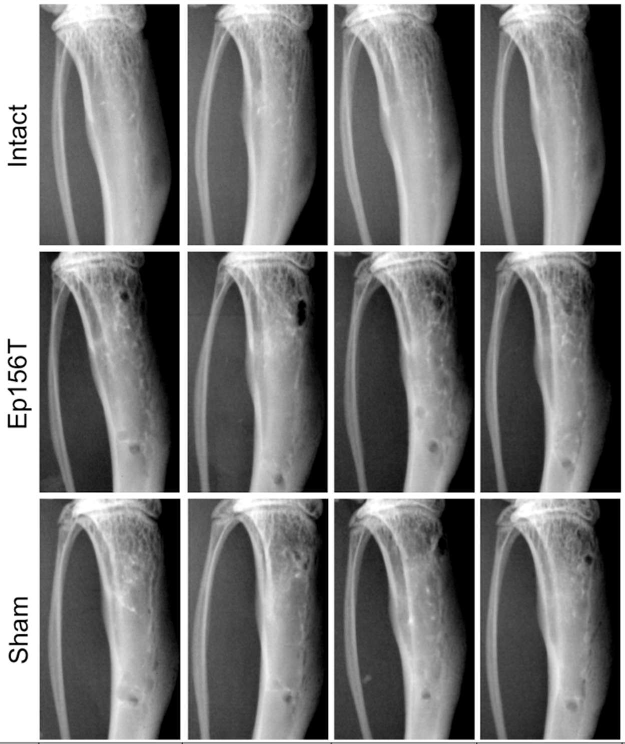


| | m1 | m2 | m3 | m4 | Mouse number |
|---|---|---|---|---|---|
| **Intact** | 8.7* | 7.8 | 9.2* | 9.2* | RIN number |
| **Ep156T** | 8.7* | 8.4* | 8.4 | 8.6* | RIN number |
| **Sham** | 7.6 | 8.9 | 8.8 | 9.2 | RIN number |
| * Samples send for sequencing | | | | | |

(*Continued*)

A. Summary of sequencing data (PC-3 xenografts and controls)

| | PC-3 | PC-3 | PC-3 | Ep156T | Ep156T | Ep156T | Intact | Intact | Intact |
|---|---|---|---|---|---|---|---|---|---|
| **lane yield (kilo bases)** | 1'598'718 | 2'465'410 | 2'480'841 | 4'028'432 | 4'159'577 | 4'146'445 | 4'971'421 | 3'394'920 | 3'956'748 |
| **total forward reads (raw data)** | 10'517'969 | 15'863'524 | 16'321'428 | 26'502'913 | 27'137'674 | 30'625'363 | 37'182'375 | 21'979'883 | 31'329'813 |
| **total reverse reads (raw data)** | 10'517'969 | 16'219'855 | 16'321'428 | 26'502'913 | 27'137'674 | 30'625'363 | 37'182'375 | 22'148'440 | 31'329'813 |
| **filtered forward reads** | 10'517'969 | 15'704'912 | 16'116'460 | 25'645'317 | 25'592'009 | 25'816'330 | 32'029'697 | 21'548'820 | 25'099'628 |
| **filtered reverse reads** | 10'517'969 | 15'704'912 | 16'116'460 | 25'645'317 | 25'592'009 | 25'818'820 | 32'032'819 | 21'550'863 | 25'102'140 |
| **filtered reads mated** | 9'325'823 | 13'755'384 | 14'030'838 | 24'520'355 | 24'610'289 | 24'690'494 | 30'708'769 | 20'373'501 | 24'106'306 |
| **aligned forward reads to HG19** | 718'295 | 1'118'470 | 1'159'312 | 512'755 | 460'318 | 655'259 | 604'508 | 493'592 | 377'454 |
| **aligned reverse reads to HG19** | 631'523 | 1'117'116 | 1'153'235 | 507'971 | 452'442 | 636'162 | 606'740 | 492'938 | 462'026 |
| **aligned paired-end reads to MM** | 8'669'883 | 12'650'246 | 13'134'739 | 23'330'151 | 23'330'151 | 22'986'314 | 29'286'936 | 18'992'356 | 23'190'572 |
| **aligned forward reads to HG19 (%)** | 6.83 | 7.12 | 7.19 | 2.00 | 1.80 | 2.54 | 1.89 | 2.29 | 1.50 |
| **aligned reverse reads to HG19 (%)** | 6.00 | 7.11 | 7.16 | 1.98 | 1.77 | 2.46 | 1.89 | 2.29 | 1.84 |
| **aligned paired-end reads to MM (%)** | 92.97 | 91.97 | 93.61 | 95.15 | 94.80 | 93.10 | 95.37 | 93.22 | 96.20 |

B. Summary of sequencing data (MDA-MB231 xenografts)

| | MDA-MB231 | MDA-MB231 | MDA-MB231 | MDA-MB231 |
|---|---|---|---|---|
| **lane yield (kilo bases)** | 12'069'093 | 6'215'286 | 10'147'908 | 9'187'215 |
| **total forward reads (raw data)** | 60'345'468 | 31'081'433 | 50'739'541 | 45'936'077 |
| **total reverse reads (raw data)** | 60'345'468 | 31'081'433 | 50'739'541 | 45'936'077 |
| **filtered forward reads** | 59'510'959 | 30'739'631 | 50'103'367 | 45'355'333 |
| **filtered reverse reads** | 57'667'328 | 29'924'490 | 48'638'310 | 44'050'129 |
| **filtered reads mated** | 75'347'314 | 37'937'452 | 58'932'476 | 60'656'734 |
| **aligned forward reads to HG19** | 14'769'519 | 8'138'883 | 14'155'538 | 10'144'292 |
| **aligned reverse reads to HG19** | 14'245'096 | 7'890'694 | 13'671'974 | 9'810'872 |
| **aligned paired-end reads to MM** | 32'584'105 | 16'296'015 | 24'849'549 | 26'454'533 |
| **aligned forward reads to HG19 (%)** | 24.82 | 26.38 | 28.25 | 22.37 |
| **aligned reverse reads to HG19 (%)** | 24.70 | 26.37 | 28.11 | 22.27 |
| **aligned paired-end reads to MM (%)** | 43.25 | 42.95 | 42.17 | 43.61 |

Lane yield, total forward/reverse reads and filtered forward/reverse reads are reported for each sample. The absolute numbers and percentages of forward/reverse reads aligning to HG19 (human reference genome) and paired-end reads aligning to the MM (mouse reference genome) are listed for each sample.

(*Continued*)

VCaP xenografts day 61

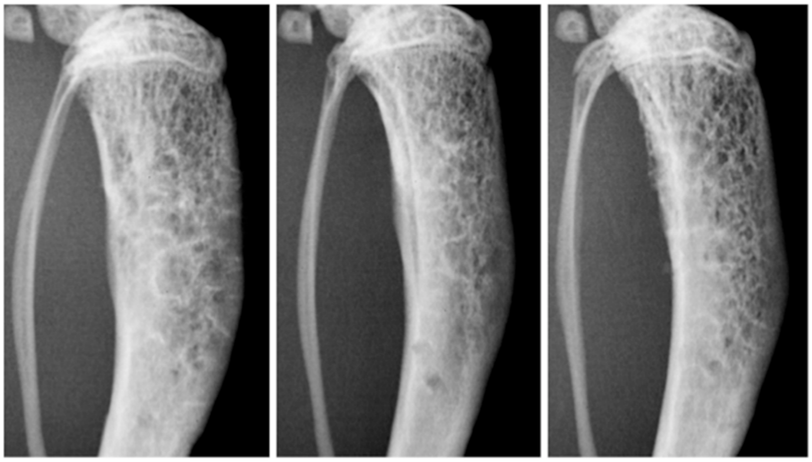

| m1 | m2 | m3 | **mouse number** |
|---|---|---|---|
| 33 | 38 | 37 | **human RNA in %** |
| 67 | 62 | 63 | **mouse RNA in %** |

**Representative radiograph of mouse tibia injected intra-osseous with 4T1.2 cells at the experimental endpoint (day 15)**

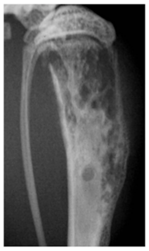

(*Continued*)

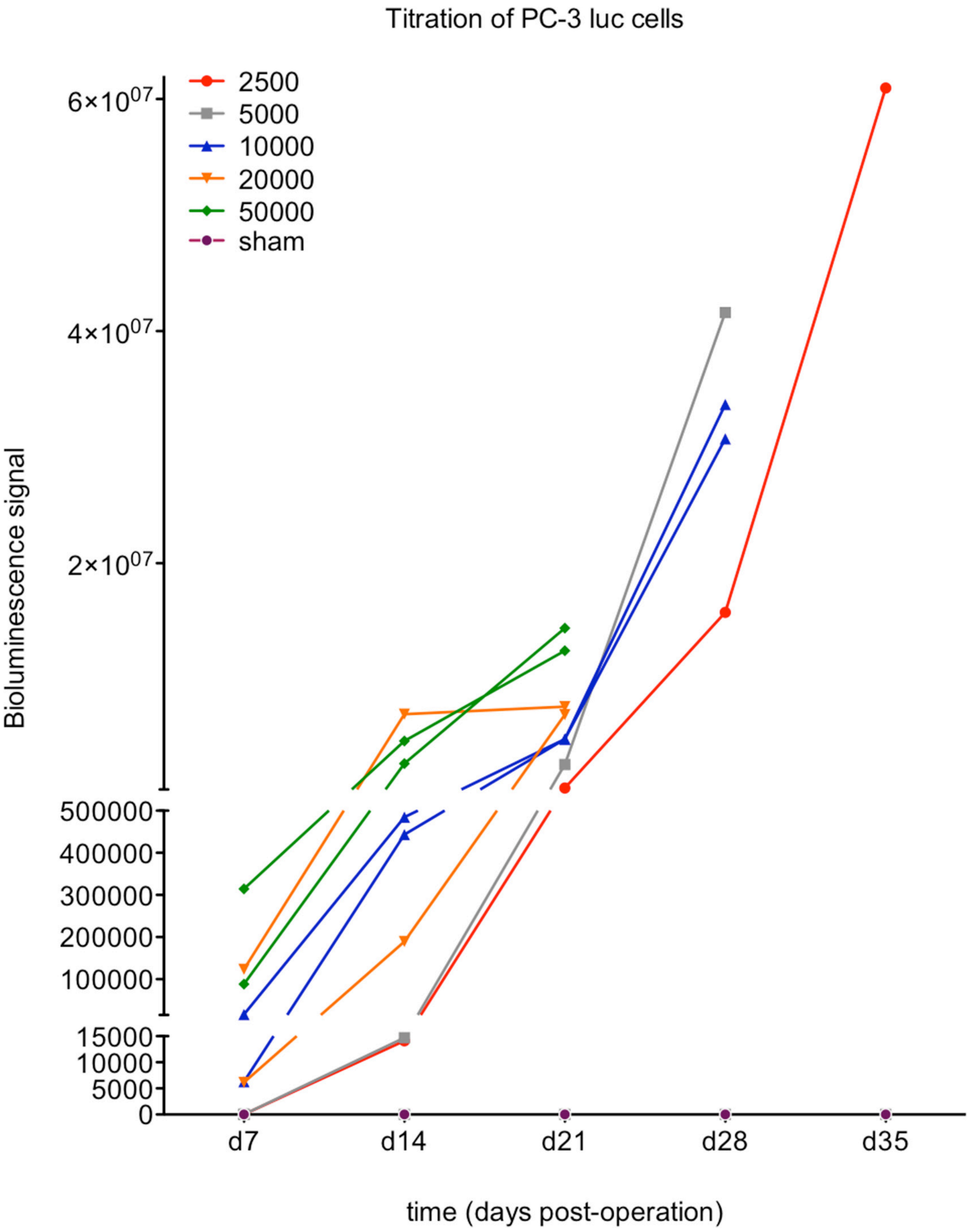


**Supplementary File: This file contains radiography images of xenografts and control bones, summary of RNA sequencing raw data and the data for the PC-3 titration experiment used to determine the minimum inoculum of cancer cells to allow sufficient time for wound healing based by marrow ablation.**
