# Supplementary material for: Osteolytic cancer cells induce vascular/axon guidance processes in the bone/bone marrow stroma
Source: Oncotarget. 2018 Jun 22;9(48):28877–96. doi: 10.18632/oncotarget.25608 (PMC6034746; doi:10.18632/oncotarget.25608)
Supplement: Supplementary file 2 [file oncotarget-09-28877-s002.docx]

**Table S1.**

| id | log2FoldChange | FC | padj | SYMBOL | GENENAME |
| --- | --- | --- | --- | --- | --- |
| ENSMUSG00000090007 | 7.10 | 136.75 | 3.23E-32 | NA | NA |
| ENSMUSG00000081834 | 5.29 | 39.03 | 9.33E-12 | NA | NA |
| ENSMUSG00000022156 | 4.44 | 21.73 | 4.12E-08 | Gzme | granzyme E |
| ENSMUSG00000059256 | 4.32 | 19.98 | 3.91E-09 | Gzmd | granzyme D |
| ENSMUSG00000020581 | 4.31 | 19.79 | 8.96E-13 | Agr2 | anterior gradient 2 |
| ENSMUSG00000026413 | 3.95 | 15.41 | 1.90E-10 | Pkp1 | plakophilin 1 |
| ENSMUSG00000089547 | 3.75 | 13.47 | 1.00E-05 | NA | NA |
| ENSMUSG00000021381 | 3.68 | 12.85 | 3.31E-05 | Barx1 | BarH-like homeobox 1 |
| ENSMUSG00000062235 | 3.67 | 12.68 | 1.55E-05 | NA | NA |
| ENSMUSG00000004892 | 3.40 | 10.59 | 2.09E-12 | Bcan | brevican |
| ENSMUSG00000079186 | 3.37 | 10.31 | 4.54E-06 | Gzmc | granzyme C |
| ENSMUSG00000079015 | 3.36 | 10.24 | 2.37E-04 | Serpina1c | serine (or cysteine) peptidase inhibitor, clade A, member 1C |
| ENSMUSG00000046856 | 3.20 | 9.19 | 2.77E-04 | Gpr1 | G protein-coupled receptor 1 |
| ENSMUSG00000089600 | 3.09 | 8.49 | 7.71E-04 | NA | NA |
| ENSMUSG00000071178 | 2.96 | 7.79 | 6.79E-04 | Serpina1b | serine (or cysteine) preptidase inhibitor, clade A, member 1B |
| ENSMUSG00000060240 | 2.86 | 7.26 | 1.22E-04 | Cend1 | cell cycle exit and neuronal differentiation 1 |
| ENSMUSG00000084277 | 2.84 | 7.18 | 1.15E-04 | NA | NA |
| ENSMUSG00000035352 | 2.82 | 7.07 | 3.24E-06 | Ccl12 | chemokine (C-C motif) ligand 12 |
| ENSMUSG00000042254 | 2.79 | 6.90 | 4.16E-04 | Cilp | cartilage intermediate layer protein, nucleotide pyrophosphohydrolase |
| ENSMUSG00000078949 | 2.73 | 6.63 | 4.73E-05 | R3hdml | R3H domain containing-like |
| ENSMUSG00000074978 | 2.71 | 6.54 | 1.47E-05 | NA | NA |
| ENSMUSG00000050663 | 2.71 | 6.53 | 8.02E-04 | Trhde | TRH-degrading enzyme |
| ENSMUSG00000002664 | 2.69 | 6.44 | 1.65E-03 | Pspn | persephin |
| ENSMUSG00000068246 | 2.67 | 6.38 | 1.64E-05 | Apol9b | apolipoprotein L 9b |
| ENSMUSG00000017943 | 2.66 | 6.32 | 9.85E-04 | Gdap1l1 | ganglioside-induced differentiation-associated protein 1-like 1 |
| ENSMUSG00000024173 | 2.64 | 6.25 | 2.82E-07 | Tpsab1 | tryptase alpha/beta 1 |
| ENSMUSG00000047428 | 2.62 | 6.14 | 4.17E-05 | Dlk2 | delta-like 2 homolog (Drosophila) |
| ENSMUSG00000021095 | 2.59 | 6.00 | 1.85E-03 | Gsc | goosecoid homeobox |
| ENSMUSG00000026343 | 2.55 | 5.87 | 1.07E-03 | Gpr39 | G protein-coupled receptor 39 |
| ENSMUSG00000049691 | 2.55 | 5.87 | 3.15E-03 | Nkx3-2 | NK3 homeobox 2 |
| ENSMUSG00000074566 | 2.53 | 5.78 | 8.09E-03 | NA | NA |
| ENSMUSG00000049556 | 2.53 | 5.76 | 9.61E-07 | Lingo1 | leucine rich repeat and Ig domain containing 1 |
| ENSMUSG00000048706 | 2.50 | 5.67 | 1.58E-06 | Lurap1l | leucine rich adaptor protein 1-like |
| ENSMUSG00000033825 | 2.50 | 5.67 | 9.10E-06 | Tpsb2 | tryptase beta 2 |
| ENSMUSG00000089022 | 2.48 | 5.60 | 4.64E-04 | NA | NA |
| ENSMUSG00000065487 | 2.47 | 5.53 | 2.03E-03 | NA | NA |
| ENSMUSG00000009185 | 2.46 | 5.51 | 1.88E-05 | Ccl8 | chemokine (C-C motif) ligand 8 |
| ENSMUSG00000047562 | 2.46 | 5.50 | 2.95E-03 | Mmp10 | matrix metallopeptidase 10 |
| ENSMUSG00000050288 | 2.43 | 5.38 | 1.17E-05 | Fzd2 | frizzled homolog 2 (Drosophila) |
| ENSMUSG00000006538 | 2.43 | 5.38 | 1.07E-05 | Ihh | Indian hedgehog |
| ENSMUSG00000072674 | 2.42 | 5.36 | 8.12E-03 | NA | NA |
| ENSMUSG00000001168 | 2.42 | 5.36 | 8.55E-03 | Oas1h | 2'-5' oligoadenylate synthetase 1H |
| ENSMUSG00000030519 | 2.41 | 5.31 | 8.29E-05 | Apba2 | amyloid beta (A4) precursor protein-binding, family A, member 2 |
| ENSMUSG00000065592 | 2.40 | 5.28 | 5.79E-03 | NA | NA |
| ENSMUSG00000005952 | 2.39 | 5.25 | 2.33E-03 | Trpv1 | transient receptor potential cation channel, subfamily V, member 1 |
| ENSMUSG00000035131 | 2.37 | 5.15 | 1.48E-05 | Brinp3 | bone morphogenetic protein/retinoic acid inducible neural specific 3 |
| ENSMUSG00000075707 | 2.36 | 5.14 | 4.63E-04 | Dio3 | deiodinase, iodothyronine type III |
| ENSMUSG00000050505 | 2.36 | 5.14 | 6.06E-03 | Pcdh20 | protocadherin 20 |
| ENSMUSG00000072849 | 2.34 | 5.07 | 6.10E-03 | Serpina1e | serine (or cysteine) peptidase inhibitor, clade A, member 1E |
| ENSMUSG00000028989 | 2.34 | 5.05 | 6.10E-08 | Angptl7 | angiopoietin-like 7 |
| ENSMUSG00000031274 | 2.33 | 5.04 | 6.46E-05 | Col4a5 | collagen, type IV, alpha 5 |
| ENSMUSG00000031849 | 2.31 | 4.94 | 4.69E-03 | Comp | cartilage oligomeric matrix protein |
| ENSMUSG00000026697 | 2.30 | 4.92 | 1.25E-06 | Myoc | myocilin |
| ENSMUSG00000018822 | 2.29 | 4.88 | 3.45E-06 | Sfrp5 | secreted frizzled-related sequence protein 5 |
| ENSMUSG00000043501 | 2.27 | 4.82 | 3.61E-05 | Lgals2 | lectin, galactose-binding, soluble 2 |
| ENSMUSG00000031760 | 2.27 | 4.81 | 2.14E-08 | Mt3 | metallothionein 3 |
| ENSMUSG00000087186 | 2.26 | 4.79 | 3.96E-03 | NA | NA |
| ENSMUSG00000006403 | 2.26 | 4.77 | 7.37E-14 | Adamts4 | a disintegrin-like and metallopeptidase (reprolysin type) with thrombospondin type 1 motif, 4 |
| ENSMUSG00000032291 | 2.25 | 4.76 | 5.89E-04 | Crabp1 | cellular retinoic acid binding protein I |
| ENSMUSG00000030732 | 2.24 | 4.73 | 2.81E-04 | Chrdl2 | chordin-like 2 |
| ENSMUSG00000047261 | 2.24 | 4.71 | 1.05E-03 | Gap43 | growth associated protein 43 |
| ENSMUSG00000029348 | 2.23 | 4.69 | 3.07E-06 | Asphd2 | aspartate beta-hydroxylase domain containing 2 |
| ENSMUSG00000017724 | 2.19 | 4.56 | 5.16E-10 | Etv4 | ets variant gene 4 (E1A enhancer binding protein, E1AF) |
| ENSMUSG00000081520 | 2.18 | 4.52 | 3.09E-03 | NA | NA |
| ENSMUSG00000044006 | 2.17 | 4.50 | 3.32E-03 | Cilp2 | cartilage intermediate layer protein 2 |
| ENSMUSG00000043913 | 2.15 | 4.44 | 4.12E-04 | Ccdc60 | coiled-coil domain containing 60 |
| ENSMUSG00000079105 | 2.15 | 4.44 | 5.86E-03 | C7 | complement component 7 |
| ENSMUSG00000052485 | 2.13 | 4.39 | 8.12E-03 | Tmem171 | transmembrane protein 171 |
| ENSMUSG00000026051 | 2.13 | 4.36 | 1.32E-03 | 1500015O10Rik | RIKEN cDNA 1500015O10 gene |
| ENSMUSG00000025784 | 2.12 | 4.36 | 2.74E-03 | Clec3b | C-type lectin domain family 3, member b |
| ENSMUSG00000002266 | 2.12 | 4.36 | 9.01E-03 | Zim1 | zinc finger, imprinted 1 |
| ENSMUSG00000031538 | 2.12 | 4.36 | 7.08E-09 | Plat | plasminogen activator, tissue |
| ENSMUSG00000027670 | 2.09 | 4.27 | 2.04E-08 | Ocstamp | osteoclast stimulatory transmembrane protein |
| ENSMUSG00000028167 | 2.07 | 4.21 | 3.02E-05 | Bdh2 | 3-hydroxybutyrate dehydrogenase, type 2 |
| ENSMUSG00000046182 | 2.06 | 4.17 | 5.42E-04 | Gsg1l | GSG1-like |
| ENSMUSG00000031070 | 2.06 | 4.17 | 8.70E-05 | Mrgprf | MAS-related GPR, member F |
| ENSMUSG00000028040 | 2.06 | 4.16 | 6.46E-05 | Efna4 | ephrin A4 |
| ENSMUSG00000088788 | 2.05 | 4.15 | 8.63E-03 | NA | NA |
| ENSMUSG00000040164 | 2.05 | 4.13 | 2.09E-04 | Kcns1 | K+ voltage-gated channel, subfamily S, 1 |
| ENSMUSG00000022382 | 2.04 | 4.12 | 1.38E-05 | Wnt7b | wingless-related MMTV integration site 7B |
| ENSMUSG00000072528 | 2.04 | 4.11 | 9.73E-03 | NA | NA |
| ENSMUSG00000030310 | 2.04 | 4.11 | 1.05E-04 | Slc6a1 | solute carrier family 6 (neurotransmitter transporter, GABA), member 1 |
| ENSMUSG00000020723 | 2.03 | 4.09 | 1.33E-03 | Cacng4 | calcium channel, voltage-dependent, gamma subunit 4 |
| ENSMUSG00000040280 | 2.02 | 4.06 | 3.12E-06 | Ndufa4l2 | NADH dehydrogenase (ubiquinone) 1 alpha subcomplex, 4-like 2 |
| ENSMUSG00000081249 | 2.02 | 4.05 | 3.15E-06 | NA | NA |
| ENSMUSG00000039476 | 2.01 | 4.04 | 8.11E-08 | Prrx2 | paired related homeobox 2 |
| ENSMUSG00000022203 | 2.01 | 4.02 | 1.14E-13 | Efs | embryonal Fyn-associated substrate |
| ENSMUSG00000047676 | 2.01 | 4.02 | 5.61E-06 | NA | NA |
| ENSMUSG00000018126 | 2.00 | 4.01 | 8.10E-04 | Baiap2l2 | BAI1-associated protein 2-like 2 |
| ENSMUSG00000033595 | 1.99 | 3.98 | 2.81E-03 | Lgi3 | leucine-rich repeat LGI family, member 3 |
| ENSMUSG00000022900 | 1.99 | 3.97 | 4.99E-03 | Ildr1 | immunoglobulin-like domain containing receptor 1 |
| ENSMUSG00000049551 | 1.99 | 3.97 | 5.90E-07 | Fzd9 | frizzled homolog 9 (Drosophila) |
| ENSMUSG00000037962 | 1.99 | 3.96 | 4.02E-03 | Fam101a | family with sequence similarity 101, member A |
| ENSMUSG00000028445 | 1.98 | 3.94 | 3.64E-07 | Enho | energy homeostasis associated |
| ENSMUSG00000037686 | 1.97 | 3.93 | 6.42E-10 | Aspg | asparaginase homolog (S. cerevisiae) |
| ENSMUSG00000015852 | 1.97 | 3.92 | 3.48E-04 | Fcrls | Fc receptor-like S, scavenger receptor |
| ENSMUSG00000083679 | 1.96 | 3.88 | 1.93E-06 | NA | NA |
| ENSMUSG00000033327 | 1.95 | 3.87 | 1.05E-04 | Tnxb | tenascin XB |
| ENSMUSG00000038932 | 1.95 | 3.87 | 9.81E-04 | Tcfl5 | transcription factor-like 5 (basic helix-loop-helix) |
| ENSMUSG00000037735 | 1.95 | 3.86 | 7.91E-03 | NA | NA |
| ENSMUSG00000036904 | 1.95 | 3.85 | 2.03E-04 | Fzd8 | frizzled homolog 8 (Drosophila) |
| ENSMUSG00000030606 | 1.94 | 3.84 | 5.92E-07 | Hapln3 | hyaluronan and proteoglycan link protein 3 |
| ENSMUSG00000057751 | 1.94 | 3.84 | 6.91E-04 | Megf6 | multiple EGF-like-domains 6 |
| ENSMUSG00000082223 | 1.94 | 3.84 | 1.27E-04 | NA | NA |
| ENSMUSG00000026259 | 1.94 | 3.84 | 1.89E-08 | Ngef | neuronal guanine nucleotide exchange factor |
| ENSMUSG00000061100 | 1.94 | 3.84 | 8.48E-03 | Retnla | resistin like alpha |
| ENSMUSG00000056174 | 1.93 | 3.81 | 6.35E-05 | Col8a2 | collagen, type VIII, alpha 2 |
| ENSMUSG00000036912 | 1.93 | 3.80 | 4.99E-03 | Piwil4 | piwi-like RNA-mediated gene silencing 4 |
| ENSMUSG00000027224 | 1.92 | 3.79 | 1.45E-03 | Duoxa1 | dual oxidase maturation factor 1 |
| ENSMUSG00000035226 | 1.90 | 3.74 | 6.10E-04 | Rims4 | regulating synaptic membrane exocytosis 4 |
| ENSMUSG00000035172 | 1.90 | 3.74 | 4.01E-06 | Plekhh3 | pleckstrin homology domain containing, family H (with MyTH4 domain) member 3 |
| ENSMUSG00000032492 | 1.90 | 3.74 | 1.99E-07 | Pth1r | parathyroid hormone 1 receptor |
| ENSMUSG00000075602 | 1.89 | 3.71 | 2.56E-05 | Ly6a | lymphocyte antigen 6 complex, locus A |
| ENSMUSG00000056481 | 1.89 | 3.71 | 5.29E-05 | Cd248 | CD248 antigen, endosialin |
| ENSMUSG00000023046 | 1.89 | 3.70 | 2.29E-05 | Igfbp6 | insulin-like growth factor binding protein 6 |
| ENSMUSG00000032997 | 1.88 | 3.67 | 7.83E-08 | Chpf | chondroitin polymerizing factor |
| ENSMUSG00000001473 | 1.87 | 3.67 | 7.01E-10 | Tubb6 | tubulin, beta 6 class V |
| ENSMUSG00000040703 | 1.87 | 3.66 | 5.52E-05 | Cyp2s1 | cytochrome P450, family 2, subfamily s, polypeptide 1 |
| ENSMUSG00000005958 | 1.87 | 3.66 | 1.61E-07 | Ephb3 | Eph receptor B3 |
| ENSMUSG00000078311 | 1.87 | 3.65 | 8.52E-05 | NA | NA |
| ENSMUSG00000072941 | 1.87 | 3.65 | 5.80E-03 | Sod3 | superoxide dismutase 3, extracellular |
| ENSMUSG00000056596 | 1.86 | 3.64 | 7.42E-05 | Trnp1 | TMF1-regulated nuclear protein 1 |
| ENSMUSG00000074676 | 1.85 | 3.61 | 8.39E-07 | Foxs1 | forkhead box S1 |
| ENSMUSG00000086409 | 1.84 | 3.59 | 2.83E-05 | NA | NA |
| ENSMUSG00000007594 | 1.84 | 3.59 | 3.85E-04 | Hapln4 | hyaluronan and proteoglycan link protein 4 |
| ENSMUSG00000062380 | 1.84 | 3.59 | 9.70E-05 | Tubb3 | tubulin, beta 3 class III |
| ENSMUSG00000027978 | 1.84 | 3.59 | 7.41E-03 | Prss12 | protease, serine 12 neurotrypsin (motopsin) |
| ENSMUSG00000043068 | 1.83 | 3.57 | 2.11E-03 | Fam89a | family with sequence similarity 89, member A |
| ENSMUSG00000029675 | 1.83 | 3.56 | 6.16E-08 | Eln | elastin |
| ENSMUSG00000016763 | 1.82 | 3.53 | 1.94E-05 | Scube1 | signal peptide, CUB domain, EGF-like 1 |
| ENSMUSG00000027386 | 1.82 | 3.52 | 6.22E-03 | Fbln7 | fibulin 7 |
| ENSMUSG00000003477 | 1.80 | 3.48 | 7.07E-03 | Inmt | indolethylamine N-methyltransferase |
| ENSMUSG00000001657 | 1.80 | 3.48 | 1.66E-03 | Hoxc8 | homeobox C8 |
| ENSMUSG00000024871 | 1.80 | 3.48 | 1.31E-04 | Doc2g | double C2, gamma |
| ENSMUSG00000000126 | 1.80 | 3.47 | 1.81E-04 | Wnt9a | wingless-type MMTV integration site 9A |
| ENSMUSG00000000567 | 1.79 | 3.46 | 3.06E-04 | Sox9 | SRY (sex determining region Y)-box 9 |
| ENSMUSG00000025407 | 1.79 | 3.46 | 2.45E-05 | Gli1 | GLI-Kruppel family member GLI1 |
| ENSMUSG00000010830 | 1.79 | 3.45 | 8.84E-08 | Kdelr3 | KDEL (Lys-Asp-Glu-Leu) endoplasmic reticulum protein retention receptor 3 |
| ENSMUSG00000061486 | 1.78 | 3.44 | 1.07E-03 | NA | NA |
| ENSMUSG00000020829 | 1.78 | 3.44 | 1.92E-06 | Slc46a1 | solute carrier family 46, member 1 |
| ENSMUSG00000049097 | 1.78 | 3.44 | 8.35E-03 | Ankrd34a | ankyrin repeat domain 34A |
| ENSMUSG00000032968 | 1.78 | 3.44 | 2.08E-04 | Inha | inhibin alpha |
| ENSMUSG00000029603 | 1.78 | 3.43 | 3.71E-03 | Dtx1 | deltex 1 homolog (Drosophila) |
| ENSMUSG00000016995 | 1.78 | 3.43 | 6.41E-04 | Matn4 | matrilin 4 |
| ENSMUSG00000001435 | 1.78 | 3.43 | 4.17E-09 | Col18a1 | collagen, type XVIII, alpha 1 |
| ENSMUSG00000083757 | 1.77 | 3.42 | 2.62E-04 | NA | NA |
| ENSMUSG00000006931 | 1.77 | 3.41 | 8.85E-08 | Leprel4 | leprecan-like 4 |
| ENSMUSG00000006369 | 1.76 | 3.39 | 2.80E-07 | Fbln1 | fibulin 1 |
| ENSMUSG00000030605 | 1.76 | 3.39 | 1.73E-05 | Mfge8 | milk fat globule-EGF factor 8 protein |
| ENSMUSG00000025150 | 1.76 | 3.39 | 9.26E-08 | Cbr2 | carbonyl reductase 2 |
| ENSMUSG00000020473 | 1.76 | 3.38 | 1.21E-05 | Aebp1 | AE binding protein 1 |
| ENSMUSG00000049721 | 1.76 | 3.38 | 4.18E-04 | Gal3st1 | galactose-3-O-sulfotransferase 1 |
| ENSMUSG00000003352 | 1.76 | 3.38 | 3.90E-08 | Cacnb3 | calcium channel, voltage-dependent, beta 3 subunit |
| ENSMUSG00000030281 | 1.75 | 3.37 | 5.13E-05 | Il17rc | interleukin 17 receptor C |
| ENSMUSG00000001555 | 1.75 | 3.37 | 1.14E-07 | Fkbp10 | FK506 binding protein 10 |
| ENSMUSG00000054418 | 1.75 | 3.37 | 2.64E-03 | NA | NA |
| ENSMUSG00000078570 | 1.75 | 3.36 | 7.68E-05 | 1110065P20Rik | RIKEN cDNA 1110065P20 gene |
| ENSMUSG00000038400 | 1.75 | 3.36 | 9.15E-09 | Pmepa1 | prostate transmembrane protein, androgen induced 1 |
| ENSMUSG00000024247 | 1.75 | 3.35 | 2.06E-06 | Pkdcc | protein kinase domain containing, cytoplasmic |
| ENSMUSG00000028047 | 1.74 | 3.35 | 2.17E-04 | Thbs3 | thrombospondin 3 |
| ENSMUSG00000047182 | 1.74 | 3.33 | 2.03E-03 | Irs3 | insulin receptor substrate 3 |
| ENSMUSG00000029718 | 1.73 | 3.33 | 9.27E-09 | Pcolce | procollagen C-endopeptidase enhancer protein |
| ENSMUSG00000083669 | 1.73 | 3.32 | 1.36E-05 | NA | NA |
| ENSMUSG00000078794 | 1.73 | 3.32 | 3.26E-04 | Dact3 | dapper homolog 3, antagonist of beta-catenin (xenopus) |
| ENSMUSG00000034685 | 1.73 | 3.31 | 9.92E-04 | Fam171a2 | family with sequence similarity 171, member A2 |
| ENSMUSG00000069378 | 1.72 | 3.30 | 8.39E-03 | Prdm6 | PR domain containing 6 |
| ENSMUSG00000041482 | 1.72 | 3.30 | 2.05E-04 | Piezo2 | piezo-type mechanosensitive ion channel component 2 |
| ENSMUSG00000064080 | 1.72 | 3.29 | 1.36E-04 | Fbln2 | fibulin 2 |
| ENSMUSG00000036957 | 1.72 | 3.29 | 6.34E-05 | Lrfn3 | leucine rich repeat and fibronectin type III domain containing 3 |
| ENSMUSG00000040690 | 1.71 | 3.27 | 3.01E-05 | Col16a1 | collagen, type XVI, alpha 1 |
| ENSMUSG00000066861 | 1.70 | 3.26 | 2.68E-06 | Oas1g | 2'-5' oligoadenylate synthetase 1G |
| ENSMUSG00000038422 | 1.70 | 3.25 | 2.57E-04 | Hdhd3 | haloacid dehalogenase-like hydrolase domain containing 3 |
| ENSMUSG00000038508 | 1.70 | 3.24 | 8.74E-03 | Gdf15 | growth differentiation factor 15 |
| ENSMUSG00000047686 | 1.69 | 3.23 | 2.81E-03 | Zcchc5 | zinc finger, CCHC domain containing 5 |
| ENSMUSG00000025511 | 1.69 | 3.22 | 8.93E-08 | Tspan4 | tetraspanin 4 |
| ENSMUSG00000032911 | 1.69 | 3.22 | 2.93E-06 | Cspg4 | chondroitin sulfate proteoglycan 4 |
| ENSMUSG00000036412 | 1.69 | 3.22 | 6.38E-04 | Arsi | arylsulfatase i |
| ENSMUSG00000015094 | 1.68 | 3.21 | 1.12E-09 | Npdc1 | neural proliferation, differentiation and control 1 |
| ENSMUSG00000037254 | 1.68 | 3.21 | 3.55E-03 | Itih2 | inter-alpha trypsin inhibitor, heavy chain 2 |
| ENSMUSG00000034161 | 1.68 | 3.20 | 5.68E-06 | Scx | scleraxis |
| ENSMUSG00000037577 | 1.67 | 3.19 | 2.99E-04 | Ephx3 | epoxide hydrolase 3 |
| ENSMUSG00000007783 | 1.67 | 3.19 | 1.94E-09 | Cpt1c | carnitine palmitoyltransferase 1c |
| ENSMUSG00000006958 | 1.67 | 3.19 | 6.40E-08 | Chrd | chordin |
| ENSMUSG00000036596 | 1.67 | 3.18 | 7.57E-06 | Cpz | carboxypeptidase Z |
| ENSMUSG00000041556 | 1.67 | 3.17 | 1.99E-03 | Fbxo2 | F-box protein 2 |
| ENSMUSG00000020902 | 1.66 | 3.17 | 2.39E-05 | Ntn1 | netrin 1 |
| ENSMUSG00000075012 | 1.66 | 3.16 | 5.81E-04 | Fjx1 | four jointed box 1 (Drosophila) |
| ENSMUSG00000055254 | 1.66 | 3.16 | 1.95E-04 | Ntrk2 | neurotrophic tyrosine kinase, receptor, type 2 |
| ENSMUSG00000021411 | 1.66 | 3.15 | 1.85E-07 | Pxdc1 | PX domain containing 1 |
| ENSMUSG00000051067 | 1.65 | 3.15 | 1.22E-05 | Lingo3 | leucine rich repeat and Ig domain containing 3 |
| ENSMUSG00000030351 | 1.65 | 3.14 | 2.07E-08 | Tspan11 | tetraspanin 11 |
| ENSMUSG00000007039 | 1.65 | 3.14 | 3.90E-08 | Ddah2 | dimethylarginine dimethylaminohydrolase 2 |
| ENSMUSG00000058966 | 1.65 | 3.14 | 1.44E-04 | Fam57b | family with sequence similarity 57, member B |
| ENSMUSG00000029061 | 1.65 | 3.13 | 3.84E-07 | Mmp23 | matrix metallopeptidase 23 |
| ENSMUSG00000033256 | 1.64 | 3.13 | 4.50E-10 | Shf | Src homology 2 domain containing F |
| ENSMUSG00000029032 | 1.64 | 3.13 | 9.36E-03 | Arhgef16 | Rho guanine nucleotide exchange factor (GEF) 16 |
| ENSMUSG00000038777 | 1.63 | 3.10 | 4.88E-04 | Sema6c | sema domain, transmembrane domain (TM), and cytoplasmic domain, (semaphorin) 6C |
| ENSMUSG00000037032 | 1.63 | 3.10 | 3.51E-08 | Apbb1 | amyloid beta (A4) precursor protein-binding, family B, member 1 |
| ENSMUSG00000037347 | 1.63 | 3.10 | 2.19E-04 | Chst7 | carbohydrate (N-acetylglucosamino) sulfotransferase 7 |
| ENSMUSG00000001270 | 1.63 | 3.09 | 1.12E-05 | Ckb | creatine kinase, brain |
| ENSMUSG00000046892 | 1.63 | 3.09 | 4.56E-04 | NA | NA |
| ENSMUSG00000000154 | 1.62 | 3.08 | 2.21E-04 | Slc22a18 | solute carrier family 22 (organic cation transporter), member 18 |
| ENSMUSG00000079017 | 1.62 | 3.08 | 1.06E-05 | Ifi27l2a | interferon, alpha-inducible protein 27 like 2A |
| ENSMUSG00000046491 | 1.62 | 3.07 | 5.65E-04 | C1qtnf2 | C1q and tumor necrosis factor related protein 2 |
| ENSMUSG00000025272 | 1.62 | 3.07 | 7.21E-04 | Tro | trophinin |
| ENSMUSG00000038600 | 1.62 | 3.07 | 8.55E-03 | Atp6v0a4 | ATPase, H+ transporting, lysosomal V0 subunit A4 |
| ENSMUSG00000024868 | 1.62 | 3.06 | 5.59E-05 | Dkk1 | dickkopf homolog 1 (Xenopus laevis) |
| ENSMUSG00000041120 | 1.61 | 3.06 | 4.83E-05 | Nbl1 | neuroblastoma, suppression of tumorigenicity 1 |
| ENSMUSG00000041559 | 1.61 | 3.06 | 2.00E-03 | Fmod | fibromodulin |
| ENSMUSG00000023411 | 1.61 | 3.05 | 5.60E-06 | Nfatc4 | nuclear factor of activated T cells, cytoplasmic, calcineurin dependent 4 |
| ENSMUSG00000060284 | 1.61 | 3.05 | 5.64E-06 | Sp7 | Sp7 transcription factor 7 |
| ENSMUSG00000032649 | 1.60 | 3.03 | 3.41E-03 | Colgalt2 | collagen beta(1-O)galactosyltransferase 2 |
| ENSMUSG00000046470 | 1.60 | 3.03 | 2.64E-05 | Sox18 | SRY (sex determining region Y)-box 18 |
| ENSMUSG00000031595 | 1.60 | 3.03 | 3.30E-05 | Pdgfrl | platelet-derived growth factor receptor-like |
| ENSMUSG00000012017 | 1.60 | 3.02 | 6.64E-05 | Scarf2 | scavenger receptor class F, member 2 |
| ENSMUSG00000052957 | 1.60 | 3.02 | 1.46E-03 | Gas1 | growth arrest specific 1 |
| ENSMUSG00000052353 | 1.60 | 3.02 | 6.20E-04 | 9930013L23Rik | RIKEN cDNA 9930013L23 gene |
| ENSMUSG00000042988 | 1.59 | 3.01 | 6.53E-04 | Notum | notum pectinacetylesterase homolog (Drosophila) |
| ENSMUSG00000041046 | 1.59 | 3.01 | 1.88E-03 | Ramp3 | receptor (calcitonin) activity modifying protein 3 |
| ENSMUSG00000025478 | 1.59 | 3.01 | 8.36E-05 | Dpysl4 | dihydropyrimidinase-like 4 |
| ENSMUSG00000021763 | 1.59 | 3.01 | 3.85E-03 | BC067074 | cDNA sequence BC067074 |
| ENSMUSG00000018930 | 1.58 | 3.00 | 6.96E-03 | Ccl4 | chemokine (C-C motif) ligand 4 |
| ENSMUSG00000028838 | 1.58 | 3.00 | 3.69E-06 | Extl1 | exostoses (multiple)-like 1 |
| ENSMUSG00000056271 | 1.58 | 3.00 | 2.17E-03 | Lman1l | lectin, mannose-binding 1 like |
| ENSMUSG00000053647 | 1.58 | 2.99 | 9.37E-09 | Gper1 | G protein-coupled estrogen receptor 1 |
| ENSMUSG00000035279 | 1.58 | 2.98 | 3.47E-03 | Ssc5d | scavenger receptor cysteine rich domain containing (5 domains) |
| ENSMUSG00000039208 | 1.58 | 2.98 | 1.30E-07 | Metrnl | meteorin, glial cell differentiation regulator-like |
| ENSMUSG00000066975 | 1.57 | 2.97 | 5.90E-03 | Cryba4 | crystallin, beta A4 |
| ENSMUSG00000029576 | 1.57 | 2.97 | 7.86E-03 | Radil | Ras association and DIL domains |
| ENSMUSG00000074178 | 1.57 | 2.97 | 1.24E-03 | NA | NA |
| ENSMUSG00000080115 | 1.57 | 2.97 | 5.15E-03 | LOC100504608 | protein FAM119B-like |
| ENSMUSG00000027457 | 1.57 | 2.96 | 9.55E-03 | Snph | syntaphilin |
| ENSMUSG00000065126 | 1.56 | 2.95 | 8.39E-03 | Snord104 | small nucleolar RNA, C/D box 104 |
| ENSMUSG00000042428 | 1.56 | 2.95 | 1.11E-07 | Mgat3 | mannoside acetylglucosaminyltransferase 3 |
| ENSMUSG00000044303 | 1.56 | 2.95 | 9.50E-04 | Cdkn2a | cyclin-dependent kinase inhibitor 2A |
| ENSMUSG00000061451 | 1.56 | 2.94 | 1.51E-03 | Tmem151a | transmembrane protein 151A |
| ENSMUSG00000043889 | 1.55 | 2.94 | 2.41E-04 | NA | NA |
| ENSMUSG00000060929 | 1.55 | 2.94 | 7.44E-05 | NA | NA |
| ENSMUSG00000064109 | 1.55 | 2.93 | 6.68E-05 | Hcst | hematopoietic cell signal transducer |
| ENSMUSG00000068631 | 1.55 | 2.92 | 2.57E-04 | NA | NA |
| ENSMUSG00000009471 | 1.54 | 2.92 | 5.46E-03 | Myod1 | myogenic differentiation 1 |
| ENSMUSG00000048752 | 1.54 | 2.91 | 1.85E-04 | Prss50 | protease, serine 50 |
| ENSMUSG00000023064 | 1.54 | 2.91 | 5.41E-04 | Sncg | synuclein, gamma |
| ENSMUSG00000044134 | 1.54 | 2.91 | 1.62E-07 | Fam109a | family with sequence similarity 109, member A |
| ENSMUSG00000032491 | 1.54 | 2.91 | 1.29E-05 | Nradd | neurotrophin receptor associated death domain |
| ENSMUSG00000032327 | 1.54 | 2.91 | 6.06E-03 | Stra6 | stimulated by retinoic acid gene 6 |
| ENSMUSG00000019539 | 1.54 | 2.90 | 1.15E-08 | Rcn3 | reticulocalbin 3, EF-hand calcium binding domain |
| ENSMUSG00000012705 | 1.53 | 2.89 | 2.29E-03 | Retn | resistin |
| ENSMUSG00000038457 | 1.53 | 2.89 | 1.69E-03 | Tmem255b | transmembrane protein 255B |
| ENSMUSG00000053318 | 1.53 | 2.88 | 5.78E-04 | Slamf8 | SLAM family member 8 |
| ENSMUSG00000029070 | 1.52 | 2.88 | 4.06E-07 | Mxra8 | matrix-remodelling associated 8 |
| ENSMUSG00000038552 | 1.52 | 2.88 | 3.23E-05 | Fndc4 | fibronectin type III domain containing 4 |
| ENSMUSG00000054675 | 1.52 | 2.88 | 2.69E-08 | Tmem119 | transmembrane protein 119 |
| ENSMUSG00000030499 | 1.52 | 2.87 | 1.14E-05 | Kctd15 | potassium channel tetramerisation domain containing 15 |
| ENSMUSG00000037977 | 1.52 | 2.87 | 2.56E-03 | 6430571L13Rik | RIKEN cDNA 6430571L13 gene |
| ENSMUSG00000007872 | 1.52 | 2.87 | 6.82E-06 | Id3 | inhibitor of DNA binding 3 |
| ENSMUSG00000003380 | 1.52 | 2.87 | 4.69E-06 | Rabac1 | Rab acceptor 1 (prenylated) |
| ENSMUSG00000042766 | 1.52 | 2.87 | 3.22E-05 | Trim46 | tripartite motif-containing 46 |
| ENSMUSG00000040502 | 1.52 | 2.87 | 2.18E-04 | March9 | membrane-associated ring finger (C3HC4) 9 |
| ENSMUSG00000055912 | 1.52 | 2.87 | 2.17E-05 | Tmem150a | transmembrane protein 150A |
| ENSMUSG00000006651 | 1.52 | 2.86 | 1.81E-05 | Aplp1 | amyloid beta (A4) precursor-like protein 1 |
| ENSMUSG00000052026 | 1.52 | 2.86 | 1.29E-04 | Slc6a7 | solute carrier family 6 (neurotransmitter transporter, L-proline), member 7 |
| ENSMUSG00000045672 | 1.52 | 2.86 | 2.49E-03 | Col27a1 | collagen, type XXVII, alpha 1 |
| ENSMUSG00000037813 | 1.52 | 2.86 | 2.77E-05 | D630003M21Rik | RIKEN cDNA D630003M21 gene |
| ENSMUSG00000070867 | 1.51 | 2.86 | 1.81E-04 | Trabd2b | TraB domain containing 2B |
| ENSMUSG00000057969 | 1.51 | 2.85 | 7.82E-08 | Sema3b | sema domain, immunoglobulin domain (Ig), short basic domain, secreted, (semaphorin) 3B |
| ENSMUSG00000025185 | 1.51 | 2.85 | 1.37E-04 | Loxl4 | lysyl oxidase-like 4 |
| ENSMUSG00000019158 | 1.51 | 2.85 | 3.08E-05 | Tmem160 | transmembrane protein 160 |
| ENSMUSG00000018012 | 1.51 | 2.85 | 1.63E-05 | Rac3 | RAS-related C3 botulinum substrate 3 |
| ENSMUSG00000038319 | 1.51 | 2.85 | 2.45E-04 | Kcnh2 | potassium voltage-gated channel, subfamily H (eag-related), member 2 |
| ENSMUSG00000060019 | 1.51 | 2.85 | 1.04E-04 | NA | NA |
| ENSMUSG00000017969 | 1.51 | 2.84 | 4.36E-08 | Ptgis | prostaglandin I2 (prostacyclin) synthase |
| ENSMUSG00000038775 | 1.51 | 2.84 | 9.21E-03 | Vill | villin-like |
| ENSMUSG00000041801 | 1.50 | 2.84 | 1.31E-05 | Phlda3 | pleckstrin homology-like domain, family A, member 3 |
| ENSMUSG00000068396 | 1.50 | 2.84 | 4.21E-04 | NA | NA |
| ENSMUSG00000031906 | 1.50 | 2.83 | 3.87E-04 | Smpd3 | sphingomyelin phosphodiesterase 3, neutral |
| ENSMUSG00000042429 | 1.50 | 2.83 | 6.30E-03 | Adora1 | adenosine A1 receptor |
| ENSMUSG00000032011 | 1.50 | 2.83 | 3.32E-05 | Thy1 | thymus cell antigen 1, theta |
| ENSMUSG00000040212 | 1.50 | 2.82 | 3.04E-06 | Emp3 | epithelial membrane protein 3 |
| ENSMUSG00000027932 | 1.50 | 2.82 | 6.72E-06 | Slc27a3 | solute carrier family 27 (fatty acid transporter), member 3 |
| ENSMUSG00000003355 | 1.49 | 2.82 | 1.77E-04 | Fkbp11 | FK506 binding protein 11 |
| ENSMUSG00000036459 | 1.49 | 2.82 | 3.55E-06 | Wtip | WT1-interacting protein |
| ENSMUSG00000020810 | 1.49 | 2.81 | 8.20E-04 | Cygb | cytoglobin |
| ENSMUSG00000025504 | 1.49 | 2.81 | 7.86E-03 | Eps8l2 | EPS8-like 2 |
| ENSMUSG00000026825 | 1.49 | 2.81 | 2.25E-06 | Dnm1 | dynamin 1 |
| ENSMUSG00000043687 | 1.49 | 2.81 | 8.66E-04 | 1190005I06Rik | RIKEN cDNA 1190005I06 gene |
| ENSMUSG00000024940 | 1.49 | 2.80 | 1.13E-05 | Ltbp3 | latent transforming growth factor beta binding protein 3 |
| ENSMUSG00000020592 | 1.48 | 2.80 | 1.08E-08 | Sdc1 | syndecan 1 |
| ENSMUSG00000024910 | 1.48 | 2.80 | 6.45E-07 | Ctsw | cathepsin W |
| ENSMUSG00000022440 | 1.48 | 2.80 | 1.15E-07 | C1qtnf6 | C1q and tumor necrosis factor related protein 6 |
| ENSMUSG00000024330 | 1.48 | 2.80 | 1.16E-05 | Col11a2 | collagen, type XI, alpha 2 |
| ENSMUSG00000059022 | 1.48 | 2.79 | 9.87E-04 | Kcp | kielin/chordin-like protein |
| ENSMUSG00000046417 | 1.48 | 2.79 | 1.54E-04 | Fam211a | family with sequence similarity 211, member A |
| ENSMUSG00000019194 | 1.48 | 2.79 | 7.92E-06 | Scn1b | sodium channel, voltage-gated, type I, beta |
| ENSMUSG00000019080 | 1.48 | 2.79 | 1.25E-04 | Mfsd3 | major facilitator superfamily domain containing 3 |
| ENSMUSG00000007888 | 1.48 | 2.79 | 4.39E-04 | Crlf1 | cytokine receptor-like factor 1 |
| ENSMUSG00000048616 | 1.48 | 2.78 | 5.84E-03 | Nog | noggin |
| ENSMUSG00000042116 | 1.47 | 2.78 | 2.29E-05 | Vwa1 | von Willebrand factor A domain containing 1 |
| ENSMUSG00000040488 | 1.47 | 2.78 | 2.50E-04 | Ltbp4 | latent transforming growth factor beta binding protein 4 |
| ENSMUSG00000024909 | 1.47 | 2.78 | 2.16E-06 | Efemp2 | epidermal growth factor-containing fibulin-like extracellular matrix protein 2 |
| ENSMUSG00000040841 | 1.47 | 2.78 | 5.80E-05 | Six5 | sine oculis-related homeobox 5 |
| ENSMUSG00000023266 | 1.47 | 2.78 | 9.16E-04 | Frs3 | fibroblast growth factor receptor substrate 3 |
| ENSMUSG00000034226 | 1.47 | 2.77 | 1.48E-03 | Rhov | ras homolog gene family, member V |
| ENSMUSG00000026278 | 1.47 | 2.77 | 6.96E-05 | Bok | BCL2-related ovarian killer protein |
| ENSMUSG00000030470 | 1.47 | 2.77 | 1.89E-03 | Csrp3 | cysteine and glycine-rich protein 3 |
| ENSMUSG00000060260 | 1.47 | 2.77 | 1.31E-04 | Pwwp2b | PWWP domain containing 2B |
| ENSMUSG00000068221 | 1.46 | 2.75 | 3.25E-04 | Pdxp | pyridoxal (pyridoxine, vitamin B6) phosphatase |
| ENSMUSG00000073368 | 1.46 | 2.75 | 2.00E-03 | NA | NA |
| ENSMUSG00000028978 | 1.46 | 2.75 | 1.67E-03 | Nos3 | nitric oxide synthase 3, endothelial cell |
| ENSMUSG00000038742 | 1.46 | 2.75 | 5.89E-03 | Angptl6 | angiopoietin-like 6 |
| ENSMUSG00000048782 | 1.46 | 2.74 | 1.69E-04 | Insc | inscuteable homolog (Drosophila) |
| ENSMUSG00000049939 | 1.46 | 2.74 | 4.95E-03 | Lrrc4 | leucine rich repeat containing 4 |
| ENSMUSG00000015337 | 1.45 | 2.74 | 6.04E-03 | Endog | endonuclease G |
| ENSMUSG00000023191 | 1.45 | 2.74 | 7.47E-07 | Leprel2 | leprecan-like 2 |
| ENSMUSG00000031790 | 1.45 | 2.74 | 2.18E-04 | Mmp15 | matrix metallopeptidase 15 |
| ENSMUSG00000033316 | 1.45 | 2.73 | 2.46E-04 | Galnt9 | UDP-N-acetyl-alpha-D-galactosamine:polypeptide N-acetylgalactosaminyltransferase 9 |
| ENSMUSG00000020793 | 1.45 | 2.73 | 2.13E-04 | Galr2 | galanin receptor 2 |
| ENSMUSG00000025213 | 1.45 | 2.73 | 1.86E-05 | Kazald1 | Kazal-type serine peptidase inhibitor domain 1 |
| ENSMUSG00000032584 | 1.45 | 2.73 | 2.14E-08 | Mst1r | macrophage stimulating 1 receptor (c-met-related tyrosine kinase) |
| ENSMUSG00000028597 | 1.44 | 2.72 | 6.20E-06 | Gpx7 | glutathione peroxidase 7 |
| ENSMUSG00000001348 | 1.44 | 2.72 | 4.02E-06 | Acp5 | acid phosphatase 5, tartrate resistant |
| ENSMUSG00000072812 | 1.44 | 2.71 | 2.09E-03 | Ahnak2 | AHNAK nucleoprotein 2 |
| ENSMUSG00000027313 | 1.44 | 2.71 | 1.22E-03 | Chac1 | ChaC, cation transport regulator 1 |
| ENSMUSG00000029641 | 1.44 | 2.71 | 1.26E-03 | Rasl11a | RAS-like, family 11, member A |
| ENSMUSG00000032523 | 1.44 | 2.70 | 4.82E-04 | Hhatl | hedgehog acyltransferase-like |
| ENSMUSG00000030598 | 1.43 | 2.70 | 1.92E-05 | Fbxo17 | F-box protein 17 |
| ENSMUSG00000054252 | 1.43 | 2.70 | 3.22E-05 | Fgfr3 | fibroblast growth factor receptor 3 |
| ENSMUSG00000028108 | 1.43 | 2.70 | 3.51E-08 | Ecm1 | extracellular matrix protein 1 |
| ENSMUSG00000027560 | 1.43 | 2.70 | 8.00E-04 | Dok5 | docking protein 5 |
| ENSMUSG00000041845 | 1.43 | 2.69 | 1.78E-03 | Rhod | ras homolog gene family, member D |
| ENSMUSG00000020312 | 1.43 | 2.69 | 2.71E-07 | Shc2 | SHC (Src homology 2 domain containing) transforming protein 2 |
| ENSMUSG00000049580 | 1.43 | 2.69 | 3.48E-05 | Tsku | tsukushi |
| ENSMUSG00000045045 | 1.43 | 2.69 | 2.90E-04 | Lrfn4 | leucine rich repeat and fibronectin type III domain containing 4 |
| ENSMUSG00000020937 | 1.43 | 2.69 | 2.93E-06 | Plcd3 | phospholipase C, delta 3 |
| ENSMUSG00000025738 | 1.42 | 2.68 | 1.12E-05 | Fbxl16 | F-box and leucine-rich repeat protein 16 |
| ENSMUSG00000024990 | 1.42 | 2.68 | 2.76E-04 | Rbp4 | retinol binding protein 4, plasma |
| ENSMUSG00000024736 | 1.42 | 2.68 | 2.12E-05 | Tmem132a | transmembrane protein 132A |
| ENSMUSG00000078532 | 1.42 | 2.68 | 2.93E-06 | Nkain1 | Na+/K+ transporting ATPase interacting 1 |
| ENSMUSG00000040323 | 1.42 | 2.68 | 4.66E-05 | NA | NA |
| ENSMUSG00000050212 | 1.42 | 2.68 | 4.92E-04 | Eva1b | eva-1 homolog B (C. elegans) |
| ENSMUSG00000027004 | 1.42 | 2.68 | 4.79E-04 | Frzb | frizzled-related protein |
| ENSMUSG00000036995 | 1.42 | 2.68 | 8.53E-03 | Asap3 | ArfGAP with SH3 domain, ankyrin repeat and PH domain 3 |
| ENSMUSG00000031740 | 1.42 | 2.68 | 2.33E-06 | Mmp2 | matrix metallopeptidase 2 |
| ENSMUSG00000039209 | 1.42 | 2.68 | 1.58E-03 | Rpl39l | ribosomal protein L39-like |
| ENSMUSG00000033722 | 1.42 | 2.67 | 4.85E-06 | BC034090 | cDNA sequence BC034090 |
| ENSMUSG00000078200 | 1.42 | 2.67 | 6.79E-04 | NA | NA |
| ENSMUSG00000036199 | 1.42 | 2.67 | 2.56E-08 | Ndufa13 | NADH dehydrogenase (ubiquinone) 1 alpha subcomplex, 13 |
| ENSMUSG00000082076 | 1.42 | 2.67 | 5.31E-04 | NA | NA |
| ENSMUSG00000032085 | 1.41 | 2.67 | 1.41E-07 | Tagln | transgelin |
| ENSMUSG00000042734 | 1.41 | 2.67 | 6.64E-05 | Ttc9 | tetratricopeptide repeat domain 9 |
| ENSMUSG00000010047 | 1.41 | 2.66 | 5.40E-05 | Hyal2 | hyaluronoglucosaminidase 2 |
| ENSMUSG00000055632 | 1.41 | 2.66 | 5.96E-03 | Hmcn2 | hemicentin 2 |
| ENSMUSG00000074861 | 1.41 | 2.66 | 1.79E-03 | NA | NA |
| ENSMUSG00000036564 | 1.41 | 2.66 | 1.35E-04 | Ndrg4 | N-myc downstream regulated gene 4 |
| ENSMUSG00000040829 | 1.41 | 2.66 | 4.61E-05 | Zmynd15 | zinc finger, MYND-type containing 15 |
| ENSMUSG00000003070 | 1.41 | 2.66 | 3.49E-03 | Efna2 | ephrin A2 |
| ENSMUSG00000014609 | 1.41 | 2.66 | 2.91E-03 | Chrne | cholinergic receptor, nicotinic, epsilon polypeptide |
| ENSMUSG00000048481 | 1.41 | 2.65 | 2.40E-04 | Mypop | Myb-related transcription factor, partner of profilin |
| ENSMUSG00000017713 | 1.41 | 2.65 | 1.29E-03 | Tha1 | threonine aldolase 1 |
| ENSMUSG00000024793 | 1.41 | 2.65 | 4.99E-03 | Tnfrsf25 | tumor necrosis factor receptor superfamily, member 25 |
| ENSMUSG00000053646 | 1.41 | 2.65 | 3.42E-05 | Plxnb1 | plexin B1 |
| ENSMUSG00000017754 | 1.41 | 2.65 | 4.38E-07 | Pltp | phospholipid transfer protein |
| ENSMUSG00000029053 | 1.40 | 2.65 | 6.40E-03 | Prkcz | protein kinase C, zeta |
| ENSMUSG00000040875 | 1.40 | 2.65 | 3.44E-03 | Osbpl10 | oxysterol binding protein-like 10 |
| ENSMUSG00000023886 | 1.40 | 2.64 | 3.32E-06 | Smoc2 | SPARC related modular calcium binding 2 |
| ENSMUSG00000025140 | 1.40 | 2.64 | 1.65E-05 | Pycr1 | pyrroline-5-carboxylate reductase 1 |
| ENSMUSG00000020773 | 1.40 | 2.64 | 1.96E-06 | Trim47 | tripartite motif-containing 47 |
| ENSMUSG00000053559 | 1.40 | 2.64 | 1.71E-05 | Smagp | small cell adhesion glycoprotein |
| ENSMUSG00000048779 | 1.40 | 2.64 | 1.93E-04 | P2ry6 | pyrimidinergic receptor P2Y, G-protein coupled, 6 |
| ENSMUSG00000002980 | 1.40 | 2.64 | 4.94E-06 | Bcam | basal cell adhesion molecule |
| ENSMUSG00000000753 | 1.40 | 2.64 | 1.31E-05 | Serpinf1 | serine (or cysteine) peptidase inhibitor, clade F, member 1 |
| ENSMUSG00000029096 | 1.40 | 2.64 | 3.75E-05 | Htra3 | HtrA serine peptidase 3 |
| ENSMUSG00000000957 | 1.40 | 2.64 | 5.91E-06 | Mmp14 | matrix metallopeptidase 14 (membrane-inserted) |
| ENSMUSG00000026208 | 1.40 | 2.63 | 3.09E-04 | Des | desmin |
| ENSMUSG00000050105 | 1.40 | 2.63 | 1.16E-08 | Grrp1 | glycine/arginine rich protein 1 |
| ENSMUSG00000034040 | 1.39 | 2.63 | 4.18E-04 | Wbscr17 | Williams-Beuren syndrome chromosome region 17 homolog (human) |
| ENSMUSG00000060572 | 1.39 | 2.63 | 4.31E-06 | Mfap2 | microfibrillar-associated protein 2 |
| ENSMUSG00000088272 | 1.39 | 2.62 | 6.30E-03 | NA | NA |
| ENSMUSG00000002847 | 1.39 | 2.62 | 2.08E-04 | Pla1a | phospholipase A1 member A |
| ENSMUSG00000036856 | 1.39 | 2.62 | 2.80E-07 | Wnt4 | wingless-related MMTV integration site 4 |
| ENSMUSG00000002228 | 1.39 | 2.62 | 3.13E-05 | Ppm1j | protein phosphatase 1J |
| ENSMUSG00000021506 | 1.39 | 2.62 | 2.89E-04 | Pitx1 | paired-like homeodomain transcription factor 1 |
| ENSMUSG00000039699 | 1.39 | 2.61 | 3.11E-03 | Batf2 | basic leucine zipper transcription factor, ATF-like 2 |
| ENSMUSG00000003534 | 1.39 | 2.61 | 1.75E-04 | Ddr1 | discoidin domain receptor family, member 1 |
| ENSMUSG00000006378 | 1.38 | 2.61 | 1.80E-03 | NA | NA |
| ENSMUSG00000026442 | 1.38 | 2.60 | 5.90E-03 | Nfasc | neurofascin |
| ENSMUSG00000032925 | 1.38 | 2.60 | 3.56E-03 | Itgbl1 | integrin, beta-like 1 |
| ENSMUSG00000074738 | 1.38 | 2.60 | 1.70E-07 | B930041F14Rik | RIKEN cDNA B930041F14 gene |
| ENSMUSG00000006360 | 1.38 | 2.60 | 7.47E-07 | Crip1 | cysteine-rich protein 1 (intestinal) |
| ENSMUSG00000037206 | 1.38 | 2.60 | 6.63E-06 | Islr | immunoglobulin superfamily containing leucine-rich repeat |
| ENSMUSG00000030796 | 1.37 | 2.59 | 1.50E-06 | Tead2 | TEA domain family member 2 |
| ENSMUSG00000022340 | 1.37 | 2.59 | 1.68E-03 | Sybu | syntabulin (syntaxin-interacting) |
| ENSMUSG00000021253 | 1.37 | 2.58 | 5.27E-05 | Tgfb3 | transforming growth factor, beta 3 |
| ENSMUSG00000020811 | 1.37 | 2.58 | 2.47E-05 | Wscd1 | WSC domain containing 1 |
| ENSMUSG00000042821 | 1.37 | 2.58 | 7.61E-05 | Snai1 | snail homolog 1 (Drosophila) |
| ENSMUSG00000022548 | 1.36 | 2.57 | 3.16E-03 | Apod | apolipoprotein D |
| ENSMUSG00000032180 | 1.36 | 2.57 | 1.72E-04 | Tmed1 | transmembrane emp24 domain containing 1 |
| ENSMUSG00000073403 | 1.36 | 2.57 | 1.54E-04 | NA | NA |
| ENSMUSG00000061848 | 1.36 | 2.57 | 4.42E-04 | NA | NA |
| ENSMUSG00000048087 | 1.36 | 2.57 | 8.46E-03 | NA | NA |
| ENSMUSG00000050796 | 1.36 | 2.57 | 5.19E-04 | B3galt6 | UDP-Gal:betaGal beta 1,3-galactosyltransferase, polypeptide 6 |
| ENSMUSG00000042436 | 1.36 | 2.57 | 4.00E-03 | Mfap4 | microfibrillar-associated protein 4 |
| ENSMUSG00000031841 | 1.36 | 2.56 | 3.51E-06 | Cdh13 | cadherin 13 |
| ENSMUSG00000040390 | 1.36 | 2.56 | 2.31E-05 | Map3k10 | mitogen-activated protein kinase kinase kinase 10 |
| ENSMUSG00000052951 | 1.36 | 2.56 | 1.34E-03 | NA | NA |
| ENSMUSG00000029769 | 1.36 | 2.56 | 2.07E-03 | Ccdc136 | coiled-coil domain containing 136 |
| ENSMUSG00000035274 | 1.36 | 2.56 | 5.96E-04 | Tpbg | trophoblast glycoprotein |
| ENSMUSG00000038276 | 1.36 | 2.56 | 5.27E-06 | Asic3 | acid-sensing (proton-gated) ion channel 3 |
| ENSMUSG00000023153 | 1.35 | 2.55 | 1.68E-03 | Tmem52 | transmembrane protein 52 |
| ENSMUSG00000027488 | 1.35 | 2.55 | 1.14E-07 | Snta1 | syntrophin, acidic 1 |
| ENSMUSG00000070509 | 1.35 | 2.55 | 2.07E-03 | Rgma | repulsive guidance molecule family member A |
| ENSMUSG00000029757 | 1.35 | 2.55 | 5.42E-04 | Dync1i1 | dynein cytoplasmic 1 intermediate chain 1 |
| ENSMUSG00000046056 | 1.35 | 2.55 | 4.57E-03 | Sbsn | suprabasin |
| ENSMUSG00000066607 | 1.35 | 2.55 | 1.01E-05 | 6030419C18Rik | RIKEN cDNA 6030419C18 gene |
| ENSMUSG00000008167 | 1.35 | 2.55 | 6.84E-07 | Fbxw9 | F-box and WD-40 domain protein 9 |
| ENSMUSG00000000094 | 1.35 | 2.55 | 7.73E-04 | Tbx4 | T-box 4 |
| ENSMUSG00000018339 | 1.35 | 2.55 | 9.37E-09 | Gpx3 | glutathione peroxidase 3 |
| ENSMUSG00000049521 | 1.35 | 2.54 | 3.01E-05 | Cdc42ep1 | CDC42 effector protein (Rho GTPase binding) 1 |
| ENSMUSG00000081895 | 1.35 | 2.54 | 2.76E-03 | NA | NA |
| ENSMUSG00000028111 | 1.35 | 2.54 | 1.53E-03 | Ctsk | cathepsin K |
| ENSMUSG00000030170 | 1.35 | 2.54 | 1.08E-05 | Wnt5b | wingless-related MMTV integration site 5B |
| ENSMUSG00000013150 | 1.34 | 2.54 | 5.14E-06 | Gfod2 | glucose-fructose oxidoreductase domain containing 2 |
| ENSMUSG00000011751 | 1.34 | 2.54 | 3.56E-03 | Sptbn4 | spectrin beta, non-erythrocytic 4 |
| ENSMUSG00000031880 | 1.34 | 2.53 | 7.47E-04 | Rrad | Ras-related associated with diabetes |
| ENSMUSG00000062694 | 1.34 | 2.53 | 6.44E-04 | Cav3 | caveolin 3 |
| ENSMUSG00000036067 | 1.34 | 2.53 | 2.19E-04 | Slc2a6 | solute carrier family 2 (facilitated glucose transporter), member 6 |
| ENSMUSG00000035314 | 1.34 | 2.53 | 1.25E-06 | Gdpd5 | glycerophosphodiester phosphodiesterase domain containing 5 |
| ENSMUSG00000040563 | 1.34 | 2.53 | 8.84E-04 | BC018242 | cDNA sequence BC018242 |
| ENSMUSG00000029161 | 1.34 | 2.53 | 7.68E-04 | Cgref1 | cell growth regulator with EF hand domain 1 |
| ENSMUSG00000014303 | 1.34 | 2.53 | 2.05E-04 | Glis2 | GLIS family zinc finger 2 |
| ENSMUSG00000017734 | 1.34 | 2.52 | 3.56E-07 | Dbndd2 | dysbindin (dystrobrevin binding protein 1) domain containing 2 |
| ENSMUSG00000000296 | 1.33 | 2.52 | 4.46E-04 | Tpd52l1 | tumor protein D52-like 1 |
| ENSMUSG00000027221 | 1.33 | 2.52 | 4.01E-04 | Chst1 | carbohydrate (keratan sulfate Gal-6) sulfotransferase 1 |
| ENSMUSG00000012889 | 1.33 | 2.52 | 5.61E-06 | Podnl1 | podocan-like 1 |
| ENSMUSG00000024076 | 1.33 | 2.52 | 3.35E-04 | Vit | vitrin |
| ENSMUSG00000025348 | 1.33 | 2.52 | 5.81E-05 | Itga7 | integrin alpha 7 |
| ENSMUSG00000038765 | 1.33 | 2.52 | 6.99E-03 | Lmx1b | LIM homeobox transcription factor 1 beta |
| ENSMUSG00000006435 | 1.33 | 2.52 | 5.66E-04 | Neurl1a | neuralized homolog 1A (Drosophila) |
| ENSMUSG00000014158 | 1.33 | 2.51 | 4.15E-07 | Trpv4 | transient receptor potential cation channel, subfamily V, member 4 |
| ENSMUSG00000060166 | 1.33 | 2.51 | 1.31E-05 | NA | NA |
| ENSMUSG00000073889 | 1.33 | 2.51 | 4.09E-06 | Il11ra1 | interleukin 11 receptor, alpha chain 1 |
| ENSMUSG00000075702 | 1.33 | 2.51 | 4.60E-07 | Selm | selenoprotein M |
| ENSMUSG00000035783 | 1.33 | 2.51 | 1.66E-07 | Acta2 | actin, alpha 2, smooth muscle, aorta |
| ENSMUSG00000023047 | 1.33 | 2.51 | 2.30E-03 | Amhr2 | anti-Mullerian hormone type 2 receptor |
| ENSMUSG00000027316 | 1.33 | 2.51 | 1.04E-05 | Gfra4 | glial cell line derived neurotrophic factor family receptor alpha 4 |
| ENSMUSG00000044328 | 1.33 | 2.51 | 2.24E-05 | Trp53i13 | transformation related protein 53 inducible protein 13 |
| ENSMUSG00000073838 | 1.33 | 2.51 | 8.50E-04 | Tufm | Tu translation elongation factor, mitochondrial |
| ENSMUSG00000042210 | 1.32 | 2.50 | 6.42E-06 | Abhd14a | abhydrolase domain containing 14A |
| ENSMUSG00000027800 | 1.32 | 2.50 | 4.01E-03 | Tm4sf1 | transmembrane 4 superfamily member 1 |
| ENSMUSG00000079507 | 1.32 | 2.49 | 9.93E-04 | H2-Q1 | histocompatibility 2, Q region locus 1 |
| ENSMUSG00000027447 | 1.32 | 2.49 | 2.87E-07 | Cst3 | cystatin C |
| ENSMUSG00000042737 | 1.32 | 2.49 | 4.87E-05 | Dpm3 | dolichyl-phosphate mannosyltransferase polypeptide 3 |
| ENSMUSG00000078695 | 1.31 | 2.49 | 6.43E-05 | NA | NA |
| ENSMUSG00000023972 | 1.31 | 2.49 | 2.44E-05 | Ptk7 | PTK7 protein tyrosine kinase 7 |
| ENSMUSG00000075268 | 1.31 | 2.49 | 4.10E-05 | NA | NA |
| ENSMUSG00000025978 | 1.31 | 2.49 | 1.69E-05 | Rftn2 | raftlin family member 2 |
| ENSMUSG00000022098 | 1.31 | 2.49 | 2.78E-06 | Bmp1 | bone morphogenetic protein 1 |
| ENSMUSG00000020695 | 1.31 | 2.49 | 4.30E-06 | Mrc2 | mannose receptor, C type 2 |
| ENSMUSG00000006576 | 1.31 | 2.48 | 7.27E-05 | Slc4a3 | solute carrier family 4 (anion exchanger), member 3 |
| ENSMUSG00000054196 | 1.31 | 2.48 | 9.84E-05 | Cthrc1 | collagen triple helix repeat containing 1 |
| ENSMUSG00000065147 | 1.31 | 2.48 | 3.09E-03 | NA | NA |
| ENSMUSG00000061780 | 1.31 | 2.48 | 4.54E-03 | Cfd | complement factor D (adipsin) |
| ENSMUSG00000038128 | 1.31 | 2.48 | 2.20E-03 | Camk4 | calcium/calmodulin-dependent protein kinase IV |
| ENSMUSG00000042828 | 1.31 | 2.48 | 3.91E-04 | Trim72 | tripartite motif-containing 72 |
| ENSMUSG00000045275 | 1.31 | 2.48 | 8.68E-03 | Lca5l | Leber congenital amaurosis 5-like |
| ENSMUSG00000048807 | 1.31 | 2.48 | 4.52E-05 | Slc35e4 | solute carrier family 35, member E4 |
| ENSMUSG00000034930 | 1.31 | 2.48 | 7.78E-03 | Rtkn | rhotekin |
| ENSMUSG00000032334 | 1.31 | 2.48 | 1.74E-04 | Loxl1 | lysyl oxidase-like 1 |
| ENSMUSG00000069806 | 1.31 | 2.47 | 1.95E-04 | NA | NA |
| ENSMUSG00000090210 | 1.31 | 2.47 | 7.74E-08 | Itga10 | integrin, alpha 10 |
| ENSMUSG00000029581 | 1.31 | 2.47 | 9.80E-04 | Fscn1 | fascin homolog 1, actin bundling protein (Strongylocentrotus purpuratus) |
| ENSMUSG00000046031 | 1.31 | 2.47 | 9.73E-03 | Fam26f | family with sequence similarity 26, member F |
| ENSMUSG00000020230 | 1.30 | 2.47 | 1.39E-04 | Prmt2 | protein arginine N-methyltransferase 2 |
| ENSMUSG00000025402 | 1.30 | 2.47 | 1.30E-03 | Nab2 | Ngfi-A binding protein 2 |
| ENSMUSG00000022562 | 1.30 | 2.47 | 5.87E-04 | Oplah | 5-oxoprolinase (ATP-hydrolysing) |
| ENSMUSG00000020722 | 1.30 | 2.47 | 3.66E-04 | Cacng1 | calcium channel, voltage-dependent, gamma subunit 1 |
| ENSMUSG00000000948 | 1.30 | 2.47 | 1.00E-03 | Snrpn | small nuclear ribonucleoprotein N |
| ENSMUSG00000015134 | 1.30 | 2.47 | 7.64E-04 | Aldh1a3 | aldehyde dehydrogenase family 1, subfamily A3 |
| ENSMUSG00000063439 | 1.29 | 2.45 | 8.27E-06 | B9d2 | B9 protein domain 2 |
| ENSMUSG00000020099 | 1.29 | 2.45 | 2.60E-04 | Unc5b | unc-5 homolog B (C. elegans) |
| ENSMUSG00000057777 | 1.29 | 2.45 | 1.39E-03 | Mab21l2 | mab-21-like 2 (C. elegans) |
| ENSMUSG00000022215 | 1.29 | 2.45 | 2.41E-03 | Fitm1 | fat storage-inducing transmembrane protein 1 |
| ENSMUSG00000039457 | 1.29 | 2.44 | 4.52E-03 | Ppl | periplakin |
| ENSMUSG00000062563 | 1.29 | 2.44 | 1.01E-03 | Cys1 | cystin 1 |
| ENSMUSG00000032725 | 1.29 | 2.44 | 1.30E-03 | Folr2 | folate receptor 2 (fetal) |
| ENSMUSG00000070424 | 1.29 | 2.44 | 2.43E-05 | Art5 | ADP-ribosyltransferase 5 |
| ENSMUSG00000027656 | 1.29 | 2.44 | 7.40E-06 | Wisp2 | WNT1 inducible signaling pathway protein 2 |
| ENSMUSG00000043895 | 1.29 | 2.44 | 3.02E-05 | S1pr2 | sphingosine-1-phosphate receptor 2 |
| ENSMUSG00000025207 | 1.29 | 2.44 | 4.54E-05 | Sema4g | sema domain, immunoglobulin domain (Ig), transmembrane domain (TM) and short cytoplasmic domain, (semaphorin) 4G |
| ENSMUSG00000051043 | 1.29 | 2.44 | 9.19E-05 | Gprc5c | G protein-coupled receptor, family C, group 5, member C |
| ENSMUSG00000031972 | 1.29 | 2.44 | 4.93E-04 | Acta1 | actin, alpha 1, skeletal muscle |
| ENSMUSG00000074971 | 1.29 | 2.44 | 9.88E-05 | Fibin | fin bud initiation factor homolog (zebrafish) |
| ENSMUSG00000001510 | 1.28 | 2.44 | 4.09E-04 | Dlx3 | distal-less homeobox 3 |
| ENSMUSG00000055148 | 1.28 | 2.43 | 3.61E-05 | Klf2 | Kruppel-like factor 2 (lung) |
| ENSMUSG00000004267 | 1.28 | 2.42 | 2.81E-04 | Eno2 | enolase 2, gamma neuronal |
| ENSMUSG00000038020 | 1.28 | 2.42 | 1.53E-03 | Rapgefl1 | Rap guanine nucleotide exchange factor (GEF)-like 1 |
| ENSMUSG00000023495 | 1.28 | 2.42 | 1.30E-04 | Pcbp4 | poly(rC) binding protein 4 |
| ENSMUSG00000002343 | 1.27 | 2.42 | 3.81E-06 | Armc6 | armadillo repeat containing 6 |
| ENSMUSG00000030683 | 1.27 | 2.42 | 8.69E-05 | Sez6l2 | seizure related 6 homolog like 2 |
| ENSMUSG00000022199 | 1.27 | 2.42 | 5.21E-04 | Slc22a17 | solute carrier family 22 (organic cation transporter), member 17 |
| ENSMUSG00000047767 | 1.27 | 2.42 | 2.14E-06 | Atg16l2 | autophagy related 16-like 2 (S. cerevisiae) |
| ENSMUSG00000071648 | 1.27 | 2.41 | 1.18E-06 | Rom1 | rod outer segment membrane protein 1 |
| ENSMUSG00000024846 | 1.27 | 2.41 | 6.01E-03 | Cst6 | cystatin E/M |
| ENSMUSG00000030401 | 1.27 | 2.41 | 7.05E-04 | Rtn2 | reticulon 2 (Z-band associated protein) |
| ENSMUSG00000020388 | 1.27 | 2.41 | 3.03E-05 | Pdlim4 | PDZ and LIM domain 4 |
| ENSMUSG00000038086 | 1.27 | 2.41 | 7.75E-04 | Hspb2 | heat shock protein 2 |
| ENSMUSG00000000901 | 1.27 | 2.41 | 4.32E-05 | Mmp11 | matrix metallopeptidase 11 |
| ENSMUSG00000002059 | 1.27 | 2.41 | 1.77E-06 | Rab34 | RAB34, member of RAS oncogene family |
| ENSMUSG00000052911 | 1.27 | 2.41 | 2.19E-07 | Lamb2 | laminin, beta 2 |
| ENSMUSG00000038390 | 1.27 | 2.41 | 2.84E-05 | Gpr162 | G protein-coupled receptor 162 |
| ENSMUSG00000028766 | 1.27 | 2.41 | 2.23E-04 | Alpl | alkaline phosphatase, liver/bone/kidney |
| ENSMUSG00000045930 | 1.26 | 2.40 | 1.25E-04 | Clec14a | C-type lectin domain family 14, member a |
| ENSMUSG00000073674 | 1.26 | 2.40 | 4.53E-04 | NA | NA |
| ENSMUSG00000026126 | 1.26 | 2.40 | 4.27E-04 | Ptpn18 | protein tyrosine phosphatase, non-receptor type 18 |
| ENSMUSG00000024232 | 1.26 | 2.40 | 3.97E-05 | Bambi | BMP and activin membrane-bound inhibitor |
| ENSMUSG00000041696 | 1.26 | 2.40 | 6.01E-05 | Rasl12 | RAS-like, family 12 |
| ENSMUSG00000031443 | 1.26 | 2.40 | 4.77E-03 | F7 | coagulation factor VII |
| ENSMUSG00000032717 | 1.26 | 2.40 | 6.79E-04 | Mdfi | MyoD family inhibitor |
| ENSMUSG00000041420 | 1.26 | 2.40 | 3.18E-05 | Meis3 | Meis homeobox 3 |
| ENSMUSG00000010064 | 1.26 | 2.40 | 4.18E-04 | Slc38a3 | solute carrier family 38, member 3 |
| ENSMUSG00000025425 | 1.26 | 2.39 | 9.78E-03 | St8sia5 | ST8 alpha-N-acetyl-neuraminide alpha-2,8-sialyltransferase 5 |
| ENSMUSG00000080002 | 1.26 | 2.39 | 5.29E-06 | NA | NA |
| ENSMUSG00000002379 | 1.26 | 2.39 | 1.21E-06 | NA | NA |
| ENSMUSG00000029482 | 1.26 | 2.39 | 5.77E-05 | Aacs | acetoacetyl-CoA synthetase |
| ENSMUSG00000058806 | 1.26 | 2.39 | 3.99E-03 | Col13a1 | collagen, type XIII, alpha 1 |
| ENSMUSG00000029163 | 1.26 | 2.39 | 4.72E-05 | Emilin1 | elastin microfibril interfacer 1 |
| ENSMUSG00000020435 | 1.26 | 2.39 | 6.31E-03 | Osbp2 | oxysterol binding protein 2 |
| ENSMUSG00000085988 | 1.25 | 2.39 | 2.11E-03 | NA | NA |
| ENSMUSG00000048450 | 1.25 | 2.39 | 2.93E-03 | Msx1 | msh homeobox 1 |
| ENSMUSG00000034675 | 1.25 | 2.38 | 3.06E-03 | Dbn1 | drebrin 1 |
| ENSMUSG00000028789 | 1.25 | 2.38 | 1.40E-04 | Adc | arginine decarboxylase |
| ENSMUSG00000027603 | 1.25 | 2.38 | 2.68E-04 | Ggt7 | gamma-glutamyltransferase 7 |
| ENSMUSG00000039908 | 1.25 | 2.38 | 5.00E-06 | Slc26a11 | solute carrier family 26, member 11 |
| ENSMUSG00000021835 | 1.25 | 2.38 | 4.69E-06 | Bmp4 | bone morphogenetic protein 4 |
| ENSMUSG00000034993 | 1.25 | 2.38 | 7.02E-06 | Vat1 | vesicle amine transport protein 1 homolog (T californica) |
| ENSMUSG00000034220 | 1.25 | 2.38 | 6.09E-10 | Gpc1 | glypican 1 |
| ENSMUSG00000025854 | 1.24 | 2.37 | 2.42E-04 | Fam20c | family with sequence similarity 20, member C |
| ENSMUSG00000031807 | 1.24 | 2.37 | 3.07E-05 | Pgls | 6-phosphogluconolactonase |
| ENSMUSG00000007207 | 1.24 | 2.36 | 6.09E-05 | Stx1a | syntaxin 1A (brain) |
| ENSMUSG00000067235 | 1.24 | 2.36 | 4.34E-03 | H2-Q10 | histocompatibility 2, Q region locus 10 |
| ENSMUSG00000034684 | 1.24 | 2.36 | 1.25E-05 | Sema3f | sema domain, immunoglobulin domain (Ig), short basic domain, secreted, (semaphorin) 3F |
| ENSMUSG00000046761 | 1.24 | 2.36 | 1.03E-03 | Fam83h | family with sequence similarity 83, member H |
| ENSMUSG00000070577 | 1.24 | 2.36 | 9.85E-03 | Gm572 | predicted gene 572 |
| ENSMUSG00000029126 | 1.24 | 2.36 | 2.32E-05 | Nsg1 | neuron specific gene family member 1 |
| ENSMUSG00000031253 | 1.24 | 2.36 | 1.19E-04 | Srpx2 | sushi-repeat-containing protein, X-linked 2 |
| ENSMUSG00000038502 | 1.24 | 2.36 | 1.39E-05 | Ptov1 | prostate tumor over expressed gene 1 |
| ENSMUSG00000075706 | 1.24 | 2.36 | 1.91E-05 | Gpx4 | glutathione peroxidase 4 |
| ENSMUSG00000007877 | 1.24 | 2.36 | 5.16E-05 | Tcap | titin-cap |
| ENSMUSG00000050910 | 1.24 | 2.36 | 3.07E-07 | Cdr2l | cerebellar degeneration-related protein 2-like |
| ENSMUSG00000002393 | 1.24 | 2.36 | 5.22E-03 | Nr2f6 | nuclear receptor subfamily 2, group F, member 6 |
| ENSMUSG00000020216 | 1.24 | 2.35 | 1.01E-03 | Jsrp1 | junctional sarcoplasmic reticulum protein 1 |
| ENSMUSG00000073684 | 1.23 | 2.35 | 6.42E-06 | 2610002J02Rik | RIKEN cDNA 2610002J02 gene |
| ENSMUSG00000067847 | 1.23 | 2.35 | 1.68E-04 | Romo1 | reactive oxygen species modulator 1 |
| ENSMUSG00000022947 | 1.23 | 2.35 | 6.09E-05 | Cbr3 | carbonyl reductase 3 |
| ENSMUSG00000002308 | 1.23 | 2.35 | 6.97E-05 | Cd320 | CD320 antigen |
| ENSMUSG00000037204 | 1.23 | 2.35 | 6.45E-07 | 9430023L20Rik | RIKEN cDNA 9430023L20 gene |
| ENSMUSG00000033379 | 1.23 | 2.34 | 1.78E-06 | Atp6v0b | ATPase, H+ transporting, lysosomal V0 subunit B |
| ENSMUSG00000051703 | 1.23 | 2.34 | 5.02E-03 | Tmem198 | transmembrane protein 198 |
| ENSMUSG00000071074 | 1.23 | 2.34 | 1.19E-08 | Yipf3 | Yip1 domain family, member 3 |
| ENSMUSG00000001508 | 1.23 | 2.34 | 3.27E-04 | Sgca | sarcoglycan, alpha (dystrophin-associated glycoprotein) |
| ENSMUSG00000004285 | 1.23 | 2.34 | 1.22E-05 | Atp6v1f | ATPase, H+ transporting, lysosomal V1 subunit F |
| ENSMUSG00000067653 | 1.23 | 2.34 | 4.66E-03 | Ankrd23 | ankyrin repeat domain 23 |
| ENSMUSG00000080242 | 1.22 | 2.34 | 4.32E-04 | Atp6v0c-ps2 | ATPase, H+ transporting, lysosomal V0 subunit C, pseudogene 2 |
| ENSMUSG00000051373 | 1.22 | 2.34 | 1.92E-04 | Ppapdc3 | phosphatidic acid phosphatase type 2 domain containing 3 |
| ENSMUSG00000051048 | 1.22 | 2.33 | 2.62E-03 | P4ha3 | procollagen-proline, 2-oxoglutarate 4-dioxygenase (proline 4-hydroxylase), alpha polypeptide III |
| ENSMUSG00000006299 | 1.22 | 2.32 | 8.07E-05 | Aamp | angio-associated migratory protein |
| ENSMUSG00000041889 | 1.22 | 2.32 | 1.43E-04 | Shisa4 | shisa homolog 4 (Xenopus laevis) |
| ENSMUSG00000030317 | 1.21 | 2.32 | 1.41E-03 | Timp4 | tissue inhibitor of metalloproteinase 4 |
| ENSMUSG00000054793 | 1.21 | 2.32 | 6.80E-03 | Cadm4 | cell adhesion molecule 4 |
| ENSMUSG00000004473 | 1.21 | 2.32 | 6.32E-05 | Clec11a | C-type lectin domain family 11, member a |
| ENSMUSG00000006205 | 1.21 | 2.32 | 5.83E-05 | Htra1 | HtrA serine peptidase 1 |
| ENSMUSG00000034394 | 1.21 | 2.32 | 2.88E-03 | Lif | leukemia inhibitory factor |
| ENSMUSG00000033735 | 1.21 | 2.32 | 3.50E-04 | Spr | sepiapterin reductase |
| ENSMUSG00000049686 | 1.21 | 2.31 | 2.38E-04 | Orai1 | ORAI calcium release-activated calcium modulator 1 |
| ENSMUSG00000020086 | 1.21 | 2.31 | 9.46E-04 | H2afy2 | H2A histone family, member Y2 |
| ENSMUSG00000030674 | 1.21 | 2.31 | 7.88E-03 | Qprt | quinolinate phosphoribosyltransferase |
| ENSMUSG00000023232 | 1.21 | 2.31 | 5.08E-03 | Serinc2 | serine incorporator 2 |
| ENSMUSG00000026211 | 1.21 | 2.31 | 1.50E-04 | Obsl1 | obscurin-like 1 |
| ENSMUSG00000016256 | 1.21 | 2.31 | 4.56E-07 | Ctsz | cathepsin Z |
| ENSMUSG00000043964 | 1.21 | 2.31 | 7.50E-05 | Orai3 | ORAI calcium release-activated calcium modulator 3 |
| ENSMUSG00000015085 | 1.21 | 2.31 | 3.69E-03 | Entpd2 | ectonucleoside triphosphate diphosphohydrolase 2 |
| ENSMUSG00000030672 | 1.21 | 2.31 | 3.74E-04 | Mylpf | myosin light chain, phosphorylatable, fast skeletal muscle |
| ENSMUSG00000021268 | 1.21 | 2.31 | 6.00E-03 | Meg3 | maternally expressed 3 |
| ENSMUSG00000027954 | 1.21 | 2.31 | 2.79E-04 | Efna1 | ephrin A1 |
| ENSMUSG00000066235 | 1.21 | 2.31 | 2.24E-05 | Pomgnt2 | protein O-linked mannose beta 1,4-N-acetylglucosaminyltransferase 2 |
| ENSMUSG00000020241 | 1.20 | 2.31 | 1.78E-05 | Col6a2 | collagen, type VI, alpha 2 |
| ENSMUSG00000026223 | 1.20 | 2.30 | 3.41E-05 | Itm2c | integral membrane protein 2C |
| ENSMUSG00000050777 | 1.20 | 2.30 | 1.07E-03 | Tmem37 | transmembrane protein 37 |
| ENSMUSG00000034175 | 1.20 | 2.30 | 3.06E-04 | Rhbdd3 | rhomboid domain containing 3 |
| ENSMUSG00000024299 | 1.20 | 2.30 | 6.41E-05 | Adamts10 | a disintegrin-like and metallopeptidase (reprolysin type) with thrombospondin type 1 motif, 10 |
| ENSMUSG00000059195 | 1.20 | 2.30 | 6.05E-03 | NA | NA |
| ENSMUSG00000001751 | 1.20 | 2.30 | 5.74E-07 | Naglu | alpha-N-acetylglucosaminidase (Sanfilippo disease IIIB) |
| ENSMUSG00000031633 | 1.20 | 2.30 | 2.47E-05 | Slc25a4 | solute carrier family 25 (mitochondrial carrier, adenine nucleotide translocator), member 4 |
| ENSMUSG00000031239 | 1.20 | 2.29 | 3.01E-04 | Itm2a | integral membrane protein 2A |
| ENSMUSG00000033597 | 1.20 | 2.29 | 2.13E-03 | Caskin1 | CASK interacting protein 1 |
| ENSMUSG00000024346 | 1.20 | 2.29 | 8.44E-05 | Pfdn1 | prefoldin 1 |
| ENSMUSG00000050248 | 1.20 | 2.29 | 9.56E-06 | Evc2 | Ellis van Creveld syndrome 2 |
| ENSMUSG00000026879 | 1.20 | 2.29 | 7.14E-08 | Gsn | gelsolin |
| ENSMUSG00000056590 | 1.19 | 2.29 | 5.71E-03 | NA | NA |
| ENSMUSG00000028782 | 1.19 | 2.28 | 2.69E-03 | Bai2 | brain-specific angiogenesis inhibitor 2 |
| ENSMUSG00000038539 | 1.19 | 2.28 | 1.24E-04 | Atf5 | activating transcription factor 5 |
| ENSMUSG00000036570 | 1.19 | 2.28 | 5.85E-06 | Fxyd1 | FXYD domain-containing ion transport regulator 1 |
| ENSMUSG00000031097 | 1.19 | 2.28 | 2.81E-04 | Tnni2 | troponin I, skeletal, fast 2 |
| ENSMUSG00000074796 | 1.19 | 2.28 | 9.78E-03 | Slc4a11 | solute carrier family 4, sodium bicarbonate transporter-like, member 11 |
| ENSMUSG00000024399 | 1.19 | 2.28 | 2.20E-04 | Ltb | lymphotoxin B |
| ENSMUSG00000023992 | 1.19 | 2.28 | 9.74E-03 | Trem2 | triggering receptor expressed on myeloid cells 2 |
| ENSMUSG00000070371 | 1.19 | 2.27 | 3.88E-04 | Prss36 | protease, serine 36 |
| ENSMUSG00000040287 | 1.19 | 2.27 | 9.68E-04 | Stac3 | SH3 and cysteine rich domain 3 |
| ENSMUSG00000002105 | 1.19 | 2.27 | 2.08E-08 | Slc39a13 | solute carrier family 39 (metal ion transporter), member 13 |
| ENSMUSG00000004947 | 1.18 | 2.27 | 7.47E-05 | Dtx2 | deltex 2 homolog (Drosophila) |
| ENSMUSG00000029166 | 1.18 | 2.27 | 5.80E-06 | Mapre3 | microtubule-associated protein, RP/EB family, member 3 |
| ENSMUSG00000039450 | 1.18 | 2.27 | 4.33E-05 | Dcxr | dicarbonyl L-xylulose reductase |
| ENSMUSG00000033423 | 1.18 | 2.27 | 1.34E-07 | Eri3 | exoribonuclease 3 |
| ENSMUSG00000024958 | 1.18 | 2.27 | 5.05E-06 | Gpr137 | G protein-coupled receptor 137 |
| ENSMUSG00000031503 | 1.18 | 2.27 | 1.86E-06 | Col4a2 | collagen, type IV, alpha 2 |
| ENSMUSG00000037966 | 1.18 | 2.27 | 1.58E-04 | Ninj1 | ninjurin 1 |
| ENSMUSG00000028641 | 1.18 | 2.27 | 1.60E-05 | Lepre1 | leprecan 1 |
| ENSMUSG00000003199 | 1.18 | 2.27 | 1.87E-07 | Mpnd | MPN domain containing |
| ENSMUSG00000023885 | 1.18 | 2.27 | 8.01E-04 | Thbs2 | thrombospondin 2 |
| ENSMUSG00000028601 | 1.18 | 2.26 | 1.54E-04 | Echdc2 | enoyl Coenzyme A hydratase domain containing 2 |
| ENSMUSG00000030772 | 1.18 | 2.26 | 6.81E-04 | Dkk3 | dickkopf homolog 3 (Xenopus laevis) |
| ENSMUSG00000027611 | 1.18 | 2.26 | 2.96E-04 | Procr | protein C receptor, endothelial |
| ENSMUSG00000024338 | 1.18 | 2.26 | 4.84E-05 | Psmb8 | proteasome (prosome, macropain) subunit, beta type 8 (large multifunctional peptidase 7) |
| ENSMUSG00000027408 | 1.18 | 2.26 | 1.03E-03 | Cpxm1 | carboxypeptidase X 1 (M14 family) |
| ENSMUSG00000018906 | 1.18 | 2.26 | 1.73E-04 | P4ha2 | procollagen-proline, 2-oxoglutarate 4-dioxygenase (proline 4-hydroxylase), alpha II polypeptide |
| ENSMUSG00000017002 | 1.18 | 2.26 | 6.75E-04 | Slpi | secretory leukocyte peptidase inhibitor |
| ENSMUSG00000020758 | 1.18 | 2.26 | 4.83E-04 | Itgb4 | integrin beta 4 |
| ENSMUSG00000013419 | 1.18 | 2.26 | 1.42E-04 | Zfp651 | zinc finger protein 651 |
| ENSMUSG00000004931 | 1.17 | 2.26 | 6.52E-05 | Apba3 | amyloid beta (A4) precursor protein-binding, family A, member 3 |
| ENSMUSG00000041939 | 1.17 | 2.26 | 1.57E-04 | Mvk | mevalonate kinase |
| ENSMUSG00000073556 | 1.17 | 2.26 | 1.36E-04 | NA | NA |
| ENSMUSG00000024012 | 1.17 | 2.26 | 8.99E-08 | Mtch1 | mitochondrial carrier homolog 1 (C. elegans) |
| ENSMUSG00000021806 | 1.17 | 2.26 | 2.35E-04 | Nid2 | nidogen 2 |
| ENSMUSG00000040289 | 1.17 | 2.26 | 7.49E-03 | Hey1 | hairy/enhancer-of-split related with YRPW motif 1 |
| ENSMUSG00000026820 | 1.17 | 2.26 | 8.85E-08 | Ptges2 | prostaglandin E synthase 2 |
| ENSMUSG00000032271 | 1.17 | 2.26 | 3.51E-03 | Nnmt | nicotinamide N-methyltransferase |
| ENSMUSG00000014846 | 1.17 | 2.25 | 5.22E-03 | Tppp3 | tubulin polymerization-promoting protein family member 3 |
| ENSMUSG00000067924 | 1.17 | 2.25 | 7.84E-06 | NA | NA |
| ENSMUSG00000084093 | 1.17 | 2.25 | 4.22E-03 | NA | NA |
| ENSMUSG00000050234 | 1.17 | 2.25 | 2.61E-04 | Gja4 | gap junction protein, alpha 4 |
| ENSMUSG00000028458 | 1.17 | 2.25 | 9.77E-05 | Tesk1 | testis specific protein kinase 1 |
| ENSMUSG00000031955 | 1.17 | 2.25 | 7.97E-06 | Bcar1 | breast cancer anti-estrogen resistance 1 |
| ENSMUSG00000048537 | 1.17 | 2.25 | 4.61E-05 | Phldb1 | pleckstrin homology-like domain, family B, member 1 |
| ENSMUSG00000033389 | 1.17 | 2.24 | 1.61E-03 | Arhgap44 | Rho GTPase activating protein 44 |
| ENSMUSG00000044337 | 1.17 | 2.24 | 1.74E-03 | Ackr3 | atypical chemokine receptor 3 |
| ENSMUSG00000003072 | 1.17 | 2.24 | 2.01E-05 | Atp5d | ATP synthase, H+ transporting, mitochondrial F1 complex, delta subunit |
| ENSMUSG00000038260 | 1.16 | 2.24 | 4.66E-05 | Trpm4 | transient receptor potential cation channel, subfamily M, member 4 |
| ENSMUSG00000032135 | 1.16 | 2.24 | 6.06E-05 | Mcam | melanoma cell adhesion molecule |
| ENSMUSG00000043099 | 1.16 | 2.24 | 3.28E-03 | Hic1 | hypermethylated in cancer 1 |
| ENSMUSG00000068758 | 1.16 | 2.24 | 5.94E-04 | Il3ra | interleukin 3 receptor, alpha chain |
| ENSMUSG00000068220 | 1.16 | 2.23 | 4.64E-04 | Lgals1 | lectin, galactose binding, soluble 1 |
| ENSMUSG00000026857 | 1.16 | 2.23 | 7.08E-03 | Ntmt1 | N-terminal Xaa-Pro-Lys N-methyltransferase 1 |
| ENSMUSG00000060600 | 1.16 | 2.23 | 4.91E-04 | Eno3 | enolase 3, beta muscle |
| ENSMUSG00000038451 | 1.16 | 2.23 | 2.46E-03 | Spsb2 | splA/ryanodine receptor domain and SOCS box containing 2 |
| ENSMUSG00000006390 | 1.16 | 2.23 | 1.42E-04 | Elovl1 | elongation of very long chain fatty acids (FEN1/Elo2, SUR4/Elo3, yeast)-like 1 |
| ENSMUSG00000042784 | 1.16 | 2.23 | 5.57E-04 | Muc1 | mucin 1, transmembrane |
| ENSMUSG00000044367 | 1.15 | 2.23 | 5.86E-05 | Slc16a13 | solute carrier family 16 (monocarboxylic acid transporters), member 13 |
| ENSMUSG00000009633 | 1.15 | 2.23 | 2.37E-03 | G0s2 | G0/G1 switch gene 2 |
| ENSMUSG00000027163 | 1.15 | 2.22 | 2.89E-06 | Commd9 | COMM domain containing 9 |
| ENSMUSG00000055681 | 1.15 | 2.22 | 7.20E-06 | Cope | coatomer protein complex, subunit epsilon |
| ENSMUSG00000055093 | 1.15 | 2.22 | 2.49E-04 | NA | NA |
| ENSMUSG00000033044 | 1.15 | 2.22 | 1.00E-03 | Dhrs7c | dehydrogenase/reductase (SDR family) member 7C |
| ENSMUSG00000035595 | 1.15 | 2.22 | 1.30E-03 | 1600002K03Rik | RIKEN cDNA 1600002K03 gene |
| ENSMUSG00000031385 | 1.15 | 2.22 | 5.74E-03 | Plxnb3 | plexin B3 |
| ENSMUSG00000027188 | 1.15 | 2.22 | 3.45E-03 | Pamr1 | peptidase domain containing associated with muscle regeneration 1 |
| ENSMUSG00000026796 | 1.15 | 2.22 | 1.04E-03 | Fam129b | family with sequence similarity 129, member B |
| ENSMUSG00000004846 | 1.15 | 2.22 | 4.54E-05 | Plod3 | procollagen-lysine, 2-oxoglutarate 5-dioxygenase 3 |
| ENSMUSG00000008136 | 1.15 | 2.22 | 1.09E-03 | Fhl2 | four and a half LIM domains 2 |
| ENSMUSG00000034259 | 1.15 | 2.22 | 7.83E-04 | Exosc4 | exosome component 4 |
| ENSMUSG00000068697 | 1.15 | 2.22 | 5.42E-03 | Myoz1 | myozenin 1 |
| ENSMUSG00000020186 | 1.15 | 2.21 | 1.31E-05 | Csrp2 | cysteine and glycine-rich protein 2 |
| ENSMUSG00000042834 | 1.15 | 2.21 | 6.99E-04 | Nrep | neuronal regeneration related protein |
| ENSMUSG00000051184 | 1.14 | 2.21 | 4.39E-05 | Zfp524 | zinc finger protein 524 |
| ENSMUSG00000035041 | 1.14 | 2.21 | 7.78E-03 | Creb3l3 | cAMP responsive element binding protein 3-like 3 |
| ENSMUSG00000008206 | 1.14 | 2.21 | 5.02E-05 | Cers4 | ceramide synthase 4 |
| ENSMUSG00000031897 | 1.14 | 2.21 | 4.80E-04 | Psmb10 | proteasome (prosome, macropain) subunit, beta type 10 |
| ENSMUSG00000025610 | 1.14 | 2.21 | 8.41E-03 | Map3k7cl | Map3k7 C-terminal like |
| ENSMUSG00000003346 | 1.14 | 2.20 | 4.26E-04 | Abhd17a | abhydrolase domain containing 17A |
| ENSMUSG00000052974 | 1.14 | 2.20 | 3.15E-03 | Cyp2f2 | cytochrome P450, family 2, subfamily f, polypeptide 2 |
| ENSMUSG00000032122 | 1.14 | 2.20 | 6.52E-06 | Slc37a2 | solute carrier family 37 (glycerol-3-phosphate transporter), member 2 |
| ENSMUSG00000001131 | 1.14 | 2.20 | 3.57E-03 | Timp1 | tissue inhibitor of metalloproteinase 1 |
| ENSMUSG00000062661 | 1.14 | 2.20 | 2.77E-03 | Ncs1 | neuronal calcium sensor 1 |
| ENSMUSG00000021260 | 1.14 | 2.20 | 2.01E-03 | Hhipl1 | hedgehog interacting protein-like 1 |
| ENSMUSG00000026548 | 1.14 | 2.20 | 5.03E-03 | Slamf9 | SLAM family member 9 |
| ENSMUSG00000003970 | 1.14 | 2.20 | 7.12E-04 | Rpl8 | ribosomal protein L8 |
| ENSMUSG00000030032 | 1.13 | 2.20 | 1.05E-03 | Wdr54 | WD repeat domain 54 |
| ENSMUSG00000049422 | 1.13 | 2.19 | 2.87E-03 | Chchd10 | coiled-coil-helix-coiled-coil-helix domain containing 10 |
| ENSMUSG00000034744 | 1.13 | 2.19 | 5.74E-05 | Nagk | N-acetylglucosamine kinase |
| ENSMUSG00000033249 | 1.13 | 2.19 | 4.77E-04 | Hsf4 | heat shock transcription factor 4 |
| ENSMUSG00000025171 | 1.13 | 2.19 | 1.33E-05 | Ubtd1 | ubiquitin domain containing 1 |
| ENSMUSG00000032615 | 1.13 | 2.19 | 1.77E-06 | Nt5m | 5',3'-nucleotidase, mitochondrial |
| ENSMUSG00000073418 | 1.13 | 2.19 | 7.94E-05 | C4b | complement component 4B (Chido blood group) |
| ENSMUSG00000020282 | 1.13 | 2.19 | 7.38E-05 | Rhbdf1 | rhomboid family 1 (Drosophila) |
| ENSMUSG00000023259 | 1.13 | 2.19 | 3.84E-04 | NA | NA |
| ENSMUSG00000034412 | 1.13 | 2.19 | 1.16E-05 | Tbc1d10a | TBC1 domain family, member 10a |
| ENSMUSG00000069920 | 1.13 | 2.19 | 5.12E-04 | B3gnt9 | UDP-GlcNAc:betaGal beta-1,3-N-acetylglucosaminyltransferase 9 |
| ENSMUSG00000045007 | 1.13 | 2.19 | 7.94E-05 | Tubg2 | tubulin, gamma 2 |
| ENSMUSG00000033998 | 1.13 | 2.18 | 3.24E-05 | Kcnk1 | potassium channel, subfamily K, member 1 |
| ENSMUSG00000048329 | 1.13 | 2.18 | 5.36E-03 | Mfsd6l | major facilitator superfamily domain containing 6-like |
| ENSMUSG00000029545 | 1.13 | 2.18 | 4.93E-06 | Acads | acyl-Coenzyme A dehydrogenase, short chain |
| ENSMUSG00000036111 | 1.12 | 2.18 | 4.37E-06 | Lmo1 | LIM domain only 1 |
| ENSMUSG00000032431 | 1.12 | 2.18 | 3.16E-05 | Crtap | cartilage associated protein |
| ENSMUSG00000030339 | 1.12 | 2.18 | 5.32E-05 | Ltbr | lymphotoxin B receptor |
| ENSMUSG00000048200 | 1.12 | 2.18 | 2.87E-04 | Efcab4a | EF-hand calcium binding domain 4A |
| ENSMUSG00000010307 | 1.12 | 2.18 | 2.19E-05 | Tmem86a | transmembrane protein 86A |
| ENSMUSG00000001240 | 1.12 | 2.18 | 3.99E-07 | Ramp2 | receptor (calcitonin) activity modifying protein 2 |
| ENSMUSG00000002771 | 1.12 | 2.18 | 1.99E-03 | Grin2d | glutamate receptor, ionotropic, NMDA2D (epsilon 4) |
| ENSMUSG00000027314 | 1.12 | 2.17 | 9.65E-05 | Dll4 | delta-like 4 (Drosophila) |
| ENSMUSG00000021464 | 1.12 | 2.17 | 4.83E-03 | Ror2 | receptor tyrosine kinase-like orphan receptor 2 |
| ENSMUSG00000029762 | 1.12 | 2.17 | 7.98E-03 | Akr1b8 | aldo-keto reductase family 1, member B8 |
| ENSMUSG00000038167 | 1.12 | 2.17 | 2.70E-03 | Plekhg6 | pleckstrin homology domain containing, family G (with RhoGef domain) member 6 |
| ENSMUSG00000071451 | 1.12 | 2.17 | 1.28E-03 | Psmg4 | proteasome (prosome, macropain) assembly chaperone 4 |
| ENSMUSG00000004105 | 1.12 | 2.17 | 1.29E-03 | Angptl2 | angiopoietin-like 2 |
| ENSMUSG00000025875 | 1.12 | 2.17 | 1.68E-04 | Tspan17 | tetraspanin 17 |
| ENSMUSG00000026043 | 1.12 | 2.17 | 1.23E-03 | Col3a1 | collagen, type III, alpha 1 |
| ENSMUSG00000048755 | 1.12 | 2.17 | 6.06E-05 | Mcat | malonyl CoA:ACP acyltransferase (mitochondrial) |
| ENSMUSG00000024440 | 1.12 | 2.17 | 6.15E-05 | Pcdh12 | protocadherin 12 |
| ENSMUSG00000005628 | 1.12 | 2.17 | 2.83E-04 | Tmod4 | tropomodulin 4 |
| ENSMUSG00000028885 | 1.12 | 2.17 | 1.55E-03 | Smpdl3b | sphingomyelin phosphodiesterase, acid-like 3B |
| ENSMUSG00000048126 | 1.12 | 2.17 | 2.10E-05 | Col6a3 | collagen, type VI, alpha 3 |
| ENSMUSG00000001020 | 1.12 | 2.17 | 1.57E-05 | S100a4 | S100 calcium binding protein A4 |
| ENSMUSG00000074486 | 1.12 | 2.17 | 2.54E-03 | Bglap2 | bone gamma-carboxyglutamate protein 2 |
| ENSMUSG00000040883 | 1.12 | 2.17 | 9.98E-04 | Tmem205 | transmembrane protein 205 |
| ENSMUSG00000042570 | 1.12 | 2.17 | 8.07E-05 | Mier2 | mesoderm induction early response 1, family member 2 |
| ENSMUSG00000026958 | 1.11 | 2.16 | 2.59E-07 | Dpp7 | dipeptidylpeptidase 7 |
| ENSMUSG00000026890 | 1.11 | 2.16 | 9.51E-03 | Lhx6 | LIM homeobox protein 6 |
| ENSMUSG00000018920 | 1.11 | 2.16 | 8.11E-04 | Cxcl16 | chemokine (C-X-C motif) ligand 16 |
| ENSMUSG00000001507 | 1.11 | 2.16 | 1.66E-04 | Itga3 | integrin alpha 3 |
| ENSMUSG00000041429 | 1.11 | 2.16 | 2.81E-04 | Nthl1 | nth (endonuclease III)-like 1 (E.coli) |
| ENSMUSG00000028214 | 1.11 | 2.16 | 5.47E-03 | Gem | GTP binding protein (gene overexpressed in skeletal muscle) |
| ENSMUSG00000031808 | 1.11 | 2.16 | 2.32E-05 | Slc27a1 | solute carrier family 27 (fatty acid transporter), member 1 |
| ENSMUSG00000028763 | 1.11 | 2.16 | 9.46E-04 | Hspg2 | perlecan (heparan sulfate proteoglycan 2) |
| ENSMUSG00000011096 | 1.11 | 2.16 | 1.13E-04 | Akt1s1 | AKT1 substrate 1 (proline-rich) |
| ENSMUSG00000004098 | 1.11 | 2.16 | 3.40E-03 | Col5a3 | collagen, type V, alpha 3 |
| ENSMUSG00000030122 | 1.11 | 2.15 | 4.45E-04 | Ptms | parathymosin |
| ENSMUSG00000034659 | 1.11 | 2.15 | 1.43E-04 | Tmem109 | transmembrane protein 109 |
| ENSMUSG00000074280 | 1.11 | 2.15 | 1.15E-04 | NA | NA |
| ENSMUSG00000036120 | 1.11 | 2.15 | 3.79E-03 | Rfxank | regulatory factor X-associated ankyrin-containing protein |
| ENSMUSG00000022750 | 1.11 | 2.15 | 5.46E-05 | Klhl22 | kelch-like 22 |
| ENSMUSG00000002058 | 1.10 | 2.15 | 2.24E-03 | Unc119 | unc-119 homolog (C. elegans) |
| ENSMUSG00000050821 | 1.10 | 2.15 | 1.36E-04 | Fam131a | family with sequence similarity 131, member A |
| ENSMUSG00000062077 | 1.10 | 2.15 | 2.59E-03 | Trim54 | tripartite motif-containing 54 |
| ENSMUSG00000045594 | 1.10 | 2.15 | 5.18E-08 | Glb1 | galactosidase, beta 1 |
| ENSMUSG00000022131 | 1.10 | 2.15 | 1.63E-05 | Gpr180 | G protein-coupled receptor 180 |
| ENSMUSG00000016349 | 1.10 | 2.15 | 5.94E-03 | Eef1a2 | eukaryotic translation elongation factor 1 alpha 2 |
| ENSMUSG00000023336 | 1.10 | 2.15 | 9.37E-04 | Wfdc1 | WAP four-disulfide core domain 1 |
| ENSMUSG00000034854 | 1.10 | 2.15 | 7.80E-05 | Mfsd12 | major facilitator superfamily domain containing 12 |
| ENSMUSG00000038264 | 1.10 | 2.15 | 1.53E-04 | Sema7a | sema domain, immunoglobulin domain (Ig), and GPI membrane anchor, (semaphorin) 7A |
| ENSMUSG00000017652 | 1.10 | 2.15 | 8.33E-03 | Cd40 | CD40 antigen |
| ENSMUSG00000023904 | 1.10 | 2.14 | 1.89E-08 | Hcfc1r1 | host cell factor C1 regulator 1 (XPO1-dependent) |
| ENSMUSG00000085939 | 1.10 | 2.14 | 4.72E-05 | NA | NA |
| ENSMUSG00000059430 | 1.10 | 2.14 | 1.16E-03 | Actg2 | actin, gamma 2, smooth muscle, enteric |
| ENSMUSG00000040350 | 1.10 | 2.14 | 4.65E-04 | Trim7 | tripartite motif-containing 7 |
| ENSMUSG00000050552 | 1.10 | 2.14 | 6.14E-04 | Lamtor4 | late endosomal/lysosomal adaptor, MAPK and MTOR activator 4 |
| ENSMUSG00000029060 | 1.10 | 2.14 | 2.96E-04 | Mib2 | mindbomb homolog 2 (Drosophila) |
| ENSMUSG00000037621 | 1.10 | 2.14 | 6.84E-08 | Atoh8 | atonal homolog 8 (Drosophila) |
| ENSMUSG00000046822 | 1.10 | 2.14 | 2.85E-05 | Slc39a3 | solute carrier family 39 (zinc transporter), member 3 |
| ENSMUSG00000034771 | 1.10 | 2.14 | 3.60E-03 | Tle2 | transducin-like enhancer of split 2, homolog of Drosophila E(spl) |
| ENSMUSG00000019467 | 1.09 | 2.14 | 1.15E-04 | Arhgef25 | Rho guanine nucleotide exchange factor (GEF) 25 |
| ENSMUSG00000029401 | 1.09 | 2.13 | 2.13E-05 | Rilpl2 | Rab interacting lysosomal protein-like 2 |
| ENSMUSG00000047423 | 1.09 | 2.13 | 2.76E-03 | AI837181 | expressed sequence AI837181 |
| ENSMUSG00000039646 | 1.09 | 2.13 | 1.63E-04 | Vasn | vasorin |
| ENSMUSG00000025651 | 1.09 | 2.13 | 1.24E-05 | Uqcrc1 | ubiquinol-cytochrome c reductase core protein 1 |
| ENSMUSG00000048402 | 1.09 | 2.13 | 1.96E-03 | Gli2 | GLI-Kruppel family member GLI2 |
| ENSMUSG00000040606 | 1.09 | 2.13 | 6.56E-04 | Kazn | kazrin, periplakin interacting protein |
| ENSMUSG00000027848 | 1.09 | 2.13 | 9.96E-05 | Olfml3 | olfactomedin-like 3 |
| ENSMUSG00000074916 | 1.09 | 2.13 | 2.65E-03 | Chst14 | carbohydrate (N-acetylgalactosamine 4-0) sulfotransferase 14 |
| ENSMUSG00000067925 | 1.09 | 2.13 | 5.28E-04 | NA | NA |
| ENSMUSG00000027546 | 1.09 | 2.12 | 1.37E-05 | Atp9a | ATPase, class II, type 9A |
| ENSMUSG00000018585 | 1.09 | 2.12 | 2.68E-05 | Atox1 | ATX1 (antioxidant protein 1) homolog 1 (yeast) |
| ENSMUSG00000074063 | 1.09 | 2.12 | 5.19E-04 | Osgin1 | oxidative stress induced growth inhibitor 1 |
| ENSMUSG00000054855 | 1.09 | 2.12 | 2.20E-03 | Rnd1 | Rho family GTPase 1 |
| ENSMUSG00000032363 | 1.09 | 2.12 | 7.29E-04 | Adamts7 | a disintegrin-like and metallopeptidase (reprolysin type) with thrombospondin type 1 motif, 7 |
| ENSMUSG00000030707 | 1.09 | 2.12 | 2.13E-04 | Coro1a | coronin, actin binding protein 1A |
| ENSMUSG00000031851 | 1.09 | 2.12 | 1.35E-05 | Ntpcr | nucleoside-triphosphatase, cancer-related |
| ENSMUSG00000037499 | 1.09 | 2.12 | 2.67E-07 | Nenf | neuron derived neurotrophic factor |
| ENSMUSG00000019659 | 1.08 | 2.12 | 1.74E-03 | Ccdc12 | coiled-coil domain containing 12 |
| ENSMUSG00000056737 | 1.08 | 2.12 | 1.37E-05 | Capg | capping protein (actin filament), gelsolin-like |
| ENSMUSG00000003299 | 1.08 | 2.12 | 4.02E-06 | Mrpl4 | mitochondrial ribosomal protein L4 |
| ENSMUSG00000043822 | 1.08 | 2.12 | 2.08E-04 | Adamtsl5 | ADAMTS-like 5 |
| ENSMUSG00000044986 | 1.08 | 2.12 | 4.32E-03 | Tst | thiosulfate sulfurtransferase, mitochondrial |
| ENSMUSG00000044092 | 1.08 | 2.12 | 5.00E-06 | C130050O18Rik | RIKEN cDNA C130050O18 gene |
| ENSMUSG00000008090 | 1.08 | 2.12 | 5.08E-05 | Fgfrl1 | fibroblast growth factor receptor-like 1 |
| ENSMUSG00000008999 | 1.08 | 2.11 | 9.19E-03 | Bmp7 | bone morphogenetic protein 7 |
| ENSMUSG00000002985 | 1.08 | 2.11 | 7.30E-04 | Apoe | apolipoprotein E |
| ENSMUSG00000021456 | 1.08 | 2.11 | 7.13E-03 | Fbp2 | fructose bisphosphatase 2 |
| ENSMUSG00000024970 | 1.08 | 2.11 | 1.12E-05 | AI846148 | expressed sequence AI846148 |
| ENSMUSG00000026930 | 1.08 | 2.11 | 2.09E-04 | Gpsm1 | G-protein signalling modulator 1 (AGS3-like, C. elegans) |
| ENSMUSG00000002804 | 1.08 | 2.11 | 1.67E-04 | Nudt14 | nudix (nucleoside diphosphate linked moiety X)-type motif 14 |
| ENSMUSG00000024186 | 1.07 | 2.11 | 5.55E-03 | Rgs11 | regulator of G-protein signaling 11 |
| ENSMUSG00000024168 | 1.07 | 2.11 | 6.48E-04 | Tmem204 | transmembrane protein 204 |
| ENSMUSG00000036206 | 1.07 | 2.10 | 7.57E-05 | Sh3bp4 | SH3-domain binding protein 4 |
| ENSMUSG00000036545 | 1.07 | 2.10 | 8.41E-06 | Adamts2 | a disintegrin-like and metallopeptidase (reprolysin type) with thrombospondin type 1 motif, 2 |
| ENSMUSG00000032606 | 1.07 | 2.10 | 1.16E-03 | Nicn1 | nicolin 1 |
| ENSMUSG00000002108 | 1.07 | 2.10 | 1.75E-04 | Nr1h3 | nuclear receptor subfamily 1, group H, member 3 |
| ENSMUSG00000024968 | 1.07 | 2.10 | 8.61E-04 | Rcor2 | REST corepressor 2 |
| ENSMUSG00000048696 | 1.07 | 2.10 | 4.42E-03 | Mex3d | mex3 homolog D (C. elegans) |
| ENSMUSG00000004937 | 1.07 | 2.09 | 3.41E-07 | Sgta | small glutamine-rich tetratricopeptide repeat (TPR)-containing, alpha |
| ENSMUSG00000002250 | 1.07 | 2.09 | 4.81E-04 | NA | NA |
| ENSMUSG00000075389 | 1.07 | 2.09 | 5.61E-03 | NA | NA |
| ENSMUSG00000019027 | 1.07 | 2.09 | 2.02E-03 | Dnah1 | dynein, axonemal, heavy chain 1 |
| ENSMUSG00000045176 | 1.07 | 2.09 | 1.61E-04 | 2310047M10Rik | RIKEN cDNA 2310047M10 gene |
| ENSMUSG00000011837 | 1.06 | 2.09 | 1.89E-04 | Snapc2 | small nuclear RNA activating complex, polypeptide 2 |
| ENSMUSG00000052504 | 1.06 | 2.09 | 4.11E-03 | Epha3 | Eph receptor A3 |
| ENSMUSG00000009876 | 1.06 | 2.09 | 6.66E-03 | Cox4i2 | cytochrome c oxidase subunit IV isoform 2 |
| ENSMUSG00000036862 | 1.06 | 2.09 | 6.75E-04 | Dchs1 | dachsous 1 (Drosophila) |
| ENSMUSG00000027420 | 1.06 | 2.09 | 1.50E-03 | Bfsp1 | beaded filament structural protein 1, in lens-CP94 |
| ENSMUSG00000038387 | 1.06 | 2.09 | 4.98E-04 | Rras | Harvey rat sarcoma oncogene, subgroup R |
| ENSMUSG00000035829 | 1.06 | 2.09 | 1.12E-03 | Ppp1r26 | protein phosphatase 1, regulatory subunit 26 |
| ENSMUSG00000024959 | 1.06 | 2.08 | 1.92E-05 | Bad | BCL2-associated agonist of cell death |
| ENSMUSG00000031828 | 1.06 | 2.08 | 1.16E-03 | Klhl36 | kelch-like 36 |
| ENSMUSG00000003420 | 1.06 | 2.08 | 1.04E-05 | Fcgrt | Fc receptor, IgG, alpha chain transporter |
| ENSMUSG00000025484 | 1.05 | 2.08 | 1.07E-03 | Bet1l | blocked early in transport 1 homolog (S. cerevisiae)-like |
| ENSMUSG00000009035 | 1.05 | 2.08 | 2.36E-04 | Tmem184b | transmembrane protein 184b |
| ENSMUSG00000081544 | 1.05 | 2.08 | 9.97E-03 | NA | NA |
| ENSMUSG00000025466 | 1.05 | 2.07 | 9.93E-04 | Fuom | fucose mutarotase |
| ENSMUSG00000023990 | 1.05 | 2.07 | 2.52E-04 | Tfeb | transcription factor EB |
| ENSMUSG00000009563 | 1.05 | 2.07 | 3.34E-05 | Tor2a | torsin family 2, member A |
| ENSMUSG00000020477 | 1.05 | 2.07 | 1.44E-04 | Mrps24 | mitochondrial ribosomal protein S24 |
| ENSMUSG00000002763 | 1.05 | 2.07 | 4.08E-04 | Pex6 | peroxisomal biogenesis factor 6 |
| ENSMUSG00000056204 | 1.05 | 2.07 | 2.47E-04 | Pgpep1 | pyroglutamyl-peptidase I |
| ENSMUSG00000035165 | 1.05 | 2.07 | 9.28E-03 | Kcne3 | potassium voltage-gated channel, Isk-related subfamily, gene 3 |
| ENSMUSG00000027223 | 1.05 | 2.07 | 3.52E-03 | Mapk8ip1 | mitogen-activated protein kinase 8 interacting protein 1 |
| ENSMUSG00000034845 | 1.05 | 2.07 | 3.66E-03 | Plvap | plasmalemma vesicle associated protein |
| ENSMUSG00000001802 | 1.05 | 2.07 | 1.58E-03 | Lrp3 | low density lipoprotein receptor-related protein 3 |
| ENSMUSG00000038055 | 1.05 | 2.07 | 1.31E-06 | Dexi | dexamethasone-induced transcript |
| ENSMUSG00000043251 | 1.05 | 2.07 | 6.53E-03 | Exoc3l | exocyst complex component 3-like |
| ENSMUSG00000047945 | 1.05 | 2.07 | 9.88E-05 | Marcksl1 | MARCKS-like 1 |
| ENSMUSG00000015312 | 1.05 | 2.07 | 2.46E-03 | Gadd45b | growth arrest and DNA-damage-inducible 45 beta |
| ENSMUSG00000039461 | 1.05 | 2.07 | 2.16E-04 | Tcta | T cell leukemia translocation altered gene |
| ENSMUSG00000041571 | 1.05 | 2.07 | 2.39E-04 | Sepw1 | selenoprotein W, muscle 1 |
| ENSMUSG00000037190 | 1.05 | 2.07 | 7.30E-04 | Cyb561d2 | cytochrome b-561 domain containing 2 |
| ENSMUSG00000050022 | 1.05 | 2.07 | 2.89E-03 | Amz1 | archaelysin family metallopeptidase 1 |
| ENSMUSG00000015363 | 1.05 | 2.07 | 1.31E-05 | Trabd | TraB domain containing |
| ENSMUSG00000025204 | 1.05 | 2.07 | 1.24E-04 | Ndufb8 | NADH dehydrogenase (ubiquinone) 1 beta subcomplex 8 |
| ENSMUSG00000007950 | 1.05 | 2.07 | 7.59E-04 | Abhd8 | abhydrolase domain containing 8 |
| ENSMUSG00000030621 | 1.05 | 2.07 | 5.90E-03 | Me3 | malic enzyme 3, NADP(+)-dependent, mitochondrial |
| ENSMUSG00000036114 | 1.05 | 2.07 | 4.98E-04 | Rpp25l | ribonuclease P/MRP 25 subunit-like |
| ENSMUSG00000031169 | 1.05 | 2.07 | 8.91E-04 | Porcn | porcupine homolog (Drosophila) |
| ENSMUSG00000026785 | 1.05 | 2.07 | 2.04E-06 | Pkn3 | protein kinase N3 |
| ENSMUSG00000036138 | 1.05 | 2.07 | 3.39E-04 | Acaa1a | acetyl-Coenzyme A acyltransferase 1A |
| ENSMUSG00000044199 | 1.05 | 2.06 | 8.10E-03 | S1pr4 | sphingosine-1-phosphate receptor 4 |
| ENSMUSG00000034463 | 1.05 | 2.06 | 3.07E-04 | Scara3 | scavenger receptor class A, member 3 |
| ENSMUSG00000022525 | 1.05 | 2.06 | 9.66E-03 | Hrasls | HRAS-like suppressor |
| ENSMUSG00000073422 | 1.05 | 2.06 | 3.77E-04 | H2-Ke6 | H2-K region expressed gene 6 |
| ENSMUSG00000031958 | 1.05 | 2.06 | 2.63E-03 | Ldhd | lactate dehydrogenase D |
| ENSMUSG00000058357 | 1.05 | 2.06 | 1.30E-03 | NA | NA |
| ENSMUSG00000014776 | 1.04 | 2.06 | 1.06E-03 | Nol3 | nucleolar protein 3 (apoptosis repressor with CARD domain) |
| ENSMUSG00000035914 | 1.04 | 2.06 | 1.87E-03 | Cd276 | CD276 antigen |
| ENSMUSG00000022584 | 1.04 | 2.06 | 5.85E-03 | Ly6c2 | lymphocyte antigen 6 complex, locus C2 |
| ENSMUSG00000034108 | 1.04 | 2.06 | 3.37E-04 | Ccs | copper chaperone for superoxide dismutase |
| ENSMUSG00000010045 | 1.04 | 2.06 | 9.47E-05 | Tmem115 | transmembrane protein 115 |
| ENSMUSG00000024187 | 1.04 | 2.06 | 3.05E-06 | Itfg3 | integrin alpha FG-GAP repeat containing 3 |
| ENSMUSG00000000031 | 1.04 | 2.06 | 9.88E-03 | NA | NA |
| ENSMUSG00000040605 | 1.04 | 2.06 | 1.55E-03 | Bace2 | beta-site APP-cleaving enzyme 2 |
| ENSMUSG00000047067 | 1.04 | 2.06 | 2.31E-03 | Dusp28 | dual specificity phosphatase 28 |
| ENSMUSG00000027661 | 1.04 | 2.06 | 2.41E-03 | Slc2a10 | solute carrier family 2 (facilitated glucose transporter), member 10 |
| ENSMUSG00000029810 | 1.04 | 2.06 | 1.46E-04 | Tmem176b | transmembrane protein 176B |
| ENSMUSG00000005057 | 1.04 | 2.05 | 3.65E-04 | Sh2b2 | SH2B adaptor protein 2 |
| ENSMUSG00000038034 | 1.04 | 2.05 | 7.03E-04 | Igsf8 | immunoglobulin superfamily, member 8 |
| ENSMUSG00000052105 | 1.04 | 2.05 | 7.18E-03 | Soga2 | SOGA family member 2 |
| ENSMUSG00000026837 | 1.04 | 2.05 | 6.39E-04 | Col5a1 | collagen, type V, alpha 1 |
| ENSMUSG00000064254 | 1.04 | 2.05 | 8.30E-04 | Ethe1 | ethylmalonic encephalopathy 1 |
| ENSMUSG00000044894 | 1.04 | 2.05 | 5.58E-04 | Uqcrq | ubiquinol-cytochrome c reductase, complex III subunit VII |
| ENSMUSG00000078588 | 1.04 | 2.05 | 6.15E-03 | Ccdc24 | coiled-coil domain containing 24 |
| ENSMUSG00000006519 | 1.04 | 2.05 | 9.01E-04 | Cyba | cytochrome b-245, alpha polypeptide |
| ENSMUSG00000063060 | 1.04 | 2.05 | 3.83E-03 | Sox7 | SRY (sex determining region Y)-box 7 |
| ENSMUSG00000030804 | 1.03 | 2.05 | 3.31E-04 | NA | NA |
| ENSMUSG00000026817 | 1.03 | 2.05 | 1.63E-03 | Ak1 | adenylate kinase 1 |
| ENSMUSG00000018599 | 1.03 | 2.05 | 1.30E-03 | Mief2 | mitochondrial elongation factor 2 |
| ENSMUSG00000027076 | 1.03 | 2.05 | 9.97E-06 | Timm10 | translocase of inner mitochondrial membrane 10 |
| ENSMUSG00000048277 | 1.03 | 2.05 | 1.18E-04 | Syngr2 | synaptogyrin 2 |
| ENSMUSG00000042804 | 1.03 | 2.05 | 2.25E-03 | Gpr153 | G protein-coupled receptor 153 |
| ENSMUSG00000021483 | 1.03 | 2.04 | 2.97E-03 | Cdk20 | cyclin-dependent kinase 20 |
| ENSMUSG00000002803 | 1.03 | 2.04 | 9.61E-07 | Btbd6 | BTB (POZ) domain containing 6 |
| ENSMUSG00000024875 | 1.03 | 2.04 | 4.09E-05 | Yif1a | Yip1 interacting factor homolog A (S. cerevisiae) |
| ENSMUSG00000050232 | 1.03 | 2.04 | 5.24E-03 | Cxcr3 | chemokine (C-X-C motif) receptor 3 |
| ENSMUSG00000040964 | 1.03 | 2.04 | 1.15E-04 | Arhgef10l | Rho guanine nucleotide exchange factor (GEF) 10-like |
| ENSMUSG00000054474 | 1.03 | 2.04 | 4.77E-03 | Thnsl2 | threonine synthase-like 2 (bacterial) |
| ENSMUSG00000032513 | 1.03 | 2.04 | 5.86E-05 | Gorasp1 | golgi reassembly stacking protein 1 |
| ENSMUSG00000038486 | 1.03 | 2.04 | 5.59E-03 | Sv2a | synaptic vesicle glycoprotein 2 a |
| ENSMUSG00000019558 | 1.03 | 2.04 | 1.63E-03 | Slc6a8 | solute carrier family 6 (neurotransmitter transporter, creatine), member 8 |
| ENSMUSG00000038845 | 1.03 | 2.04 | 1.90E-03 | NA | NA |
| ENSMUSG00000025537 | 1.03 | 2.04 | 8.60E-03 | Phkg1 | phosphorylase kinase gamma 1 |
| ENSMUSG00000074170 | 1.03 | 2.04 | 2.93E-07 | Plekhf1 | pleckstrin homology domain containing, family F (with FYVE domain) member 1 |
| ENSMUSG00000029093 | 1.03 | 2.04 | 2.81E-04 | Sorcs2 | sortilin-related VPS10 domain containing receptor 2 |
| ENSMUSG00000082575 | 1.02 | 2.03 | 7.45E-05 | NA | NA |
| ENSMUSG00000001506 | 1.02 | 2.03 | 4.98E-03 | Col1a1 | collagen, type I, alpha 1 |
| ENSMUSG00000029122 | 1.02 | 2.03 | 4.20E-05 | Evc | Ellis van Creveld gene syndrome |
| ENSMUSG00000029710 | 1.02 | 2.03 | 5.10E-05 | Ephb4 | Eph receptor B4 |
| ENSMUSG00000039157 | 1.02 | 2.03 | 6.39E-04 | Fam102a | family with sequence similarity 102, member A |
| ENSMUSG00000027894 | 1.02 | 2.03 | 9.80E-03 | Slc6a17 | solute carrier family 6 (neurotransmitter transporter), member 17 |
| ENSMUSG00000028848 | 1.02 | 2.03 | 3.33E-05 | Gpn2 | GPN-loop GTPase 2 |
| ENSMUSG00000034863 | 1.02 | 2.03 | 2.60E-03 | Ano8 | anoctamin 8 |
| ENSMUSG00000048644 | 1.02 | 2.03 | 5.40E-03 | Ctxn1 | cortexin 1 |
| ENSMUSG00000035413 | 1.02 | 2.03 | 3.24E-03 | Tmem98 | transmembrane protein 98 |
| ENSMUSG00000006395 | 1.02 | 2.03 | 3.87E-04 | Hyi | hydroxypyruvate isomerase homolog (E. coli) |
| ENSMUSG00000066705 | 1.02 | 2.03 | 8.83E-04 | Fxyd6 | FXYD domain-containing ion transport regulator 6 |
| ENSMUSG00000036820 | 1.02 | 2.03 | 9.64E-05 | Amdhd2 | amidohydrolase domain containing 2 |
| ENSMUSG00000017390 | 1.02 | 2.03 | 4.16E-04 | Aldoc | aldolase C, fructose-bisphosphate |
| ENSMUSG00000002504 | 1.02 | 2.03 | 2.47E-05 | Slc9a3r2 | solute carrier family 9 (sodium/hydrogen exchanger), member 3 regulator 2 |
| ENSMUSG00000071649 | 1.02 | 2.03 | 2.56E-03 | B3gat3 | beta-1,3-glucuronyltransferase 3 (glucuronosyltransferase I) |
| ENSMUSG00000015981 | 1.02 | 2.03 | 2.59E-03 | Stk32c | serine/threonine kinase 32C |
| ENSMUSG00000020475 | 1.02 | 2.02 | 3.47E-03 | Pgam2 | phosphoglycerate mutase 2 |
| ENSMUSG00000068551 | 1.02 | 2.02 | 2.77E-04 | Zfp467 | zinc finger protein 467 |
| ENSMUSG00000034254 | 1.02 | 2.02 | 2.69E-03 | Agpat1 | 1-acylglycerol-3-phosphate O-acyltransferase 1 (lysophosphatidic acid acyltransferase, alpha) |
| ENSMUSG00000074576 | 1.02 | 2.02 | 5.27E-03 | Mocs3 | molybdenum cofactor synthesis 3 |
| ENSMUSG00000023905 | 1.01 | 2.02 | 9.61E-03 | Tnfrsf12a | tumor necrosis factor receptor superfamily, member 12a |
| ENSMUSG00000026956 | 1.01 | 2.02 | 3.20E-07 | Uap1l1 | UDP-N-acteylglucosamine pyrophosphorylase 1-like 1 |
| ENSMUSG00000028743 | 1.01 | 2.02 | 6.01E-05 | Akr7a5 | aldo-keto reductase family 7, member A5 (aflatoxin aldehyde reductase) |
| ENSMUSG00000000693 | 1.01 | 2.02 | 1.62E-04 | Loxl3 | lysyl oxidase-like 3 |
| ENSMUSG00000004996 | 1.01 | 2.02 | 1.87E-05 | Mri1 | methylthioribose-1-phosphate isomerase homolog (S. cerevisiae) |
| ENSMUSG00000017737 | 1.01 | 2.02 | 2.58E-07 | Mmp9 | matrix metallopeptidase 9 |
| ENSMUSG00000051790 | 1.01 | 2.01 | 5.17E-03 | Nlgn2 | neuroligin 2 |
| ENSMUSG00000039405 | 1.01 | 2.01 | 1.74E-03 | Prss23 | protease, serine 23 |
| ENSMUSG00000070436 | 1.01 | 2.01 | 2.09E-05 | Serpinh1 | serine (or cysteine) peptidase inhibitor, clade H, member 1 |
| ENSMUSG00000030431 | 1.01 | 2.01 | 4.09E-04 | Tmem238 | transmembrane protein 238 |
| ENSMUSG00000037349 | 1.01 | 2.01 | 2.19E-05 | Nudt22 | nudix (nucleoside diphosphate linked moiety X)-type motif 22 |
| ENSMUSG00000030284 | 1.01 | 2.01 | 4.67E-04 | Creld1 | cysteine-rich with EGF-like domains 1 |
| ENSMUSG00000001750 | 1.01 | 2.01 | 1.28E-05 | Tcirg1 | T cell, immune regulator 1, ATPase, H+ transporting, lysosomal V0 protein A3 |
| ENSMUSG00000022817 | 1.01 | 2.01 | 1.59E-05 | Itgb5 | integrin beta 5 |
| ENSMUSG00000031387 | 1.00 | 2.01 | 6.94E-06 | Renbp | renin binding protein |
| ENSMUSG00000024906 | 1.00 | 2.01 | 7.91E-04 | Mus81 | MUS81 endonuclease homolog (yeast) |
| ENSMUSG00000033313 | 1.00 | 2.01 | 1.29E-03 | Fbxl8 | F-box and leucine-rich repeat protein 8 |
| ENSMUSG00000082536 | 1.00 | 2.00 | 1.68E-03 | NA | NA |
| ENSMUSG00000075279 | 1.00 | 2.00 | 9.23E-06 | NA | NA |
| ENSMUSG00000029312 | 1.00 | 2.00 | 4.25E-04 | Klhl8 | kelch-like 8 |
| ENSMUSG00000050390 | 1.00 | 2.00 | 7.04E-03 | C77080 | expressed sequence C77080 |
| ENSMUSG00000039759 | 1.00 | 2.00 | 1.43E-04 | Thap3 | THAP domain containing, apoptosis associated protein 3 |
| ENSMUSG00000033542 | 1.00 | 2.00 | 2.39E-03 | Arhgef5 | Rho guanine nucleotide exchange factor (GEF) 5 |
| ENSMUSG00000016427 | 1.00 | 2.00 | 6.75E-04 | Ndufa1 | NADH dehydrogenase (ubiquinone) 1 alpha subcomplex, 1 |
| ENSMUSG00000059864 | -2.99 | -7.943085832 | 1.05E-03 | Olfr1393 | olfactory receptor 1393 |
| ENSMUSG00000051497 | -2.85 | -7.201559714 | 9.67E-04 | Kcnj16 | potassium inwardly-rectifying channel, subfamily J, member 16 |
| ENSMUSG00000048334 | -2.84 | -7.184593126 | 4.78E-06 | NA | NA |
| ENSMUSG00000086749 | -2.81 | -7.034825978 | 1.36E-03 | NA | NA |
| ENSMUSG00000081603 | -2.79 | -6.90458597 | 1.83E-05 | NA | NA |
| ENSMUSG00000085860 | -2.69 | -6.45450652 | 3.47E-03 | 2410003L11Rik | RIKEN cDNA 2410003L11 gene |
| ENSMUSG00000075569 | -2.63 | -6.194600572 | 1.13E-03 | Rsph10b | radial spoke head 10 homolog B (Chlamydomonas) |
| ENSMUSG00000081179 | -2.57 | -5.952644409 | 4.72E-08 | NA | NA |
| ENSMUSG00000083186 | -2.47 | -5.527993221 | 7.30E-06 | NA | NA |
| ENSMUSG00000087118 | -2.43 | -5.395013326 | 2.04E-04 | NA | NA |
| ENSMUSG00000053641 | -2.42 | -5.334896091 | 5.11E-05 | Dennd4a | DENN/MADD domain containing 4A |
| ENSMUSG00000053541 | -2.40 | -5.27849164 | 5.86E-03 | Gm4759 | GTPase, very large interferon inducible 1 pseudogene |
| ENSMUSG00000074662 | -2.40 | -5.271226944 | 9.01E-03 | NA | NA |
| ENSMUSG00000090215 | -2.36 | -5.149917674 | 1.09E-07 | Trim34b | tripartite motif-containing 34B |
| ENSMUSG00000044211 | -2.36 | -5.133489958 | 5.61E-06 | NA | NA |
| ENSMUSG00000083834 | -2.36 | -5.12267797 | 9.31E-04 | NA | NA |
| ENSMUSG00000034997 | -2.32 | -4.989061717 | 2.24E-04 | Htr2a | 5-hydroxytryptamine (serotonin) receptor 2A |
| ENSMUSG00000056531 | -2.31 | -4.94743392 | 2.72E-05 | Ccdc18 | coiled-coil domain containing 18 |
| ENSMUSG00000039697 | -2.30 | -4.920448864 | 1.63E-07 | Ncoa7 | nuclear receptor coactivator 7 |
| ENSMUSG00000087635 | -2.27 | -4.837056465 | 4.48E-04 | NA | NA |
| ENSMUSG00000078649 | -2.27 | -4.823840747 | 5.25E-03 | NA | NA |
| ENSMUSG00000081138 | -2.25 | -4.76569905 | 2.02E-04 | NA | NA |
| ENSMUSG00000028312 | -2.23 | -4.703116839 | 7.56E-08 | Smc2 | structural maintenance of chromosomes 2 |
| ENSMUSG00000082872 | -2.23 | -4.680480519 | 2.97E-05 | NA | NA |
| ENSMUSG00000022360 | -2.22 | -4.648935932 | 6.33E-07 | Atad2 | ATPase family, AAA domain containing 2 |
| ENSMUSG00000019971 | -2.21 | -4.627902361 | 3.69E-06 | Cep290 | centrosomal protein 290 |
| ENSMUSG00000083681 | -2.20 | -4.607774961 | 7.00E-05 | NA | NA |
| ENSMUSG00000022105 | -2.20 | -4.60615497 | 9.26E-08 | Rb1 | retinoblastoma 1 |
| ENSMUSG00000085085 | -2.20 | -4.602829634 | 9.87E-03 | NA | NA |
| ENSMUSG00000039809 | -2.19 | -4.550530635 | 2.69E-04 | Gabbr2 | gamma-aminobutyric acid (GABA) B receptor, 2 |
| ENSMUSG00000027505 | -2.18 | -4.539456839 | 2.98E-03 | Fam209 | family with sequence similarity 209 |
| ENSMUSG00000078495 | -2.18 | -4.538217412 | 2.87E-07 | Gm13157 | predicted gene 13157 |
| ENSMUSG00000042133 | -2.18 | -4.527683103 | 3.95E-06 | Ppig | peptidyl-prolyl isomerase G (cyclophilin G) |
| ENSMUSG00000047534 | -2.16 | -4.474767143 | 9.38E-07 | Mis18bp1 | MIS18 binding protein 1 |
| ENSMUSG00000037593 | -2.15 | -4.429892302 | 3.39E-03 | BC030499 | cDNA sequence BC030499 |
| ENSMUSG00000074867 | -2.15 | -4.428955439 | 2.20E-05 | Zfp808 | zinc finger protein 80 |
| ENSMUSG00000057440 | -2.15 | -4.424087072 | 1.36E-04 | Mpp7 | membrane protein, palmitoylated 7 (MAGUK p55 subfamily member 7) |
| ENSMUSG00000025899 | -2.13 | -4.362663578 | 7.82E-08 | Alkbh8 | alkB, alkylation repair homolog 8 (E. coli) |
| ENSMUSG00000081871 | -2.12 | -4.334789371 | 5.36E-03 | NA | NA |
| ENSMUSG00000031004 | -2.11 | -4.314153864 | 2.35E-07 | Mki67 | antigen identified by monoclonal antibody Ki 67 |
| ENSMUSG00000052812 | -2.10 | -4.288315745 | 2.02E-03 | Atad2b | ATPase family, AAA domain containing 2B |
| ENSMUSG00000032407 | -2.09 | -4.263822218 | 1.16E-08 | U2surp | U2 snRNP-associated SURP domain containing |
| ENSMUSG00000021714 | -2.09 | -4.260562285 | 1.55E-05 | Cenpk | centromere protein K |
| ENSMUSG00000044966 | -2.09 | -4.253809704 | 2.20E-04 | Fbxo48 | F-box protein 48 |
| ENSMUSG00000082321 | -2.08 | -4.215816474 | 6.74E-06 | NA | NA |
| ENSMUSG00000046404 | -2.07 | -4.198040416 | 9.47E-03 | Yod1 | YOD1 OTU deubiquitinating enzyme 1 homologue (S. cerevisiae) |
| ENSMUSG00000003282 | -2.07 | -4.191596427 | 7.93E-03 | Plag1 | pleiomorphic adenoma gene 1 |
| ENSMUSG00000082145 | -2.06 | -4.182667371 | 7.45E-07 | NA | NA |
| ENSMUSG00000050243 | -2.06 | -4.17538175 | 1.62E-06 | NA | NA |
| ENSMUSG00000058331 | -2.06 | -4.16085862 | 1.64E-04 | Zfp85 | zinc finger protein 85 |
| ENSMUSG00000049164 | -2.06 | -4.158075575 | 3.91E-04 | Zfp518a | zinc finger protein 518A |
| ENSMUSG00000084806 | -2.05 | -4.153435732 | 7.79E-03 | Gm15232 | predicted gene 15232 |
| ENSMUSG00000050921 | -2.05 | -4.14704032 | 8.11E-05 | P2ry10 | purinergic receptor P2Y, G-protein coupled 10 |
| ENSMUSG00000086513 | -2.04 | -4.126025746 | 8.20E-03 | NA | NA |
| ENSMUSG00000083407 | -2.04 | -4.123670757 | 9.70E-03 | NA | NA |
| ENSMUSG00000078784 | -2.04 | -4.102052604 | 2.14E-03 | NA | NA |
| ENSMUSG00000066037 | -2.04 | -4.099712558 | 2.17E-06 | Hnrnpr | heterogeneous nuclear ribonucleoprotein R |
| ENSMUSG00000057796 | -2.03 | -4.093111541 | 1.49E-03 | NA | NA |
| ENSMUSG00000039304 | -2.03 | -4.091958167 | 1.77E-07 | Tnfsf10 | tumor necrosis factor (ligand) superfamily, member 10 |
| ENSMUSG00000028282 | -2.03 | -4.091713017 | 2.40E-08 | Casp8ap2 | caspase 8 associated protein 2 |
| ENSMUSG00000033972 | -2.03 | -4.082236664 | 6.61E-07 | Zfp944 | zinc finger protein 944 |
| ENSMUSG00000071748 | -2.03 | -4.074747193 | 3.45E-05 | NA | NA |
| ENSMUSG00000078973 | -2.03 | -4.074008312 | 3.96E-04 | NA | NA |
| ENSMUSG00000084081 | -2.02 | -4.068308798 | 1.96E-05 | NA | NA |
| ENSMUSG00000030393 | -2.02 | -4.05683039 | 3.76E-04 | Zik1 | zinc finger protein interacting with K protein 1 |
| ENSMUSG00000079108 | -2.02 | -4.049790122 | 3.17E-05 | NA | NA |
| ENSMUSG00000035992 | -2.02 | -4.042958949 | 8.78E-06 | Fnip1 | folliculin interacting protein 1 |
| ENSMUSG00000078136 | -2.01 | -4.03964925 | 5.95E-03 | NA | NA |
| ENSMUSG00000064061 | -2.01 | -4.035496759 | 1.19E-06 | Dzip3 | DAZ interacting protein 3, zinc finger |
| ENSMUSG00000040321 | -2.01 | -4.033768677 | 5.17E-07 | Zfp770 | zinc finger protein 770 |
| ENSMUSG00000073176 | -2.01 | -4.032561994 | 4.56E-04 | Zfp449 | zinc finger protein 449 |
| ENSMUSG00000038646 | -2.00 | -4.013688426 | 7.42E-04 | NA | NA |
| ENSMUSG00000024795 | -2.00 | -4.0094536 | 1.41E-07 | Kif20b | kinesin family member 20B |
| ENSMUSG00000028212 | -2.00 | -3.995587377 | 2.87E-07 | Ccne2 | cyclin E2 |
| ENSMUSG00000003226 | -2.00 | -3.990052391 | 1.29E-08 | Ranbp2 | RAN binding protein 2 |
| ENSMUSG00000024943 | -1.98 | -3.940092695 | 8.63E-08 | Smc5 | structural maintenance of chromosomes 5 |
| ENSMUSG00000081557 | -1.98 | -3.938563197 | 8.28E-03 | NA | NA |
| ENSMUSG00000052920 | -1.97 | -3.925661101 | 5.93E-04 | Prkg1 | protein kinase, cGMP-dependent, type I |
| ENSMUSG00000066232 | -1.97 | -3.916784884 | 9.26E-08 | Ipo7 | importin 7 |
| ENSMUSG00000035293 | -1.97 | -3.90675286 | 7.43E-10 | G2e3 | G2/M-phase specific E3 ubiquitin ligase |
| ENSMUSG00000062949 | -1.96 | -3.896548441 | 3.18E-05 | Atp11c | ATPase, class VI, type 11C |
| ENSMUSG00000082674 | -1.94 | -3.83282803 | 1.00E-05 | NA | NA |
| ENSMUSG00000000037 | -1.94 | -3.832153329 | 3.74E-04 | Scml2 | sex comb on midleg-like 2 (Drosophila) |
| ENSMUSG00000017485 | -1.94 | -3.827258506 | 1.08E-07 | Top2b | topoisomerase (DNA) II beta |
| ENSMUSG00000052825 | -1.93 | -3.822438117 | 2.28E-05 | NA | NA |
| ENSMUSG00000037355 | -1.93 | -3.809947682 | 1.90E-05 | Uvssa | UV stimulated scaffold protein A |
| ENSMUSG00000027326 | -1.93 | -3.807503251 | 2.80E-07 | Casc5 | cancer susceptibility candidate 5 |
| ENSMUSG00000035476 | -1.93 | -3.801633459 | 7.24E-08 | Tab3 | TGF-beta activated kinase 1/MAP3K7 binding protein 3 |
| ENSMUSG00000064090 | -1.92 | -3.795185866 | 1.77E-07 | Vrk2 | vaccinia related kinase 2 |
| ENSMUSG00000020290 | -1.92 | -3.787446668 | 7.96E-12 | Xpo1 | exportin 1, CRM1 homolog (yeast) |
| ENSMUSG00000017550 | -1.92 | -3.783572161 | 5.75E-06 | Atad5 | ATPase family, AAA domain containing 5 |
| ENSMUSG00000031583 | -1.92 | -3.779089659 | 2.20E-09 | Wrn | Werner syndrome homolog (human) |
| ENSMUSG00000029253 | -1.92 | -3.775704957 | 3.06E-07 | Cenpc1 | centromere protein C1 |
| ENSMUSG00000043484 | -1.92 | -3.772763349 | 3.05E-04 | NA | NA |
| ENSMUSG00000040693 | -1.91 | -3.762945576 | 1.04E-03 | Slco4c1 | solute carrier organic anion transporter family, member 4C1 |
| ENSMUSG00000067916 | -1.91 | -3.7488679 | 4.15E-03 | Gm13139 | predicted gene 13139 |
| ENSMUSG00000081952 | -1.90 | -3.741636516 | 5.26E-04 | NA | NA |
| ENSMUSG00000025001 | -1.90 | -3.728219501 | 3.32E-07 | Hells | helicase, lymphoid specific |
| ENSMUSG00000054125 | -1.89 | -3.712652937 | 5.55E-04 | NA | NA |
| ENSMUSG00000018986 | -1.89 | -3.711713454 | 1.00E-05 | Slfn3 | schlafen 3 |
| ENSMUSG00000050077 | -1.89 | -3.698123264 | 9.70E-05 | NA | NA |
| ENSMUSG00000069743 | -1.89 | -3.694195308 | 3.41E-03 | Zfp820 | zinc finger protein 820 |
| ENSMUSG00000085606 | -1.88 | -3.676954852 | 1.33E-04 | NA | NA |
| ENSMUSG00000034317 | -1.88 | -3.672170395 | 9.91E-08 | Trim59 | tripartite motif-containing 59 |
| ENSMUSG00000069913 | -1.88 | -3.668811313 | 3.51E-06 | NA | NA |
| ENSMUSG00000021792 | -1.87 | -3.665559739 | 5.15E-06 | Fam213a | family with sequence similarity 213, member A |
| ENSMUSG00000064345 | -1.87 | -3.662168033 | 3.18E-03 | ND2 | NADH dehydrogenase subunit 2 |
| ENSMUSG00000020631 | -1.87 | -3.651492882 | 5.83E-08 | NA | NA |
| ENSMUSG00000083438 | -1.87 | -3.647143938 | 4.36E-05 | NA | NA |
| ENSMUSG00000056267 | -1.86 | -3.638019651 | 8.69E-05 | Cep70 | centrosomal protein 70 |
| ENSMUSG00000007480 | -1.86 | -3.618258283 | 4.22E-04 | Mc5r | melanocortin 5 receptor |
| ENSMUSG00000024614 | -1.85 | -3.596311596 | 9.37E-09 | Tmx3 | thioredoxin-related transmembrane protein 3 |
| ENSMUSG00000025898 | -1.84 | -3.58769411 | 1.21E-05 | Cwf19l2 | CWF19-like 2, cell cycle control (S. pombe) |
| ENSMUSG00000030641 | -1.84 | -3.583769546 | 7.68E-07 | 4632434I11Rik | RIKEN cDNA 4632434I11 gene |
| ENSMUSG00000021377 | -1.84 | -3.580006901 | 7.77E-07 | Dek | DEK oncogene (DNA binding) |
| ENSMUSG00000033213 | -1.84 | -3.572184319 | 3.59E-04 | AA467197 | expressed sequence AA467197 |
| ENSMUSG00000021109 | -1.84 | -3.570300486 | 3.07E-06 | Hif1a | hypoxia inducible factor 1, alpha subunit |
| ENSMUSG00000042029 | -1.83 | -3.566527208 | 5.23E-06 | Ncapg2 | non-SMC condensin II complex, subunit G2 |
| ENSMUSG00000040297 | -1.83 | -3.562912826 | 4.34E-08 | Suco | SUN domain containing ossification factor |
| ENSMUSG00000008307 | -1.82 | -3.540274175 | 4.93E-03 | 1700109H08Rik | RIKEN cDNA 1700109H08 gene |
| ENSMUSG00000001833 | -1.82 | -3.536574011 | 4.41E-06 | Sept7 | septin 7 |
| ENSMUSG00000076609 | -1.82 | -3.530811385 | 1.80E-03 | NA | NA |
| ENSMUSG00000060429 | -1.82 | -3.530630443 | 8.14E-05 | Sntb1 | syntrophin, basic 1 |
| ENSMUSG00000062588 | -1.81 | -3.516313049 | 2.70E-05 | NA | NA |
| ENSMUSG00000045328 | -1.81 | -3.5118072 | 1.31E-05 | Cenpe | centromere protein E |
| ENSMUSG00000044501 | -1.81 | -3.511446472 | 8.00E-04 | Zfp758 | zinc finger protein 758 |
| ENSMUSG00000040455 | -1.81 | -3.497387329 | 9.15E-09 | Usp45 | ubiquitin specific petidase 45 |
| ENSMUSG00000052019 | -1.81 | -3.494363279 | 1.46E-03 | NA | NA |
| ENSMUSG00000036769 | -1.80 | -3.489205476 | 5.18E-08 | Wdr44 | WD repeat domain 44 |
| ENSMUSG00000069045 | -1.80 | -3.475733082 | 8.41E-06 | Ddx3y | DEAD (Asp-Glu-Ala-Asp) box polypeptide 3, Y-linked |
| ENSMUSG00000034349 | -1.80 | -3.474780478 | 3.51E-07 | Smc4 | structural maintenance of chromosomes 4 |
| ENSMUSG00000069495 | -1.79 | -3.464317893 | 3.21E-06 | Epc2 | enhancer of polycomb homolog 2 (Drosophila) |
| ENSMUSG00000019966 | -1.79 | -3.457409652 | 7.67E-05 | Kitl | kit ligand |
| ENSMUSG00000032028 | -1.79 | -3.451598558 | 4.41E-06 | Nxpe2 | neurexophilin and PC-esterase domain family, member 2 |
| ENSMUSG00000027550 | -1.79 | -3.449857817 | 1.39E-06 | Lrrcc1 | leucine rich repeat and coiled-coil domain containing 1 |
| ENSMUSG00000066057 | -1.78 | -3.442579191 | 3.81E-06 | Gm1976 | predicted gene 1976 |
| ENSMUSG00000039841 | -1.78 | -3.436623612 | 1.01E-07 | Zfp800 | zinc finger protein 800 |
| ENSMUSG00000042742 | -1.78 | -3.433678588 | 2.06E-04 | B630005N14Rik | RIKEN cDNA B630005N14 gene |
| ENSMUSG00000038344 | -1.78 | -3.43280736 | 3.47E-08 | Txlng | taxilin gamma |
| ENSMUSG00000026605 | -1.78 | -3.432361823 | 9.18E-07 | Cenpf | centromere protein F |
| ENSMUSG00000020300 | -1.78 | -3.431522403 | 1.63E-04 | Cpeb4 | cytoplasmic polyadenylation element binding protein 4 |
| ENSMUSG00000033031 | -1.78 | -3.424427543 | 3.05E-07 | C330027C09Rik | RIKEN cDNA C330027C09 gene |
| ENSMUSG00000028256 | -1.77 | -3.417355172 | 5.68E-05 | Odf2l | outer dense fiber of sperm tails 2-like |
| ENSMUSG00000074154 | -1.77 | -3.415185288 | 4.46E-04 | NA | NA |
| ENSMUSG00000051579 | -1.77 | -3.406864665 | 2.19E-04 | Tceal8 | transcription elongation factor A (SII)-like 8 |
| ENSMUSG00000031229 | -1.76 | -3.397445504 | 5.93E-07 | Atrx | alpha thalassemia/mental retardation syndrome X-linked homolog (human) |
| ENSMUSG00000084862 | -1.76 | -3.396761956 | 8.10E-03 | NA | NA |
| ENSMUSG00000021712 | -1.76 | -3.383975056 | 4.95E-07 | Trim23 | tripartite motif-containing 23 |
| ENSMUSG00000074732 | -1.76 | -3.382387108 | 7.95E-03 | NA | NA |
| ENSMUSG00000028133 | -1.76 | -3.381332748 | 2.67E-05 | Rwdd3 | RWD domain containing 3 |
| ENSMUSG00000031095 | -1.76 | -3.376146924 | 2.48E-07 | Cul4b | cullin 4B |
| ENSMUSG00000000787 | -1.75 | -3.37381259 | 4.71E-05 | Ddx3x | DEAD/H (Asp-Glu-Ala-Asp/His) box polypeptide 3, X-linked |
| ENSMUSG00000032551 | -1.75 | -3.370155273 | 3.05E-06 | 1110059G10Rik | RIKEN cDNA 1110059G10 gene |
| ENSMUSG00000060445 | -1.75 | -3.36938811 | 2.58E-03 | Sycp2 | synaptonemal complex protein 2 |
| ENSMUSG00000031174 | -1.75 | -3.365962141 | 7.00E-05 | Rpgr | retinitis pigmentosa GTPase regulator |
| ENSMUSG00000035133 | -1.74 | -3.349578943 | 1.31E-04 | Arhgap5 | Rho GTPase activating protein 5 |
| ENSMUSG00000027981 | -1.74 | -3.345356773 | 3.59E-05 | Rnpc3 | RNA-binding region (RNP1, RRM) containing 3 |
| ENSMUSG00000033186 | -1.74 | -3.340007315 | 1.29E-06 | Mzt1 | mitotic spindle organizing protein 1 |
| ENSMUSG00000029202 | -1.74 | -3.337044521 | 5.91E-07 | Pds5a | PDS5, regulator of cohesion maintenance, homolog A (S. cerevisiae) |
| ENSMUSG00000027115 | -1.74 | -3.330427657 | 2.21E-06 | Kif18a | kinesin family member 18A |
| ENSMUSG00000031592 | -1.74 | -3.329998266 | 3.35E-05 | Pcm1 | pericentriolar material 1 |
| ENSMUSG00000037315 | -1.74 | -3.329852914 | 2.66E-07 | Jade3 | jade family PHD finger 3 |
| ENSMUSG00000068188 | -1.73 | -3.32837663 | 6.87E-04 | NA | NA |
| ENSMUSG00000085829 | -1.73 | -3.315399566 | 4.38E-03 | NA | NA |
| ENSMUSG00000052912 | -1.73 | -3.315179181 | 2.95E-05 | Smarca5-ps | SWI/SNF related, matrix associated, actin depenent ragulator of chromatin, subfamily a, member 5, pseudogene |
| ENSMUSG00000026775 | -1.73 | -3.31322038 | 6.90E-09 | Yme1l1 | YME1-like 1 (S. cerevisiae) |
| ENSMUSG00000006678 | -1.73 | -3.312391643 | 1.10E-08 | Pola1 | polymerase (DNA directed), alpha 1 |
| ENSMUSG00000002190 | -1.73 | -3.311155821 | 3.64E-03 | Clgn | calmegin |
| ENSMUSG00000025907 | -1.73 | -3.307409702 | 3.89E-08 | Rb1cc1 | RB1-inducible coiled-coil 1 |
| ENSMUSG00000017548 | -1.72 | -3.304491455 | 3.89E-08 | Suz12 | suppressor of zeste 12 homolog (Drosophila) |
| ENSMUSG00000025766 | -1.72 | -3.295722239 | 1.22E-05 | D3Ertd751e | DNA segment, Chr 3, ERATO Doi 751, expressed |
| ENSMUSG00000031715 | -1.72 | -3.29403303 | 9.18E-06 | Smarca5 | SWI/SNF related, matrix associated, actin dependent regulator of chromatin, subfamily a, member 5 |
| ENSMUSG00000056145 | -1.72 | -3.293796036 | 7.03E-03 | AI504432 | expressed sequence AI504432 |
| ENSMUSG00000021843 | -1.71 | -3.279970501 | 2.18E-05 | Ktn1 | kinectin 1 |
| ENSMUSG00000059834 | -1.71 | -3.272788718 | 2.68E-04 | Sclt1 | sodium channel and clathrin linker 1 |
| ENSMUSG00000067931 | -1.71 | -3.270800913 | 1.02E-05 | Zfp948 | zinc finger protein 948 |
| ENSMUSG00000071528 | -1.71 | -3.267792513 | 5.96E-03 | Usmg5 | upregulated during skeletal muscle growth 5 |
| ENSMUSG00000030528 | -1.71 | -3.267005049 | 1.35E-05 | Blm | Bloom syndrome, RecQ helicase-like |
| ENSMUSG00000083849 | -1.70 | -3.259380024 | 2.40E-03 | NA | NA |
| ENSMUSG00000046311 | -1.70 | -3.257936577 | 6.25E-10 | Zfp62 | zinc finger protein 62 |
| ENSMUSG00000024974 | -1.70 | -3.254434261 | 2.35E-07 | Smc3 | structural maintenance of chromosomes 3 |
| ENSMUSG00000037369 | -1.70 | -3.254161155 | 8.58E-09 | Kdm6a | lysine (K)-specific demethylase 6A |
| ENSMUSG00000086587 | -1.70 | -3.253952363 | 1.05E-03 | NA | NA |
| ENSMUSG00000037236 | -1.70 | -3.248141656 | 1.58E-08 | Matr3 | matrin 3 |
| ENSMUSG00000078135 | -1.70 | -3.248122667 | 1.39E-05 | NA | NA |
| ENSMUSG00000034653 | -1.70 | -3.247056866 | 9.80E-05 | Ythdc2 | YTH domain containing 2 |
| ENSMUSG00000051255 | -1.70 | -3.242846946 | 1.65E-04 | NA | NA |
| ENSMUSG00000027699 | -1.70 | -3.241527871 | 3.09E-07 | Ect2 | ect2 oncogene |
| ENSMUSG00000054693 | -1.69 | -3.229797978 | 2.87E-08 | Adam10 | a disintegrin and metallopeptidase domain 10 |
| ENSMUSG00000020493 | -1.69 | -3.227266699 | 1.41E-07 | Prr11 | proline rich 11 |
| ENSMUSG00000040651 | -1.69 | -3.222927974 | 9.01E-04 | D14Abb1e | DNA segment, Chr 14, Abbott 1 expressed |
| ENSMUSG00000073492 | -1.69 | -3.222791257 | 3.32E-04 | NA | NA |
| ENSMUSG00000032556 | -1.69 | -3.220063832 | 9.20E-03 | Bfsp2 | beaded filament structural protein 2, phakinin |
| ENSMUSG00000035236 | -1.69 | -3.218584905 | 2.47E-05 | Scai | suppressor of cancer cell invasion |
| ENSMUSG00000066307 | -1.69 | -3.216367446 | 3.91E-04 | NA | NA |
| ENSMUSG00000027281 | -1.69 | -3.21607982 | 3.20E-07 | Slx4ip | SLX4 interacting protein |
| ENSMUSG00000021549 | -1.69 | -3.215570248 | 1.16E-06 | Rasa1 | RAS p21 protein activator 1 |
| ENSMUSG00000036943 | -1.68 | -3.213427371 | 1.71E-06 | Rab8b | RAB8B, member RAS oncogene family |
| ENSMUSG00000059588 | -1.68 | -3.212940148 | 6.18E-05 | Calcrl | calcitonin receptor-like |
| ENSMUSG00000032864 | -1.68 | -3.209560555 | 2.06E-03 | Rag2 | recombination activating gene 2 |
| ENSMUSG00000063810 | -1.68 | -3.207550421 | 1.37E-05 | Alms1 | Alstrom syndrome 1 |
| ENSMUSG00000026357 | -1.68 | -3.202454419 | 5.79E-06 | Rgs18 | regulator of G-protein signaling 18 |
| ENSMUSG00000079472 | -1.68 | -3.201462992 | 1.60E-03 | NA | NA |
| ENSMUSG00000068882 | -1.68 | -3.197104376 | 1.83E-05 | Ssb | Sjogren syndrome antigen B |
| ENSMUSG00000054737 | -1.68 | -3.197079886 | 2.70E-05 | Zfp182 | zinc finger protein 182 |
| ENSMUSG00000029403 | -1.68 | -3.196714383 | 7.85E-04 | Cdkl2 | cyclin-dependent kinase-like 2 (CDC2-related kinase) |
| ENSMUSG00000037475 | -1.68 | -3.195450419 | 8.85E-08 | Thoc2 | THO complex 2 |
| ENSMUSG00000067942 | -1.67 | -3.186101294 | 2.33E-08 | Zfp160 | zinc finger protein 160 |
| ENSMUSG00000058624 | -1.67 | -3.179651535 | 1.30E-06 | Gda | guanine deaminase |
| ENSMUSG00000030165 | -1.67 | -3.178866982 | 6.50E-04 | Klrd1 | killer cell lectin-like receptor, subfamily D, member 1 |
| ENSMUSG00000022876 | -1.67 | -3.177958759 | 4.67E-05 | Samsn1 | SAM domain, SH3 domain and nuclear localization signals, 1 |
| ENSMUSG00000074910 | -1.67 | -3.177833447 | 6.25E-06 | NA | NA |
| ENSMUSG00000058883 | -1.66 | -3.168688547 | 7.71E-03 | Zfp708 | zinc finger protein 708 |
| ENSMUSG00000075297 | -1.66 | -3.165053244 | 5.58E-03 | H60b | histocompatibility 60b |
| ENSMUSG00000026098 | -1.66 | -3.162279123 | 1.03E-05 | Pms1 | postmeiotic segregation increased 1 (S. cerevisiae) |
| ENSMUSG00000083899 | -1.66 | -3.159930702 | 1.78E-04 | NA | NA |
| ENSMUSG00000056383 | -1.65 | -3.147731366 | 6.45E-07 | AI987944 | expressed sequence AI987944 |
| ENSMUSG00000021537 | -1.65 | -3.147501665 | 1.09E-04 | Cetn3 | centrin 3 |
| ENSMUSG00000028333 | -1.65 | -3.146322635 | 6.88E-05 | Anp32b | acidic (leucine-rich) nuclear phosphoprotein 32 family, member B |
| ENSMUSG00000053051 | -1.65 | -3.14511099 | 4.20E-05 | NA | NA |
| ENSMUSG00000044150 | -1.65 | -3.139234978 | 1.81E-04 | A830080D01Rik | RIKEN cDNA A830080D01 gene |
| ENSMUSG00000042271 | -1.65 | -3.13880086 | 1.32E-05 | Nxt2 | nuclear transport factor 2-like export factor 2 |
| ENSMUSG00000085687 | -1.65 | -3.137640303 | 6.92E-04 | NA | NA |
| ENSMUSG00000053347 | -1.65 | -3.135636086 | 3.42E-05 | Zfp943 | zinc finger prtoein 943 |
| ENSMUSG00000032423 | -1.65 | -3.132869203 | 9.37E-09 | Syncrip | synaptotagmin binding, cytoplasmic RNA interacting protein |
| ENSMUSG00000085917 | -1.65 | -3.132115489 | 1.18E-03 | NA | NA |
| ENSMUSG00000024991 | -1.65 | -3.12801922 | 1.10E-07 | Eif3a | eukaryotic translation initiation factor 3, subunit A |
| ENSMUSG00000071350 | -1.65 | -3.127747236 | 1.10E-08 | Setdb2 | SET domain, bifurcated 2 |
| ENSMUSG00000029178 | -1.64 | -3.124618927 | 3.48E-05 | Klf3 | Kruppel-like factor 3 (basic) |
| ENSMUSG00000004319 | -1.64 | -3.123936147 | 6.98E-06 | Clcn3 | chloride channel 3 |
| ENSMUSG00000032905 | -1.64 | -3.123859272 | 3.89E-05 | Atg12 | autophagy related 12 |
| ENSMUSG00000051285 | -1.64 | -3.115496113 | 1.77E-07 | Pcmtd1 | protein-L-isoaspartate (D-aspartate) O-methyltransferase domain containing 1 |
| ENSMUSG00000041685 | -1.64 | -3.114485846 | 2.12E-05 | Fcho2 | FCH domain only 2 |
| ENSMUSG00000052572 | -1.64 | -3.108325998 | 2.00E-05 | Dlg2 | discs, large homolog 2 (Drosophila) |
| ENSMUSG00000062743 | -1.63 | -3.103477788 | 9.57E-04 | Zfp677 | zinc finger protein 677 |
| ENSMUSG00000087153 | -1.63 | -3.100746016 | 1.95E-04 | LOC101056094 | cadherin-11-like |
| ENSMUSG00000031010 | -1.63 | -3.100362391 | 1.87E-05 | Usp9x | ubiquitin specific peptidase 9, X chromosome |
| ENSMUSG00000036768 | -1.63 | -3.099223439 | 2.48E-06 | Kif15 | kinesin family member 15 |
| ENSMUSG00000025912 | -1.63 | -3.095709217 | 7.31E-04 | Mybl1 | myeloblastosis oncogene-like 1 |
| ENSMUSG00000031517 | -1.63 | -3.094798827 | 8.27E-05 | Gpm6a | glycoprotein m6a |
| ENSMUSG00000030660 | -1.63 | -3.094095204 | 9.61E-04 | Pik3c2a | phosphatidylinositol 3-kinase, C2 domain containing, alpha polypeptide |
| ENSMUSG00000028175 | -1.63 | -3.092109276 | 2.06E-03 | Depdc1a | DEP domain containing 1a |
| ENSMUSG00000078914 | -1.63 | -3.091543569 | 3.75E-03 | NA | NA |
| ENSMUSG00000039789 | -1.63 | -3.086318994 | 5.82E-06 | Zfp597 | zinc finger protein 597 |
| ENSMUSG00000046111 | -1.62 | -3.072181071 | 2.63E-06 | 5830418K08Rik | RIKEN cDNA 5830418K08 gene |
| ENSMUSG00000062270 | -1.62 | -3.071208384 | 1.46E-03 | NA | NA |
| ENSMUSG00000032293 | -1.62 | -3.069520935 | 2.19E-04 | Ireb2 | iron responsive element binding protein 2 |
| ENSMUSG00000048379 | -1.62 | -3.069402143 | 2.85E-04 | Socs4 | suppressor of cytokine signaling 4 |
| ENSMUSG00000022126 | -1.62 | -3.068937732 | 7.53E-03 | Irg1 | immunoresponsive gene 1 |
| ENSMUSG00000024423 | -1.62 | -3.068124413 | 6.90E-09 | Impact | impact, RWD domain protein |
| ENSMUSG00000016984 | -1.62 | -3.066001748 | 3.27E-06 | Etaa1 | Ewing tumor-associated antigen 1 |
| ENSMUSG00000022034 | -1.61 | -3.060836143 | 5.96E-06 | Esco2 | establishment of cohesion 1 homolog 2 (S. cerevisiae) |
| ENSMUSG00000031644 | -1.61 | -3.057233283 | 4.09E-05 | Nek1 | NIMA (never in mitosis gene a)-related expressed kinase 1 |
| ENSMUSG00000081583 | -1.61 | -3.054344987 | 4.94E-03 | NA | NA |
| ENSMUSG00000074165 | -1.61 | -3.050439404 | 1.21E-04 | Zfp788 | zinc finger protein 788 |
| ENSMUSG00000028132 | -1.61 | -3.049813559 | 4.03E-06 | Tmem56 | transmembrane protein 56 |
| ENSMUSG00000061136 | -1.61 | -3.049250026 | 1.94E-07 | Prpf40a | PRP40 pre-mRNA processing factor 40 homolog A (yeast) |
| ENSMUSG00000054387 | -1.61 | -3.045205513 | 6.76E-04 | Mdm4 | transformed mouse 3T3 cell double minute 4 |
| ENSMUSG00000031939 | -1.60 | -3.040941007 | 6.59E-06 | Taf1d | TATA box binding protein (Tbp)-associated factor, RNA polymerase I, D |
| ENSMUSG00000084094 | -1.60 | -3.040697943 | 1.88E-03 | NA | NA |
| ENSMUSG00000027677 | -1.60 | -3.038741113 | 1.12E-09 | Ttc14 | tetratricopeptide repeat domain 14 |
| ENSMUSG00000025862 | -1.60 | -3.038124075 | 4.12E-07 | Stag2 | stromal antigen 2 |
| ENSMUSG00000024524 | -1.60 | -3.036465251 | 5.93E-03 | Gnal | guanine nucleotide binding protein, alpha stimulating, olfactory type |
| ENSMUSG00000036022 | -1.60 | -3.031620967 | 4.78E-04 | Fam122b | family with sequence similarity 122, member B |
| ENSMUSG00000082876 | -1.60 | -3.02310371 | 2.41E-04 | NA | NA |
| ENSMUSG00000057399 | -1.59 | -3.019145766 | 3.03E-03 | NA | NA |
| ENSMUSG00000059142 | -1.59 | -3.018843487 | 9.01E-04 | Zfp945 | zinc finger protein 945 |
| ENSMUSG00000034825 | -1.59 | -3.018419684 | 4.61E-05 | Nrip3 | nuclear receptor interacting protein 3 |
| ENSMUSG00000038587 | -1.59 | -3.01546119 | 2.52E-07 | Akap12 | A kinase (PRKA) anchor protein (gravin) 12 |
| ENSMUSG00000083364 | -1.59 | -3.015041641 | 6.71E-06 | NA | NA |
| ENSMUSG00000042489 | -1.59 | -3.013928389 | 5.79E-06 | Clspn | claspin |
| ENSMUSG00000026977 | -1.59 | -3.013622714 | 2.99E-08 | March7 | membrane-associated ring finger (C3HC4) 7 |
| ENSMUSG00000022686 | -1.59 | -3.010896383 | 3.97E-05 | B3gnt5 | UDP-GlcNAc:betaGal beta-1,3-N-acetylglucosaminyltransferase 5 |
| ENSMUSG00000048149 | -1.59 | -3.007637633 | 8.49E-05 | NA | NA |
| ENSMUSG00000082925 | -1.59 | -3.006267865 | 5.58E-04 | NA | NA |
| ENSMUSG00000026779 | -1.59 | -3.001627228 | 5.04E-06 | Mastl | microtubule associated serine/threonine kinase-like |
| ENSMUSG00000021733 | -1.59 | -3.001264231 | 3.08E-06 | Slc4a7 | solute carrier family 4, sodium bicarbonate cotransporter, member 7 |
| ENSMUSG00000083819 | -1.58 | -2.999744755 | 8.77E-04 | NA | NA |
| ENSMUSG00000015880 | -1.58 | -2.986317677 | 1.79E-05 | Ncapg | non-SMC condensin I complex, subunit G |
| ENSMUSG00000049658 | -1.58 | -2.98505407 | 1.49E-06 | Bdp1 | B double prime 1, subunit of RNA polymerase III transcription initiation factor IIIB |
| ENSMUSG00000020024 | -1.57 | -2.977919477 | 9.88E-05 | Ccdc41 | coiled-coil domain containing 41 |
| ENSMUSG00000033799 | -1.57 | -2.974797408 | 2.80E-07 | BC016423 | cDNA sequence BC016423 |
| ENSMUSG00000064289 | -1.57 | -2.973705648 | 3.27E-06 | Tank | TRAF family member-associated Nf-kappa B activator |
| ENSMUSG00000052714 | -1.57 | -2.973380265 | 3.88E-04 | NA | NA |
| ENSMUSG00000025626 | -1.57 | -2.970636071 | 6.42E-10 | Phf6 | PHD finger protein 6 |
| ENSMUSG00000029422 | -1.57 | -2.967623827 | 1.52E-05 | Rsrc2 | arginine/serine-rich coiled-coil 2 |
| ENSMUSG00000048279 | -1.57 | -2.964809044 | 5.16E-06 | Sacs | sacsin |
| ENSMUSG00000066107 | -1.57 | -2.963447372 | 1.00E-03 | Gm12666 | predicted gene 12666 |
| ENSMUSG00000029366 | -1.57 | -2.961024966 | 5.05E-11 | Dck | deoxycytidine kinase |
| ENSMUSG00000036202 | -1.57 | -2.959255374 | 2.75E-05 | Rif1 | Rap1 interacting factor 1 homolog (yeast) |
| ENSMUSG00000028484 | -1.57 | -2.958792985 | 8.79E-07 | Psip1 | PC4 and SFRS1 interacting protein 1 |
| ENSMUSG00000036501 | -1.56 | -2.956279507 | 2.89E-06 | Fam13b | family with sequence similarity 13, member B |
| ENSMUSG00000024680 | -1.56 | -2.954232479 | 3.00E-03 | Ms4a2 | membrane-spanning 4-domains, subfamily A, member 2 |
| ENSMUSG00000004110 | -1.56 | -2.946688439 | 8.05E-04 | Cacna1e | calcium channel, voltage-dependent, R type, alpha 1E subunit |
| ENSMUSG00000003452 | -1.56 | -2.94539482 | 9.93E-04 | Bicd1 | bicaudal D homolog 1 (Drosophila) |
| ENSMUSG00000044749 | -1.56 | -2.942918362 | 3.49E-03 | Abca6 | ATP-binding cassette, sub-family A (ABC1), member 6 |
| ENSMUSG00000020189 | -1.56 | -2.940398624 | 1.14E-06 | Osbpl8 | oxysterol binding protein-like 8 |
| ENSMUSG00000035492 | -1.56 | -2.938769775 | 8.78E-06 | NA | NA |
| ENSMUSG00000048047 | -1.55 | -2.938104649 | 7.28E-04 | Zbtb33 | zinc finger and BTB domain containing 33 |
| ENSMUSG00000025050 | -1.55 | -2.937979151 | 8.58E-05 | Pcgf6 | polycomb group ring finger 6 |
| ENSMUSG00000089686 | -1.55 | -2.937730967 | 4.89E-03 | NA | NA |
| ENSMUSG00000051147 | -1.55 | -2.93701866 | 7.56E-07 | Nat2 | N-acetyltransferase 2 (arylamine N-acetyltransferase) |
| ENSMUSG00000022514 | -1.55 | -2.935291682 | 3.57E-04 | Il1rap | interleukin 1 receptor accessory protein |
| ENSMUSG00000048490 | -1.55 | -2.933332686 | 5.52E-05 | Nrip1 | nuclear receptor interacting protein 1 |
| ENSMUSG00000028675 | -1.55 | -2.929863724 | 3.41E-06 | Pnrc2 | proline-rich nuclear receptor coactivator 2 |
| ENSMUSG00000022867 | -1.55 | -2.921850909 | 6.61E-07 | Usp25 | ubiquitin specific peptidase 25 |
| ENSMUSG00000030154 | -1.55 | -2.92089303 | 6.57E-05 | Klrb1f | killer cell lectin-like receptor subfamily B member 1F |
| ENSMUSG00000014226 | -1.55 | -2.920134407 | 1.43E-04 | Cacybp | calcyclin binding protein |
| ENSMUSG00000081718 | -1.54 | -2.917542521 | 1.85E-03 | NA | NA |
| ENSMUSG00000030929 | -1.54 | -2.9166884 | 1.76E-06 | Eri2 | exoribonuclease 2 |
| ENSMUSG00000071266 | -1.54 | -2.915696056 | 2.04E-03 | Zfp946 | zinc finger protein 946 |
| ENSMUSG00000073905 | -1.54 | -2.915244613 | 7.95E-06 | NA | NA |
| ENSMUSG00000031647 | -1.54 | -2.914358255 | 1.65E-05 | Mfap3l | microfibrillar-associated protein 3-like |
| ENSMUSG00000034480 | -1.54 | -2.912832796 | 2.54E-05 | Diap2 | diaphanous homolog 2 (Drosophila) |
| ENSMUSG00000028180 | -1.54 | -2.911692398 | 4.69E-09 | Zranb2 | zinc finger, RAN-binding domain containing 2 |
| ENSMUSG00000033904 | -1.54 | -2.911245413 | 1.67E-06 | Ccp110 | centriolar coiled coil protein 110 |
| ENSMUSG00000031433 | -1.54 | -2.908706779 | 4.16E-03 | Rbm41 | RNA binding motif protein 41 |
| ENSMUSG00000033952 | -1.54 | -2.903489563 | 2.91E-05 | Aspm | asp (abnormal spindle)-like, microcephaly associated (Drosophila) |
| ENSMUSG00000023919 | -1.54 | -2.901394794 | 3.35E-04 | Cenpq | centromere protein Q |
| ENSMUSG00000022314 | -1.53 | -2.896868091 | 1.60E-06 | Rad21 | RAD21 homolog (S. pombe) |
| ENSMUSG00000086453 | -1.53 | -2.896382474 | 1.81E-03 | NA | NA |
| ENSMUSG00000032221 | -1.53 | -2.895917259 | 3.98E-04 | Mns1 | meiosis-specific nuclear structural protein 1 |
| ENSMUSG00000036676 | -1.53 | -2.895535882 | 1.25E-05 | Tmtc3 | transmembrane and tetratricopeptide repeat containing 3 |
| ENSMUSG00000039967 | -1.53 | -2.894932858 | 7.55E-04 | Zfp292 | zinc finger protein 292 |
| ENSMUSG00000044098 | -1.53 | -2.894812245 | 4.25E-03 | Rsbn1 | rosbin, round spermatid basic protein 1 |
| ENSMUSG00000078933 | -1.53 | -2.894090185 | 3.04E-04 | NA | NA |
| ENSMUSG00000024498 | -1.53 | -2.884053276 | 1.49E-05 | Tcerg1 | transcription elongation regulator 1 (CA150) |
| ENSMUSG00000027641 | -1.53 | -2.880235956 | 1.29E-06 | Rbl1 | retinoblastoma-like 1 (p107) |
| ENSMUSG00000047036 | -1.53 | -2.87970197 | 1.01E-03 | Zfp445 | zinc finger protein 445 |
| ENSMUSG00000015533 | -1.52 | -2.875383145 | 8.93E-03 | Itga2 | integrin alpha 2 |
| ENSMUSG00000084953 | -1.52 | -2.872898572 | 3.11E-03 | NA | NA |
| ENSMUSG00000028134 | -1.52 | -2.872684694 | 6.01E-05 | Ptbp2 | polypyrimidine tract binding protein 2 |
| ENSMUSG00000057396 | -1.52 | -2.87196918 | 2.59E-03 | Zfp759 | zinc finger protein 759 |
| ENSMUSG00000074802 | -1.52 | -2.871664993 | 1.73E-05 | Gas2l3 | growth arrest-specific 2 like 3 |
| ENSMUSG00000026582 | -1.52 | -2.868224005 | 1.48E-05 | Sele | selectin, endothelial cell |
| ENSMUSG00000038174 | -1.52 | -2.867241661 | 4.43E-04 | Fam126b | family with sequence similarity 126, member B |
| ENSMUSG00000067878 | -1.52 | -2.866825113 | 1.86E-03 | Mtap7d3 | MAP7 domain containing 3 |
| ENSMUSG00000035310 | -1.52 | -2.86649463 | 8.60E-05 | Lin54 | lin-54 homolog (C. elegans) |
| ENSMUSG00000011831 | -1.52 | -2.862976649 | 4.94E-08 | Evi5 | ecotropic viral integration site 5 |
| ENSMUSG00000027379 | -1.52 | -2.862288103 | 4.60E-07 | Bub1 | budding uninhibited by benzimidazoles 1 homolog (S. cerevisiae) |
| ENSMUSG00000031246 | -1.52 | -2.861472631 | 6.09E-10 | Sh3bgrl | SH3-binding domain glutamic acid-rich protein like |
| ENSMUSG00000072969 | -1.52 | -2.860931216 | 6.54E-05 | Armcx5 | armadillo repeat containing, X-linked 5 |
| ENSMUSG00000027962 | -1.51 | -2.856657052 | 1.66E-07 | Vcam1 | vascular cell adhesion molecule 1 |
| ENSMUSG00000032253 | -1.51 | -2.855505975 | 8.49E-05 | Phip | pleckstrin homology domain interacting protein |
| ENSMUSG00000026532 | -1.51 | -2.854281015 | 1.10E-04 | Spta1 | spectrin alpha, erythrocytic 1 |
| ENSMUSG00000082840 | -1.51 | -2.851404177 | 4.93E-03 | NA | NA |
| ENSMUSG00000019907 | -1.51 | -2.850627297 | 2.88E-05 | Ppp1r12a | protein phosphatase 1, regulatory (inhibitor) subunit 12A |
| ENSMUSG00000036863 | -1.51 | -2.849896435 | 1.07E-04 | Syde2 | synapse defective 1, Rho GTPase, homolog 2 (C. elegans) |
| ENSMUSG00000069793 | -1.51 | -2.844585929 | 2.06E-04 | Slfn9 | schlafen 9 |
| ENSMUSG00000028560 | -1.51 | -2.8438847 | 2.45E-08 | Usp1 | ubiquitin specific peptidase 1 |
| ENSMUSG00000023852 | -1.51 | -2.841762128 | 1.53E-05 | Chd1 | chromodomain helicase DNA binding protein 1 |
| ENSMUSG00000048489 | -1.51 | -2.841552755 | 4.51E-04 | 8430408G22Rik | RIKEN cDNA 8430408G22 gene |
| ENSMUSG00000040170 | -1.51 | -2.841388673 | 1.31E-05 | Fmo2 | flavin containing monooxygenase 2 |
| ENSMUSG00000034761 | -1.51 | -2.840391867 | 9.42E-07 | Map4k5 | mitogen-activated protein kinase kinase kinase kinase 5 |
| ENSMUSG00000010608 | -1.50 | -2.834493568 | 9.60E-08 | Rbm25 | RNA binding motif protein 25 |
| ENSMUSG00000011960 | -1.50 | -2.83312526 | 7.57E-03 | Ccnt1 | cyclin T1 |
| ENSMUSG00000067869 | -1.50 | -2.831368066 | 6.45E-06 | NA | NA |
| ENSMUSG00000051235 | -1.50 | -2.828456685 | 7.28E-06 | Gen1 | Gen homolog 1, endonuclease (Drosophila) |
| ENSMUSG00000061544 | -1.50 | -2.824608768 | 4.01E-03 | Zfp229 | zinc finger protein 229 |
| ENSMUSG00000066798 | -1.49 | -2.816604726 | 7.07E-04 | Zbtb6 | zinc finger and BTB domain containing 6 |
| ENSMUSG00000055900 | -1.49 | -2.815254874 | 1.75E-05 | Tmem69 | transmembrane protein 69 |
| ENSMUSG00000026712 | -1.49 | -2.815009692 | 5.14E-06 | Mrc1 | mannose receptor, C type 1 |
| ENSMUSG00000042595 | -1.49 | -2.814361732 | 5.45E-03 | Fam199x | family with sequence similarity 199, X-linked |
| ENSMUSG00000028030 | -1.49 | -2.81406748 | 5.23E-03 | Tbck | TBC1 domain containing kinase |
| ENSMUSG00000027109 | -1.49 | -2.813189947 | 3.30E-06 | Sp3 | trans-acting transcription factor 3 |
| ENSMUSG00000030016 | -1.49 | -2.812126035 | 1.78E-05 | Zfml | zinc finger, matrin-like |
| ENSMUSG00000049092 | -1.49 | -2.810050736 | 3.97E-04 | Gpr137c | G protein-coupled receptor 137C |
| ENSMUSG00000048327 | -1.49 | -2.809629264 | 1.42E-08 | Ckap2l | cytoskeleton associated protein 2-like |
| ENSMUSG00000004591 | -1.49 | -2.808091557 | 3.54E-05 | Pkn2 | protein kinase N2 |
| ENSMUSG00000024491 | -1.49 | -2.806606237 | 1.66E-04 | Rbm27 | RNA binding motif protein 27 |
| ENSMUSG00000053965 | -1.49 | -2.803810975 | 2.38E-03 | Pde5a | phosphodiesterase 5A, cGMP-specific |
| ENSMUSG00000051329 | -1.49 | -2.80376562 | 5.92E-07 | Nup160 | nucleoporin 160 |
| ENSMUSG00000039396 | -1.49 | -2.800580267 | 6.06E-05 | Neil3 | nei like 3 (E. coli) |
| ENSMUSG00000035840 | -1.49 | -2.800315985 | 1.75E-04 | Lysmd3 | LysM, putative peptidoglycan-binding, domain containing 3 |
| ENSMUSG00000021936 | -1.49 | -2.799566997 | 3.15E-06 | Mapk8 | mitogen-activated protein kinase 8 |
| ENSMUSG00000037922 | -1.48 | -2.797709755 | 5.07E-04 | Bank1 | B cell scaffold protein with ankyrin repeats 1 |
| ENSMUSG00000038379 | -1.48 | -2.797344835 | 8.79E-07 | Ttk | Ttk protein kinase |
| ENSMUSG00000087696 | -1.48 | -2.791946796 | 9.13E-03 | NA | NA |
| ENSMUSG00000033107 | -1.48 | -2.790694536 | 2.68E-03 | Rnf125 | ring finger protein 125 |
| ENSMUSG00000036916 | -1.48 | -2.789810061 | 1.07E-03 | Zfp280c | zinc finger protein 280C |
| ENSMUSG00000067851 | -1.48 | -2.784209851 | 1.02E-04 | Arfgef1 | ADP-ribosylation factor guanine nucleotide-exchange factor 1(brefeldin A-inhibited) |
| ENSMUSG00000030254 | -1.48 | -2.780425755 | 8.93E-08 | Rad18 | RAD18 homolog (S. cerevisiae) |
| ENSMUSG00000043019 | -1.47 | -2.777210818 | 6.20E-04 | Edem3 | ER degradation enhancer, mannosidase alpha-like 3 |
| ENSMUSG00000058093 | -1.47 | -2.773679154 | 2.13E-04 | AA987161 | expressed sequence AA987161 |
| ENSMUSG00000044583 | -1.47 | -2.77271699 | 1.37E-05 | Tlr7 | toll-like receptor 7 |
| ENSMUSG00000034297 | -1.47 | -2.770635652 | 1.63E-05 | Med13 | mediator complex subunit 13 |
| ENSMUSG00000025860 | -1.47 | -2.77053854 | 6.60E-07 | Xiap | X-linked inhibitor of apoptosis |
| ENSMUSG00000036777 | -1.47 | -2.769369588 | 4.39E-05 | Anln | anillin, actin binding protein |
| ENSMUSG00000025531 | -1.47 | -2.769291552 | 3.87E-06 | Chm | choroidermia |
| ENSMUSG00000084098 | -1.47 | -2.766220086 | 1.03E-03 | NA | NA |
| ENSMUSG00000022789 | -1.47 | -2.766178464 | 1.11E-08 | Dnm1l | dynamin 1-like |
| ENSMUSG00000051316 | -1.47 | -2.763418181 | 3.30E-04 | Taf7 | TAF7 RNA polymerase II, TATA box binding protein (TBP)-associated factor |
| ENSMUSG00000022205 | -1.47 | -2.761694309 | 5.56E-03 | Sub1 | SUB1 homolog (S. cerevisiae) |
| ENSMUSG00000015749 | -1.46 | -2.760622229 | 4.41E-06 | Anp32e | acidic (leucine-rich) nuclear phosphoprotein 32 family, member E |
| ENSMUSG00000034574 | -1.46 | -2.758040364 | 2.52E-07 | Daam1 | dishevelled associated activator of morphogenesis 1 |
| ENSMUSG00000031200 | -1.46 | -2.757279291 | 1.52E-04 | NA | NA |
| ENSMUSG00000064367 | -1.46 | -2.755559301 | 7.29E-04 | ND5 | NADH dehydrogenase subunit 5 |
| ENSMUSG00000022309 | -1.46 | -2.755383972 | 1.77E-06 | Angpt1 | angiopoietin 1 |
| ENSMUSG00000028211 | -1.46 | -2.754056841 | 1.33E-05 | Trp53inp1 | transformation related protein 53 inducible nuclear protein 1 |
| ENSMUSG00000070372 | -1.46 | -2.75267859 | 1.09E-06 | Capza1 | capping protein (actin filament) muscle Z-line, alpha 1 |
| ENSMUSG00000083736 | -1.46 | -2.748599351 | 1.60E-05 | NA | NA |
| ENSMUSG00000014074 | -1.46 | -2.747105961 | 1.17E-06 | Rnf168 | ring finger protein 168 |
| ENSMUSG00000070392 | -1.46 | -2.746445667 | 7.33E-03 | NA | NA |
| ENSMUSG00000037808 | -1.46 | -2.745524939 | 1.22E-05 | Fam76b | family with sequence similarity 76, member B |
| ENSMUSG00000050912 | -1.46 | -2.744372576 | 1.61E-07 | Tmem123 | transmembrane protein 123 |
| ENSMUSG00000024079 | -1.46 | -2.741731067 | 7.68E-08 | Eif2ak2 | eukaryotic translation initiation factor 2-alpha kinase 2 |
| ENSMUSG00000041763 | -1.45 | -2.734933537 | 5.90E-07 | Tpp2 | tripeptidyl peptidase II |
| ENSMUSG00000038481 | -1.45 | -2.734534997 | 2.09E-06 | Cdk19 | cyclin-dependent kinase 19 |
| ENSMUSG00000020464 | -1.45 | -2.733380861 | 1.05E-10 | Pnpt1 | polyribonucleotide nucleotidyltransferase 1 |
| ENSMUSG00000044626 | -1.45 | -2.732353893 | 8.73E-03 | Liph | lipase, member H |
| ENSMUSG00000027082 | -1.45 | -2.731613183 | 2.01E-05 | Tfpi | tissue factor pathway inhibitor |
| ENSMUSG00000082820 | -1.45 | -2.728605659 | 5.14E-04 | NA | NA |
| ENSMUSG00000034329 | -1.45 | -2.724875688 | 1.13E-05 | Brip1 | BRCA1 interacting protein C-terminal helicase 1 |
| ENSMUSG00000014932 | -1.45 | -2.724803805 | 1.25E-03 | Yes1 | Yamaguchi sarcoma viral (v-yes) oncogene homolog 1 |
| ENSMUSG00000049606 | -1.44 | -2.721260437 | 3.35E-05 | Zfp644 | zinc finger protein 644 |
| ENSMUSG00000003882 | -1.44 | -2.721148549 | 4.02E-08 | Il7r | interleukin 7 receptor |
| ENSMUSG00000022021 | -1.44 | -2.717760649 | 6.25E-07 | Diap3 | diaphanous homolog 3 (Drosophila) |
| ENSMUSG00000086741 | -1.44 | -2.716452987 | 1.17E-03 | Gm15816 | predicted gene 15816 |
| ENSMUSG00000028572 | -1.44 | -2.71500669 | 1.73E-04 | Hook1 | hook homolog 1 (Drosophila) |
| ENSMUSG00000020280 | -1.44 | -2.71478246 | 9.78E-08 | Pus10 | pseudouridylate synthase 10 |
| ENSMUSG00000036112 | -1.44 | -2.713479257 | 2.87E-07 | Metap2 | methionine aminopeptidase 2 |
| ENSMUSG00000055763 | -1.44 | -2.71345147 | 2.02E-03 | NA | NA |
| ENSMUSG00000053101 | -1.44 | -2.71122585 | 1.07E-04 | Gpr141 | G protein-coupled receptor 141 |
| ENSMUSG00000026614 | -1.44 | -2.709719185 | 3.13E-04 | Slc30a10 | solute carrier family 30, member 10 |
| ENSMUSG00000083338 | -1.44 | -2.709688007 | 5.11E-03 | NA | NA |
| ENSMUSG00000030313 | -1.44 | -2.709501128 | 1.93E-05 | Dennd5b | DENN/MADD domain containing 5B |
| ENSMUSG00000032410 | -1.43 | -2.702387803 | 4.39E-03 | Xrn1 | 5'-3' exoribonuclease 1 |
| ENSMUSG00000057335 | -1.43 | -2.702346595 | 1.93E-04 | Cep170 | centrosomal protein 170 |
| ENSMUSG00000060149 | -1.43 | -2.702013133 | 1.52E-03 | NA | NA |
| ENSMUSG00000034566 | -1.43 | -2.700606581 | 3.53E-03 | Atp5h | ATP synthase, H+ transporting, mitochondrial F0 complex, subunit D |
| ENSMUSG00000029920 | -1.43 | -2.700431368 | 2.87E-06 | Smarcad1 | SWI/SNF-related, matrix-associated actin-dependent regulator of chromatin, subfamily a, containing DEAD/H box 1 |
| ENSMUSG00000026399 | -1.43 | -2.700024882 | 3.62E-05 | Cd55 | CD55 antigen |
| ENSMUSG00000029787 | -1.43 | -2.690223396 | 2.14E-04 | Avl9 | AVL9 homolog (S. cerevisiase) |
| ENSMUSG00000041238 | -1.43 | -2.689612094 | 1.15E-06 | Rbbp8 | retinoblastoma binding protein 8 |
| ENSMUSG00000066800 | -1.43 | -2.68955942 | 1.76E-03 | Rnasel | ribonuclease L (2', 5'-oligoisoadenylate synthetase-dependent) |
| ENSMUSG00000057551 | -1.43 | -2.687161237 | 1.80E-05 | Zfp317 | zinc finger protein 317 |
| ENSMUSG00000033054 | -1.43 | -2.686990616 | 1.29E-08 | Npat | nuclear protein in the AT region |
| ENSMUSG00000020982 | -1.43 | -2.686234726 | 2.77E-06 | Nemf | nuclear export mediator factor |
| ENSMUSG00000037685 | -1.43 | -2.68548795 | 7.20E-06 | Atp8a1 | ATPase, aminophospholipid transporter (APLT), class I, type 8A, member 1 |
| ENSMUSG00000022533 | -1.42 | -2.682768965 | 2.28E-04 | Atp13a3 | ATPase type 13A3 |
| ENSMUSG00000029798 | -1.42 | -2.682019245 | 1.28E-12 | Herc6 | hect domain and RLD 6 |
| ENSMUSG00000036822 | -1.42 | -2.681159428 | 2.54E-05 | Topors | topoisomerase I binding, arginine/serine-rich |
| ENSMUSG00000066048 | -1.42 | -2.67737082 | 8.24E-04 | NA | NA |
| ENSMUSG00000034109 | -1.42 | -2.676449159 | 7.56E-05 | Golim4 | golgi integral membrane protein 4 |
| ENSMUSG00000034218 | -1.42 | -2.676004785 | 2.83E-05 | Atm | ataxia telangiectasia mutated homolog (human) |
| ENSMUSG00000035614 | -1.42 | -2.675811281 | 3.74E-04 | Fam179b | family with sequence similarity 179, member B |
| ENSMUSG00000028718 | -1.42 | -2.673650124 | 6.94E-06 | Stil | Scl/Tal1 interrupting locus |
| ENSMUSG00000028252 | -1.42 | -2.670747838 | 9.19E-05 | Ccnc | cyclin C |
| ENSMUSG00000003929 | -1.42 | -2.669793373 | 6.25E-06 | Zfp81 | zinc finger protein 81 |
| ENSMUSG00000050192 | -1.42 | -2.668026726 | 2.00E-05 | Eif5a2 | eukaryotic translation initiation factor 5A2 |
| ENSMUSG00000026028 | -1.41 | -2.665804598 | 7.83E-04 | Trak2 | trafficking protein, kinesin binding 2 |
| ENSMUSG00000021028 | -1.41 | -2.665443733 | 1.15E-03 | Mbip | MAP3K12 binding inhibitory protein 1 |
| ENSMUSG00000049878 | -1.41 | -2.66484909 | 2.21E-06 | Rlf | rearranged L-myc fusion sequence |
| ENSMUSG00000066880 | -1.41 | -2.663944323 | 7.20E-04 | Zfp617 | zinc finger protein 617 |
| ENSMUSG00000024056 | -1.41 | -2.661938395 | 1.30E-04 | Ndc80 | NDC80 homolog, kinetochore complex component (S. cerevisiae) |
| ENSMUSG00000071267 | -1.41 | -2.660511708 | 4.09E-04 | Zfp942 | zinc finger protein 942 |
| ENSMUSG00000000838 | -1.41 | -2.657158724 | 2.36E-06 | Fmr1 | fragile X mental retardation syndrome 1 |
| ENSMUSG00000006586 | -1.41 | -2.656981135 | 5.78E-03 | Runx1t1 | runt-related transcription factor 1; translocated to, 1 (cyclin D-related) |
| ENSMUSG00000020541 | -1.41 | -2.656298905 | 1.89E-08 | Tom1l1 | target of myb1-like 1 (chicken) |
| ENSMUSG00000062078 | -1.41 | -2.654778663 | 3.65E-04 | Qk | quaking |
| ENSMUSG00000024293 | -1.41 | -2.653586674 | 4.39E-05 | Esco1 | establishment of cohesion 1 homolog 1 (S. cerevisiae) |
| ENSMUSG00000035151 | -1.41 | -2.652178789 | 4.29E-06 | Elmod2 | ELMO/CED-12 domain containing 2 |
| ENSMUSG00000048874 | -1.41 | -2.652172988 | 4.56E-06 | Phf3 | PHD finger protein 3 |
| ENSMUSG00000014956 | -1.41 | -2.650351982 | 8.89E-12 | Ppp1cb | protein phosphatase 1, catalytic subunit, beta isoform |
| ENSMUSG00000024989 | -1.41 | -2.649044609 | 5.92E-07 | Cep55 | centrosomal protein 55 |
| ENSMUSG00000033166 | -1.40 | -2.646164248 | 1.77E-05 | Dis3 | DIS3 mitotic control homolog (S. cerevisiae) |
| ENSMUSG00000041328 | -1.40 | -2.641887888 | 2.12E-05 | Pcf11 | cleavage and polyadenylation factor subunit homolog (S. cerevisiae) |
| ENSMUSG00000040464 | -1.40 | -2.64117487 | 8.75E-04 | Gtpbp10 | GTP-binding protein 10 (putative) |
| ENSMUSG00000079991 | -1.40 | -2.640191604 | 7.32E-03 | NA | NA |
| ENSMUSG00000027823 | -1.40 | -2.637555218 | 3.46E-07 | Gmps | guanine monophosphate synthetase |
| ENSMUSG00000078139 | -1.40 | -2.637330663 | 1.16E-05 | NA | NA |
| ENSMUSG00000071543 | -1.40 | -2.633205826 | 7.71E-03 | NA | NA |
| ENSMUSG00000045932 | -1.40 | -2.63318364 | 8.99E-08 | Ifit2 | interferon-induced protein with tetratricopeptide repeats 2 |
| ENSMUSG00000026781 | -1.40 | -2.632983994 | 5.73E-05 | Acbd5 | acyl-Coenzyme A binding domain containing 5 |
| ENSMUSG00000055639 | -1.40 | -2.632792319 | 2.97E-03 | Dach1 | dachshund 1 (Drosophila) |
| ENSMUSG00000028693 | -1.40 | -2.632494283 | 4.73E-05 | Nasp | nuclear autoantigenic sperm protein (histone-binding) |
| ENSMUSG00000061665 | -1.40 | -2.630497282 | 2.93E-06 | Cd2ap | CD2-associated protein |
| ENSMUSG00000065281 | -1.40 | -2.629982338 | 5.59E-03 | NA | NA |
| ENSMUSG00000027287 | -1.39 | -2.628221807 | 1.26E-06 | Snap23 | synaptosomal-associated protein 23 |
| ENSMUSG00000021302 | -1.39 | -2.625481932 | 1.59E-05 | Ggps1 | geranylgeranyl diphosphate synthase 1 |
| ENSMUSG00000079340 | -1.39 | -2.624704158 | 2.86E-03 | NA | NA |
| ENSMUSG00000021112 | -1.39 | -2.623631323 | 1.09E-04 | Mpp5 | membrane protein, palmitoylated 5 (MAGUK p55 subfamily member 5) |
| ENSMUSG00000071796 | -1.39 | -2.623557711 | 5.39E-03 | 6820431F20Rik | cadherin 11 pseudogene |
| ENSMUSG00000017291 | -1.39 | -2.623379157 | 9.24E-06 | Taok1 | TAO kinase 1 |
| ENSMUSG00000060090 | -1.39 | -2.620934717 | 1.10E-08 | Rp2h | retinitis pigmentosa 2 homolog (human) |
| ENSMUSG00000031986 | -1.39 | -2.619110138 | 3.01E-03 | Sprtn | SprT-like N-terminal domain |
| ENSMUSG00000082194 | -1.39 | -2.616734789 | 4.42E-05 | NA | NA |
| ENSMUSG00000026434 | -1.39 | -2.61662728 | 4.32E-07 | Nucks1 | nuclear casein kinase and cyclin-dependent kinase substrate 1 |
| ENSMUSG00000021520 | -1.39 | -2.615382177 | 6.65E-04 | Uqcrb | ubiquinol-cytochrome c reductase binding protein |
| ENSMUSG00000051341 | -1.39 | -2.615048177 | 8.61E-05 | Zfp52 | zinc finger protein 52 |
| ENSMUSG00000021413 | -1.39 | -2.614305759 | 3.06E-05 | Prpf4b | PRP4 pre-mRNA processing factor 4 homolog B (yeast) |
| ENSMUSG00000033906 | -1.39 | -2.612634114 | 6.31E-05 | Zdhhc15 | zinc finger, DHHC domain containing 15 |
| ENSMUSG00000012483 | -1.38 | -2.610337982 | 4.60E-03 | Rpa3 | replication protein A3 |
| ENSMUSG00000040522 | -1.38 | -2.60898694 | 9.52E-06 | Tlr8 | toll-like receptor 8 |
| ENSMUSG00000082154 | -1.38 | -2.607476107 | 8.88E-03 | NA | NA |
| ENSMUSG00000051220 | -1.38 | -2.606772615 | 7.10E-06 | Ercc6l | excision repair cross-complementing rodent repair deficiency complementation group 6 like |
| ENSMUSG00000037544 | -1.38 | -2.604459508 | 8.87E-05 | Dlgap5 | discs, large (Drosophila) homolog-associated protein 5 |
| ENSMUSG00000037674 | -1.38 | -2.602770764 | 1.20E-04 | Rfx7 | regulatory factor X, 7 |
| ENSMUSG00000039187 | -1.38 | -2.601718606 | 2.68E-04 | Fanci | Fanconi anemia, complementation group I |
| ENSMUSG00000056342 | -1.38 | -2.601318733 | 2.98E-05 | Usp34 | ubiquitin specific peptidase 34 |
| ENSMUSG00000021693 | -1.38 | -2.600545891 | 1.77E-07 | Kif2a | kinesin family member 2A |
| ENSMUSG00000021945 | -1.38 | -2.598191796 | 1.67E-04 | Zmym2 | zinc finger, MYM-type 2 |
| ENSMUSG00000031333 | -1.38 | -2.597555299 | 2.83E-05 | Abcb7 | ATP-binding cassette, sub-family B (MDR/TAP), member 7 |
| ENSMUSG00000021754 | -1.38 | -2.596505877 | 2.16E-06 | Map3k1 | mitogen-activated protein kinase kinase kinase 1 |
| ENSMUSG00000035365 | -1.38 | -2.595683546 | 1.87E-05 | Parpbp | PARP1 binding protein |
| ENSMUSG00000022329 | -1.38 | -2.595668344 | 8.24E-06 | Stk3 | serine/threonine kinase 3 |
| ENSMUSG00000060044 | -1.38 | -2.595572231 | 4.95E-03 | Tmem26 | transmembrane protein 26 |
| ENSMUSG00000059742 | -1.37 | -2.592702769 | 3.58E-03 | Kcnh7 | potassium voltage-gated channel, subfamily H (eag-related), member 7 |
| ENSMUSG00000059897 | -1.37 | -2.592237157 | 5.51E-03 | Zfp930 | zinc finger protein 930 |
| ENSMUSG00000031314 | -1.37 | -2.590445993 | 5.77E-05 | Taf1 | TAF1 RNA polymerase II, TATA box binding protein (TBP)-associated factor |
| ENSMUSG00000038323 | -1.37 | -2.590069823 | 3.30E-03 | 1700066M21Rik | RIKEN cDNA 1700066M21 gene |
| ENSMUSG00000032712 | -1.37 | -2.589755846 | 9.37E-05 | 2810474O19Rik | RIKEN cDNA 2810474O19 gene |
| ENSMUSG00000046230 | -1.37 | -2.588674392 | 1.67E-06 | Vps13a | vacuolar protein sorting 13A (yeast) |
| ENSMUSG00000021709 | -1.37 | -2.588118977 | 8.81E-06 | Erbb2ip | Erbb2 interacting protein |
| ENSMUSG00000024054 | -1.37 | -2.587445717 | 7.56E-08 | Smchd1 | SMC hinge domain containing 1 |
| ENSMUSG00000039270 | -1.37 | -2.586337341 | 4.51E-04 | Megf9 | multiple EGF-like-domains 9 |
| ENSMUSG00000024533 | -1.37 | -2.585004259 | 1.61E-06 | Spire1 | spire homolog 1 (Drosophila) |
| ENSMUSG00000025262 | -1.37 | -2.584397387 | 8.49E-03 | Fam120c | family with sequence similarity 120, member C |
| ENSMUSG00000000804 | -1.37 | -2.583400933 | 3.94E-05 | Usp32 | ubiquitin specific peptidase 32 |
| ENSMUSG00000026219 | -1.37 | -2.579606071 | 6.71E-06 | Trip12 | thyroid hormone receptor interactor 12 |
| ENSMUSG00000027810 | -1.36 | -2.574590973 | 1.69E-05 | Eif2a | eukaryotic translation initiation factor 2A |
| ENSMUSG00000074903 | -1.36 | -2.572916154 | 1.31E-06 | NA | NA |
| ENSMUSG00000027706 | -1.36 | -2.572913221 | 5.77E-05 | Sec62 | SEC62 homolog (S. cerevisiae) |
| ENSMUSG00000027014 | -1.36 | -2.572577211 | 2.00E-04 | Cwc22 | CWC22 spliceosome-associated protein homolog (S. cerevisiae) |
| ENSMUSG00000031245 | -1.36 | -2.570180986 | 6.08E-04 | Hmgn5 | high-mobility group nucleosome binding domain 5 |
| ENSMUSG00000031112 | -1.36 | -2.569492293 | 1.33E-04 | 2610018G03Rik | RIKEN cDNA 2610018G03 gene |
| ENSMUSG00000026683 | -1.36 | -2.567440737 | 5.59E-05 | Nuf2 | NUF2, NDC80 kinetochore complex component, homolog (S. cerevisiae) |
| ENSMUSG00000067189 | -1.36 | -2.566207867 | 5.29E-03 | NA | NA |
| ENSMUSG00000053289 | -1.36 | -2.559352423 | 3.25E-06 | Ddx10 | DEAD (Asp-Glu-Ala-Asp) box polypeptide 10 |
| ENSMUSG00000017146 | -1.35 | -2.557042583 | 2.38E-04 | Brca1 | breast cancer 1 |
| ENSMUSG00000042460 | -1.35 | -2.551933898 | 3.57E-03 | C1galt1 | core 1 synthase, glycoprotein-N-acetylgalactosamine 3-beta-galactosyltransferase, 1 |
| ENSMUSG00000061607 | -1.35 | -2.5517521 | 2.72E-05 | Mdc1 | mediator of DNA damage checkpoint 1 |
| ENSMUSG00000020589 | -1.35 | -2.551712888 | 3.49E-04 | Fam49a | family with sequence similarity 49, member A |
| ENSMUSG00000024283 | -1.35 | -2.546631506 | 5.32E-05 | Wac | WW domain containing adaptor with coiled-coil |
| ENSMUSG00000030142 | -1.35 | -2.544107838 | 2.81E-04 | Clec4e | C-type lectin domain family 4, member e |
| ENSMUSG00000037958 | -1.35 | -2.54297789 | 3.29E-04 | Ccdc55 | coiled-coil domain containing 55 |
| ENSMUSG00000018379 | -1.35 | -2.542894922 | 9.56E-06 | NA | NA |
| ENSMUSG00000074746 | -1.35 | -2.541940624 | 8.87E-04 | Pdzd8 | PDZ domain containing 8 |
| ENSMUSG00000027692 | -1.35 | -2.541637393 | 6.86E-04 | Tnik | TRAF2 and NCK interacting kinase |
| ENSMUSG00000063108 | -1.34 | -2.539577818 | 2.35E-04 | Zfp26 | zinc finger protein 26 |
| ENSMUSG00000040446 | -1.34 | -2.539319691 | 1.53E-04 | Rprd1a | regulation of nuclear pre-mRNA domain containing 1A |
| ENSMUSG00000015342 | -1.34 | -2.537801348 | 8.77E-04 | Xk | Kell blood group precursor (McLeod phenotype) homolog |
| ENSMUSG00000054641 | -1.34 | -2.535254372 | 6.03E-08 | Mmrn1 | multimerin 1 |
| ENSMUSG00000037270 | -1.34 | -2.535239886 | 1.47E-04 | 4932438A13Rik | RIKEN cDNA 4932438A13 gene |
| ENSMUSG00000037416 | -1.34 | -2.532010106 | 1.52E-06 | Dmxl1 | Dmx-like 1 |
| ENSMUSG00000019841 | -1.34 | -2.5317633 | 8.93E-09 | Rev3l | REV3-like, catalytic subunit of DNA polymerase zeta RAD54 like (S. cerevisiae) |
| ENSMUSG00000070544 | -1.34 | -2.531385801 | 2.08E-09 | Top1 | topoisomerase (DNA) I |
| ENSMUSG00000029911 | -1.34 | -2.53038133 | 2.00E-03 | Ssbp1 | single-stranded DNA binding protein 1 |
| ENSMUSG00000022247 | -1.34 | -2.529286253 | 1.35E-03 | Brix1 | BRX1, biogenesis of ribosomes, homolog (S. cerevisiae) |
| ENSMUSG00000037608 | -1.34 | -2.527559142 | 1.58E-05 | Bclaf1 | BCL2-associated transcription factor 1 |
| ENSMUSG00000028035 | -1.34 | -2.52599931 | 1.77E-07 | Dnajb4 | DnaJ (Hsp40) homolog, subfamily B, member 4 |
| ENSMUSG00000021938 | -1.34 | -2.5240947 | 1.38E-04 | Pspc1 | paraspeckle protein 1 |
| ENSMUSG00000087313 | -1.33 | -2.522204127 | 2.27E-04 | NA | NA |
| ENSMUSG00000031786 | -1.33 | -2.521404007 | 6.88E-05 | Ccdc135 | coiled-coil domain containing 135 |
| ENSMUSG00000025939 | -1.33 | -2.520505901 | 2.38E-06 | Ube2w | ubiquitin-conjugating enzyme E2W (putative) |
| ENSMUSG00000042167 | -1.33 | -2.520349134 | 2.22E-04 | Papd4 | PAP associated domain containing 4 |
| ENSMUSG00000023892 | -1.33 | -2.517108845 | 1.59E-04 | Zfp51 | zinc finger protein 51 |
| ENSMUSG00000022119 | -1.33 | -2.517011311 | 2.38E-03 | Rbm26 | RNA binding motif protein 26 |
| ENSMUSG00000084756 | -1.33 | -2.516967696 | 2.52E-03 | NA | NA |
| ENSMUSG00000021676 | -1.33 | -2.51552513 | 8.12E-06 | Iqgap2 | IQ motif containing GTPase activating protein 2 |
| ENSMUSG00000030275 | -1.33 | -2.514485597 | 1.25E-03 | Etnk1 | ethanolamine kinase 1 |
| ENSMUSG00000022369 | -1.33 | -2.511310503 | 9.26E-08 | Mtbp | Mdm2, transformed 3T3 cell double minute p53 binding protein |
| ENSMUSG00000066613 | -1.33 | -2.511164634 | 2.21E-03 | Zfp932 | zinc finger protein 932 |
| ENSMUSG00000048118 | -1.33 | -2.510826905 | 1.86E-06 | Arid4a | AT rich interactive domain 4A (RBP1-like) |
| ENSMUSG00000085611 | -1.33 | -2.510587402 | 2.26E-03 | NA | NA |
| ENSMUSG00000086438 | -1.33 | -2.509869575 | 3.77E-03 | Asb17os | ankyrin repeat and SOCS box-containing 17, opposite strand |
| ENSMUSG00000020074 | -1.33 | -2.508154173 | 1.19E-05 | Ccar1 | cell division cycle and apoptosis regulator 1 |
| ENSMUSG00000027306 | -1.33 | -2.507535987 | 2.23E-05 | Nusap1 | nucleolar and spindle associated protein 1 |
| ENSMUSG00000075470 | -1.33 | -2.505930205 | 6.49E-03 | Alg10b | asparagine-linked glycosylation 10B (alpha-1,2-glucosyltransferase) |
| ENSMUSG00000051910 | -1.32 | -2.504468496 | 3.25E-05 | Sox6 | SRY (sex determining region Y)-box 6 |
| ENSMUSG00000056536 | -1.32 | -2.503229099 | 8.00E-04 | Pign | phosphatidylinositol glycan anchor biosynthesis, class N |
| ENSMUSG00000020594 | -1.32 | -2.498808158 | 1.33E-04 | Pum2 | pumilio 2 (Drosophila) |
| ENSMUSG00000022307 | -1.32 | -2.49783492 | 3.41E-06 | Oxr1 | oxidation resistance 1 |
| ENSMUSG00000021189 | -1.32 | -2.496730032 | 9.08E-06 | Atxn3 | ataxin 3 |
| ENSMUSG00000030759 | -1.32 | -2.494520874 | 1.26E-06 | Far1 | fatty acyl CoA reductase 1 |
| ENSMUSG00000002428 | -1.32 | -2.494476937 | 6.42E-05 | Hltf | helicase-like transcription factor |
| ENSMUSG00000036461 | -1.32 | -2.490469457 | 7.97E-06 | Elf1 | E74-like factor 1 |
| ENSMUSG00000059839 | -1.31 | -2.487103743 | 3.15E-03 | Zfp874b | zinc finger protein 874b |
| ENSMUSG00000036368 | -1.31 | -2.486440855 | 6.03E-03 | Rmdn2 | regulator of microtubule dynamics 2 |
| ENSMUSG00000056144 | -1.31 | -2.485819709 | 2.25E-04 | Trim34a | tripartite motif-containing 34A |
| ENSMUSG00000029655 | -1.31 | -2.485605195 | 1.98E-04 | N4bp2l2 | NEDD4 binding protein 2-like 2 |
| ENSMUSG00000070501 | -1.31 | -2.480786133 | 2.40E-03 | BC094916 | cDNA sequence BC094916 |
| ENSMUSG00000023940 | -1.31 | -2.478337752 | 1.15E-04 | Sgol1 | shugoshin-like 1 (S. pombe) |
| ENSMUSG00000027881 | -1.31 | -2.478002808 | 4.14E-05 | Prpf38b | PRP38 pre-mRNA processing factor 38 (yeast) domain containing B |
| ENSMUSG00000006423 | -1.31 | -2.476910397 | 4.80E-05 | C330007P06Rik | RIKEN cDNA C330007P06 gene |
| ENSMUSG00000028307 | -1.31 | -2.47683405 | 9.69E-03 | Aldob | aldolase B, fructose-bisphosphate |
| ENSMUSG00000048188 | -1.31 | -2.47541406 | 9.56E-06 | NA | NA |
| ENSMUSG00000075592 | -1.31 | -2.475321932 | 4.81E-04 | Nynrin | NYN domain and retroviral integrase containing |
| ENSMUSG00000037572 | -1.31 | -2.474725558 | 9.61E-07 | Wdhd1 | WD repeat and HMG-box DNA binding protein 1 |
| ENSMUSG00000025060 | -1.31 | -2.47367206 | 9.43E-07 | Slk | STE20-like kinase |
| ENSMUSG00000021510 | -1.31 | -2.473386148 | 3.13E-04 | A530054K11Rik | RIKEN cDNA A530054K11 gene |
| ENSMUSG00000022148 | -1.31 | -2.473066603 | 2.19E-05 | Fyb | FYN binding protein |
| ENSMUSG00000042851 | -1.31 | -2.47294386 | 1.14E-03 | Zc3h6 | zinc finger CCCH type containing 6 |
| ENSMUSG00000020397 | -1.31 | -2.472208182 | 1.45E-07 | Med7 | mediator complex subunit 7 |
| ENSMUSG00000035247 | -1.31 | -2.471242677 | 3.69E-05 | Hectd1 | HECT domain containing 1 |
| ENSMUSG00000049460 | -1.30 | -2.470348852 | 1.06E-03 | NA | NA |
| ENSMUSG00000022837 | -1.30 | -2.470271388 | 1.64E-03 | Iqcb1 | IQ calmodulin-binding motif containing 1 |
| ENSMUSG00000067017 | -1.30 | -2.470158842 | 2.71E-07 | NA | NA |
| ENSMUSG00000028995 | -1.30 | -2.469450827 | 2.29E-04 | Fam126a | family with sequence similarity 126, member A |
| ENSMUSG00000046753 | -1.30 | -2.46774344 | 7.99E-04 | Ccdc66 | coiled-coil domain containing 66 |
| ENSMUSG00000072980 | -1.30 | -2.466862366 | 5.06E-05 | Oip5 | Opa interacting protein 5 |
| ENSMUSG00000058748 | -1.30 | -2.466688208 | 5.31E-04 | Zfp958 | zinc finger protein 958 |
| ENSMUSG00000086869 | -1.30 | -2.46551752 | 1.61E-04 | NA | NA |
| ENSMUSG00000021929 | -1.30 | -2.457912661 | 1.43E-04 | Kpna3 | karyopherin (importin) alpha 3 |
| ENSMUSG00000002881 | -1.30 | -2.455984883 | 4.03E-06 | Nab1 | Ngfi-A binding protein 1 |
| ENSMUSG00000038725 | -1.30 | -2.45544278 | 8.89E-04 | Pkhd1l1 | polycystic kidney and hepatic disease 1-like 1 |
| ENSMUSG00000024542 | -1.30 | -2.455161292 | 1.16E-07 | Cep192 | centrosomal protein 192 |
| ENSMUSG00000047902 | -1.29 | -2.452115582 | 2.16E-04 | NA | NA |
| ENSMUSG00000039531 | -1.29 | -2.451901003 | 6.10E-04 | Zufsp | zinc finger with UFM1-specific peptidase domain |
| ENSMUSG00000027162 | -1.29 | -2.449538203 | 1.18E-05 | Lin7c | lin-7 homolog C (C. elegans) |
| ENSMUSG00000038822 | -1.29 | -2.44903088 | 1.92E-06 | Hace1 | HECT domain and ankyrin repeat containing, E3 ubiquitin protein ligase 1 |
| ENSMUSG00000035873 | -1.29 | -2.448813344 | 9.77E-05 | Pawr | PRKC, apoptosis, WT1, regulator |
| ENSMUSG00000012640 | -1.29 | -2.448505678 | 1.44E-04 | Zfp715 | zinc finger protein 715 |
| ENSMUSG00000040520 | -1.29 | -2.448229165 | 7.26E-06 | Manea | mannosidase, endo-alpha |
| ENSMUSG00000017418 | -1.29 | -2.447954284 | 3.67E-04 | Arl5b | ADP-ribosylation factor-like 5B |
| ENSMUSG00000021470 | -1.29 | -2.447674793 | 2.48E-06 | Ercc6l2 | excision repair cross-complementing rodent repair deficiency, complementation group 6 like 2 |
| ENSMUSG00000086651 | -1.29 | -2.446344459 | 8.73E-05 | NA | NA |
| ENSMUSG00000026004 | -1.29 | -2.446076613 | 4.76E-04 | Kansl1l | KAT8 regulatory NSL complex subunit 1-like |
| ENSMUSG00000083557 | -1.29 | -2.44484134 | 5.71E-03 | NA | NA |
| ENSMUSG00000057808 | -1.29 | -2.442842633 | 4.94E-05 | NA | NA |
| ENSMUSG00000075444 | -1.29 | -2.439876628 | 7.76E-04 | NA | NA |
| ENSMUSG00000029657 | -1.29 | -2.438159218 | 6.38E-03 | Hsph1 | heat shock 105kDa/110kDa protein 1 |
| ENSMUSG00000082274 | -1.29 | -2.437978455 | 7.19E-04 | NA | NA |
| ENSMUSG00000047141 | -1.29 | -2.437085014 | 1.93E-03 | Zfp654 | zinc finger protein 654 |
| ENSMUSG00000041134 | -1.28 | -2.436361417 | 2.87E-03 | Cyyr1 | cysteine and tyrosine-rich protein 1 |
| ENSMUSG00000052917 | -1.28 | -2.434800415 | 1.08E-05 | Senp7 | SUMO1/sentrin specific peptidase 7 |
| ENSMUSG00000045427 | -1.28 | -2.434614784 | 1.04E-07 | Hnrnph2 | heterogeneous nuclear ribonucleoprotein H2 |
| ENSMUSG00000068134 | -1.28 | -2.432182895 | 3.51E-05 | Zfp120 | zinc finger protein 120 |
| ENSMUSG00000034023 | -1.28 | -2.431489005 | 1.25E-05 | Fancd2 | Fanconi anemia, complementation group D2 |
| ENSMUSG00000034724 | -1.28 | -2.430125468 | 6.35E-09 | Cnot6l | CCR4-NOT transcription complex, subunit 6-like |
| ENSMUSG00000021952 | -1.28 | -2.429491863 | 3.99E-03 | Xpo4 | exportin 4 |
| ENSMUSG00000070729 | -1.28 | -2.427706249 | 7.56E-07 | NA | NA |
| ENSMUSG00000042208 | -1.28 | -2.427645438 | 6.45E-06 | 0610010F05Rik | RIKEN cDNA 0610010F05 gene |
| ENSMUSG00000089672 | -1.28 | -2.425554602 | 1.27E-03 | Gp49a | glycoprotein 49 A |
| ENSMUSG00000027201 | -1.28 | -2.424706571 | 8.78E-05 | Myef2 | myelin basic protein expression factor 2, repressor |
| ENSMUSG00000090100 | -1.28 | -2.42313525 | 3.92E-05 | Ttbk2 | tau tubulin kinase 2 |
| ENSMUSG00000044934 | -1.28 | -2.420066277 | 1.29E-06 | Zfp367 | zinc finger protein 367 |
| ENSMUSG00000020954 | -1.27 | -2.419836409 | 8.84E-04 | Strn3 | striatin, calmodulin binding protein 3 |
| ENSMUSG00000034252 | -1.27 | -2.419248825 | 2.47E-05 | Senp6 | SUMO/sentrin specific peptidase 6 |
| ENSMUSG00000035248 | -1.27 | -2.419205261 | 5.31E-05 | Zcchc6 | zinc finger, CCHC domain containing 6 |
| ENSMUSG00000029227 | -1.27 | -2.418733463 | 4.21E-05 | Fip1l1 | FIP1 like 1 (S. cerevisiae) |
| ENSMUSG00000028967 | -1.27 | -2.418529142 | 3.22E-05 | Errfi1 | ERBB receptor feedback inhibitor 1 |
| ENSMUSG00000026770 | -1.27 | -2.418454767 | 8.40E-03 | Il2ra | interleukin 2 receptor, alpha chain |
| ENSMUSG00000030249 | -1.27 | -2.41617386 | 3.27E-04 | Abcc9 | ATP-binding cassette, sub-family C (CFTR/MRP), member 9 |
| ENSMUSG00000035834 | -1.27 | -2.413951344 | 5.91E-06 | Polr3g | polymerase (RNA) III (DNA directed) polypeptide G |
| ENSMUSG00000023926 | -1.27 | -2.409235356 | 1.05E-03 | Rhag | Rhesus blood group-associated A glycoprotein |
| ENSMUSG00000043424 | -1.27 | -2.409101546 | 3.48E-05 | Eif3j2 | eukaryotic translation initiation factor 3, subunit J2 |
| ENSMUSG00000056552 | -1.27 | -2.40847088 | 2.41E-03 | NA | NA |
| ENSMUSG00000047554 | -1.27 | -2.406986968 | 3.71E-04 | Tmem41b | transmembrane protein 41B |
| ENSMUSG00000020641 | -1.27 | -2.404015512 | 1.15E-07 | Rsad2 | radical S-adenosyl methionine domain containing 2 |
| ENSMUSG00000024077 | -1.26 | -2.400350072 | 2.28E-05 | Strn | striatin, calmodulin binding protein |
| ENSMUSG00000022865 | -1.26 | -2.400323421 | 1.73E-04 | Cxadr | coxsackie virus and adenovirus receptor |
| ENSMUSG00000064138 | -1.26 | -2.399550027 | 1.34E-04 | Fam172a | family with sequence similarity 172, member A |
| ENSMUSG00000028248 | -1.26 | -2.397694155 | 2.50E-08 | Sfrs18 | serine/arginine-rich splicing factor 18 |
| ENSMUSG00000039968 | -1.26 | -2.396436714 | 1.93E-04 | Rsbn1l | round spermatid basic protein 1-like |
| ENSMUSG00000031601 | -1.26 | -2.396156711 | 5.94E-07 | Cnot7 | CCR4-NOT transcription complex, subunit 7 |
| ENSMUSG00000027132 | -1.26 | -2.394305057 | 9.05E-04 | Katnbl1 | katanin p80 subunit B like 1 |
| ENSMUSG00000037262 | -1.26 | -2.394009802 | 2.24E-06 | Kin | antigenic determinant of rec-A protein |
| ENSMUSG00000037001 | -1.26 | -2.393228769 | 1.43E-05 | Zfp39 | zinc finger protein 39 |
| ENSMUSG00000082189 | -1.26 | -2.393055325 | 9.62E-03 | NA | NA |
| ENSMUSG00000041498 | -1.26 | -2.391520577 | 2.42E-04 | Kif14 | kinesin family member 14 |
| ENSMUSG00000034028 | -1.26 | -2.390392056 | 6.10E-04 | Cd226 | CD226 antigen |
| ENSMUSG00000021981 | -1.26 | -2.389631919 | 1.13E-03 | Cab39l | calcium binding protein 39-like |
| ENSMUSG00000083367 | -1.26 | -2.389382745 | 1.56E-05 | NA | NA |
| ENSMUSG00000087528 | -1.26 | -2.388863059 | 1.71E-04 | NA | NA |
| ENSMUSG00000062190 | -1.26 | -2.387016664 | 5.91E-07 | Lancl2 | LanC (bacterial lantibiotic synthetase component C)-like 2 |
| ENSMUSG00000027959 | -1.25 | -2.386668081 | 1.76E-04 | Sass6 | spindle assembly 6 homolog (C. elegans) |
| ENSMUSG00000032854 | -1.25 | -2.386484837 | 2.22E-03 | Ugt8a | UDP galactosyltransferase 8A |
| ENSMUSG00000026708 | -1.25 | -2.386083344 | 1.84E-05 | Cenpl | centromere protein L |
| ENSMUSG00000020952 | -1.25 | -2.385420192 | 6.86E-05 | Scfd1 | Sec1 family domain containing 1 |
| ENSMUSG00000074909 | -1.25 | -2.384431161 | 6.75E-04 | Ranbp6 | RAN binding protein 6 |
| ENSMUSG00000026463 | -1.25 | -2.384139388 | 2.96E-04 | Atp2b4 | ATPase, Ca++ transporting, plasma membrane 4 |
| ENSMUSG00000035597 | -1.25 | -2.383277983 | 3.61E-05 | Prpf39 | PRP39 pre-mRNA processing factor 39 homolog (yeast) |
| ENSMUSG00000017831 | -1.25 | -2.381618561 | 6.53E-05 | Rab5a | RAB5A, member RAS oncogene family |
| ENSMUSG00000078122 | -1.25 | -2.381326011 | 1.50E-03 | F630028O10Rik | RIKEN cDNA F630028O10 gene |
| ENSMUSG00000021548 | -1.25 | -2.37848092 | 3.66E-04 | Ccnh | cyclin H |
| ENSMUSG00000029169 | -1.25 | -2.377110992 | 2.14E-08 | Dhx15 | DEAH (Asp-Glu-Ala-His) box polypeptide 15 |
| ENSMUSG00000020120 | -1.25 | -2.374589932 | 1.77E-07 | Plek | pleckstrin |
| ENSMUSG00000078453 | -1.25 | -2.374432567 | 3.75E-05 | NA | NA |
| ENSMUSG00000035623 | -1.25 | -2.371650409 | 4.34E-05 | Rsf1 | remodeling and spacing factor 1 |
| ENSMUSG00000082625 | -1.24 | -2.369111628 | 7.61E-03 | NA | NA |
| ENSMUSG00000039361 | -1.24 | -2.368379677 | 1.68E-08 | Picalm | phosphatidylinositol binding clathrin assembly protein |
| ENSMUSG00000026721 | -1.24 | -2.368124708 | 6.28E-07 | Rabgap1l | RAB GTPase activating protein 1-like |
| ENSMUSG00000079575 | -1.24 | -2.368065172 | 2.05E-04 | NA | NA |
| ENSMUSG00000029405 | -1.24 | -2.368001165 | 1.11E-05 | G3bp2 | GTPase activating protein (SH3 domain) binding protein 2 |
| ENSMUSG00000027787 | -1.24 | -2.367199649 | 5.13E-05 | Nmd3 | NMD3 homolog (S. cerevisiae) |
| ENSMUSG00000029780 | -1.24 | -2.364334006 | 1.46E-04 | Nt5c3 | 5'-nucleotidase, cytosolic III |
| ENSMUSG00000041997 | -1.24 | -2.363338247 | 6.82E-08 | Tlk1 | tousled-like kinase 1 |
| ENSMUSG00000021585 | -1.24 | -2.363093012 | 3.41E-07 | Cast | calpastatin |
| ENSMUSG00000022710 | -1.24 | -2.362273757 | 7.68E-07 | Usp7 | ubiquitin specific peptidase 7 |
| ENSMUSG00000022100 | -1.24 | -2.361172252 | 1.75E-04 | Xpo7 | exportin 7 |
| ENSMUSG00000029246 | -1.24 | -2.360536264 | 8.57E-10 | Ppat | phosphoribosyl pyrophosphate amidotransferase |
| ENSMUSG00000049103 | -1.24 | -2.359457013 | 2.15E-04 | Ccr2 | chemokine (C-C motif) receptor 2 |
| ENSMUSG00000022422 | -1.24 | -2.358779461 | 3.47E-04 | Dscc1 | defective in sister chromatid cohesion 1 homolog (S. cerevisiae) |
| ENSMUSG00000030180 | -1.24 | -2.358579166 | 1.34E-04 | Kdm5a | lysine (K)-specific demethylase 5A |
| ENSMUSG00000063895 | -1.24 | -2.358109226 | 1.67E-05 | Nupl1 | nucleoporin like 1 |
| ENSMUSG00000025437 | -1.24 | -2.357819502 | 7.05E-07 | Usp33 | ubiquitin specific peptidase 33 |
| ENSMUSG00000019996 | -1.24 | -2.357127953 | 4.14E-05 | Map7 | microtubule-associated protein 7 |
| ENSMUSG00000041225 | -1.24 | -2.356901765 | 5.86E-05 | Arhgap12 | Rho GTPase activating protein 12 |
| ENSMUSG00000069910 | -1.24 | -2.356645546 | 1.35E-05 | Spdl1 | spindle apparatus coiled-coil protein 1 |
| ENSMUSG00000024290 | -1.24 | -2.35595477 | 1.08E-05 | Rock1 | Rho-associated coiled-coil containing protein kinase 1 |
| ENSMUSG00000074656 | -1.24 | -2.355716747 | 1.22E-05 | Eif2s2 | eukaryotic translation initiation factor 2, subunit 2 (beta) |
| ENSMUSG00000041846 | -1.24 | -2.355661325 | 1.83E-06 | Smek1 | SMEK homolog 1, suppressor of mek1 (Dictyostelium) |
| ENSMUSG00000039585 | -1.23 | -2.353030965 | 2.49E-06 | Myo9a | myosin IXa |
| ENSMUSG00000027843 | -1.23 | -2.352876644 | 4.08E-04 | Ptpn22 | protein tyrosine phosphatase, non-receptor type 22 (lymphoid) |
| ENSMUSG00000041935 | -1.23 | -2.352668738 | 5.26E-03 | AW549877 | expressed sequence AW549877 |
| ENSMUSG00000022899 | -1.23 | -2.347791892 | 2.64E-03 | Slc15a2 | solute carrier family 15 (H+/peptide transporter), member 2 |
| ENSMUSG00000044229 | -1.23 | -2.345765458 | 6.86E-05 | Nxpe4 | neurexophilin and PC-esterase domain family, member 4 |
| ENSMUSG00000025757 | -1.23 | -2.345636035 | 2.54E-05 | Hspa4l | heat shock protein 4 like |
| ENSMUSG00000032409 | -1.23 | -2.345243676 | 2.47E-06 | Atr | ataxia telangiectasia and Rad3 related |
| ENSMUSG00000000078 | -1.23 | -2.344039361 | 2.93E-06 | Klf6 | Kruppel-like factor 6 |
| ENSMUSG00000022747 | -1.23 | -2.343637736 | 1.45E-04 | St3gal6 | ST3 beta-galactoside alpha-2,3-sialyltransferase 6 |
| ENSMUSG00000057329 | -1.23 | -2.343482556 | 2.91E-05 | Bcl2 | B cell leukemia/lymphoma 2 |
| ENSMUSG00000031309 | -1.23 | -2.343441866 | 5.71E-06 | Rps6ka3 | ribosomal protein S6 kinase polypeptide 3 |
| ENSMUSG00000057894 | -1.23 | -2.34315935 | 9.65E-04 | Zfp329 | zinc finger protein 329 |
| ENSMUSG00000028518 | -1.23 | -2.342661468 | 8.82E-03 | Prkaa2 | protein kinase, AMP-activated, alpha 2 catalytic subunit |
| ENSMUSG00000074794 | -1.23 | -2.342655497 | 2.66E-05 | Arrdc3 | arrestin domain containing 3 |
| ENSMUSG00000027285 | -1.23 | -2.34162776 | 6.20E-06 | Haus2 | HAUS augmin-like complex, subunit 2 |
| ENSMUSG00000015314 | -1.23 | -2.341563549 | 1.31E-03 | Slamf6 | SLAM family member 6 |
| ENSMUSG00000021908 | -1.23 | -2.341207738 | 9.86E-06 | NA | NA |
| ENSMUSG00000024350 | -1.23 | -2.340305062 | 1.22E-03 | Dnajc18 | DnaJ (Hsp40) homolog, subfamily C, member 18 |
| ENSMUSG00000074460 | -1.23 | -2.338808215 | 1.97E-04 | NA | NA |
| ENSMUSG00000083545 | -1.22 | -2.336719215 | 4.27E-03 | NA | NA |
| ENSMUSG00000028552 | -1.22 | -2.336005841 | 1.32E-07 | Eps15 | epidermal growth factor receptor pathway substrate 15 |
| ENSMUSG00000020914 | -1.22 | -2.335626009 | 9.15E-06 | Top2a | topoisomerase (DNA) II alpha |
| ENSMUSG00000033417 | -1.22 | -2.335239282 | 3.87E-04 | Cacul1 | CDK2 associated, cullin domain 1 |
| ENSMUSG00000037921 | -1.22 | -2.334868881 | 4.32E-04 | Ddx60 | DEAD (Asp-Glu-Ala-Asp) box polypeptide 60 |
| ENSMUSG00000025758 | -1.22 | -2.333424981 | 7.57E-05 | Plk4 | polo-like kinase 4 |
| ENSMUSG00000027184 | -1.22 | -2.332914492 | 1.32E-07 | Caprin1 | cell cycle associated protein 1 |
| ENSMUSG00000044468 | -1.22 | -2.332832217 | 3.28E-04 | Fam46c | family with sequence similarity 46, member C |
| ENSMUSG00000050697 | -1.22 | -2.332402907 | 5.06E-05 | Prkaa1 | protein kinase, AMP-activated, alpha 1 catalytic subunit |
| ENSMUSG00000015882 | -1.22 | -2.331317748 | 1.90E-04 | Lcorl | ligand dependent nuclear receptor corepressor-like |
| ENSMUSG00000039219 | -1.22 | -2.33083362 | 8.47E-06 | Arid4b | AT rich interactive domain 4B (RBP1-like) |
| ENSMUSG00000021965 | -1.22 | -2.328996289 | 1.87E-05 | Ska3 | spindle and kinetochore associated complex subunit 3 |
| ENSMUSG00000020368 | -1.22 | -2.328812634 | 1.10E-07 | Canx | calnexin |
| ENSMUSG00000067430 | -1.22 | -2.327872202 | 2.55E-03 | Zfp763 | zinc finger protein 763 |
| ENSMUSG00000031381 | -1.22 | -2.327102473 | 2.06E-06 | Piga | phosphatidylinositol glycan anchor biosynthesis, class A |
| ENSMUSG00000022026 | -1.22 | -2.326790845 | 5.85E-04 | Olfm4 | olfactomedin 4 |
| ENSMUSG00000036223 | -1.22 | -2.32648048 | 9.15E-04 | Ska1 | spindle and kinetochore associated complex subunit 1 |
| ENSMUSG00000074785 | -1.22 | -2.32604684 | 2.73E-03 | Plxnc1 | plexin C1 |
| ENSMUSG00000027399 | -1.22 | -2.325892088 | 1.21E-03 | Il1a | interleukin 1 alpha |
| ENSMUSG00000026657 | -1.22 | -2.325843171 | 1.22E-04 | Frmd4a | FERM domain containing 4A |
| ENSMUSG00000024511 | -1.22 | -2.324571073 | 1.92E-06 | Rab27b | RAB27b, member RAS oncogene family |
| ENSMUSG00000026039 | -1.22 | -2.324331416 | 9.91E-05 | Sgol2 | shugoshin-like 2 (S. pombe) |
| ENSMUSG00000081865 | -1.22 | -2.324200094 | 6.33E-05 | NA | NA |
| ENSMUSG00000028218 | -1.22 | -2.323922939 | 1.46E-03 | Fam92a | family with sequence similarity 92, member A |
| ENSMUSG00000035898 | -1.22 | -2.323917015 | 4.56E-03 | Uba6 | ubiquitin-like modifier activating enzyme 6 |
| ENSMUSG00000050565 | -1.22 | -2.32353879 | 5.13E-07 | Tor1aip2 | torsin A interacting protein 2 |
| ENSMUSG00000063663 | -1.22 | -2.322625958 | 1.26E-03 | Brwd3 | bromodomain and WD repeat domain containing 3 |
| ENSMUSG00000020124 | -1.21 | -2.321144441 | 2.47E-05 | Usp15 | ubiquitin specific peptidase 15 |
| ENSMUSG00000037876 | -1.21 | -2.320859484 | 2.20E-04 | Jmjd1c | jumonji domain containing 1C |
| ENSMUSG00000063052 | -1.21 | -2.320770727 | 2.28E-05 | Lrrc40 | leucine rich repeat containing 40 |
| ENSMUSG00000058729 | -1.21 | -2.320693423 | 2.14E-08 | Lin9 | lin-9 homolog (C. elegans) |
| ENSMUSG00000006740 | -1.21 | -2.31850075 | 3.46E-03 | Kif5b | kinesin family member 5B |
| ENSMUSG00000046179 | -1.21 | -2.317157823 | 2.26E-04 | E2f8 | E2F transcription factor 8 |
| ENSMUSG00000032915 | -1.21 | -2.316884559 | 1.54E-03 | Emr4 | EGF-like module containing, mucin-like, hormone receptor-like sequence 4 |
| ENSMUSG00000037313 | -1.21 | -2.316445969 | 1.47E-04 | Tacc3 | transforming, acidic coiled-coil containing protein 3 |
| ENSMUSG00000041147 | -1.21 | -2.315636543 | 8.19E-05 | Brca2 | breast cancer 2 |
| ENSMUSG00000030322 | -1.21 | -2.313941874 | 2.69E-04 | Mbd4 | methyl-CpG binding domain protein 4 |
| ENSMUSG00000036499 | -1.21 | -2.313339387 | 3.65E-04 | Eea1 | early endosome antigen 1 |
| ENSMUSG00000051839 | -1.21 | -2.313272416 | 1.51E-03 | Gypa | glycophorin A |
| ENSMUSG00000037124 | -1.21 | -2.311722251 | 7.14E-03 | Trim58 | tripartite motif-containing 58 |
| ENSMUSG00000038047 | -1.21 | -2.310445881 | 2.47E-05 | Haus6 | HAUS augmin-like complex, subunit 6 |
| ENSMUSG00000064368 | -1.21 | -2.30998875 | 5.08E-03 | ND6 | NADH dehydrogenase subunit 6 |
| ENSMUSG00000040022 | -1.21 | -2.307528145 | 5.89E-04 | Rab11fip2 | RAB11 family interacting protein 2 (class I) |
| ENSMUSG00000034163 | -1.21 | -2.306731599 | 3.44E-04 | Zfc3h1 | zinc finger, C3H1-type containing |
| ENSMUSG00000020653 | -1.21 | -2.306610058 | 1.47E-04 | Klf11 | Kruppel-like factor 11 |
| ENSMUSG00000021270 | -1.21 | -2.306528316 | 9.00E-04 | Hsp90aa1 | heat shock protein 90, alpha (cytosolic), class A member 1 |
| ENSMUSG00000041671 | -1.20 | -2.304750434 | 4.96E-03 | Pyroxd1 | pyridine nucleotide-disulphide oxidoreductase domain 1 |
| ENSMUSG00000027508 | -1.20 | -2.303409973 | 8.12E-05 | Pag1 | phosphoprotein associated with glycosphingolipid microdomains 1 |
| ENSMUSG00000057858 | -1.20 | -2.302506741 | 7.50E-04 | D19Ertd737e | DNA segment, Chr 19, ERATO Doi 737, expressed |
| ENSMUSG00000035161 | -1.20 | -2.302360102 | 4.09E-04 | Ints6 | integrator complex subunit 6 |
| ENSMUSG00000041219 | -1.20 | -2.302212224 | 4.01E-05 | Arhgap11a | Rho GTPase activating protein 11A |
| ENSMUSG00000036390 | -1.20 | -2.301683141 | 1.16E-04 | Gadd45a | growth arrest and DNA-damage-inducible 45 alpha |
| ENSMUSG00000036377 | -1.20 | -2.300975887 | 2.36E-03 | C530008M17Rik | RIKEN cDNA C530008M17 gene |
| ENSMUSG00000032219 | -1.20 | -2.300650538 | 2.64E-04 | NA | NA |
| ENSMUSG00000078301 | -1.20 | -2.29993153 | 1.59E-03 | NA | NA |
| ENSMUSG00000002297 | -1.20 | -2.297469956 | 1.21E-04 | Dbf4 | DBF4 homolog (S. cerevisiae) |
| ENSMUSG00000036371 | -1.20 | -2.296150899 | 2.57E-04 | Serbp1 | serpine1 mRNA binding protein 1 |
| ENSMUSG00000028578 | -1.20 | -2.295464717 | 3.14E-03 | Caap1 | caspase activity and apoptosis inhibitor 1 |
| ENSMUSG00000033184 | -1.20 | -2.294720415 | 3.01E-05 | Tmed7 | transmembrane emp24 protein transport domain containing 7 |
| ENSMUSG00000067367 | -1.20 | -2.294178215 | 2.91E-04 | Lyar | Ly1 antibody reactive clone |
| ENSMUSG00000063894 | -1.20 | -2.293763818 | 2.62E-05 | Zkscan8 | zinc finger with KRAB and SCAN domains 8 |
| ENSMUSG00000056260 | -1.20 | -2.293328609 | 2.43E-04 | Lrif1 | ligand dependent nuclear receptor interacting factor 1 |
| ENSMUSG00000090124 | -1.20 | -2.291348878 | 2.26E-03 | Ugt1a7c | UDP glucuronosyltransferase 1 family, polypeptide A7C |
| ENSMUSG00000039763 | -1.19 | -2.288792847 | 9.02E-03 | Dnajc28 | DnaJ (Hsp40) homolog, subfamily C, member 28 |
| ENSMUSG00000058799 | -1.19 | -2.287463249 | 2.91E-05 | Nap1l1 | nucleosome assembly protein 1-like 1 |
| ENSMUSG00000034906 | -1.19 | -2.287426677 | 4.09E-05 | Ncaph | non-SMC condensin I complex, subunit H |
| ENSMUSG00000031585 | -1.19 | -2.28620052 | 2.64E-03 | Gtf2e2 | general transcription factor II E, polypeptide 2 (beta subunit) |
| ENSMUSG00000047648 | -1.19 | -2.285864821 | 5.06E-05 | Fbxo30 | F-box protein 30 |
| ENSMUSG00000021668 | -1.19 | -2.285288099 | 1.08E-03 | Polk | polymerase (DNA directed), kappa |
| ENSMUSG00000066415 | -1.19 | -2.285223006 | 2.48E-06 | Msl2 | male-specific lethal 2 homolog (Drosophila) |
| ENSMUSG00000030619 | -1.19 | -2.284112189 | 4.98E-05 | Eed | embryonic ectoderm development |
| ENSMUSG00000029729 | -1.19 | -2.283145499 | 1.12E-05 | Zkscan1 | zinc finger with KRAB and SCAN domains 1 |
| ENSMUSG00000040818 | -1.19 | -2.283144149 | 1.77E-06 | Dennd6a | DENN/MADD domain containing 6A |
| ENSMUSG00000024068 | -1.19 | -2.283011183 | 2.39E-04 | Spast | spastin |
| ENSMUSG00000089942 | -1.19 | -2.282656216 | 3.01E-03 | Pira2 | paired-Ig-like receptor A2 |
| ENSMUSG00000022512 | -1.19 | -2.282386932 | 9.99E-04 | Cldn1 | claudin 1 |
| ENSMUSG00000040855 | -1.19 | -2.282178159 | 4.00E-03 | Reps2 | RALBP1 associated Eps domain containing protein 2 |
| ENSMUSG00000005371 | -1.19 | -2.2818333 | 1.00E-04 | Fbxo11 | F-box protein 11 |
| ENSMUSG00000033931 | -1.19 | -2.281202668 | 1.25E-05 | Rbm34 | RNA binding motif protein 34 |
| ENSMUSG00000031922 | -1.19 | -2.279139635 | 2.16E-05 | Cep57 | centrosomal protein 57 |
| ENSMUSG00000022378 | -1.19 | -2.278049263 | 5.74E-04 | Fam49b | family with sequence similarity 49, member B |
| ENSMUSG00000026980 | -1.19 | -2.275472463 | 8.80E-04 | Ly75 | lymphocyte antigen 75 |
| ENSMUSG00000031527 | -1.19 | -2.274583044 | 1.96E-08 | Eri1 | exoribonuclease 1 |
| ENSMUSG00000039166 | -1.18 | -2.272070239 | 7.81E-05 | Akap7 | A kinase (PRKA) anchor protein 7 |
| ENSMUSG00000026395 | -1.18 | -2.271183105 | 1.35E-07 | Ptprc | protein tyrosine phosphatase, receptor type, C |
| ENSMUSG00000060657 | -1.18 | -2.269984289 | 1.46E-03 | Marf1 | meiosis arrest female 1 |
| ENSMUSG00000021379 | -1.18 | -2.268813878 | 7.84E-03 | Id4 | inhibitor of DNA binding 4 |
| ENSMUSG00000046138 | -1.18 | -2.268609311 | 6.06E-05 | 9930021J03Rik | RIKEN cDNA 9930021J03 gene |
| ENSMUSG00000039480 | -1.18 | -2.268424143 | 7.16E-08 | Nt5dc1 | 5'-nucleotidase domain containing 1 |
| ENSMUSG00000022698 | -1.18 | -2.26837158 | 1.92E-06 | Naa50 | N(alpha)-acetyltransferase 50, NatE catalytic subunit |
| ENSMUSG00000040123 | -1.18 | -2.267759901 | 5.32E-05 | Zmym5 | zinc finger, MYM-type 5 |
| ENSMUSG00000030138 | -1.18 | -2.267062313 | 3.04E-05 | Bms1 | BMS1 homolog, ribosome assembly protein (yeast) |
| ENSMUSG00000018341 | -1.18 | -2.266759794 | 3.43E-04 | Il12rb2 | interleukin 12 receptor, beta 2 |
| ENSMUSG00000023284 | -1.18 | -2.263572783 | 1.67E-03 | Zfp605 | zinc finger protein 605 |
| ENSMUSG00000026491 | -1.18 | -2.263495742 | 1.52E-05 | Ahctf1 | AT hook containing transcription factor 1 |
| ENSMUSG00000050157 | -1.18 | -2.262155841 | 1.77E-03 | NA | NA |
| ENSMUSG00000026069 | -1.18 | -2.260878242 | 9.31E-04 | Il1rl1 | interleukin 1 receptor-like 1 |
| ENSMUSG00000012443 | -1.18 | -2.258431676 | 2.35E-04 | Kif11 | kinesin family member 11 |
| ENSMUSG00000001376 | -1.17 | -2.257912499 | 1.22E-03 | Ccdc132 | coiled-coil domain containing 132 |
| ENSMUSG00000021546 | -1.17 | -2.2572338 | 3.13E-04 | Hnrnpk | heterogeneous nuclear ribonucleoprotein K |
| ENSMUSG00000021831 | -1.17 | -2.256692624 | 2.00E-05 | Ero1l | ERO1-like (S. cerevisiae) |
| ENSMUSG00000028228 | -1.17 | -2.256275632 | 1.12E-04 | Cpne3 | copine III |
| ENSMUSG00000029823 | -1.17 | -2.255555833 | 1.22E-05 | Luc7l2 | LUC7-like 2 (S. cerevisiae) |
| ENSMUSG00000055228 | -1.17 | -2.255040638 | 7.14E-03 | NA | NA |
| ENSMUSG00000004642 | -1.17 | -2.254999763 | 3.07E-06 | Slbp | stem-loop binding protein |
| ENSMUSG00000061897 | -1.17 | -2.253658815 | 2.02E-03 | NA | NA |
| ENSMUSG00000024174 | -1.17 | -2.250448708 | 4.05E-05 | NA | NA |
| ENSMUSG00000022855 | -1.17 | -2.249597559 | 1.22E-05 | Senp2 | SUMO/sentrin specific peptidase 2 |
| ENSMUSG00000032555 | -1.17 | -2.248003578 | 2.82E-05 | Topbp1 | topoisomerase (DNA) II binding protein 1 |
| ENSMUSG00000026987 | -1.17 | -2.2472184 | 1.78E-04 | Baz2b | bromodomain adjacent to zinc finger domain, 2B |
| ENSMUSG00000083027 | -1.17 | -2.24423222 | 5.75E-03 | NA | NA |
| ENSMUSG00000028187 | -1.17 | -2.244160379 | 9.20E-05 | Rpf1 | ribosome production factor 1 homolog (S. cerevisiae) |
| ENSMUSG00000022797 | -1.17 | -2.24388784 | 1.02E-03 | Tfrc | transferrin receptor |
| ENSMUSG00000040569 | -1.17 | -2.243524945 | 6.66E-03 | Slc26a7 | solute carrier family 26, member 7 |
| ENSMUSG00000022659 | -1.17 | -2.243140261 | 2.75E-04 | Gcsam | germinal center associated, signaling and motility |
| ENSMUSG00000032745 | -1.17 | -2.242969634 | 9.19E-05 | Gpbp1 | GC-rich promoter binding protein 1 |
| ENSMUSG00000041781 | -1.16 | -2.241544745 | 7.94E-05 | Cpsf2 | cleavage and polyadenylation specific factor 2 |
| ENSMUSG00000044060 | -1.16 | -2.240477686 | 1.25E-03 | A830010M20Rik | RIKEN cDNA A830010M20 gene |
| ENSMUSG00000030779 | -1.16 | -2.239767548 | 4.39E-04 | Rbbp6 | retinoblastoma binding protein 6 |
| ENSMUSG00000028381 | -1.16 | -2.238991319 | 1.85E-06 | Ugcg | UDP-glucose ceramide glucosyltransferase |
| ENSMUSG00000072501 | -1.16 | -2.236600657 | 7.14E-04 | Phf20l1 | PHD finger protein 20-like 1 |
| ENSMUSG00000047126 | -1.16 | -2.236488594 | 1.06E-07 | Cltc | clathrin, heavy polypeptide (Hc) |
| ENSMUSG00000035726 | -1.16 | -2.235617988 | 1.35E-05 | Supt16 | suppressor of Ty 16 |
| ENSMUSG00000029208 | -1.16 | -2.235371609 | 2.67E-04 | Guf1 | GUF1 GTPase homolog (S. cerevisiae) |
| ENSMUSG00000079139 | -1.16 | -2.235175679 | 1.72E-04 | NA | NA |
| ENSMUSG00000037458 | -1.16 | -2.234596241 | 5.46E-05 | Azin1 | antizyme inhibitor 1 |
| ENSMUSG00000022361 | -1.16 | -2.232240402 | 5.37E-07 | Zhx1 | zinc fingers and homeoboxes 1 |
| ENSMUSG00000002944 | -1.16 | -2.232066609 | 3.21E-05 | Cd36 | CD36 antigen |
| ENSMUSG00000000276 | -1.16 | -2.231505565 | 8.21E-04 | Dgke | diacylglycerol kinase, epsilon |
| ENSMUSG00000024287 | -1.16 | -2.230722999 | 7.70E-03 | Thoc1 | THO complex 1 |
| ENSMUSG00000022336 | -1.16 | -2.229997535 | 4.09E-03 | Eif3e | eukaryotic translation initiation factor 3, subunit E |
| ENSMUSG00000021118 | -1.16 | -2.229884584 | 7.42E-05 | Plek2 | pleckstrin 2 |
| ENSMUSG00000082101 | -1.16 | -2.229571866 | 3.42E-03 | Slfn14 | schlafen 14 |
| ENSMUSG00000060377 | -1.16 | -2.22943805 | 5.58E-03 | NA | NA |
| ENSMUSG00000020063 | -1.16 | -2.2292189 | 1.64E-05 | Sirt1 | sirtuin 1 |
| ENSMUSG00000037197 | -1.16 | -2.229082226 | 2.42E-04 | Rbm17 | RNA binding motif protein 17 |
| ENSMUSG00000043241 | -1.16 | -2.228431543 | 5.20E-05 | Upf2 | UPF2 regulator of nonsense transcripts homolog (yeast) |
| ENSMUSG00000024474 | -1.16 | -2.227880487 | 1.12E-04 | Ik | IK cytokine |
| ENSMUSG00000033487 | -1.15 | -2.224165507 | 6.66E-04 | Fndc3a | fibronectin type III domain containing 3A |
| ENSMUSG00000034206 | -1.15 | -2.224020515 | 5.95E-05 | Polq | polymerase (DNA directed), theta |
| ENSMUSG00000027242 | -1.15 | -2.223750325 | 1.60E-04 | Wdr76 | WD repeat domain 76 |
| ENSMUSG00000029840 | -1.15 | -2.222541517 | 7.72E-11 | Mtpn | myotrophin |
| ENSMUSG00000040044 | -1.15 | -2.222323621 | 5.29E-07 | Orc3 | origin recognition complex, subunit 3 |
| ENSMUSG00000035762 | -1.15 | -2.221985223 | 2.13E-04 | Tmem161b | transmembrane protein 161B |
| ENSMUSG00000029267 | -1.15 | -2.221939622 | 8.78E-05 | Mtf2 | metal response element binding transcription factor 2 |
| ENSMUSG00000059288 | -1.15 | -2.221582983 | 3.33E-07 | Cdyl | chromodomain protein, Y chromosome-like |
| ENSMUSG00000070942 | -1.15 | -2.220896423 | 5.76E-04 | Il1rl2 | interleukin 1 receptor-like 2 |
| ENSMUSG00000073639 | -1.15 | -2.220748523 | 1.04E-05 | Rab18 | RAB18, member RAS oncogene family |
| ENSMUSG00000035021 | -1.15 | -2.21939586 | 1.03E-03 | Baz1a | bromodomain adjacent to zinc finger domain 1A |
| ENSMUSG00000032261 | -1.15 | -2.219302248 | 1.31E-04 | Sh3bgrl2 | SH3 domain binding glutamic acid-rich protein like 2 |
| ENSMUSG00000071226 | -1.15 | -2.218219194 | 1.55E-03 | Cecr2 | cat eye syndrome chromosome region, candidate 2 |
| ENSMUSG00000020694 | -1.15 | -2.218175347 | 2.63E-06 | Tlk2 | tousled-like kinase 2 (Arabidopsis) |
| ENSMUSG00000081152 | -1.15 | -2.218172874 | 1.90E-04 | NA | NA |
| ENSMUSG00000068798 | -1.15 | -2.21759421 | 2.02E-03 | Rap1a | RAS-related protein-1a |
| ENSMUSG00000014361 | -1.15 | -2.216905203 | 4.37E-04 | Mertk | c-mer proto-oncogene tyrosine kinase |
| ENSMUSG00000025266 | -1.15 | -2.216666651 | 1.41E-07 | Gnl3l | guanine nucleotide binding protein-like 3 (nucleolar)-like |
| ENSMUSG00000090035 | -1.15 | -2.213775163 | 1.51E-05 | Galnt4 | UDP-N-acetyl-alpha-D-galactosamine:polypeptide N-acetylgalactosaminyltransferase 4 |
| ENSMUSG00000036053 | -1.15 | -2.211651019 | 8.52E-04 | Fmnl2 | formin-like 2 |
| ENSMUSG00000024081 | -1.14 | -2.211185612 | 5.91E-07 | Cebpz | CCAAT/enhancer binding protein zeta |
| ENSMUSG00000053774 | -1.14 | -2.208167175 | 2.61E-03 | Ubxn7 | UBX domain protein 7 |
| ENSMUSG00000045210 | -1.14 | -2.207974305 | 8.66E-04 | Vcpip1 | valosin containing protein (p97)/p47 complex interacting protein 1 |
| ENSMUSG00000004698 | -1.14 | -2.207842884 | 5.53E-03 | Hdac9 | histone deacetylase 9 |
| ENSMUSG00000020490 | -1.14 | -2.207623458 | 3.37E-03 | Btnl10 | butyrophilin-like 10 |
| ENSMUSG00000060373 | -1.14 | -2.20641122 | 1.02E-03 | Hnrnpc | heterogeneous nuclear ribonucleoprotein C |
| ENSMUSG00000036438 | -1.14 | -2.206377689 | 7.49E-03 | NA | NA |
| ENSMUSG00000027763 | -1.14 | -2.205782674 | 5.47E-05 | Mbnl1 | muscleblind-like 1 (Drosophila) |
| ENSMUSG00000014905 | -1.14 | -2.205014467 | 2.09E-03 | Dnajb9 | DnaJ (Hsp40) homolog, subfamily B, member 9 |
| ENSMUSG00000073067 | -1.14 | -2.204738159 | 7.46E-03 | 9130019P16Rik | RIKEN cDNA 9130019P16 gene |
| ENSMUSG00000030729 | -1.14 | -2.204459012 | 5.73E-08 | Pgm2l1 | phosphoglucomutase 2-like 1 |
| ENSMUSG00000027111 | -1.14 | -2.203501686 | 1.63E-06 | Itga6 | integrin alpha 6 |
| ENSMUSG00000084113 | -1.14 | -2.202797152 | 8.31E-03 | NA | NA |
| ENSMUSG00000033470 | -1.14 | -2.202782113 | 7.66E-03 | Cysltr2 | cysteinyl leukotriene receptor 2 |
| ENSMUSG00000025199 | -1.14 | -2.202281632 | 3.31E-06 | Chuk | conserved helix-loop-helix ubiquitous kinase |
| ENSMUSG00000082988 | -1.14 | -2.201906439 | 1.01E-03 | NA | NA |
| ENSMUSG00000037860 | -1.14 | -2.201655176 | 4.46E-04 | Aim2 | absent in melanoma 2 |
| ENSMUSG00000014496 | -1.14 | -2.201393532 | 2.27E-03 | Ankrd28 | ankyrin repeat domain 28 |
| ENSMUSG00000031242 | -1.14 | -2.201264535 | 3.03E-07 | NA | NA |
| ENSMUSG00000003131 | -1.14 | -2.200432119 | 4.56E-06 | Pafah1b2 | platelet-activating factor acetylhydrolase, isoform 1b, subunit 2 |
| ENSMUSG00000024083 | -1.14 | -2.200023653 | 2.64E-03 | Pja2 | praja 2, RING-H2 motif containing |
| ENSMUSG00000027778 | -1.14 | -2.19860918 | 1.36E-07 | Ift80 | intraflagellar transport 80 |
| ENSMUSG00000036391 | -1.14 | -2.198558801 | 7.36E-03 | Sec24a | Sec24 related gene family, member A (S. cerevisiae) |
| ENSMUSG00000028291 | -1.14 | -2.19680286 | 2.82E-04 | Akirin2 | akirin 2 |
| ENSMUSG00000021870 | -1.14 | -2.196651757 | 3.45E-06 | Slmap | sarcolemma associated protein |
| ENSMUSG00000081451 | -1.14 | -2.196420284 | 2.84E-03 | NA | NA |
| ENSMUSG00000040648 | -1.14 | -2.196275403 | 5.95E-05 | Ppip5k2 | diphosphoinositol pentakisphosphate kinase 2 |
| ENSMUSG00000034560 | -1.13 | -2.195954641 | 1.40E-04 | A230046K03Rik | RIKEN cDNA A230046K03 gene |
| ENSMUSG00000064127 | -1.13 | -2.19521703 | 4.82E-04 | Med14 | mediator complex subunit 14 |
| ENSMUSG00000026915 | -1.13 | -2.195106656 | 3.48E-05 | Strbp | spermatid perinuclear RNA binding protein |
| ENSMUSG00000062203 | -1.13 | -2.194575566 | 2.12E-05 | Gspt1 | G1 to S phase transition 1 |
| ENSMUSG00000000340 | -1.13 | -2.191964248 | 1.03E-04 | Dbt | dihydrolipoamide branched chain transacylase E2 |
| ENSMUSG00000037818 | -1.13 | -2.191400007 | 1.37E-04 | 3110057O12Rik | RIKEN cDNA 3110057O12 gene |
| ENSMUSG00000036572 | -1.13 | -2.190761797 | 4.89E-04 | Upf3b | UPF3 regulator of nonsense transcripts homolog B (yeast) |
| ENSMUSG00000028343 | -1.13 | -2.18929784 | 3.63E-03 | Erp44 | endoplasmic reticulum protein 44 |
| ENSMUSG00000042408 | -1.13 | -2.189155202 | 7.77E-04 | Zmym6 | zinc finger, MYM-type 6 |
| ENSMUSG00000066551 | -1.13 | -2.187543513 | 4.76E-04 | NA | NA |
| ENSMUSG00000028037 | -1.13 | -2.187523007 | 3.24E-03 | Ifi44 | interferon-induced protein 44 |
| ENSMUSG00000017707 | -1.13 | -2.187522529 | 4.18E-07 | Serinc3 | serine incorporator 3 |
| ENSMUSG00000022000 | -1.13 | -2.186432879 | 8.15E-05 | Zc3h13 | zinc finger CCCH type containing 13 |
| ENSMUSG00000005610 | -1.13 | -2.185029205 | 3.81E-06 | Eif4g2 | eukaryotic translation initiation factor 4, gamma 2 |
| ENSMUSG00000033411 | -1.13 | -2.184866881 | 5.68E-05 | Ctdspl2 | CTD (carboxy-terminal domain, RNA polymerase II, polypeptide A) small phosphatase like 2 |
| ENSMUSG00000031226 | -1.13 | -2.1847547 | 1.45E-03 | Pbdc1 | polysaccharide biosynthesis domain containing 1 |
| ENSMUSG00000012519 | -1.13 | -2.183724141 | 2.64E-03 | Mlkl | mixed lineage kinase domain-like |
| ENSMUSG00000036353 | -1.13 | -2.182698876 | 3.81E-06 | P2ry12 | purinergic receptor P2Y, G-protein coupled 12 |
| ENSMUSG00000026896 | -1.13 | -2.182323724 | 4.90E-05 | Ifih1 | interferon induced with helicase C domain 1 |
| ENSMUSG00000022400 | -1.13 | -2.182265939 | 2.11E-03 | NA | NA |
| ENSMUSG00000032050 | -1.13 | -2.181559863 | 6.01E-06 | Rdx | radixin |
| ENSMUSG00000030521 | -1.12 | -2.17982412 | 3.96E-04 | Mphosph10 | M-phase phosphoprotein 10 (U3 small nucleolar ribonucleoprotein) |
| ENSMUSG00000061186 | -1.12 | -2.179549564 | 1.79E-03 | Sfmbt2 | Scm-like with four mbt domains 2 |
| ENSMUSG00000073707 | -1.12 | -2.178234555 | 4.86E-03 | NA | NA |
| ENSMUSG00000004364 | -1.12 | -2.177243979 | 6.01E-05 | Cul3 | cullin 3 |
| ENSMUSG00000081068 | -1.12 | -2.177114522 | 2.62E-03 | NA | NA |
| ENSMUSG00000038069 | -1.12 | -2.177104483 | 5.99E-04 | Cdkn2aip | CDKN2A interacting protein |
| ENSMUSG00000020362 | -1.12 | -2.176912614 | 1.61E-07 | Cnot6 | CCR4-NOT transcription complex, subunit 6 |
| ENSMUSG00000028382 | -1.12 | -2.173254477 | 1.60E-05 | Ptbp3 | polypyrimidine tract binding protein 3 |
| ENSMUSG00000030921 | -1.12 | -2.173034805 | 1.23E-05 | Trim30a | tripartite motif-containing 30A |
| ENSMUSG00000069204 | -1.12 | -2.172220333 | 1.91E-04 | NA | NA |
| ENSMUSG00000036591 | -1.12 | -2.171851203 | 4.62E-04 | Arhgap21 | Rho GTPase activating protein 21 |
| ENSMUSG00000024087 | -1.12 | -2.17084531 | 3.69E-06 | Cyp1b1 | cytochrome P450, family 1, subfamily b, polypeptide 1 |
| ENSMUSG00000007379 | -1.12 | -2.170089699 | 7.91E-05 | Dennd2c | DENN/MADD domain containing 2C |
| ENSMUSG00000072568 | -1.12 | -2.168048418 | 4.34E-04 | Fam84b | family with sequence similarity 84, member B |
| ENSMUSG00000026579 | -1.12 | -2.167885054 | 3.27E-03 | F5 | coagulation factor V |
| ENSMUSG00000084353 | -1.12 | -2.167399863 | 8.02E-03 | NA | NA |
| ENSMUSG00000034640 | -1.12 | -2.166116445 | 2.13E-04 | Tiparp | TCDD-inducible poly(ADP-ribose) polymerase |
| ENSMUSG00000024238 | -1.11 | -2.165561459 | 4.61E-04 | Zeb1 | zinc finger E-box binding homeobox 1 |
| ENSMUSG00000038206 | -1.11 | -2.16392223 | 7.64E-05 | Fbxo8 | F-box protein 8 |
| ENSMUSG00000020170 | -1.11 | -2.16145739 | 5.15E-05 | Frs2 | fibroblast growth factor receptor substrate 2 |
| ENSMUSG00000027968 | -1.11 | -2.160742735 | 6.31E-04 | Larp7 | La ribonucleoprotein domain family, member 7 |
| ENSMUSG00000047037 | -1.11 | -2.160260474 | 1.15E-04 | Nipa1 | non imprinted in Prader-Willi/Angelman syndrome 1 homolog (human) |
| ENSMUSG00000019961 | -1.11 | -2.159393789 | 1.59E-04 | Tmpo | thymopoietin |
| ENSMUSG00000054889 | -1.11 | -2.159064701 | 8.13E-03 | Dsp | desmoplakin |
| ENSMUSG00000032740 | -1.11 | -2.159052045 | 4.29E-06 | Ccdc88a | coiled coil domain containing 88A |
| ENSMUSG00000044068 | -1.11 | -2.158755351 | 2.97E-04 | Zrsr1 | zinc finger (CCCH type), RNA binding motif and serine/arginine rich 1 |
| ENSMUSG00000004980 | -1.11 | -2.158743856 | 2.27E-05 | Hnrnpa2b1 | heterogeneous nuclear ribonucleoprotein A2/B1 |
| ENSMUSG00000026222 | -1.11 | -2.15820769 | 3.02E-04 | Sp100 | nuclear antigen Sp100 |
| ENSMUSG00000020994 | -1.11 | -2.15722754 | 4.11E-03 | Pnn | pinin |
| ENSMUSG00000061533 | -1.11 | -2.156802333 | 1.15E-04 | Cep128 | centrosomal protein 128 |
| ENSMUSG00000031093 | -1.11 | -2.156616992 | 6.53E-05 | Dock11 | dedicator of cytokinesis 11 |
| ENSMUSG00000035125 | -1.11 | -2.155227811 | 1.99E-03 | Gcfc2 | GC-rich sequence DNA binding factor 2 |
| ENSMUSG00000047139 | -1.11 | -2.15518511 | 3.62E-04 | Cd24a | CD24a antigen |
| ENSMUSG00000035455 | -1.11 | -2.154159805 | 8.97E-06 | Fignl1 | fidgetin-like 1 |
| ENSMUSG00000021770 | -1.11 | -2.153671062 | 3.91E-04 | Samd8 | sterile alpha motif domain containing 8 |
| ENSMUSG00000039617 | -1.11 | -2.152972162 | 5.12E-07 | NA | NA |
| ENSMUSG00000031196 | -1.10 | -2.148369656 | 1.29E-03 | F8 | coagulation factor VIII |
| ENSMUSG00000021669 | -1.10 | -2.147103018 | 8.26E-03 | Col4a3bp | collagen, type IV, alpha 3 (Goodpasture antigen) binding protein |
| ENSMUSG00000024091 | -1.10 | -2.145272379 | 8.93E-04 | Vapa | vesicle-associated membrane protein, associated protein A |
| ENSMUSG00000045962 | -1.10 | -2.144675848 | 2.00E-05 | Wnk1 | WNK lysine deficient protein kinase 1 |
| ENSMUSG00000043929 | -1.10 | -2.144092022 | 8.90E-03 | Klhl15 | kelch-like 15 |
| ENSMUSG00000030156 | -1.10 | -2.144064671 | 3.42E-03 | Cd69 | CD69 antigen |
| ENSMUSG00000079418 | -1.10 | -2.143857021 | 9.96E-05 | Atg4a | autophagy related 4A, cysteine peptidase |
| ENSMUSG00000053453 | -1.10 | -2.143503208 | 2.81E-05 | Thoc7 | THO complex 7 homolog (Drosophila) |
| ENSMUSG00000042348 | -1.10 | -2.143218095 | 1.26E-04 | Arl15 | ADP-ribosylation factor-like 15 |
| ENSMUSG00000058706 | -1.10 | -2.143179448 | 1.44E-03 | 0610030E20Rik | RIKEN cDNA 0610030E20 gene |
| ENSMUSG00000025612 | -1.10 | -2.142351064 | 9.93E-04 | Bach1 | BTB and CNC homology 1 |
| ENSMUSG00000042508 | -1.10 | -2.140934031 | 4.37E-05 | Dmtf1 | cyclin D binding myb-like transcription factor 1 |
| ENSMUSG00000041408 | -1.10 | -2.139216334 | 4.44E-05 | Wapal | wings apart-like homolog (Drosophila) |
| ENSMUSG00000005800 | -1.10 | -2.13821226 | 6.08E-03 | Mmp8 | matrix metallopeptidase 8 |
| ENSMUSG00000024576 | -1.10 | -2.138194405 | 3.20E-07 | Csnk1a1 | casein kinase 1, alpha 1 |
| ENSMUSG00000036323 | -1.09 | -2.135880374 | 1.09E-06 | Srp72 | signal recognition particle 72 |
| ENSMUSG00000031355 | -1.09 | -2.135657558 | 8.34E-07 | Arhgap6 | Rho GTPase activating protein 6 |
| ENSMUSG00000020687 | -1.09 | -2.135383821 | 3.25E-04 | Cdc27 | cell division cycle 27 |
| ENSMUSG00000000708 | -1.09 | -2.135162237 | 5.46E-05 | Kat2b | K(lysine) acetyltransferase 2B |
| ENSMUSG00000050310 | -1.09 | -2.134015748 | 1.50E-03 | Rictor | RPTOR independent companion of MTOR, complex 2 |
| ENSMUSG00000034801 | -1.09 | -2.132980934 | 9.70E-05 | Sos2 | son of sevenless homolog 2 (Drosophila) |
| ENSMUSG00000024789 | -1.09 | -2.131926582 | 8.43E-06 | Jak2 | Janus kinase 2 |
| ENSMUSG00000079555 | -1.09 | -2.131828549 | 8.74E-04 | Haus3 | HAUS augmin-like complex, subunit 3 |
| ENSMUSG00000027589 | -1.09 | -2.131263182 | 2.03E-05 | Pcmtd2 | protein-L-isoaspartate (D-aspartate) O-methyltransferase domain containing 2 |
| ENSMUSG00000056579 | -1.09 | -2.131176577 | 4.34E-06 | Tug1 | taurine upregulated gene 1 |
| ENSMUSG00000020561 | -1.09 | -2.130337518 | 3.22E-03 | Twistnb | TWIST neighbor |
| ENSMUSG00000006262 | -1.09 | -2.129645847 | 6.86E-07 | Mob1b | MOB kinase activator 1B |
| ENSMUSG00000037999 | -1.09 | -2.129628766 | 1.86E-03 | Arap2 | ArfGAP with RhoGAP domain, ankyrin repeat and PH domain 2 |
| ENSMUSG00000002997 | -1.09 | -2.129146623 | 6.37E-05 | Prkar2b | protein kinase, cAMP dependent regulatory, type II beta |
| ENSMUSG00000027835 | -1.09 | -2.128228021 | 3.77E-03 | Pdcd10 | programmed cell death 10 |
| ENSMUSG00000025220 | -1.09 | -2.12811665 | 1.48E-04 | Mgea5 | meningioma expressed antigen 5 (hyaluronidase) |
| ENSMUSG00000024241 | -1.09 | -2.127336293 | 3.13E-05 | Sos1 | son of sevenless homolog 1 (Drosophila) |
| ENSMUSG00000020990 | -1.09 | -2.125521562 | 2.60E-03 | Cdkl1 | cyclin-dependent kinase-like 1 (CDC2-related kinase) |
| ENSMUSG00000073542 | -1.09 | -2.125258772 | 2.72E-05 | Cep76 | centrosomal protein 76 |
| ENSMUSG00000035024 | -1.09 | -2.125241183 | 1.59E-04 | Ncapd3 | non-SMC condensin II complex, subunit D3 |
| ENSMUSG00000004096 | -1.09 | -2.124920853 | 3.94E-03 | Cwc15 | CWC15 homolog (S. cerevisiae) |
| ENSMUSG00000040710 | -1.09 | -2.124884653 | 5.77E-04 | St8sia4 | ST8 alpha-N-acetyl-neuraminide alpha-2,8-sialyltransferase 4 |
| ENSMUSG00000027346 | -1.09 | -2.124800044 | 3.84E-07 | Gpcpd1 | glycerophosphocholine phosphodiesterase GDE1 homolog (S. cerevisiae) |
| ENSMUSG00000031262 | -1.09 | -2.124135681 | 1.55E-04 | Cenpi | centromere protein I |
| ENSMUSG00000034192 | -1.09 | -2.121917171 | 7.64E-03 | Lsm3 | LSM3 homolog, U6 small nuclear RNA associated (S. cerevisiae) |
| ENSMUSG00000026348 | -1.09 | -2.121399023 | 7.46E-03 | Acmsd | amino carboxymuconate semialdehyde decarboxylase |
| ENSMUSG00000035941 | -1.08 | -2.121146486 | 1.24E-04 | Ibtk | inhibitor of Bruton agammaglobulinemia tyrosine kinase |
| ENSMUSG00000024513 | -1.08 | -2.119901591 | 9.81E-04 | Mbd2 | methyl-CpG binding domain protein 2 |
| ENSMUSG00000038535 | -1.08 | -2.119157296 | 1.29E-08 | Zfp280d | zinc finger protein 280D |
| ENSMUSG00000046434 | -1.08 | -2.117960674 | 3.70E-04 | NA | NA |
| ENSMUSG00000029761 | -1.08 | -2.117426833 | 1.93E-04 | Cald1 | caldesmon 1 |
| ENSMUSG00000026516 | -1.08 | -2.117293213 | 1.26E-05 | Nvl | nuclear VCP-like |
| ENSMUSG00000028332 | -1.08 | -2.116231595 | 2.30E-03 | Hemgn | hemogen |
| ENSMUSG00000036902 | -1.08 | -2.116192739 | 2.84E-03 | Neto2 | neuropilin (NRP) and tolloid (TLL)-like 2 |
| ENSMUSG00000026672 | -1.08 | -2.113773889 | 1.67E-04 | Optn | optineurin |
| ENSMUSG00000035798 | -1.08 | -2.113225437 | 6.29E-03 | Zdhhc17 | zinc finger, DHHC domain containing 17 |
| ENSMUSG00000021022 | -1.08 | -2.109804434 | 2.56E-03 | Ppp2r3c | protein phosphatase 2, regulatory subunit B'', gamma |
| ENSMUSG00000023169 | -1.08 | -2.109308632 | 5.65E-03 | Slc38a1 | solute carrier family 38, member 1 |
| ENSMUSG00000073647 | -1.08 | -2.108885086 | 5.90E-03 | NA | NA |
| ENSMUSG00000028328 | -1.08 | -2.108765105 | 6.95E-05 | Tmod1 | tropomodulin 1 |
| ENSMUSG00000032561 | -1.08 | -2.107485797 | 5.60E-03 | Acpp | acid phosphatase, prostate |
| ENSMUSG00000053740 | -1.07 | -2.106638705 | 1.78E-03 | NA | NA |
| ENSMUSG00000022961 | -1.07 | -2.106069582 | 1.24E-03 | Son | Son DNA binding protein |
| ENSMUSG00000041633 | -1.07 | -2.10557244 | 1.75E-03 | Kctd12b | potassium channel tetramerisation domain containing 12b |
| ENSMUSG00000031278 | -1.07 | -2.105469686 | 1.52E-03 | Acsl4 | acyl-CoA synthetase long-chain family member 4 |
| ENSMUSG00000086635 | -1.07 | -2.105445306 | 7.15E-03 | NA | NA |
| ENSMUSG00000026020 | -1.07 | -2.105229181 | 4.52E-05 | Nop58 | NOP58 ribonucleoprotein |
| ENSMUSG00000037119 | -1.07 | -2.103831756 | 1.14E-03 | D15Ertd621e | DNA segment, Chr 15, ERATO Doi 621, expressed |
| ENSMUSG00000047466 | -1.07 | -2.102813628 | 4.99E-03 | 8030462N17Rik | RIKEN cDNA 8030462N17 gene |
| ENSMUSG00000028577 | -1.07 | -2.10217844 | 5.33E-04 | Plaa | phospholipase A2, activating protein |
| ENSMUSG00000040225 | -1.07 | -2.102177888 | 2.21E-03 | Prrc2c | proline-rich coiled-coil 2C |
| ENSMUSG00000034021 | -1.07 | -2.101313388 | 2.36E-06 | Pds5b | PDS5, regulator of cohesion maintenance, homolog B (S. cerevisiae) |
| ENSMUSG00000020330 | -1.07 | -2.100890153 | 1.15E-04 | Hmmr | hyaluronan mediated motility receptor (RHAMM) |
| ENSMUSG00000054752 | -1.07 | -2.100825533 | 6.62E-03 | Fsd1l | fibronectin type III and SPRY domain containing 1-like |
| ENSMUSG00000021392 | -1.07 | -2.100798905 | 6.61E-04 | Nol8 | nucleolar protein 8 |
| ENSMUSG00000024384 | -1.07 | -2.100486803 | 4.19E-06 | Iws1 | IWS1 homolog (S. cerevisiae) |
| ENSMUSG00000059811 | -1.07 | -2.099431574 | 4.09E-04 | Atl2 | atlastin GTPase 2 |
| ENSMUSG00000047735 | -1.07 | -2.098102057 | 3.55E-05 | Samd9l | sterile alpha motif domain containing 9-like |
| ENSMUSG00000026761 | -1.07 | -2.096806212 | 3.11E-04 | Orc4 | origin recognition complex, subunit 4 |
| ENSMUSG00000059586 | -1.07 | -2.095791763 | 6.27E-04 | Nsmce2 | non-SMC element 2 homolog (MMS21, S. cerevisiae) |
| ENSMUSG00000020530 | -1.07 | -2.09429976 | 2.93E-06 | Ggnbp2 | gametogenetin binding protein 2 |
| ENSMUSG00000021374 | -1.07 | -2.094058896 | 4.36E-04 | Nup153 | nucleoporin 153 |
| ENSMUSG00000015340 | -1.07 | -2.093754954 | 8.85E-05 | Cybb | cytochrome b-245, beta polypeptide |
| ENSMUSG00000027193 | -1.07 | -2.093024244 | 3.53E-08 | Api5 | apoptosis inhibitor 5 |
| ENSMUSG00000044268 | -1.07 | -2.092310259 | 2.21E-04 | NA | NA |
| ENSMUSG00000026393 | -1.06 | -2.092011324 | 3.76E-04 | Nek7 | NIMA (never in mitosis gene a)-related expressed kinase 7 |
| ENSMUSG00000036990 | -1.06 | -2.091151756 | 1.33E-03 | Otud4 | OTU domain containing 4 |
| ENSMUSG00000031843 | -1.06 | -2.091052516 | 6.40E-04 | Mphosph6 | M phase phosphoprotein 6 |
| ENSMUSG00000036825 | -1.06 | -2.089924916 | 1.19E-04 | Ssx2ip | synovial sarcoma, X breakpoint 2 interacting protein |
| ENSMUSG00000019943 | -1.06 | -2.089884811 | 1.90E-03 | Atp2b1 | ATPase, Ca++ transporting, plasma membrane 1 |
| ENSMUSG00000028906 | -1.06 | -2.089554086 | 1.71E-04 | Epb4.1 | erythrocyte protein band 4.1 |
| ENSMUSG00000029613 | -1.06 | -2.089407068 | 3.07E-05 | Eif2ak1 | eukaryotic translation initiation factor 2 alpha kinase 1 |
| ENSMUSG00000020634 | -1.06 | -2.089080962 | 7.68E-05 | Ubxn2a | UBX domain protein 2A |
| ENSMUSG00000030704 | -1.06 | -2.087676443 | 3.01E-06 | Rab6a | RAB6A, member RAS oncogene family |
| ENSMUSG00000018199 | -1.06 | -2.086873555 | 1.72E-04 | Trove2 | TROVE domain family, member 2 |
| ENSMUSG00000000600 | -1.06 | -2.086570084 | 1.50E-05 | Krit1 | KRIT1, ankyrin repeat containing |
| ENSMUSG00000039701 | -1.06 | -2.086157684 | 2.89E-03 | Usp53 | ubiquitin specific peptidase 53 |
| ENSMUSG00000032400 | -1.06 | -2.085755863 | 1.12E-04 | Zwilch | zwilch kinetochore protein |
| ENSMUSG00000037474 | -1.06 | -2.085048467 | 4.83E-04 | Dtl | denticleless homolog (Drosophila) |
| ENSMUSG00000052446 | -1.06 | -2.084724436 | 2.08E-04 | Zfp961 | zinc finger protein 961 |
| ENSMUSG00000022364 | -1.06 | -2.084283152 | 9.57E-04 | Tbc1d31 | TBC1 domain family, member 31 |
| ENSMUSG00000032397 | -1.06 | -2.083203945 | 1.48E-04 | Tipin | timeless interacting protein |
| ENSMUSG00000044906 | -1.06 | -2.082144394 | 3.52E-04 | 4930503L19Rik | RIKEN cDNA 4930503L19 gene |
| ENSMUSG00000032349 | -1.06 | -2.081649982 | 1.41E-07 | Elovl5 | ELOVL family member 5, elongation of long chain fatty acids (yeast) |
| ENSMUSG00000026196 | -1.06 | -2.080967098 | 4.11E-04 | Bard1 | BRCA1 associated RING domain 1 |
| ENSMUSG00000021500 | -1.06 | -2.080646826 | 9.70E-05 | Ddx46 | DEAD (Asp-Glu-Ala-Asp) box polypeptide 46 |
| ENSMUSG00000006169 | -1.06 | -2.080020725 | 7.08E-05 | Clint1 | clathrin interactor 1 |
| ENSMUSG00000020659 | -1.06 | -2.079730368 | 7.45E-05 | Cbll1 | Casitas B-lineage lymphoma-like 1 |
| ENSMUSG00000078143 | -1.06 | -2.07970005 | 8.07E-03 | NA | NA |
| ENSMUSG00000027680 | -1.06 | -2.079124529 | 2.62E-03 | Fxr1 | fragile X mental retardation gene 1, autosomal homolog |
| ENSMUSG00000038943 | -1.06 | -2.078747635 | 3.93E-04 | Prc1 | protein regulator of cytokinesis 1 |
| ENSMUSG00000062593 | -1.06 | -2.078493527 | 5.83E-05 | Lilrb4 | leukocyte immunoglobulin-like receptor, subfamily B, member 4 |
| ENSMUSG00000086229 | -1.06 | -2.078432131 | 2.65E-03 | NA | NA |
| ENSMUSG00000027882 | -1.06 | -2.077751732 | 1.02E-05 | Stxbp3a | syntaxin binding protein 3A |
| ENSMUSG00000025925 | -1.05 | -2.077636753 | 3.26E-04 | Terf1 | telomeric repeat binding factor 1 |
| ENSMUSG00000037234 | -1.05 | -2.077606615 | 1.31E-05 | Hook3 | hook homolog 3 (Drosophila) |
| ENSMUSG00000033964 | -1.05 | -2.077139126 | 1.92E-03 | Zbtb41 | zinc finger and BTB domain containing 41 homolog |
| ENSMUSG00000059474 | -1.05 | -2.0763295 | 2.16E-05 | Mbtd1 | mbt domain containing 1 |
| ENSMUSG00000038005 | -1.05 | -2.076120846 | 4.10E-04 | 2700029M09Rik | RIKEN cDNA 2700029M09 gene |
| ENSMUSG00000059237 | -1.05 | -2.075346213 | 1.38E-03 | NA | NA |
| ENSMUSG00000035246 | -1.05 | -2.07511127 | 9.86E-04 | Pcyt1b | phosphate cytidylyltransferase 1, choline, beta isoform |
| ENSMUSG00000023921 | -1.05 | -2.074403206 | 6.94E-06 | Mut | methylmalonyl-Coenzyme A mutase |
| ENSMUSG00000044795 | -1.05 | -2.073755379 | 3.06E-04 | Cyb5d1 | cytochrome b5 domain containing 1 |
| ENSMUSG00000032386 | -1.05 | -2.073181072 | 1.56E-05 | Trip4 | thyroid hormone receptor interactor 4 |
| ENSMUSG00000028261 | -1.05 | -2.073006533 | 5.27E-03 | Ndufaf4 | NADH dehydrogenase (ubiquinone) 1 alpha subcomplex, assembly factor 4 |
| ENSMUSG00000029623 | -1.05 | -2.071744174 | 3.84E-04 | Pdap1 | PDGFA associated protein 1 |
| ENSMUSG00000022003 | -1.05 | -2.071051054 | 1.33E-04 | Slc25a30 | solute carrier family 25, member 30 |
| ENSMUSG00000037896 | -1.05 | -2.070856466 | 1.68E-03 | Rcor1 | REST corepressor 1 |
| ENSMUSG00000026112 | -1.05 | -2.070729232 | 1.06E-05 | Coa5 | cytochrome C oxidase assembly factor 5 |
| ENSMUSG00000027365 | -1.05 | -2.070642001 | 3.67E-05 | Trpm7 | transient receptor potential cation channel, subfamily M, member 7 |
| ENSMUSG00000027620 | -1.05 | -2.070242306 | 5.14E-06 | Rbm39 | RNA binding motif protein 39 |
| ENSMUSG00000031201 | -1.05 | -2.070153722 | 2.81E-04 | Brcc3 | BRCA1/BRCA2-containing complex, subunit 3 |
| ENSMUSG00000015961 | -1.05 | -2.068719279 | 6.08E-07 | Adss | adenylosuccinate synthetase, non muscle |
| ENSMUSG00000021282 | -1.05 | -2.068647364 | 1.32E-04 | Eif5 | eukaryotic translation initiation factor 5 |
| ENSMUSG00000035851 | -1.05 | -2.068530626 | 9.52E-06 | Ythdc1 | YTH domain containing 1 |
| ENSMUSG00000005233 | -1.05 | -2.068496471 | 5.57E-04 | Spc25 | SPC25, NDC80 kinetochore complex component, homolog (S. cerevisiae) |
| ENSMUSG00000081792 | -1.05 | -2.068440515 | 6.99E-03 | NA | NA |
| ENSMUSG00000084230 | -1.05 | -2.067877139 | 2.86E-05 | NA | NA |
| ENSMUSG00000033943 | -1.05 | -2.067603718 | 6.70E-04 | Mga | MAX gene associated |
| ENSMUSG00000032113 | -1.05 | -2.067521566 | 3.71E-04 | Chek1 | checkpoint kinase 1 |
| ENSMUSG00000005362 | -1.05 | -2.066543334 | 9.95E-04 | Crbn | cereblon |
| ENSMUSG00000034723 | -1.05 | -2.06627892 | 7.50E-04 | Tmx4 | thioredoxin-related transmembrane protein 4 |
| ENSMUSG00000040549 | -1.05 | -2.064958218 | 1.28E-06 | Ckap5 | cytoskeleton associated protein 5 |
| ENSMUSG00000034311 | -1.05 | -2.064889164 | 6.58E-04 | Kif4 | kinesin family member 4 |
| ENSMUSG00000027427 | -1.05 | -2.064669032 | 8.78E-06 | Polr3f | polymerase (RNA) III (DNA directed) polypeptide F |
| ENSMUSG00000063884 | -1.05 | -2.064467532 | 4.75E-04 | Ptcd3 | pentatricopeptide repeat domain 3 |
| ENSMUSG00000028370 | -1.05 | -2.063890235 | 2.09E-03 | Pappa | pregnancy-associated plasma protein A |
| ENSMUSG00000047757 | -1.04 | -2.063346763 | 4.49E-03 | Fancb | Fanconi anemia, complementation group B |
| ENSMUSG00000036928 | -1.04 | -2.061693617 | 5.79E-03 | Stag3 | stromal antigen 3 |
| ENSMUSG00000031984 | -1.04 | -2.061308716 | 7.11E-05 | 2810004N23Rik | RIKEN cDNA 2810004N23 gene |
| ENSMUSG00000030978 | -1.04 | -2.060189688 | 2.66E-08 | Rrm1 | ribonucleotide reductase M1 |
| ENSMUSG00000022020 | -1.04 | -2.056853307 | 1.22E-05 | Naa16 | N(alpha)-acetyltransferase 16, NatA auxiliary subunit |
| ENSMUSG00000081289 | -1.04 | -2.055504409 | 4.40E-03 | NA | NA |
| ENSMUSG00000028224 | -1.04 | -2.055342742 | 2.13E-03 | Nbn | nibrin |
| ENSMUSG00000020413 | -1.04 | -2.05530793 | 1.62E-06 | Hus1 | Hus1 homolog (S. pombe) |
| ENSMUSG00000023883 | -1.04 | -2.054709119 | 5.10E-04 | Phf10 | PHD finger protein 10 |
| ENSMUSG00000024135 | -1.04 | -2.05460337 | 3.73E-06 | Srbd1 | S1 RNA binding domain 1 |
| ENSMUSG00000056025 | -1.04 | -2.053407174 | 7.95E-03 | Clca1 | chloride channel calcium activated 1 |
| ENSMUSG00000037533 | -1.04 | -2.052557048 | 3.62E-03 | Rapgef6 | Rap guanine nucleotide exchange factor (GEF) 6 |
| ENSMUSG00000082458 | -1.04 | -2.052478629 | 3.52E-03 | NA | NA |
| ENSMUSG00000049493 | -1.04 | -2.051930934 | 2.24E-04 | Pls1 | plastin 1 (I-isoform) |
| ENSMUSG00000001986 | -1.04 | -2.051791862 | 1.19E-03 | Gria3 | glutamate receptor, ionotropic, AMPA3 (alpha 3) |
| ENSMUSG00000028053 | -1.04 | -2.051377224 | 2.10E-04 | Ash1l | ash1 (absent, small, or homeotic)-like (Drosophila) |
| ENSMUSG00000038766 | -1.04 | -2.051234543 | 2.60E-03 | Gabpb2 | GA repeat binding protein, beta 2 |
| ENSMUSG00000020185 | -1.04 | -2.049886485 | 1.76E-03 | E2f7 | E2F transcription factor 7 |
| ENSMUSG00000024800 | -1.04 | -2.049784406 | 5.58E-03 | Rpp30 | ribonuclease P/MRP 30 subunit |
| ENSMUSG00000046351 | -1.04 | -2.049446723 | 3.57E-03 | Zfp322a | zinc finger protein 322A |
| ENSMUSG00000074934 | -1.03 | -2.048667913 | 4.77E-04 | Grem1 | gremlin 1 |
| ENSMUSG00000037343 | -1.03 | -2.047127055 | 3.10E-05 | Taf2 | TAF2 RNA polymerase II, TATA box binding protein (TBP)-associated factor |
| ENSMUSG00000040351 | -1.03 | -2.046532375 | 4.18E-04 | Ankib1 | ankyrin repeat and IBR domain containing 1 |
| ENSMUSG00000022016 | -1.03 | -2.0461339 | 2.45E-04 | Akap11 | A kinase (PRKA) anchor protein 11 |
| ENSMUSG00000084159 | -1.03 | -2.045611414 | 5.35E-03 | NA | NA |
| ENSMUSG00000026187 | -1.03 | -2.045567662 | 3.31E-04 | Xrcc5 | X-ray repair complementing defective repair in Chinese hamster cells 5 |
| ENSMUSG00000031918 | -1.03 | -2.044783005 | 1.85E-04 | Mtmr2 | myotubularin related protein 2 |
| ENSMUSG00000024228 | -1.03 | -2.042368779 | 4.83E-03 | Nudt12 | nudix (nucleoside diphosphate linked moiety X)-type motif 12 |
| ENSMUSG00000030423 | -1.03 | -2.042238217 | 5.21E-03 | Pop4 | processing of precursor 4, ribonuclease P/MRP family, (S. cerevisiae) |
| ENSMUSG00000004880 | -1.03 | -2.041433628 | 9.27E-04 | Lbr | lamin B receptor |
| ENSMUSG00000032570 | -1.03 | -2.041420254 | 3.12E-04 | Atp2c1 | ATPase, Ca++-sequestering |
| ENSMUSG00000027770 | -1.03 | -2.040976545 | 1.85E-04 | Dhx36 | DEAH (Asp-Glu-Ala-His) box polypeptide 36 |
| ENSMUSG00000008976 | -1.03 | -2.040957584 | 1.43E-04 | Gabpa | GA repeat binding protein, alpha |
| ENSMUSG00000041417 | -1.03 | -2.04077151 | 1.19E-04 | Pik3r1 | phosphatidylinositol 3-kinase, regulatory subunit, polypeptide 1 (p85 alpha) |
| ENSMUSG00000035171 | -1.03 | -2.040516284 | 8.15E-03 | 1110059E24Rik | RIKEN cDNA 1110059E24 gene |
| ENSMUSG00000020900 | -1.03 | -2.040058993 | 1.22E-03 | Myh10 | myosin, heavy polypeptide 10, non-muscle |
| ENSMUSG00000040270 | -1.03 | -2.040011464 | 1.48E-03 | Bach2 | BTB and CNC homology 2 |
| ENSMUSG00000027804 | -1.03 | -2.039925347 | 2.93E-06 | Ppid | peptidylprolyl isomerase D (cyclophilin D) |
| ENSMUSG00000033596 | -1.03 | -2.039754333 | 6.45E-06 | Rfwd3 | ring finger and WD repeat domain 3 |
| ENSMUSG00000036898 | -1.03 | -2.039059642 | 6.89E-03 | Zfp157 | zinc finger protein 157 |
| ENSMUSG00000032621 | -1.03 | -2.038526221 | 9.96E-05 | Srek1 | splicing regulatory glutamine/lysine-rich protein 1 |
| ENSMUSG00000041229 | -1.03 | -2.037562621 | 1.81E-05 | Phf8 | PHD finger protein 8 |
| ENSMUSG00000028394 | -1.03 | -2.036529543 | 1.58E-04 | Pole3 | polymerase (DNA directed), epsilon 3 (p17 subunit) |
| ENSMUSG00000054720 | -1.03 | -2.036508479 | 2.44E-08 | Lrrc8c | leucine rich repeat containing 8 family, member C |
| ENSMUSG00000022827 | -1.03 | -2.036017783 | 1.53E-03 | Rabl3 | RAB, member of RAS oncogene family-like 3 |
| ENSMUSG00000019982 | -1.03 | -2.035820235 | 1.55E-03 | Myb | myeloblastosis oncogene |
| ENSMUSG00000036469 | -1.03 | -2.03562176 | 5.81E-04 | March1 | membrane-associated ring finger (C3HC4) 1 |
| ENSMUSG00000020863 | -1.03 | -2.035407462 | 1.74E-05 | Luc7l3 | LUC7-like 3 (S. cerevisiae) |
| ENSMUSG00000029802 | -1.02 | -2.034795169 | 9.29E-06 | Abcg2 | ATP-binding cassette, sub-family G (WHITE), member 2 |
| ENSMUSG00000028522 | -1.02 | -2.034291615 | 8.12E-05 | Mier1 | mesoderm induction early response 1 homolog (Xenopus laevis |
| ENSMUSG00000027715 | -1.02 | -2.031448812 | 2.22E-04 | Ccna2 | cyclin A2 |
| ENSMUSG00000027829 | -1.02 | -2.030362414 | 1.83E-04 | Ccnl1 | cyclin L1 |
| ENSMUSG00000053846 | -1.02 | -2.030333919 | 4.85E-03 | Lipg | lipase, endothelial |
| ENSMUSG00000071281 | -1.02 | -2.029927545 | 2.97E-03 | Zfp65 | zinc finger protein 65 |
| ENSMUSG00000022141 | -1.02 | -2.028345536 | 8.47E-06 | Nipbl | Nipped-B homolog (Drosophila) |
| ENSMUSG00000018160 | -1.02 | -2.028151688 | 2.48E-03 | Med1 | mediator complex subunit 1 |
| ENSMUSG00000035367 | -1.02 | -2.02785582 | 5.76E-04 | Rmi1 | RMI1, RecQ mediated genome instability 1, homolog (S. cerevisiae) |
| ENSMUSG00000064351 | -1.02 | -2.026485235 | 3.58E-03 | COX1 | cytochrome c oxidase subunit I |
| ENSMUSG00000032411 | -1.02 | -2.025705497 | 5.32E-04 | Tfdp2 | transcription factor Dp 2 |
| ENSMUSG00000013662 | -1.02 | -2.024810884 | 3.58E-05 | Atad1 | ATPase family, AAA domain containing 1 |
| ENSMUSG00000028528 | -1.02 | -2.02360555 | 8.31E-03 | Dnajc6 | DnaJ (Hsp40) homolog, subfamily C, member 6 |
| ENSMUSG00000024887 | -1.02 | -2.022738348 | 6.40E-03 | Asah2 | N-acylsphingosine amidohydrolase 2 |
| ENSMUSG00000022673 | -1.02 | -2.021859715 | 1.76E-06 | Mcm4 | minichromosome maintenance deficient 4 homolog (S. cerevisiae) |
| ENSMUSG00000001542 | -1.02 | -2.021674997 | 4.26E-05 | Ell2 | elongation factor RNA polymerase II 2 |
| ENSMUSG00000039233 | -1.01 | -2.019878001 | 2.51E-06 | Tbce | tubulin-specific chaperone E |
| ENSMUSG00000029014 | -1.01 | -2.019755349 | 3.98E-03 | Dnajc2 | DnaJ (Hsp40) homolog, subfamily C, member 2 |
| ENSMUSG00000023846 | -1.01 | -2.018702605 | 7.33E-05 | Riok2 | RIO kinase 2 (yeast) |
| ENSMUSG00000022337 | -1.01 | -2.018096425 | 4.02E-03 | Emc2 | ER membrane protein complex subunit 2 |
| ENSMUSG00000042105 | -1.01 | -2.017534114 | 1.03E-03 | Inpp5f | inositol polyphosphate-5-phosphatase F |
| ENSMUSG00000029414 | -1.01 | -2.017257099 | 4.42E-04 | Kntc1 | kinetochore associated 1 |
| ENSMUSG00000033014 | -1.01 | -2.016223826 | 3.58E-03 | Trim33 | tripartite motif-containing 33 |
| ENSMUSG00000068115 | -1.01 | -2.015989987 | 2.75E-04 | Ninl | ninein-like |
| ENSMUSG00000039634 | -1.01 | -2.014780658 | 5.89E-03 | Zfp189 | zinc finger protein 189 |
| ENSMUSG00000073792 | -1.01 | -2.014026559 | 3.15E-03 | Alg6 | asparagine-linked glycosylation 6 (alpha-1,3,-glucosyltransferase) |
| ENSMUSG00000028098 | -1.01 | -2.012722572 | 2.00E-05 | Rnf115 | ring finger protein 115 |
| ENSMUSG00000006715 | -1.01 | -2.011851879 | 4.70E-04 | Gmnn | geminin |
| ENSMUSG00000058298 | -1.01 | -2.0117462 | 1.03E-04 | Mcm9 | minichromosome maintenance complex component 9 |
| ENSMUSG00000039630 | -1.01 | -2.011685751 | 8.73E-05 | Hnrnpu | heterogeneous nuclear ribonucleoprotein U |
| ENSMUSG00000023025 | -1.01 | -2.01078379 | 2.91E-03 | Larp4 | La ribonucleoprotein domain family, member 4 |
| ENSMUSG00000048661 | -1.01 | -2.010671195 | 1.36E-03 | Lemd3 | LEM domain containing 3 |
| ENSMUSG00000027469 | -1.01 | -2.010643618 | 1.72E-04 | Tpx2 | TPX2, microtubule-associated protein homolog (Xenopus laevis) |
| ENSMUSG00000030059 | -1.01 | -2.01046846 | 1.27E-03 | Tmf1 | TATA element modulatory factor 1 |
| ENSMUSG00000029521 | -1.01 | -2.008893717 | 5.24E-05 | Chek2 | checkpoint kinase 2 |
| ENSMUSG00000068959 | -1.01 | -2.008370768 | 8.61E-03 | Zfp619 | zinc finger protein 619 |
| ENSMUSG00000052331 | -1.01 | -2.008366807 | 4.35E-03 | Ankrd44 | ankyrin repeat domain 44 |
| ENSMUSG00000026723 | -1.01 | -2.00706646 | 2.24E-03 | Trdmt1 | tRNA aspartic acid methyltransferase 1 |
| ENSMUSG00000028883 | -1.00 | -2.00666968 | 1.76E-03 | Sema3a | sema domain, immunoglobulin domain (Ig), short basic domain, secreted, (semaphorin) 3A |
| ENSMUSG00000002289 | -1.00 | -2.006530891 | 1.61E-03 | Angptl4 | angiopoietin-like 4 |
| ENSMUSG00000055204 | -1.00 | -2.006404857 | 4.52E-03 | Ankrd17 | ankyrin repeat domain 17 |
| ENSMUSG00000033847 | -1.00 | -2.005230429 | 2.41E-03 | Pla2g4c | phospholipase A2, group IVC (cytosolic, calcium-independent) |
| ENSMUSG00000029833 | -1.00 | -2.005112049 | 8.93E-04 | Trim24 | tripartite motif-containing 24 |
| ENSMUSG00000070733 | -1.00 | -2.004936685 | 3.63E-03 | Fryl | furry homolog-like (Drosophila) |
| ENSMUSG00000021408 | -1.00 | -2.00492358 | 6.86E-05 | Ripk1 | receptor (TNFRSF)-interacting serine-threonine kinase 1 |
| ENSMUSG00000027091 | -1.00 | -2.004026729 | 9.48E-05 | Zc3h15 | zinc finger CCCH-type containing 15 |
| ENSMUSG00000032350 | -1.00 | -2.00392588 | 1.37E-04 | Gclc | glutamate-cysteine ligase, catalytic subunit |
| ENSMUSG00000033502 | -1.00 | -2.003053518 | 6.57E-05 | Cdc14a | CDC14 cell division cycle 14A |
| ENSMUSG00000034663 | -1.00 | -2.002290202 | 1.81E-06 | Bmp2k | BMP2 inducible kinase |
| ENSMUSG00000009030 | -1.00 | -2.001822045 | 1.68E-03 | Pdcl | phosducin-like |
| ENSMUSG00000071359 | -1.00 | -2.001292625 | 2.79E-03 | Tbpl1 | TATA box binding protein-like 1 |
| ENSMUSG00000035868 | -1.00 | -2.001073497 | 2.06E-04 | 3110052M02Rik | RIKEN cDNA 3110052M02 gene |
| ENSMUSG00000015755 | -1.00 | -2.000885106 | 5.30E-04 | Tab2 | TGF-beta activated kinase 1/MAP3K7 binding protein 2 |
| ENSMUSG00000035437 | -1.00 | -2.000551938 | 1.57E-03 | Rabgap1 | RAB GTPase activating protein 1 |
| ENSMUSG00000033543 | -1.00 | -2.000494124 | 2.85E-04 | Gtf2a2 | general transcription factor II A, 2 |
| ENSMUSG00000042699 | -1.00 | -2.000254216 | 1.07E-07 | Dhx9 | DEAH (Asp-Glu-Ala-His) box polypeptide 9 |
