# Supplementary material for: Osteolytic cancer cells induce vascular/axon guidance processes in the bone/bone marrow stroma
Source: Oncotarget. 2018 Jun 22;9(48):28877–96. doi: 10.18632/oncotarget.25608 (PMC6034746; doi:10.18632/oncotarget.25608)
Supplement: Supplementary file 3 [file oncotarget-09-28877-s003.docx]

**Table S2.**

| id | log2FoldChange | FC | padj | SYMBOL | GENENAME |
| --- | --- | --- | --- | --- | --- |
| ENSMUSG00000056050 | 11.45 | 2791.46 | 1.98E-83 | Mia3 | melanoma inhibitory activity 3 |
| ENSMUSG00000042901 | 10.12 | 1114.43 | 3.29E-60 | Aida | axin interactor, dorsalization associated |
| ENSMUSG00000046836 | 9.99 | 1014.46 | 2.06E-58 | Brox | BRO1 domain and CAAX motif containing |
| ENSMUSG00000059256 | 9.83 | 912.87 | 1.60E-61 | Gzmd | granzyme D |
| ENSMUSG00000079084 | 9.77 | 870.29 | 1.66E-54 | Ccdc82 | coiled-coil domain containing 82 |
| ENSMUSG00000051412 | 9.13 | 561.25 | 2.69E-46 | Vamp7 | vesicle-associated membrane protein 7 |
| ENSMUSG00000067038 | 9.10 | 546.96 | 2.21E-52 | NA | NA |
| ENSMUSG00000063897 | 9.01 | 516.23 | 8.97E-45 | Dhrsx | dehydrogenase/reductase (SDR family) X chromosome |
| ENSMUSG00000079742 | 8.85 | 461.94 | 1.17E-42 | NA | NA |
| ENSMUSG00000030768 | 8.84 | 459.21 | 1.71E-42 | Disp1 | dispatched homolog 1 (Drosophila) |
| ENSMUSG00000078180 | 8.73 | 424.26 | 3.14E-41 | NA | NA |
| ENSMUSG00000079685 | 8.61 | 390.06 | 5.80E-39 | Ulbp1 | UL16 binding protein 1 |
| ENSMUSG00000022156 | 8.61 | 389.40 | 1.92E-36 | Gzme | granzyme E |
| ENSMUSG00000047562 | 8.50 | 362.39 | 1.52E-44 | Mmp10 | matrix metallopeptidase 10 |
| ENSMUSG00000078739 | 8.31 | 317.47 | 8.73E-35 | NA | NA |
| ENSMUSG00000079673 | 8.30 | 315.87 | 4.78E-36 | NA | NA |
| ENSMUSG00000082171 | 8.09 | 271.67 | 1.62E-38 | NA | NA |
| ENSMUSG00000074829 | 8.06 | 267.77 | 1.83E-33 | NA | NA |
| ENSMUSG00000079834 | 8.05 | 265.36 | 2.69E-33 | Tmlhe | trimethyllysine hydroxylase, epsilon |
| ENSMUSG00000087977 | 7.71 | 208.72 | 2.85E-29 | NA | NA |
| ENSMUSG00000070366 | 7.57 | 190.20 | 3.64E-30 | Ppapdc1a | phosphatidic acid phosphatase type 2 domain containing 1A |
| ENSMUSG00000072258 | 7.53 | 184.83 | 5.14E-28 | Taf1a | TATA box binding protein (Tbp)-associated factor, RNA polymerase I, A |
| ENSMUSG00000062235 | 7.25 | 152.74 | 2.88E-24 | NA | NA |
| ENSMUSG00000079083 | 7.18 | 145.26 | 6.86E-25 | Jrkl | jerky homolog-like (mouse) |
| ENSMUSG00000061167 | 7.18 | 144.60 | 8.28E-25 | NA | NA |
| ENSMUSG00000079808 | 7.09 | 135.92 | 6.55E-24 | LOC102638047 | sp110 nuclear body protein-like |
| ENSMUSG00000079186 | 7.09 | 135.90 | 5.89E-31 | Gzmc | granzyme C |
| ENSMUSG00000069070 | 6.87 | 117.19 | 3.19E-22 | NA | NA |
| ENSMUSG00000079568 | 6.84 | 114.31 | 6.98E-21 | NA | NA |
| ENSMUSG00000087701 | 6.81 | 112.33 | 6.88E-32 | NA | NA |
| ENSMUSG00000068246 | 6.81 | 112.15 | 8.78E-48 | Apol9b | apolipoprotein L 9b |
| ENSMUSG00000009185 | 6.74 | 107.13 | 5.95E-50 | Ccl8 | chemokine (C-C motif) ligand 8 |
| ENSMUSG00000047323 | 6.72 | 105.09 | 6.66E-21 | NA | NA |
| ENSMUSG00000020581 | 6.71 | 105.01 | 1.40E-41 | Agr2 | anterior gradient 2 |
| ENSMUSG00000062038 | 6.65 | 100.59 | 1.36E-26 | NA | NA |
| ENSMUSG00000089022 | 6.64 | 99.78 | 1.39E-37 | NA | NA |
| ENSMUSG00000082029 | 6.46 | 88.08 | 3.70E-82 | NA | NA |
| ENSMUSG00000035352 | 6.44 | 86.74 | 3.10E-42 | Ccl12 | chemokine (C-C motif) ligand 12 |
| ENSMUSG00000035373 | 6.39 | 83.91 | 4.94E-36 | Ccl7 | chemokine (C-C motif) ligand 7 |
| ENSMUSG00000029675 | 6.27 | 77.12 | 6.88E-118 | Eln | elastin |
| ENSMUSG00000082348 | 6.23 | 75.29 | 3.37E-17 | NA | NA |
| ENSMUSG00000026343 | 6.23 | 75.23 | 2.37E-26 | Gpr39 | G protein-coupled receptor 39 |
| ENSMUSG00000049538 | 6.22 | 74.33 | 3.79E-22 | Adamts16 | a disintegrin-like and metallopeptidase (reprolysin type) with thrombospondin type 1 motif, 16 |
| ENSMUSG00000076281 | 6.19 | 72.99 | 5.02E-42 | NA | NA |
| ENSMUSG00000035557 | 6.16 | 71.70 | 4.03E-32 | Krt17 | keratin 17 |
| ENSMUSG00000068629 | 6.07 | 67.28 | 2.95E-16 | NA | NA |
| ENSMUSG00000085442 | 6.06 | 66.81 | 2.57E-44 | NA | NA |
| ENSMUSG00000089435 | 5.97 | 62.68 | 6.80E-18 | NA | NA |
| ENSMUSG00000050370 | 5.92 | 60.72 | 1.12E-24 | Ch25h | cholesterol 25-hydroxylase |
| ENSMUSG00000062783 | 5.87 | 58.61 | 6.46E-15 | NA | NA |
| ENSMUSG00000081885 | 5.86 | 58.22 | 8.05E-15 | NA | NA |
| ENSMUSG00000006403 | 5.85 | 57.87 | 1.43E-141 | Adamts4 | a disintegrin-like and metallopeptidase (reprolysin type) with thrombospondin type 1 motif, 4 |
| ENSMUSG00000034855 | 5.81 | 56.23 | 6.94E-60 | Cxcl10 | chemokine (C-X-C motif) ligand 10 |
| ENSMUSG00000060383 | 5.81 | 56.05 | 2.06E-14 | NA | NA |
| ENSMUSG00000075515 | 5.76 | 54.35 | 4.41E-31 | NA | NA |
| ENSMUSG00000090007 | 5.74 | 53.49 | 4.36E-24 | NA | NA |
| ENSMUSG00000074978 | 5.71 | 52.23 | 6.24E-31 | NA | NA |
| ENSMUSG00000062933 | 5.70 | 51.99 | 1.48E-50 | NA | NA |
| ENSMUSG00000081738 | 5.67 | 50.95 | 2.49E-24 | NA | NA |
| ENSMUSG00000087762 | 5.65 | 50.04 | 2.91E-16 | NA | NA |
| ENSMUSG00000082762 | 5.63 | 49.52 | 2.12E-57 | NA | NA |
| ENSMUSG00000082202 | 5.62 | 49.33 | 3.08E-13 | NA | NA |
| ENSMUSG00000083326 | 5.61 | 48.92 | 1.81E-26 | NA | NA |
| ENSMUSG00000083833 | 5.61 | 48.89 | 5.27E-32 | NA | NA |
| ENSMUSG00000048706 | 5.53 | 46.36 | 1.39E-41 | Lurap1l | leucine rich adaptor protein 1-like |
| ENSMUSG00000025930 | 5.52 | 45.74 | 2.09E-37 | Msc | musculin |
| ENSMUSG00000036960 | 5.50 | 45.29 | 2.10E-27 | Clca5 | chloride channel calcium activated 5 |
| ENSMUSG00000068196 | 5.48 | 44.67 | 2.64E-74 | Col8a1 | collagen, type VIII, alpha 1 |
| ENSMUSG00000031538 | 5.39 | 41.95 | 6.07E-78 | Plat | plasminogen activator, tissue |
| ENSMUSG00000057596 | 5.36 | 41.20 | 4.66E-57 | Trim30d | tripartite motif-containing 30D |
| ENSMUSG00000032487 | 5.20 | 36.81 | 4.06E-30 | Ptgs2 | prostaglandin-endoperoxide synthase 2 |
| ENSMUSG00000083911 | 5.19 | 36.56 | 1.75E-33 | NA | NA |
| ENSMUSG00000052504 | 5.19 | 36.46 | 1.33E-79 | Epha3 | Eph receptor A3 |
| ENSMUSG00000075707 | 5.18 | 36.36 | 2.36E-23 | Dio3 | deiodinase, iodothyronine type III |
| ENSMUSG00000057657 | 5.18 | 36.26 | 3.47E-23 | NA | NA |
| ENSMUSG00000078680 | 5.18 | 36.22 | 1.96E-10 | Mup10 | major urinary protein 10 |
| ENSMUSG00000089100 | 5.14 | 35.33 | 3.26E-11 | NA | NA |
| ENSMUSG00000059049 | 5.09 | 34.13 | 3.46E-37 | Frem1 | Fras1 related extracellular matrix protein 1 |
| ENSMUSG00000029236 | 5.07 | 33.59 | 4.93E-11 | Nmu | neuromedin U |
| ENSMUSG00000090110 | 5.07 | 33.49 | 2.29E-10 | NA | NA |
| ENSMUSG00000071269 | 5.06 | 33.25 | 1.24E-28 | NA | NA |
| ENSMUSG00000055110 | 5.05 | 33.18 | 2.54E-10 | A630012P03Rik | RIKEN cDNA A630012P03 gene |
| ENSMUSG00000064050 | 5.02 | 32.45 | 1.48E-27 | NA | NA |
| ENSMUSG00000053626 | 4.98 | 31.54 | 4.19E-33 | Tll1 | tolloid-like |
| ENSMUSG00000065592 | 4.97 | 31.30 | 1.75E-13 | NA | NA |
| ENSMUSG00000064354 | 4.95 | 30.87 | 1.29E-23 | COX2 | cytochrome c oxidase subunit II |
| ENSMUSG00000046182 | 4.94 | 30.80 | 3.06E-30 | Gsg1l | GSG1-like |
| ENSMUSG00000071562 | 4.93 | 30.56 | 1.27E-14 | Stfa1 | stefin A1 |
| ENSMUSG00000044576 | 4.93 | 30.40 | 5.24E-15 | Gareml | GRB2 associated, regulator of MAPK1-like |
| ENSMUSG00000074715 | 4.92 | 30.25 | 1.10E-09 | Ccl28 | chemokine (C-C motif) ligand 28 |
| ENSMUSG00000025922 | 4.91 | 30.01 | 4.38E-39 | NA | NA |
| ENSMUSG00000057244 | 4.89 | 29.61 | 1.37E-13 | NA | NA |
| ENSMUSG00000084178 | 4.88 | 29.48 | 5.92E-10 | NA | NA |
| ENSMUSG00000058050 | 4.88 | 29.45 | 4.34E-19 | NA | NA |
| ENSMUSG00000040829 | 4.86 | 29.00 | 1.47E-80 | Zmynd15 | zinc finger, MYND-type containing 15 |
| ENSMUSG00000062896 | 4.83 | 28.42 | 2.50E-38 | NA | NA |
| ENSMUSG00000025321 | 4.82 | 28.31 | 9.57E-27 | Itgb8 | integrin beta 8 |
| ENSMUSG00000071311 | 4.82 | 28.25 | 1.70E-11 | Gpr31b | G protein-coupled receptor 31, D17Leh66b region |
| ENSMUSG00000066443 | 4.82 | 28.23 | 2.53E-25 | NA | NA |
| ENSMUSG00000069014 | 4.80 | 27.89 | 8.09E-12 | NA | NA |
| ENSMUSG00000041700 | 4.79 | 27.58 | 2.73E-10 | Lhfpl1 | lipoma HMGIC fusion partner-like 1 |
| ENSMUSG00000074782 | 4.78 | 27.47 | 6.75E-40 | 4833422C13Rik | RIKEN cDNA 4833422C13 gene |
| ENSMUSG00000071361 | 4.77 | 27.22 | 1.29E-09 | Mcpt9 | mast cell protease 9 |
| ENSMUSG00000057346 | 4.75 | 26.91 | 5.63E-29 | Apol9a | apolipoprotein L 9a |
| ENSMUSG00000062611 | 4.75 | 26.84 | 2.11E-49 | NA | NA |
| ENSMUSG00000079218 | 4.74 | 26.71 | 6.71E-09 | NA | NA |
| ENSMUSG00000088277 | 4.74 | 26.67 | 6.29E-26 | NA | NA |
| ENSMUSG00000009214 | 4.74 | 26.64 | 8.03E-09 | Tmem8c | transmembrane protein 8C |
| ENSMUSG00000061833 | 4.73 | 26.53 | 1.10E-11 | NA | NA |
| ENSMUSG00000081834 | 4.73 | 26.49 | 4.09E-12 | NA | NA |
| ENSMUSG00000083852 | 4.73 | 26.49 | 2.74E-15 | NA | NA |
| ENSMUSG00000007888 | 4.71 | 26.17 | 8.26E-47 | Crlf1 | cytokine receptor-like factor 1 |
| ENSMUSG00000081176 | 4.68 | 25.58 | 9.12E-15 | NA | NA |
| ENSMUSG00000088805 | 4.67 | 25.54 | 1.11E-09 | NA | NA |
| ENSMUSG00000074157 | 4.67 | 25.49 | 1.33E-37 | NA | NA |
| ENSMUSG00000026166 | 4.67 | 25.41 | 1.14E-08 | Ccl20 | chemokine (C-C motif) ligand 20 |
| ENSMUSG00000066826 | 4.66 | 25.32 | 1.73E-09 | NA | NA |
| ENSMUSG00000075359 | 4.66 | 25.31 | 1.23E-08 | NA | NA |
| ENSMUSG00000037010 | 4.65 | 25.18 | 3.06E-79 | Apln | apelin |
| ENSMUSG00000026413 | 4.65 | 25.15 | 9.16E-20 | Pkp1 | plakophilin 1 |
| ENSMUSG00000068245 | 4.65 | 25.11 | 1.34E-45 | Phf11d | PHD finger protein 11D |
| ENSMUSG00000082705 | 4.65 | 25.07 | 5.74E-23 | NA | NA |
| ENSMUSG00000081671 | 4.64 | 24.96 | 1.23E-09 | NA | NA |
| ENSMUSG00000045055 | 4.64 | 24.91 | 2.41E-24 | NA | NA |
| ENSMUSG00000073802 | 4.64 | 24.86 | 3.19E-38 | Cdkn2b | cyclin-dependent kinase inhibitor 2B (p15, inhibits CDK4) |
| ENSMUSG00000082329 | 4.63 | 24.79 | 1.26E-09 | NA | NA |
| ENSMUSG00000041482 | 4.61 | 24.43 | 3.01E-37 | Piezo2 | piezo-type mechanosensitive ion channel component 2 |
| ENSMUSG00000063388 | 4.61 | 24.39 | 1.28E-29 | NA | NA |
| ENSMUSG00000068289 | 4.59 | 24.11 | 4.27E-10 | Cma2 | chymase 2, mast cell |
| ENSMUSG00000063011 | 4.58 | 23.91 | 2.50E-21 | Msln | mesothelin |
| ENSMUSG00000079191 | 4.57 | 23.83 | 2.87E-08 | NA | NA |
| ENSMUSG00000085164 | 4.57 | 23.83 | 5.14E-11 | NA | NA |
| ENSMUSG00000004098 | 4.56 | 23.56 | 4.54E-51 | Col5a3 | collagen, type V, alpha 3 |
| ENSMUSG00000089600 | 4.55 | 23.35 | 1.61E-08 | NA | NA |
| ENSMUSG00000078134 | 4.53 | 23.06 | 3.41E-11 | NA | NA |
| ENSMUSG00000073632 | 4.53 | 23.02 | 1.72E-10 | NA | NA |
| ENSMUSG00000030043 | 4.52 | 23.01 | 1.43E-28 | Tacr1 | tachykinin receptor 1 |
| ENSMUSG00000089328 | 4.52 | 22.87 | 1.92E-14 | NA | NA |
| ENSMUSG00000017724 | 4.51 | 22.82 | 1.41E-64 | Etv4 | ets variant gene 4 (E1A enhancer binding protein, E1AF) |
| ENSMUSG00000090231 | 4.51 | 22.81 | 1.33E-14 | Cfb | complement factor B |
| ENSMUSG00000022382 | 4.51 | 22.78 | 4.11E-38 | Wnt7b | wingless-related MMTV integration site 7B |
| ENSMUSG00000081992 | 4.49 | 22.40 | 4.77E-19 | NA | NA |
| ENSMUSG00000026475 | 4.47 | 22.18 | 6.79E-60 | Rgs16 | regulator of G-protein signaling 16 |
| ENSMUSG00000039385 | 4.47 | 22.10 | 3.04E-38 | Cdh6 | cadherin 6 |
| ENSMUSG00000079569 | 4.46 | 21.94 | 1.19E-07 | NA | NA |
| ENSMUSG00000051323 | 4.44 | 21.73 | 3.02E-29 | Pcdh19 | protocadherin 19 |
| ENSMUSG00000081684 | 4.42 | 21.41 | 1.46E-33 | NA | NA |
| ENSMUSG00000027800 | 4.41 | 21.27 | 1.74E-33 | Tm4sf1 | transmembrane 4 superfamily member 1 |
| ENSMUSG00000040229 | 4.40 | 21.08 | 2.86E-31 | Gpr34 | G protein-coupled receptor 34 |
| ENSMUSG00000030562 | 4.40 | 21.07 | 2.34E-22 | Nox4 | NADPH oxidase 4 |
| ENSMUSG00000065541 | 4.38 | 20.88 | 1.53E-14 | Mir24-2 | microRNA 24-2 |
| ENSMUSG00000024810 | 4.38 | 20.86 | 4.46E-23 | Il33 | interleukin 33 |
| ENSMUSG00000075224 | 4.38 | 20.83 | 1.20E-24 | Lrrc55 | leucine rich repeat containing 55 |
| ENSMUSG00000066809 | 4.36 | 20.54 | 2.73E-38 | NA | NA |
| ENSMUSG00000029664 | 4.35 | 20.34 | 1.12E-74 | Tfpi2 | tissue factor pathway inhibitor 2 |
| ENSMUSG00000025746 | 4.34 | 20.28 | 4.83E-18 | Il6 | interleukin 6 |
| ENSMUSG00000039209 | 4.34 | 20.28 | 1.74E-40 | Rpl39l | ribosomal protein L39-like |
| ENSMUSG00000063556 | 4.34 | 20.27 | 3.18E-11 | NA | NA |
| ENSMUSG00000073821 | 4.31 | 19.86 | 8.78E-14 | NA | NA |
| ENSMUSG00000042834 | 4.31 | 19.82 | 9.88E-57 | Nrep | neuronal regeneration related protein |
| ENSMUSG00000062758 | 4.30 | 19.75 | 7.41E-22 | NA | NA |
| ENSMUSG00000036699 | 4.30 | 19.74 | 8.24E-15 | Zcchc12 | zinc finger, CCHC domain containing 12 |
| ENSMUSG00000004371 | 4.30 | 19.69 | 1.37E-15 | Il11 | interleukin 11 |
| ENSMUSG00000050578 | 4.30 | 19.64 | 1.16E-38 | Mmp13 | matrix metallopeptidase 13 |
| ENSMUSG00000055795 | 4.30 | 19.63 | 5.30E-08 | NA | NA |
| ENSMUSG00000082632 | 4.29 | 19.59 | 5.53E-11 | NA | NA |
| ENSMUSG00000080896 | 4.29 | 19.56 | 1.67E-49 | NA | NA |
| ENSMUSG00000044303 | 4.26 | 19.18 | 2.91E-36 | Cdkn2a | cyclin-dependent kinase inhibitor 2A |
| ENSMUSG00000052353 | 4.25 | 19.07 | 2.01E-29 | 9930013L23Rik | RIKEN cDNA 9930013L23 gene |
| ENSMUSG00000056904 | 4.25 | 19.04 | 1.61E-40 | NA | NA |
| ENSMUSG00000029348 | 4.24 | 18.96 | 3.99E-30 | Asphd2 | aspartate beta-hydroxylase domain containing 2 |
| ENSMUSG00000023279 | 4.24 | 18.93 | 7.91E-13 | Bmp15 | bone morphogenetic protein 15 |
| ENSMUSG00000029377 | 4.24 | 18.87 | 4.47E-09 | Ereg | epiregulin |
| ENSMUSG00000027204 | 4.22 | 18.70 | 5.39E-81 | Fbn1 | fibrillin 1 |
| ENSMUSG00000072324 | 4.22 | 18.68 | 9.40E-08 | NA | NA |
| ENSMUSG00000044703 | 4.22 | 18.65 | 2.88E-36 | Phf11a | PHD finger protein 11A |
| ENSMUSG00000029380 | 4.21 | 18.56 | 5.02E-16 | Cxcl1 | chemokine (C-X-C motif) ligand 1 |
| ENSMUSG00000047261 | 4.21 | 18.56 | 2.00E-15 | Gap43 | growth associated protein 43 |
| ENSMUSG00000073062 | 4.20 | 18.41 | 2.98E-19 | Zxdb | zinc finger, X-linked, duplicated B |
| ENSMUSG00000022367 | 4.20 | 18.39 | 8.26E-53 | Has2 | hyaluronan synthase 2 |
| ENSMUSG00000082127 | 4.19 | 18.25 | 7.45E-07 | NA | NA |
| ENSMUSG00000088246 | 4.18 | 18.07 | 1.55E-07 | NA | NA |
| ENSMUSG00000089249 | 4.17 | 17.98 | 5.22E-07 | NA | NA |
| ENSMUSG00000062488 | 4.16 | 17.82 | 8.64E-35 | I830012O16Rik | RIKEN cDNA I830012O16 gene |
| ENSMUSG00000069106 | 4.15 | 17.80 | 1.53E-08 | NA | NA |
| ENSMUSG00000060183 | 4.15 | 17.80 | 1.45E-08 | Cxcl11 | chemokine (C-X-C motif) ligand 11 |
| ENSMUSG00000073229 | 4.15 | 17.78 | 1.63E-08 | NA | NA |
| ENSMUSG00000054293 | 4.14 | 17.65 | 6.12E-34 | A630033H20Rik | RIKEN cDNA A630033H20 gene |
| ENSMUSG00000032135 | 4.13 | 17.45 | 7.14E-71 | Mcam | melanoma cell adhesion molecule |
| ENSMUSG00000004892 | 4.12 | 17.43 | 1.43E-25 | Bcan | brevican |
| ENSMUSG00000015652 | 4.12 | 17.38 | 1.08E-25 | Steap1 | six transmembrane epithelial antigen of the prostate 1 |
| ENSMUSG00000054072 | 4.11 | 17.22 | 6.58E-45 | Iigp1 | interferon inducible GTPase 1 |
| ENSMUSG00000045871 | 4.11 | 17.21 | 8.80E-46 | Slitrk6 | SLIT and NTRK-like family, member 6 |
| ENSMUSG00000048040 | 4.10 | 17.15 | 5.01E-20 | NA | NA |
| ENSMUSG00000086905 | 4.10 | 17.12 | 3.21E-09 | NA | NA |
| ENSMUSG00000040284 | 4.09 | 17.08 | 1.00E-06 | Gzmg | granzyme G |
| ENSMUSG00000001131 | 4.09 | 16.98 | 1.23E-38 | Timp1 | tissue inhibitor of metalloproteinase 1 |
| ENSMUSG00000026548 | 4.07 | 16.85 | 6.47E-41 | Slamf9 | SLAM family member 9 |
| ENSMUSG00000026253 | 4.07 | 16.84 | 2.22E-07 | Chrng | cholinergic receptor, nicotinic, gamma polypeptide |
| ENSMUSG00000028031 | 4.07 | 16.83 | 1.32E-10 | Dkk2 | dickkopf homolog 2 (Xenopus laevis) |
| ENSMUSG00000084129 | 4.07 | 16.80 | 2.48E-07 | NA | NA |
| ENSMUSG00000074896 | 4.07 | 16.78 | 1.75E-34 | Ifit3 | interferon-induced protein with tetratricopeptide repeats 3 |
| ENSMUSG00000052316 | 4.06 | 16.66 | 1.03E-42 | Lrrc15 | leucine rich repeat containing 15 |
| ENSMUSG00000071052 | 4.06 | 16.66 | 1.67E-16 | NA | NA |
| ENSMUSG00000035131 | 4.04 | 16.43 | 1.60E-21 | Brinp3 | bone morphogenetic protein/retinoic acid inducible neural specific 3 |
| ENSMUSG00000061402 | 4.04 | 16.43 | 3.68E-16 | NA | NA |
| ENSMUSG00000073491 | 4.04 | 16.43 | 1.01E-09 | Pydc4 | pyrin domain containing 4 |
| ENSMUSG00000052736 | 4.04 | 16.42 | 6.38E-07 | Klrc2 | killer cell lectin-like receptor subfamily C, member 2 |
| ENSMUSG00000023905 | 4.04 | 16.40 | 4.49E-42 | Tnfrsf12a | tumor necrosis factor receptor superfamily, member 12a |
| ENSMUSG00000066530 | 4.03 | 16.39 | 1.92E-08 | NA | NA |
| ENSMUSG00000060096 | 4.03 | 16.38 | 5.84E-16 | Amd-ps3 | S-adenosylmethionine decarboxylase, pseudogene 3 |
| ENSMUSG00000004961 | 4.03 | 16.33 | 1.55E-20 | Syt5 | synaptotagmin V |
| ENSMUSG00000075602 | 4.02 | 16.23 | 1.19E-27 | Ly6a | lymphocyte antigen 6 complex, locus A |
| ENSMUSG00000028362 | 4.02 | 16.18 | 6.63E-26 | Tnfsf8 | tumor necrosis factor (ligand) superfamily, member 8 |
| ENSMUSG00000080715 | 4.01 | 16.15 | 1.39E-11 | NA | NA |
| ENSMUSG00000031639 | 4.01 | 16.15 | 2.34E-73 | Tlr3 | toll-like receptor 3 |
| ENSMUSG00000051855 | 4.00 | 16.02 | 1.22E-59 | Mest | mesoderm specific transcript |
| ENSMUSG00000038775 | 4.00 | 16.02 | 2.01E-22 | Vill | villin-like |
| ENSMUSG00000065487 | 3.99 | 15.90 | 3.46E-10 | NA | NA |
| ENSMUSG00000080763 | 3.98 | 15.81 | 3.20E-09 | NA | NA |
| ENSMUSG00000067147 | 3.97 | 15.69 | 2.48E-13 | NA | NA |
| ENSMUSG00000076442 | 3.97 | 15.64 | 1.25E-41 | NA | NA |
| ENSMUSG00000065605 | 3.96 | 15.59 | 4.28E-06 | NA | NA |
| ENSMUSG00000032057 | 3.96 | 15.52 | 1.05E-07 | 4833427G06Rik | RIKEN cDNA 4833427G06 gene |
| ENSMUSG00000066632 | 3.95 | 15.47 | 9.45E-11 | NA | NA |
| ENSMUSG00000040703 | 3.95 | 15.43 | 2.95E-26 | Cyp2s1 | cytochrome P450, family 2, subfamily s, polypeptide 1 |
| ENSMUSG00000071040 | 3.95 | 15.41 | 4.58E-06 | NA | NA |
| ENSMUSG00000022227 | 3.94 | 15.36 | 1.64E-07 | Mcpt1 | mast cell protease 1 |
| ENSMUSG00000034457 | 3.92 | 15.11 | 1.63E-18 | Eda2r | ectodysplasin A2 receptor |
| ENSMUSG00000074000 | 3.91 | 15.05 | 3.24E-07 | NA | NA |
| ENSMUSG00000039146 | 3.91 | 15.03 | 1.75E-48 | Ifi44l | interferon-induced protein 44 like |
| ENSMUSG00000065226 | 3.90 | 14.96 | 2.15E-14 | NA | NA |
| ENSMUSG00000023945 | 3.90 | 14.94 | 3.31E-06 | Slc5a7 | solute carrier family 5 (choline transporter), member 7 |
| ENSMUSG00000089317 | 3.90 | 14.90 | 2.17E-08 | Snord123 | small nucleolar RNA, C/D box 123 |
| ENSMUSG00000082399 | 3.90 | 14.88 | 5.20E-08 | NA | NA |
| ENSMUSG00000071003 | 3.88 | 14.77 | 4.06E-07 | NA | NA |
| ENSMUSG00000078248 | 3.88 | 14.74 | 7.31E-06 | NA | NA |
| ENSMUSG00000060795 | 3.88 | 14.69 | 4.47E-07 | NA | NA |
| ENSMUSG00000028698 | 3.87 | 14.66 | 1.96E-56 | Pik3r3 | phosphatidylinositol 3 kinase, regulatory subunit, polypeptide 3 (p55) |
| ENSMUSG00000068606 | 3.87 | 14.57 | 5.13E-20 | Gm4841 | predicted gene 4841 |
| ENSMUSG00000020592 | 3.86 | 14.51 | 7.20E-78 | Sdc1 | syndecan 1 |
| ENSMUSG00000088032 | 3.85 | 14.40 | 1.31E-06 | NA | NA |
| ENSMUSG00000026043 | 3.85 | 14.39 | 1.18E-42 | Col3a1 | collagen, type III, alpha 1 |
| ENSMUSG00000089646 | 3.84 | 14.33 | 3.58E-08 | NA | NA |
| ENSMUSG00000089988 | 3.84 | 14.31 | 1.06E-07 | NA | NA |
| ENSMUSG00000079303 | 3.83 | 14.26 | 9.76E-06 | NA | NA |
| ENSMUSG00000074676 | 3.83 | 14.22 | 4.38E-39 | Foxs1 | forkhead box S1 |
| ENSMUSG00000074971 | 3.83 | 14.20 | 5.18E-47 | Fibin | fin bud initiation factor homolog (zebrafish) |
| ENSMUSG00000027670 | 3.83 | 14.18 | 6.00E-38 | Ocstamp | osteoclast stimulatory transmembrane protein |
| ENSMUSG00000035165 | 3.82 | 14.15 | 7.86E-43 | Kcne3 | potassium voltage-gated channel, Isk-related subfamily, gene 3 |
| ENSMUSG00000064493 | 3.82 | 14.10 | 2.47E-12 | NA | NA |
| ENSMUSG00000021994 | 3.81 | 14.06 | 3.35E-80 | Wnt5a | wingless-related MMTV integration site 5A |
| ENSMUSG00000039405 | 3.80 | 13.97 | 4.32E-51 | Prss23 | protease, serine 23 |
| ENSMUSG00000054162 | 3.80 | 13.94 | 8.63E-06 | Spock3 | sparc/osteonectin, cwcv and kazal-like domains proteoglycan 3 |
| ENSMUSG00000081772 | 3.80 | 13.94 | 3.43E-06 | NA | NA |
| ENSMUSG00000048126 | 3.79 | 13.82 | 3.68E-71 | Col6a3 | collagen, type VI, alpha 3 |
| ENSMUSG00000041605 | 3.79 | 13.82 | 2.54E-09 | 5730559C18Rik | RIKEN cDNA 5730559C18 gene |
| ENSMUSG00000087488 | 3.79 | 13.80 | 6.01E-06 | NA | NA |
| ENSMUSG00000001168 | 3.78 | 13.77 | 6.91E-07 | Oas1h | 2'-5' oligoadenylate synthetase 1H |
| ENSMUSG00000078673 | 3.78 | 13.74 | 9.93E-08 | NA | NA |
| ENSMUSG00000062070 | 3.78 | 13.73 | 4.50E-45 | NA | NA |
| ENSMUSG00000066315 | 3.78 | 13.71 | 2.76E-53 | NA | NA |
| ENSMUSG00000000058 | 3.77 | 13.69 | 5.33E-37 | Cav2 | caveolin 2 |
| ENSMUSG00000084277 | 3.77 | 13.66 | 8.75E-10 | NA | NA |
| ENSMUSG00000066270 | 3.77 | 13.62 | 7.32E-06 | NA | NA |
| ENSMUSG00000029121 | 3.76 | 13.57 | 1.24E-13 | Crmp1 | collapsin response mediator protein 1 |
| ENSMUSG00000084067 | 3.76 | 13.55 | 2.34E-35 | NA | NA |
| ENSMUSG00000026459 | 3.76 | 13.55 | 2.49E-10 | Myog | myogenin |
| ENSMUSG00000089954 | 3.76 | 13.54 | 1.62E-05 | NA | NA |
| ENSMUSG00000083596 | 3.76 | 13.51 | 1.73E-11 | NA | NA |
| ENSMUSG00000024672 | 3.75 | 13.48 | 7.33E-27 | Ms4a7 | membrane-spanning 4-domains, subfamily A, member 7 |
| ENSMUSG00000079794 | 3.73 | 13.24 | 2.77E-05 | NA | NA |
| ENSMUSG00000060887 | 3.72 | 13.21 | 1.48E-10 | NA | NA |
| ENSMUSG00000001435 | 3.72 | 13.19 | 2.40E-53 | Col18a1 | collagen, type XVIII, alpha 1 |
| ENSMUSG00000027514 | 3.71 | 13.05 | 1.11E-47 | Zbp1 | Z-DNA binding protein 1 |
| ENSMUSG00000083626 | 3.70 | 12.99 | 2.05E-54 | NA | NA |
| ENSMUSG00000031239 | 3.70 | 12.97 | 5.20E-44 | Itm2a | integral membrane protein 2A |
| ENSMUSG00000034795 | 3.69 | 12.95 | 1.03E-15 | Ccdc122 | coiled-coil domain containing 122 |
| ENSMUSG00000073295 | 3.69 | 12.95 | 5.08E-06 | NA | NA |
| ENSMUSG00000059179 | 3.69 | 12.95 | 2.02E-10 | NA | NA |
| ENSMUSG00000003657 | 3.69 | 12.92 | 5.81E-09 | Calb2 | calbindin 2 |
| ENSMUSG00000036912 | 3.69 | 12.89 | 9.24E-12 | Piwil4 | piwi-like RNA-mediated gene silencing 4 |
| ENSMUSG00000087143 | 3.69 | 12.87 | 6.78E-11 | NA | NA |
| ENSMUSG00000070032 | 3.68 | 12.82 | 2.82E-05 | NA | NA |
| ENSMUSG00000079963 | 3.68 | 12.79 | 1.66E-05 | NA | NA |
| ENSMUSG00000073397 | 3.68 | 12.78 | 2.73E-05 | NA | NA |
| ENSMUSG00000061762 | 3.67 | 12.76 | 5.85E-06 | Tac1 | tachykinin 1 |
| ENSMUSG00000076137 | 3.67 | 12.73 | 1.02E-05 | NA | NA |
| ENSMUSG00000066116 | 3.66 | 12.68 | 3.05E-05 | NA | NA |
| ENSMUSG00000050288 | 3.66 | 12.65 | 6.47E-16 | Fzd2 | frizzled homolog 2 (Drosophila) |
| ENSMUSG00000084328 | 3.66 | 12.62 | 3.03E-05 | NA | NA |
| ENSMUSG00000023341 | 3.65 | 12.58 | 1.18E-40 | Mx2 | myxovirus (influenza virus) resistance 2 |
| ENSMUSG00000079227 | 3.65 | 12.56 | 1.76E-56 | Ccr5 | chemokine (C-C motif) receptor 5 |
| ENSMUSG00000066861 | 3.65 | 12.54 | 1.77E-37 | Oas1g | 2'-5' oligoadenylate synthetase 1G |
| ENSMUSG00000019789 | 3.65 | 12.54 | 1.30E-31 | Hey2 | hairy/enhancer-of-split related with YRPW motif 2 |
| ENSMUSG00000079224 | 3.65 | 12.53 | 3.91E-06 | NA | NA |
| ENSMUSG00000058542 | 3.64 | 12.50 | 1.25E-05 | NA | NA |
| ENSMUSG00000062358 | 3.64 | 12.50 | 1.20E-10 | NA | NA |
| ENSMUSG00000026535 | 3.64 | 12.50 | 1.41E-112 | NA | NA |
| ENSMUSG00000015852 | 3.64 | 12.45 | 1.28E-16 | Fcrls | Fc receptor-like S, scavenger receptor |
| ENSMUSG00000070469 | 3.63 | 12.42 | 1.79E-25 | Adamtsl3 | ADAMTS-like 3 |
| ENSMUSG00000082729 | 3.63 | 12.41 | 1.26E-20 | NA | NA |
| ENSMUSG00000073640 | 3.63 | 12.37 | 3.80E-05 | NA | NA |
| ENSMUSG00000010175 | 3.63 | 12.37 | 6.20E-11 | Prox1 | prospero homeobox 1 |
| ENSMUSG00000058914 | 3.63 | 12.34 | 3.13E-07 | C1qtnf3 | C1q and tumor necrosis factor related protein 3 |
| ENSMUSG00000041886 | 3.62 | 12.33 | 3.71E-05 | Macc1 | metastasis associated in colon cancer 1 |
| ENSMUSG00000033882 | 3.62 | 12.26 | 7.47E-08 | Rbm46 | RNA binding motif protein 46 |
| ENSMUSG00000059511 | 3.61 | 12.22 | 1.54E-07 | NA | NA |
| ENSMUSG00000052430 | 3.61 | 12.22 | 1.29E-10 | Bmpr1b | bone morphogenetic protein receptor, type 1B |
| ENSMUSG00000085573 | 3.61 | 12.20 | 6.54E-06 | NA | NA |
| ENSMUSG00000007805 | 3.61 | 12.17 | 4.06E-07 | Twist2 | twist basic helix-loop-helix transcription factor 2 |
| ENSMUSG00000089948 | 3.60 | 12.16 | 4.35E-05 | NA | NA |
| ENSMUSG00000034810 | 3.60 | 12.15 | 6.19E-24 | Scn7a | sodium channel, voltage-gated, type VII, alpha |
| ENSMUSG00000032085 | 3.60 | 12.11 | 1.62E-62 | Tagln | transgelin |
| ENSMUSG00000059634 | 3.60 | 12.09 | 1.10E-09 | NA | NA |
| ENSMUSG00000022015 | 3.58 | 11.99 | 7.11E-44 | Tnfsf11 | tumor necrosis factor (ligand) superfamily, member 11 |
| ENSMUSG00000026494 | 3.58 | 11.96 | 3.69E-21 | Kif26b | kinesin family member 26B |
| ENSMUSG00000056481 | 3.58 | 11.95 | 1.57E-20 | Cd248 | CD248 antigen, endosialin |
| ENSMUSG00000063506 | 3.57 | 11.90 | 1.25E-17 | Arhgap22 | Rho GTPase activating protein 22 |
| ENSMUSG00000042734 | 3.57 | 11.88 | 1.17E-39 | Ttc9 | tetratricopeptide repeat domain 9 |
| ENSMUSG00000081239 | 3.57 | 11.87 | 5.42E-30 | NA | NA |
| ENSMUSG00000082241 | 3.57 | 11.84 | 1.03E-06 | NA | NA |
| ENSMUSG00000014932 | 3.57 | 11.84 | 9.47E-60 | Yes1 | Yamaguchi sarcoma viral (v-yes) oncogene homolog 1 |
| ENSMUSG00000054196 | 3.56 | 11.83 | 1.29E-39 | Cthrc1 | collagen triple helix repeat containing 1 |
| ENSMUSG00000066543 | 3.55 | 11.75 | 3.60E-14 | NA | NA |
| ENSMUSG00000080824 | 3.55 | 11.73 | 1.20E-33 | NA | NA |
| ENSMUSG00000071722 | 3.55 | 11.73 | 6.38E-13 | Spin4 | spindlin family, member 4 |
| ENSMUSG00000038702 | 3.55 | 11.72 | 3.41E-49 | Dsel | dermatan sulfate epimerase-like |
| ENSMUSG00000037225 | 3.55 | 11.72 | 9.82E-11 | Fgf2 | fibroblast growth factor 2 |
| ENSMUSG00000019836 | 3.55 | 11.70 | 5.94E-05 | NA | NA |
| ENSMUSG00000044689 | 3.55 | 11.68 | 1.05E-05 | Gm13749 | predicted gene 13749 |
| ENSMUSG00000086538 | 3.54 | 11.62 | 6.06E-23 | NA | NA |
| ENSMUSG00000047678 | 3.54 | 11.61 | 1.85E-06 | Gpr82 | G protein-coupled receptor 82 |
| ENSMUSG00000058625 | 3.53 | 11.56 | 4.80E-19 | NA | NA |
| ENSMUSG00000079192 | 3.53 | 11.55 | 6.61E-05 | NA | NA |
| ENSMUSG00000057135 | 3.53 | 11.54 | 2.51E-23 | Scimp | SLP adaptor and CSK interacting membrane protein |
| ENSMUSG00000069962 | 3.52 | 11.50 | 5.80E-06 | NA | NA |
| ENSMUSG00000085135 | 3.52 | 11.48 | 7.12E-07 | NA | NA |
| ENSMUSG00000047497 | 3.52 | 11.48 | 6.99E-17 | Adamts12 | a disintegrin-like and metallopeptidase (reprolysin type) with thrombospondin type 1 motif, 12 |
| ENSMUSG00000062380 | 3.52 | 11.44 | 1.08E-23 | Tubb3 | tubulin, beta 3 class III |
| ENSMUSG00000079831 | 3.51 | 11.42 | 7.39E-05 | NA | NA |
| ENSMUSG00000064612 | 3.51 | 11.41 | 7.19E-05 | NA | NA |
| ENSMUSG00000078142 | 3.51 | 11.36 | 8.27E-05 | NA | NA |
| ENSMUSG00000000126 | 3.51 | 11.36 | 1.79E-19 | Wnt9a | wingless-type MMTV integration site 9A |
| ENSMUSG00000018920 | 3.50 | 11.34 | 6.56E-40 | Cxcl16 | chemokine (C-X-C motif) ligand 16 |
| ENSMUSG00000073555 | 3.50 | 11.31 | 4.11E-35 | Gm4951 | predicted gene 4951 |
| ENSMUSG00000034459 | 3.50 | 11.30 | 1.34E-42 | Ifit1 | interferon-induced protein with tetratricopeptide repeats 1 |
| ENSMUSG00000001493 | 3.50 | 11.30 | 3.77E-20 | Meox1 | mesenchyme homeobox 1 |
| ENSMUSG00000020186 | 3.50 | 11.29 | 6.00E-65 | Csrp2 | cysteine and glycine-rich protein 2 |
| ENSMUSG00000074516 | 3.49 | 11.24 | 3.03E-09 | LOC100503055 | 60S ribosomal protein L29-like |
| ENSMUSG00000019890 | 3.49 | 11.20 | 1.08E-06 | Nts | neurotensin |
| ENSMUSG00000079339 | 3.48 | 11.18 | 4.01E-20 | Gm14446 | predicted gene 14446 |
| ENSMUSG00000029092 | 3.48 | 11.17 | 9.33E-05 | NA | NA |
| ENSMUSG00000067833 | 3.48 | 11.16 | 3.43E-10 | NA | NA |
| ENSMUSG00000082743 | 3.48 | 11.16 | 1.46E-09 | NA | NA |
| ENSMUSG00000034394 | 3.48 | 11.12 | 1.42E-28 | Lif | leukemia inhibitory factor |
| ENSMUSG00000048450 | 3.47 | 11.11 | 4.42E-28 | Msx1 | msh homeobox 1 |
| ENSMUSG00000039519 | 3.47 | 11.09 | 2.93E-13 | Cyp7b1 | cytochrome P450, family 7, subfamily b, polypeptide 1 |
| ENSMUSG00000062184 | 3.47 | 11.06 | 1.29E-09 | Hs6st2 | heparan sulfate 6-O-sulfotransferase 2 |
| ENSMUSG00000022863 | 3.47 | 11.05 | 1.25E-08 | NA | NA |
| ENSMUSG00000020000 | 3.46 | 11.04 | 4.83E-14 | Moxd1 | monooxygenase, DBH-like 1 |
| ENSMUSG00000080980 | 3.46 | 11.01 | 4.12E-05 | NA | NA |
| ENSMUSG00000084364 | 3.46 | 10.98 | 1.01E-04 | NA | NA |
| ENSMUSG00000000386 | 3.46 | 10.98 | 1.05E-35 | Mx1 | myxovirus (influenza virus) resistance 1 |
| ENSMUSG00000075588 | 3.46 | 10.97 | 3.12E-08 | Hoxb2 | homeobox B2 |
| ENSMUSG00000031880 | 3.45 | 10.96 | 1.67E-28 | Rrad | Ras-related associated with diabetes |
| ENSMUSG00000078922 | 3.45 | 10.96 | 1.93E-26 | NA | NA |
| ENSMUSG00000081058 | 3.44 | 10.86 | 1.08E-04 | NA | NA |
| ENSMUSG00000060586 | 3.44 | 10.83 | 7.14E-89 | H2-Eb1 | histocompatibility 2, class II antigen E beta |
| ENSMUSG00000024593 | 3.44 | 10.83 | 1.77E-38 | Megf10 | multiple EGF-like-domains 10 |
| ENSMUSG00000053461 | 3.44 | 10.82 | 1.11E-04 | Hhipl2 | hedgehog interacting protein-like 2 |
| ENSMUSG00000059835 | 3.43 | 10.80 | 3.23E-08 | NA | NA |
| ENSMUSG00000036322 | 3.43 | 10.80 | 5.95E-60 | NA | NA |
| ENSMUSG00000034488 | 3.43 | 10.79 | 2.99E-27 | Edil3 | EGF-like repeats and discoidin I-like domains 3 |
| ENSMUSG00000010601 | 3.43 | 10.75 | 1.25E-05 | Apol7a | apolipoprotein L 7a |
| ENSMUSG00000053318 | 3.41 | 10.66 | 1.38E-23 | Slamf8 | SLAM family member 8 |
| ENSMUSG00000036594 | 3.41 | 10.64 | 1.13E-52 | H2-Aa | histocompatibility 2, class II antigen A, alpha |
| ENSMUSG00000046561 | 3.41 | 10.62 | 5.22E-13 | Arsj | arylsulfatase J |
| ENSMUSG00000020908 | 3.40 | 10.58 | 1.93E-10 | Myh3 | myosin, heavy polypeptide 3, skeletal muscle, embryonic |
| ENSMUSG00000059249 | 3.40 | 10.53 | 2.82E-45 | NA | NA |
| ENSMUSG00000015653 | 3.40 | 10.53 | 1.02E-63 | Steap2 | six transmembrane epithelial antigen of prostate 2 |
| ENSMUSG00000029659 | 3.40 | 10.52 | 1.12E-20 | Medag | mesenteric estrogen dependent adipogenesis |
| ENSMUSG00000087284 | 3.39 | 10.46 | 1.59E-14 | NA | NA |
| ENSMUSG00000075724 | 3.38 | 10.44 | 1.46E-04 | NA | NA |
| ENSMUSG00000082099 | 3.38 | 10.43 | 5.42E-13 | NA | NA |
| ENSMUSG00000087974 | 3.38 | 10.41 | 1.25E-06 | NA | NA |
| ENSMUSG00000030560 | 3.37 | 10.37 | 4.31E-150 | Ctsc | cathepsin C |
| ENSMUSG00000061356 | 3.37 | 10.35 | 1.06E-07 | Nuggc | nuclear GTPase, germinal center associated |
| ENSMUSG00000022661 | 3.37 | 10.34 | 8.66E-32 | Cd200 | CD200 antigen |
| ENSMUSG00000045545 | 3.37 | 10.34 | 3.04E-05 | Krt14 | keratin 14 |
| ENSMUSG00000074689 | 3.37 | 10.34 | 7.81E-05 | NA | NA |
| ENSMUSG00000041347 | 3.37 | 10.34 | 9.09E-07 | Bdkrb1 | bradykinin receptor, beta 1 |
| ENSMUSG00000037843 | 3.37 | 10.34 | 1.41E-06 | Vstm2l | V-set and transmembrane domain containing 2-like |
| ENSMUSG00000019779 | 3.37 | 10.32 | 6.96E-27 | Frk | fyn-related kinase |
| ENSMUSG00000081810 | 3.37 | 10.32 | 5.45E-10 | NA | NA |
| ENSMUSG00000080998 | 3.37 | 10.32 | 3.69E-05 | NA | NA |
| ENSMUSG00000040133 | 3.36 | 10.29 | 2.52E-28 | Gpr176 | G protein-coupled receptor 176 |
| ENSMUSG00000079105 | 3.36 | 10.27 | 3.38E-08 | C7 | complement component 7 |
| ENSMUSG00000073234 | 3.36 | 10.27 | 3.21E-05 | Gm8773 | predicted gene 8773 |
| ENSMUSG00000021070 | 3.34 | 10.15 | 3.48E-14 | Bdkrb2 | bradykinin receptor, beta 2 |
| ENSMUSG00000088901 | 3.34 | 10.12 | 1.87E-04 | NA | NA |
| ENSMUSG00000049420 | 3.34 | 10.11 | 5.50E-12 | Tmem200a | transmembrane protein 200A |
| ENSMUSG00000038156 | 3.34 | 10.09 | 9.86E-52 | Spon1 | spondin 1, (f-spondin) extracellular matrix protein |
| ENSMUSG00000049872 | 3.33 | 10.07 | 9.72E-24 | Fam26e | family with sequence similarity 26, member E |
| ENSMUSG00000024883 | 3.33 | 10.06 | 1.20E-33 | Rin1 | Ras and Rab interactor 1 |
| ENSMUSG00000032796 | 3.33 | 10.04 | 3.38E-34 | Lama1 | laminin, alpha 1 |
| ENSMUSG00000028327 | 3.33 | 10.03 | 6.62E-09 | 1300002K09Rik | RIKEN cDNA 1300002K09 gene |
| ENSMUSG00000021614 | 3.33 | 10.03 | 6.94E-105 | Vcan | versican |
| ENSMUSG00000028037 | 3.33 | 10.03 | 2.06E-28 | Ifi44 | interferon-induced protein 44 |
| ENSMUSG00000085620 | 3.33 | 10.03 | 2.05E-04 | NA | NA |
| ENSMUSG00000058873 | 3.32 | 10.00 | 2.10E-04 | NA | NA |
| ENSMUSG00000040473 | 3.32 | 10.00 | 4.98E-17 | A330021E22Rik | RIKEN cDNA A330021E22 gene |
| ENSMUSG00000017652 | 3.32 | 9.99 | 1.05E-25 | Cd40 | CD40 antigen |
| ENSMUSG00000084104 | 3.32 | 9.98 | 5.14E-49 | NA | NA |
| ENSMUSG00000073489 | 3.32 | 9.96 | 1.92E-40 | Ifi204 | interferon activated gene 204 |
| ENSMUSG00000031502 | 3.31 | 9.94 | 3.47E-61 | Col4a1 | collagen, type IV, alpha 1 |
| ENSMUSG00000033849 | 3.31 | 9.93 | 5.07E-20 | B3galt2 | UDP-Gal:betaGlcNAc beta 1,3-galactosyltransferase, polypeptide 2 |
| ENSMUSG00000028270 | 3.31 | 9.92 | 1.23E-24 | Gbp2 | guanylate binding protein 2 |
| ENSMUSG00000084166 | 3.31 | 9.90 | 1.56E-10 | NA | NA |
| ENSMUSG00000088272 | 3.31 | 9.89 | 1.50E-15 | NA | NA |
| ENSMUSG00000032332 | 3.30 | 9.84 | 3.75E-34 | Col12a1 | collagen, type XII, alpha 1 |
| ENSMUSG00000089812 | 3.30 | 9.83 | 1.01E-12 | NA | NA |
| ENSMUSG00000032511 | 3.29 | 9.80 | 4.37E-10 | Scn5a | sodium channel, voltage-gated, type V, alpha |
| ENSMUSG00000027656 | 3.29 | 9.75 | 4.12E-46 | Wisp2 | WNT1 inducible signaling pathway protein 2 |
| ENSMUSG00000067285 | 3.28 | 9.74 | 2.50E-04 | Gm16223 | predicted gene 16223 |
| ENSMUSG00000064770 | 3.28 | 9.73 | 2.55E-04 | NA | NA |
| ENSMUSG00000035566 | 3.28 | 9.70 | 2.57E-12 | Pcdh17 | protocadherin 17 |
| ENSMUSG00000081562 | 3.28 | 9.69 | 3.20E-15 | NA | NA |
| ENSMUSG00000019846 | 3.28 | 9.68 | 3.81E-88 | Lama4 | laminin, alpha 4 |
| ENSMUSG00000069171 | 3.27 | 9.67 | 1.14E-21 | Nr2f1 | nuclear receptor subfamily 2, group F, member 1 |
| ENSMUSG00000085040 | 3.27 | 9.63 | 1.13E-04 | NA | NA |
| ENSMUSG00000018919 | 3.27 | 9.62 | 6.28E-06 | Tm4sf5 | transmembrane 4 superfamily member 5 |
| ENSMUSG00000053647 | 3.27 | 9.62 | 8.49E-62 | Gper1 | G protein-coupled estrogen receptor 1 |
| ENSMUSG00000062661 | 3.26 | 9.58 | 1.44E-30 | Ncs1 | neuronal calcium sensor 1 |
| ENSMUSG00000025887 | 3.26 | 9.57 | 1.87E-33 | Casp12 | caspase 12 |
| ENSMUSG00000026109 | 3.26 | 9.57 | 3.63E-06 | Tmeff2 | transmembrane protein with EGF-like and two follistatin-like domains 2 |
| ENSMUSG00000067657 | 3.26 | 9.56 | 7.97E-11 | NA | NA |
| ENSMUSG00000086982 | 3.25 | 9.55 | 1.65E-04 | NA | NA |
| ENSMUSG00000046718 | 3.25 | 9.51 | 4.33E-29 | Bst2 | bone marrow stromal cell antigen 2 |
| ENSMUSG00000022303 | 3.24 | 9.47 | 5.85E-19 | Dcstamp | dentrocyte expressed seven transmembrane protein |
| ENSMUSG00000000355 | 3.23 | 9.40 | 4.87E-30 | Mcts1 | malignant T cell amplified sequence 1 |
| ENSMUSG00000086755 | 3.23 | 9.39 | 2.05E-05 | NA | NA |
| ENSMUSG00000073406 | 3.22 | 9.31 | 6.21E-22 | H2-Bl | histocompatibility 2, blastocyst |
| ENSMUSG00000042428 | 3.22 | 9.30 | 3.26E-49 | Mgat3 | mannoside acetylglucosaminyltransferase 3 |
| ENSMUSG00000056174 | 3.21 | 9.28 | 8.71E-16 | Col8a2 | collagen, type VIII, alpha 2 |
| ENSMUSG00000074506 | 3.21 | 9.28 | 1.58E-09 | NA | NA |
| ENSMUSG00000072531 | 3.21 | 9.28 | 3.56E-04 | NA | NA |
| ENSMUSG00000073664 | 3.21 | 9.26 | 5.76E-45 | Nbeal1 | neurobeachin like 1 |
| ENSMUSG00000082113 | 3.21 | 9.23 | 3.73E-04 | NA | NA |
| ENSMUSG00000022816 | 3.21 | 9.23 | 4.68E-49 | Fstl1 | follistatin-like 1 |
| ENSMUSG00000048621 | 3.21 | 9.22 | 4.10E-11 | Gm6377 | predicted gene 6377 |
| ENSMUSG00000082464 | 3.21 | 9.22 | 8.77E-05 | NA | NA |
| ENSMUSG00000074147 | 3.20 | 9.17 | 7.09E-35 | NA | NA |
| ENSMUSG00000083642 | 3.19 | 9.14 | 1.02E-04 | NA | NA |
| ENSMUSG00000015222 | 3.19 | 9.12 | 7.63E-16 | Map2 | microtubule-associated protein 2 |
| ENSMUSG00000022261 | 3.19 | 9.12 | 1.30E-35 | Sdc2 | syndecan 2 |
| ENSMUSG00000022321 | 3.19 | 9.12 | 1.66E-06 | Cdh10 | cadherin 10 |
| ENSMUSG00000037849 | 3.19 | 9.10 | 3.51E-14 | NA | NA |
| ENSMUSG00000023411 | 3.18 | 9.07 | 1.07E-28 | Nfatc4 | nuclear factor of activated T cells, cytoplasmic, calcineurin dependent 4 |
| ENSMUSG00000048347 | 3.18 | 9.06 | 1.24E-14 | Pcdhb18 | protocadherin beta 18 |
| ENSMUSG00000046856 | 3.18 | 9.06 | 3.56E-05 | Gpr1 | G protein-coupled receptor 1 |
| ENSMUSG00000084838 | 3.18 | 9.05 | 3.25E-13 | NA | NA |
| ENSMUSG00000065431 | 3.17 | 9.02 | 4.52E-04 | NA | NA |
| ENSMUSG00000033676 | 3.17 | 9.02 | 6.39E-11 | Gabrb3 | gamma-aminobutyric acid (GABA) A receptor, subunit beta 3 |
| ENSMUSG00000065475 | 3.17 | 9.01 | 1.19E-04 | NA | NA |
| ENSMUSG00000082035 | 3.17 | 9.00 | 1.66E-05 | NA | NA |
| ENSMUSG00000058736 | 3.17 | 8.98 | 3.10E-04 | NA | NA |
| ENSMUSG00000054366 | 3.17 | 8.98 | 2.92E-11 | NA | NA |
| ENSMUSG00000023886 | 3.17 | 8.97 | 3.04E-38 | Smoc2 | SPARC related modular calcium binding 2 |
| ENSMUSG00000065445 | 3.16 | 8.95 | 4.20E-04 | NA | NA |
| ENSMUSG00000083863 | 3.16 | 8.95 | 1.88E-12 | NA | NA |
| ENSMUSG00000023013 | 3.16 | 8.93 | 2.66E-05 | Aqp2 | aquaporin 2 |
| ENSMUSG00000027107 | 3.16 | 8.91 | 1.79E-12 | Chrna1 | cholinergic receptor, nicotinic, alpha polypeptide 1 (muscle) |
| ENSMUSG00000052821 | 3.15 | 8.91 | 3.93E-29 | Cysltr1 | cysteinyl leukotriene receptor 1 |
| ENSMUSG00000083496 | 3.15 | 8.90 | 3.69E-09 | NA | NA |
| ENSMUSG00000059585 | 3.15 | 8.90 | 2.76E-08 | NA | NA |
| ENSMUSG00000004891 | 3.15 | 8.89 | 7.98E-34 | Nes | nestin |
| ENSMUSG00000031503 | 3.15 | 8.88 | 1.95E-55 | Col4a2 | collagen, type IV, alpha 2 |
| ENSMUSG00000041696 | 3.15 | 8.88 | 1.40E-40 | Rasl12 | RAS-like, family 12 |
| ENSMUSG00000073678 | 3.15 | 8.88 | 1.06E-04 | Pgap1 | post-GPI attachment to proteins 1 |
| ENSMUSG00000050854 | 3.14 | 8.84 | 1.20E-09 | Tmem125 | transmembrane protein 125 |
| ENSMUSG00000013483 | 3.14 | 8.79 | 5.98E-16 | Card14 | caspase recruitment domain family, member 14 |
| ENSMUSG00000042116 | 3.13 | 8.75 | 1.08E-29 | Vwa1 | von Willebrand factor A domain containing 1 |
| ENSMUSG00000084221 | 3.13 | 8.75 | 4.02E-08 | NA | NA |
| ENSMUSG00000036466 | 3.13 | 8.74 | 1.16E-12 | Megf11 | multiple EGF-like-domains 11 |
| ENSMUSG00000087626 | 3.12 | 8.71 | 3.11E-06 | NA | NA |
| ENSMUSG00000028259 | 3.12 | 8.71 | 6.66E-07 | Fhl5 | four and a half LIM domains 5 |
| ENSMUSG00000030606 | 3.12 | 8.70 | 6.38E-28 | Hapln3 | hyaluronan and proteoglycan link protein 3 |
| ENSMUSG00000085412 | 3.12 | 8.68 | 1.84E-05 | NA | NA |
| ENSMUSG00000074273 | 3.11 | 8.61 | 7.96E-11 | NA | NA |
| ENSMUSG00000032271 | 3.11 | 8.61 | 1.34E-27 | Nnmt | nicotinamide N-methyltransferase |
| ENSMUSG00000034377 | 3.11 | 8.61 | 2.46E-19 | NA | NA |
| ENSMUSG00000064080 | 3.11 | 8.61 | 8.49E-17 | Fbln2 | fibulin 2 |
| ENSMUSG00000055963 | 3.10 | 8.59 | 2.75E-19 | Triqk | triple QxxK/R motif containing |
| ENSMUSG00000078686 | 3.10 | 8.58 | 4.11E-04 | NA | NA |
| ENSMUSG00000024208 | 3.10 | 8.58 | 1.65E-15 | Uqcc2 | ubiquinol-cytochrome c reductase complex assembly factor 2 |
| ENSMUSG00000065593 | 3.10 | 8.58 | 3.61E-04 | NA | NA |
| ENSMUSG00000082244 | 3.10 | 8.58 | 9.28E-07 | NA | NA |
| ENSMUSG00000070058 | 3.10 | 8.57 | 4.11E-04 | NA | NA |
| ENSMUSG00000052336 | 3.10 | 8.57 | 9.15E-34 | Cx3cr1 | chemokine (C-X3-C motif) receptor 1 |
| ENSMUSG00000027276 | 3.10 | 8.57 | 5.65E-42 | Jag1 | jagged 1 |
| ENSMUSG00000027716 | 3.10 | 8.57 | 2.95E-12 | Trpc3 | transient receptor potential cation channel, subfamily C, member 3 |
| ENSMUSG00000032744 | 3.10 | 8.56 | 9.81E-30 | Heyl | hairy/enhancer-of-split related with YRPW motif-like |
| ENSMUSG00000055555 | 3.10 | 8.55 | 2.79E-05 | 4930502E18Rik | RIKEN cDNA 4930502E18 gene |
| ENSMUSG00000039221 | 3.09 | 8.54 | 5.43E-09 | Rpl22l1 | ribosomal protein L22 like 1 |
| ENSMUSG00000066475 | 3.09 | 8.52 | 8.60E-16 | NA | NA |
| ENSMUSG00000044528 | 3.09 | 8.52 | 6.30E-09 | Tram1l1 | translocation associated membrane protein 1-like 1 |
| ENSMUSG00000021411 | 3.09 | 8.51 | 2.47E-34 | Pxdc1 | PX domain containing 1 |
| ENSMUSG00000046078 | 3.09 | 8.50 | 6.56E-04 | NA | NA |
| ENSMUSG00000079840 | 3.08 | 8.46 | 6.78E-04 | NA | NA |
| ENSMUSG00000064741 | 3.08 | 8.45 | 3.67E-06 | NA | NA |
| ENSMUSG00000080503 | 3.08 | 8.44 | 7.05E-04 | NA | NA |
| ENSMUSG00000053615 | 3.08 | 8.43 | 2.33E-04 | NA | NA |
| ENSMUSG00000032099 | 3.08 | 8.43 | 6.96E-04 | Pate4 | prostate and testis expressed 4 |
| ENSMUSG00000083004 | 3.07 | 8.42 | 8.93E-05 | NA | NA |
| ENSMUSG00000033149 | 3.07 | 8.39 | 1.35E-50 | Phldb2 | pleckstrin homology-like domain, family B, member 2 |
| ENSMUSG00000081953 | 3.06 | 8.36 | 2.40E-07 | NA | NA |
| ENSMUSG00000027840 | 3.06 | 8.35 | 4.03E-29 | Wnt2b | wingless related MMTV integration site 2b |
| ENSMUSG00000085607 | 3.06 | 8.33 | 5.22E-15 | NA | NA |
| ENSMUSG00000016918 | 3.06 | 8.31 | 2.89E-64 | Sulf1 | sulfatase 1 |
| ENSMUSG00000042045 | 3.05 | 8.31 | 1.77E-05 | Sln | sarcolipin |
| ENSMUSG00000074415 | 3.05 | 8.30 | 5.77E-19 | 2610203C20Rik | RIKEN cDNA 2610203C20 gene |
| ENSMUSG00000031778 | 3.05 | 8.29 | 3.32E-38 | Cx3cl1 | chemokine (C-X3-C motif) ligand 1 |
| ENSMUSG00000081620 | 3.05 | 8.28 | 6.52E-16 | NA | NA |
| ENSMUSG00000062929 | 3.05 | 8.25 | 2.35E-32 | Cfl2 | cofilin 2, muscle |
| ENSMUSG00000026414 | 3.04 | 8.24 | 1.12E-08 | Tnnt2 | troponin T2, cardiac |
| ENSMUSG00000016128 | 3.04 | 8.22 | 5.87E-41 | Stard13 | StAR-related lipid transfer (START) domain containing 13 |
| ENSMUSG00000079363 | 3.04 | 8.22 | 3.14E-51 | Gbp4 | guanylate binding protein 4 |
| ENSMUSG00000082072 | 3.04 | 8.21 | 8.23E-04 | NA | NA |
| ENSMUSG00000032530 | 3.03 | 8.20 | 3.45E-06 | Lyzl4 | lysozyme-like 4 |
| ENSMUSG00000025272 | 3.03 | 8.19 | 3.08E-17 | Tro | trophinin |
| ENSMUSG00000084433 | 3.03 | 8.18 | 8.64E-04 | NA | NA |
| ENSMUSG00000044117 | 3.03 | 8.18 | 3.58E-07 | 2900011O08Rik | RIKEN cDNA 2900011O08 gene |
| ENSMUSG00000085295 | 3.03 | 8.16 | 1.58E-18 | NA | NA |
| ENSMUSG00000021095 | 3.03 | 8.15 | 1.43E-05 | Gsc | goosecoid homeobox |
| ENSMUSG00000025666 | 3.02 | 8.14 | 3.97E-25 | Tmem47 | transmembrane protein 47 |
| ENSMUSG00000051515 | 3.02 | 8.13 | 3.42E-04 | NA | NA |
| ENSMUSG00000082128 | 3.02 | 8.13 | 9.93E-06 | NA | NA |
| ENSMUSG00000022440 | 3.02 | 8.11 | 2.51E-40 | C1qtnf6 | C1q and tumor necrosis factor related protein 6 |
| ENSMUSG00000021565 | 3.01 | 8.08 | 9.73E-05 | Slc6a19 | solute carrier family 6 (neurotransmitter transporter), member 19 |
| ENSMUSG00000046731 | 3.01 | 8.07 | 1.07E-39 | Kctd11 | potassium channel tetramerisation domain containing 11 |
| ENSMUSG00000027087 | 3.01 | 8.06 | 2.41E-32 | Itgav | integrin alpha V |
| ENSMUSG00000079889 | 3.01 | 8.06 | 9.04E-08 | NA | NA |
| ENSMUSG00000022766 | 3.01 | 8.05 | 3.65E-08 | Serpind1 | serine (or cysteine) peptidase inhibitor, clade D, member 1 |
| ENSMUSG00000072551 | 3.01 | 8.04 | 6.42E-05 | NA | NA |
| ENSMUSG00000081892 | 3.01 | 8.03 | 1.18E-05 | NA | NA |
| ENSMUSG00000065453 | 3.00 | 8.02 | 2.00E-04 | Mirlet7d | microRNA let7d |
| ENSMUSG00000067558 | 3.00 | 8.01 | 9.61E-06 | NA | NA |
| ENSMUSG00000040690 | 3.00 | 7.99 | 7.22E-19 | Col16a1 | collagen, type XVI, alpha 1 |
| ENSMUSG00000028591 | 2.99 | 7.97 | 2.16E-04 | Pramef12 | PRAME family member 12 |
| ENSMUSG00000040875 | 2.99 | 7.97 | 1.50E-16 | Osbpl10 | oxysterol binding protein-like 10 |
| ENSMUSG00000049929 | 2.99 | 7.96 | 1.50E-11 | Lpar4 | lysophosphatidic acid receptor 4 |
| ENSMUSG00000039529 | 2.99 | 7.95 | 6.98E-46 | Atp8b1 | ATPase, class I, type 8B, member 1 |
| ENSMUSG00000021136 | 2.99 | 7.92 | 1.08E-51 | Smoc1 | SPARC related modular calcium binding 1 |
| ENSMUSG00000079219 | 2.98 | 7.91 | 1.05E-03 | NA | NA |
| ENSMUSG00000029417 | 2.98 | 7.91 | 2.37E-16 | Cxcl9 | chemokine (C-X-C motif) ligand 9 |
| ENSMUSG00000023885 | 2.98 | 7.88 | 3.55E-25 | Thbs2 | thrombospondin 2 |
| ENSMUSG00000040264 | 2.97 | 7.85 | 3.20E-29 | Gbp5 | guanylate binding protein 5 |
| ENSMUSG00000031290 | 2.97 | 7.84 | 1.60E-24 | Lrch2 | leucine-rich repeats and calponin homology (CH) domain containing 2 |
| ENSMUSG00000042279 | 2.97 | 7.83 | 8.62E-04 | H1foo | H1 histone family, member O, oocyte-specific |
| ENSMUSG00000028269 | 2.97 | 7.82 | 2.86E-23 | Gbp2b | guanylate binding protein 2b |
| ENSMUSG00000082195 | 2.96 | 7.81 | 2.37E-21 | Gm13034 | SWI/SNF related, matrix associated, actin dependent regulator of chromatin, subfamily a, member 5 pseudogene |
| ENSMUSG00000075591 | 2.96 | 7.81 | 3.36E-04 | NA | NA |
| ENSMUSG00000090000 | 2.96 | 7.79 | 8.94E-35 | Ier3ip1 | immediate early response 3 interacting protein 1 |
| ENSMUSG00000045873 | 2.96 | 7.79 | 3.87E-04 | NA | NA |
| ENSMUSG00000041673 | 2.96 | 7.79 | 3.07E-06 | Lrrc18 | leucine rich repeat containing 18 |
| ENSMUSG00000044365 | 2.96 | 7.77 | 2.27E-04 | Cxxc4 | CXXC finger 4 |
| ENSMUSG00000035783 | 2.96 | 7.77 | 6.31E-47 | Acta2 | actin, alpha 2, smooth muscle, aorta |
| ENSMUSG00000050974 | 2.96 | 7.76 | 4.59E-07 | NA | NA |
| ENSMUSG00000015134 | 2.96 | 7.76 | 7.97E-23 | Aldh1a3 | aldehyde dehydrogenase family 1, subfamily A3 |
| ENSMUSG00000038402 | 2.96 | 7.76 | 6.79E-12 | Foxf2 | forkhead box F2 |
| ENSMUSG00000030046 | 2.95 | 7.74 | 1.21E-03 | Bmp10 | bone morphogenetic protein 10 |
| ENSMUSG00000022122 | 2.95 | 7.74 | 3.91E-20 | Ednrb | endothelin receptor type B |
| ENSMUSG00000083199 | 2.95 | 7.73 | 7.60E-06 | NA | NA |
| ENSMUSG00000036446 | 2.95 | 7.73 | 8.94E-29 | Lum | lumican |
| ENSMUSG00000073452 | 2.95 | 7.71 | 2.76E-09 | NA | NA |
| ENSMUSG00000031125 | 2.94 | 7.70 | 2.54E-04 | 3830403N18Rik | RIKEN cDNA 3830403N18 gene |
| ENSMUSG00000022656 | 2.94 | 7.68 | 5.64E-30 | Pvrl3 | poliovirus receptor-related 3 |
| ENSMUSG00000040289 | 2.94 | 7.66 | 4.61E-17 | Hey1 | hairy/enhancer-of-split related with YRPW motif 1 |
| ENSMUSG00000053820 | 2.93 | 7.65 | 1.31E-03 | Bcl2a1c | B cell leukemia/lymphoma 2 related protein A1c |
| ENSMUSG00000070564 | 2.93 | 7.64 | 2.66E-04 | Ntn5 | netrin 5 |
| ENSMUSG00000080242 | 2.93 | 7.63 | 4.76E-25 | Atp6v0c-ps2 | ATPase, H+ transporting, lysosomal V0 subunit C, pseudogene 2 |
| ENSMUSG00000088642 | 2.93 | 7.63 | 1.01E-03 | NA | NA |
| ENSMUSG00000087400 | 2.93 | 7.60 | 8.17E-10 | NA | NA |
| ENSMUSG00000049288 | 2.92 | 7.59 | 1.40E-22 | Lix1l | Lix1-like |
| ENSMUSG00000029851 | 2.92 | 7.58 | 2.12E-23 | Fam115c | family with sequence similarity 115, member C |
| ENSMUSG00000083283 | 2.92 | 7.58 | 1.37E-05 | NA | NA |
| ENSMUSG00000018930 | 2.92 | 7.57 | 1.83E-10 | Ccl4 | chemokine (C-C motif) ligand 4 |
| ENSMUSG00000087600 | 2.92 | 7.57 | 1.06E-03 | NA | NA |
| ENSMUSG00000083429 | 2.92 | 7.56 | 3.10E-05 | NA | NA |
| ENSMUSG00000061048 | 2.92 | 7.55 | 2.09E-05 | Cdh3 | cadherin 3 |
| ENSMUSG00000027978 | 2.92 | 7.55 | 1.20E-07 | Prss12 | protease, serine 12 neurotrypsin (motopsin) |
| ENSMUSG00000064272 | 2.92 | 7.55 | 4.15E-06 | Gpbar1 | G protein-coupled bile acid receptor 1 |
| ENSMUSG00000040428 | 2.91 | 7.54 | 1.28E-25 | Plekha4 | pleckstrin homology domain containing, family A (phosphoinositide binding specific) member 4 |
| ENSMUSG00000021260 | 2.91 | 7.52 | 4.47E-25 | Hhipl1 | hedgehog interacting protein-like 1 |
| ENSMUSG00000029838 | 2.91 | 7.51 | 5.46E-43 | Ptn | pleiotrophin |
| ENSMUSG00000026837 | 2.91 | 7.50 | 7.74E-32 | Col5a1 | collagen, type V, alpha 1 |
| ENSMUSG00000064696 | 2.91 | 7.50 | 3.79E-04 | NA | NA |
| ENSMUSG00000072883 | 2.91 | 7.49 | 1.09E-03 | NA | NA |
| ENSMUSG00000030615 | 2.90 | 7.49 | 3.91E-47 | Tmem126a | transmembrane protein 126A |
| ENSMUSG00000022504 | 2.90 | 7.47 | 6.65E-28 | Ciita | class II transactivator |
| ENSMUSG00000038074 | 2.90 | 7.47 | 2.81E-31 | Fkbp14 | FK506 binding protein 14 |
| ENSMUSG00000038932 | 2.90 | 7.46 | 3.51E-10 | Tcfl5 | transcription factor-like 5 (basic helix-loop-helix) |
| ENSMUSG00000087385 | 2.90 | 7.46 | 2.34E-04 | AA415398 | expressed sequence AA415398 |
| ENSMUSG00000033717 | 2.90 | 7.45 | 6.95E-34 | Adra2a | adrenergic receptor, alpha 2a |
| ENSMUSG00000083380 | 2.90 | 7.45 | 1.18E-07 | NA | NA |
| ENSMUSG00000040653 | 2.90 | 7.44 | 4.50E-15 | Ppp1r14c | protein phosphatase 1, regulatory (inhibitor) subunit 14c |
| ENSMUSG00000052485 | 2.89 | 7.42 | 8.41E-06 | Tmem171 | transmembrane protein 171 |
| ENSMUSG00000081539 | 2.89 | 7.41 | 1.56E-03 | NA | NA |
| ENSMUSG00000088119 | 2.89 | 7.39 | 1.59E-03 | NA | NA |
| ENSMUSG00000015443 | 2.89 | 7.39 | 1.60E-03 | Gzmn | granzyme N |
| ENSMUSG00000037411 | 2.88 | 7.38 | 2.28E-07 | Serpine1 | serine (or cysteine) peptidase inhibitor, clade E, member 1 |
| ENSMUSG00000022178 | 2.88 | 7.38 | 1.48E-34 | Ajuba | ajuba LIM protein |
| ENSMUSG00000032419 | 2.88 | 7.38 | 1.20E-24 | Tbx18 | T-box18 |
| ENSMUSG00000035133 | 2.88 | 7.38 | 1.71E-28 | Arhgap5 | Rho GTPase activating protein 5 |
| ENSMUSG00000045932 | 2.88 | 7.37 | 3.91E-54 | Ifit2 | interferon-induced protein with tetratricopeptide repeats 2 |
| ENSMUSG00000044408 | 2.88 | 7.37 | 4.88E-33 | Sptssa | serine palmitoyltransferase, small subunit A |
| ENSMUSG00000074142 | 2.88 | 7.36 | 7.74E-32 | NA | NA |
| ENSMUSG00000067276 | 2.88 | 7.36 | 3.42E-12 | Capn6 | calpain 6 |
| ENSMUSG00000079803 | 2.88 | 7.35 | 1.65E-03 | NA | NA |
| ENSMUSG00000059814 | 2.87 | 7.34 | 2.97E-18 | NA | NA |
| ENSMUSG00000085173 | 2.87 | 7.32 | 5.53E-06 | NA | NA |
| ENSMUSG00000066000 | 2.87 | 7.31 | 3.48E-17 | 2610305D13Rik | RIKEN cDNA 2610305D13 gene |
| ENSMUSG00000021665 | 2.87 | 7.31 | 1.06E-40 | Hexb | hexosaminidase B |
| ENSMUSG00000027102 | 2.87 | 7.30 | 2.26E-13 | Hoxd8 | homeobox D8 |
| ENSMUSG00000081657 | 2.87 | 7.29 | 2.55E-10 | NA | NA |
| ENSMUSG00000079017 | 2.86 | 7.27 | 2.14E-21 | Ifi27l2a | interferon, alpha-inducible protein 27 like 2A |
| ENSMUSG00000028268 | 2.86 | 7.27 | 1.21E-37 | Gbp3 | guanylate binding protein 3 |
| ENSMUSG00000032766 | 2.86 | 7.26 | 3.08E-31 | Gng11 | guanine nucleotide binding protein (G protein), gamma 11 |
| ENSMUSG00000057796 | 2.86 | 7.26 | 1.24E-18 | NA | NA |
| ENSMUSG00000021822 | 2.86 | 7.26 | 6.14E-52 | Plau | plasminogen activator, urokinase |
| ENSMUSG00000037944 | 2.86 | 7.26 | 3.11E-07 | Ccr7 | chemokine (C-C motif) receptor 7 |
| ENSMUSG00000046169 | 2.85 | 7.22 | 1.57E-23 | Adamts6 | a disintegrin-like and metallopeptidase (reprolysin type) with thrombospondin type 1 motif, 6 |
| ENSMUSG00000028347 | 2.85 | 7.20 | 3.40E-17 | Tmeff1 | transmembrane protein with EGF-like and two follistatin-like domains 1 |
| ENSMUSG00000078921 | 2.84 | 7.16 | 5.04E-43 | NA | NA |
| ENSMUSG00000066724 | 2.84 | 7.16 | 1.92E-03 | NA | NA |
| ENSMUSG00000037568 | 2.84 | 7.15 | 1.14E-25 | Vash2 | vasohibin 2 |
| ENSMUSG00000054976 | 2.84 | 7.14 | 1.43E-14 | Nyap2 | neuronal tyrosine-phophorylated phosphoinositide 3-kinase adaptor 2 |
| ENSMUSG00000074148 | 2.84 | 7.14 | 3.53E-17 | NA | NA |
| ENSMUSG00000038141 | 2.83 | 7.13 | 3.67E-09 | Tmem181a | transmembrane protein 181A |
| ENSMUSG00000066677 | 2.83 | 7.12 | 2.61E-16 | Pydc3 | pyrin domain containing 3 |
| ENSMUSG00000021098 | 2.83 | 7.12 | 1.40E-07 | 4930447C04Rik | RIKEN cDNA 4930447C04 gene |
| ENSMUSG00000022940 | 2.83 | 7.11 | 1.38E-17 | Pigp | phosphatidylinositol glycan anchor biosynthesis, class P |
| ENSMUSG00000047104 | 2.83 | 7.11 | 2.01E-03 | Pbp2 | phosphatidylethanolamine binding protein 2 |
| ENSMUSG00000024766 | 2.83 | 7.10 | 2.27E-17 | Lipo1 | lipase, member O1 |
| ENSMUSG00000028459 | 2.83 | 7.09 | 6.70E-22 | Cd72 | CD72 antigen |
| ENSMUSG00000083392 | 2.82 | 7.08 | 5.84E-04 | NA | NA |
| ENSMUSG00000043020 | 2.82 | 7.08 | 2.37E-07 | Wdr63 | WD repeat domain 63 |
| ENSMUSG00000027351 | 2.82 | 7.08 | 6.27E-98 | Spred1 | sprouty protein with EVH-1 domain 1, related sequence |
| ENSMUSG00000086274 | 2.82 | 7.07 | 1.83E-03 | NA | NA |
| ENSMUSG00000063632 | 2.82 | 7.07 | 6.27E-11 | Sox11 | SRY (sex determining region Y)-box 11 |
| ENSMUSG00000038984 | 2.82 | 7.07 | 9.87E-05 | Tspyl5 | testis-specific protein, Y-encoded-like 5 |
| ENSMUSG00000073197 | 2.82 | 7.06 | 2.11E-03 | 5730507C01Rik | RIKEN cDNA 5730507C01 gene |
| ENSMUSG00000073549 | 2.82 | 7.05 | 5.03E-07 | NA | NA |
| ENSMUSG00000040552 | 2.81 | 7.02 | 1.15E-16 | C3ar1 | complement component 3a receptor 1 |
| ENSMUSG00000067780 | 2.81 | 7.02 | 2.42E-11 | Pi15 | peptidase inhibitor 15 |
| ENSMUSG00000057182 | 2.81 | 7.02 | 2.87E-10 | Scn3a | sodium channel, voltage-gated, type III, alpha |
| ENSMUSG00000061654 | 2.81 | 7.02 | 2.17E-03 | Spry3 | sprouty homolog 3 (Drosophila) |
| ENSMUSG00000022055 | 2.81 | 7.01 | 1.57E-03 | Nefl | neurofilament, light polypeptide |
| ENSMUSG00000024084 | 2.81 | 6.99 | 6.86E-34 | Qpct | glutaminyl-peptide cyclotransferase (glutaminyl cyclase) |
| ENSMUSG00000031274 | 2.80 | 6.99 | 5.35E-09 | Col4a5 | collagen, type IV, alpha 5 |
| ENSMUSG00000027661 | 2.80 | 6.98 | 2.59E-26 | Slc2a10 | solute carrier family 2 (facilitated glucose transporter), member 10 |
| ENSMUSG00000075399 | 2.80 | 6.98 | 2.34E-04 | NA | NA |
| ENSMUSG00000044338 | 2.80 | 6.97 | 3.36E-22 | Aplnr | apelin receptor |
| ENSMUSG00000040899 | 2.80 | 6.97 | 1.53E-08 | Ccr6 | chemokine (C-C motif) receptor 6 |
| ENSMUSG00000079471 | 2.80 | 6.97 | 2.16E-03 | Gm7325 | predicted gene 7325 |
| ENSMUSG00000078190 | 2.80 | 6.97 | 1.20E-20 | Dnm3os | dynamin 3, opposite strand |
| ENSMUSG00000084141 | 2.80 | 6.96 | 4.58E-05 | NA | NA |
| ENSMUSG00000002847 | 2.79 | 6.94 | 1.15E-21 | Pla1a | phospholipase A1 member A |
| ENSMUSG00000078183 | 2.79 | 6.94 | 1.61E-12 | NA | NA |
| ENSMUSG00000020674 | 2.79 | 6.93 | 1.66E-22 | Pxdn | peroxidasin homolog (Drosophila) |
| ENSMUSG00000059493 | 2.79 | 6.92 | 3.05E-19 | Nhs | Nance-Horan syndrome (human) |
| ENSMUSG00000043613 | 2.79 | 6.89 | 3.47E-04 | Mmp3 | matrix metallopeptidase 3 |
| ENSMUSG00000088769 | 2.78 | 6.88 | 1.75E-04 | NA | NA |
| ENSMUSG00000036334 | 2.78 | 6.88 | 3.06E-24 | Igsf10 | immunoglobulin superfamily, member 10 |
| ENSMUSG00000038879 | 2.78 | 6.86 | 4.60E-06 | Nipal2 | NIPA-like domain containing 2 |
| ENSMUSG00000036995 | 2.78 | 6.86 | 2.28E-10 | Asap3 | ArfGAP with SH3 domain, ankyrin repeat and PH domain 3 |
| ENSMUSG00000089547 | 2.78 | 6.85 | 2.63E-04 | NA | NA |
| ENSMUSG00000037784 | 2.77 | 6.84 | 5.70E-37 | Dzip1l | DAZ interacting protein 1-like |
| ENSMUSG00000072844 | 2.77 | 6.84 | 1.92E-10 | G530011O06Rik | RIKEN cDNA G530011O06 gene |
| ENSMUSG00000020393 | 2.77 | 6.83 | 2.04E-48 | Kremen1 | kringle containing transmembrane protein 1 |
| ENSMUSG00000045377 | 2.77 | 6.83 | 8.14E-10 | Tmem88 | transmembrane protein 88 |
| ENSMUSG00000084691 | 2.77 | 6.83 | 1.11E-04 | NA | NA |
| ENSMUSG00000020695 | 2.77 | 6.82 | 1.20E-32 | Mrc2 | mannose receptor, C type 2 |
| ENSMUSG00000071344 | 2.77 | 6.81 | 1.14E-14 | NA | NA |
| ENSMUSG00000043223 | 2.77 | 6.81 | 3.65E-05 | NA | NA |
| ENSMUSG00000029561 | 2.77 | 6.80 | 3.56E-19 | Oasl2 | 2'-5' oligoadenylate synthetase-like 2 |
| ENSMUSG00000061013 | 2.77 | 6.80 | 8.39E-09 | Mkx | mohawk homeobox |
| ENSMUSG00000020099 | 2.77 | 6.80 | 1.73E-21 | Unc5b | unc-5 homolog B (C. elegans) |
| ENSMUSG00000052026 | 2.76 | 6.80 | 8.64E-19 | Slc6a7 | solute carrier family 6 (neurotransmitter transporter, L-proline), member 7 |
| ENSMUSG00000044674 | 2.76 | 6.79 | 9.88E-31 | Fzd1 | frizzled homolog 1 (Drosophila) |
| ENSMUSG00000048388 | 2.76 | 6.79 | 3.39E-21 | Fam171b | family with sequence similarity 171, member B |
| ENSMUSG00000020241 | 2.76 | 6.77 | 2.46E-33 | Col6a2 | collagen, type VI, alpha 2 |
| ENSMUSG00000025586 | 2.76 | 6.76 | 2.60E-14 | Cpeb1 | cytoplasmic polyadenylation element binding protein 1 |
| ENSMUSG00000027896 | 2.76 | 6.75 | 5.19E-40 | Slc16a4 | solute carrier family 16 (monocarboxylic acid transporters), member 4 |
| ENSMUSG00000029761 | 2.75 | 6.75 | 2.87E-32 | Cald1 | caldesmon 1 |
| ENSMUSG00000031202 | 2.75 | 6.74 | 3.60E-05 | Rab39b | RAB39B, member RAS oncogene family |
| ENSMUSG00000037370 | 2.75 | 6.74 | 6.39E-37 | Enpp1 | ectonucleotide pyrophosphatase/phosphodiesterase 1 |
| ENSMUSG00000072528 | 2.75 | 6.74 | 1.87E-05 | NA | NA |
| ENSMUSG00000082844 | 2.75 | 6.74 | 2.74E-03 | NA | NA |
| ENSMUSG00000042104 | 2.75 | 6.73 | 3.64E-34 | Uggt2 | UDP-glucose glycoprotein glucosyltransferase 2 |
| ENSMUSG00000013584 | 2.75 | 6.73 | 1.85E-16 | Aldh1a2 | aldehyde dehydrogenase family 1, subfamily A2 |
| ENSMUSG00000028736 | 2.75 | 6.72 | 2.46E-05 | Pax7 | paired box 7 |
| ENSMUSG00000020857 | 2.75 | 6.71 | 9.37E-24 | NA | NA |
| ENSMUSG00000079298 | 2.75 | 6.71 | 4.34E-08 | Klrb1b | killer cell lectin-like receptor subfamily B member 1B |
| ENSMUSG00000070524 | 2.74 | 6.70 | 4.22E-10 | Fcrlb | Fc receptor-like B |
| ENSMUSG00000030116 | 2.74 | 6.70 | 1.07E-06 | Mfap5 | microfibrillar associated protein 5 |
| ENSMUSG00000004885 | 2.74 | 6.70 | 2.85E-10 | Crabp2 | cellular retinoic acid binding protein II |
| ENSMUSG00000006369 | 2.74 | 6.69 | 2.05E-22 | Fbln1 | fibulin 1 |
| ENSMUSG00000086429 | 2.74 | 6.69 | 2.24E-24 | Gt(ROSA)26Sor | gene trap ROSA 26, Philippe Soriano |
| ENSMUSG00000069662 | 2.74 | 6.68 | 1.75E-15 | Marcks | myristoylated alanine rich protein kinase C substrate |
| ENSMUSG00000036144 | 2.74 | 6.68 | 1.33E-15 | Meox2 | mesenchyme homeobox 2 |
| ENSMUSG00000086150 | 2.74 | 6.67 | 6.07E-12 | Bach2os | BTB and CNC homology 2, opposite strand |
| ENSMUSG00000086105 | 2.74 | 6.67 | 1.36E-03 | NA | NA |
| ENSMUSG00000032997 | 2.73 | 6.65 | 1.05E-21 | Chpf | chondroitin polymerizing factor |
| ENSMUSG00000039001 | 2.73 | 6.65 | 7.13E-17 | NA | NA |
| ENSMUSG00000032011 | 2.73 | 6.64 | 3.42E-20 | Thy1 | thymus cell antigen 1, theta |
| ENSMUSG00000002900 | 2.73 | 6.64 | 1.32E-47 | Lamb1 | laminin B1 |
| ENSMUSG00000025044 | 2.73 | 6.64 | 4.27E-37 | Msr1 | macrophage scavenger receptor 1 |
| ENSMUSG00000000581 | 2.73 | 6.63 | 3.62E-37 | C1d | C1D nuclear receptor co-repressor |
| ENSMUSG00000034675 | 2.73 | 6.63 | 2.35E-15 | Dbn1 | drebrin 1 |
| ENSMUSG00000079190 | 2.72 | 6.61 | 3.05E-03 | LOC100041057 | nuclear body protein SP140-like |
| ENSMUSG00000041624 | 2.72 | 6.61 | 1.62E-04 | Gucy1a2 | guanylate cyclase 1, soluble, alpha 2 |
| ENSMUSG00000038305 | 2.72 | 6.60 | 7.40E-14 | Spats2l | spermatogenesis associated, serine-rich 2-like |
| ENSMUSG00000021701 | 2.72 | 6.60 | 1.02E-12 | Plk2 | polo-like kinase 2 |
| ENSMUSG00000022586 | 2.72 | 6.60 | 1.77E-05 | Ly6i | lymphocyte antigen 6 complex, locus I |
| ENSMUSG00000058163 | 2.72 | 6.60 | 7.15E-27 | Gm5431 | predicted gene 5431 |
| ENSMUSG00000086304 | 2.72 | 6.59 | 2.18E-24 | NA | NA |
| ENSMUSG00000052951 | 2.72 | 6.58 | 5.05E-18 | NA | NA |
| ENSMUSG00000089769 | 2.72 | 6.58 | 1.17E-11 | NA | NA |
| ENSMUSG00000055958 | 2.72 | 6.58 | 1.68E-03 | NA | NA |
| ENSMUSG00000021381 | 2.72 | 6.57 | 7.21E-04 | Barx1 | BarH-like homeobox 1 |
| ENSMUSG00000079362 | 2.71 | 6.56 | 9.45E-22 | Gbp6 | guanylate binding protein 6 |
| ENSMUSG00000074598 | 2.71 | 6.56 | 2.91E-04 | NA | NA |
| ENSMUSG00000020354 | 2.71 | 6.56 | 1.12E-10 | Sgcd | sarcoglycan, delta (dystrophin-associated glycoprotein) |
| ENSMUSG00000047686 | 2.71 | 6.55 | 5.33E-10 | Zcchc5 | zinc finger, CCHC domain containing 5 |
| ENSMUSG00000068392 | 2.71 | 6.54 | 2.13E-03 | Rnase13 | ribonuclease, RNase A family, 13 (non-active) |
| ENSMUSG00000005802 | 2.71 | 6.54 | 2.41E-41 | Slc30a4 | solute carrier family 30 (zinc transporter), member 4 |
| ENSMUSG00000034460 | 2.71 | 6.53 | 2.06E-17 | Six4 | sine oculis-related homeobox 4 |
| ENSMUSG00000040164 | 2.71 | 6.53 | 5.82E-10 | Kcns1 | K+ voltage-gated channel, subfamily S, 1 |
| ENSMUSG00000082838 | 2.71 | 6.52 | 3.26E-03 | NA | NA |
| ENSMUSG00000036459 | 2.71 | 6.52 | 2.06E-26 | Wtip | WT1-interacting protein |
| ENSMUSG00000022722 | 2.70 | 6.52 | 2.40E-23 | Arl6 | ADP-ribosylation factor-like 6 |
| ENSMUSG00000086330 | 2.70 | 6.52 | 1.95E-05 | 1700007J10Rik | RIKEN cDNA 1700007J10 gene |
| ENSMUSG00000000957 | 2.70 | 6.51 | 1.17E-26 | Mmp14 | matrix metallopeptidase 14 (membrane-inserted) |
| ENSMUSG00000030124 | 2.70 | 6.51 | 7.59E-31 | Lag3 | lymphocyte-activation gene 3 |
| ENSMUSG00000039476 | 2.70 | 6.51 | 2.97E-19 | Prrx2 | paired related homeobox 2 |
| ENSMUSG00000028167 | 2.70 | 6.51 | 2.51E-11 | Bdh2 | 3-hydroxybutyrate dehydrogenase, type 2 |
| ENSMUSG00000030638 | 2.70 | 6.50 | 1.34E-05 | Sh3gl3 | SH3-domain GRB2-like 3 |
| ENSMUSG00000081071 | 2.70 | 6.50 | 7.68E-05 | NA | NA |
| ENSMUSG00000079194 | 2.70 | 6.50 | 3.35E-03 | NA | NA |
| ENSMUSG00000028488 | 2.70 | 6.49 | 7.98E-07 | Sh3gl2 | SH3-domain GRB2-like 2 |
| ENSMUSG00000032769 | 2.70 | 6.49 | 3.19E-19 | Trpa1 | transient receptor potential cation channel, subfamily A, member 1 |
| ENSMUSG00000022057 | 2.69 | 6.46 | 7.05E-06 | Adamdec1 | ADAM-like, decysin 1 |
| ENSMUSG00000081235 | 2.69 | 6.45 | 3.47E-03 | NA | NA |
| ENSMUSG00000057924 | 2.69 | 6.45 | 1.86E-06 | NA | NA |
| ENSMUSG00000020473 | 2.69 | 6.45 | 6.50E-16 | Aebp1 | AE binding protein 1 |
| ENSMUSG00000037605 | 2.69 | 6.44 | 2.13E-11 | Lphn3 | latrophilin 3 |
| ENSMUSG00000047238 | 2.69 | 6.44 | 3.01E-33 | Mageh1 | melanoma antigen, family H, 1 |
| ENSMUSG00000047509 | 2.69 | 6.44 | 1.29E-28 | NA | NA |
| ENSMUSG00000079147 | 2.69 | 6.44 | 2.91E-03 | NA | NA |
| ENSMUSG00000036412 | 2.69 | 6.44 | 2.34E-11 | Arsi | arylsulfatase i |
| ENSMUSG00000027750 | 2.68 | 6.42 | 7.47E-19 | Postn | periostin, osteoblast specific factor |
| ENSMUSG00000049699 | 2.68 | 6.42 | 1.42E-04 | Ucn2 | urocortin 2 |
| ENSMUSG00000017057 | 2.68 | 6.42 | 5.70E-58 | Il13ra1 | interleukin 13 receptor, alpha 1 |
| ENSMUSG00000074408 | 2.68 | 6.41 | 2.66E-08 | NA | NA |
| ENSMUSG00000067377 | 2.68 | 6.41 | 4.27E-13 | Tspan6 | tetraspanin 6 |
| ENSMUSG00000034755 | 2.68 | 6.40 | 1.63E-07 | Pcdh11x | protocadherin 11 X-linked |
| ENSMUSG00000042595 | 2.68 | 6.40 | 2.09E-18 | Fam199x | family with sequence similarity 199, X-linked |
| ENSMUSG00000084592 | 2.68 | 6.40 | 1.98E-03 | NA | NA |
| ENSMUSG00000060240 | 2.68 | 6.39 | 2.71E-05 | Cend1 | cell cycle exit and neuronal differentiation 1 |
| ENSMUSG00000085977 | 2.68 | 6.39 | 6.77E-08 | NA | NA |
| ENSMUSG00000034205 | 2.67 | 6.38 | 2.50E-17 | NA | NA |
| ENSMUSG00000021877 | 2.67 | 6.37 | 2.55E-29 | Arf4 | ADP-ribosylation factor 4 |
| ENSMUSG00000026586 | 2.67 | 6.37 | 2.73E-33 | Prrx1 | paired related homeobox 1 |
| ENSMUSG00000055593 | 2.67 | 6.36 | 4.77E-17 | NA | NA |
| ENSMUSG00000020059 | 2.67 | 6.36 | 1.53E-03 | Sycp3 | synaptonemal complex protein 3 |
| ENSMUSG00000021087 | 2.67 | 6.36 | 4.89E-11 | Rtn1 | reticulon 1 |
| ENSMUSG00000040483 | 2.67 | 6.35 | 1.45E-26 | Xaf1 | XIAP associated factor 1 |
| ENSMUSG00000035407 | 2.67 | 6.35 | 4.93E-16 | Kank4 | KN motif and ankyrin repeat domains 4 |
| ENSMUSG00000000693 | 2.66 | 6.34 | 1.77E-34 | Loxl3 | lysyl oxidase-like 3 |
| ENSMUSG00000017144 | 2.66 | 6.34 | 8.85E-34 | Rnd3 | Rho family GTPase 3 |
| ENSMUSG00000032911 | 2.66 | 6.34 | 2.40E-19 | Cspg4 | chondroitin sulfate proteoglycan 4 |
| ENSMUSG00000073202 | 2.66 | 6.33 | 4.64E-15 | NA | NA |
| ENSMUSG00000054446 | 2.66 | 6.32 | 3.91E-03 | Cpa1 | carboxypeptidase A1, pancreatic |
| ENSMUSG00000004267 | 2.66 | 6.32 | 1.18E-21 | Eno2 | enolase 2, gamma neuronal |
| ENSMUSG00000083356 | 2.66 | 6.31 | 1.08E-03 | NA | NA |
| ENSMUSG00000030519 | 2.66 | 6.30 | 1.44E-07 | Apba2 | amyloid beta (A4) precursor protein-binding, family A, member 2 |
| ENSMUSG00000047330 | 2.65 | 6.29 | 1.94E-10 | Kcne4 | potassium voltage-gated channel, Isk-related subfamily, gene 4 |
| ENSMUSG00000083562 | 2.65 | 6.28 | 4.02E-03 | NA | NA |
| ENSMUSG00000053219 | 2.65 | 6.28 | 2.25E-04 | NA | NA |
| ENSMUSG00000083411 | 2.65 | 6.28 | 1.19E-04 | NA | NA |
| ENSMUSG00000031530 | 2.65 | 6.28 | 2.01E-16 | Dusp4 | dual specificity phosphatase 4 |
| ENSMUSG00000079295 | 2.65 | 6.28 | 2.70E-03 | Klrb1-ps1 | killer cell lectin-like receptor subfamily B member 1, pseudogene 1 |
| ENSMUSG00000037868 | 2.65 | 6.28 | 6.30E-09 | Egr2 | early growth response 2 |
| ENSMUSG00000070063 | 2.65 | 6.28 | 1.06E-04 | NA | NA |
| ENSMUSG00000062609 | 2.65 | 6.27 | 4.59E-23 | Kcnj15 | potassium inwardly-rectifying channel, subfamily J, member 15 |
| ENSMUSG00000077493 | 2.65 | 6.27 | 4.30E-04 | NA | NA |
| ENSMUSG00000023191 | 2.65 | 6.26 | 1.81E-28 | Leprel2 | leprecan-like 2 |
| ENSMUSG00000069367 | 2.65 | 6.26 | 3.47E-04 | NA | NA |
| ENSMUSG00000028369 | 2.65 | 6.26 | 8.38E-39 | Svep1 | sushi, von Willebrand factor type A, EGF and pentraxin domain containing 1 |
| ENSMUSG00000077614 | 2.64 | 6.25 | 1.86E-03 | NA | NA |
| ENSMUSG00000042682 | 2.64 | 6.24 | 7.37E-12 | Selk | selenoprotein K |
| ENSMUSG00000065765 | 2.64 | 6.24 | 4.14E-03 | NA | NA |
| ENSMUSG00000030670 | 2.64 | 6.23 | 1.06E-03 | Cyp2r1 | cytochrome P450, family 2, subfamily r, polypeptide 1 |
| ENSMUSG00000084304 | 2.64 | 6.23 | 9.81E-19 | NA | NA |
| ENSMUSG00000054493 | 2.64 | 6.22 | 1.62E-03 | NA | NA |
| ENSMUSG00000024679 | 2.64 | 6.22 | 1.97E-35 | Ms4a6d | membrane-spanning 4-domains, subfamily A, member 6D |
| ENSMUSG00000024521 | 2.63 | 6.20 | 1.30E-17 | Pmaip1 | phorbol-12-myristate-13-acetate-induced protein 1 |
| ENSMUSG00000080540 | 2.63 | 6.19 | 4.06E-03 | NA | NA |
| ENSMUSG00000087303 | 2.63 | 6.18 | 6.79E-06 | NA | NA |
| ENSMUSG00000074908 | 2.63 | 6.18 | 9.85E-08 | NA | NA |
| ENSMUSG00000037358 | 2.63 | 6.17 | 1.14E-10 | 4930578C19Rik | RIKEN cDNA 4930578C19 gene |
| ENSMUSG00000082676 | 2.62 | 6.17 | 3.21E-03 | NA | NA |
| ENSMUSG00000083610 | 2.62 | 6.17 | 3.63E-19 | NA | NA |
| ENSMUSG00000057751 | 2.62 | 6.15 | 5.20E-08 | Megf6 | multiple EGF-like-domains 6 |
| ENSMUSG00000027338 | 2.62 | 6.15 | 3.24E-11 | NA | NA |
| ENSMUSG00000079564 | 2.62 | 6.15 | 3.95E-03 | NA | NA |
| ENSMUSG00000029762 | 2.62 | 6.14 | 7.62E-15 | Akr1b8 | aldo-keto reductase family 1, member B8 |
| ENSMUSG00000074054 | 2.62 | 6.14 | 6.99E-04 | NA | NA |
| ENSMUSG00000048852 | 2.61 | 6.11 | 5.60E-09 | Gm12185 | predicted gene 12185 |
| ENSMUSG00000043342 | 2.61 | 6.11 | 6.33E-06 | Hoxd9 | homeobox D9 |
| ENSMUSG00000085867 | 2.61 | 6.11 | 3.42E-03 | NA | NA |
| ENSMUSG00000054080 | 2.61 | 6.11 | 4.65E-03 | NA | NA |
| ENSMUSG00000021678 | 2.61 | 6.11 | 2.55E-05 | F2rl1 | coagulation factor II (thrombin) receptor-like 1 |
| ENSMUSG00000027864 | 2.61 | 6.09 | 8.76E-22 | Ptgfrn | prostaglandin F2 receptor negative regulator |
| ENSMUSG00000070372 | 2.60 | 6.07 | 6.18E-39 | Capza1 | capping protein (actin filament) muscle Z-line, alpha 1 |
| ENSMUSG00000087413 | 2.60 | 6.07 | 3.77E-08 | NA | NA |
| ENSMUSG00000086283 | 2.60 | 6.06 | 8.15E-05 | NA | NA |
| ENSMUSG00000030281 | 2.59 | 6.04 | 2.13E-13 | Il17rc | interleukin 17 receptor C |
| ENSMUSG00000026042 | 2.59 | 6.03 | 3.91E-33 | Col5a2 | collagen, type V, alpha 2 |
| ENSMUSG00000026536 | 2.59 | 6.02 | 2.96E-23 | Mnda | myeloid cell nuclear differentiation antigen |
| ENSMUSG00000061731 | 2.59 | 6.02 | 2.57E-22 | Ext1 | exostoses (multiple) 1 |
| ENSMUSG00000038415 | 2.58 | 6.00 | 1.34E-06 | Foxq1 | forkhead box Q1 |
| ENSMUSG00000025650 | 2.58 | 6.00 | 3.89E-21 | Col7a1 | collagen, type VII, alpha 1 |
| ENSMUSG00000041046 | 2.58 | 5.99 | 3.23E-10 | Ramp3 | receptor (calcitonin) activity modifying protein 3 |
| ENSMUSG00000056870 | 2.58 | 5.98 | 1.29E-14 | Gulp1 | GULP, engulfment adaptor PTB domain containing 1 |
| ENSMUSG00000040310 | 2.58 | 5.98 | 1.01E-18 | Alx4 | aristaless-like homeobox 4 |
| ENSMUSG00000037813 | 2.58 | 5.98 | 2.72E-20 | D630003M21Rik | RIKEN cDNA D630003M21 gene |
| ENSMUSG00000074917 | 2.58 | 5.97 | 7.47E-11 | NA | NA |
| ENSMUSG00000007613 | 2.58 | 5.97 | 7.08E-67 | Tgfbr1 | transforming growth factor, beta receptor I |
| ENSMUSG00000016487 | 2.58 | 5.96 | 1.70E-29 | Ppfibp1 | PTPRF interacting protein, binding protein 1 (liprin beta 1) |
| ENSMUSG00000070423 | 2.58 | 5.96 | 1.31E-14 | Olfr558 | olfactory receptor 558 |
| ENSMUSG00000038119 | 2.58 | 5.96 | 1.01E-13 | Cdon | cell adhesion molecule-related/down-regulated by oncogenes |
| ENSMUSG00000078633 | 2.57 | 5.96 | 3.86E-05 | NA | NA |
| ENSMUSG00000031489 | 2.57 | 5.95 | 2.61E-05 | Adrb3 | adrenergic receptor, beta 3 |
| ENSMUSG00000081093 | 2.57 | 5.95 | 2.28E-08 | NA | NA |
| ENSMUSG00000081800 | 2.57 | 5.95 | 1.29E-04 | NA | NA |
| ENSMUSG00000064323 | 2.57 | 5.95 | 2.05E-06 | NA | NA |
| ENSMUSG00000031740 | 2.57 | 5.95 | 1.57E-25 | Mmp2 | matrix metallopeptidase 2 |
| ENSMUSG00000022338 | 2.57 | 5.95 | 6.14E-14 | Eny2 | enhancer of yellow 2 homolog (Drosophila) |
| ENSMUSG00000073182 | 2.57 | 5.93 | 5.43E-03 | NA | NA |
| ENSMUSG00000059751 | 2.57 | 5.93 | 1.10E-09 | NA | NA |
| ENSMUSG00000038151 | 2.57 | 5.93 | 1.49E-24 | Prdm1 | PR domain containing 1, with ZNF domain |
| ENSMUSG00000030772 | 2.56 | 5.91 | 1.32E-19 | Dkk3 | dickkopf homolog 3 (Xenopus laevis) |
| ENSMUSG00000024675 | 2.56 | 5.90 | 2.28E-17 | Ms4a4c | membrane-spanning 4-domains, subfamily A, member 4C |
| ENSMUSG00000065359 | 2.56 | 5.90 | 4.73E-04 | NA | NA |
| ENSMUSG00000027603 | 2.56 | 5.90 | 1.82E-25 | Ggt7 | gamma-glutamyltransferase 7 |
| ENSMUSG00000086481 | 2.56 | 5.89 | 4.39E-07 | NA | NA |
| ENSMUSG00000034551 | 2.56 | 5.89 | 6.87E-06 | Hdx | highly divergent homeobox |
| ENSMUSG00000029797 | 2.56 | 5.88 | 1.13E-11 | Sspo | SCO-spondin |
| ENSMUSG00000079801 | 2.56 | 5.88 | 5.65E-03 | NA | NA |
| ENSMUSG00000060487 | 2.56 | 5.88 | 2.58E-05 | Samd5 | sterile alpha motif domain containing 5 |
| ENSMUSG00000022676 | 2.55 | 5.88 | 5.42E-19 | Snai2 | snail homolog 2 (Drosophila) |
| ENSMUSG00000081520 | 2.55 | 5.88 | 1.89E-05 | NA | NA |
| ENSMUSG00000031558 | 2.55 | 5.87 | 6.73E-31 | Slit2 | slit homolog 2 (Drosophila) |
| ENSMUSG00000016529 | 2.55 | 5.86 | 1.47E-06 | Il10 | interleukin 10 |
| ENSMUSG00000023078 | 2.55 | 5.85 | 6.42E-08 | Cxcl13 | chemokine (C-X-C motif) ligand 13 |
| ENSMUSG00000045555 | 2.55 | 5.85 | 3.90E-09 | Mettl24 | methyltransferase like 24 |
| ENSMUSG00000026836 | 2.55 | 5.85 | 1.12E-22 | Acvr1 | activin A receptor, type 1 |
| ENSMUSG00000078851 | 2.55 | 5.85 | 7.52E-07 | Hist3h2a | histone cluster 3, H2a |
| ENSMUSG00000072941 | 2.54 | 5.84 | 1.01E-05 | Sod3 | superoxide dismutase 3, extracellular |
| ENSMUSG00000032925 | 2.54 | 5.83 | 6.96E-11 | Itgbl1 | integrin, beta-like 1 |
| ENSMUSG00000081205 | 2.54 | 5.83 | 1.53E-04 | NA | NA |
| ENSMUSG00000078835 | 2.54 | 5.83 | 5.91E-03 | NA | NA |
| ENSMUSG00000079571 | 2.54 | 5.83 | 5.91E-03 | NA | NA |
| ENSMUSG00000026278 | 2.54 | 5.82 | 1.07E-17 | Bok | BCL2-related ovarian killer protein |
| ENSMUSG00000004296 | 2.54 | 5.82 | 4.55E-04 | Il12b | interleukin 12b |
| ENSMUSG00000081622 | 2.54 | 5.82 | 7.27E-04 | NA | NA |
| ENSMUSG00000075010 | 2.54 | 5.81 | 4.50E-15 | AW112010 | expressed sequence AW112010 |
| ENSMUSG00000024597 | 2.54 | 5.81 | 1.74E-22 | Slc12a2 | solute carrier family 12, member 2 |
| ENSMUSG00000024677 | 2.54 | 5.80 | 1.26E-74 | Ms4a6b | membrane-spanning 4-domains, subfamily A, member 6B |
| ENSMUSG00000030077 | 2.54 | 5.80 | 2.09E-13 | Chl1 | cell adhesion molecule with homology to L1CAM |
| ENSMUSG00000042712 | 2.53 | 5.79 | 5.92E-16 | Wbp5 | WW domain binding protein 5 |
| ENSMUSG00000021318 | 2.53 | 5.79 | 4.12E-19 | Gli3 | GLI-Kruppel family member GLI3 |
| ENSMUSG00000030208 | 2.53 | 5.78 | 1.98E-22 | Emp1 | epithelial membrane protein 1 |
| ENSMUSG00000038390 | 2.53 | 5.78 | 3.81E-27 | Gpr162 | G protein-coupled receptor 162 |
| ENSMUSG00000062196 | 2.53 | 5.77 | 4.88E-05 | NA | NA |
| ENSMUSG00000073210 | 2.53 | 5.77 | 2.18E-06 | NA | NA |
| ENSMUSG00000081833 | 2.53 | 5.76 | 8.18E-06 | NA | NA |
| ENSMUSG00000085752 | 2.53 | 5.76 | 1.43E-04 | NA | NA |
| ENSMUSG00000043263 | 2.52 | 5.74 | 3.47E-28 | Pyhin1 | pyrin and HIN domain family, member 1 |
| ENSMUSG00000082387 | 2.52 | 5.74 | 6.39E-03 | NA | NA |
| ENSMUSG00000037921 | 2.52 | 5.73 | 3.38E-26 | Ddx60 | DEAD (Asp-Glu-Ala-Asp) box polypeptide 60 |
| ENSMUSG00000059022 | 2.52 | 5.73 | 1.29E-12 | Kcp | kielin/chordin-like protein |
| ENSMUSG00000024425 | 2.52 | 5.72 | 5.20E-58 | Ndfip1 | Nedd4 family interacting protein 1 |
| ENSMUSG00000073448 | 2.52 | 5.72 | 1.26E-04 | NA | NA |
| ENSMUSG00000044827 | 2.51 | 5.71 | 9.05E-23 | Tlr1 | toll-like receptor 1 |
| ENSMUSG00000021806 | 2.51 | 5.69 | 4.99E-22 | Nid2 | nidogen 2 |
| ENSMUSG00000049774 | 2.51 | 5.68 | 3.93E-03 | NA | NA |
| ENSMUSG00000064841 | 2.51 | 5.68 | 9.66E-08 | NA | NA |
| ENSMUSG00000063953 | 2.51 | 5.68 | 6.92E-10 | Amd2 | S-adenosylmethionine decarboxylase 2 |
| ENSMUSG00000049511 | 2.51 | 5.68 | 5.80E-03 | Htr1b | 5-hydroxytryptamine (serotonin) receptor 1B |
| ENSMUSG00000031595 | 2.50 | 5.67 | 2.44E-15 | Pdgfrl | platelet-derived growth factor receptor-like |
| ENSMUSG00000032548 | 2.50 | 5.66 | 2.04E-38 | Slco2a1 | solute carrier organic anion transporter family, member 2a1 |
| ENSMUSG00000090053 | 2.50 | 5.65 | 6.92E-03 | Palm2 | paralemmin 2 |
| ENSMUSG00000039878 | 2.50 | 5.65 | 1.43E-03 | Slc39a5 | solute carrier family 39 (metal ion transporter), member 5 |
| ENSMUSG00000008682 | 2.50 | 5.64 | 2.15E-22 | NA | NA |
| ENSMUSG00000035799 | 2.49 | 5.64 | 3.04E-11 | Twist1 | twist basic helix-loop-helix transcription factor 1 |
| ENSMUSG00000025597 | 2.49 | 5.64 | 7.31E-10 | Klhl4 | kelch-like 4 |
| ENSMUSG00000058656 | 2.49 | 5.63 | 2.73E-05 | Samd12 | sterile alpha motif domain containing 12 |
| ENSMUSG00000025658 | 2.49 | 5.61 | 2.11E-04 | Cnksr2 | connector enhancer of kinase suppressor of Ras 2 |
| ENSMUSG00000089681 | 2.49 | 5.61 | 7.05E-03 | NA | NA |
| ENSMUSG00000030905 | 2.49 | 5.61 | 9.95E-07 | Crym | crystallin, mu |
| ENSMUSG00000010830 | 2.49 | 5.61 | 7.65E-20 | Kdelr3 | KDEL (Lys-Asp-Glu-Leu) endoplasmic reticulum protein retention receptor 3 |
| ENSMUSG00000063297 | 2.49 | 5.61 | 1.52E-04 | Luzp2 | leucine zipper protein 2 |
| ENSMUSG00000074158 | 2.49 | 5.61 | 2.32E-04 | 9830147E19Rik | RIKEN cDNA 9830147E19 gene |
| ENSMUSG00000021253 | 2.49 | 5.61 | 3.20E-19 | Tgfb3 | transforming growth factor, beta 3 |
| ENSMUSG00000029669 | 2.49 | 5.61 | 3.59E-33 | Tspan12 | tetraspanin 12 |
| ENSMUSG00000024168 | 2.49 | 5.61 | 6.90E-23 | Tmem204 | transmembrane protein 204 |
| ENSMUSG00000027803 | 2.49 | 5.60 | 1.10E-40 | Wwtr1 | WW domain containing transcription regulator 1 |
| ENSMUSG00000053141 | 2.48 | 5.59 | 4.17E-07 | Ptprt | protein tyrosine phosphatase, receptor type, T |
| ENSMUSG00000085457 | 2.48 | 5.59 | 5.85E-06 | 1110046J04Rik | RIKEN cDNA 1110046J04 gene |
| ENSMUSG00000018800 | 2.48 | 5.59 | 4.50E-15 | Abca5 | ATP-binding cassette, sub-family A (ABC1), member 5 |
| ENSMUSG00000028618 | 2.48 | 5.58 | 6.65E-33 | Tmem59 | transmembrane protein 59 |
| ENSMUSG00000089993 | 2.48 | 5.58 | 8.20E-29 | NA | NA |
| ENSMUSG00000037722 | 2.48 | 5.58 | 1.02E-26 | Gnpnat1 | glucosamine-phosphate N-acetyltransferase 1 |
| ENSMUSG00000084098 | 2.48 | 5.57 | 6.74E-17 | NA | NA |
| ENSMUSG00000078200 | 2.47 | 5.56 | 1.61E-16 | NA | NA |
| ENSMUSG00000064811 | 2.47 | 5.56 | 3.38E-03 | NA | NA |
| ENSMUSG00000063253 | 2.47 | 5.56 | 2.49E-07 | Scoc | short coiled-coil protein |
| ENSMUSG00000059333 | 2.47 | 5.56 | 1.06E-04 | NA | NA |
| ENSMUSG00000003665 | 2.47 | 5.55 | 1.54E-03 | Has1 | hyaluronan synthase1 |
| ENSMUSG00000086513 | 2.47 | 5.55 | 1.80E-10 | NA | NA |
| ENSMUSG00000051048 | 2.47 | 5.55 | 3.78E-14 | P4ha3 | procollagen-proline, 2-oxoglutarate 4-dioxygenase (proline 4-hydroxylase), alpha polypeptide III |
| ENSMUSG00000056836 | 2.47 | 5.55 | 8.26E-21 | NA | NA |
| ENSMUSG00000040606 | 2.47 | 5.55 | 4.53E-22 | Kazn | kazrin, periplakin interacting protein |
| ENSMUSG00000048007 | 2.47 | 5.54 | 1.73E-08 | NA | NA |
| ENSMUSG00000086918 | 2.47 | 5.53 | 1.09E-07 | 4930429F24Rik | RIKEN cDNA 4930429F24 gene |
| ENSMUSG00000050730 | 2.47 | 5.53 | 2.05E-26 | Arhgap42 | Rho GTPase activating protein 42 |
| ENSMUSG00000008813 | 2.46 | 5.52 | 7.79E-03 | Tppp2 | tubulin polymerization-promoting protein family member 2 |
| ENSMUSG00000024620 | 2.46 | 5.52 | 3.60E-38 | Pdgfrb | platelet derived growth factor receptor, beta polypeptide |
| ENSMUSG00000031616 | 2.46 | 5.52 | 1.77E-31 | Ednra | endothelin receptor type A |
| ENSMUSG00000074183 | 2.46 | 5.52 | 7.53E-03 | NA | NA |
| ENSMUSG00000042451 | 2.46 | 5.52 | 1.12E-03 | Mybph | myosin binding protein H |
| ENSMUSG00000078121 | 2.46 | 5.51 | 7.84E-03 | NA | NA |
| ENSMUSG00000028487 | 2.46 | 5.50 | 2.38E-10 | Bnc2 | basonuclin 2 |
| ENSMUSG00000033350 | 2.46 | 5.50 | 2.04E-22 | Chst2 | carbohydrate sulfotransferase 2 |
| ENSMUSG00000035274 | 2.46 | 5.48 | 1.95E-14 | Tpbg | trophoblast glycoprotein |
| ENSMUSG00000023232 | 2.45 | 5.48 | 1.58E-12 | Serinc2 | serine incorporator 2 |
| ENSMUSG00000024440 | 2.45 | 5.48 | 1.35E-30 | Pcdh12 | protocadherin 12 |
| ENSMUSG00000043631 | 2.45 | 5.47 | 3.27E-13 | Ecm2 | extracellular matrix protein 2, female organ and adipocyte specific |
| ENSMUSG00000005952 | 2.45 | 5.47 | 1.91E-04 | Trpv1 | transient receptor potential cation channel, subfamily V, member 1 |
| ENSMUSG00000043366 | 2.45 | 5.47 | 1.04E-10 | Olfr78 | olfactory receptor 78 |
| ENSMUSG00000078648 | 2.45 | 5.47 | 8.11E-03 | NA | NA |
| ENSMUSG00000026764 | 2.45 | 5.47 | 4.68E-08 | Kif5c | kinesin family member 5C |
| ENSMUSG00000060519 | 2.45 | 5.47 | 9.43E-42 | Tor3a | torsin family 3, member A |
| ENSMUSG00000040187 | 2.45 | 5.46 | 8.66E-12 | Arntl2 | aryl hydrocarbon receptor nuclear translocator-like 2 |
| ENSMUSG00000080928 | 2.45 | 5.46 | 7.91E-03 | NA | NA |
| ENSMUSG00000055137 | 2.45 | 5.45 | 1.33E-04 | Sugct | succinyl-CoA glutarate-CoA transferase |
| ENSMUSG00000029185 | 2.45 | 5.45 | 3.32E-31 | Fam114a1 | family with sequence similarity 114, member A1 |
| ENSMUSG00000066113 | 2.45 | 5.45 | 3.16E-16 | Adamtsl1 | ADAMTS-like 1 |
| ENSMUSG00000000359 | 2.44 | 5.44 | 2.64E-11 | Rem1 | rad and gem related GTP binding protein 1 |
| ENSMUSG00000043542 | 2.44 | 5.43 | 1.98E-27 | Zc2hc1a | zinc finger, C2HC-type containing 1A |
| ENSMUSG00000081778 | 2.44 | 5.43 | 6.52E-04 | NA | NA |
| ENSMUSG00000049047 | 2.44 | 5.42 | 7.59E-32 | Armcx3 | armadillo repeat containing, X-linked 3 |
| ENSMUSG00000001661 | 2.44 | 5.42 | 5.28E-10 | Hoxc6 | homeobox C6 |
| ENSMUSG00000041801 | 2.44 | 5.42 | 6.52E-18 | Phlda3 | pleckstrin homology-like domain, family A, member 3 |
| ENSMUSG00000034777 | 2.44 | 5.41 | 7.27E-03 | Vax2 | ventral anterior homeobox 2 |
| ENSMUSG00000021306 | 2.43 | 5.40 | 4.82E-23 | Gpr137b | G protein-coupled receptor 137B |
| ENSMUSG00000026443 | 2.43 | 5.40 | 6.29E-05 | Lrrn2 | leucine rich repeat protein 2, neuronal |
| ENSMUSG00000078295 | 2.43 | 5.40 | 8.43E-03 | NA | NA |
| ENSMUSG00000089628 | 2.43 | 5.39 | 2.18E-05 | NA | NA |
| ENSMUSG00000024072 | 2.43 | 5.39 | 3.08E-22 | Yipf4 | Yip1 domain family, member 4 |
| ENSMUSG00000032452 | 2.43 | 5.38 | 5.37E-05 | Clstn2 | calsyntenin 2 |
| ENSMUSG00000064917 | 2.43 | 5.38 | 8.75E-03 | NA | NA |
| ENSMUSG00000031561 | 2.42 | 5.36 | 1.12E-12 | Tenm3 | teneurin transmembrane protein 3 |
| ENSMUSG00000033565 | 2.42 | 5.36 | 3.69E-20 | Rbfox2 | RNA binding protein, fox-1 homolog (C. elegans) 2 |
| ENSMUSG00000083737 | 2.42 | 5.36 | 9.09E-03 | NA | NA |
| ENSMUSG00000035914 | 2.42 | 5.35 | 6.63E-19 | Cd276 | CD276 antigen |
| ENSMUSG00000020181 | 2.42 | 5.35 | 3.08E-15 | Nav3 | neuron navigator 3 |
| ENSMUSG00000078794 | 2.42 | 5.35 | 1.37E-09 | Dact3 | dapper homolog 3, antagonist of beta-catenin (xenopus) |
| ENSMUSG00000040121 | 2.42 | 5.35 | 3.82E-05 | Rep15 | RAB15 effector protein |
| ENSMUSG00000046402 | 2.42 | 5.34 | 1.51E-15 | Rbp1 | retinol binding protein 1, cellular |
| ENSMUSG00000021643 | 2.42 | 5.34 | 2.26E-13 | Serf1 | small EDRK-rich factor 1 |
| ENSMUSG00000014782 | 2.42 | 5.34 | 7.62E-14 | Plekhg4 | pleckstrin homology domain containing, family G (with RhoGef domain) member 4 |
| ENSMUSG00000073565 | 2.42 | 5.34 | 1.66E-12 | Prr16 | proline rich 16 |
| ENSMUSG00000055801 | 2.42 | 5.33 | 9.17E-03 | NA | NA |
| ENSMUSG00000078427 | 2.41 | 5.33 | 5.32E-14 | NA | NA |
| ENSMUSG00000038624 | 2.41 | 5.33 | 6.92E-03 | Nepn | nephrocan |
| ENSMUSG00000026418 | 2.41 | 5.33 | 5.92E-05 | Tnni1 | troponin I, skeletal, slow 1 |
| ENSMUSG00000026574 | 2.41 | 5.33 | 1.17E-05 | Dpt | dermatopontin |
| ENSMUSG00000051331 | 2.41 | 5.33 | 1.07E-10 | Cacna1c | calcium channel, voltage-dependent, L type, alpha 1C subunit |
| ENSMUSG00000065336 | 2.41 | 5.33 | 1.58E-05 | NA | NA |
| ENSMUSG00000080840 | 2.41 | 5.32 | 1.17E-06 | NA | NA |
| ENSMUSG00000085698 | 2.41 | 5.32 | 3.10E-03 | NA | NA |
| ENSMUSG00000068798 | 2.41 | 5.32 | 3.06E-20 | Rap1a | RAS-related protein-1a |
| ENSMUSG00000081199 | 2.41 | 5.32 | 3.86E-08 | NA | NA |
| ENSMUSG00000021217 | 2.41 | 5.31 | 4.80E-23 | Tshz3 | teashirt zinc finger family member 3 |
| ENSMUSG00000065480 | 2.41 | 5.31 | 9.28E-03 | Mir133b | microRNA 133b |
| ENSMUSG00000084952 | 2.41 | 5.31 | 7.32E-03 | NA | NA |
| ENSMUSG00000024726 | 2.41 | 5.30 | 5.59E-06 | 2410127L17Rik | RIKEN cDNA 2410127L17 gene |
| ENSMUSG00000079255 | 2.40 | 5.29 | 8.83E-03 | NA | NA |
| ENSMUSG00000035305 | 2.40 | 5.29 | 1.39E-12 | Ror1 | receptor tyrosine kinase-like orphan receptor 1 |
| ENSMUSG00000050150 | 2.40 | 5.28 | 1.36E-03 | Slc9b1 | solute carrier family 9, subfamily B (NHA1, cation proton antiporter 1), member 1 |
| ENSMUSG00000039252 | 2.40 | 5.28 | 6.60E-06 | Lgi2 | leucine-rich repeat LGI family, member 2 |
| ENSMUSG00000039985 | 2.40 | 5.27 | 1.30E-21 | Fam60a | family with sequence similarity 60, member A |
| ENSMUSG00000020826 | 2.40 | 5.27 | 1.66E-06 | Nos2 | nitric oxide synthase 2, inducible |
| ENSMUSG00000008136 | 2.40 | 5.26 | 3.78E-17 | Fhl2 | four and a half LIM domains 2 |
| ENSMUSG00000068122 | 2.40 | 5.26 | 8.05E-03 | Agtr2 | angiotensin II receptor, type 2 |
| ENSMUSG00000083757 | 2.40 | 5.26 | 3.25E-09 | NA | NA |
| ENSMUSG00000038244 | 2.39 | 5.26 | 3.38E-28 | Mical2 | microtubule associated monooxygenase, calponin and LIM domain containing 2 |
| ENSMUSG00000079699 | 2.39 | 5.26 | 9.73E-03 | Gm6592 | predicted gene 6592 |
| ENSMUSG00000081920 | 2.39 | 5.25 | 5.34E-04 | NA | NA |
| ENSMUSG00000040717 | 2.39 | 5.25 | 4.89E-14 | Il17rd | interleukin 17 receptor D |
| ENSMUSG00000002265 | 2.39 | 5.25 | 4.99E-15 | Peg3 | paternally expressed 3 |
| ENSMUSG00000011752 | 2.39 | 5.24 | 1.61E-14 | Pgam1 | phosphoglycerate mutase 1 |
| ENSMUSG00000085238 | 2.39 | 5.24 | 9.50E-03 | 4930479D17Rik | RIKEN cDNA 4930479D17 gene |
| ENSMUSG00000031853 | 2.39 | 5.24 | 1.90E-04 | BC021891 | cDNA sequence BC021891 |
| ENSMUSG00000087912 | 2.39 | 5.24 | 9.90E-03 | NA | NA |
| ENSMUSG00000000159 | 2.39 | 5.23 | 4.45E-07 | Igsf5 | immunoglobulin superfamily, member 5 |
| ENSMUSG00000089800 | 2.39 | 5.23 | 7.12E-03 | NA | NA |
| ENSMUSG00000024535 | 2.39 | 5.22 | 3.38E-24 | Snx24 | sorting nexing 24 |
| ENSMUSG00000053524 | 2.38 | 5.22 | 7.46E-03 | NA | NA |
| ENSMUSG00000078616 | 2.38 | 5.21 | 3.31E-09 | Trim30c | tripartite motif-containing 30C |
| ENSMUSG00000060935 | 2.38 | 5.20 | 5.70E-58 | AI597468 | expressed sequence AI597468 |
| ENSMUSG00000024727 | 2.38 | 5.19 | 3.35E-08 | Trpm6 | transient receptor potential cation channel, subfamily M, member 6 |
| ENSMUSG00000086382 | 2.37 | 5.19 | 1.29E-04 | Chrna1os | cholinergic receptor, nicotinic, alpha polypeptide 1 (muscle), opposite strand |
| ENSMUSG00000084319 | 2.37 | 5.19 | 9.60E-18 | NA | NA |
| ENSMUSG00000050505 | 2.37 | 5.18 | 1.38E-03 | Pcdh20 | protocadherin 20 |
| ENSMUSG00000038721 | 2.37 | 5.18 | 1.59E-08 | Hoxb7 | homeobox B7 |
| ENSMUSG00000068545 | 2.37 | 5.17 | 1.98E-03 | NA | NA |
| ENSMUSG00000029126 | 2.37 | 5.17 | 5.28E-24 | Nsg1 | neuron specific gene family member 1 |
| ENSMUSG00000072812 | 2.37 | 5.16 | 8.73E-10 | Ahnak2 | AHNAK nucleoprotein 2 |
| ENSMUSG00000067106 | 2.37 | 5.15 | 2.03E-08 | NA | NA |
| ENSMUSG00000006638 | 2.37 | 5.15 | 1.44E-03 | Abhd1 | abhydrolase domain containing 1 |
| ENSMUSG00000049281 | 2.36 | 5.15 | 1.05E-05 | Scn3b | sodium channel, voltage-gated, type III, beta |
| ENSMUSG00000055849 | 2.36 | 5.15 | 6.36E-17 | NA | NA |
| ENSMUSG00000027832 | 2.36 | 5.13 | 2.10E-24 | Ptx3 | pentraxin related gene |
| ENSMUSG00000001025 | 2.36 | 5.13 | 5.69E-22 | S100a6 | S100 calcium binding protein A6 (calcyclin) |
| ENSMUSG00000046623 | 2.36 | 5.12 | 5.98E-03 | Gjb4 | gap junction protein, beta 4 |
| ENSMUSG00000024371 | 2.36 | 5.12 | 1.42E-23 | C2 | complement component 2 (within H-2S) |
| ENSMUSG00000038112 | 2.36 | 5.12 | 3.72E-07 | AW551984 | expressed sequence AW551984 |
| ENSMUSG00000004473 | 2.36 | 5.12 | 1.64E-21 | Clec11a | C-type lectin domain family 11, member a |
| ENSMUSG00000029814 | 2.36 | 5.12 | 3.46E-07 | Igf2bp3 | insulin-like growth factor 2 mRNA binding protein 3 |
| ENSMUSG00000082087 | 2.36 | 5.12 | 2.51E-07 | NA | NA |
| ENSMUSG00000087579 | 2.35 | 5.11 | 6.14E-07 | 1500017E21Rik | RIKEN cDNA 1500017E21 gene |
| ENSMUSG00000047996 | 2.35 | 5.11 | 1.40E-16 | Prrg1 | proline rich Gla (G-carboxyglutamic acid) 1 |
| ENSMUSG00000036667 | 2.35 | 5.11 | 6.00E-25 | Fam115a | family with sequence similarity 115, member A |
| ENSMUSG00000036256 | 2.35 | 5.11 | 4.43E-34 | Igfbp7 | insulin-like growth factor binding protein 7 |
| ENSMUSG00000086416 | 2.35 | 5.10 | 1.04E-03 | NA | NA |
| ENSMUSG00000029390 | 2.35 | 5.10 | 6.91E-34 | NA | NA |
| ENSMUSG00000073074 | 2.35 | 5.09 | 1.06E-04 | NA | NA |
| ENSMUSG00000018263 | 2.35 | 5.09 | 4.84E-03 | Tbx5 | T-box 5 |
| ENSMUSG00000081631 | 2.35 | 5.09 | 1.10E-07 | NA | NA |
| ENSMUSG00000038642 | 2.35 | 5.09 | 8.63E-53 | Ctss | cathepsin S |
| ENSMUSG00000035279 | 2.35 | 5.09 | 2.07E-07 | Ssc5d | scavenger receptor cysteine rich domain containing (5 domains) |
| ENSMUSG00000049939 | 2.35 | 5.09 | 1.30E-09 | Lrrc4 | leucine rich repeat containing 4 |
| ENSMUSG00000038400 | 2.35 | 5.08 | 2.02E-21 | Pmepa1 | prostate transmembrane protein, androgen induced 1 |
| ENSMUSG00000081323 | 2.35 | 5.08 | 2.96E-05 | NA | NA |
| ENSMUSG00000078247 | 2.34 | 5.08 | 5.99E-13 | Airn | antisense Igf2r RNA |
| ENSMUSG00000026826 | 2.34 | 5.08 | 1.71E-13 | Nr4a2 | nuclear receptor subfamily 4, group A, member 2 |
| ENSMUSG00000020080 | 2.34 | 5.07 | 5.82E-04 | Hkdc1 | hexokinase domain containing 1 |
| ENSMUSG00000027676 | 2.34 | 5.07 | 2.52E-08 | Ccdc39 | coiled-coil domain containing 39 |
| ENSMUSG00000020122 | 2.34 | 5.07 | 3.10E-42 | Egfr | epidermal growth factor receptor |
| ENSMUSG00000050503 | 2.34 | 5.07 | 1.16E-06 | Fbxl22 | F-box and leucine-rich repeat protein 22 |
| ENSMUSG00000024397 | 2.34 | 5.06 | 1.54E-21 | Aif1 | allograft inflammatory factor 1 |
| ENSMUSG00000028691 | 2.34 | 5.06 | 1.78E-20 | NA | NA |
| ENSMUSG00000018417 | 2.34 | 5.06 | 6.84E-28 | Myo1b | myosin IB |
| ENSMUSG00000086301 | 2.34 | 5.06 | 5.98E-04 | NA | NA |
| ENSMUSG00000021760 | 2.34 | 5.05 | 1.03E-10 | Gpx8 | glutathione peroxidase 8 (putative) |
| ENSMUSG00000038646 | 2.34 | 5.05 | 8.97E-10 | NA | NA |
| ENSMUSG00000024538 | 2.34 | 5.05 | 3.12E-22 | Ppic | peptidylprolyl isomerase C |
| ENSMUSG00000020650 | 2.34 | 5.05 | 2.55E-19 | Bcap29 | B cell receptor associated protein 29 |
| ENSMUSG00000085334 | 2.33 | 5.04 | 1.33E-15 | NA | NA |
| ENSMUSG00000049517 | 2.33 | 5.04 | 5.81E-08 | NA | NA |
| ENSMUSG00000049866 | 2.33 | 5.04 | 5.98E-23 | Arl4c | ADP-ribosylation factor-like 4C |
| ENSMUSG00000036782 | 2.33 | 5.03 | 6.01E-17 | Klhl13 | kelch-like 13 |
| ENSMUSG00000056679 | 2.33 | 5.03 | 2.12E-13 | Gpr173 | G-protein coupled receptor 173 |
| ENSMUSG00000031253 | 2.33 | 5.03 | 9.28E-19 | Srpx2 | sushi-repeat-containing protein, X-linked 2 |
| ENSMUSG00000052371 | 2.33 | 5.03 | 1.70E-04 | NA | NA |
| ENSMUSG00000062270 | 2.33 | 5.03 | 1.49E-14 | NA | NA |
| ENSMUSG00000074968 | 2.33 | 5.03 | 5.69E-03 | Ano3 | anoctamin 3 |
| ENSMUSG00000026424 | 2.33 | 5.02 | 4.73E-06 | Gpr37l1 | G protein-coupled receptor 37-like 1 |
| ENSMUSG00000080850 | 2.33 | 5.02 | 2.79E-04 | NA | NA |
| ENSMUSG00000019590 | 2.33 | 5.02 | 4.53E-19 | Cyb561 | cytochrome b-561 |
| ENSMUSG00000027827 | 2.33 | 5.02 | 5.85E-16 | Kcnab1 | potassium voltage-gated channel, shaker-related subfamily, beta member 1 |
| ENSMUSG00000035681 | 2.33 | 5.01 | 1.37E-05 | Kcnc2 | potassium voltage gated channel, Shaw-related subfamily, member 2 |
| ENSMUSG00000078087 | 2.32 | 5.01 | 7.71E-05 | NA | NA |
| ENSMUSG00000020875 | 2.32 | 5.00 | 2.56E-04 | Hoxb9 | homeobox B9 |
| ENSMUSG00000088929 | 2.32 | 5.00 | 3.12E-06 | NA | NA |
| ENSMUSG00000039323 | 2.32 | 5.00 | 1.04E-13 | Igfbp2 | insulin-like growth factor binding protein 2 |
| ENSMUSG00000038560 | 2.32 | 5.00 | 4.70E-04 | Sp6 | trans-acting transcription factor 6 |
| ENSMUSG00000006651 | 2.32 | 4.99 | 2.46E-16 | Aplp1 | amyloid beta (A4) precursor-like protein 1 |
| ENSMUSG00000036305 | 2.32 | 4.99 | 1.30E-18 | NA | NA |
| ENSMUSG00000081855 | 2.32 | 4.98 | 2.57E-06 | NA | NA |
| ENSMUSG00000040584 | 2.32 | 4.98 | 2.87E-33 | Abcb1a | ATP-binding cassette, sub-family B (MDR/TAP), member 1A |
| ENSMUSG00000050621 | 2.32 | 4.98 | 2.30E-03 | NA | NA |
| ENSMUSG00000027660 | 2.31 | 4.97 | 1.27E-24 | Skil | SKI-like |
| ENSMUSG00000052776 | 2.31 | 4.97 | 7.19E-19 | Oas1a | 2'-5' oligoadenylate synthetase 1A |
| ENSMUSG00000078771 | 2.31 | 4.97 | 5.68E-28 | Evi2a | ecotropic viral integration site 2a |
| ENSMUSG00000084407 | 2.31 | 4.97 | 3.91E-03 | NA | NA |
| ENSMUSG00000085494 | 2.31 | 4.97 | 2.66E-07 | NA | NA |
| ENSMUSG00000072902 | 2.31 | 4.97 | 3.65E-04 | NA | NA |
| ENSMUSG00000084808 | 2.31 | 4.97 | 1.17E-11 | NA | NA |
| ENSMUSG00000005397 | 2.31 | 4.96 | 1.71E-10 | Nid1 | nidogen 1 |
| ENSMUSG00000085988 | 2.31 | 4.96 | 1.78E-13 | NA | NA |
| ENSMUSG00000046031 | 2.31 | 4.96 | 3.87E-09 | Fam26f | family with sequence similarity 26, member F |
| ENSMUSG00000034161 | 2.31 | 4.95 | 1.21E-15 | Scx | scleraxis |
| ENSMUSG00000041390 | 2.31 | 4.95 | 1.04E-23 | Mdfic | MyoD family inhibitor domain containing |
| ENSMUSG00000031138 | 2.31 | 4.95 | 6.45E-05 | F9 | coagulation factor IX |
| ENSMUSG00000021589 | 2.31 | 4.94 | 4.22E-24 | Rhobtb3 | Rho-related BTB domain containing 3 |
| ENSMUSG00000042284 | 2.31 | 4.94 | 1.86E-22 | Itga1 | integrin alpha 1 |
| ENSMUSG00000019975 | 2.30 | 4.94 | 1.29E-15 | Ikbip | IKBKB interacting protein |
| ENSMUSG00000021775 | 2.30 | 4.94 | 4.74E-15 | Nr1d2 | nuclear receptor subfamily 1, group D, member 2 |
| ENSMUSG00000051065 | 2.30 | 4.93 | 5.06E-17 | Mb21d2 | Mab-21 domain containing 2 |
| ENSMUSG00000058153 | 2.30 | 4.93 | 2.68E-04 | Sez6l | seizure related 6 homolog like |
| ENSMUSG00000089361 | 2.30 | 4.93 | 7.37E-03 | NA | NA |
| ENSMUSG00000088651 | 2.30 | 4.93 | 1.00E-07 | NA | NA |
| ENSMUSG00000081673 | 2.30 | 4.93 | 1.97E-07 | NA | NA |
| ENSMUSG00000072623 | 2.30 | 4.92 | 6.93E-17 | Zfp9 | zinc finger protein 9 |
| ENSMUSG00000010529 | 2.30 | 4.92 | 3.99E-05 | Gm266 | predicted gene 266 |
| ENSMUSG00000044548 | 2.30 | 4.91 | 1.18E-20 | Dact1 | dapper homolog 1, antagonist of beta-catenin (xenopus) |
| ENSMUSG00000045763 | 2.30 | 4.91 | 3.81E-19 | Basp1 | brain abundant, membrane attached signal protein 1 |
| ENSMUSG00000052364 | 2.30 | 4.91 | 1.81E-09 | B630019K06Rik | novel protein similar to F-box and leucine-rich repeat protein 17 (Fbxl17) |
| ENSMUSG00000088632 | 2.30 | 4.91 | 4.11E-03 | NA | NA |
| ENSMUSG00000029581 | 2.29 | 4.90 | 2.27E-12 | Fscn1 | fascin homolog 1, actin bundling protein (Strongylocentrotus purpuratus) |
| ENSMUSG00000029309 | 2.29 | 4.90 | 1.81E-10 | Sparcl1 | SPARC-like 1 |
| ENSMUSG00000047213 | 2.29 | 4.90 | 2.08E-19 | Ythdf3 | YTH domain family 3 |
| ENSMUSG00000084131 | 2.29 | 4.89 | 1.08E-05 | NA | NA |
| ENSMUSG00000034040 | 2.29 | 4.89 | 5.43E-13 | Wbscr17 | Williams-Beuren syndrome chromosome region 17 homolog (human) |
| ENSMUSG00000069208 | 2.29 | 4.88 | 1.10E-12 | Zfp825 | zinc finger protein 825 |
| ENSMUSG00000084350 | 2.29 | 4.88 | 1.89E-05 | NA | NA |
| ENSMUSG00000025245 | 2.29 | 4.87 | 7.99E-29 | Lztfl1 | leucine zipper transcription factor-like 1 |
| ENSMUSG00000050700 | 2.28 | 4.87 | 5.63E-08 | Emilin3 | elastin microfibril interfacer 3 |
| ENSMUSG00000024696 | 2.28 | 4.87 | 6.66E-21 | Lpxn | leupaxin |
| ENSMUSG00000053113 | 2.28 | 4.86 | 6.00E-17 | Socs3 | suppressor of cytokine signaling 3 |
| ENSMUSG00000040323 | 2.28 | 4.86 | 9.19E-16 | NA | NA |
| ENSMUSG00000039997 | 2.28 | 4.86 | 1.83E-34 | NA | NA |
| ENSMUSG00000016382 | 2.28 | 4.85 | 1.60E-17 | Pls3 | plastin 3 (T-isoform) |
| ENSMUSG00000083674 | 2.28 | 4.85 | 1.78E-04 | NA | NA |
| ENSMUSG00000027210 | 2.28 | 4.84 | 2.28E-06 | Meis2 | Meis homeobox 2 |
| ENSMUSG00000075012 | 2.27 | 4.83 | 4.36E-09 | Fjx1 | four jointed box 1 (Drosophila) |
| ENSMUSG00000029334 | 2.27 | 4.83 | 1.09E-03 | Prkg2 | protein kinase, cGMP-dependent, type II |
| ENSMUSG00000024145 | 2.27 | 4.83 | 2.45E-14 | Pigf | phosphatidylinositol glycan anchor biosynthesis, class F |
| ENSMUSG00000079428 | 2.27 | 4.83 | 8.63E-06 | Tceal7 | transcription elongation factor A (SII)-like 7 |
| ENSMUSG00000043164 | 2.27 | 4.82 | 5.61E-03 | Tmem212 | transmembrane protein 212 |
| ENSMUSG00000047910 | 2.27 | 4.82 | 4.70E-13 | Pcdhb16 | protocadherin beta 16 |
| ENSMUSG00000060961 | 2.27 | 4.82 | 9.15E-11 | Slc4a4 | solute carrier family 4 (anion exchanger), member 4 |
| ENSMUSG00000047963 | 2.27 | 4.82 | 6.79E-17 | Stbd1 | starch binding domain 1 |
| ENSMUSG00000026342 | 2.27 | 4.82 | 1.18E-36 | Slc35f5 | solute carrier family 35, member F5 |
| ENSMUSG00000076258 | 2.27 | 4.82 | 1.01E-03 | NA | NA |
| ENSMUSG00000087006 | 2.27 | 4.81 | 1.00E-06 | NA | NA |
| ENSMUSG00000083554 | 2.27 | 4.81 | 1.40E-05 | NA | NA |
| ENSMUSG00000028194 | 2.27 | 4.81 | 9.05E-10 | Ddah1 | dimethylarginine dimethylaminohydrolase 1 |
| ENSMUSG00000059301 | 2.27 | 4.81 | 9.52E-04 | Gm5434 | ubiquitin-conjugating enzyme E2F (putative) pseudogene |
| ENSMUSG00000052922 | 2.27 | 4.81 | 6.23E-03 | Bpi | bactericidal permeablility increasing protein |
| ENSMUSG00000029298 | 2.26 | 4.80 | 2.02E-19 | Gbp9 | guanylate-binding protein 9 |
| ENSMUSG00000085611 | 2.26 | 4.80 | 6.73E-22 | NA | NA |
| ENSMUSG00000055254 | 2.26 | 4.79 | 4.69E-10 | Ntrk2 | neurotrophic tyrosine kinase, receptor, type 2 |
| ENSMUSG00000043614 | 2.26 | 4.79 | 1.87E-08 | Vps37d | vacuolar protein sorting 37D (yeast) |
| ENSMUSG00000078863 | 2.26 | 4.78 | 5.93E-13 | NA | NA |
| ENSMUSG00000027314 | 2.26 | 4.78 | 6.68E-24 | Dll4 | delta-like 4 (Drosophila) |
| ENSMUSG00000014813 | 2.26 | 4.77 | 2.05E-06 | Stc1 | stanniocalcin 1 |
| ENSMUSG00000053205 | 2.25 | 4.77 | 9.39E-08 | NA | NA |
| ENSMUSG00000078496 | 2.25 | 4.77 | 1.44E-10 | Gm13152 | predicted gene 13152 |
| ENSMUSG00000048612 | 2.25 | 4.76 | 2.92E-23 | Myof | myoferlin |
| ENSMUSG00000043895 | 2.25 | 4.76 | 1.07E-19 | S1pr2 | sphingosine-1-phosphate receptor 2 |
| ENSMUSG00000032528 | 2.25 | 4.76 | 4.12E-07 | Vipr1 | vasoactive intestinal peptide receptor 1 |
| ENSMUSG00000037375 | 2.25 | 4.75 | 5.58E-10 | Hhat | hedgehog acyltransferase |
| ENSMUSG00000065599 | 2.25 | 4.75 | 4.01E-03 | NA | NA |
| ENSMUSG00000063406 | 2.25 | 4.75 | 7.94E-23 | Tmed5 | transmembrane emp24 protein transport domain containing 5 |
| ENSMUSG00000028019 | 2.25 | 4.74 | 9.11E-24 | Pdgfc | platelet-derived growth factor, C polypeptide |
| ENSMUSG00000044807 | 2.24 | 4.74 | 6.41E-25 | Zfp354c | zinc finger protein 354C |
| ENSMUSG00000015957 | 2.24 | 4.73 | 1.65E-06 | Wnt11 | wingless-related MMTV integration site 11 |
| ENSMUSG00000050714 | 2.24 | 4.73 | 5.44E-17 | Zbtb26 | zinc finger and BTB domain containing 26 |
| ENSMUSG00000042357 | 2.24 | 4.73 | 6.29E-03 | Gjb5 | gap junction protein, beta 5 |
| ENSMUSG00000082394 | 2.24 | 4.73 | 8.28E-14 | NA | NA |
| ENSMUSG00000020155 | 2.24 | 4.72 | 2.04E-08 | Kcnmb1 | potassium large conductance calcium-activated channel, subfamily M, beta member 1 |
| ENSMUSG00000043903 | 2.24 | 4.72 | 3.94E-19 | Gm22 | predicted gene 22 |
| ENSMUSG00000073530 | 2.24 | 4.71 | 2.98E-07 | Pappa2 | pappalysin 2 |
| ENSMUSG00000044933 | 2.24 | 4.71 | 4.11E-03 | Sstr3 | somatostatin receptor 3 |
| ENSMUSG00000089991 | 2.23 | 4.71 | 5.40E-06 | NA | NA |
| ENSMUSG00000070407 | 2.23 | 4.70 | 2.79E-10 | Hs3st3b1 | heparan sulfate (glucosamine) 3-O-sulfotransferase 3B1 |
| ENSMUSG00000031298 | 2.23 | 4.70 | 4.86E-09 | Gpr64 | G protein-coupled receptor 64 |
| ENSMUSG00000028111 | 2.23 | 4.70 | 2.48E-10 | Ctsk | cathepsin K |
| ENSMUSG00000019718 | 2.23 | 4.70 | 2.48E-13 | L3hypdh | L-3-hydroxyproline dehydratase (trans-) |
| ENSMUSG00000087511 | 2.23 | 4.70 | 1.47E-07 | NA | NA |
| ENSMUSG00000054675 | 2.23 | 4.70 | 1.50E-23 | Tmem119 | transmembrane protein 119 |
| ENSMUSG00000037440 | 2.23 | 4.70 | 2.61E-04 | Vnn1 | vanin 1 |
| ENSMUSG00000079671 | 2.23 | 4.69 | 4.78E-07 | 2610203C22Rik | RIKEN cDNA 2610203C22 gene |
| ENSMUSG00000025498 | 2.23 | 4.69 | 4.50E-17 | Irf7 | interferon regulatory factor 7 |
| ENSMUSG00000064390 | 2.23 | 4.68 | 3.38E-03 | NA | NA |
| ENSMUSG00000032243 | 2.23 | 4.68 | 2.75E-27 | Itga11 | integrin alpha 11 |
| ENSMUSG00000045414 | 2.22 | 4.67 | 1.03E-24 | 1190002N15Rik | RIKEN cDNA 1190002N15 gene |
| ENSMUSG00000021733 | 2.22 | 4.67 | 4.58E-24 | Slc4a7 | solute carrier family 4, sodium bicarbonate cotransporter, member 7 |
| ENSMUSG00000043313 | 2.22 | 4.66 | 2.33E-06 | Pcdhb19 | protocadherin beta 19 |
| ENSMUSG00000030917 | 2.22 | 4.66 | 1.73E-20 | Tmem159 | transmembrane protein 159 |
| ENSMUSG00000026686 | 2.22 | 4.66 | 2.84E-04 | Lmx1a | LIM homeobox transcription factor 1 alpha |
| ENSMUSG00000084115 | 2.22 | 4.66 | 1.53E-03 | NA | NA |
| ENSMUSG00000074040 | 2.22 | 4.66 | 8.87E-03 | NA | NA |
| ENSMUSG00000086786 | 2.22 | 4.66 | 1.66E-03 | LOC101056149 | uncharacterized LOC101056149 |
| ENSMUSG00000033925 | 2.22 | 4.65 | 1.03E-03 | NA | NA |
| ENSMUSG00000032064 | 2.22 | 4.65 | 5.55E-14 | Dixdc1 | DIX domain containing 1 |
| ENSMUSG00000058743 | 2.22 | 4.65 | 2.77E-10 | Kcnj14 | potassium inwardly-rectifying channel, subfamily J, member 14 |
| ENSMUSG00000028597 | 2.21 | 4.64 | 9.17E-18 | Gpx7 | glutathione peroxidase 7 |
| ENSMUSG00000083019 | 2.21 | 4.64 | 1.08E-04 | NA | NA |
| ENSMUSG00000058816 | 2.21 | 4.63 | 6.33E-09 | NA | NA |
| ENSMUSG00000075318 | 2.21 | 4.63 | 7.01E-07 | Scn2a1 | sodium channel, voltage-gated, type II, alpha 1 |
| ENSMUSG00000034462 | 2.21 | 4.62 | 2.11E-18 | Pkd2 | polycystic kidney disease 2 |
| ENSMUSG00000070867 | 2.21 | 4.62 | 2.02E-11 | Trabd2b | TraB domain containing 2B |
| ENSMUSG00000020676 | 2.21 | 4.61 | 4.17E-09 | Ccl11 | chemokine (C-C motif) ligand 11 |
| ENSMUSG00000026866 | 2.21 | 4.61 | 1.06E-18 | Kynu | kynureninase (L-kynurenine hydrolase) |
| ENSMUSG00000016262 | 2.21 | 4.61 | 1.73E-09 | Sertad4 | SERTA domain containing 4 |
| ENSMUSG00000033287 | 2.20 | 4.61 | 2.31E-13 | Kctd17 | potassium channel tetramerisation domain containing 17 |
| ENSMUSG00000020607 | 2.20 | 4.61 | 5.97E-07 | Fam84a | family with sequence similarity 84, member A |
| ENSMUSG00000031554 | 2.20 | 4.61 | 3.06E-07 | Adam5 | a disintegrin and metallopeptidase domain 5 |
| ENSMUSG00000028214 | 2.20 | 4.60 | 3.88E-12 | Gem | GTP binding protein (gene overexpressed in skeletal muscle) |
| ENSMUSG00000027955 | 2.20 | 4.60 | 1.50E-14 | Fam198b | family with sequence similarity 198, member B |
| ENSMUSG00000023972 | 2.20 | 4.60 | 5.66E-18 | Ptk7 | PTK7 protein tyrosine kinase 7 |
| ENSMUSG00000000416 | 2.20 | 4.60 | 1.05E-16 | Cttnbp2 | cortactin binding protein 2 |
| ENSMUSG00000060904 | 2.20 | 4.60 | 1.12E-23 | Arl1 | ADP-ribosylation factor-like 1 |
| ENSMUSG00000030470 | 2.20 | 4.60 | 2.19E-08 | Csrp3 | cysteine and glycine-rich protein 3 |
| ENSMUSG00000020364 | 2.20 | 4.60 | 2.11E-07 | Zfp354a | zinc finger protein 354A |
| ENSMUSG00000024671 | 2.20 | 4.59 | 1.66E-09 | NA | NA |
| ENSMUSG00000034520 | 2.20 | 4.59 | 5.40E-18 | Gjc1 | gap junction protein, gamma 1 |
| ENSMUSG00000028358 | 2.20 | 4.59 | 4.11E-20 | Zfp618 | zinc finger protein 618 |
| ENSMUSG00000087479 | 2.20 | 4.58 | 9.15E-03 | NA | NA |
| ENSMUSG00000027500 | 2.19 | 4.57 | 7.07E-10 | Stmn2 | stathmin-like 2 |
| ENSMUSG00000007039 | 2.19 | 4.57 | 1.30E-19 | Ddah2 | dimethylarginine dimethylaminohydrolase 2 |
| ENSMUSG00000033595 | 2.19 | 4.57 | 9.25E-05 | Lgi3 | leucine-rich repeat LGI family, member 3 |
| ENSMUSG00000038009 | 2.19 | 4.57 | 9.48E-03 | Dnajc22 | DnaJ (Hsp40) homolog, subfamily C, member 22 |
| ENSMUSG00000053475 | 2.19 | 4.55 | 3.30E-08 | Tnfaip6 | tumor necrosis factor alpha induced protein 6 |
| ENSMUSG00000045934 | 2.19 | 4.55 | 1.98E-17 | Mtmr11 | myotubularin related protein 11 |
| ENSMUSG00000044697 | 2.19 | 4.55 | 2.09E-03 | NA | NA |
| ENSMUSG00000058070 | 2.18 | 4.55 | 1.56E-17 | Eml1 | echinoderm microtubule associated protein like 1 |
| ENSMUSG00000032491 | 2.18 | 4.55 | 5.67E-15 | Nradd | neurotrophin receptor associated death domain |
| ENSMUSG00000022306 | 2.18 | 4.54 | 4.03E-08 | Zfpm2 | zinc finger protein, multitype 2 |
| ENSMUSG00000001630 | 2.18 | 4.54 | 1.57E-23 | Stk38l | serine/threonine kinase 38 like |
| ENSMUSG00000026021 | 2.18 | 4.54 | 7.95E-13 | Sumo1 | SMT3 suppressor of mif two 3 homolog 1 (yeast) |
| ENSMUSG00000001555 | 2.18 | 4.54 | 1.27E-15 | Fkbp10 | FK506 binding protein 10 |
| ENSMUSG00000045733 | 2.18 | 4.53 | 1.47E-04 | Sprn | shadow of prion protein |
| ENSMUSG00000079449 | 2.18 | 4.53 | 6.17E-03 | NA | NA |
| ENSMUSG00000028007 | 2.18 | 4.53 | 4.28E-16 | Snx7 | sorting nexin 7 |
| ENSMUSG00000043398 | 2.18 | 4.53 | 5.85E-04 | Gpr135 | G protein-coupled receptor 135 |
| ENSMUSG00000028108 | 2.18 | 4.53 | 7.91E-25 | Ecm1 | extracellular matrix protein 1 |
| ENSMUSG00000083246 | 2.18 | 4.53 | 1.62E-03 | NA | NA |
| ENSMUSG00000051486 | 2.18 | 4.53 | 1.01E-08 | Pcdhb11 | protocadherin beta 11 |
| ENSMUSG00000083307 | 2.18 | 4.53 | 8.49E-07 | NA | NA |
| ENSMUSG00000027520 | 2.18 | 4.53 | 1.46E-03 | Zdbf2 | zinc finger, DBF-type containing 2 |
| ENSMUSG00000050347 | 2.18 | 4.52 | 6.54E-07 | NA | NA |
| ENSMUSG00000032122 | 2.18 | 4.52 | 2.71E-26 | Slc37a2 | solute carrier family 37 (glycerol-3-phosphate transporter), member 2 |
| ENSMUSG00000075611 | 2.18 | 4.52 | 7.48E-04 | NA | NA |
| ENSMUSG00000056128 | 2.17 | 4.51 | 6.82E-03 | NA | NA |
| ENSMUSG00000062382 | 2.17 | 4.51 | 7.51E-25 | NA | NA |
| ENSMUSG00000052481 | 2.17 | 4.50 | 6.37E-03 | NA | NA |
| ENSMUSG00000022324 | 2.17 | 4.50 | 3.80E-15 | Matn2 | matrilin 2 |
| ENSMUSG00000062694 | 2.17 | 4.49 | 4.78E-12 | Cav3 | caveolin 3 |
| ENSMUSG00000027669 | 2.17 | 4.49 | 2.37E-34 | Gnb4 | guanine nucleotide binding protein (G protein), beta 4 |
| ENSMUSG00000031099 | 2.17 | 4.49 | 2.67E-08 | Smarca1 | SWI/SNF related, matrix associated, actin dependent regulator of chromatin, subfamily a, member 1 |
| ENSMUSG00000018459 | 2.17 | 4.49 | 2.61E-07 | Slc13a3 | solute carrier family 13 (sodium-dependent dicarboxylate transporter), member 3 |
| ENSMUSG00000022012 | 2.16 | 4.48 | 1.13E-09 | Enox1 | ecto-NOX disulfide-thiol exchanger 1 |
| ENSMUSG00000043850 | 2.16 | 4.47 | 2.45E-03 | Clrn1 | clarin 1 |
| ENSMUSG00000020142 | 2.16 | 4.47 | 7.08E-39 | Slc1a4 | solute carrier family 1 (glutamate/neutral amino acid transporter), member 4 |
| ENSMUSG00000089722 | 2.16 | 4.47 | 4.41E-04 | NA | NA |
| ENSMUSG00000041559 | 2.16 | 4.46 | 9.58E-07 | Fmod | fibromodulin |
| ENSMUSG00000012017 | 2.15 | 4.44 | 1.15E-10 | Scarf2 | scavenger receptor class F, member 2 |
| ENSMUSG00000000392 | 2.15 | 4.44 | 5.98E-20 | Fap | fibroblast activation protein |
| ENSMUSG00000021423 | 2.15 | 4.44 | 6.89E-23 | Ly86 | lymphocyte antigen 86 |
| ENSMUSG00000074690 | 2.15 | 4.43 | 1.24E-04 | NA | NA |
| ENSMUSG00000020811 | 2.15 | 4.43 | 9.57E-19 | Wscd1 | WSC domain containing 1 |
| ENSMUSG00000043943 | 2.15 | 4.43 | 8.06E-14 | Naalad2 | N-acetylated alpha-linked acidic dipeptidase 2 |
| ENSMUSG00000031340 | 2.14 | 4.42 | 2.38E-06 | Gabre | gamma-aminobutyric acid (GABA) A receptor, subunit epsilon |
| ENSMUSG00000052698 | 2.14 | 4.41 | 7.14E-17 | Tln2 | talin 2 |
| ENSMUSG00000001473 | 2.14 | 4.41 | 7.06E-18 | Tubb6 | tubulin, beta 6 class V |
| ENSMUSG00000003062 | 2.14 | 4.41 | 6.31E-25 | Stard3nl | STARD3 N-terminal like |
| ENSMUSG00000044043 | 2.14 | 4.41 | 1.08E-07 | Pcdhb14 | protocadherin beta 14 |
| ENSMUSG00000089886 | 2.14 | 4.41 | 2.80E-03 | NA | NA |
| ENSMUSG00000082284 | 2.14 | 4.40 | 3.75E-05 | NA | NA |
| ENSMUSG00000032314 | 2.14 | 4.40 | 9.71E-23 | Etfa | electron transferring flavoprotein, alpha polypeptide |
| ENSMUSG00000036139 | 2.14 | 4.40 | 3.38E-06 | Hoxc9 | homeobox C9 |
| ENSMUSG00000075400 | 2.14 | 4.40 | 8.69E-09 | NA | NA |
| ENSMUSG00000039005 | 2.14 | 4.40 | 4.17E-11 | Tlr4 | toll-like receptor 4 |
| ENSMUSG00000036242 | 2.14 | 4.39 | 1.74E-06 | 3632451O06Rik | RIKEN cDNA 3632451O06 gene |
| ENSMUSG00000037994 | 2.14 | 4.39 | 8.49E-12 | Slc9b2 | solute carrier family 9, subfamily B (NHA2, cation proton antiporter 2), member 2 |
| ENSMUSG00000020658 | 2.13 | 4.39 | 9.54E-10 | Efr3b | EFR3 homolog B (S. cerevisiae) |
| ENSMUSG00000056832 | 2.13 | 4.39 | 1.56E-16 | Ttc26 | tetratricopeptide repeat domain 26 |
| ENSMUSG00000062127 | 2.13 | 4.39 | 5.65E-28 | Cttnbp2nl | CTTNBP2 N-terminal like |
| ENSMUSG00000070044 | 2.13 | 4.38 | 1.34E-23 | Fam149a | family with sequence similarity 149, member A |
| ENSMUSG00000054594 | 2.13 | 4.38 | 4.64E-08 | Oscar | osteoclast associated receptor |
| ENSMUSG00000048720 | 2.13 | 4.38 | 5.16E-20 | Tbc1d12 | TBC1D12: TBC1 domain family, member 12 |
| ENSMUSG00000078570 | 2.13 | 4.38 | 5.91E-09 | 1110065P20Rik | RIKEN cDNA 1110065P20 gene |
| ENSMUSG00000026725 | 2.13 | 4.38 | 2.10E-19 | Tnn | tenascin N |
| ENSMUSG00000015947 | 2.13 | 4.38 | 3.64E-26 | Fcgr1 | Fc receptor, IgG, high affinity I |
| ENSMUSG00000020399 | 2.13 | 4.37 | 2.16E-22 | Havcr2 | hepatitis A virus cellular receptor 2 |
| ENSMUSG00000032584 | 2.13 | 4.37 | 1.52E-24 | Mst1r | macrophage stimulating 1 receptor (c-met-related tyrosine kinase) |
| ENSMUSG00000029304 | 2.13 | 4.37 | 1.85E-13 | Spp1 | secreted phosphoprotein 1 |
| ENSMUSG00000029352 | 2.13 | 4.37 | 1.36E-09 | Crybb3 | crystallin, beta B3 |
| ENSMUSG00000029436 | 2.13 | 4.37 | 2.66E-21 | Mmp17 | matrix metallopeptidase 17 |
| ENSMUSG00000033576 | 2.13 | 4.37 | 1.80E-24 | Apol6 | apolipoprotein L 6 |
| ENSMUSG00000037406 | 2.12 | 4.36 | 2.79E-03 | Htra4 | HtrA serine peptidase 4 |
| ENSMUSG00000026604 | 2.12 | 4.36 | 1.16E-16 | Ptpn14 | protein tyrosine phosphatase, non-receptor type 14 |
| ENSMUSG00000024501 | 2.12 | 4.36 | 9.78E-22 | Dpysl3 | dihydropyrimidinase-like 3 |
| ENSMUSG00000027202 | 2.12 | 4.36 | 4.74E-04 | Slc12a1 | solute carrier family 12, member 1 |
| ENSMUSG00000033355 | 2.12 | 4.35 | 3.17E-13 | Rtp4 | receptor transporter protein 4 |
| ENSMUSG00000070720 | 2.12 | 4.35 | 1.08E-05 | Tmem200b | transmembrane protein 200B |
| ENSMUSG00000056258 | 2.12 | 4.34 | 2.75E-03 | Kcnq3 | potassium voltage-gated channel, subfamily Q, member 3 |
| ENSMUSG00000039103 | 2.12 | 4.34 | 2.12E-07 | Nexn | nexilin |
| ENSMUSG00000087212 | 2.12 | 4.34 | 1.16E-04 | NA | NA |
| ENSMUSG00000024798 | 2.12 | 4.34 | 1.06E-04 | Htr7 | 5-hydroxytryptamine (serotonin) receptor 7 |
| ENSMUSG00000079457 | 2.12 | 4.34 | 7.64E-03 | NA | NA |
| ENSMUSG00000081629 | 2.12 | 4.34 | 1.50E-08 | NA | NA |
| ENSMUSG00000046186 | 2.12 | 4.33 | 2.18E-14 | Cd109 | CD109 antigen |
| ENSMUSG00000046727 | 2.11 | 4.33 | 5.28E-08 | Cystm1 | cysteine-rich transmembrane module containing 1 |
| ENSMUSG00000031555 | 2.11 | 4.33 | 4.41E-27 | Adam9 | a disintegrin and metallopeptidase domain 9 (meltrin gamma) |
| ENSMUSG00000032383 | 2.11 | 4.33 | 7.51E-25 | Ppib | peptidylprolyl isomerase B |
| ENSMUSG00000069806 | 2.11 | 4.33 | 7.87E-14 | NA | NA |
| ENSMUSG00000084111 | 2.11 | 4.33 | 6.70E-03 | NA | NA |
| ENSMUSG00000069184 | 2.11 | 4.33 | 1.01E-05 | Zfp72 | zinc finger protein 72 |
| ENSMUSG00000084127 | 2.11 | 4.32 | 3.77E-04 | NA | NA |
| ENSMUSG00000022485 | 2.11 | 4.32 | 1.29E-05 | Hoxc5 | homeobox C5 |
| ENSMUSG00000040280 | 2.11 | 4.32 | 4.18E-09 | Ndufa4l2 | NADH dehydrogenase (ubiquinone) 1 alpha subcomplex, 4-like 2 |
| ENSMUSG00000086866 | 2.11 | 4.32 | 9.88E-04 | NA | NA |
| ENSMUSG00000015750 | 2.11 | 4.32 | 6.19E-28 | Aph1a | anterior pharynx defective 1a homolog (C. elegans) |
| ENSMUSG00000027684 | 2.11 | 4.32 | 2.22E-13 | Mecom | MDS1 and EVI1 complex locus |
| ENSMUSG00000000317 | 2.11 | 4.32 | 2.57E-19 | Bcl6b | B cell CLL/lymphoma 6, member B |
| ENSMUSG00000081228 | 2.11 | 4.32 | 5.37E-08 | NA | NA |
| ENSMUSG00000089929 | 2.11 | 4.32 | 4.24E-04 | Bcl2a1b | B cell leukemia/lymphoma 2 related protein A1b |
| ENSMUSG00000033420 | 2.11 | 4.31 | 9.03E-15 | Antxr1 | anthrax toxin receptor 1 |
| ENSMUSG00000032757 | 2.11 | 4.31 | 2.02E-21 | Bet1 | blocked early in transport 1 homolog (S. cerevisiae) |
| ENSMUSG00000006576 | 2.11 | 4.31 | 2.92E-15 | Slc4a3 | solute carrier family 4 (anion exchanger), member 3 |
| ENSMUSG00000004791 | 2.11 | 4.30 | 6.04E-14 | Pgf | placental growth factor |
| ENSMUSG00000073849 | 2.10 | 4.30 | 4.82E-08 | NA | NA |
| ENSMUSG00000059447 | 2.10 | 4.30 | 6.64E-14 | Hadhb | hydroxyacyl-Coenzyme A dehydrogenase/3-ketoacyl-Coenzyme A thiolase/enoyl-Coenzyme A hydratase (trifunctional protein), beta subunit |
| ENSMUSG00000045036 | 2.10 | 4.29 | 3.64E-04 | Tmem232 | transmembrane protein 232 |
| ENSMUSG00000031246 | 2.10 | 4.29 | 1.34E-29 | Sh3bgrl | SH3-binding domain glutamic acid-rich protein like |
| ENSMUSG00000086231 | 2.10 | 4.29 | 1.53E-03 | NA | NA |
| ENSMUSG00000022094 | 2.10 | 4.29 | 3.74E-19 | Slc39a14 | solute carrier family 39 (zinc transporter), member 14 |
| ENSMUSG00000020092 | 2.10 | 4.28 | 2.55E-24 | Pald1 | phosphatase domain containing, paladin 1 |
| ENSMUSG00000067916 | 2.10 | 4.28 | 1.07E-06 | Gm13139 | predicted gene 13139 |
| ENSMUSG00000017734 | 2.10 | 4.28 | 2.89E-23 | Dbndd2 | dysbindin (dystrobrevin binding protein 1) domain containing 2 |
| ENSMUSG00000050663 | 2.10 | 4.28 | 3.33E-03 | Trhde | TRH-degrading enzyme |
| ENSMUSG00000087365 | 2.10 | 4.28 | 3.19E-05 | NA | NA |
| ENSMUSG00000030671 | 2.10 | 4.28 | 7.16E-15 | Pde3b | phosphodiesterase 3B, cGMP-inhibited |
| ENSMUSG00000074677 | 2.10 | 4.27 | 2.98E-12 | LOC100038947 | signal-regulatory protein beta 1-like |
| ENSMUSG00000019230 | 2.09 | 4.27 | 4.50E-05 | Lhx9 | LIM homeobox protein 9 |
| ENSMUSG00000082002 | 2.09 | 4.27 | 9.74E-04 | NA | NA |
| ENSMUSG00000050022 | 2.09 | 4.27 | 1.20E-13 | Amz1 | archaelysin family metallopeptidase 1 |
| ENSMUSG00000033900 | 2.09 | 4.26 | 1.58E-10 | Map9 | microtubule-associated protein 9 |
| ENSMUSG00000026251 | 2.09 | 4.26 | 4.54E-04 | Chrnd | cholinergic receptor, nicotinic, delta polypeptide |
| ENSMUSG00000041378 | 2.09 | 4.26 | 3.85E-08 | Cldn5 | claudin 5 |
| ENSMUSG00000026335 | 2.09 | 4.26 | 1.73E-27 | Pam | peptidylglycine alpha-amidating monooxygenase |
| ENSMUSG00000018906 | 2.09 | 4.26 | 3.37E-16 | P4ha2 | procollagen-proline, 2-oxoglutarate 4-dioxygenase (proline 4-hydroxylase), alpha II polypeptide |
| ENSMUSG00000020427 | 2.09 | 4.26 | 6.86E-13 | Igfbp3 | insulin-like growth factor binding protein 3 |
| ENSMUSG00000032291 | 2.09 | 4.26 | 1.43E-04 | Crabp1 | cellular retinoic acid binding protein I |
| ENSMUSG00000022941 | 2.09 | 4.26 | 3.07E-06 | Ripply3 | ripply3 homolog (zebrafish) |
| ENSMUSG00000036564 | 2.09 | 4.25 | 3.56E-12 | Ndrg4 | N-myc downstream regulated gene 4 |
| ENSMUSG00000065280 | 2.09 | 4.25 | 1.12E-06 | NA | NA |
| ENSMUSG00000034560 | 2.09 | 4.25 | 5.52E-22 | A230046K03Rik | RIKEN cDNA A230046K03 gene |
| ENSMUSG00000002059 | 2.09 | 4.25 | 8.39E-23 | Rab34 | RAB34, member of RAS oncogene family |
| ENSMUSG00000078532 | 2.09 | 4.24 | 3.78E-19 | Nkain1 | Na+/K+ transporting ATPase interacting 1 |
| ENSMUSG00000052825 | 2.08 | 4.24 | 4.39E-09 | NA | NA |
| ENSMUSG00000042828 | 2.08 | 4.24 | 7.65E-12 | Trim72 | tripartite motif-containing 72 |
| ENSMUSG00000061086 | 2.08 | 4.24 | 6.47E-06 | Myl4 | myosin, light polypeptide 4 |
| ENSMUSG00000054203 | 2.08 | 4.23 | 2.09E-14 | Ifi205 | interferon activated gene 205 |
| ENSMUSG00000082292 | 2.08 | 4.23 | 9.64E-29 | NA | NA |
| ENSMUSG00000074457 | 2.08 | 4.23 | 4.46E-27 | S100a16 | S100 calcium binding protein A16 |
| ENSMUSG00000048138 | 2.08 | 4.23 | 2.51E-03 | Dmrt2 | doublesex and mab-3 related transcription factor 2 |
| ENSMUSG00000025551 | 2.08 | 4.23 | 1.39E-04 | Fgf14 | fibroblast growth factor 14 |
| ENSMUSG00000021458 | 2.08 | 4.23 | 8.32E-32 | 2010111I01Rik | RIKEN cDNA 2010111I01 gene |
| ENSMUSG00000001300 | 2.08 | 4.22 | 2.20E-14 | Efnb2 | ephrin B2 |
| ENSMUSG00000085954 | 2.08 | 4.22 | 9.16E-03 | NA | NA |
| ENSMUSG00000041012 | 2.08 | 4.22 | 1.31E-08 | Cmtm8 | CKLF-like MARVEL transmembrane domain containing 8 |
| ENSMUSG00000028978 | 2.08 | 4.22 | 6.12E-08 | Nos3 | nitric oxide synthase 3, endothelial cell |
| ENSMUSG00000007872 | 2.08 | 4.22 | 1.16E-13 | Id3 | inhibitor of DNA binding 3 |
| ENSMUSG00000087635 | 2.08 | 4.21 | 1.13E-06 | NA | NA |
| ENSMUSG00000030895 | 2.08 | 4.21 | 7.36E-05 | Hpx | hemopexin |
| ENSMUSG00000022817 | 2.08 | 4.21 | 7.95E-28 | Itgb5 | integrin beta 5 |
| ENSMUSG00000042138 | 2.07 | 4.21 | 1.00E-17 | Msantd2 | Myb/SANT-like DNA-binding domain containing 2 |
| ENSMUSG00000070348 | 2.07 | 4.21 | 4.35E-16 | Ccnd1 | cyclin D1 |
| ENSMUSG00000046589 | 2.07 | 4.21 | 5.57E-05 | Lrrc8e | leucine rich repeat containing 8 family, member E |
| ENSMUSG00000025348 | 2.07 | 4.21 | 2.07E-14 | Itga7 | integrin alpha 7 |
| ENSMUSG00000040511 | 2.07 | 4.21 | 5.65E-42 | Pvr | poliovirus receptor |
| ENSMUSG00000001507 | 2.07 | 4.20 | 1.91E-18 | Itga3 | integrin alpha 3 |
| ENSMUSG00000029718 | 2.07 | 4.19 | 4.46E-17 | Pcolce | procollagen C-endopeptidase enhancer protein |
| ENSMUSG00000052155 | 2.07 | 4.19 | 1.07E-21 | Acvr2a | activin receptor IIA |
| ENSMUSG00000042254 | 2.07 | 4.19 | 3.60E-03 | Cilp | cartilage intermediate layer protein, nucleotide pyrophosphohydrolase |
| ENSMUSG00000070709 | 2.07 | 4.19 | 1.01E-16 | 1700049G17Rik | RIKEN cDNA 1700049G17 gene |
| ENSMUSG00000029576 | 2.07 | 4.19 | 2.06E-05 | Radil | Ras association and DIL domains |
| ENSMUSG00000022408 | 2.07 | 4.19 | 8.04E-12 | Fam83f | family with sequence similarity 83, member F |
| ENSMUSG00000024481 | 2.07 | 4.19 | 1.54E-03 | 4833403I15Rik | RIKEN cDNA 4833403I15 gene |
| ENSMUSG00000031351 | 2.07 | 4.19 | 1.89E-05 | Zfp185 | zinc finger protein 185 |
| ENSMUSG00000052392 | 2.06 | 4.18 | 8.87E-04 | Acot4 | acyl-CoA thioesterase 4 |
| ENSMUSG00000037060 | 2.06 | 4.18 | 2.16E-14 | Prkcdbp | protein kinase C, delta binding protein |
| ENSMUSG00000085596 | 2.06 | 4.18 | 9.75E-07 | NA | NA |
| ENSMUSG00000074700 | 2.06 | 4.18 | 4.42E-03 | NA | NA |
| ENSMUSG00000024614 | 2.06 | 4.18 | 7.56E-19 | Tmx3 | thioredoxin-related transmembrane protein 3 |
| ENSMUSG00000084882 | 2.06 | 4.17 | 4.57E-03 | NA | NA |
| ENSMUSG00000084314 | 2.06 | 4.17 | 2.64E-05 | NA | NA |
| ENSMUSG00000039735 | 2.06 | 4.17 | 6.53E-17 | Fnbp1l | formin binding protein 1-like |
| ENSMUSG00000052450 | 2.06 | 4.17 | 4.04E-05 | NA | NA |
| ENSMUSG00000027238 | 2.06 | 4.17 | 4.87E-07 | Frmd5 | FERM domain containing 5 |
| ENSMUSG00000086281 | 2.06 | 4.17 | 4.37E-20 | NA | NA |
| ENSMUSG00000079547 | 2.06 | 4.17 | 1.35E-27 | H2-DMb1 | histocompatibility 2, class II, locus Mb1 |
| ENSMUSG00000044499 | 2.06 | 4.17 | 2.16E-04 | Hs3st5 | heparan sulfate (glucosamine) 3-O-sulfotransferase 5 |
| ENSMUSG00000073635 | 2.06 | 4.16 | 2.89E-10 | NA | NA |
| ENSMUSG00000030499 | 2.06 | 4.16 | 4.61E-13 | Kctd15 | potassium channel tetramerisation domain containing 15 |
| ENSMUSG00000031904 | 2.06 | 4.16 | 5.04E-38 | Slc7a6 | solute carrier family 7 (cationic amino acid transporter, y+ system), member 6 |
| ENSMUSG00000046491 | 2.06 | 4.16 | 6.55E-08 | C1qtnf2 | C1q and tumor necrosis factor related protein 2 |
| ENSMUSG00000022330 | 2.05 | 4.15 | 3.92E-04 | Osr2 | odd-skipped related 2 |
| ENSMUSG00000048285 | 2.05 | 4.15 | 6.05E-26 | Frmd6 | FERM domain containing 6 |
| ENSMUSG00000004771 | 2.05 | 4.15 | 2.31E-14 | Rab11a | RAB11a, member RAS oncogene family |
| ENSMUSG00000085521 | 2.05 | 4.15 | 7.06E-16 | NA | NA |
| ENSMUSG00000032725 | 2.05 | 4.15 | 1.74E-10 | Folr2 | folate receptor 2 (fetal) |
| ENSMUSG00000022883 | 2.05 | 4.15 | 4.61E-07 | Robo1 | roundabout homolog 1 (Drosophila) |
| ENSMUSG00000022098 | 2.05 | 4.14 | 5.03E-19 | Bmp1 | bone morphogenetic protein 1 |
| ENSMUSG00000083679 | 2.05 | 4.14 | 2.52E-09 | NA | NA |
| ENSMUSG00000033898 | 2.05 | 4.14 | 6.30E-03 | Cfhr2 | complement factor H-related 2 |
| ENSMUSG00000090112 | 2.05 | 4.14 | 1.36E-14 | Shprh | SNF2 histone linker PHD RING helicase |
| ENSMUSG00000045275 | 2.05 | 4.14 | 1.56E-07 | Lca5l | Leber congenital amaurosis 5-like |
| ENSMUSG00000040026 | 2.05 | 4.14 | 4.10E-04 | Saa3 | serum amyloid A 3 |
| ENSMUSG00000078566 | 2.05 | 4.14 | 2.51E-13 | Bnip3 | BCL2/adenovirus E1B interacting protein 3 |
| ENSMUSG00000080364 | 2.05 | 4.13 | 3.28E-03 | NA | NA |
| ENSMUSG00000027797 | 2.05 | 4.13 | 1.57E-09 | Dclk1 | doublecortin-like kinase 1 |
| ENSMUSG00000051367 | 2.04 | 4.13 | 3.57E-14 | Six1 | sine oculis-related homeobox 1 |
| ENSMUSG00000085604 | 2.04 | 4.12 | 2.89E-03 | NA | NA |
| ENSMUSG00000078606 | 2.04 | 4.12 | 3.86E-11 | Gm4070 | predicted gene 4070 |
| ENSMUSG00000021876 | 2.04 | 4.12 | 1.48E-21 | Rnase4 | ribonuclease, RNase A family 4 |
| ENSMUSG00000047033 | 2.04 | 4.12 | 6.48E-05 | Pcdhb15 | protocadherin beta 15 |
| ENSMUSG00000037855 | 2.04 | 4.11 | 3.41E-10 | Zfp365 | zinc finger protein 365 |
| ENSMUSG00000044042 | 2.04 | 4.10 | 5.74E-17 | Fmn1 | formin 1 |
| ENSMUSG00000082223 | 2.04 | 4.10 | 9.45E-07 | NA | NA |
| ENSMUSG00000087576 | 2.03 | 4.10 | 4.22E-04 | NA | NA |
| ENSMUSG00000036298 | 2.03 | 4.10 | 6.82E-09 | Slc2a13 | solute carrier family 2 (facilitated glucose transporter), member 13 |
| ENSMUSG00000046550 | 2.03 | 4.08 | 1.32E-04 | Spin2c | spindlin family, member 2C |
| ENSMUSG00000027377 | 2.03 | 4.08 | 2.45E-07 | Mall | mal, T cell differentiation protein-like |
| ENSMUSG00000021268 | 2.03 | 4.08 | 2.08E-08 | Meg3 | maternally expressed 3 |
| ENSMUSG00000078836 | 2.03 | 4.07 | 2.73E-03 | NA | NA |
| ENSMUSG00000035045 | 2.02 | 4.07 | 2.77E-07 | Zc3h12b | zinc finger CCCH-type containing 12B |
| ENSMUSG00000083083 | 2.02 | 4.07 | 9.22E-04 | NA | NA |
| ENSMUSG00000022340 | 2.02 | 4.06 | 2.70E-09 | Sybu | syntabulin (syntaxin-interacting) |
| ENSMUSG00000045672 | 2.02 | 4.06 | 1.65E-06 | Col27a1 | collagen, type XXVII, alpha 1 |
| ENSMUSG00000044647 | 2.02 | 4.06 | 1.12E-07 | Csrnp3 | cysteine-serine-rich nuclear protein 3 |
| ENSMUSG00000046387 | 2.02 | 4.05 | 2.22E-11 | Pcdhb17 | protocadherin beta 17 |
| ENSMUSG00000029061 | 2.02 | 4.05 | 4.13E-14 | Mmp23 | matrix metallopeptidase 23 |
| ENSMUSG00000039831 | 2.02 | 4.05 | 3.80E-29 | Arhgap29 | Rho GTPase activating protein 29 |
| ENSMUSG00000026121 | 2.02 | 4.05 | 4.38E-15 | Sema4c | sema domain, immunoglobulin domain (Ig), transmembrane domain (TM) and short cytoplasmic domain, (semaphorin) 4C |
| ENSMUSG00000026315 | 2.01 | 4.04 | 1.95E-23 | Serpinb8 | serine (or cysteine) peptidase inhibitor, clade B, member 8 |
| ENSMUSG00000043099 | 2.01 | 4.04 | 2.08E-10 | Hic1 | hypermethylated in cancer 1 |
| ENSMUSG00000065829 | 2.01 | 4.03 | 8.05E-03 | NA | NA |
| ENSMUSG00000020256 | 2.01 | 4.03 | 5.55E-14 | Aldh1l2 | aldehyde dehydrogenase 1 family, member L2 |
| ENSMUSG00000074513 | 2.01 | 4.03 | 6.95E-16 | Arfip1 | ADP-ribosylation factor interacting protein 1 |
| ENSMUSG00000043329 | 2.01 | 4.03 | 8.03E-04 | NA | NA |
| ENSMUSG00000034783 | 2.01 | 4.02 | 7.13E-05 | Cd207 | CD207 antigen |
| ENSMUSG00000041479 | 2.01 | 4.02 | 1.15E-09 | Syt15 | synaptotagmin XV |
| ENSMUSG00000038037 | 2.01 | 4.02 | 1.07E-05 | Socs1 | suppressor of cytokine signaling 1 |
| ENSMUSG00000061603 | 2.01 | 4.02 | 4.00E-09 | Akap6 | A kinase (PRKA) anchor protein 6 |
| ENSMUSG00000028040 | 2.01 | 4.02 | 2.58E-06 | Efna4 | ephrin A4 |
| ENSMUSG00000045201 | 2.01 | 4.01 | 5.09E-03 | Lrrc3b | leucine rich repeat containing 3B |
| ENSMUSG00000081406 | 2.01 | 4.01 | 4.51E-14 | NA | NA |
| ENSMUSG00000029671 | 2.01 | 4.01 | 2.39E-15 | Wnt16 | wingless-related MMTV integration site 16 |
| ENSMUSG00000054626 | 2.01 | 4.01 | 7.13E-04 | Xlr | X-linked lymphocyte-regulated |
| ENSMUSG00000063108 | 2.00 | 4.01 | 1.07E-19 | Zfp26 | zinc finger protein 26 |
| ENSMUSG00000052727 | 2.00 | 4.01 | 4.16E-07 | Map1b | microtubule-associated protein 1B |
| ENSMUSG00000042878 | 2.00 | 4.01 | 2.77E-06 | NA | NA |
| ENSMUSG00000047759 | 2.00 | 4.01 | 1.37E-03 | Hs3st3a1 | heparan sulfate (glucosamine) 3-O-sulfotransferase 3A1 |
| ENSMUSG00000085461 | 2.00 | 4.01 | 2.29E-03 | NA | NA |
| ENSMUSG00000051554 | 2.00 | 4.01 | 5.40E-03 | NA | NA |
| ENSMUSG00000079252 | 2.00 | 4.01 | 1.75E-33 | NA | NA |
| ENSMUSG00000060143 | 2.00 | 4.01 | 5.46E-04 | NA | NA |
| ENSMUSG00000041845 | 2.00 | 4.00 | 1.05E-07 | Rhod | ras homolog gene family, member D |
| ENSMUSG00000053317 | 2.00 | 4.00 | 5.25E-08 | Sec61b | Sec61 beta subunit |
| ENSMUSG00000022754 | 2.00 | 4.00 | 5.25E-18 | Tmem45a | transmembrane protein 45a |
| ENSMUSG00000024483 | 2.00 | 4.00 | 7.37E-10 | Ankhd1 | ankyrin repeat and KH domain containing 1 |
| ENSMUSG00000032334 | 2.00 | 4.00 | 4.61E-12 | Loxl1 | lysyl oxidase-like 1 |
| ENSMUSG00000046667 | 2.00 | 4.00 | 5.39E-06 | Rbm12b1 | RNA binding motif protein 12 B1 |
| ENSMUSG00000081726 | 2.00 | 4.00 | 9.31E-04 | NA | NA |
| ENSMUSG00000022665 | 2.00 | 4.00 | 8.24E-09 | Ccdc80 | coiled-coil domain containing 80 |
| ENSMUSG00000083964 | 2.00 | 3.99 | 3.28E-03 | NA | NA |
| ENSMUSG00000029544 | 2.00 | 3.99 | 2.02E-11 | Cabp1 | calcium binding protein 1 |
| ENSMUSG00000038264 | 2.00 | 3.99 | 6.55E-17 | Sema7a | sema domain, immunoglobulin domain (Ig), and GPI membrane anchor, (semaphorin) 7A |
| ENSMUSG00000089852 | 2.00 | 3.99 | 2.43E-04 | NA | NA |
| ENSMUSG00000047348 | 1.99 | 3.98 | 9.75E-05 | NA | NA |
| ENSMUSG00000073851 | 1.99 | 3.98 | 2.20E-05 | NA | NA |
| ENSMUSG00000019874 | 1.99 | 3.98 | 8.22E-05 | Fabp7 | fatty acid binding protein 7, brain |
| ENSMUSG00000049233 | 1.99 | 3.98 | 3.39E-15 | NA | NA |
| ENSMUSG00000024059 | 1.99 | 3.98 | 4.39E-08 | Clip4 | CAP-GLY domain containing linker protein family, member 4 |
| ENSMUSG00000025478 | 1.99 | 3.98 | 2.64E-10 | Dpysl4 | dihydropyrimidinase-like 4 |
| ENSMUSG00000083261 | 1.99 | 3.98 | 6.78E-11 | NA | NA |
| ENSMUSG00000086860 | 1.99 | 3.97 | 3.36E-03 | Gm1720 | predicted gene 1720 |
| ENSMUSG00000020814 | 1.99 | 3.97 | 3.13E-18 | Mxra7 | matrix-remodelling associated 7 |
| ENSMUSG00000026726 | 1.99 | 3.97 | 6.37E-08 | Cubn | cubilin (intrinsic factor-cobalamin receptor) |
| ENSMUSG00000038210 | 1.99 | 3.97 | 2.38E-05 | Hoxa11 | homeobox A11 |
| ENSMUSG00000041660 | 1.99 | 3.97 | 1.74E-03 | Bbox1 | butyrobetaine (gamma), 2-oxoglutarate dioxygenase 1 (gamma-butyrobetaine hydroxylase) |
| ENSMUSG00000071042 | 1.99 | 3.97 | 8.48E-14 | Rasgrp3 | RAS, guanyl releasing protein 3 |
| ENSMUSG00000085282 | 1.99 | 3.96 | 2.62E-08 | Gm15663 | predicted gene 15663 |
| ENSMUSG00000052726 | 1.99 | 3.96 | 8.59E-09 | Kcnt2 | potassium channel, subfamily T, member 2 |
| ENSMUSG00000082588 | 1.98 | 3.96 | 1.74E-06 | NA | NA |
| ENSMUSG00000042182 | 1.98 | 3.95 | 7.72E-08 | Bend6 | BEN domain containing 6 |
| ENSMUSG00000028986 | 1.98 | 3.95 | 3.77E-18 | Klhl7 | kelch-like 7 |
| ENSMUSG00000085894 | 1.98 | 3.95 | 6.61E-08 | NA | NA |
| ENSMUSG00000046580 | 1.98 | 3.95 | 4.02E-07 | NA | NA |
| ENSMUSG00000025165 | 1.98 | 3.95 | 1.04E-03 | Sectm1a | secreted and transmembrane 1A |
| ENSMUSG00000034173 | 1.98 | 3.94 | 5.38E-07 | 2410018M08Rik | RIKEN cDNA 2410018M08 gene |
| ENSMUSG00000033542 | 1.98 | 3.94 | 2.28E-14 | Arhgef5 | Rho guanine nucleotide exchange factor (GEF) 5 |
| ENSMUSG00000064419 | 1.98 | 3.94 | 8.05E-03 | NA | NA |
| ENSMUSG00000075229 | 1.98 | 3.94 | 4.39E-08 | Ccdc58 | coiled-coil domain containing 58 |
| ENSMUSG00000034684 | 1.98 | 3.94 | 7.92E-18 | Sema3f | sema domain, immunoglobulin domain (Ig), short basic domain, secreted, (semaphorin) 3F |
| ENSMUSG00000023349 | 1.98 | 3.94 | 8.84E-10 | Clec4n | C-type lectin domain family 4, member n |
| ENSMUSG00000022371 | 1.98 | 3.93 | 5.32E-11 | Col14a1 | collagen, type XIV, alpha 1 |
| ENSMUSG00000079507 | 1.97 | 3.93 | 2.99E-09 | H2-Q1 | histocompatibility 2, Q region locus 1 |
| ENSMUSG00000061100 | 1.97 | 3.93 | 1.48E-03 | Retnla | resistin like alpha |
| ENSMUSG00000037613 | 1.97 | 3.93 | 4.65E-26 | Tnfrsf23 | tumor necrosis factor receptor superfamily, member 23 |
| ENSMUSG00000041992 | 1.97 | 3.93 | 1.91E-06 | Rapgef5 | Rap guanine nucleotide exchange factor (GEF) 5 |
| ENSMUSG00000035735 | 1.97 | 3.92 | 1.47E-08 | Dagla | diacylglycerol lipase, alpha |
| ENSMUSG00000036934 | 1.97 | 3.92 | 2.91E-11 | 4921524J17Rik | RIKEN cDNA 4921524J17 gene |
| ENSMUSG00000035692 | 1.97 | 3.92 | 1.84E-12 | Isg15 | ISG15 ubiquitin-like modifier |
| ENSMUSG00000086228 | 1.97 | 3.92 | 6.33E-03 | Gm514 | predicted gene 514 |
| ENSMUSG00000045842 | 1.97 | 3.92 | 2.15E-03 | NA | NA |
| ENSMUSG00000027900 | 1.97 | 3.91 | 3.13E-29 | Dram2 | DNA-damage regulated autophagy modulator 2 |
| ENSMUSG00000006931 | 1.97 | 3.91 | 5.33E-13 | Leprel4 | leprecan-like 4 |
| ENSMUSG00000082120 | 1.97 | 3.91 | 1.83E-07 | NA | NA |
| ENSMUSG00000005958 | 1.97 | 3.91 | 2.01E-11 | Ephb3 | Eph receptor B3 |
| ENSMUSG00000022044 | 1.97 | 3.91 | 5.94E-03 | Stmn4 | stathmin-like 4 |
| ENSMUSG00000030598 | 1.97 | 3.90 | 4.59E-14 | Fbxo17 | F-box protein 17 |
| ENSMUSG00000055407 | 1.96 | 3.90 | 7.56E-07 | Map6 | microtubule-associated protein 6 |
| ENSMUSG00000070605 | 1.96 | 3.90 | 2.35E-05 | Gm13251 | predicted gene 13251 |
| ENSMUSG00000043664 | 1.96 | 3.90 | 1.44E-04 | Tmem221 | transmembrane protein 221 |
| ENSMUSG00000058420 | 1.96 | 3.90 | 6.29E-03 | Syt17 | synaptotagmin XVII |
| ENSMUSG00000019467 | 1.96 | 3.90 | 1.79E-17 | Arhgef25 | Rho guanine nucleotide exchange factor (GEF) 25 |
| ENSMUSG00000020027 | 1.96 | 3.89 | 8.19E-17 | Socs2 | suppressor of cytokine signaling 2 |
| ENSMUSG00000043295 | 1.96 | 3.89 | 1.14E-05 | NA | NA |
| ENSMUSG00000036206 | 1.96 | 3.89 | 2.32E-19 | Sh3bp4 | SH3-domain binding protein 4 |
| ENSMUSG00000028965 | 1.96 | 3.89 | 5.24E-07 | Tnfrsf9 | tumor necrosis factor receptor superfamily, member 9 |
| ENSMUSG00000059588 | 1.96 | 3.89 | 8.18E-10 | Calcrl | calcitonin receptor-like |
| ENSMUSG00000025875 | 1.96 | 3.89 | 2.34E-16 | Tspan17 | tetraspanin 17 |
| ENSMUSG00000027224 | 1.96 | 3.89 | 1.19E-04 | Duoxa1 | dual oxidase maturation factor 1 |
| ENSMUSG00000031327 | 1.96 | 3.89 | 4.23E-07 | Chic1 | cysteine-rich hydrophobic domain 1 |
| ENSMUSG00000047414 | 1.96 | 3.88 | 2.50E-23 | Flrt2 | fibronectin leucine rich transmembrane protein 2 |
| ENSMUSG00000022053 | 1.96 | 3.88 | 3.18E-07 | Ebf2 | early B cell factor 2 |
| ENSMUSG00000066667 | 1.96 | 3.88 | 6.06E-03 | NA | NA |
| ENSMUSG00000012889 | 1.95 | 3.87 | 4.24E-16 | Podnl1 | podocan-like 1 |
| ENSMUSG00000019899 | 1.95 | 3.87 | 1.70E-13 | Lama2 | laminin, alpha 2 |
| ENSMUSG00000042115 | 1.95 | 3.87 | 4.69E-03 | Klhdc8a | kelch domain containing 8A |
| ENSMUSG00000044534 | 1.95 | 3.87 | 1.34E-10 | Ackr2 | atypical chemokine receptor 2 |
| ENSMUSG00000035585 | 1.95 | 3.87 | 1.13E-17 | Tsen34 | tRNA splicing endonuclease 34 homolog (S. cerevisiae) |
| ENSMUSG00000087229 | 1.95 | 3.85 | 5.71E-05 | NA | NA |
| ENSMUSG00000020388 | 1.95 | 3.85 | 2.03E-15 | Pdlim4 | PDZ and LIM domain 4 |
| ENSMUSG00000033585 | 1.95 | 3.85 | 1.51E-08 | Ndn | necdin |
| ENSMUSG00000073176 | 1.95 | 3.85 | 6.88E-11 | Zfp449 | zinc finger protein 449 |
| ENSMUSG00000048251 | 1.95 | 3.85 | 9.66E-05 | Bcl11b | B cell leukemia/lymphoma 11B |
| ENSMUSG00000022505 | 1.94 | 3.85 | 4.66E-28 | Emp2 | epithelial membrane protein 2 |
| ENSMUSG00000047227 | 1.94 | 3.85 | 3.29E-04 | Gm527 | predicted gene 527 |
| ENSMUSG00000028339 | 1.94 | 3.85 | 7.11E-08 | Col15a1 | collagen, type XV, alpha 1 |
| ENSMUSG00000052957 | 1.94 | 3.84 | 4.52E-06 | Gas1 | growth arrest specific 1 |
| ENSMUSG00000052837 | 1.94 | 3.84 | 4.93E-10 | Junb | jun B proto-oncogene |
| ENSMUSG00000044712 | 1.94 | 3.84 | 5.40E-22 | Slc38a6 | solute carrier family 38, member 6 |
| ENSMUSG00000087168 | 1.94 | 3.84 | 6.34E-03 | NA | NA |
| ENSMUSG00000044141 | 1.94 | 3.83 | 2.32E-03 | NA | NA |
| ENSMUSG00000058258 | 1.94 | 3.83 | 1.18E-04 | Idi1 | isopentenyl-diphosphate delta isomerase |
| ENSMUSG00000067736 | 1.94 | 3.83 | 6.35E-03 | NA | NA |
| ENSMUSG00000022887 | 1.94 | 3.83 | 1.81E-06 | Masp1 | mannan-binding lectin serine peptidase 1 |
| ENSMUSG00000007655 | 1.94 | 3.83 | 1.17E-10 | Cav1 | caveolin 1, caveolae protein |
| ENSMUSG00000032816 | 1.93 | 3.82 | 1.09E-09 | Igdcc4 | immunoglobulin superfamily, DCC subclass, member 4 |
| ENSMUSG00000067929 | 1.93 | 3.82 | 9.12E-07 | NA | NA |
| ENSMUSG00000059430 | 1.93 | 3.82 | 1.01E-13 | Actg2 | actin, gamma 2, smooth muscle, enteric |
| ENSMUSG00000039286 | 1.93 | 3.82 | 1.24E-06 | Fndc3b | fibronectin type III domain containing 3B |
| ENSMUSG00000025203 | 1.93 | 3.82 | 4.40E-19 | Scd2 | stearoyl-Coenzyme A desaturase 2 |
| ENSMUSG00000084910 | 1.93 | 3.82 | 3.49E-05 | NA | NA |
| ENSMUSG00000028047 | 1.93 | 3.81 | 1.31E-06 | Thbs3 | thrombospondin 3 |
| ENSMUSG00000052125 | 1.93 | 3.81 | 1.12E-06 | F730043M19Rik | RIKEN cDNA F730043M19 gene |
| ENSMUSG00000018199 | 1.93 | 3.81 | 2.54E-24 | Trove2 | TROVE domain family, member 2 |
| ENSMUSG00000036887 | 1.93 | 3.81 | 3.23E-17 | C1qa | complement component 1, q subcomponent, alpha polypeptide |
| ENSMUSG00000038722 | 1.93 | 3.80 | 6.80E-20 | Bud31 | BUD31 homolog (yeast) |
| ENSMUSG00000028015 | 1.92 | 3.79 | 2.23E-32 | Ctso | cathepsin O |
| ENSMUSG00000045106 | 1.92 | 3.79 | 6.75E-04 | NA | NA |
| ENSMUSG00000050914 | 1.92 | 3.79 | 5.33E-06 | Ankrd37 | ankyrin repeat domain 37 |
| ENSMUSG00000035107 | 1.92 | 3.79 | 1.57E-17 | Dcbld2 | discoidin, CUB and LCCL domain containing 2 |
| ENSMUSG00000052137 | 1.92 | 3.79 | 7.26E-13 | Rbm12b2 | RNA binding motif protein 12 B2 |
| ENSMUSG00000046999 | 1.92 | 3.79 | 6.67E-03 | 1110032F04Rik | RIKEN cDNA 1110032F04 gene |
| ENSMUSG00000080115 | 1.92 | 3.78 | 3.50E-05 | LOC100504608 | protein FAM119B-like |
| ENSMUSG00000074750 | 1.92 | 3.78 | 9.34E-14 | NA | NA |
| ENSMUSG00000058073 | 1.92 | 3.78 | 1.11E-06 | NA | NA |
| ENSMUSG00000038552 | 1.92 | 3.78 | 1.22E-10 | Fndc4 | fibronectin type III domain containing 4 |
| ENSMUSG00000048616 | 1.92 | 3.78 | 7.88E-06 | Nog | noggin |
| ENSMUSG00000028389 | 1.92 | 3.78 | 8.13E-06 | Zfp37 | zinc finger protein 37 |
| ENSMUSG00000079111 | 1.92 | 3.77 | 1.49E-21 | Kdelr2 | KDEL (Lys-Asp-Glu-Leu) endoplasmic reticulum protein retention receptor 2 |
| ENSMUSG00000020019 | 1.91 | 3.77 | 5.84E-12 | Ntn4 | netrin 4 |
| ENSMUSG00000032418 | 1.91 | 3.77 | 1.40E-11 | Me1 | malic enzyme 1, NADP(+)-dependent, cytosolic |
| ENSMUSG00000075307 | 1.91 | 3.77 | 6.62E-09 | Klhl41 | kelch-like 41 |
| ENSMUSG00000020431 | 1.91 | 3.77 | 5.22E-06 | Adcy1 | adenylate cyclase 1 |
| ENSMUSG00000049001 | 1.91 | 3.77 | 1.84E-15 | Ndnf | neuron-derived neurotrophic factor |
| ENSMUSG00000037362 | 1.91 | 3.77 | 5.57E-03 | Nov | nephroblastoma overexpressed gene |
| ENSMUSG00000044550 | 1.91 | 3.77 | 2.63E-05 | Tceal3 | transcription elongation factor A (SII)-like 3 |
| ENSMUSG00000076431 | 1.91 | 3.77 | 3.93E-12 | Sox4 | SRY (sex determining region Y)-box 4 |
| ENSMUSG00000089554 | 1.91 | 3.76 | 4.13E-04 | NA | NA |
| ENSMUSG00000001155 | 1.91 | 3.76 | 3.20E-03 | Ftcd | formiminotransferase cyclodeaminase |
| ENSMUSG00000018171 | 1.91 | 3.75 | 1.10E-19 | Vmp1 | vacuole membrane protein 1 |
| ENSMUSG00000024480 | 1.91 | 3.75 | 2.92E-06 | Ap3s1 | adaptor-related protein complex 3, sigma 1 subunit |
| ENSMUSG00000026458 | 1.91 | 3.75 | 3.49E-24 | Ppfia4 | protein tyrosine phosphatase, receptor type, f polypeptide (PTPRF), interacting protein (liprin), alpha 4 |
| ENSMUSG00000027996 | 1.91 | 3.75 | 3.04E-03 | Sfrp2 | secreted frizzled-related protein 2 |
| ENSMUSG00000045136 | 1.91 | 3.75 | 3.23E-08 | Tubb2b | tubulin, beta 2B class IIB |
| ENSMUSG00000029371 | 1.91 | 3.75 | 8.44E-08 | Cxcl5 | chemokine (C-X-C motif) ligand 5 |
| ENSMUSG00000041362 | 1.91 | 3.75 | 4.78E-12 | 4930506M07Rik | RIKEN cDNA 4930506M07 gene |
| ENSMUSG00000030256 | 1.91 | 3.75 | 3.03E-09 | Bhlhe41 | basic helix-loop-helix family, member e41 |
| ENSMUSG00000049891 | 1.90 | 3.74 | 6.19E-13 | NA | NA |
| ENSMUSG00000000031 | 1.90 | 3.74 | 8.81E-09 | NA | NA |
| ENSMUSG00000039530 | 1.90 | 3.74 | 1.87E-30 | Tusc3 | tumor suppressor candidate 3 |
| ENSMUSG00000022150 | 1.90 | 3.74 | 2.21E-23 | Dab2 | disabled 2, mitogen-responsive phosphoprotein |
| ENSMUSG00000023000 | 1.90 | 3.74 | 4.81E-04 | Dhh | desert hedgehog |
| ENSMUSG00000027221 | 1.90 | 3.73 | 3.34E-10 | Chst1 | carbohydrate (keratan sulfate Gal-6) sulfotransferase 1 |
| ENSMUSG00000007777 | 1.90 | 3.73 | 1.40E-18 | 0610009B22Rik | RIKEN cDNA 0610009B22 gene |
| ENSMUSG00000083022 | 1.90 | 3.73 | 3.51E-05 | NA | NA |
| ENSMUSG00000026573 | 1.90 | 3.72 | 6.08E-04 | Xcl1 | chemokine (C motif) ligand 1 |
| ENSMUSG00000041935 | 1.90 | 3.72 | 1.29E-08 | AW549877 | expressed sequence AW549877 |
| ENSMUSG00000042473 | 1.90 | 3.72 | 5.55E-21 | Tbc1d8b | TBC1 domain family, member 8B |
| ENSMUSG00000054580 | 1.89 | 3.72 | 2.87E-13 | Pla2r1 | phospholipase A2 receptor 1 |
| ENSMUSG00000034685 | 1.89 | 3.72 | 1.97E-05 | Fam171a2 | family with sequence similarity 171, member A2 |
| ENSMUSG00000030770 | 1.89 | 3.72 | 4.35E-25 | Parva | parvin, alpha |
| ENSMUSG00000032324 | 1.89 | 3.71 | 3.39E-21 | Tspan3 | tetraspanin 3 |
| ENSMUSG00000074140 | 1.89 | 3.71 | 1.79E-03 | NA | NA |
| ENSMUSG00000021390 | 1.89 | 3.71 | 1.95E-09 | Ogn | osteoglycin |
| ENSMUSG00000066324 | 1.89 | 3.71 | 1.03E-28 | Impad1 | inositol monophosphatase domain containing 1 |
| ENSMUSG00000045103 | 1.89 | 3.71 | 2.21E-10 | Dmd | dystrophin, muscular dystrophy |
| ENSMUSG00000039126 | 1.89 | 3.71 | 4.72E-05 | Prune2 | prune homolog 2 (Drosophila) |
| ENSMUSG00000035172 | 1.89 | 3.71 | 4.29E-08 | Plekhh3 | pleckstrin homology domain containing, family H (with MyTH4 domain) member 3 |
| ENSMUSG00000010307 | 1.89 | 3.70 | 1.05E-18 | Tmem86a | transmembrane protein 86A |
| ENSMUSG00000044600 | 1.89 | 3.70 | 7.31E-27 | Smim7 | small integral membrane protein 7 |
| ENSMUSG00000031347 | 1.89 | 3.70 | 1.93E-10 | Cetn2 | centrin 2 |
| ENSMUSG00000061039 | 1.89 | 3.70 | 8.70E-03 | Olfr920 | olfactory receptor 920 |
| ENSMUSG00000036036 | 1.89 | 3.70 | 6.38E-08 | Zfp57 | zinc finger protein 57 |
| ENSMUSG00000034164 | 1.89 | 3.70 | 5.83E-16 | Emid1 | EMI domain containing 1 |
| ENSMUSG00000030074 | 1.89 | 3.70 | 7.91E-11 | Gxylt2 | glucoside xylosyltransferase 2 |
| ENSMUSG00000063060 | 1.89 | 3.70 | 1.20E-11 | Sox7 | SRY (sex determining region Y)-box 7 |
| ENSMUSG00000028789 | 1.89 | 3.69 | 7.38E-13 | Adc | arginine decarboxylase |
| ENSMUSG00000024041 | 1.88 | 3.69 | 8.09E-03 | Cryaa | crystallin, alpha A |
| ENSMUSG00000040710 | 1.88 | 3.69 | 1.02E-16 | St8sia4 | ST8 alpha-N-acetyl-neuraminide alpha-2,8-sialyltransferase 4 |
| ENSMUSG00000024803 | 1.88 | 3.69 | 9.48E-03 | Ankrd1 | ankyrin repeat domain 1 (cardiac muscle) |
| ENSMUSG00000037548 | 1.88 | 3.69 | 1.53E-35 | H2-DMb2 | histocompatibility 2, class II, locus Mb2 |
| ENSMUSG00000060509 | 1.88 | 3.69 | 3.21E-05 | Xcr1 | chemokine (C motif) receptor 1 |
| ENSMUSG00000059921 | 1.88 | 3.69 | 1.07E-04 | Unc5c | unc-5 homolog C (C. elegans) |
| ENSMUSG00000086472 | 1.88 | 3.68 | 5.03E-03 | NA | NA |
| ENSMUSG00000052631 | 1.88 | 3.68 | 3.81E-04 | Sh2d6 | SH2 domain containing 6 |
| ENSMUSG00000054200 | 1.88 | 3.68 | 6.01E-03 | Ffar4 | free fatty acid receptor 4 |
| ENSMUSG00000060467 | 1.88 | 3.68 | 5.25E-07 | NA | NA |
| ENSMUSG00000049421 | 1.88 | 3.68 | 5.44E-23 | Zfp260 | zinc finger protein 260 |
| ENSMUSG00000024427 | 1.88 | 3.68 | 1.81E-18 | Spry4 | sprouty homolog 4 (Drosophila) |
| ENSMUSG00000070355 | 1.88 | 3.67 | 4.69E-04 | NA | NA |
| ENSMUSG00000075047 | 1.88 | 3.67 | 1.37E-03 | NA | NA |
| ENSMUSG00000027859 | 1.88 | 3.67 | 6.59E-05 | Ngf | nerve growth factor |
| ENSMUSG00000026103 | 1.88 | 3.67 | 1.39E-37 | Gls | glutaminase |
| ENSMUSG00000031375 | 1.87 | 3.67 | 5.11E-11 | Bgn | biglycan |
| ENSMUSG00000086882 | 1.87 | 3.66 | 3.96E-04 | NA | NA |
| ENSMUSG00000039497 | 1.87 | 3.66 | 1.22E-32 | Dse | dermatan sulfate epimerase |
| ENSMUSG00000038463 | 1.87 | 3.66 | 2.45E-18 | Olfml2b | olfactomedin-like 2B |
| ENSMUSG00000027366 | 1.87 | 3.66 | 9.42E-22 | Sppl2a | signal peptide peptidase like 2A |
| ENSMUSG00000020435 | 1.87 | 3.66 | 1.61E-07 | Osbp2 | oxysterol binding protein 2 |
| ENSMUSG00000074579 | 1.87 | 3.65 | 4.74E-04 | Lekr1 | leucine, glutamate and lysine rich 1 |
| ENSMUSG00000079419 | 1.87 | 3.65 | 2.12E-28 | Ms4a6c | membrane-spanning 4-domains, subfamily A, member 6C |
| ENSMUSG00000020007 | 1.87 | 3.65 | 1.90E-07 | Il20ra | interleukin 20 receptor, alpha |
| ENSMUSG00000024529 | 1.87 | 3.65 | 1.07E-07 | Lox | lysyl oxidase |
| ENSMUSG00000008398 | 1.87 | 3.65 | 1.63E-21 | Elk3 | ELK3, member of ETS oncogene family |
| ENSMUSG00000038174 | 1.87 | 3.65 | 1.41E-13 | Fam126b | family with sequence similarity 126, member B |
| ENSMUSG00000070565 | 1.87 | 3.65 | 6.13E-07 | Rasal2 | RAS protein activator like 2 |
| ENSMUSG00000006736 | 1.87 | 3.64 | 5.00E-18 | Tspan31 | tetraspanin 31 |
| ENSMUSG00000046785 | 1.87 | 3.64 | 1.28E-11 | Epm2aip1 | EPM2A (laforin) interacting protein 1 |
| ENSMUSG00000074232 | 1.86 | 3.64 | 1.90E-03 | NA | NA |
| ENSMUSG00000009378 | 1.86 | 3.64 | 3.49E-04 | Slc16a12 | solute carrier family 16 (monocarboxylic acid transporters), member 12 |
| ENSMUSG00000074345 | 1.86 | 3.64 | 9.52E-04 | Tnfaip8l3 | tumor necrosis factor, alpha-induced protein 8-like 3 |
| ENSMUSG00000070436 | 1.86 | 3.64 | 9.45E-22 | Serpinh1 | serine (or cysteine) peptidase inhibitor, clade H, member 1 |
| ENSMUSG00000085105 | 1.86 | 3.64 | 1.73E-03 | NA | NA |
| ENSMUSG00000058897 | 1.86 | 3.64 | 7.22E-15 | Col25a1 | collagen, type XXV, alpha 1 |
| ENSMUSG00000044757 | 1.86 | 3.64 | 6.81E-06 | NA | NA |
| ENSMUSG00000002266 | 1.86 | 3.63 | 7.88E-03 | Zim1 | zinc finger, imprinted 1 |
| ENSMUSG00000027848 | 1.86 | 3.63 | 7.14E-16 | Olfml3 | olfactomedin-like 3 |
| ENSMUSG00000067653 | 1.86 | 3.63 | 2.47E-07 | Ankrd23 | ankyrin repeat domain 23 |
| ENSMUSG00000031434 | 1.86 | 3.63 | 9.26E-10 | Morc4 | microrchidia 4 |
| ENSMUSG00000050967 | 1.86 | 3.63 | 4.15E-04 | Creg2 | cellular repressor of E1A-stimulated genes 2 |
| ENSMUSG00000013698 | 1.86 | 3.63 | 3.20E-16 | Pea15a | phosphoprotein enriched in astrocytes 15A |
| ENSMUSG00000090063 | 1.86 | 3.63 | 6.46E-07 | NA | NA |
| ENSMUSG00000073599 | 1.86 | 3.62 | 7.79E-09 | Ecscr | endothelial cell surface expressed chemotaxis and apoptosis regulator |
| ENSMUSG00000031343 | 1.86 | 3.62 | 1.29E-07 | Gabra3 | gamma-aminobutyric acid (GABA) A receptor, subunit alpha 3 |
| ENSMUSG00000020231 | 1.86 | 3.62 | 1.02E-11 | Dip2a | DIP2 disco-interacting protein 2 homolog A (Drosophila) |
| ENSMUSG00000070871 | 1.86 | 3.62 | 4.53E-16 | Ccnyl1 | cyclin Y-like 1 |
| ENSMUSG00000030787 | 1.85 | 3.62 | 2.41E-17 | Lyve1 | lymphatic vessel endothelial hyaluronan receptor 1 |
| ENSMUSG00000055301 | 1.85 | 3.62 | 2.40E-12 | Adh7 | alcohol dehydrogenase 7 (class IV), mu or sigma polypeptide |
| ENSMUSG00000049097 | 1.85 | 3.61 | 9.54E-04 | Ankrd34a | ankyrin repeat domain 34A |
| ENSMUSG00000074813 | 1.85 | 3.61 | 6.33E-11 | NA | NA |
| ENSMUSG00000042670 | 1.85 | 3.61 | 8.17E-06 | Immp1l | IMP1 inner mitochondrial membrane peptidase-like (S. cerevisiae) |
| ENSMUSG00000030107 | 1.85 | 3.61 | 2.50E-16 | Usp18 | ubiquitin specific peptidase 18 |
| ENSMUSG00000053038 | 1.85 | 3.61 | 1.16E-14 | NA | NA |
| ENSMUSG00000049719 | 1.85 | 3.61 | 1.53E-04 | Prss46 | protease, serine 46 |
| ENSMUSG00000016496 | 1.85 | 3.61 | 2.39E-19 | Cd274 | CD274 antigen |
| ENSMUSG00000021741 | 1.85 | 3.61 | 6.45E-06 | NA | NA |
| ENSMUSG00000026589 | 1.85 | 3.60 | 4.01E-17 | Sec16b | SEC16 homolog B (S. cerevisiae) |
| ENSMUSG00000020937 | 1.85 | 3.60 | 5.76E-15 | Plcd3 | phospholipase C, delta 3 |
| ENSMUSG00000071532 | 1.85 | 3.60 | 2.18E-04 | NA | NA |
| ENSMUSG00000006219 | 1.85 | 3.60 | 2.46E-19 | Fblim1 | filamin binding LIM protein 1 |
| ENSMUSG00000020176 | 1.85 | 3.60 | 1.80E-22 | Grb10 | growth factor receptor bound protein 10 |
| ENSMUSG00000074916 | 1.85 | 3.60 | 3.40E-10 | Chst14 | carbohydrate (N-acetylgalactosamine 4-0) sulfotransferase 14 |
| ENSMUSG00000048636 | 1.85 | 3.60 | 3.19E-03 | NA | NA |
| ENSMUSG00000089838 | 1.85 | 3.60 | 5.41E-10 | NA | NA |
| ENSMUSG00000042436 | 1.85 | 3.59 | 2.83E-06 | Mfap4 | microfibrillar-associated protein 4 |
| ENSMUSG00000025894 | 1.85 | 3.59 | 3.07E-15 | Aasdhppt | aminoadipate-semialdehyde dehydrogenase-phosphopantetheinyl transferase |
| ENSMUSG00000061848 | 1.85 | 3.59 | 1.12E-08 | NA | NA |
| ENSMUSG00000032714 | 1.84 | 3.59 | 8.59E-33 | Syde1 | synapse defective 1, Rho GTPase, homolog 1 (C. elegans) |
| ENSMUSG00000025104 | 1.84 | 3.59 | 3.92E-13 | Hdgfrp3 | hepatoma-derived growth factor, related protein 3 |
| ENSMUSG00000026228 | 1.84 | 3.59 | 7.34E-04 | Htr2b | 5-hydroxytryptamine (serotonin) receptor 2B |
| ENSMUSG00000008450 | 1.84 | 3.59 | 3.93E-04 | NA | NA |
| ENSMUSG00000034780 | 1.84 | 3.59 | 1.63E-07 | B3galt1 | UDP-Gal:betaGlcNAc beta 1,3-galactosyltransferase, polypeptide 1 |
| ENSMUSG00000019558 | 1.84 | 3.58 | 5.94E-12 | Slc6a8 | solute carrier family 6 (neurotransmitter transporter, creatine), member 8 |
| ENSMUSG00000033361 | 1.84 | 3.58 | 5.40E-10 | Prrg3 | proline rich Gla (G-carboxyglutamic acid) 3 (transmembrane) |
| ENSMUSG00000036545 | 1.84 | 3.58 | 8.52E-21 | Adamts2 | a disintegrin-like and metallopeptidase (reprolysin type) with thrombospondin type 1 motif, 2 |
| ENSMUSG00000075312 | 1.84 | 3.58 | 4.54E-14 | NA | NA |
| ENSMUSG00000021732 | 1.84 | 3.58 | 3.47E-03 | Fgf10 | fibroblast growth factor 10 |
| ENSMUSG00000058600 | 1.84 | 3.58 | 3.90E-23 | NA | NA |
| ENSMUSG00000025402 | 1.84 | 3.58 | 3.48E-08 | Nab2 | Ngfi-A binding protein 2 |
| ENSMUSG00000069808 | 1.84 | 3.58 | 6.17E-18 | Fam57a | family with sequence similarity 57, member A |
| ENSMUSG00000038128 | 1.84 | 3.58 | 1.31E-08 | Camk4 | calcium/calmodulin-dependent protein kinase IV |
| ENSMUSG00000044217 | 1.84 | 3.58 | 2.16E-03 | Aqp5 | aquaporin 5 |
| ENSMUSG00000007207 | 1.84 | 3.57 | 1.14E-13 | Stx1a | syntaxin 1A (brain) |
| ENSMUSG00000044350 | 1.84 | 3.57 | 1.15E-14 | Lacc1 | laccase (multicopper oxidoreductase) domain containing 1 |
| ENSMUSG00000026479 | 1.84 | 3.57 | 3.93E-08 | Lamc2 | laminin, gamma 2 |
| ENSMUSG00000026749 | 1.84 | 3.57 | 1.25E-18 | Nek6 | NIMA (never in mitosis gene a)-related expressed kinase 6 |
| ENSMUSG00000062014 | 1.84 | 3.57 | 3.05E-42 | Gmfb | glia maturation factor, beta |
| ENSMUSG00000026768 | 1.83 | 3.57 | 1.12E-05 | Itga8 | integrin alpha 8 |
| ENSMUSG00000041476 | 1.83 | 3.57 | 3.39E-06 | Smpx | small muscle protein, X-linked |
| ENSMUSG00000087658 | 1.83 | 3.56 | 8.23E-05 | NA | NA |
| ENSMUSG00000070424 | 1.83 | 3.56 | 9.11E-15 | Art5 | ADP-ribosyltransferase 5 |
| ENSMUSG00000019791 | 1.83 | 3.56 | 4.29E-14 | Hint3 | histidine triad nucleotide binding protein 3 |
| ENSMUSG00000021549 | 1.83 | 3.56 | 2.14E-11 | Rasa1 | RAS p21 protein activator 1 |
| ENSMUSG00000087573 | 1.83 | 3.56 | 1.07E-05 | NA | NA |
| ENSMUSG00000044134 | 1.83 | 3.55 | 1.87E-14 | Fam109a | family with sequence similarity 109, member A |
| ENSMUSG00000041731 | 1.83 | 3.55 | 6.67E-07 | Pgm5 | phosphoglucomutase 5 |
| ENSMUSG00000028610 | 1.83 | 3.55 | 2.57E-03 | Dmrtb1 | DMRT-like family B with proline-rich C-terminal, 1 |
| ENSMUSG00000042717 | 1.83 | 3.55 | 3.67E-06 | Ppp1r3a | protein phosphatase 1, regulatory (inhibitor) subunit 3A |
| ENSMUSG00000042505 | 1.83 | 3.55 | 9.71E-13 | Acn9 | ACN9 homolog (S. cerevisiae) |
| ENSMUSG00000073591 | 1.83 | 3.55 | 2.37E-08 | Pcdhb22 | protocadherin beta 22 |
| ENSMUSG00000026673 | 1.83 | 3.55 | 7.81E-12 | NA | NA |
| ENSMUSG00000039699 | 1.83 | 3.55 | 1.40E-06 | Batf2 | basic leucine zipper transcription factor, ATF-like 2 |
| ENSMUSG00000044162 | 1.83 | 3.55 | 2.24E-03 | Tnip3 | TNFAIP3 interacting protein 3 |
| ENSMUSG00000089682 | 1.83 | 3.55 | 2.24E-32 | Bcl2l2 | BCL2-like 2 |
| ENSMUSG00000035713 | 1.83 | 3.55 | 5.55E-04 | Usp35 | ubiquitin specific peptidase 35 |
| ENSMUSG00000026888 | 1.83 | 3.55 | 3.78E-15 | Grb14 | growth factor receptor bound protein 14 |
| ENSMUSG00000056073 | 1.83 | 3.55 | 4.31E-03 | Grik2 | glutamate receptor, ionotropic, kainate 2 (beta 2) |
| ENSMUSG00000069792 | 1.82 | 3.54 | 1.42E-07 | Wfdc17 | WAP four-disulfide core domain 17 |
| ENSMUSG00000036781 | 1.82 | 3.54 | 2.42E-09 | Rps27l | ribosomal protein S27-like |
| ENSMUSG00000031250 | 1.82 | 3.54 | 1.17E-04 | Tnmd | tenomodulin |
| ENSMUSG00000002233 | 1.82 | 3.54 | 5.62E-17 | Rhoc | ras homolog gene family, member C |
| ENSMUSG00000005054 | 1.82 | 3.54 | 1.73E-16 | Cstb | cystatin B |
| ENSMUSG00000014773 | 1.82 | 3.54 | 2.39E-11 | Dll1 | delta-like 1 (Drosophila) |
| ENSMUSG00000080848 | 1.82 | 3.53 | 5.16E-03 | NA | NA |
| ENSMUSG00000078862 | 1.82 | 3.53 | 1.27E-11 | Gm14326 | predicted gene 14326 |
| ENSMUSG00000047676 | 1.82 | 3.53 | 1.18E-06 | NA | NA |
| ENSMUSG00000042429 | 1.82 | 3.53 | 2.63E-05 | Adora1 | adenosine A1 receptor |
| ENSMUSG00000037490 | 1.82 | 3.53 | 4.34E-06 | Slc2a12 | solute carrier family 2 (facilitated glucose transporter), member 12 |
| ENSMUSG00000048776 | 1.82 | 3.53 | 7.13E-04 | Pthlh | parathyroid hormone-like peptide |
| ENSMUSG00000052143 | 1.82 | 3.53 | 5.89E-06 | NA | NA |
| ENSMUSG00000025608 | 1.82 | 3.53 | 6.66E-21 | Podxl | podocalyxin-like |
| ENSMUSG00000034591 | 1.82 | 3.52 | 1.09E-32 | Slc41a2 | solute carrier family 41, member 2 |
| ENSMUSG00000036957 | 1.82 | 3.52 | 2.14E-07 | Lrfn3 | leucine rich repeat and fibronectin type III domain containing 3 |
| ENSMUSG00000064634 | 1.82 | 3.52 | 4.78E-03 | NA | NA |
| ENSMUSG00000037962 | 1.82 | 3.52 | 2.38E-03 | Fam101a | family with sequence similarity 101, member A |
| ENSMUSG00000025856 | 1.82 | 3.52 | 4.30E-15 | Pdgfa | platelet derived growth factor, alpha |
| ENSMUSG00000022246 | 1.81 | 3.52 | 6.63E-18 | Rai14 | retinoic acid induced 14 |
| ENSMUSG00000031197 | 1.81 | 3.52 | 2.50E-09 | Vbp1 | von Hippel-Lindau binding protein 1 |
| ENSMUSG00000004151 | 1.81 | 3.52 | 4.40E-12 | NA | NA |
| ENSMUSG00000060550 | 1.81 | 3.51 | 4.93E-16 | NA | NA |
| ENSMUSG00000041548 | 1.81 | 3.51 | 1.03E-08 | Hspb8 | heat shock protein 8 |
| ENSMUSG00000002728 | 1.81 | 3.51 | 1.03E-28 | Naa20 | N(alpha)-acetyltransferase 20, NatB catalytic subunit |
| ENSMUSG00000036896 | 1.81 | 3.51 | 2.80E-14 | C1qc | complement component 1, q subcomponent, C chain |
| ENSMUSG00000006958 | 1.81 | 3.50 | 8.44E-14 | Chrd | chordin |
| ENSMUSG00000022528 | 1.81 | 3.50 | 4.78E-12 | Hes1 | hairy and enhancer of split 1 (Drosophila) |
| ENSMUSG00000032180 | 1.81 | 3.50 | 1.49E-09 | Tmed1 | transmembrane emp24 domain containing 1 |
| ENSMUSG00000071533 | 1.81 | 3.50 | 1.68E-18 | Pcnp | PEST proteolytic signal containing nuclear protein |
| ENSMUSG00000031604 | 1.81 | 3.50 | 9.32E-07 | Sc4mol | sterol-C4-methyl oxidase-like |
| ENSMUSG00000032251 | 1.81 | 3.50 | 3.18E-10 | Irak1bp1 | interleukin-1 receptor-associated kinase 1 binding protein 1 |
| ENSMUSG00000026478 | 1.81 | 3.50 | 5.13E-15 | Lamc1 | laminin, gamma 1 |
| ENSMUSG00000053080 | 1.81 | 3.50 | 2.05E-26 | 2700081O15Rik | RIKEN cDNA 2700081O15 gene |
| ENSMUSG00000090222 | 1.81 | 3.50 | 1.46E-03 | NA | NA |
| ENSMUSG00000055818 | 1.81 | 3.50 | 4.71E-03 | NA | NA |
| ENSMUSG00000050490 | 1.81 | 3.50 | 2.33E-24 | NA | NA |
| ENSMUSG00000042804 | 1.80 | 3.49 | 7.11E-11 | Gpr153 | G protein-coupled receptor 153 |
| ENSMUSG00000085401 | 1.80 | 3.49 | 1.75E-08 | NA | NA |
| ENSMUSG00000043870 | 1.80 | 3.49 | 6.52E-10 | NA | NA |
| ENSMUSG00000001089 | 1.80 | 3.49 | 8.09E-22 | Luzp1 | leucine zipper protein 1 |
| ENSMUSG00000030605 | 1.80 | 3.49 | 1.81E-07 | Mfge8 | milk fat globule-EGF factor 8 protein |
| ENSMUSG00000029070 | 1.80 | 3.49 | 4.85E-13 | Mxra8 | matrix-remodelling associated 8 |
| ENSMUSG00000001467 | 1.80 | 3.49 | 1.82E-14 | Cyp51 | cytochrome P450, family 51 |
| ENSMUSG00000063594 | 1.80 | 3.49 | 2.97E-04 | Gng8 | guanine nucleotide binding protein (G protein), gamma 8 |
| ENSMUSG00000048521 | 1.80 | 3.48 | 9.75E-05 | Cxcr6 | chemokine (C-X-C motif) receptor 6 |
| ENSMUSG00000082247 | 1.80 | 3.48 | 8.44E-04 | NA | NA |
| ENSMUSG00000084390 | 1.80 | 3.48 | 2.20E-04 | NA | NA |
| ENSMUSG00000085316 | 1.80 | 3.48 | 1.28E-03 | D330050G23Rik | RIKEN cDNA D330050G23 gene |
| ENSMUSG00000024414 | 1.80 | 3.48 | 1.02E-16 | Mrpl27 | mitochondrial ribosomal protein L27 |
| ENSMUSG00000014303 | 1.80 | 3.48 | 1.59E-09 | Glis2 | GLIS family zinc finger 2 |
| ENSMUSG00000025265 | 1.80 | 3.48 | 1.57E-12 | Fgd1 | FYVE, RhoGEF and PH domain containing 1 |
| ENSMUSG00000061619 | 1.80 | 3.48 | 7.88E-03 | NA | NA |
| ENSMUSG00000004709 | 1.80 | 3.48 | 8.15E-25 | Cd244 | CD244 natural killer cell receptor 2B4 |
| ENSMUSG00000049414 | 1.80 | 3.48 | 3.52E-05 | NA | NA |
| ENSMUSG00000027792 | 1.80 | 3.47 | 5.96E-03 | Bche | butyrylcholinesterase |
| ENSMUSG00000089797 | 1.80 | 3.47 | 6.38E-04 | NA | NA |
| ENSMUSG00000031070 | 1.79 | 3.47 | 6.21E-05 | Mrgprf | MAS-related GPR, member F |
| ENSMUSG00000028445 | 1.79 | 3.47 | 2.11E-08 | Enho | energy homeostasis associated |
| ENSMUSG00000054178 | 1.79 | 3.47 | 5.80E-03 | NA | NA |
| ENSMUSG00000083465 | 1.79 | 3.47 | 7.77E-21 | NA | NA |
| ENSMUSG00000005124 | 1.79 | 3.46 | 1.58E-16 | Wisp1 | WNT1 inducible signaling pathway protein 1 |
| ENSMUSG00000043991 | 1.79 | 3.46 | 4.31E-23 | Pura | purine rich element binding protein A |
| ENSMUSG00000025059 | 1.79 | 3.46 | 5.46E-13 | Gyk | glycerol kinase |
| ENSMUSG00000049044 | 1.79 | 3.46 | 9.03E-11 | Rapgef4 | Rap guanine nucleotide exchange factor (GEF) 4 |
| ENSMUSG00000026153 | 1.79 | 3.46 | 8.87E-11 | Fam135a | family with sequence similarity 135, member A |
| ENSMUSG00000045790 | 1.79 | 3.46 | 7.50E-03 | Ccdc149 | coiled-coil domain containing 149 |
| ENSMUSG00000027861 | 1.79 | 3.45 | 4.05E-04 | Casq2 | calsequestrin 2 |
| ENSMUSG00000025529 | 1.79 | 3.45 | 1.87E-03 | Zfp711 | zinc finger protein 711 |
| ENSMUSG00000029096 | 1.79 | 3.45 | 2.27E-10 | Htra3 | HtrA serine peptidase 3 |
| ENSMUSG00000005973 | 1.79 | 3.45 | 6.14E-11 | Rcn1 | reticulocalbin 1 |
| ENSMUSG00000039163 | 1.79 | 3.45 | 4.41E-08 | Cmc1 | COX assembly mitochondrial protein 1 |
| ENSMUSG00000057604 | 1.79 | 3.45 | 8.21E-12 | Lmcd1 | LIM and cysteine-rich domains 1 |
| ENSMUSG00000030754 | 1.79 | 3.45 | 4.51E-45 | Copb1 | coatomer protein complex, subunit beta 1 |
| ENSMUSG00000032932 | 1.78 | 3.45 | 1.22E-20 | Hspa13 | heat shock protein 70 family, member 13 |
| ENSMUSG00000072594 | 1.78 | 3.44 | 7.73E-10 | NA | NA |
| ENSMUSG00000027611 | 1.78 | 3.44 | 5.16E-12 | Procr | protein C receptor, endothelial |
| ENSMUSG00000073716 | 1.78 | 3.44 | 1.96E-03 | NA | NA |
| ENSMUSG00000048222 | 1.78 | 3.44 | 1.39E-08 | NA | NA |
| ENSMUSG00000008333 | 1.78 | 3.44 | 1.34E-18 | Snrpb2 | U2 small nuclear ribonucleoprotein B |
| ENSMUSG00000053110 | 1.78 | 3.44 | 1.08E-24 | Yap1 | yes-associated protein 1 |
| ENSMUSG00000062397 | 1.78 | 3.43 | 4.45E-05 | NA | NA |
| ENSMUSG00000069899 | 1.78 | 3.43 | 8.10E-10 | NA | NA |
| ENSMUSG00000005836 | 1.78 | 3.43 | 5.23E-03 | Gata6 | GATA binding protein 6 |
| ENSMUSG00000002565 | 1.78 | 3.42 | 1.24E-04 | Scin | scinderin |
| ENSMUSG00000041779 | 1.78 | 3.42 | 2.53E-26 | Tram2 | translocating chain-associating membrane protein 2 |
| ENSMUSG00000033953 | 1.78 | 3.42 | 3.04E-24 | Ppp3r1 | protein phosphatase 3, regulatory subunit B, alpha isoform (calcineurin B, type I) |
| ENSMUSG00000050212 | 1.77 | 3.42 | 1.92E-07 | Eva1b | eva-1 homolog B (C. elegans) |
| ENSMUSG00000056271 | 1.77 | 3.42 | 1.63E-05 | Lman1l | lectin, mannose-binding 1 like |
| ENSMUSG00000054314 | 1.77 | 3.42 | 1.42E-04 | NA | NA |
| ENSMUSG00000028410 | 1.77 | 3.42 | 1.12E-07 | Dnaja1 | DnaJ (Hsp40) homolog, subfamily A, member 1 |
| ENSMUSG00000024998 | 1.77 | 3.42 | 4.53E-10 | Plce1 | phospholipase C, epsilon 1 |
| ENSMUSG00000067189 | 1.77 | 3.41 | 1.28E-12 | NA | NA |
| ENSMUSG00000085128 | 1.77 | 3.41 | 7.75E-03 | NA | NA |
| ENSMUSG00000028583 | 1.77 | 3.41 | 6.84E-06 | NA | NA |
| ENSMUSG00000031465 | 1.77 | 3.41 | 1.12E-18 | Angpt2 | angiopoietin 2 |
| ENSMUSG00000014329 | 1.77 | 3.41 | 8.33E-07 | Bicc1 | bicaudal C homolog 1 (Drosophila) |
| ENSMUSG00000041075 | 1.77 | 3.41 | 1.49E-15 | Fzd7 | frizzled homolog 7 (Drosophila) |
| ENSMUSG00000031673 | 1.77 | 3.41 | 3.38E-16 | Cdh11 | cadherin 11 |
| ENSMUSG00000063687 | 1.77 | 3.40 | 2.05E-04 | Pcdhb5 | protocadherin beta 5 |
| ENSMUSG00000051185 | 1.77 | 3.40 | 2.77E-09 | Fam174a | family with sequence similarity 174, member A |
| ENSMUSG00000000938 | 1.77 | 3.40 | 4.09E-10 | Hoxa10 | homeobox A10 |
| ENSMUSG00000071855 | 1.77 | 3.40 | 3.73E-05 | Ccdc112 | coiled-coil domain containing 112 |
| ENSMUSG00000017446 | 1.76 | 3.40 | 2.81E-26 | C1qtnf1 | C1q and tumor necrosis factor related protein 1 |
| ENSMUSG00000034930 | 1.76 | 3.40 | 9.93E-06 | Rtkn | rhotekin |
| ENSMUSG00000026154 | 1.76 | 3.40 | 7.58E-10 | 1110058L19Rik | RIKEN cDNA 1110058L19 gene |
| ENSMUSG00000035158 | 1.76 | 3.39 | 4.50E-13 | Mitf | microphthalmia-associated transcription factor |
| ENSMUSG00000042439 | 1.76 | 3.39 | 7.51E-18 | Zfp532 | zinc finger protein 532 |
| ENSMUSG00000046761 | 1.76 | 3.39 | 2.25E-09 | Fam83h | family with sequence similarity 83, member H |
| ENSMUSG00000060530 | 1.76 | 3.39 | 2.84E-03 | NA | NA |
| ENSMUSG00000045761 | 1.76 | 3.39 | 4.64E-04 | Fam179a | family with sequence similarity 179, member A |
| ENSMUSG00000062937 | 1.76 | 3.39 | 7.96E-13 | Mtap | methylthioadenosine phosphorylase |
| ENSMUSG00000039478 | 1.76 | 3.39 | 3.86E-07 | Micu3 | mitochondrial calcium uptake family, member 3 |
| ENSMUSG00000033955 | 1.76 | 3.39 | 1.11E-17 | Tnks1bp1 | tankyrase 1 binding protein 1 |
| ENSMUSG00000023022 | 1.76 | 3.39 | 2.21E-25 | Lima1 | LIM domain and actin binding 1 |
| ENSMUSG00000070327 | 1.76 | 3.39 | 1.19E-22 | Rnf213 | ring finger protein 213 |
| ENSMUSG00000031608 | 1.76 | 3.39 | 4.65E-15 | Galnt7 | UDP-N-acetyl-alpha-D-galactosamine: polypeptide N-acetylgalactosaminyltransferase 7 |
| ENSMUSG00000035236 | 1.76 | 3.38 | 1.61E-12 | Scai | suppressor of cancer cell invasion |
| ENSMUSG00000071568 | 1.76 | 3.38 | 2.58E-06 | NA | NA |
| ENSMUSG00000019997 | 1.76 | 3.38 | 1.12E-07 | Ctgf | connective tissue growth factor |
| ENSMUSG00000028719 | 1.76 | 3.38 | 1.97E-15 | Cmpk1 | cytidine monophosphate (UMP-CMP) kinase 1 |
| ENSMUSG00000029403 | 1.76 | 3.38 | 8.37E-13 | Cdkl2 | cyclin-dependent kinase-like 2 (CDC2-related kinase) |
| ENSMUSG00000078108 | 1.76 | 3.38 | 3.54E-12 | NA | NA |
| ENSMUSG00000016327 | 1.76 | 3.38 | 3.65E-04 | Atp1b4 | ATPase, (Na+)/K+ transporting, beta 4 polypeptide |
| ENSMUSG00000035878 | 1.76 | 3.38 | 4.22E-05 | Hykk | hydroxylysine kinase 1 |
| ENSMUSG00000000901 | 1.76 | 3.38 | 2.27E-12 | Mmp11 | matrix metallopeptidase 11 |
| ENSMUSG00000029655 | 1.75 | 3.38 | 1.42E-13 | N4bp2l2 | NEDD4 binding protein 2-like 2 |
| ENSMUSG00000024610 | 1.75 | 3.38 | 1.22E-19 | Cd74 | CD74 antigen (invariant polypeptide of major histocompatibility complex, class II antigen-associated) |
| ENSMUSG00000021950 | 1.75 | 3.37 | 2.98E-03 | Anxa8 | annexin A8 |
| ENSMUSG00000055697 | 1.75 | 3.37 | 2.83E-03 | NA | NA |
| ENSMUSG00000032966 | 1.75 | 3.37 | 9.41E-12 | Fkbp1a | FK506 binding protein 1a |
| ENSMUSG00000071317 | 1.75 | 3.37 | 4.74E-12 | Bves | blood vessel epicardial substance |
| ENSMUSG00000065987 | 1.75 | 3.37 | 5.68E-03 | Cd209b | CD209b antigen |
| ENSMUSG00000067219 | 1.75 | 3.37 | 2.39E-03 | Nipal1 | NIPA-like domain containing 1 |
| ENSMUSG00000036676 | 1.75 | 3.37 | 5.00E-12 | Tmtc3 | transmembrane and tetratricopeptide repeat containing 3 |
| ENSMUSG00000021796 | 1.75 | 3.36 | 2.02E-22 | Bmpr1a | bone morphogenetic protein receptor, type 1A |
| ENSMUSG00000032194 | 1.75 | 3.36 | 2.13E-15 | Kank2 | KN motif and ankyrin repeat domains 2 |
| ENSMUSG00000021278 | 1.75 | 3.36 | 6.74E-03 | Amn | amnionless |
| ENSMUSG00000051736 | 1.75 | 3.36 | 3.05E-05 | Fam229b | family with sequence similarity 229, member B |
| ENSMUSG00000057322 | 1.75 | 3.36 | 2.01E-07 | Rpl38 | ribosomal protein L38 |
| ENSMUSG00000020105 | 1.75 | 3.36 | 1.95E-11 | Lrig3 | leucine-rich repeats and immunoglobulin-like domains 3 |
| ENSMUSG00000089636 | 1.75 | 3.36 | 5.92E-08 | NA | NA |
| ENSMUSG00000022893 | 1.75 | 3.36 | 8.65E-15 | Adamts1 | a disintegrin-like and metallopeptidase (reprolysin type) with thrombospondin type 1 motif, 1 |
| ENSMUSG00000075118 | 1.75 | 3.36 | 8.24E-15 | NA | NA |
| ENSMUSG00000084899 | 1.75 | 3.35 | 8.50E-04 | NA | NA |
| ENSMUSG00000045498 | 1.74 | 3.35 | 1.42E-05 | Pcdhb3 | protocadherin beta 3 |
| ENSMUSG00000025151 | 1.74 | 3.35 | 8.09E-21 | Maged1 | melanoma antigen, family D, 1 |
| ENSMUSG00000021214 | 1.74 | 3.35 | 4.39E-05 | Akr1c18 | aldo-keto reductase family 1, member C18 |
| ENSMUSG00000036743 | 1.74 | 3.35 | 2.58E-05 | Psma8 | proteasome (prosome, macropain) subunit, alpha type, 8 |
| ENSMUSG00000029090 | 1.74 | 3.35 | 1.17E-16 | Gpr125 | G protein-coupled receptor 125 |
| ENSMUSG00000059422 | 1.74 | 3.35 | 7.85E-11 | NA | NA |
| ENSMUSG00000015085 | 1.74 | 3.35 | 7.40E-08 | Entpd2 | ectonucleoside triphosphate diphosphohydrolase 2 |
| ENSMUSG00000048503 | 1.74 | 3.35 | 1.44E-07 | Tmem136 | transmembrane protein 136 |
| ENSMUSG00000070514 | 1.74 | 3.35 | 7.50E-08 | NA | NA |
| ENSMUSG00000030516 | 1.74 | 3.35 | 5.42E-15 | Tjp1 | tight junction protein 1 |
| ENSMUSG00000054057 | 1.74 | 3.34 | 5.79E-07 | A930004D18Rik | RIKEN cDNA A930004D18 gene |
| ENSMUSG00000025464 | 1.74 | 3.34 | 7.96E-09 | Paox | polyamine oxidase (exo-N4-amino) |
| ENSMUSG00000060671 | 1.74 | 3.34 | 1.75E-22 | Atp8b2 | ATPase, class I, type 8B, member 2 |
| ENSMUSG00000080021 | 1.74 | 3.34 | 2.30E-05 | NA | NA |
| ENSMUSG00000030149 | 1.74 | 3.34 | 2.01E-05 | Klrk1 | killer cell lectin-like receptor subfamily K, member 1 |
| ENSMUSG00000039239 | 1.74 | 3.33 | 3.81E-09 | Tgfb2 | transforming growth factor, beta 2 |
| ENSMUSG00000060913 | 1.74 | 3.33 | 1.40E-07 | Trim55 | tripartite motif-containing 55 |
| ENSMUSG00000045573 | 1.74 | 3.33 | 4.69E-05 | Penk | preproenkephalin |
| ENSMUSG00000054404 | 1.74 | 3.33 | 9.57E-09 | Slfn5 | schlafen 5 |
| ENSMUSG00000027499 | 1.74 | 3.33 | 3.71E-05 | Pkia | protein kinase inhibitor, alpha |
| ENSMUSG00000025352 | 1.73 | 3.33 | 1.06E-13 | Gdf11 | growth differentiation factor 11 |
| ENSMUSG00000074046 | 1.73 | 3.33 | 2.99E-04 | NA | NA |
| ENSMUSG00000026358 | 1.73 | 3.33 | 1.98E-04 | Rgs1 | regulator of G-protein signaling 1 |
| ENSMUSG00000029767 | 1.73 | 3.32 | 1.96E-19 | Calu | calumenin |
| ENSMUSG00000022131 | 1.73 | 3.32 | 4.19E-17 | Gpr180 | G protein-coupled receptor 180 |
| ENSMUSG00000046191 | 1.73 | 3.32 | 2.71E-06 | Pcdhb20 | protocadherin beta 20 |
| ENSMUSG00000058571 | 1.73 | 3.32 | 2.43E-14 | Gpc6 | glypican 6 |
| ENSMUSG00000029992 | 1.73 | 3.32 | 1.01E-39 | Gfpt1 | glutamine fructose-6-phosphate transaminase 1 |
| ENSMUSG00000038507 | 1.73 | 3.31 | 3.30E-19 | Parp12 | poly (ADP-ribose) polymerase family, member 12 |
| ENSMUSG00000085945 | 1.73 | 3.31 | 1.77E-05 | NA | NA |
| ENSMUSG00000062098 | 1.73 | 3.31 | 8.47E-20 | Btbd3 | BTB (POZ) domain containing 3 |
| ENSMUSG00000070501 | 1.73 | 3.31 | 2.08E-11 | BC094916 | cDNA sequence BC094916 |
| ENSMUSG00000041025 | 1.73 | 3.31 | 7.34E-18 | Iffo2 | intermediate filament family orphan 2 |
| ENSMUSG00000056199 | 1.73 | 3.31 | 4.57E-11 | NA | NA |
| ENSMUSG00000038301 | 1.73 | 3.31 | 6.81E-13 | Snx10 | sorting nexin 10 |
| ENSMUSG00000042190 | 1.73 | 3.31 | 9.30E-14 | Cmklr1 | chemokine-like receptor 1 |
| ENSMUSG00000003070 | 1.72 | 3.31 | 8.72E-06 | Efna2 | ephrin A2 |
| ENSMUSG00000086084 | 1.72 | 3.30 | 1.57E-03 | NA | NA |
| ENSMUSG00000023032 | 1.72 | 3.30 | 3.36E-10 | Slc4a8 | solute carrier family 4 (anion exchanger), member 8 |
| ENSMUSG00000078735 | 1.72 | 3.30 | 7.16E-03 | NA | NA |
| ENSMUSG00000019933 | 1.72 | 3.30 | 6.46E-05 | 2310015B20Rik | RIKEN cDNA 2310015B20 gene |
| ENSMUSG00000001348 | 1.72 | 3.30 | 3.44E-11 | Acp5 | acid phosphatase 5, tartrate resistant |
| ENSMUSG00000015733 | 1.72 | 3.30 | 1.26E-26 | Capza2 | capping protein (actin filament) muscle Z-line, alpha 2 |
| ENSMUSG00000071871 | 1.72 | 3.30 | 5.33E-06 | NA | NA |
| ENSMUSG00000046879 | 1.72 | 3.30 | 2.53E-23 | Irgm1 | immunity-related GTPase family M member 1 |
| ENSMUSG00000031217 | 1.72 | 3.30 | 3.77E-14 | Efnb1 | ephrin B1 |
| ENSMUSG00000026659 | 1.72 | 3.30 | 3.90E-13 | Dusp12 | dual specificity phosphatase 12 |
| ENSMUSG00000025129 | 1.72 | 3.30 | 1.05E-03 | Ppp1r27 | protein phosphatase 1, regulatory subunit 27 |
| ENSMUSG00000047992 | 1.72 | 3.30 | 2.05E-06 | Fam69c | family with sequence similarity 69, member C |
| ENSMUSG00000084888 | 1.72 | 3.30 | 1.61E-03 | NA | NA |
| ENSMUSG00000073374 | 1.72 | 3.30 | 4.77E-05 | NA | NA |
| ENSMUSG00000001020 | 1.72 | 3.30 | 4.55E-16 | S100a4 | S100 calcium binding protein A4 |
| ENSMUSG00000039911 | 1.72 | 3.30 | 1.29E-11 | Spsb1 | splA/ryanodine receptor domain and SOCS box containing 1 |
| ENSMUSG00000071414 | 1.72 | 3.30 | 7.94E-05 | NA | NA |
| ENSMUSG00000034738 | 1.72 | 3.30 | 9.43E-07 | Nostrin | nitric oxide synthase trafficker |
| ENSMUSG00000079306 | 1.72 | 3.30 | 7.21E-03 | NA | NA |
| ENSMUSG00000046818 | 1.72 | 3.29 | 1.69E-06 | Ddit4l | DNA-damage-inducible transcript 4-like |
| ENSMUSG00000040867 | 1.72 | 3.29 | 3.93E-08 | Begain | brain-enriched guanylate kinase-associated |
| ENSMUSG00000075394 | 1.72 | 3.29 | 8.29E-06 | Hoxc4 | homeobox C4 |
| ENSMUSG00000050029 | 1.72 | 3.29 | 6.95E-20 | Rap2c | RAP2C, member of RAS oncogene family |
| ENSMUSG00000075376 | 1.72 | 3.28 | 3.78E-18 | Rc3h2 | ring finger and CCCH-type zinc finger domains 2 |
| ENSMUSG00000042743 | 1.72 | 3.28 | 2.62E-11 | Sgtb | small glutamine-rich tetratricopeptide repeat (TPR)-containing, beta |
| ENSMUSG00000049556 | 1.71 | 3.28 | 1.04E-04 | Lingo1 | leucine rich repeat and Ig domain containing 1 |
| ENSMUSG00000050248 | 1.71 | 3.28 | 2.05E-15 | Evc2 | Ellis van Creveld syndrome 2 |
| ENSMUSG00000027552 | 1.71 | 3.28 | 2.17E-08 | E2f5 | E2F transcription factor 5 |
| ENSMUSG00000060572 | 1.71 | 3.28 | 6.07E-12 | Mfap2 | microfibrillar-associated protein 2 |
| ENSMUSG00000048027 | 1.71 | 3.28 | 3.17E-14 | Rgmb | repulsive guidance molecule family member B |
| ENSMUSG00000051790 | 1.71 | 3.27 | 8.56E-09 | Nlgn2 | neuroligin 2 |
| ENSMUSG00000044921 | 1.71 | 3.27 | 5.60E-06 | Rassf9 | Ras association (RalGDS/AF-6) domain family (N-terminal) member 9 |
| ENSMUSG00000061410 | 1.71 | 3.27 | 2.94E-14 | Zcchc14 | zinc finger, CCHC domain containing 14 |
| ENSMUSG00000010721 | 1.71 | 3.27 | 9.87E-17 | Lmbr1 | limb region 1 |
| ENSMUSG00000044763 | 1.71 | 3.27 | 1.94E-16 | Trmt10c | tRNA methyltransferase 10C |
| ENSMUSG00000011008 | 1.71 | 3.27 | 1.13E-10 | Mcoln2 | mucolipin 2 |
| ENSMUSG00000032220 | 1.71 | 3.27 | 1.24E-06 | Myo1e | myosin IE |
| ENSMUSG00000041180 | 1.71 | 3.27 | 9.85E-06 | Hectd2 | HECT domain containing 2 |
| ENSMUSG00000027957 | 1.71 | 3.27 | 9.87E-16 | Slc35a3 | solute carrier family 35 (UDP-N-acetylglucosamine (UDP-GlcNAc) transporter), member 3 |
| ENSMUSG00000031342 | 1.71 | 3.27 | 1.56E-15 | Gpm6b | glycoprotein m6b |
| ENSMUSG00000028145 | 1.71 | 3.27 | 3.70E-11 | Them4 | thioesterase superfamily member 4 |
| ENSMUSG00000029919 | 1.71 | 3.26 | 4.17E-13 | Hpgds | hematopoietic prostaglandin D synthase |
| ENSMUSG00000032186 | 1.70 | 3.26 | 5.39E-08 | Tmod2 | tropomodulin 2 |
| ENSMUSG00000069372 | 1.70 | 3.26 | 1.22E-04 | Ctxn3 | cortexin 3 |
| ENSMUSG00000020467 | 1.70 | 3.26 | 4.72E-04 | Efemp1 | epidermal growth factor-containing fibulin-like extracellular matrix protein 1 |
| ENSMUSG00000057530 | 1.70 | 3.26 | 8.87E-32 | Ece1 | endothelin converting enzyme 1 |
| ENSMUSG00000041144 | 1.70 | 3.26 | 4.57E-03 | NA | NA |
| ENSMUSG00000038418 | 1.70 | 3.26 | 2.06E-05 | Egr1 | early growth response 1 |
| ENSMUSG00000021103 | 1.70 | 3.26 | 2.21E-11 | Mnat1 | menage a trois 1 |
| ENSMUSG00000016494 | 1.70 | 3.25 | 1.75E-14 | Cd34 | CD34 antigen |
| ENSMUSG00000034422 | 1.70 | 3.25 | 9.15E-45 | Parp14 | poly (ADP-ribose) polymerase family, member 14 |
| ENSMUSG00000021130 | 1.70 | 3.25 | 3.58E-07 | Galnt16 | UDP-N-acetyl-alpha-D-galactosamine:polypeptide N-acetylgalactosaminyltransferase 16 |
| ENSMUSG00000050910 | 1.70 | 3.25 | 4.56E-18 | Cdr2l | cerebellar degeneration-related protein 2-like |
| ENSMUSG00000037735 | 1.70 | 3.25 | 5.68E-03 | NA | NA |
| ENSMUSG00000073987 | 1.70 | 3.25 | 6.96E-11 | Ggh | gamma-glutamyl hydrolase |
| ENSMUSG00000086298 | 1.70 | 3.25 | 1.22E-05 | NA | NA |
| ENSMUSG00000032002 | 1.70 | 3.25 | 2.39E-19 | Dcun1d5 | DCN1, defective in cullin neddylation 1, domain containing 5 (S. cerevisiae) |
| ENSMUSG00000002983 | 1.70 | 3.25 | 6.04E-14 | Relb | avian reticuloendotheliosis viral (v-rel) oncogene related B |
| ENSMUSG00000033722 | 1.70 | 3.25 | 1.21E-11 | BC034090 | cDNA sequence BC034090 |
| ENSMUSG00000031734 | 1.70 | 3.24 | 1.17E-08 | Irx3 | Iroquois related homeobox 3 |
| ENSMUSG00000025355 | 1.70 | 3.24 | 1.42E-10 | Mmp19 | matrix metallopeptidase 19 |
| ENSMUSG00000066880 | 1.70 | 3.24 | 1.06E-10 | Zfp617 | zinc finger protein 617 |
| ENSMUSG00000004317 | 1.70 | 3.24 | 5.56E-15 | Clcn5 | chloride channel 5 |
| ENSMUSG00000026235 | 1.70 | 3.24 | 4.72E-11 | Epha4 | Eph receptor A4 |
| ENSMUSG00000022199 | 1.69 | 3.24 | 2.28E-08 | Slc22a17 | solute carrier family 22 (organic cation transporter), member 17 |
| ENSMUSG00000035923 | 1.69 | 3.23 | 7.98E-09 | Myf6 | myogenic factor 6 |
| ENSMUSG00000053070 | 1.69 | 3.23 | 1.40E-03 | 9230110C19Rik | RIKEN cDNA 9230110C19 gene |
| ENSMUSG00000032649 | 1.69 | 3.23 | 2.74E-04 | Colgalt2 | collagen beta(1-O)galactosyltransferase 2 |
| ENSMUSG00000049477 | 1.69 | 3.23 | 1.23E-08 | NA | NA |
| ENSMUSG00000043885 | 1.69 | 3.23 | 1.52E-15 | Slc36a4 | solute carrier family 36 (proton/amino acid symporter), member 4 |
| ENSMUSG00000054690 | 1.69 | 3.22 | 1.69E-27 | Emcn | endomucin |
| ENSMUSG00000037475 | 1.69 | 3.22 | 5.21E-13 | Thoc2 | THO complex 2 |
| ENSMUSG00000033799 | 1.69 | 3.22 | 6.95E-16 | BC016423 | cDNA sequence BC016423 |
| ENSMUSG00000005656 | 1.69 | 3.22 | 1.60E-26 | Snx6 | sorting nexin 6 |
| ENSMUSG00000038607 | 1.69 | 3.22 | 1.77E-20 | Gng10 | guanine nucleotide binding protein (G protein), gamma 10 |
| ENSMUSG00000049580 | 1.68 | 3.21 | 4.18E-09 | Tsku | tsukushi |
| ENSMUSG00000025511 | 1.68 | 3.21 | 1.17E-10 | Tspan4 | tetraspanin 4 |
| ENSMUSG00000026748 | 1.68 | 3.21 | 6.00E-14 | Plxdc2 | plexin domain containing 2 |
| ENSMUSG00000027412 | 1.68 | 3.21 | 2.33E-14 | Lpin3 | lipin 3 |
| ENSMUSG00000024750 | 1.68 | 3.21 | 4.53E-13 | Zfand5 | zinc finger, AN1-type domain 5 |
| ENSMUSG00000051703 | 1.68 | 3.21 | 4.06E-07 | Tmem198 | transmembrane protein 198 |
| ENSMUSG00000033268 | 1.68 | 3.21 | 3.39E-03 | Duox1 | dual oxidase 1 |
| ENSMUSG00000021765 | 1.68 | 3.21 | 1.02E-14 | Fst | follistatin |
| ENSMUSG00000049225 | 1.68 | 3.21 | 2.17E-11 | Pdp1 | pyruvate dehyrogenase phosphatase catalytic subunit 1 |
| ENSMUSG00000053399 | 1.68 | 3.21 | 2.75E-03 | Adamts18 | a disintegrin-like and metallopeptidase (reprolysin type) with thrombospondin type 1 motif, 18 |
| ENSMUSG00000001158 | 1.68 | 3.20 | 1.13E-10 | Snrnp27 | small nuclear ribonucleoprotein 27 (U4/U6.U5) |
| ENSMUSG00000042453 | 1.68 | 3.20 | 6.49E-21 | Reln | reelin |
| ENSMUSG00000051456 | 1.68 | 3.20 | 4.06E-05 | Hspb3 | heat shock protein 3 |
| ENSMUSG00000079343 | 1.68 | 3.20 | 1.33E-07 | Gm5077 | predicted gene 5077 |
| ENSMUSG00000024242 | 1.68 | 3.20 | 1.43E-12 | Map4k3 | mitogen-activated protein kinase kinase kinase kinase 3 |
| ENSMUSG00000040268 | 1.68 | 3.20 | 8.73E-26 | Plekha1 | pleckstrin homology domain containing, family A (phosphoinositide binding specific) member 1 |
| ENSMUSG00000022842 | 1.68 | 3.19 | 1.36E-14 | Ece2 | endothelin converting enzyme 2 |
| ENSMUSG00000087259 | 1.67 | 3.19 | 3.95E-03 | 2610035D17Rik | RIKEN cDNA 2610035D17 gene |
| ENSMUSG00000007594 | 1.67 | 3.19 | 1.07E-04 | Hapln4 | hyaluronan and proteoglycan link protein 4 |
| ENSMUSG00000080316 | 1.67 | 3.19 | 2.41E-07 | Spaca6 | sperm acrosome associated 6 |
| ENSMUSG00000022434 | 1.67 | 3.19 | 5.74E-25 | Fam118a | family with sequence similarity 118, member A |
| ENSMUSG00000085766 | 1.67 | 3.19 | 1.04E-04 | NA | NA |
| ENSMUSG00000069763 | 1.67 | 3.18 | 4.20E-06 | Tmem100 | transmembrane protein 100 |
| ENSMUSG00000073405 | 1.67 | 3.18 | 1.33E-05 | NA | NA |
| ENSMUSG00000037347 | 1.67 | 3.18 | 5.91E-06 | Chst7 | carbohydrate (N-acetylglucosamino) sulfotransferase 7 |
| ENSMUSG00000025140 | 1.67 | 3.18 | 3.20E-10 | Pycr1 | pyrroline-5-carboxylate reductase 1 |
| ENSMUSG00000038936 | 1.67 | 3.18 | 1.41E-08 | Sccpdh | saccharopine dehydrogenase (putative) |
| ENSMUSG00000032224 | 1.67 | 3.18 | 8.98E-04 | Fam81a | family with sequence similarity 81, member A |
| ENSMUSG00000032423 | 1.67 | 3.18 | 9.02E-15 | Syncrip | synaptotagmin binding, cytoplasmic RNA interacting protein |
| ENSMUSG00000033102 | 1.67 | 3.17 | 8.23E-04 | Cdc14b | CDC14 cell division cycle 14B |
| ENSMUSG00000064918 | 1.67 | 3.17 | 9.77E-03 | NA | NA |
| ENSMUSG00000022629 | 1.67 | 3.17 | 3.57E-07 | Kif21a | kinesin family member 21A |
| ENSMUSG00000019876 | 1.67 | 3.17 | 8.18E-10 | Pkib | protein kinase inhibitor beta, cAMP dependent, testis specific |
| ENSMUSG00000018846 | 1.67 | 3.17 | 1.04E-08 | Pank3 | pantothenate kinase 3 |
| ENSMUSG00000054978 | 1.66 | 3.17 | 4.91E-03 | Kbtbd13 | kelch repeat and BTB (POZ) domain containing 13 |
| ENSMUSG00000037686 | 1.66 | 3.17 | 6.26E-11 | Aspg | asparaginase homolog (S. cerevisiae) |
| ENSMUSG00000040711 | 1.66 | 3.17 | 6.05E-13 | Sh3pxd2b | SH3 and PX domains 2B |
| ENSMUSG00000079076 | 1.66 | 3.17 | 3.48E-03 | Gm3086 | RuvB-like protein 1 pseudogene |
| ENSMUSG00000003541 | 1.66 | 3.16 | 1.78E-14 | Ier3 | immediate early response 3 |
| ENSMUSG00000021745 | 1.66 | 3.16 | 5.32E-13 | Ptprg | protein tyrosine phosphatase, receptor type, G |
| ENSMUSG00000032363 | 1.66 | 3.16 | 2.30E-10 | Adamts7 | a disintegrin-like and metallopeptidase (reprolysin type) with thrombospondin type 1 motif, 7 |
| ENSMUSG00000061371 | 1.66 | 3.16 | 1.77E-06 | Zfp873 | zinc finger protein 873 |
| ENSMUSG00000063564 | 1.66 | 3.16 | 2.60E-08 | Col23a1 | collagen, type XXIII, alpha 1 |
| ENSMUSG00000055675 | 1.66 | 3.16 | 5.34E-29 | Kbtbd11 | kelch repeat and BTB (POZ) domain containing 11 |
| ENSMUSG00000051934 | 1.66 | 3.15 | 1.98E-13 | Spats2 | spermatogenesis associated, serine-rich 2 |
| ENSMUSG00000019891 | 1.66 | 3.15 | 2.30E-17 | Dcbld1 | discoidin, CUB and LCCL domain containing 1 |
| ENSMUSG00000030452 | 1.66 | 3.15 | 2.56E-26 | Nipa2 | non imprinted in Prader-Willi/Angelman syndrome 2 homolog (human) |
| ENSMUSG00000047819 | 1.65 | 3.15 | 3.58E-06 | Tigd4 | tigger transposable element derived 4 |
| ENSMUSG00000044424 | 1.65 | 3.15 | 6.80E-15 | NA | NA |
| ENSMUSG00000048779 | 1.65 | 3.15 | 1.19E-07 | P2ry6 | pyrimidinergic receptor P2Y, G-protein coupled, 6 |
| ENSMUSG00000056153 | 1.65 | 3.15 | 3.38E-33 | Socs6 | suppressor of cytokine signaling 6 |
| ENSMUSG00000020902 | 1.65 | 3.15 | 5.29E-07 | Ntn1 | netrin 1 |
| ENSMUSG00000022601 | 1.65 | 3.14 | 4.65E-07 | Zbtb11 | zinc finger and BTB domain containing 11 |
| ENSMUSG00000025938 | 1.65 | 3.14 | 3.55E-06 | Slco5a1 | solute carrier organic anion transporter family, member 5A1 |
| ENSMUSG00000027533 | 1.65 | 3.14 | 8.26E-08 | Fabp5 | fatty acid binding protein 5, epidermal |
| ENSMUSG00000060002 | 1.65 | 3.14 | 2.88E-13 | Chpt1 | choline phosphotransferase 1 |
| ENSMUSG00000080902 | 1.65 | 3.14 | 7.08E-04 | NA | NA |
| ENSMUSG00000060703 | 1.65 | 3.14 | 2.07E-18 | Cd302 | CD302 antigen |
| ENSMUSG00000047307 | 1.65 | 3.14 | 1.06E-03 | Pcdhb13 | protocadherin beta 13 |
| ENSMUSG00000073674 | 1.65 | 3.13 | 2.45E-08 | NA | NA |
| ENSMUSG00000045062 | 1.65 | 3.13 | 4.33E-08 | Pcdhb7 | protocadherin beta 7 |
| ENSMUSG00000020828 | 1.65 | 3.13 | 7.81E-20 | Pld2 | phospholipase D2 |
| ENSMUSG00000030259 | 1.65 | 3.13 | 4.75E-06 | Rassf8 | Ras association (RalGDS/AF-6) domain family (N-terminal) member 8 |
| ENSMUSG00000051375 | 1.65 | 3.13 | 9.84E-13 | Pcdh1 | protocadherin 1 |
| ENSMUSG00000039178 | 1.65 | 3.13 | 3.82E-11 | Tbc1d19 | TBC1 domain family, member 19 |
| ENSMUSG00000053666 | 1.65 | 3.13 | 5.29E-04 | NA | NA |
| ENSMUSG00000030353 | 1.65 | 3.13 | 2.93E-06 | Tead4 | TEA domain family member 4 |
| ENSMUSG00000024043 | 1.64 | 3.13 | 4.41E-16 | Arhgap28 | Rho GTPase activating protein 28 |
| ENSMUSG00000001053 | 1.64 | 3.13 | 4.95E-14 | N4bp3 | NEDD4 binding protein 3 |
| ENSMUSG00000023266 | 1.64 | 3.13 | 3.97E-06 | Frs3 | fibroblast growth factor receptor substrate 3 |
| ENSMUSG00000021319 | 1.64 | 3.13 | 3.05E-21 | Sfrp4 | secreted frizzled-related protein 4 |
| ENSMUSG00000028364 | 1.64 | 3.12 | 1.32E-09 | Tnc | tenascin C |
| ENSMUSG00000042515 | 1.64 | 3.12 | 2.28E-06 | Mum1l1 | melanoma associated antigen (mutated) 1-like 1 |
| ENSMUSG00000046463 | 1.64 | 3.12 | 4.67E-03 | NA | NA |
| ENSMUSG00000045045 | 1.64 | 3.12 | 5.55E-07 | Lrfn4 | leucine rich repeat and fibronectin type III domain containing 4 |
| ENSMUSG00000032184 | 1.64 | 3.12 | 2.04E-07 | Lysmd2 | LysM, putative peptidoglycan-binding, domain containing 2 |
| ENSMUSG00000028293 | 1.64 | 3.11 | 1.75E-34 | Slc35a1 | solute carrier family 35 (CMP-sialic acid transporter), member 1 |
| ENSMUSG00000037104 | 1.64 | 3.11 | 5.66E-18 | Socs5 | suppressor of cytokine signaling 5 |
| ENSMUSG00000063727 | 1.64 | 3.11 | 9.90E-07 | Tnfrsf11b | tumor necrosis factor receptor superfamily, member 11b (osteoprotegerin) |
| ENSMUSG00000022681 | 1.64 | 3.11 | 1.47E-21 | Ntan1 | N-terminal Asn amidase |
| ENSMUSG00000027770 | 1.64 | 3.11 | 3.45E-16 | Dhx36 | DEAH (Asp-Glu-Ala-His) box polypeptide 36 |
| ENSMUSG00000027822 | 1.64 | 3.11 | 4.39E-21 | Slc33a1 | solute carrier family 33 (acetyl-CoA transporter), member 1 |
| ENSMUSG00000058690 | 1.64 | 3.11 | 1.46E-13 | Ccser2 | coiled-coil serine rich 2 |
| ENSMUSG00000029279 | 1.64 | 3.11 | 5.23E-05 | Brdt | bromodomain, testis-specific |
| ENSMUSG00000017607 | 1.64 | 3.11 | 1.74E-06 | Tns4 | tensin 4 |
| ENSMUSG00000032340 | 1.63 | 3.10 | 4.57E-13 | Neo1 | neogenin |
| ENSMUSG00000060743 | 1.63 | 3.10 | 1.75E-08 | NA | NA |
| ENSMUSG00000027713 | 1.63 | 3.10 | 1.96E-03 | 1810062G17Rik | RIKEN cDNA 1810062G17 gene |
| ENSMUSG00000030255 | 1.63 | 3.10 | 3.89E-15 | Sspn | sarcospan |
| ENSMUSG00000024143 | 1.63 | 3.10 | 3.74E-20 | Rhoq | ras homolog gene family, member Q |
| ENSMUSG00000029781 | 1.63 | 3.10 | 4.88E-11 | Fkbp9 | FK506 binding protein 9 |
| ENSMUSG00000027995 | 1.63 | 3.10 | 3.01E-11 | Tlr2 | toll-like receptor 2 |
| ENSMUSG00000032320 | 1.63 | 3.10 | 2.19E-08 | Rcn2 | reticulocalbin 2 |
| ENSMUSG00000020086 | 1.63 | 3.09 | 1.67E-08 | H2afy2 | H2A histone family, member Y2 |
| ENSMUSG00000028970 | 1.63 | 3.09 | 6.92E-23 | Abcb1b | ATP-binding cassette, sub-family B (MDR/TAP), member 1B |
| ENSMUSG00000022525 | 1.63 | 3.09 | 3.52E-07 | Hrasls | HRAS-like suppressor |
| ENSMUSG00000078311 | 1.63 | 3.09 | 5.92E-05 | NA | NA |
| ENSMUSG00000032611 | 1.63 | 3.09 | 6.28E-03 | 1700102P08Rik | RIKEN cDNA 1700102P08 gene |
| ENSMUSG00000052563 | 1.63 | 3.09 | 4.64E-08 | D930048N14Rik | RIKEN cDNA D930048N14 gene |
| ENSMUSG00000073144 | 1.63 | 3.09 | 4.48E-07 | 4930599N23Rik | RIKEN cDNA 4930599N23 gene |
| ENSMUSG00000001657 | 1.63 | 3.09 | 5.76E-04 | Hoxc8 | homeobox C8 |
| ENSMUSG00000027015 | 1.63 | 3.09 | 1.77E-14 | Cybrd1 | cytochrome b reductase 1 |
| ENSMUSG00000071604 | 1.63 | 3.08 | 1.36E-12 | Fam189a2 | family with sequence similarity 189, member A2 |
| ENSMUSG00000019256 | 1.62 | 3.08 | 8.94E-07 | Ahr | aryl-hydrocarbon receptor |
| ENSMUSG00000002308 | 1.62 | 3.08 | 1.42E-11 | Cd320 | CD320 antigen |
| ENSMUSG00000020282 | 1.62 | 3.08 | 4.95E-12 | Rhbdf1 | rhomboid family 1 (Drosophila) |
| ENSMUSG00000057835 | 1.62 | 3.08 | 2.18E-04 | Zfp119a | zinc finger protein 119a |
| ENSMUSG00000029769 | 1.62 | 3.08 | 5.42E-06 | Ccdc136 | coiled-coil domain containing 136 |
| ENSMUSG00000008892 | 1.62 | 3.08 | 7.32E-09 | Vdac3 | voltage-dependent anion channel 3 |
| ENSMUSG00000027347 | 1.62 | 3.08 | 2.87E-09 | Rasgrp1 | RAS guanyl releasing protein 1 |
| ENSMUSG00000080002 | 1.62 | 3.08 | 7.91E-13 | NA | NA |
| ENSMUSG00000021917 | 1.62 | 3.07 | 7.66E-17 | Spcs1 | signal peptidase complex subunit 1 homolog (S. cerevisiae) |
| ENSMUSG00000085396 | 1.62 | 3.07 | 1.21E-12 | NA | NA |
| ENSMUSG00000051451 | 1.62 | 3.07 | 3.51E-06 | Crebzf | CREB/ATF bZIP transcription factor |
| ENSMUSG00000046434 | 1.62 | 3.07 | 4.97E-12 | NA | NA |
| ENSMUSG00000054519 | 1.62 | 3.07 | 5.92E-07 | Zfp867 | zinc finger protein 867 |
| ENSMUSG00000051427 | 1.62 | 3.07 | 1.72E-14 | Ccdc157 | coiled-coil domain containing 157 |
| ENSMUSG00000017615 | 1.62 | 3.07 | 4.40E-28 | Tnfaip1 | tumor necrosis factor, alpha-induced protein 1 (endothelial) |
| ENSMUSG00000022912 | 1.62 | 3.07 | 9.70E-21 | Pros1 | protein S (alpha) |
| ENSMUSG00000020484 | 1.61 | 3.06 | 3.09E-17 | Xbp1 | X-box binding protein 1 |
| ENSMUSG00000035954 | 1.61 | 3.06 | 5.41E-10 | Dock4 | dedicator of cytokinesis 4 |
| ENSMUSG00000036103 | 1.61 | 3.06 | 1.90E-12 | Colec12 | collectin sub-family member 12 |
| ENSMUSG00000028700 | 1.61 | 3.06 | 1.64E-18 | Pomgnt1 | protein O-linked mannose beta 1,2-N-acetylglucosaminyltransferase |
| ENSMUSG00000039607 | 1.61 | 3.05 | 3.87E-05 | Rbms3 | RNA binding motif, single stranded interacting protein |
| ENSMUSG00000056116 | 1.61 | 3.05 | 2.46E-19 | NA | NA |
| ENSMUSG00000030862 | 1.61 | 3.05 | 2.83E-04 | Cpxm2 | carboxypeptidase X 2 (M14 family) |
| ENSMUSG00000089940 | 1.61 | 3.05 | 7.56E-05 | NA | NA |
| ENSMUSG00000031875 | 1.61 | 3.05 | 1.39E-17 | Cmtm3 | CKLF-like MARVEL transmembrane domain containing 3 |
| ENSMUSG00000033436 | 1.61 | 3.05 | 2.80E-18 | Armcx2 | armadillo repeat containing, X-linked 2 |
| ENSMUSG00000025810 | 1.61 | 3.05 | 2.96E-10 | Nrp1 | neuropilin 1 |
| ENSMUSG00000032537 | 1.61 | 3.05 | 4.25E-07 | Ephb1 | Eph receptor B1 |
| ENSMUSG00000080716 | 1.61 | 3.05 | 7.32E-05 | NA | NA |
| ENSMUSG00000034487 | 1.61 | 3.04 | 1.87E-08 | Kdelc2 | KDEL (Lys-Asp-Glu-Leu) containing 2 |
| ENSMUSG00000034910 | 1.61 | 3.04 | 3.48E-17 | Pygo1 | pygopus 1 |
| ENSMUSG00000004562 | 1.61 | 3.04 | 2.27E-17 | Arhgef40 | Rho guanine nucleotide exchange factor (GEF) 40 |
| ENSMUSG00000027199 | 1.61 | 3.04 | 7.38E-45 | Gatm | glycine amidinotransferase (L-arginine:glycine amidinotransferase) |
| ENSMUSG00000019122 | 1.61 | 3.04 | 6.28E-10 | Ccl9 | chemokine (C-C motif) ligand 9 |
| ENSMUSG00000038181 | 1.60 | 3.04 | 4.80E-12 | Chpf2 | chondroitin polymerizing factor 2 |
| ENSMUSG00000087381 | 1.60 | 3.04 | 6.46E-03 | NA | NA |
| ENSMUSG00000033082 | 1.60 | 3.04 | 2.60E-07 | Clec1a | C-type lectin domain family 1, member a |
| ENSMUSG00000067924 | 1.60 | 3.04 | 8.11E-15 | NA | NA |
| ENSMUSG00000015396 | 1.60 | 3.04 | 4.94E-05 | Cd83 | CD83 antigen |
| ENSMUSG00000072762 | 1.60 | 3.04 | 1.96E-04 | NA | NA |
| ENSMUSG00000080198 | 1.60 | 3.04 | 2.26E-03 | NA | NA |
| ENSMUSG00000048280 | 1.60 | 3.04 | 4.17E-04 | Zfp738 | zinc finger protein 738 |
| ENSMUSG00000026585 | 1.60 | 3.04 | 6.79E-19 | Kifap3 | kinesin-associated protein 3 |
| ENSMUSG00000054034 | 1.60 | 3.04 | 1.48E-06 | Tceal5 | transcription elongation factor A (SII)-like 5 |
| ENSMUSG00000042340 | 1.60 | 3.04 | 1.75E-03 | Ctf1 | cardiotrophin 1 |
| ENSMUSG00000003418 | 1.60 | 3.03 | 6.95E-05 | St8sia6 | ST8 alpha-N-acetyl-neuraminide alpha-2,8-sialyltransferase 6 |
| ENSMUSG00000025940 | 1.60 | 3.03 | 3.67E-20 | Tmem70 | transmembrane protein 70 |
| ENSMUSG00000075271 | 1.60 | 3.03 | 1.59E-05 | Ttc30a1 | tetratricopeptide repeat domain 30A1 |
| ENSMUSG00000042501 | 1.60 | 3.03 | 2.46E-04 | Cpa6 | carboxypeptidase A6 |
| ENSMUSG00000070371 | 1.60 | 3.03 | 2.46E-10 | Prss36 | protease, serine 36 |
| ENSMUSG00000071656 | 1.60 | 3.03 | 1.40E-04 | Lrrn4cl | LRRN4 C-terminal like |
| ENSMUSG00000086782 | 1.60 | 3.02 | 2.44E-07 | E130102H24Rik | RIKEN cDNA E130102H24 gene |
| ENSMUSG00000003355 | 1.60 | 3.02 | 1.62E-06 | Fkbp11 | FK506 binding protein 11 |
| ENSMUSG00000030921 | 1.60 | 3.02 | 2.39E-15 | Trim30a | tripartite motif-containing 30A |
| ENSMUSG00000014177 | 1.60 | 3.02 | 4.27E-13 | Tvp23b | trans-golgi network vesicle protein 23B |
| ENSMUSG00000030789 | 1.60 | 3.02 | 5.02E-05 | Itgax | integrin alpha X |
| ENSMUSG00000073418 | 1.60 | 3.02 | 1.83E-11 | C4b | complement component 4B (Chido blood group) |
| ENSMUSG00000040158 | 1.60 | 3.02 | 1.11E-11 | Tax1bp3 | Tax1 (human T cell leukemia virus type I) binding protein 3 |
| ENSMUSG00000031169 | 1.59 | 3.02 | 4.94E-10 | Porcn | porcupine homolog (Drosophila) |
| ENSMUSG00000038648 | 1.59 | 3.02 | 3.51E-17 | Creb3l2 | cAMP responsive element binding protein 3-like 2 |
| ENSMUSG00000044811 | 1.59 | 3.02 | 1.07E-22 | AF251705 | cDNA sequence AF251705 |
| ENSMUSG00000084844 | 1.59 | 3.02 | 3.36E-04 | NA | NA |
| ENSMUSG00000072632 | 1.59 | 3.02 | 2.11E-04 | NA | NA |
| ENSMUSG00000032121 | 1.59 | 3.02 | 4.00E-13 | Tmem218 | transmembrane protein 218 |
| ENSMUSG00000048416 | 1.59 | 3.02 | 3.27E-05 | Mlf1 | myeloid leukemia factor 1 |
| ENSMUSG00000025809 | 1.59 | 3.02 | 1.30E-29 | Itgb1 | integrin beta 1 (fibronectin receptor beta) |
| ENSMUSG00000048440 | 1.59 | 3.01 | 4.27E-18 | Cyp4f16 | cytochrome P450, family 4, subfamily f, polypeptide 16 |
| ENSMUSG00000030022 | 1.59 | 3.01 | 1.19E-07 | Adamts9 | a disintegrin-like and metallopeptidase (reprolysin type) with thrombospondin type 1 motif, 9 |
| ENSMUSG00000087672 | 1.59 | 3.01 | 8.01E-08 | NA | NA |
| ENSMUSG00000048376 | 1.59 | 3.01 | 1.37E-10 | F2r | coagulation factor II (thrombin) receptor |
| ENSMUSG00000042523 | 1.59 | 3.01 | 2.33E-14 | Dnal1 | dynein, axonemal, light chain 1 |
| ENSMUSG00000015094 | 1.59 | 3.01 | 2.31E-12 | Npdc1 | neural proliferation, differentiation and control 1 |
| ENSMUSG00000028845 | 1.59 | 3.01 | 3.07E-04 | Tekt2 | tektin 2 |
| ENSMUSG00000022799 | 1.59 | 3.01 | 2.12E-13 | Arhgap31 | Rho GTPase activating protein 31 |
| ENSMUSG00000038530 | 1.59 | 3.01 | 5.25E-12 | Rgs4 | regulator of G-protein signaling 4 |
| ENSMUSG00000043068 | 1.59 | 3.00 | 1.32E-03 | Fam89a | family with sequence similarity 89, member A |
| ENSMUSG00000002228 | 1.59 | 3.00 | 6.09E-09 | Ppm1j | protein phosphatase 1J |
| ENSMUSG00000022074 | 1.59 | 3.00 | 1.00E-11 | Tnfrsf10b | tumor necrosis factor receptor superfamily, member 10b |
| ENSMUSG00000022244 | 1.59 | 3.00 | 2.96E-09 | Amacr | alpha-methylacyl-CoA racemase |
| ENSMUSG00000033446 | 1.59 | 3.00 | 1.12E-10 | Lpar6 | lysophosphatidic acid receptor 6 |
| ENSMUSG00000000266 | 1.59 | 3.00 | 5.54E-13 | Mid2 | midline 2 |
| ENSMUSG00000026971 | 1.58 | 3.00 | 5.65E-04 | Itgb6 | integrin beta 6 |
| ENSMUSG00000020407 | 1.58 | 3.00 | 1.47E-11 | Upp1 | uridine phosphorylase 1 |
| ENSMUSG00000024548 | 1.58 | 3.00 | 3.30E-09 | Setbp1 | SET binding protein 1 |
| ENSMUSG00000051236 | 1.58 | 3.00 | 1.07E-08 | Msrb3 | methionine sulfoxide reductase B3 |
| ENSMUSG00000029158 | 1.58 | 2.99 | 5.27E-06 | Yipf7 | Yip1 domain family, member 7 |
| ENSMUSG00000068794 | 1.58 | 2.99 | 9.38E-05 | Col28a1 | collagen, type XXVIII, alpha 1 |
| ENSMUSG00000034640 | 1.58 | 2.99 | 1.43E-14 | Tiparp | TCDD-inducible poly(ADP-ribose) polymerase |
| ENSMUSG00000039043 | 1.58 | 2.99 | 1.05E-11 | 2610034B18Rik | RIKEN cDNA 2610034B18 gene |
| ENSMUSG00000027855 | 1.58 | 2.99 | 7.98E-03 | Sycp1 | synaptonemal complex protein 1 |
| ENSMUSG00000034435 | 1.58 | 2.99 | 1.80E-03 | Tmem30b | transmembrane protein 30B |
| ENSMUSG00000083836 | 1.58 | 2.99 | 1.83E-04 | NA | NA |
| ENSMUSG00000027341 | 1.58 | 2.99 | 2.24E-14 | Tmem230 | transmembrane protein 230 |
| ENSMUSG00000028348 | 1.58 | 2.99 | 6.23E-05 | Murc | muscle-related coiled-coil protein |
| ENSMUSG00000073371 | 1.58 | 2.98 | 1.55E-03 | NA | NA |
| ENSMUSG00000024736 | 1.58 | 2.98 | 1.12E-08 | Tmem132a | transmembrane protein 132A |
| ENSMUSG00000036862 | 1.58 | 2.98 | 7.69E-10 | Dchs1 | dachsous 1 (Drosophila) |
| ENSMUSG00000026796 | 1.58 | 2.98 | 7.11E-08 | Fam129b | family with sequence similarity 129, member B |
| ENSMUSG00000082154 | 1.58 | 2.98 | 9.60E-06 | NA | NA |
| ENSMUSG00000050493 | 1.58 | 2.98 | 4.40E-14 | Fam167b | family with sequence similarity 167, member B |
| ENSMUSG00000089808 | 1.58 | 2.98 | 5.08E-03 | NA | NA |
| ENSMUSG00000020810 | 1.57 | 2.98 | 3.11E-05 | Cygb | cytoglobin |
| ENSMUSG00000070570 | 1.57 | 2.98 | 6.27E-03 | Slc17a7 | solute carrier family 17 (sodium-dependent inorganic phosphate cotransporter), member 7 |
| ENSMUSG00000048807 | 1.57 | 2.97 | 2.72E-09 | Slc35e4 | solute carrier family 35, member E4 |
| ENSMUSG00000001039 | 1.57 | 2.97 | 4.92E-10 | B9d1 | B9 protein domain 1 |
| ENSMUSG00000024909 | 1.57 | 2.97 | 1.31E-09 | Efemp2 | epidermal growth factor-containing fibulin-like extracellular matrix protein 2 |
| ENSMUSG00000040296 | 1.57 | 2.97 | 3.29E-25 | Ddx58 | DEAD (Asp-Glu-Ala-Asp) box polypeptide 58 |
| ENSMUSG00000026739 | 1.57 | 2.96 | 1.15E-10 | Bmi1 | Bmi1 polycomb ring finger oncogene |
| ENSMUSG00000083179 | 1.57 | 2.96 | 6.14E-09 | NA | NA |
| ENSMUSG00000045930 | 1.57 | 2.96 | 6.46E-09 | Clec14a | C-type lectin domain family 14, member a |
| ENSMUSG00000019916 | 1.57 | 2.96 | 1.21E-13 | P4ha1 | procollagen-proline, 2-oxoglutarate 4-dioxygenase (proline 4-hydroxylase), alpha 1 polypeptide |
| ENSMUSG00000029924 | 1.57 | 2.96 | 1.65E-40 | Slc37a3 | solute carrier family 37 (glycerol-3-phosphate transporter), member 3 |
| ENSMUSG00000026110 | 1.57 | 2.96 | 8.01E-15 | Mgat4a | mannoside acetylglucosaminyltransferase 4, isoenzyme A |
| ENSMUSG00000027002 | 1.57 | 2.96 | 1.43E-20 | Nckap1 | NCK-associated protein 1 |
| ENSMUSG00000055489 | 1.56 | 2.96 | 1.95E-07 | Ano5 | anoctamin 5 |
| ENSMUSG00000028885 | 1.56 | 2.96 | 1.59E-08 | Smpdl3b | sphingomyelin phosphodiesterase, acid-like 3B |
| ENSMUSG00000032850 | 1.56 | 2.96 | 4.40E-06 | Rnft2 | ring finger protein, transmembrane 2 |
| ENSMUSG00000031790 | 1.56 | 2.96 | 1.92E-06 | Mmp15 | matrix metallopeptidase 15 |
| ENSMUSG00000053012 | 1.56 | 2.95 | 1.34E-08 | Krcc1 | lysine-rich coiled-coil 1 |
| ENSMUSG00000062946 | 1.56 | 2.95 | 3.81E-06 | NA | NA |
| ENSMUSG00000030551 | 1.56 | 2.95 | 3.04E-07 | Nr2f2 | nuclear receptor subfamily 2, group F, member 2 |
| ENSMUSG00000081483 | 1.56 | 2.95 | 5.88E-03 | NA | NA |
| ENSMUSG00000073407 | 1.56 | 2.95 | 5.33E-03 | Gm6034 | predicted gene 6034 |
| ENSMUSG00000040605 | 1.56 | 2.95 | 1.50E-09 | Bace2 | beta-site APP-cleaving enzyme 2 |
| ENSMUSG00000032238 | 1.56 | 2.95 | 8.76E-13 | Rora | RAR-related orphan receptor alpha |
| ENSMUSG00000021359 | 1.56 | 2.95 | 1.01E-04 | Tfap2a | transcription factor AP-2, alpha |
| ENSMUSG00000041261 | 1.56 | 2.95 | 4.66E-03 | Car8 | carbonic anhydrase 8 |
| ENSMUSG00000028576 | 1.56 | 2.95 | 7.14E-07 | Ift74 | intraflagellar transport 74 |
| ENSMUSG00000037236 | 1.56 | 2.95 | 3.49E-11 | Matr3 | matrin 3 |
| ENSMUSG00000033793 | 1.56 | 2.95 | 1.97E-21 | Atp6v1h | ATPase, H+ transporting, lysosomal V1 subunit H |
| ENSMUSG00000085092 | 1.56 | 2.94 | 4.28E-03 | NA | NA |
| ENSMUSG00000028173 | 1.56 | 2.94 | 1.84E-35 | Wls | wntless homolog (Drosophila) |
| ENSMUSG00000088315 | 1.56 | 2.94 | 7.52E-03 | NA | NA |
| ENSMUSG00000019894 | 1.56 | 2.94 | 4.26E-03 | Slc6a15 | solute carrier family 6 (neurotransmitter transporter), member 15 |
| ENSMUSG00000020312 | 1.55 | 2.94 | 6.64E-12 | Shc2 | SHC (Src homology 2 domain containing) transforming protein 2 |
| ENSMUSG00000084241 | 1.55 | 2.94 | 1.39E-07 | NA | NA |
| ENSMUSG00000035095 | 1.55 | 2.93 | 5.90E-06 | Fam167a | family with sequence similarity 167, member A |
| ENSMUSG00000073556 | 1.55 | 2.93 | 1.76E-11 | NA | NA |
| ENSMUSG00000069114 | 1.55 | 2.93 | 2.96E-06 | Zbtb10 | zinc finger and BTB domain containing 10 |
| ENSMUSG00000005220 | 1.55 | 2.93 | 3.79E-04 | Corin | corin |
| ENSMUSG00000089817 | 1.55 | 2.93 | 2.47E-04 | NA | NA |
| ENSMUSG00000030245 | 1.55 | 2.93 | 1.83E-13 | Golt1b | golgi transport 1 homolog B (S. cerevisiae) |
| ENSMUSG00000026308 | 1.55 | 2.93 | 3.98E-05 | Klhl30 | kelch-like 30 |
| ENSMUSG00000037712 | 1.55 | 2.92 | 2.59E-10 | Fermt2 | fermitin family homolog 2 (Drosophila) |
| ENSMUSG00000074218 | 1.55 | 2.92 | 3.02E-05 | Cox7a1 | cytochrome c oxidase subunit VIIa 1 |
| ENSMUSG00000007617 | 1.55 | 2.92 | 3.24E-09 | Homer1 | homer homolog 1 (Drosophila) |
| ENSMUSG00000022992 | 1.55 | 2.92 | 8.11E-18 | Kansl2 | KAT8 regulatory NSL complex subunit 2 |
| ENSMUSG00000009545 | 1.55 | 2.92 | 4.71E-05 | Kcnq1 | potassium voltage-gated channel, subfamily Q, member 1 |
| ENSMUSG00000038600 | 1.55 | 2.92 | 2.48E-03 | Atp6v0a4 | ATPase, H+ transporting, lysosomal V0 subunit A4 |
| ENSMUSG00000038146 | 1.55 | 2.92 | 2.72E-07 | Notch3 | notch 3 |
| ENSMUSG00000034620 | 1.55 | 2.92 | 7.95E-17 | Tmem5 | transmembrane protein 5 |
| ENSMUSG00000026678 | 1.54 | 2.92 | 6.20E-15 | Rgs5 | regulator of G-protein signaling 5 |
| ENSMUSG00000025289 | 1.54 | 2.92 | 8.94E-22 | Prdx4 | peroxiredoxin 4 |
| ENSMUSG00000073889 | 1.54 | 2.91 | 5.95E-11 | Il11ra1 | interleukin 11 receptor, alpha chain 1 |
| ENSMUSG00000028664 | 1.54 | 2.91 | 1.03E-09 | Ephb2 | Eph receptor B2 |
| ENSMUSG00000040624 | 1.54 | 2.91 | 1.63E-10 | Plekhg1 | pleckstrin homology domain containing, family G (with RhoGef domain) member 1 |
| ENSMUSG00000066279 | 1.54 | 2.91 | 2.52E-04 | Chrna10 | cholinergic receptor, nicotinic, alpha polypeptide 10 |
| ENSMUSG00000021482 | 1.54 | 2.91 | 5.51E-16 | Aaed1 | AhpC/TSA antioxidant enzyme domain containing 1 |
| ENSMUSG00000050199 | 1.54 | 2.91 | 1.28E-11 | Lgr4 | leucine-rich repeat-containing G protein-coupled receptor 4 |
| ENSMUSG00000054588 | 1.54 | 2.91 | 5.24E-06 | Gbp10 | guanylate-binding protein 10 |
| ENSMUSG00000025979 | 1.54 | 2.91 | 1.09E-13 | Mob4 | MOB family member 4, phocein |
| ENSMUSG00000057093 | 1.54 | 2.91 | 6.34E-04 | C030039L03Rik | RIKEN cDNA C030039L03 gene |
| ENSMUSG00000017667 | 1.54 | 2.90 | 3.18E-10 | Zfp334 | zinc finger protein 334 |
| ENSMUSG00000072235 | 1.54 | 2.90 | 3.21E-11 | Tuba1a | tubulin, alpha 1A |
| ENSMUSG00000018604 | 1.54 | 2.90 | 3.27E-07 | Tbx3 | T-box 3 |
| ENSMUSG00000032495 | 1.54 | 2.90 | 3.85E-05 | Lrrc2 | leucine rich repeat containing 2 |
| ENSMUSG00000044636 | 1.54 | 2.90 | 4.44E-13 | Csrnp2 | cysteine-serine-rich nuclear protein 2 |
| ENSMUSG00000036478 | 1.54 | 2.90 | 4.47E-10 | Btg1 | B cell translocation gene 1, anti-proliferative |
| ENSMUSG00000052276 | 1.54 | 2.90 | 2.81E-06 | Ostn | osteocrin |
| ENSMUSG00000044231 | 1.54 | 2.90 | 1.22E-03 | Nhlrc1 | NHL repeat containing 1 |
| ENSMUSG00000060989 | 1.54 | 2.90 | 1.20E-07 | NA | NA |
| ENSMUSG00000061778 | 1.54 | 2.90 | 1.19E-09 | Mospd2 | motile sperm domain containing 2 |
| ENSMUSG00000049804 | 1.54 | 2.90 | 5.49E-19 | Armcx4 | armadillo repeat containing, X-linked 4 |
| ENSMUSG00000027937 | 1.54 | 2.90 | 4.06E-16 | Jtb | jumping translocation breakpoint |
| ENSMUSG00000002808 | 1.53 | 2.90 | 6.36E-21 | Epdr1 | ependymin related protein 1 (zebrafish) |
| ENSMUSG00000028082 | 1.53 | 2.90 | 1.65E-13 | Sh3d19 | SH3 domain protein D19 |
| ENSMUSG00000078104 | 1.53 | 2.89 | 1.47E-12 | NA | NA |
| ENSMUSG00000020032 | 1.53 | 2.89 | 2.48E-14 | Nuak1 | NUAK family, SNF1-like kinase, 1 |
| ENSMUSG00000052459 | 1.53 | 2.89 | 1.66E-23 | Atp6v1a | ATPase, H+ transporting, lysosomal V1 subunit A |
| ENSMUSG00000025040 | 1.53 | 2.89 | 1.17E-10 | Fundc1 | FUN14 domain containing 1 |
| ENSMUSG00000021552 | 1.53 | 2.89 | 1.55E-06 | Gkap1 | G kinase anchoring protein 1 |
| ENSMUSG00000031365 | 1.53 | 2.89 | 1.13E-13 | Zfp275 | zinc finger protein 275 |
| ENSMUSG00000065365 | 1.53 | 2.89 | 4.44E-03 | NA | NA |
| ENSMUSG00000040663 | 1.53 | 2.89 | 3.13E-08 | NA | NA |
| ENSMUSG00000026223 | 1.53 | 2.89 | 2.33E-10 | Itm2c | integral membrane protein 2C |
| ENSMUSG00000028879 | 1.53 | 2.89 | 5.66E-18 | Stx12 | syntaxin 12 |
| ENSMUSG00000033256 | 1.53 | 2.89 | 2.83E-13 | Shf | Src homology 2 domain containing F |
| ENSMUSG00000055210 | 1.53 | 2.89 | 8.94E-06 | Foxd2 | forkhead box D2 |
| ENSMUSG00000023186 | 1.53 | 2.89 | 2.13E-19 | Vwa5a | von Willebrand factor A domain containing 5A |
| ENSMUSG00000032307 | 1.53 | 2.88 | 2.85E-09 | Ube2q2 | ubiquitin-conjugating enzyme E2Q (putative) 2 |
| ENSMUSG00000053819 | 1.53 | 2.88 | 1.22E-15 | Camk2d | calcium/calmodulin-dependent protein kinase II, delta |
| ENSMUSG00000043300 | 1.53 | 2.88 | 5.67E-09 | B3galnt1 | UDP-GalNAc:betaGlcNAc beta 1,3-galactosaminyltransferase, polypeptide 1 |
| ENSMUSG00000025986 | 1.53 | 2.88 | 1.34E-13 | Slc39a10 | solute carrier family 39 (zinc transporter), member 10 |
| ENSMUSG00000019132 | 1.53 | 2.88 | 1.74E-17 | BC005537 | cDNA sequence BC005537 |
| ENSMUSG00000037455 | 1.53 | 2.88 | 9.19E-16 | Slc18b1 | solute carrier family 18, subfamily B, member 1 |
| ENSMUSG00000025212 | 1.53 | 2.88 | 3.60E-18 | Sfxn3 | sideroflexin 3 |
| ENSMUSG00000080935 | 1.52 | 2.88 | 9.98E-07 | NA | NA |
| ENSMUSG00000058927 | 1.52 | 2.87 | 1.40E-08 | NA | NA |
| ENSMUSG00000020620 | 1.52 | 2.87 | 7.39E-05 | Abca8b | ATP-binding cassette, sub-family A (ABC1), member 8b |
| ENSMUSG00000031851 | 1.52 | 2.87 | 4.26E-14 | Ntpcr | nucleoside-triphosphatase, cancer-related |
| ENSMUSG00000028641 | 1.52 | 2.87 | 1.72E-11 | Lepre1 | leprecan 1 |
| ENSMUSG00000025326 | 1.52 | 2.87 | 4.83E-10 | Ube3a | ubiquitin protein ligase E3A |
| ENSMUSG00000049775 | 1.52 | 2.87 | 2.22E-05 | Tmsb4x | thymosin, beta 4, X chromosome |
| ENSMUSG00000065087 | 1.52 | 2.87 | 4.77E-04 | Snord22 | small nucleolar RNA, C/D box 22 |
| ENSMUSG00000022800 | 1.52 | 2.87 | 5.14E-12 | Fyttd1 | forty-two-three domain containing 1 |
| ENSMUSG00000015968 | 1.52 | 2.87 | 5.14E-07 | Cacna1d | calcium channel, voltage-dependent, L type, alpha 1D subunit |
| ENSMUSG00000041084 | 1.52 | 2.87 | 8.86E-10 | Ostc | oligosaccharyltransferase complex subunit |
| ENSMUSG00000020363 | 1.52 | 2.87 | 1.07E-03 | Gfpt2 | glutamine fructose-6-phosphate transaminase 2 |
| ENSMUSG00000041552 | 1.52 | 2.87 | 4.47E-03 | Ptchd1 | patched domain containing 1 |
| ENSMUSG00000041594 | 1.52 | 2.86 | 7.11E-10 | Tmtc4 | transmembrane and tetratricopeptide repeat containing 4 |
| ENSMUSG00000044147 | 1.52 | 2.86 | 2.75E-06 | Arf6 | ADP-ribosylation factor 6 |
| ENSMUSG00000048782 | 1.52 | 2.86 | 2.53E-06 | Insc | inscuteable homolog (Drosophila) |
| ENSMUSG00000032420 | 1.52 | 2.86 | 1.53E-08 | Nt5e | 5' nucleotidase, ecto |
| ENSMUSG00000062082 | 1.52 | 2.86 | 2.09E-04 | Cd200r4 | CD200 receptor 4 |
| ENSMUSG00000049521 | 1.51 | 2.86 | 1.70E-08 | Cdc42ep1 | CDC42 effector protein (Rho GTPase binding) 1 |
| ENSMUSG00000055771 | 1.51 | 2.86 | 1.68E-05 | NA | NA |
| ENSMUSG00000067787 | 1.51 | 2.85 | 1.82E-14 | Blcap | bladder cancer associated protein homolog (human) |
| ENSMUSG00000050350 | 1.51 | 2.85 | 2.53E-06 | Gpr18 | G protein-coupled receptor 18 |
| ENSMUSG00000029469 | 1.51 | 2.85 | 2.49E-06 | Ift81 | intraflagellar transport 81 |
| ENSMUSG00000042233 | 1.51 | 2.85 | 6.81E-04 | 2010015L04Rik | RIKEN cDNA 2010015L04 gene |
| ENSMUSG00000044033 | 1.51 | 2.85 | 2.60E-04 | Ccdc141 | coiled-coil domain containing 141 |
| ENSMUSG00000031963 | 1.51 | 2.85 | 2.14E-09 | Bmper | BMP-binding endothelial regulator |
| ENSMUSG00000039081 | 1.51 | 2.85 | 2.96E-05 | Zfp503 | zinc finger protein 503 |
| ENSMUSG00000047878 | 1.51 | 2.85 | 1.94E-06 | A4galt | alpha 1,4-galactosyltransferase |
| ENSMUSG00000035357 | 1.51 | 2.85 | 5.27E-08 | Pdzrn3 | PDZ domain containing RING finger 3 |
| ENSMUSG00000031119 | 1.51 | 2.85 | 6.56E-22 | Gpc4 | glypican 4 |
| ENSMUSG00000056590 | 1.51 | 2.85 | 4.42E-06 | NA | NA |
| ENSMUSG00000063296 | 1.51 | 2.85 | 1.78E-05 | Tmem117 | transmembrane protein 117 |
| ENSMUSG00000079625 | 1.51 | 2.84 | 4.09E-07 | Tm4sf19 | transmembrane 4 L six family member 19 |
| ENSMUSG00000021874 | 1.51 | 2.84 | 3.99E-03 | 4933413J09Rik | RIKEN cDNA 4933413J09 gene |
| ENSMUSG00000089526 | 1.51 | 2.84 | 1.35E-03 | NA | NA |
| ENSMUSG00000040151 | 1.51 | 2.84 | 6.43E-22 | Hs2st1 | heparan sulfate 2-O-sulfotransferase 1 |
| ENSMUSG00000071286 | 1.51 | 2.84 | 2.23E-09 | NA | NA |
| ENSMUSG00000068762 | 1.51 | 2.84 | 8.59E-03 | Gstm6 | glutathione S-transferase, mu 6 |
| ENSMUSG00000031438 | 1.51 | 2.84 | 1.11E-05 | Rnf128 | ring finger protein 128 |
| ENSMUSG00000007783 | 1.51 | 2.84 | 2.43E-11 | Cpt1c | carnitine palmitoyltransferase 1c |
| ENSMUSG00000033083 | 1.51 | 2.84 | 1.42E-12 | Tbc1d4 | TBC1 domain family, member 4 |
| ENSMUSG00000033740 | 1.51 | 2.84 | 1.29E-03 | St18 | suppression of tumorigenicity 18 |
| ENSMUSG00000029276 | 1.51 | 2.84 | 1.71E-05 | Glmn | glomulin, FKBP associated protein |
| ENSMUSG00000010461 | 1.51 | 2.84 | 5.65E-06 | Eya4 | eyes absent 4 homolog (Drosophila) |
| ENSMUSG00000026875 | 1.50 | 2.84 | 7.94E-09 | Traf1 | TNF receptor-associated factor 1 |
| ENSMUSG00000046807 | 1.50 | 2.84 | 7.93E-07 | Fam211b | family with sequence similarity 211, member B |
| ENSMUSG00000035245 | 1.50 | 2.83 | 1.90E-20 | Eogt | EGF domain-specific O-linked N-acetylglucosamine (GlcNAc) transferase |
| ENSMUSG00000055653 | 1.50 | 2.83 | 4.37E-05 | Gpc3 | glypican 3 |
| ENSMUSG00000084950 | 1.50 | 2.83 | 2.74E-03 | Gm5577 | predicted gene 5577 |
| ENSMUSG00000030345 | 1.50 | 2.83 | 1.18E-03 | Dyrk4 | dual-specificity tyrosine-(Y)-phosphorylation regulated kinase 4 |
| ENSMUSG00000037892 | 1.50 | 2.83 | 1.37E-09 | Pcdh18 | protocadherin 18 |
| ENSMUSG00000039328 | 1.50 | 2.83 | 1.78E-13 | Rnf122 | ring finger protein 122 |
| ENSMUSG00000013662 | 1.50 | 2.83 | 1.58E-15 | Atad1 | ATPase family, AAA domain containing 1 |
| ENSMUSG00000021109 | 1.50 | 2.83 | 4.28E-06 | Hif1a | hypoxia inducible factor 1, alpha subunit |
| ENSMUSG00000022947 | 1.50 | 2.83 | 1.04E-09 | Cbr3 | carbonyl reductase 3 |
| ENSMUSG00000018821 | 1.50 | 2.83 | 9.87E-11 | Avpi1 | arginine vasopressin-induced 1 |
| ENSMUSG00000017491 | 1.50 | 2.82 | 8.03E-06 | Rarb | retinoic acid receptor, beta |
| ENSMUSG00000090066 | 1.50 | 2.82 | 6.36E-04 | LOC102634333 | uncharacterized LOC102634333 |
| ENSMUSG00000079351 | 1.50 | 2.82 | 4.70E-03 | NA | NA |
| ENSMUSG00000017466 | 1.50 | 2.82 | 1.77E-10 | Timp2 | tissue inhibitor of metalloproteinase 2 |
| ENSMUSG00000035208 | 1.50 | 2.82 | 1.99E-07 | Slfn8 | schlafen 8 |
| ENSMUSG00000026321 | 1.50 | 2.82 | 2.71E-09 | Tnfrsf11a | tumor necrosis factor receptor superfamily, member 11a |
| ENSMUSG00000028556 | 1.49 | 2.82 | 1.34E-07 | Dock7 | dedicator of cytokinesis 7 |
| ENSMUSG00000021079 | 1.49 | 2.82 | 2.28E-07 | Timm9 | translocase of inner mitochondrial membrane 9 |
| ENSMUSG00000013089 | 1.49 | 2.82 | 1.04E-14 | Etv5 | ets variant gene 5 |
| ENSMUSG00000001627 | 1.49 | 2.82 | 2.43E-14 | Ifrd1 | interferon-related developmental regulator 1 |
| ENSMUSG00000004768 | 1.49 | 2.82 | 2.48E-14 | Rab23 | RAB23, member RAS oncogene family |
| ENSMUSG00000026104 | 1.49 | 2.81 | 1.35E-15 | Stat1 | signal transducer and activator of transcription 1 |
| ENSMUSG00000056313 | 1.49 | 2.81 | 8.68E-03 | 1810011O10Rik | RIKEN cDNA 1810011O10 gene |
| ENSMUSG00000071073 | 1.49 | 2.81 | 3.19E-03 | Lrrc73 | leucine rich repeat containing 73 |
| ENSMUSG00000037816 | 1.49 | 2.81 | 5.95E-12 | Fbxw17 | F-box and WD-40 domain protein 17 |
| ENSMUSG00000000094 | 1.49 | 2.81 | 4.97E-06 | Tbx4 | T-box 4 |
| ENSMUSG00000032431 | 1.49 | 2.81 | 2.57E-11 | Crtap | cartilage associated protein |
| ENSMUSG00000029238 | 1.49 | 2.81 | 3.53E-09 | Clock | circadian locomotor output cycles kaput |
| ENSMUSG00000019782 | 1.49 | 2.81 | 8.78E-13 | NA | NA |
| ENSMUSG00000001036 | 1.49 | 2.80 | 2.01E-19 | Epn2 | epsin 2 |
| ENSMUSG00000087443 | 1.49 | 2.80 | 4.53E-04 | NA | NA |
| ENSMUSG00000042826 | 1.49 | 2.80 | 8.40E-08 | Fgf11 | fibroblast growth factor 11 |
| ENSMUSG00000027805 | 1.49 | 2.80 | 4.85E-07 | Pfn2 | profilin 2 |
| ENSMUSG00000080845 | 1.49 | 2.80 | 7.67E-07 | NA | NA |
| ENSMUSG00000027006 | 1.48 | 2.80 | 5.66E-29 | Dnajc10 | DnaJ (Hsp40) homolog, subfamily C, member 10 |
| ENSMUSG00000060181 | 1.48 | 2.80 | 3.52E-17 | Slc35e3 | solute carrier family 35, member E3 |
| ENSMUSG00000031885 | 1.48 | 2.80 | 9.16E-13 | Cbfb | core binding factor beta |
| ENSMUSG00000074364 | 1.48 | 2.80 | 5.37E-19 | Ehd2 | EH-domain containing 2 |
| ENSMUSG00000073792 | 1.48 | 2.79 | 4.19E-11 | Alg6 | asparagine-linked glycosylation 6 (alpha-1,3,-glucosyltransferase) |
| ENSMUSG00000022257 | 1.48 | 2.79 | 1.58E-18 | Laptm4b | lysosomal-associated protein transmembrane 4B |
| ENSMUSG00000051343 | 1.48 | 2.79 | 3.29E-18 | Rab11fip5 | RAB11 family interacting protein 5 (class I) |
| ENSMUSG00000008734 | 1.48 | 2.79 | 5.33E-16 | Gprc5b | G protein-coupled receptor, family C, group 5, member B |
| ENSMUSG00000046318 | 1.48 | 2.79 | 4.85E-14 | Ccbe1 | collagen and calcium binding EGF domains 1 |
| ENSMUSG00000030796 | 1.48 | 2.79 | 2.35E-10 | Tead2 | TEA domain family member 2 |
| ENSMUSG00000061535 | 1.48 | 2.79 | 2.26E-03 | C1qtnf7 | C1q and tumor necrosis factor related protein 7 |
| ENSMUSG00000017677 | 1.48 | 2.79 | 1.06E-21 | Wsb1 | WD repeat and SOCS box-containing 1 |
| ENSMUSG00000030351 | 1.48 | 2.79 | 2.69E-10 | Tspan11 | tetraspanin 11 |
| ENSMUSG00000078920 | 1.48 | 2.79 | 1.17E-20 | Ifi47 | interferon gamma inducible protein 47 |
| ENSMUSG00000048520 | 1.48 | 2.79 | 5.39E-03 | Fbxl13 | F-box and leucine-rich repeat protein 13 |
| ENSMUSG00000042272 | 1.48 | 2.79 | 2.79E-19 | Sestd1 | SEC14 and spectrin domains 1 |
| ENSMUSG00000020707 | 1.48 | 2.79 | 2.94E-12 | Rnf135 | ring finger protein 135 |
| ENSMUSG00000036745 | 1.48 | 2.79 | 3.69E-08 | Ttll7 | tubulin tyrosine ligase-like family, member 7 |
| ENSMUSG00000034706 | 1.48 | 2.79 | 2.81E-04 | Dnaic2 | dynein, axonemal, intermediate chain 2 |
| ENSMUSG00000031480 | 1.48 | 2.79 | 1.06E-10 | Thsd1 | thrombospondin, type I, domain 1 |
| ENSMUSG00000034714 | 1.48 | 2.78 | 3.60E-07 | Ttyh2 | tweety homolog 2 (Drosophila) |
| ENSMUSG00000051043 | 1.48 | 2.78 | 6.11E-08 | Gprc5c | G protein-coupled receptor, family C, group 5, member C |
| ENSMUSG00000038022 | 1.48 | 2.78 | 1.35E-10 | Fam188b | family with sequence similarity 188, member B |
| ENSMUSG00000002732 | 1.48 | 2.78 | 1.80E-04 | Fkbp7 | FK506 binding protein 7 |
| ENSMUSG00000040596 | 1.48 | 2.78 | 5.81E-12 | Pogk | pogo transposable element with KRAB domain |
| ENSMUSG00000043496 | 1.48 | 2.78 | 1.01E-03 | Tril | TLR4 interactor with leucine-rich repeats |
| ENSMUSG00000042751 | 1.48 | 2.78 | 1.15E-06 | Nmnat2 | nicotinamide nucleotide adenylyltransferase 2 |
| ENSMUSG00000010067 | 1.48 | 2.78 | 2.76E-16 | Rassf1 | Ras association (RalGDS/AF-6) domain family member 1 |
| ENSMUSG00000040613 | 1.47 | 2.78 | 1.21E-17 | Apobec1 | apolipoprotein B mRNA editing enzyme, catalytic polypeptide 1 |
| ENSMUSG00000047843 | 1.47 | 2.78 | 1.21E-06 | Bri3 | brain protein I3 |
| ENSMUSG00000020116 | 1.47 | 2.78 | 7.43E-14 | Pno1 | partner of NOB1 homolog (S. cerevisiae) |
| ENSMUSG00000019831 | 1.47 | 2.78 | 2.64E-04 | Wasf1 | WAS protein family, member 1 |
| ENSMUSG00000027398 | 1.47 | 2.78 | 7.38E-06 | Il1b | interleukin 1 beta |
| ENSMUSG00000022146 | 1.47 | 2.77 | 1.22E-13 | Osmr | oncostatin M receptor |
| ENSMUSG00000003534 | 1.47 | 2.77 | 1.86E-06 | Ddr1 | discoidin domain receptor family, member 1 |
| ENSMUSG00000009731 | 1.47 | 2.77 | 9.15E-11 | Kcnd1 | potassium voltage-gated channel, Shal-related family, member 1 |
| ENSMUSG00000028780 | 1.47 | 2.77 | 9.42E-04 | Sema3c | sema domain, immunoglobulin domain (Ig), short basic domain, secreted, (semaphorin) 3C |
| ENSMUSG00000021575 | 1.47 | 2.77 | 6.03E-03 | Ahrr | aryl-hydrocarbon receptor repressor |
| ENSMUSG00000040488 | 1.47 | 2.77 | 1.80E-05 | Ltbp4 | latent transforming growth factor beta binding protein 4 |
| ENSMUSG00000030313 | 1.47 | 2.77 | 2.35E-15 | Dennd5b | DENN/MADD domain containing 5B |
| ENSMUSG00000040998 | 1.47 | 2.77 | 2.17E-10 | Npnt | nephronectin |
| ENSMUSG00000041245 | 1.47 | 2.77 | 1.99E-03 | Wnk3 | WNK lysine deficient protein kinase 3 |
| ENSMUSG00000068617 | 1.47 | 2.77 | 9.88E-03 | Efcab1 | EF hand calcium binding domain 1 |
| ENSMUSG00000052852 | 1.47 | 2.77 | 1.11E-07 | Reep1 | receptor accessory protein 1 |
| ENSMUSG00000027828 | 1.47 | 2.76 | 1.30E-27 | Ssr3 | signal sequence receptor, gamma |
| ENSMUSG00000021367 | 1.47 | 2.76 | 5.28E-05 | Edn1 | endothelin 1 |
| ENSMUSG00000003380 | 1.47 | 2.76 | 1.44E-07 | Rabac1 | Rab acceptor 1 (prenylated) |
| ENSMUSG00000022698 | 1.46 | 2.76 | 1.46E-13 | Naa50 | N(alpha)-acetyltransferase 50, NatE catalytic subunit |
| ENSMUSG00000031357 | 1.46 | 2.76 | 1.58E-11 | Syap1 | synapse associated protein 1 |
| ENSMUSG00000031284 | 1.46 | 2.76 | 5.54E-03 | Pak3 | p21 protein (Cdc42/Rac)-activated kinase 3 |
| ENSMUSG00000084911 | 1.46 | 2.76 | 5.91E-03 | NA | NA |
| ENSMUSG00000051379 | 1.46 | 2.75 | 1.50E-03 | Flrt3 | fibronectin leucine rich transmembrane protein 3 |
| ENSMUSG00000032374 | 1.46 | 2.75 | 2.52E-10 | Plod2 | procollagen lysine, 2-oxoglutarate 5-dioxygenase 2 |
| ENSMUSG00000044461 | 1.46 | 2.75 | 7.20E-06 | Shisa2 | shisa homolog 2 (Xenopus laevis) |
| ENSMUSG00000078815 | 1.46 | 2.75 | 4.48E-04 | NA | NA |
| ENSMUSG00000038522 | 1.46 | 2.75 | 1.23E-03 | AI317395 | expressed sequence AI317395 |
| ENSMUSG00000020521 | 1.46 | 2.75 | 7.59E-20 | Rnft1 | ring finger protein, transmembrane 1 |
| ENSMUSG00000042622 | 1.46 | 2.75 | 5.75E-05 | Maff | v-maf musculoaponeurotic fibrosarcoma oncogene family, protein F (avian) |
| ENSMUSG00000025484 | 1.46 | 2.75 | 2.47E-08 | Bet1l | blocked early in transport 1 homolog (S. cerevisiae)-like |
| ENSMUSG00000034765 | 1.46 | 2.75 | 2.73E-13 | Dusp5 | dual specificity phosphatase 5 |
| ENSMUSG00000041936 | 1.46 | 2.75 | 2.94E-10 | Agrn | agrin |
| ENSMUSG00000026502 | 1.46 | 2.75 | 3.41E-20 | Desi2 | desumoylating isopeptidase 2 |
| ENSMUSG00000001134 | 1.46 | 2.75 | 1.69E-04 | Uxt | ubiquitously expressed transcript |
| ENSMUSG00000001642 | 1.46 | 2.75 | 4.30E-04 | Akr1b3 | aldo-keto reductase family 1, member B3 (aldose reductase) |
| ENSMUSG00000035762 | 1.46 | 2.75 | 1.27E-14 | Tmem161b | transmembrane protein 161B |
| ENSMUSG00000047434 | 1.46 | 2.75 | 3.21E-15 | Xxylt1 | xyloside xylosyltransferase 1 |
| ENSMUSG00000069920 | 1.46 | 2.74 | 3.43E-08 | B3gnt9 | UDP-GlcNAc:betaGal beta-1,3-N-acetylglucosaminyltransferase 9 |
| ENSMUSG00000029009 | 1.46 | 2.74 | 1.53E-12 | Mthfr | 5,10-methylenetetrahydrofolate reductase |
| ENSMUSG00000032253 | 1.46 | 2.74 | 1.78E-06 | Phip | pleckstrin homology domain interacting protein |
| ENSMUSG00000054099 | 1.46 | 2.74 | 6.01E-06 | Slc25a40 | solute carrier family 25, member 40 |
| ENSMUSG00000073368 | 1.45 | 2.74 | 2.00E-04 | NA | NA |
| ENSMUSG00000031939 | 1.45 | 2.74 | 7.25E-10 | Taf1d | TATA box binding protein (Tbp)-associated factor, RNA polymerase I, D |
| ENSMUSG00000050530 | 1.45 | 2.74 | 1.16E-16 | Fam171a1 | family with sequence similarity 171, member A1 |
| ENSMUSG00000020728 | 1.45 | 2.73 | 7.63E-04 | Cep112 | centrosomal protein 112 |
| ENSMUSG00000035314 | 1.45 | 2.73 | 6.14E-11 | Gdpd5 | glycerophosphodiester phosphodiesterase domain containing 5 |
| ENSMUSG00000008575 | 1.45 | 2.73 | 1.36E-14 | Nfib | nuclear factor I/B |
| ENSMUSG00000021211 | 1.45 | 2.73 | 1.16E-03 | Akr1c12 | aldo-keto reductase family 1, member C12 |
| ENSMUSG00000002032 | 1.45 | 2.73 | 6.19E-05 | Tmem25 | transmembrane protein 25 |
| ENSMUSG00000026566 | 1.45 | 2.73 | 9.85E-15 | Mpzl1 | myelin protein zero-like 1 |
| ENSMUSG00000034111 | 1.45 | 2.73 | 3.42E-09 | Tmed8 | transmembrane emp24 domain containing 8 |
| ENSMUSG00000039765 | 1.45 | 2.73 | 1.23E-09 | Cc2d2a | coiled-coil and C2 domain containing 2A |
| ENSMUSG00000069755 | 1.45 | 2.73 | 1.63E-04 | NA | NA |
| ENSMUSG00000044629 | 1.45 | 2.73 | 2.77E-09 | Cnrip1 | cannabinoid receptor interacting protein 1 |
| ENSMUSG00000039191 | 1.45 | 2.73 | 5.38E-11 | Rbpj | recombination signal binding protein for immunoglobulin kappa J region |
| ENSMUSG00000043424 | 1.45 | 2.73 | 1.77E-10 | Eif3j2 | eukaryotic translation initiation factor 3, subunit J2 |
| ENSMUSG00000041343 | 1.45 | 2.72 | 4.10E-05 | Ankrd42 | ankyrin repeat domain 42 |
| ENSMUSG00000045312 | 1.44 | 2.72 | 1.24E-13 | Lhfpl2 | lipoma HMGIC fusion partner-like 2 |
| ENSMUSG00000028300 | 1.44 | 2.72 | 2.37E-26 | 3110043O21Rik | RIKEN cDNA 3110043O21 gene |
| ENSMUSG00000021062 | 1.44 | 2.72 | 2.41E-08 | Rab15 | RAB15, member RAS oncogene family |
| ENSMUSG00000040751 | 1.44 | 2.72 | 8.82E-10 | Lat2 | linker for activation of T cells family, member 2 |
| ENSMUSG00000036904 | 1.44 | 2.72 | 1.51E-03 | Fzd8 | frizzled homolog 8 (Drosophila) |
| ENSMUSG00000021464 | 1.44 | 2.72 | 9.21E-06 | Ror2 | receptor tyrosine kinase-like orphan receptor 2 |
| ENSMUSG00000063605 | 1.44 | 2.72 | 9.88E-18 | Ccdc102a | coiled-coil domain containing 102A |
| ENSMUSG00000021028 | 1.44 | 2.72 | 1.60E-06 | Mbip | MAP3K12 binding inhibitory protein 1 |
| ENSMUSG00000078525 | 1.44 | 2.72 | 2.12E-12 | NA | NA |
| ENSMUSG00000027122 | 1.44 | 2.71 | 1.13E-18 | Arl14ep | ADP-ribosylation factor-like 14 effector protein |
| ENSMUSG00000028370 | 1.44 | 2.71 | 5.50E-08 | Pappa | pregnancy-associated plasma protein A |
| ENSMUSG00000034163 | 1.44 | 2.71 | 1.44E-07 | Zfc3h1 | zinc finger, C3H1-type containing |
| ENSMUSG00000032006 | 1.44 | 2.71 | 2.01E-07 | Pdgfd | platelet-derived growth factor, D polypeptide |
| ENSMUSG00000028246 | 1.44 | 2.71 | 3.31E-05 | Faxc | failed axon connections homolog (Drosophila) |
| ENSMUSG00000063543 | 1.44 | 2.71 | 3.03E-08 | NA | NA |
| ENSMUSG00000036019 | 1.44 | 2.71 | 9.16E-07 | Tmtc2 | transmembrane and tetratricopeptide repeat containing 2 |
| ENSMUSG00000024124 | 1.44 | 2.71 | 4.87E-03 | Prss30 | protease, serine 30 |
| ENSMUSG00000040459 | 1.44 | 2.71 | 1.17E-12 | Arglu1 | arginine and glutamate rich 1 |
| ENSMUSG00000006476 | 1.44 | 2.71 | 3.09E-10 | Nsmf | NMDA receptor synaptonuclear signaling and neuronal migration factor |
| ENSMUSG00000051723 | 1.44 | 2.71 | 8.96E-03 | NA | NA |
| ENSMUSG00000020100 | 1.44 | 2.71 | 5.60E-12 | Slc29a3 | solute carrier family 29 (nucleoside transporters), member 3 |
| ENSMUSG00000034557 | 1.44 | 2.71 | 9.62E-10 | Zfyve9 | zinc finger, FYVE domain containing 9 |
| ENSMUSG00000026730 | 1.44 | 2.71 | 1.29E-10 | Pter | phosphotriesterase related |
| ENSMUSG00000032042 | 1.44 | 2.71 | 4.84E-21 | Srpr | signal recognition particle receptor ('docking protein') |
| ENSMUSG00000039234 | 1.44 | 2.71 | 2.01E-11 | Sec24d | Sec24 related gene family, member D (S. cerevisiae) |
| ENSMUSG00000023150 | 1.44 | 2.71 | 5.62E-19 | Ivns1abp | influenza virus NS1A binding protein |
| ENSMUSG00000053870 | 1.44 | 2.70 | 4.81E-09 | Fpgt | fucose-1-phosphate guanylyltransferase |
| ENSMUSG00000031382 | 1.43 | 2.70 | 5.01E-04 | Asb11 | ankyrin repeat and SOCS box-containing 11 |
| ENSMUSG00000001864 | 1.43 | 2.70 | 1.48E-03 | Aif1l | allograft inflammatory factor 1-like |
| ENSMUSG00000003721 | 1.43 | 2.70 | 1.67E-28 | Insig2 | insulin induced gene 2 |
| ENSMUSG00000035062 | 1.43 | 2.70 | 3.83E-05 | Zc4h2 | zinc finger, C4H2 domain containing |
| ENSMUSG00000069833 | 1.43 | 2.70 | 1.17E-13 | Ahnak | AHNAK nucleoprotein (desmoyokin) |
| ENSMUSG00000079484 | 1.43 | 2.70 | 2.08E-04 | Phyhd1 | phytanoyl-CoA dioxygenase domain containing 1 |
| ENSMUSG00000026890 | 1.43 | 2.70 | 2.51E-05 | Lhx6 | LIM homeobox protein 6 |
| ENSMUSG00000053600 | 1.43 | 2.70 | 5.06E-07 | Zfp472 | zinc finger protein 472 |
| ENSMUSG00000050271 | 1.43 | 2.70 | 4.32E-10 | D8Ertd82e | DNA segment, Chr 8, ERATO Doi 82, expressed |
| ENSMUSG00000066637 | 1.43 | 2.70 | 1.49E-09 | Ttc32 | tetratricopeptide repeat domain 32 |
| ENSMUSG00000027932 | 1.43 | 2.70 | 1.38E-07 | Slc27a3 | solute carrier family 27 (fatty acid transporter), member 3 |
| ENSMUSG00000080836 | 1.43 | 2.70 | 2.45E-05 | Gm6444 | predicted gene 6444 |
| ENSMUSG00000022203 | 1.43 | 2.69 | 2.24E-10 | Efs | embryonal Fyn-associated substrate |
| ENSMUSG00000032009 | 1.43 | 2.69 | 3.49E-07 | Sesn3 | sestrin 3 |
| ENSMUSG00000028399 | 1.43 | 2.69 | 4.45E-14 | Ptprd | protein tyrosine phosphatase, receptor type, D |
| ENSMUSG00000090084 | 1.43 | 2.69 | 3.23E-09 | Srpx | sushi-repeat-containing protein |
| ENSMUSG00000016252 | 1.43 | 2.69 | 3.02E-09 | Atp5e | ATP synthase, H+ transporting, mitochondrial F1 complex, epsilon subunit |
| ENSMUSG00000043336 | 1.43 | 2.69 | 1.47E-10 | Filip1l | filamin A interacting protein 1-like |
| ENSMUSG00000023025 | 1.43 | 2.69 | 7.55E-08 | Larp4 | La ribonucleoprotein domain family, member 4 |
| ENSMUSG00000020260 | 1.43 | 2.69 | 3.92E-13 | Pofut2 | protein O-fucosyltransferase 2 |
| ENSMUSG00000030203 | 1.43 | 2.69 | 4.45E-22 | Dusp16 | dual specificity phosphatase 16 |
| ENSMUSG00000078861 | 1.43 | 2.69 | 3.82E-05 | Zfp931 | zinc finger protein 931 |
| ENSMUSG00000035041 | 1.42 | 2.69 | 3.79E-05 | Creb3l3 | cAMP responsive element binding protein 3-like 3 |
| ENSMUSG00000057895 | 1.42 | 2.68 | 2.93E-05 | Zfp105 | zinc finger protein 105 |
| ENSMUSG00000037239 | 1.42 | 2.68 | 8.83E-11 | Spred3 | sprouty-related, EVH1 domain containing 3 |
| ENSMUSG00000039105 | 1.42 | 2.68 | 1.71E-06 | Atp6v1g1 | ATPase, H+ transporting, lysosomal V1 subunit G1 |
| ENSMUSG00000004939 | 1.42 | 2.68 | 2.53E-03 | Nmrk2 | nicotinamide riboside kinase 2 |
| ENSMUSG00000051022 | 1.42 | 2.68 | 3.06E-05 | Hs3st1 | heparan sulfate (glucosamine) 3-O-sulfotransferase 1 |
| ENSMUSG00000052656 | 1.42 | 2.68 | 6.86E-14 | Rnf103 | ring finger protein 103 |
| ENSMUSG00000040562 | 1.42 | 2.68 | 4.58E-05 | Gstm2 | glutathione S-transferase, mu 2 |
| ENSMUSG00000030745 | 1.42 | 2.68 | 9.00E-08 | Il21r | interleukin 21 receptor |
| ENSMUSG00000003476 | 1.42 | 2.67 | 3.64E-05 | Crhr2 | corticotropin releasing hormone receptor 2 |
| ENSMUSG00000022548 | 1.42 | 2.67 | 3.20E-04 | Apod | apolipoprotein D |
| ENSMUSG00000055228 | 1.42 | 2.67 | 4.28E-07 | NA | NA |
| ENSMUSG00000025555 | 1.42 | 2.67 | 2.38E-19 | Farp1 | FERM, RhoGEF (Arhgef) and pleckstrin domain protein 1 (chondrocyte-derived) |
| ENSMUSG00000087022 | 1.42 | 2.67 | 1.12E-04 | 9130024F11Rik | RIKEN cDNA 9130024F11 gene |
| ENSMUSG00000075389 | 1.42 | 2.67 | 1.37E-06 | NA | NA |
| ENSMUSG00000073617 | 1.42 | 2.67 | 6.69E-03 | NA | NA |
| ENSMUSG00000052395 | 1.42 | 2.67 | 2.16E-09 | Rft1 | RFT1 homolog (S. cerevisiae) |
| ENSMUSG00000063179 | 1.42 | 2.67 | 2.02E-12 | Pstk | phosphoseryl-tRNA kinase |
| ENSMUSG00000047592 | 1.42 | 2.67 | 2.02E-05 | Nxpe5 | neurexophilin and PC-esterase domain family, member 5 |
| ENSMUSG00000031990 | 1.42 | 2.67 | 1.63E-20 | Jam3 | junction adhesion molecule 3 |
| ENSMUSG00000053646 | 1.42 | 2.67 | 5.91E-07 | Plxnb1 | plexin B1 |
| ENSMUSG00000007682 | 1.42 | 2.67 | 1.99E-09 | Dio2 | deiodinase, iodothyronine, type II |
| ENSMUSG00000079724 | 1.42 | 2.67 | 5.36E-04 | NA | NA |
| ENSMUSG00000039578 | 1.42 | 2.67 | 4.93E-05 | Ccser1 | coiled-coil serine rich 1 |
| ENSMUSG00000074480 | 1.42 | 2.67 | 1.45E-10 | Mex3a | mex3 homolog A (C. elegans) |
| ENSMUSG00000029207 | 1.41 | 2.66 | 2.14E-13 | Apbb2 | amyloid beta (A4) precursor protein-binding, family B, member 2 |
| ENSMUSG00000031508 | 1.41 | 2.66 | 1.78E-13 | Ankrd10 | ankyrin repeat domain 10 |
| ENSMUSG00000028367 | 1.41 | 2.66 | 4.21E-12 | Txn1 | thioredoxin 1 |
| ENSMUSG00000063558 | 1.41 | 2.66 | 1.60E-09 | Aox1 | aldehyde oxidase 1 |
| ENSMUSG00000041684 | 1.41 | 2.66 | 1.94E-17 | Bivm | basic, immunoglobulin-like variable motif containing |
| ENSMUSG00000024990 | 1.41 | 2.66 | 1.95E-05 | Rbp4 | retinol binding protein 4, plasma |
| ENSMUSG00000086623 | 1.41 | 2.66 | 4.24E-03 | NA | NA |
| ENSMUSG00000027677 | 1.41 | 2.66 | 1.17E-12 | Ttc14 | tetratricopeptide repeat domain 14 |
| ENSMUSG00000078285 | 1.41 | 2.66 | 2.92E-03 | NA | NA |
| ENSMUSG00000038260 | 1.41 | 2.66 | 1.50E-09 | Trpm4 | transient receptor potential cation channel, subfamily M, member 4 |
| ENSMUSG00000025964 | 1.41 | 2.66 | 8.17E-05 | Adam23 | a disintegrin and metallopeptidase domain 23 |
| ENSMUSG00000021706 | 1.41 | 2.65 | 1.06E-12 | Zfyve16 | zinc finger, FYVE domain containing 16 |
| ENSMUSG00000063427 | 1.41 | 2.65 | 5.25E-05 | NA | NA |
| ENSMUSG00000030103 | 1.41 | 2.65 | 1.26E-06 | Bhlhe40 | basic helix-loop-helix family, member e40 |
| ENSMUSG00000014353 | 1.41 | 2.65 | 1.53E-14 | Tmem87b | transmembrane protein 87B |
| ENSMUSG00000026761 | 1.41 | 2.65 | 9.20E-11 | Orc4 | origin recognition complex, subunit 4 |
| ENSMUSG00000023992 | 1.41 | 2.65 | 2.64E-04 | Trem2 | triggering receptor expressed on myeloid cells 2 |
| ENSMUSG00000031841 | 1.41 | 2.65 | 7.26E-09 | Cdh13 | cadherin 13 |
| ENSMUSG00000027954 | 1.41 | 2.65 | 2.91E-07 | Efna1 | ephrin A1 |
| ENSMUSG00000005501 | 1.41 | 2.65 | 1.19E-14 | Usp40 | ubiquitin specific peptidase 40 |
| ENSMUSG00000025939 | 1.41 | 2.65 | 4.05E-13 | Ube2w | ubiquitin-conjugating enzyme E2W (putative) |
| ENSMUSG00000035121 | 1.41 | 2.65 | 2.54E-03 | Neil2 | nei like 2 (E. coli) |
| ENSMUSG00000020614 | 1.41 | 2.65 | 2.07E-11 | Fam20a | family with sequence similarity 20, member A |
| ENSMUSG00000026785 | 1.41 | 2.65 | 1.46E-15 | Pkn3 | protein kinase N3 |
| ENSMUSG00000033964 | 1.41 | 2.65 | 4.93E-08 | Zbtb41 | zinc finger and BTB domain containing 41 homolog |
| ENSMUSG00000020794 | 1.40 | 2.65 | 1.24E-15 | Ube2g1 | ubiquitin-conjugating enzyme E2G 1 |
| ENSMUSG00000075419 | 1.40 | 2.65 | 1.43E-08 | Dolk | dolichol kinase |
| ENSMUSG00000006378 | 1.40 | 2.65 | 1.04E-04 | NA | NA |
| ENSMUSG00000060284 | 1.40 | 2.64 | 2.71E-06 | Sp7 | Sp7 transcription factor 7 |
| ENSMUSG00000021536 | 1.40 | 2.64 | 1.97E-12 | Adcy2 | adenylate cyclase 2 |
| ENSMUSG00000046351 | 1.40 | 2.64 | 9.54E-08 | Zfp322a | zinc finger protein 322A |
| ENSMUSG00000030870 | 1.40 | 2.64 | 2.19E-17 | Ubfd1 | ubiquitin family domain containing 1 |
| ENSMUSG00000058145 | 1.40 | 2.64 | 2.63E-04 | Adamts17 | a disintegrin-like and metallopeptidase (reprolysin type) with thrombospondin type 1 motif, 17 |
| ENSMUSG00000034473 | 1.40 | 2.64 | 1.92E-07 | Sec22a | SEC22 vesicle trafficking protein homolog A (S. cerevisiae) |
| ENSMUSG00000006435 | 1.40 | 2.64 | 1.65E-05 | Neurl1a | neuralized homolog 1A (Drosophila) |
| ENSMUSG00000025921 | 1.40 | 2.64 | 1.25E-16 | Rdh10 | retinol dehydrogenase 10 (all-trans) |
| ENSMUSG00000033327 | 1.40 | 2.64 | 1.45E-03 | Tnxb | tenascin XB |
| ENSMUSG00000052921 | 1.40 | 2.64 | 1.17E-11 | Arhgef15 | Rho guanine nucleotide exchange factor (GEF) 15 |
| ENSMUSG00000025880 | 1.40 | 2.64 | 6.63E-08 | Smad7 | SMAD family member 7 |
| ENSMUSG00000068250 | 1.40 | 2.64 | 1.56E-09 | Amn1 | antagonist of mitotic exit network 1 |
| ENSMUSG00000051285 | 1.40 | 2.64 | 4.46E-09 | Pcmtd1 | protein-L-isoaspartate (D-aspartate) O-methyltransferase domain containing 1 |
| ENSMUSG00000018442 | 1.40 | 2.63 | 5.68E-14 | Derl2 | Der1-like domain family, member 2 |
| ENSMUSG00000038765 | 1.40 | 2.63 | 5.17E-04 | Lmx1b | LIM homeobox transcription factor 1 beta |
| ENSMUSG00000022884 | 1.40 | 2.63 | 5.18E-16 | Eif4a2 | eukaryotic translation initiation factor 4A2 |
| ENSMUSG00000030748 | 1.40 | 2.63 | 5.34E-08 | Il4ra | interleukin 4 receptor, alpha |
| ENSMUSG00000066705 | 1.40 | 2.63 | 2.96E-08 | Fxyd6 | FXYD domain-containing ion transport regulator 6 |
| ENSMUSG00000024987 | 1.39 | 2.63 | 3.67E-03 | Cyp26a1 | cytochrome P450, family 26, subfamily a, polypeptide 1 |
| ENSMUSG00000031149 | 1.39 | 2.63 | 8.20E-08 | Praf2 | PRA1 domain family 2 |
| ENSMUSG00000038349 | 1.39 | 2.63 | 1.34E-06 | Plcl1 | phospholipase C-like 1 |
| ENSMUSG00000051671 | 1.39 | 2.63 | 4.01E-03 | Coa6 | cytochrome c oxidase assembly factor 6 |
| ENSMUSG00000045519 | 1.39 | 2.63 | 6.96E-07 | Zfp560 | zinc finger protein 560 |
| ENSMUSG00000040563 | 1.39 | 2.62 | 3.18E-05 | BC018242 | cDNA sequence BC018242 |
| ENSMUSG00000033684 | 1.39 | 2.62 | 2.30E-14 | Qsox1 | quiescin Q6 sulfhydryl oxidase 1 |
| ENSMUSG00000022305 | 1.39 | 2.62 | 3.46E-08 | Lrp12 | low density lipoprotein-related protein 12 |
| ENSMUSG00000020458 | 1.39 | 2.62 | 2.72E-18 | Rtn4 | reticulon 4 |
| ENSMUSG00000090210 | 1.39 | 2.62 | 2.24E-12 | Itga10 | integrin, alpha 10 |
| ENSMUSG00000053137 | 1.39 | 2.62 | 1.52E-08 | Mapk11 | mitogen-activated protein kinase 11 |
| ENSMUSG00000046480 | 1.39 | 2.62 | 5.98E-05 | Scn4b | sodium channel, type IV, beta |
| ENSMUSG00000075700 | 1.39 | 2.62 | 1.97E-15 | Selt | selenoprotein T |
| ENSMUSG00000027555 | 1.39 | 2.62 | 3.19E-12 | Car13 | carbonic anhydrase 13 |
| ENSMUSG00000040729 | 1.39 | 2.62 | 2.16E-04 | AK129341 | cDNA sequence AK129341 |
| ENSMUSG00000021427 | 1.39 | 2.62 | 1.16E-19 | Ssr1 | signal sequence receptor, alpha |
| ENSMUSG00000086409 | 1.39 | 2.62 | 1.77E-04 | NA | NA |
| ENSMUSG00000024346 | 1.39 | 2.62 | 3.76E-08 | Pfdn1 | prefoldin 1 |
| ENSMUSG00000043629 | 1.39 | 2.61 | 5.01E-03 | 1700019D03Rik | RIKEN cDNA 1700019D03 gene |
| ENSMUSG00000039706 | 1.39 | 2.61 | 3.63E-10 | Ldb2 | LIM domain binding 2 |
| ENSMUSG00000027993 | 1.39 | 2.61 | 3.11E-06 | Trim2 | tripartite motif-containing 2 |
| ENSMUSG00000018387 | 1.39 | 2.61 | 7.95E-05 | Shroom1 | shroom family member 1 |
| ENSMUSG00000030042 | 1.39 | 2.61 | 3.73E-19 | Pole4 | polymerase (DNA-directed), epsilon 4 (p12 subunit) |
| ENSMUSG00000072572 | 1.39 | 2.61 | 5.56E-03 | Slc39a2 | solute carrier family 39 (zinc transporter), member 2 |
| ENSMUSG00000037015 | 1.39 | 2.61 | 7.41E-15 | NA | NA |
| ENSMUSG00000012422 | 1.38 | 2.61 | 4.70E-13 | Tmem167 | transmembrane protein 167 |
| ENSMUSG00000024678 | 1.38 | 2.61 | 1.37E-07 | Ms4a4d | membrane-spanning 4-domains, subfamily A, member 4D |
| ENSMUSG00000026049 | 1.38 | 2.61 | 4.01E-06 | Tex30 | testis expressed 30 |
| ENSMUSG00000064372 | 1.38 | 2.61 | 1.37E-03 | NA | NA |
| ENSMUSG00000037254 | 1.38 | 2.61 | 4.62E-03 | Itih2 | inter-alpha trypsin inhibitor, heavy chain 2 |
| ENSMUSG00000060336 | 1.38 | 2.61 | 2.11E-03 | Zfp937 | zinc finger protein 937 |
| ENSMUSG00000001558 | 1.38 | 2.61 | 9.02E-12 | Klhl10 | kelch-like 10 |
| ENSMUSG00000037977 | 1.38 | 2.60 | 1.35E-03 | 6430571L13Rik | RIKEN cDNA 6430571L13 gene |
| ENSMUSG00000024759 | 1.38 | 2.60 | 5.47E-05 | Atl3 | atlastin GTPase 3 |
| ENSMUSG00000022358 | 1.38 | 2.60 | 1.98E-04 | Fbxo32 | F-box protein 32 |
| ENSMUSG00000031958 | 1.38 | 2.60 | 9.76E-07 | Ldhd | lactate dehydrogenase D |
| ENSMUSG00000033184 | 1.38 | 2.60 | 2.51E-09 | Tmed7 | transmembrane emp24 protein transport domain containing 7 |
| ENSMUSG00000027195 | 1.38 | 2.60 | 1.60E-16 | Hsd17b12 | hydroxysteroid (17-beta) dehydrogenase 12 |
| ENSMUSG00000054474 | 1.38 | 2.60 | 1.36E-06 | Thnsl2 | threonine synthase-like 2 (bacterial) |
| ENSMUSG00000032968 | 1.38 | 2.59 | 8.23E-04 | Inha | inhibin alpha |
| ENSMUSG00000019857 | 1.37 | 2.59 | 5.98E-07 | Asf1a | ASF1 anti-silencing function 1 homolog A (S. cerevisiae) |
| ENSMUSG00000074733 | 1.37 | 2.59 | 6.02E-10 | 5830428H23Rik | RIKEN cDNA 5830428H23 gene |
| ENSMUSG00000028841 | 1.37 | 2.59 | 2.98E-03 | Cnksr1 | connector enhancer of kinase suppressor of Ras 1 |
| ENSMUSG00000021763 | 1.37 | 2.59 | 2.88E-03 | BC067074 | cDNA sequence BC067074 |
| ENSMUSG00000046280 | 1.37 | 2.59 | 4.02E-14 | She | src homology 2 domain-containing transforming protein E |
| ENSMUSG00000068270 | 1.37 | 2.59 | 5.96E-15 | Shroom4 | shroom family member 4 |
| ENSMUSG00000041120 | 1.37 | 2.59 | 5.09E-05 | Nbl1 | neuroblastoma, suppression of tumorigenicity 1 |
| ENSMUSG00000079734 | 1.37 | 2.59 | 2.33E-05 | NA | NA |
| ENSMUSG00000019917 | 1.37 | 2.59 | 1.08E-05 | Sept10 | septin 10 |
| ENSMUSG00000033965 | 1.37 | 2.58 | 1.09E-06 | Slc16a2 | solute carrier family 16 (monocarboxylic acid transporters), member 2 |
| ENSMUSG00000027001 | 1.37 | 2.58 | 4.19E-05 | Dusp19 | dual specificity phosphatase 19 |
| ENSMUSG00000000753 | 1.37 | 2.58 | 4.17E-07 | Serpinf1 | serine (or cysteine) peptidase inhibitor, clade F, member 1 |
| ENSMUSG00000024317 | 1.37 | 2.58 | 7.35E-07 | Rnf138 | ring finger protein 138 |
| ENSMUSG00000063694 | 1.37 | 2.58 | 6.63E-06 | NA | NA |
| ENSMUSG00000039461 | 1.37 | 2.58 | 3.75E-09 | Tcta | T cell leukemia translocation altered gene |
| ENSMUSG00000073856 | 1.37 | 2.58 | 9.25E-07 | Iqck | IQ motif containing K |
| ENSMUSG00000082258 | 1.37 | 2.58 | 3.85E-03 | NA | NA |
| ENSMUSG00000019878 | 1.37 | 2.58 | 4.79E-05 | Hsf2 | heat shock factor 2 |
| ENSMUSG00000037032 | 1.37 | 2.58 | 2.15E-08 | Apbb1 | amyloid beta (A4) precursor protein-binding, family B, member 1 |
| ENSMUSG00000046447 | 1.37 | 2.58 | 5.45E-10 | Camk2n1 | calcium/calmodulin-dependent protein kinase II inhibitor 1 |
| ENSMUSG00000057497 | 1.37 | 2.58 | 3.51E-19 | Fam136a | family with sequence similarity 136, member A |
| ENSMUSG00000036282 | 1.37 | 2.58 | 1.80E-07 | Naa30 | N(alpha)-acetyltransferase 30, NatC catalytic subunit |
| ENSMUSG00000037096 | 1.37 | 2.58 | 6.27E-10 | NA | NA |
| ENSMUSG00000063160 | 1.37 | 2.58 | 1.62E-07 | Numbl | numb-like |
| ENSMUSG00000047714 | 1.37 | 2.58 | 2.37E-08 | Ppp1r2 | protein phosphatase 1, regulatory (inhibitor) subunit 2 |
| ENSMUSG00000003352 | 1.36 | 2.58 | 3.11E-07 | Cacnb3 | calcium channel, voltage-dependent, beta 3 subunit |
| ENSMUSG00000019210 | 1.36 | 2.57 | 4.62E-14 | Atp6v1e1 | ATPase, H+ transporting, lysosomal V1 subunit E1 |
| ENSMUSG00000067925 | 1.36 | 2.57 | 8.53E-08 | NA | NA |
| ENSMUSG00000066607 | 1.36 | 2.57 | 4.12E-08 | 6030419C18Rik | RIKEN cDNA 6030419C18 gene |
| ENSMUSG00000085348 | 1.36 | 2.57 | 5.44E-04 | LOC102633540 | uncharacterized LOC102633540 |
| ENSMUSG00000026728 | 1.36 | 2.57 | 1.16E-12 | Vim | vimentin |
| ENSMUSG00000034343 | 1.36 | 2.57 | 3.95E-03 | Ube2f | ubiquitin-conjugating enzyme E2F (putative) |
| ENSMUSG00000047712 | 1.36 | 2.57 | 2.22E-07 | Ust | uronyl-2-sulfotransferase |
| ENSMUSG00000038462 | 1.36 | 2.57 | 1.45E-12 | Uqcrfs1 | ubiquinol-cytochrome c reductase, Rieske iron-sulfur polypeptide 1 |
| ENSMUSG00000078779 | 1.36 | 2.57 | 5.88E-09 | Zfp59 | zinc finger protein 59 |
| ENSMUSG00000026697 | 1.36 | 2.57 | 9.45E-04 | Myoc | myocilin |
| ENSMUSG00000021668 | 1.36 | 2.57 | 9.29E-07 | Polk | polymerase (DNA directed), kappa |
| ENSMUSG00000082740 | 1.36 | 2.57 | 1.03E-03 | NA | NA |
| ENSMUSG00000042607 | 1.36 | 2.57 | 2.30E-04 | Asb4 | ankyrin repeat and SOCS box-containing 4 |
| ENSMUSG00000081390 | 1.36 | 2.56 | 9.48E-03 | NA | NA |
| ENSMUSG00000004105 | 1.36 | 2.56 | 3.29E-06 | Angptl2 | angiopoietin-like 2 |
| ENSMUSG00000085180 | 1.36 | 2.56 | 8.42E-04 | NA | NA |
| ENSMUSG00000078578 | 1.36 | 2.56 | 1.34E-11 | Ube2d3 | ubiquitin-conjugating enzyme E2D 3 |
| ENSMUSG00000052310 | 1.36 | 2.56 | 1.00E-11 | Slc39a1 | solute carrier family 39 (zinc transporter), member 1 |
| ENSMUSG00000079057 | 1.36 | 2.56 | 1.28E-07 | Cyp4v3 | cytochrome P450, family 4, subfamily v, polypeptide 3 |
| ENSMUSG00000063172 | 1.36 | 2.56 | 4.86E-04 | Hspb11 | heat shock protein family B (small), member 11 |
| ENSMUSG00000063087 | 1.36 | 2.56 | 1.70E-03 | Gm10125 | predicted gene 10125 |
| ENSMUSG00000030790 | 1.36 | 2.56 | 4.73E-06 | Adm | adrenomedullin |
| ENSMUSG00000078202 | 1.36 | 2.56 | 5.42E-08 | Nrarp | Notch-regulated ankyrin repeat protein |
| ENSMUSG00000027438 | 1.36 | 2.56 | 7.11E-10 | Napb | N-ethylmaleimide sensitive fusion protein attachment protein beta |
| ENSMUSG00000039646 | 1.36 | 2.56 | 1.91E-08 | Vasn | vasorin |
| ENSMUSG00000056069 | 1.35 | 2.56 | 2.59E-11 | Fam105a | family with sequence similarity 105, member A |
| ENSMUSG00000074796 | 1.35 | 2.56 | 2.10E-04 | Slc4a11 | solute carrier family 4, sodium bicarbonate transporter-like, member 11 |
| ENSMUSG00000041483 | 1.35 | 2.56 | 1.58E-04 | Zfp281 | zinc finger protein 281 |
| ENSMUSG00000086291 | 1.35 | 2.56 | 4.09E-04 | NA | NA |
| ENSMUSG00000024079 | 1.35 | 2.55 | 1.94E-10 | Eif2ak2 | eukaryotic translation initiation factor 2-alpha kinase 2 |
| ENSMUSG00000038319 | 1.35 | 2.55 | 7.76E-05 | Kcnh2 | potassium voltage-gated channel, subfamily H (eag-related), member 2 |
| ENSMUSG00000020817 | 1.35 | 2.55 | 8.88E-15 | Rabep1 | rabaptin, RAB GTPase binding effector protein 1 |
| ENSMUSG00000041827 | 1.35 | 2.55 | 2.08E-08 | Oasl1 | 2'-5' oligoadenylate synthetase-like 1 |
| ENSMUSG00000038233 | 1.35 | 2.55 | 7.66E-07 | Fam198a | family with sequence similarity 198, member A |
| ENSMUSG00000049985 | 1.35 | 2.55 | 9.92E-03 | Ankrd55 | ankyrin repeat domain 55 |
| ENSMUSG00000021506 | 1.35 | 2.55 | 2.53E-05 | Pitx1 | paired-like homeodomain transcription factor 1 |
| ENSMUSG00000030613 | 1.35 | 2.55 | 2.95E-05 | Ccdc90b | coiled-coil domain containing 90B |
| ENSMUSG00000022043 | 1.35 | 2.55 | 1.46E-16 | Trim35 | tripartite motif-containing 35 |
| ENSMUSG00000027099 | 1.35 | 2.54 | 5.32E-22 | Mtx2 | metaxin 2 |
| ENSMUSG00000039981 | 1.35 | 2.54 | 1.98E-07 | Zc3h12d | zinc finger CCCH type containing 12D |
| ENSMUSG00000024013 | 1.35 | 2.54 | 1.82E-08 | Fgd2 | FYVE, RhoGEF and PH domain containing 2 |
| ENSMUSG00000072857 | 1.34 | 2.54 | 1.56E-06 | NA | NA |
| ENSMUSG00000006221 | 1.34 | 2.54 | 1.02E-03 | Hspb7 | heat shock protein family, member 7 (cardiovascular) |
| ENSMUSG00000040747 | 1.34 | 2.54 | 2.75E-20 | Cd53 | CD53 antigen |
| ENSMUSG00000056427 | 1.34 | 2.54 | 7.11E-08 | Slit3 | slit homolog 3 (Drosophila) |
| ENSMUSG00000020928 | 1.34 | 2.54 | 1.61E-03 | Higd1b | HIG1 domain family, member 1B |
| ENSMUSG00000036061 | 1.34 | 2.54 | 9.85E-11 | Smug1 | single-strand selective monofunctional uracil DNA glycosylase |
| ENSMUSG00000051212 | 1.34 | 2.54 | 9.49E-08 | Gpr183 | G protein-coupled receptor 183 |
| ENSMUSG00000043259 | 1.34 | 2.53 | 3.52E-07 | Fam13c | family with sequence similarity 13, member C |
| ENSMUSG00000005610 | 1.34 | 2.53 | 2.68E-11 | Eif4g2 | eukaryotic translation initiation factor 4, gamma 2 |
| ENSMUSG00000026387 | 1.34 | 2.53 | 1.91E-03 | Sctr | secretin receptor |
| ENSMUSG00000002778 | 1.34 | 2.53 | 1.70E-11 | Kdelr1 | KDEL (Lys-Asp-Glu-Leu) endoplasmic reticulum protein retention receptor 1 |
| ENSMUSG00000025283 | 1.34 | 2.53 | 7.96E-17 | Sat1 | spermidine/spermine N1-acetyl transferase 1 |
| ENSMUSG00000029913 | 1.34 | 2.53 | 1.05E-08 | Prdm5 | PR domain containing 5 |
| ENSMUSG00000085148 | 1.34 | 2.53 | 1.85E-07 | Mir22hg | Mir22 host gene (non-protein coding) |
| ENSMUSG00000040124 | 1.34 | 2.53 | 2.04E-11 | Gorab | golgin, RAB6-interacting |
| ENSMUSG00000056367 | 1.34 | 2.53 | 1.74E-07 | Actr3b | ARP3 actin-related protein 3B |
| ENSMUSG00000027799 | 1.34 | 2.53 | 2.42E-07 | Nbea | neurobeachin |
| ENSMUSG00000025738 | 1.34 | 2.53 | 2.45E-07 | Fbxl16 | F-box and leucine-rich repeat protein 16 |
| ENSMUSG00000062931 | 1.34 | 2.53 | 1.94E-05 | Zfp938 | zinc finger protein 938 |
| ENSMUSG00000051615 | 1.34 | 2.53 | 2.21E-29 | Rap2a | RAS related protein 2a |
| ENSMUSG00000025216 | 1.34 | 2.53 | 9.67E-04 | Lbx1 | ladybird homeobox homolog 1 (Drosophila) |
| ENSMUSG00000036977 | 1.34 | 2.53 | 8.00E-06 | Anapc10 | anaphase promoting complex subunit 10 |
| ENSMUSG00000079317 | 1.34 | 2.53 | 1.21E-05 | Trappc2 | trafficking protein particle complex 2 |
| ENSMUSG00000014075 | 1.34 | 2.53 | 3.18E-08 | Tctex1d2 | Tctex1 domain containing 2 |
| ENSMUSG00000031618 | 1.34 | 2.53 | 4.67E-04 | Nr3c2 | nuclear receptor subfamily 3, group C, member 2 |
| ENSMUSG00000032381 | 1.33 | 2.52 | 8.07E-14 | Fam96a | family with sequence similarity 96, member A |
| ENSMUSG00000034573 | 1.33 | 2.52 | 2.49E-12 | Ptpn13 | protein tyrosine phosphatase, non-receptor type 13 |
| ENSMUSG00000005716 | 1.33 | 2.52 | 3.53E-04 | Pvalb | parvalbumin |
| ENSMUSG00000026829 | 1.33 | 2.52 | 3.04E-05 | Gbgt1 | globoside alpha-1,3-N-acetylgalactosaminyltransferase 1 |
| ENSMUSG00000032690 | 1.33 | 2.52 | 5.64E-10 | Oas2 | 2'-5' oligoadenylate synthetase 2 |
| ENSMUSG00000085311 | 1.33 | 2.52 | 7.15E-03 | NA | NA |
| ENSMUSG00000037640 | 1.33 | 2.52 | 2.11E-07 | Zfp60 | zinc finger protein 60 |
| ENSMUSG00000031007 | 1.33 | 2.51 | 2.50E-21 | Atp6ap2 | ATPase, H+ transporting, lysosomal accessory protein 2 |
| ENSMUSG00000022911 | 1.33 | 2.51 | 1.30E-09 | Arl13b | ADP-ribosylation factor-like 13B |
| ENSMUSG00000046798 | 1.33 | 2.51 | 8.70E-13 | Cldn12 | claudin 12 |
| ENSMUSG00000031198 | 1.33 | 2.51 | 2.35E-11 | Fundc2 | FUN14 domain containing 2 |
| ENSMUSG00000082401 | 1.33 | 2.51 | 4.36E-10 | NA | NA |
| ENSMUSG00000057409 | 1.33 | 2.51 | 3.16E-06 | Zfp53 | zinc finger protein 53 |
| ENSMUSG00000026546 | 1.33 | 2.51 | 2.19E-08 | Ccdc19 | coiled-coil domain containing 19 |
| ENSMUSG00000004952 | 1.33 | 2.51 | 6.40E-09 | Rasa4 | RAS p21 protein activator 4 |
| ENSMUSG00000023959 | 1.33 | 2.51 | 6.08E-06 | Clic5 | chloride intracellular channel 5 |
| ENSMUSG00000062760 | 1.33 | 2.51 | 6.09E-05 | 1810041L15Rik | RIKEN cDNA 1810041L15 gene |
| ENSMUSG00000032558 | 1.33 | 2.51 | 1.17E-09 | Nphp3 | nephronophthisis 3 (adolescent) |
| ENSMUSG00000028631 | 1.33 | 2.51 | 4.60E-04 | Kcnq4 | potassium voltage-gated channel, subfamily Q, member 4 |
| ENSMUSG00000026249 | 1.32 | 2.51 | 1.43E-10 | Serpine2 | serine (or cysteine) peptidase inhibitor, clade E, member 2 |
| ENSMUSG00000029231 | 1.32 | 2.50 | 1.06E-10 | Pdgfra | platelet derived growth factor receptor, alpha polypeptide |
| ENSMUSG00000021930 | 1.32 | 2.50 | 5.87E-06 | Spryd7 | SPRY domain containing 7 |
| ENSMUSG00000024245 | 1.32 | 2.50 | 3.76E-04 | Tmem178 | transmembrane protein 178 |
| ENSMUSG00000086914 | 1.32 | 2.50 | 1.05E-03 | LOC102636154 | uncharacterized LOC102636154 |
| ENSMUSG00000015468 | 1.32 | 2.50 | 2.93E-10 | Notch4 | notch 4 |
| ENSMUSG00000040666 | 1.32 | 2.50 | 7.80E-05 | Sh3bgr | SH3-binding domain glutamic acid-rich protein |
| ENSMUSG00000075585 | 1.32 | 2.50 | 8.72E-05 | NA | NA |
| ENSMUSG00000022847 | 1.32 | 2.50 | 9.75E-04 | Thpo | thrombopoietin |
| ENSMUSG00000025268 | 1.32 | 2.50 | 1.72E-08 | Maged2 | melanoma antigen, family D, 2 |
| ENSMUSG00000038521 | 1.32 | 2.50 | 4.45E-06 | C1s | complement component 1, s subcomponent |
| ENSMUSG00000022860 | 1.32 | 2.50 | 2.68E-03 | Chodl | chondrolectin |
| ENSMUSG00000042821 | 1.32 | 2.50 | 5.48E-06 | Snai1 | snail homolog 1 (Drosophila) |
| ENSMUSG00000029161 | 1.32 | 2.50 | 9.22E-05 | Cgref1 | cell growth regulator with EF hand domain 1 |
| ENSMUSG00000039908 | 1.32 | 2.50 | 1.32E-09 | Slc26a11 | solute carrier family 26, member 11 |
| ENSMUSG00000037826 | 1.32 | 2.50 | 8.19E-11 | Ppm1k | protein phosphatase 1K (PP2C domain containing) |
| ENSMUSG00000028199 | 1.32 | 2.50 | 4.14E-06 | Cryz | crystallin, zeta |
| ENSMUSG00000025816 | 1.32 | 2.50 | 7.13E-12 | Sec61a2 | Sec61, alpha subunit 2 (S. cerevisiae) |
| ENSMUSG00000024274 | 1.32 | 2.50 | 1.25E-03 | NA | NA |
| ENSMUSG00000041420 | 1.32 | 2.50 | 1.23E-07 | Meis3 | Meis homeobox 3 |
| ENSMUSG00000025150 | 1.32 | 2.50 | 1.74E-06 | Cbr2 | carbonyl reductase 2 |
| ENSMUSG00000072566 | 1.32 | 2.50 | 3.03E-07 | NA | NA |
| ENSMUSG00000025475 | 1.32 | 2.50 | 3.96E-03 | Gpr123 | G protein-coupled receptor 123 |
| ENSMUSG00000040782 | 1.32 | 2.49 | 2.53E-09 | Rfwd2 | ring finger and WD repeat domain 2 |
| ENSMUSG00000073139 | 1.32 | 2.49 | 7.19E-11 | BC023829 | cDNA sequence BC023829 |
| ENSMUSG00000027175 | 1.32 | 2.49 | 4.59E-09 | Tcp11l1 | t-complex 11 like 1 |
| ENSMUSG00000031636 | 1.32 | 2.49 | 2.42E-04 | Pdlim3 | PDZ and LIM domain 3 |
| ENSMUSG00000028919 | 1.32 | 2.49 | 2.52E-09 | Arhgef19 | Rho guanine nucleotide exchange factor (GEF) 19 |
| ENSMUSG00000039684 | 1.32 | 2.49 | 5.48E-18 | NA | NA |
| ENSMUSG00000047379 | 1.32 | 2.49 | 6.71E-12 | B3gnt1 | UDP-GlcNAc:betaGal beta-1,3-N-acetylglucosaminyltransferase 1 |
| ENSMUSG00000034168 | 1.31 | 2.49 | 1.27E-03 | Irf2bpl | interferon regulatory factor 2 binding protein-like |
| ENSMUSG00000079588 | 1.31 | 2.49 | 1.41E-04 | Tmem182 | transmembrane protein 182 |
| ENSMUSG00000041695 | 1.31 | 2.49 | 2.44E-05 | Kcnj2 | potassium inwardly-rectifying channel, subfamily J, member 2 |
| ENSMUSG00000042787 | 1.31 | 2.49 | 1.87E-08 | Exog | endo/exonuclease (5'-3'), endonuclease G-like |
| ENSMUSG00000026921 | 1.31 | 2.49 | 6.60E-12 | Egfl7 | EGF-like domain 7 |
| ENSMUSG00000004383 | 1.31 | 2.49 | 7.97E-08 | Large | like-glycosyltransferase |
| ENSMUSG00000001497 | 1.31 | 2.49 | 6.49E-03 | Pax9 | paired box 9 |
| ENSMUSG00000035969 | 1.31 | 2.48 | 1.93E-07 | Rusc2 | RUN and SH3 domain containing 2 |
| ENSMUSG00000019853 | 1.31 | 2.48 | 4.90E-05 | Hebp2 | heme binding protein 2 |
| ENSMUSG00000036330 | 1.31 | 2.48 | 2.13E-03 | Slc18a1 | solute carrier family 18 (vesicular monoamine), member 1 |
| ENSMUSG00000022014 | 1.31 | 2.48 | 7.16E-08 | Epsti1 | epithelial stromal interaction 1 (breast) |
| ENSMUSG00000036832 | 1.31 | 2.48 | 3.76E-05 | Lpar3 | lysophosphatidic acid receptor 3 |
| ENSMUSG00000052560 | 1.31 | 2.48 | 1.88E-04 | Cpne8 | copine VIII |
| ENSMUSG00000021432 | 1.31 | 2.48 | 3.60E-15 | Slc35b3 | solute carrier family 35, member B3 |
| ENSMUSG00000056947 | 1.31 | 2.48 | 3.02E-03 | Mab21l1 | mab-21-like 1 (C. elegans) |
| ENSMUSG00000032232 | 1.31 | 2.48 | 1.33E-11 | Cgnl1 | cingulin-like 1 |
| ENSMUSG00000017737 | 1.31 | 2.48 | 5.28E-16 | Mmp9 | matrix metallopeptidase 9 |
| ENSMUSG00000043421 | 1.31 | 2.48 | 1.73E-05 | Hilpda | hypoxia inducible lipid droplet associated |
| ENSMUSG00000041688 | 1.31 | 2.48 | 4.23E-03 | Amot | angiomotin |
| ENSMUSG00000020773 | 1.31 | 2.48 | 8.91E-08 | Trim47 | tripartite motif-containing 47 |
| ENSMUSG00000030852 | 1.31 | 2.48 | 5.31E-04 | Tacc2 | transforming, acidic coiled-coil containing protein 2 |
| ENSMUSG00000072836 | 1.31 | 2.47 | 5.55E-03 | NA | NA |
| ENSMUSG00000050390 | 1.31 | 2.47 | 2.37E-05 | C77080 | expressed sequence C77080 |
| ENSMUSG00000005078 | 1.31 | 2.47 | 8.76E-15 | Jkamp | JNK1/MAPK8-associated membrane protein |
| ENSMUSG00000021250 | 1.31 | 2.47 | 1.54E-06 | Fos | FBJ osteosarcoma oncogene |
| ENSMUSG00000026828 | 1.31 | 2.47 | 3.36E-04 | Galnt5 | UDP-N-acetyl-alpha-D-galactosamine:polypeptide N-acetylgalactosaminyltransferase 5 |
| ENSMUSG00000025170 | 1.31 | 2.47 | 2.28E-05 | Rab40b | Rab40b, member RAS oncogene family |
| ENSMUSG00000073639 | 1.31 | 2.47 | 4.98E-10 | Rab18 | RAB18, member RAS oncogene family |
| ENSMUSG00000026864 | 1.31 | 2.47 | 8.88E-08 | Hspa5 | heat shock protein 5 |
| ENSMUSG00000068523 | 1.30 | 2.47 | 4.98E-05 | NA | NA |
| ENSMUSG00000034501 | 1.30 | 2.47 | 3.75E-13 | Pcnxl4 | pecanex-like 4 (Drosophila) |
| ENSMUSG00000006411 | 1.30 | 2.47 | 7.35E-08 | Pvrl4 | poliovirus receptor-related 4 |
| ENSMUSG00000086877 | 1.30 | 2.47 | 1.85E-03 | NA | NA |
| ENSMUSG00000023249 | 1.30 | 2.47 | 1.53E-13 | Parp3 | poly (ADP-ribose) polymerase family, member 3 |
| ENSMUSG00000013033 | 1.30 | 2.47 | 6.57E-11 | Lphn1 | latrophilin 1 |
| ENSMUSG00000034101 | 1.30 | 2.47 | 1.27E-17 | Ctnnd1 | catenin (cadherin associated protein), delta 1 |
| ENSMUSG00000059796 | 1.30 | 2.46 | 1.05E-08 | Eif4a1 | eukaryotic translation initiation factor 4A1 |
| ENSMUSG00000021712 | 1.30 | 2.46 | 3.21E-07 | Trim23 | tripartite motif-containing 23 |
| ENSMUSG00000079434 | 1.30 | 2.46 | 6.83E-03 | Neu2 | neuraminidase 2 |
| ENSMUSG00000033788 | 1.30 | 2.46 | 7.28E-16 | Dysf | dysferlin |
| ENSMUSG00000039007 | 1.30 | 2.46 | 6.17E-14 | Cpq | carboxypeptidase Q |
| ENSMUSG00000027879 | 1.30 | 2.46 | 3.06E-17 | Sec22b | SEC22 vesicle trafficking protein homolog B (S. cerevisiae) |
| ENSMUSG00000046610 | 1.30 | 2.46 | 8.38E-03 | Oacyl | O-acyltransferase like |
| ENSMUSG00000047643 | 1.30 | 2.46 | 2.16E-03 | NA | NA |
| ENSMUSG00000000555 | 1.30 | 2.46 | 7.15E-14 | Itga5 | integrin alpha 5 (fibronectin receptor alpha) |
| ENSMUSG00000015437 | 1.30 | 2.46 | 9.81E-05 | Gzmb | granzyme B |
| ENSMUSG00000029648 | 1.30 | 2.46 | 5.08E-09 | Flt1 | FMS-like tyrosine kinase 1 |
| ENSMUSG00000043501 | 1.30 | 2.46 | 6.93E-03 | Lgals2 | lectin, galactose-binding, soluble 2 |
| ENSMUSG00000016995 | 1.30 | 2.46 | 4.40E-03 | Matn4 | matrilin 4 |
| ENSMUSG00000035171 | 1.30 | 2.46 | 8.87E-06 | 1110059E24Rik | RIKEN cDNA 1110059E24 gene |
| ENSMUSG00000014776 | 1.30 | 2.46 | 6.01E-07 | Nol3 | nucleolar protein 3 (apoptosis repressor with CARD domain) |
| ENSMUSG00000034412 | 1.30 | 2.46 | 7.14E-10 | Tbc1d10a | TBC1 domain family, member 10a |
| ENSMUSG00000030020 | 1.30 | 2.46 | 5.51E-07 | Prickle2 | prickle homolog 2 (Drosophila) |
| ENSMUSG00000049624 | 1.30 | 2.46 | 7.03E-13 | Slc17a5 | solute carrier family 17 (anion/sugar transporter), member 5 |
| ENSMUSG00000049532 | 1.30 | 2.46 | 4.92E-15 | Sall2 | sal-like 2 (Drosophila) |
| ENSMUSG00000022772 | 1.30 | 2.46 | 2.02E-06 | Senp5 | SUMO/sentrin specific peptidase 5 |
| ENSMUSG00000028180 | 1.30 | 2.46 | 1.62E-10 | Zranb2 | zinc finger, RAN-binding domain containing 2 |
| ENSMUSG00000021909 | 1.29 | 2.45 | 1.54E-03 | NA | NA |
| ENSMUSG00000051359 | 1.29 | 2.45 | 1.74E-04 | Ncald | neurocalcin delta |
| ENSMUSG00000052373 | 1.29 | 2.45 | 4.23E-04 | Mpp3 | membrane protein, palmitoylated 3 (MAGUK p55 subfamily member 3) |
| ENSMUSG00000029228 | 1.29 | 2.45 | 2.54E-03 | Lnx1 | ligand of numb-protein X 1 |
| ENSMUSG00000062373 | 1.29 | 2.45 | 1.03E-10 | Tmem65 | transmembrane protein 65 |
| ENSMUSG00000030187 | 1.29 | 2.45 | 8.81E-10 | Klra2 | killer cell lectin-like receptor, subfamily A, member 2 |
| ENSMUSG00000039431 | 1.29 | 2.45 | 5.25E-03 | Mtmr7 | myotubularin related protein 7 |
| ENSMUSG00000044330 | 1.29 | 2.45 | 1.96E-11 | NA | NA |
| ENSMUSG00000033644 | 1.29 | 2.45 | 3.15E-05 | Piwil2 | piwi-like RNA-mediated gene silencing 2 |
| ENSMUSG00000085906 | 1.29 | 2.45 | 5.69E-04 | NA | NA |
| ENSMUSG00000033933 | 1.29 | 2.45 | 1.39E-14 | Vhl | von Hippel-Lindau tumor suppressor |
| ENSMUSG00000035104 | 1.29 | 2.44 | 2.11E-08 | Eva1a | eva-1 homolog A (C. elegans) |
| ENSMUSG00000042793 | 1.29 | 2.44 | 3.37E-04 | Lgr6 | leucine-rich repeat-containing G protein-coupled receptor 6 |
| ENSMUSG00000002249 | 1.29 | 2.44 | 5.83E-08 | Tead3 | TEA domain family member 3 |
| ENSMUSG00000027777 | 1.29 | 2.44 | 1.16E-06 | NA | NA |
| ENSMUSG00000020283 | 1.29 | 2.44 | 1.66E-10 | Pex13 | peroxisomal biogenesis factor 13 |
| ENSMUSG00000022844 | 1.29 | 2.44 | 4.29E-07 | Pdia5 | protein disulfide isomerase associated 5 |
| ENSMUSG00000057335 | 1.29 | 2.44 | 5.08E-05 | Cep170 | centrosomal protein 170 |
| ENSMUSG00000028926 | 1.29 | 2.44 | 9.10E-12 | Cdk14 | cyclin-dependent kinase 14 |
| ENSMUSG00000018574 | 1.28 | 2.44 | 1.76E-12 | Acadvl | acyl-Coenzyme A dehydrogenase, very long chain |
| ENSMUSG00000043929 | 1.28 | 2.44 | 6.87E-06 | Klhl15 | kelch-like 15 |
| ENSMUSG00000028645 | 1.28 | 2.43 | 1.97E-08 | Slc2a1 | solute carrier family 2 (facilitated glucose transporter), member 1 |
| ENSMUSG00000015312 | 1.28 | 2.43 | 7.37E-06 | Gadd45b | growth arrest and DNA-damage-inducible 45 beta |
| ENSMUSG00000034463 | 1.28 | 2.43 | 1.15E-07 | Scara3 | scavenger receptor class A, member 3 |
| ENSMUSG00000022464 | 1.28 | 2.43 | 3.04E-03 | Slc38a4 | solute carrier family 38, member 4 |
| ENSMUSG00000071291 | 1.28 | 2.43 | 3.55E-06 | Zfp58 | zinc finger protein 58 |
| ENSMUSG00000020386 | 1.28 | 2.43 | 6.89E-07 | Sar1b | SAR1 gene homolog B (S. cerevisiae) |
| ENSMUSG00000038836 | 1.28 | 2.43 | 1.33E-07 | Agbl3 | ATP/GTP binding protein-like 3 |
| ENSMUSG00000020644 | 1.28 | 2.43 | 7.68E-09 | Id2 | inhibitor of DNA binding 2 |
| ENSMUSG00000025016 | 1.28 | 2.43 | 6.41E-24 | Tm9sf3 | transmembrane 9 superfamily member 3 |
| ENSMUSG00000044864 | 1.28 | 2.43 | 9.96E-12 | Ankrd50 | ankyrin repeat domain 50 |
| ENSMUSG00000086878 | 1.28 | 2.43 | 4.43E-03 | NA | NA |
| ENSMUSG00000020886 | 1.28 | 2.43 | 2.04E-07 | Dlg4 | discs, large homolog 4 (Drosophila) |
| ENSMUSG00000085517 | 1.28 | 2.43 | 2.18E-05 | NA | NA |
| ENSMUSG00000028396 | 1.28 | 2.42 | 4.71E-05 | 2310002L09Rik | RIKEN cDNA 2310002L09 gene |
| ENSMUSG00000023009 | 1.28 | 2.42 | 1.30E-11 | Nckap5l | NCK-associated protein 5-like |
| ENSMUSG00000074861 | 1.28 | 2.42 | 3.64E-04 | NA | NA |
| ENSMUSG00000025956 | 1.28 | 2.42 | 3.17E-08 | Mettl21a | methyltransferase like 21A |
| ENSMUSG00000030428 | 1.28 | 2.42 | 1.01E-03 | Ttyh1 | tweety homolog 1 (Drosophila) |
| ENSMUSG00000051234 | 1.28 | 2.42 | 2.41E-07 | Rnf7 | ring finger protein 7 |
| ENSMUSG00000028256 | 1.27 | 2.42 | 5.74E-05 | Odf2l | outer dense fiber of sperm tails 2-like |
| ENSMUSG00000035390 | 1.27 | 2.42 | 1.27E-10 | Brsk1 | BR serine/threonine kinase 1 |
| ENSMUSG00000036309 | 1.27 | 2.42 | 4.91E-09 | Skp1a | S-phase kinase-associated protein 1A |
| ENSMUSG00000028184 | 1.27 | 2.42 | 4.08E-07 | Lphn2 | latrophilin 2 |
| ENSMUSG00000056429 | 1.27 | 2.42 | 3.46E-22 | NA | NA |
| ENSMUSG00000073131 | 1.27 | 2.42 | 1.90E-05 | Vma21 | VMA21 vacuolar H+-ATPase homolog (S. cerevisiae) |
| ENSMUSG00000071180 | 1.27 | 2.42 | 1.83E-10 | Smim15 | small integral membrane protein 15 |
| ENSMUSG00000063972 | 1.27 | 2.42 | 1.38E-04 | Nr6a1 | nuclear receptor subfamily 6, group A, member 1 |
| ENSMUSG00000044502 | 1.27 | 2.41 | 6.98E-10 | Bod1 | biorientation of chromosomes in cell division 1 |
| ENSMUSG00000030804 | 1.27 | 2.41 | 9.94E-08 | NA | NA |
| ENSMUSG00000042063 | 1.27 | 2.41 | 5.22E-08 | Zfp386 | zinc finger protein 386 (Kruppel-like) |
| ENSMUSG00000014905 | 1.27 | 2.41 | 1.69E-05 | Dnajb9 | DnaJ (Hsp40) homolog, subfamily B, member 9 |
| ENSMUSG00000025912 | 1.27 | 2.41 | 8.01E-07 | Mybl1 | myeloblastosis oncogene-like 1 |
| ENSMUSG00000055926 | 1.27 | 2.41 | 4.24E-06 | Gm14137 | predicted gene 14137 |
| ENSMUSG00000024935 | 1.27 | 2.41 | 7.86E-03 | Slc1a1 | solute carrier family 1 (neuronal/epithelial high affinity glutamate transporter, system Xag), member 1 |
| ENSMUSG00000047945 | 1.27 | 2.41 | 1.35E-08 | Marcksl1 | MARCKS-like 1 |
| ENSMUSG00000035992 | 1.27 | 2.41 | 7.09E-04 | Fnip1 | folliculin interacting protein 1 |
| ENSMUSG00000060636 | 1.27 | 2.41 | 2.39E-07 | NA | NA |
| ENSMUSG00000020952 | 1.27 | 2.41 | 4.20E-07 | Scfd1 | Sec1 family domain containing 1 |
| ENSMUSG00000082016 | 1.27 | 2.41 | 3.51E-10 | NA | NA |
| ENSMUSG00000078781 | 1.27 | 2.41 | 5.55E-03 | NA | NA |
| ENSMUSG00000038086 | 1.27 | 2.41 | 5.76E-05 | Hspb2 | heat shock protein 2 |
| ENSMUSG00000048960 | 1.27 | 2.40 | 7.55E-04 | Prex2 | phosphatidylinositol-3,4,5-trisphosphate-dependent Rac exchange factor 2 |
| ENSMUSG00000001768 | 1.27 | 2.40 | 1.34E-20 | Rin2 | Ras and Rab interactor 2 |
| ENSMUSG00000002020 | 1.27 | 2.40 | 7.02E-06 | Ltbp2 | latent transforming growth factor beta binding protein 2 |
| ENSMUSG00000045404 | 1.27 | 2.40 | 1.03E-06 | Kcnk13 | potassium channel, subfamily K, member 13 |
| ENSMUSG00000008206 | 1.26 | 2.40 | 1.75E-08 | Cers4 | ceramide synthase 4 |
| ENSMUSG00000006386 | 1.26 | 2.40 | 2.14E-10 | Tek | endothelial-specific receptor tyrosine kinase |
| ENSMUSG00000089824 | 1.26 | 2.40 | 1.39E-08 | Rbm12 | RNA binding motif protein 12 |
| ENSMUSG00000049488 | 1.26 | 2.40 | 2.79E-07 | Tmem67 | transmembrane protein 67 |
| ENSMUSG00000031642 | 1.26 | 2.40 | 1.77E-13 | Sh3rf1 | SH3 domain containing ring finger 1 |
| ENSMUSG00000023067 | 1.26 | 2.40 | 2.93E-09 | Cdkn1a | cyclin-dependent kinase inhibitor 1A (P21) |
| ENSMUSG00000032531 | 1.26 | 2.40 | 1.37E-08 | Amotl2 | angiomotin-like 2 |
| ENSMUSG00000028264 | 1.26 | 2.40 | 1.82E-03 | Spaca1 | sperm acrosome associated 1 |
| ENSMUSG00000021816 | 1.26 | 2.40 | 4.03E-16 | Ppp3cb | protein phosphatase 3, catalytic subunit, beta isoform |
| ENSMUSG00000004936 | 1.26 | 2.40 | 1.93E-13 | Map2k1 | mitogen-activated protein kinase kinase 1 |
| ENSMUSG00000022184 | 1.26 | 2.40 | 8.87E-08 | Fbxo4 | F-box protein 4 |
| ENSMUSG00000036368 | 1.26 | 2.39 | 3.28E-04 | Rmdn2 | regulator of microtubule dynamics 2 |
| ENSMUSG00000020682 | 1.26 | 2.39 | 5.22E-08 | Mmp28 | matrix metallopeptidase 28 (epilysin) |
| ENSMUSG00000050105 | 1.26 | 2.39 | 9.21E-11 | Grrp1 | glycine/arginine rich protein 1 |
| ENSMUSG00000002393 | 1.26 | 2.39 | 7.74E-04 | Nr2f6 | nuclear receptor subfamily 2, group F, member 6 |
| ENSMUSG00000024544 | 1.26 | 2.39 | 1.96E-09 | Ldlrad4 | low density lipoprotein receptor class A domain containing 4 |
| ENSMUSG00000071573 | 1.26 | 2.39 | 3.16E-04 | Rnls | renalase, FAD-dependent amine oxidase |
| ENSMUSG00000030386 | 1.26 | 2.39 | 1.42E-07 | Zfp606 | zinc finger protein 606 |
| ENSMUSG00000049184 | 1.26 | 2.39 | 1.47E-04 | Purg | purine-rich element binding protein G |
| ENSMUSG00000048332 | 1.26 | 2.39 | 6.14E-08 | Lhfp | lipoma HMGIC fusion partner |
| ENSMUSG00000045107 | 1.26 | 2.39 | 4.14E-05 | Saysd1 | SAYSVFN motif domain containing 1 |
| ENSMUSG00000002688 | 1.26 | 2.39 | 8.55E-06 | Prkd1 | protein kinase D1 |
| ENSMUSG00000033377 | 1.25 | 2.39 | 2.62E-06 | Palmd | palmdelphin |
| ENSMUSG00000025544 | 1.25 | 2.39 | 3.53E-14 | Tm9sf2 | transmembrane 9 superfamily member 2 |
| ENSMUSG00000087502 | 1.25 | 2.38 | 4.37E-03 | NA | NA |
| ENSMUSG00000050088 | 1.25 | 2.38 | 5.52E-20 | 1600012H06Rik | RIKEN cDNA 1600012H06 gene |
| ENSMUSG00000026617 | 1.25 | 2.38 | 1.87E-12 | Bpnt1 | bisphosphate 3'-nucleotidase 1 |
| ENSMUSG00000028563 | 1.25 | 2.38 | 1.29E-04 | Tm2d1 | TM2 domain containing 1 |
| ENSMUSG00000016833 | 1.25 | 2.38 | 4.20E-05 | Mrps18c | mitochondrial ribosomal protein S18C |
| ENSMUSG00000078780 | 1.25 | 2.38 | 2.29E-04 | Gm5150 | predicted gene 5150 |
| ENSMUSG00000030306 | 1.25 | 2.38 | 2.12E-06 | Tmtc1 | transmembrane and tetratricopeptide repeat containing 1 |
| ENSMUSG00000024294 | 1.25 | 2.38 | 5.07E-10 | Mib1 | mindbomb homolog 1 (Drosophila) |
| ENSMUSG00000070577 | 1.25 | 2.38 | 1.53E-03 | Gm572 | predicted gene 572 |
| ENSMUSG00000085013 | 1.25 | 2.38 | 6.33E-06 | NA | NA |
| ENSMUSG00000044949 | 1.25 | 2.38 | 3.18E-06 | Ubtd2 | ubiquitin domain containing 2 |
| ENSMUSG00000006281 | 1.25 | 2.38 | 2.06E-06 | Tep1 | telomerase associated protein 1 |
| ENSMUSG00000058447 | 1.25 | 2.38 | 1.55E-04 | NA | NA |
| ENSMUSG00000039579 | 1.25 | 2.38 | 3.12E-04 | Grin3a | glutamate receptor ionotropic, NMDA3A |
| ENSMUSG00000053460 | 1.25 | 2.38 | 6.01E-15 | Ggcx | gamma-glutamyl carboxylase |
| ENSMUSG00000000340 | 1.25 | 2.38 | 1.13E-10 | Dbt | dihydrolipoamide branched chain transacylase E2 |
| ENSMUSG00000002105 | 1.25 | 2.37 | 4.98E-13 | Slc39a13 | solute carrier family 39 (metal ion transporter), member 13 |
| ENSMUSG00000018012 | 1.25 | 2.37 | 1.48E-05 | Rac3 | RAS-related C3 botulinum substrate 3 |
| ENSMUSG00000029661 | 1.25 | 2.37 | 5.45E-06 | Col1a2 | collagen, type I, alpha 2 |
| ENSMUSG00000083097 | 1.25 | 2.37 | 2.03E-06 | NA | NA |
| ENSMUSG00000032606 | 1.24 | 2.37 | 3.41E-06 | Nicn1 | nicolin 1 |
| ENSMUSG00000024754 | 1.24 | 2.37 | 6.07E-05 | Tmem2 | transmembrane protein 2 |
| ENSMUSG00000029468 | 1.24 | 2.37 | 7.30E-19 | P2rx7 | purinergic receptor P2X, ligand-gated ion channel, 7 |
| ENSMUSG00000084349 | 1.24 | 2.37 | 1.32E-07 | NA | NA |
| ENSMUSG00000055436 | 1.24 | 2.37 | 3.11E-14 | Srsf11 | serine/arginine-rich splicing factor 11 |
| ENSMUSG00000040370 | 1.24 | 2.36 | 1.50E-08 | Lyrm5 | LYR motif containing 5 |
| ENSMUSG00000024793 | 1.24 | 2.36 | 3.61E-03 | Tnfrsf25 | tumor necrosis factor receptor superfamily, member 25 |
| ENSMUSG00000039096 | 1.24 | 2.36 | 8.49E-05 | Rsad1 | radical S-adenosyl methionine domain containing 1 |
| ENSMUSG00000053580 | 1.24 | 2.36 | 8.83E-11 | Tanc2 | tetratricopeptide repeat, ankyrin repeat and coiled-coil containing 2 |
| ENSMUSG00000046096 | 1.24 | 2.36 | 1.26E-07 | BC030336 | cDNA sequence BC030336 |
| ENSMUSG00000033991 | 1.24 | 2.36 | 2.42E-08 | Ttc37 | tetratricopeptide repeat domain 37 |
| ENSMUSG00000040774 | 1.24 | 2.36 | 5.47E-10 | Cept1 | choline/ethanolaminephosphotransferase 1 |
| ENSMUSG00000025451 | 1.24 | 2.36 | 2.76E-08 | Paip1 | polyadenylate binding protein-interacting protein 1 |
| ENSMUSG00000086316 | 1.24 | 2.36 | 1.22E-06 | NA | NA |
| ENSMUSG00000058152 | 1.24 | 2.36 | 7.20E-05 | Chsy3 | chondroitin sulfate synthase 3 |
| ENSMUSG00000005267 | 1.24 | 2.36 | 1.03E-04 | Zfp287 | zinc finger protein 287 |
| ENSMUSG00000051578 | 1.24 | 2.36 | 2.65E-03 | NA | NA |
| ENSMUSG00000041773 | 1.24 | 2.36 | 1.36E-14 | Enc1 | ectodermal-neural cortex 1 |
| ENSMUSG00000038859 | 1.24 | 2.36 | 2.92E-03 | Baiap2l1 | BAI1-associated protein 2-like 1 |
| ENSMUSG00000075054 | 1.24 | 2.36 | 2.71E-08 | Yae1d1 | Yae1 domain containing 1 |
| ENSMUSG00000027173 | 1.24 | 2.36 | 1.48E-03 | Depdc7 | DEP domain containing 7 |
| ENSMUSG00000060227 | 1.24 | 2.36 | 9.02E-08 | Casc4 | cancer susceptibility candidate 4 |
| ENSMUSG00000030691 | 1.24 | 2.36 | 9.33E-18 | Fchsd2 | FCH and double SH3 domains 2 |
| ENSMUSG00000029068 | 1.24 | 2.36 | 1.80E-23 | Ccnl2 | cyclin L2 |
| ENSMUSG00000025324 | 1.24 | 2.36 | 1.56E-16 | Atp10a | ATPase, class V, type 10A |
| ENSMUSG00000045838 | 1.23 | 2.35 | 3.98E-09 | A430105I19Rik | RIKEN cDNA A430105I19 gene |
| ENSMUSG00000029569 | 1.23 | 2.35 | 1.93E-13 | Tmem168 | transmembrane protein 168 |
| ENSMUSG00000036764 | 1.23 | 2.35 | 3.23E-06 | Dnajc12 | DnaJ (Hsp40) homolog, subfamily C, member 12 |
| ENSMUSG00000063760 | 1.23 | 2.35 | 3.40E-05 | Rnf217 | ring finger protein 217 |
| ENSMUSG00000009035 | 1.23 | 2.35 | 2.77E-07 | Tmem184b | transmembrane protein 184b |
| ENSMUSG00000048047 | 1.23 | 2.35 | 8.02E-04 | Zbtb33 | zinc finger and BTB domain containing 33 |
| ENSMUSG00000073755 | 1.23 | 2.35 | 6.76E-08 | 5730409E04Rik | RIKEN cDNA 5730409E04Rik gene |
| ENSMUSG00000040265 | 1.23 | 2.35 | 8.39E-05 | Dnm3 | dynamin 3 |
| ENSMUSG00000021540 | 1.23 | 2.35 | 1.10E-09 | Smad5 | SMAD family member 5 |
| ENSMUSG00000026771 | 1.23 | 2.35 | 2.18E-06 | Spopl | speckle-type POZ protein-like |
| ENSMUSG00000035456 | 1.23 | 2.34 | 3.54E-03 | Prdm8 | PR domain containing 8 |
| ENSMUSG00000048592 | 1.23 | 2.34 | 2.22E-03 | NA | NA |
| ENSMUSG00000045896 | 1.23 | 2.34 | 2.18E-13 | Paip2b | poly(A) binding protein interacting protein 2B |
| ENSMUSG00000033543 | 1.23 | 2.34 | 4.60E-09 | Gtf2a2 | general transcription factor II A, 2 |
| ENSMUSG00000039887 | 1.23 | 2.34 | 8.62E-10 | Alg14 | asparagine-linked glycosylation 14 |
| ENSMUSG00000038172 | 1.23 | 2.34 | 3.14E-10 | Ttc39b | tetratricopeptide repeat domain 39B |
| ENSMUSG00000074001 | 1.23 | 2.34 | 7.71E-03 | Klhl40 | kelch-like 40 |
| ENSMUSG00000001521 | 1.23 | 2.34 | 1.57E-09 | Tulp3 | tubby-like protein 3 |
| ENSMUSG00000045205 | 1.23 | 2.34 | 7.50E-10 | Dpy19l4 | dpy-19-like 4 (C. elegans) |
| ENSMUSG00000038903 | 1.23 | 2.34 | 1.86E-03 | Ccdc68 | coiled-coil domain containing 68 |
| ENSMUSG00000032434 | 1.22 | 2.34 | 7.94E-19 | Cmtm6 | CKLF-like MARVEL transmembrane domain containing 6 |
| ENSMUSG00000020088 | 1.22 | 2.34 | 1.25E-06 | Sar1a | SAR1 gene homolog A (S. cerevisiae) |
| ENSMUSG00000057315 | 1.22 | 2.34 | 4.39E-09 | Arhgap24 | Rho GTPase activating protein 24 |
| ENSMUSG00000020070 | 1.22 | 2.34 | 6.18E-12 | Rufy2 | RUN and FYVE domain-containing 2 |
| ENSMUSG00000062101 | 1.22 | 2.34 | 4.53E-04 | Zfp119b | zinc finger protein 119b |
| ENSMUSG00000020321 | 1.22 | 2.34 | 3.78E-13 | Mdh1 | malate dehydrogenase 1, NAD (soluble) |
| ENSMUSG00000019494 | 1.22 | 2.33 | 1.89E-18 | Cops6 | COP9 (constitutive photomorphogenic) homolog, subunit 6 (Arabidopsis thaliana) |
| ENSMUSG00000075297 | 1.22 | 2.33 | 7.26E-04 | H60b | histocompatibility 60b |
| ENSMUSG00000032373 | 1.22 | 2.33 | 7.22E-03 | Car12 | carbonic anyhydrase 12 |
| ENSMUSG00000039304 | 1.22 | 2.33 | 5.00E-06 | Tnfsf10 | tumor necrosis factor (ligand) superfamily, member 10 |
| ENSMUSG00000015120 | 1.22 | 2.33 | 1.97E-13 | Ube2i | ubiquitin-conjugating enzyme E2I |
| ENSMUSG00000038227 | 1.22 | 2.33 | 3.40E-09 | Hoxa9 | homeobox A9 |
| ENSMUSG00000025978 | 1.22 | 2.33 | 1.70E-06 | Rftn2 | raftlin family member 2 |
| ENSMUSG00000045092 | 1.22 | 2.33 | 1.24E-08 | S1pr1 | sphingosine-1-phosphate receptor 1 |
| ENSMUSG00000049321 | 1.22 | 2.33 | 5.62E-05 | Zfp2 | zinc finger protein 2 |
| ENSMUSG00000029201 | 1.22 | 2.33 | 5.99E-11 | Ugdh | UDP-glucose dehydrogenase |
| ENSMUSG00000022215 | 1.22 | 2.33 | 7.38E-04 | Fitm1 | fat storage-inducing transmembrane protein 1 |
| ENSMUSG00000046768 | 1.22 | 2.33 | 5.03E-12 | Rhoj | ras homolog gene family, member J |
| ENSMUSG00000037259 | 1.22 | 2.33 | 4.83E-03 | Dzank1 | double zinc ribbon and ankyrin repeat domains 1 |
| ENSMUSG00000025607 | 1.22 | 2.33 | 9.98E-19 | Copg2 | coatomer protein complex, subunit gamma 2 |
| ENSMUSG00000027397 | 1.22 | 2.33 | 9.26E-19 | Slc20a1 | solute carrier family 20, member 1 |
| ENSMUSG00000026596 | 1.22 | 2.33 | 4.04E-13 | Abl2 | v-abl Abelson murine leukemia viral oncogene 2 (arg, Abelson-related gene) |
| ENSMUSG00000029994 | 1.22 | 2.33 | 5.41E-08 | Anxa4 | annexin A4 |
| ENSMUSG00000046618 | 1.22 | 2.33 | 4.61E-09 | Olfml2a | olfactomedin-like 2A |
| ENSMUSG00000048497 | 1.22 | 2.32 | 4.36E-05 | Mmgt2 | membrane magnesium transporter 2 |
| ENSMUSG00000044405 | 1.22 | 2.32 | 9.67E-03 | Adig | adipogenin |
| ENSMUSG00000029312 | 1.22 | 2.32 | 9.74E-08 | Klhl8 | kelch-like 8 |
| ENSMUSG00000072825 | 1.22 | 2.32 | 1.64E-06 | Cep170b | centrosomal protein 170B |
| ENSMUSG00000031286 | 1.22 | 2.32 | 1.03E-04 | Glt28d2 | glycosyltransferase 28 domain containing 2 |
| ENSMUSG00000053390 | 1.21 | 2.32 | 5.75E-09 | Zfp952 | zinc finger protein 952 |
| ENSMUSG00000030223 | 1.21 | 2.32 | 2.72E-08 | Ptpro | protein tyrosine phosphatase, receptor type, O |
| ENSMUSG00000020152 | 1.21 | 2.32 | 3.38E-30 | Actr2 | ARP2 actin-related protein 2 |
| ENSMUSG00000024778 | 1.21 | 2.32 | 4.17E-10 | Fas | Fas (TNF receptor superfamily member 6) |
| ENSMUSG00000029642 | 1.21 | 2.32 | 4.43E-05 | Polr1d | polymerase (RNA) I polypeptide D |
| ENSMUSG00000081094 | 1.21 | 2.32 | 1.13E-09 | NA | NA |
| ENSMUSG00000051339 | 1.21 | 2.32 | 4.20E-07 | 2900026A02Rik | RIKEN cDNA 2900026A02 gene |
| ENSMUSG00000022906 | 1.21 | 2.32 | 3.77E-22 | Parp9 | poly (ADP-ribose) polymerase family, member 9 |
| ENSMUSG00000044501 | 1.21 | 2.32 | 9.86E-04 | Zfp758 | zinc finger protein 758 |
| ENSMUSG00000023068 | 1.21 | 2.31 | 1.92E-09 | Nus1 | nuclear undecaprenyl pyrophosphate synthase 1 homolog (S. cerevisiae) |
| ENSMUSG00000000154 | 1.21 | 2.31 | 7.35E-04 | Slc22a18 | solute carrier family 22 (organic cation transporter), member 18 |
| ENSMUSG00000026740 | 1.21 | 2.31 | 2.39E-07 | Dnajc1 | DnaJ (Hsp40) homolog, subfamily C, member 1 |
| ENSMUSG00000029134 | 1.21 | 2.31 | 4.25E-03 | Plb1 | phospholipase B1 |
| ENSMUSG00000026047 | 1.21 | 2.31 | 7.64E-14 | Kdelc1 | KDEL (Lys-Asp-Glu-Leu) containing 1 |
| ENSMUSG00000028232 | 1.21 | 2.31 | 4.88E-11 | Tmem68 | transmembrane protein 68 |
| ENSMUSG00000021898 | 1.21 | 2.31 | 4.56E-04 | Asb14 | ankyrin repeat and SOCS box-containing 14 |
| ENSMUSG00000040033 | 1.21 | 2.31 | 2.81E-29 | Stat2 | signal transducer and activator of transcription 2 |
| ENSMUSG00000031808 | 1.21 | 2.31 | 2.85E-08 | Slc27a1 | solute carrier family 27 (fatty acid transporter), member 1 |
| ENSMUSG00000024695 | 1.21 | 2.31 | 1.93E-07 | Zfp91 | zinc finger protein 91 |
| ENSMUSG00000049076 | 1.21 | 2.31 | 1.41E-09 | Acap2 | ArfGAP with coiled-coil, ankyrin repeat and PH domains 2 |
| ENSMUSG00000031548 | 1.21 | 2.31 | 7.27E-05 | Sfrp1 | secreted frizzled-related protein 1 |
| ENSMUSG00000078238 | 1.21 | 2.31 | 1.06E-06 | NA | NA |
| ENSMUSG00000044086 | 1.20 | 2.31 | 6.50E-04 | Lmod3 | leiomodin 3 (fetal) |
| ENSMUSG00000033880 | 1.20 | 2.31 | 2.57E-14 | Lgals3bp | lectin, galactoside-binding, soluble, 3 binding protein |
| ENSMUSG00000038679 | 1.20 | 2.30 | 1.20E-03 | Trps1 | trichorhinophalangeal syndrome I (human) |
| ENSMUSG00000028273 | 1.20 | 2.30 | 4.87E-07 | Pdlim5 | PDZ and LIM domain 5 |
| ENSMUSG00000039153 | 1.20 | 2.30 | 1.23E-08 | Runx2 | runt related transcription factor 2 |
| ENSMUSG00000058402 | 1.20 | 2.30 | 1.88E-08 | Zfp420 | zinc finger protein 420 |
| ENSMUSG00000018900 | 1.20 | 2.30 | 1.71E-11 | Slc22a5 | solute carrier family 22 (organic cation transporter), member 5 |
| ENSMUSG00000026623 | 1.20 | 2.30 | 2.52E-07 | Lpgat1 | lysophosphatidylglycerol acyltransferase 1 |
| ENSMUSG00000036916 | 1.20 | 2.30 | 6.67E-04 | Zfp280c | zinc finger protein 280C |
| ENSMUSG00000038127 | 1.20 | 2.30 | 7.16E-09 | Ccdc50 | coiled-coil domain containing 50 |
| ENSMUSG00000068396 | 1.20 | 2.30 | 1.12E-03 | NA | NA |
| ENSMUSG00000042918 | 1.20 | 2.30 | 1.55E-03 | Mamstr | MEF2 activating motif and SAP domain containing transcriptional regulator |
| ENSMUSG00000024381 | 1.20 | 2.30 | 5.23E-07 | Bin1 | bridging integrator 1 |
| ENSMUSG00000032051 | 1.20 | 2.30 | 2.06E-08 | Fdx1 | ferredoxin 1 |
| ENSMUSG00000016206 | 1.20 | 2.30 | 4.35E-08 | H2-M3 | histocompatibility 2, M region locus 3 |
| ENSMUSG00000043079 | 1.20 | 2.30 | 2.97E-07 | Synpo | synaptopodin |
| ENSMUSG00000074797 | 1.20 | 2.30 | 2.35E-05 | Itpa | inosine triphosphatase (nucleoside triphosphate pyrophosphatase) |
| ENSMUSG00000026696 | 1.20 | 2.29 | 5.16E-11 | Vamp4 | vesicle-associated membrane protein 4 |
| ENSMUSG00000019806 | 1.20 | 2.29 | 4.22E-06 | Aig1 | androgen-induced 1 |
| ENSMUSG00000025089 | 1.20 | 2.29 | 7.27E-06 | Gfra1 | glial cell line derived neurotrophic factor family receptor alpha 1 |
| ENSMUSG00000037119 | 1.20 | 2.29 | 1.01E-05 | D15Ertd621e | DNA segment, Chr 15, ERATO Doi 621, expressed |
| ENSMUSG00000074170 | 1.20 | 2.29 | 5.13E-14 | Plekhf1 | pleckstrin homology domain containing, family F (with FYVE domain) member 1 |
| ENSMUSG00000038540 | 1.20 | 2.29 | 3.27E-03 | Tmc3 | transmembrane channel-like gene family 3 |
| ENSMUSG00000028550 | 1.20 | 2.29 | 1.18E-06 | Atg4c | autophagy related 4C, cysteine peptidase |
| ENSMUSG00000045435 | 1.20 | 2.29 | 9.93E-05 | Tmem60 | transmembrane protein 60 |
| ENSMUSG00000025278 | 1.20 | 2.29 | 1.01E-08 | Flnb | filamin, beta |
| ENSMUSG00000039759 | 1.20 | 2.29 | 1.75E-08 | Thap3 | THAP domain containing, apoptosis associated protein 3 |
| ENSMUSG00000024234 | 1.19 | 2.29 | 1.04E-07 | Mtpap | mitochondrial poly(A) polymerase |
| ENSMUSG00000024173 | 1.19 | 2.29 | 9.47E-03 | Tpsab1 | tryptase alpha/beta 1 |
| ENSMUSG00000049439 | 1.19 | 2.29 | 4.70E-10 | Cyp20a1 | cytochrome P450, family 20, subfamily a, polypeptide 1 |
| ENSMUSG00000032089 | 1.19 | 2.29 | 2.27E-06 | Il10ra | interleukin 10 receptor, alpha |
| ENSMUSG00000034659 | 1.19 | 2.29 | 1.00E-06 | Tmem109 | transmembrane protein 109 |
| ENSMUSG00000054793 | 1.19 | 2.28 | 1.00E-03 | Cadm4 | cell adhesion molecule 4 |
| ENSMUSG00000057858 | 1.19 | 2.28 | 1.61E-06 | D19Ertd737e | DNA segment, Chr 19, ERATO Doi 737, expressed |
| ENSMUSG00000052144 | 1.19 | 2.28 | 7.41E-10 | Ppp4r2 | protein phosphatase 4, regulatory subunit 2 |
| ENSMUSG00000082076 | 1.19 | 2.28 | 5.90E-04 | NA | NA |
| ENSMUSG00000002985 | 1.19 | 2.28 | 1.01E-05 | Apoe | apolipoprotein E |
| ENSMUSG00000031309 | 1.19 | 2.28 | 5.65E-08 | Rps6ka3 | ribosomal protein S6 kinase polypeptide 3 |
| ENSMUSG00000052914 | 1.19 | 2.28 | 3.93E-08 | Cyp2j6 | cytochrome P450, family 2, subfamily j, polypeptide 6 |
| ENSMUSG00000053253 | 1.19 | 2.28 | 1.22E-13 | Ndfip2 | Nedd4 family interacting protein 2 |
| ENSMUSG00000053051 | 1.19 | 2.28 | 3.01E-07 | NA | NA |
| ENSMUSG00000079184 | 1.19 | 2.28 | 1.70E-13 | Mphosph8 | M-phase phosphoprotein 8 |
| ENSMUSG00000026770 | 1.19 | 2.28 | 5.33E-04 | Il2ra | interleukin 2 receptor, alpha chain |
| ENSMUSG00000043391 | 1.19 | 2.28 | 8.12E-08 | 2510009E07Rik | RIKEN cDNA 2510009E07 gene |
| ENSMUSG00000028525 | 1.19 | 2.28 | 8.55E-06 | Pde4b | phosphodiesterase 4B, cAMP specific |
| ENSMUSG00000073725 | 1.19 | 2.28 | 6.99E-22 | Lmbrd1 | LMBR1 domain containing 1 |
| ENSMUSG00000046892 | 1.19 | 2.28 | 2.99E-03 | NA | NA |
| ENSMUSG00000040836 | 1.19 | 2.28 | 3.24E-04 | Gpr161 | G protein-coupled receptor 161 |
| ENSMUSG00000021240 | 1.19 | 2.27 | 5.52E-11 | Abcd4 | ATP-binding cassette, sub-family D (ALD), member 4 |
| ENSMUSG00000057777 | 1.19 | 2.27 | 4.17E-04 | Mab21l2 | mab-21-like 2 (C. elegans) |
| ENSMUSG00000053702 | 1.19 | 2.27 | 1.62E-03 | Nebl | nebulette |
| ENSMUSG00000023092 | 1.18 | 2.27 | 7.50E-06 | Fhl1 | four and a half LIM domains 1 |
| ENSMUSG00000033460 | 1.18 | 2.27 | 2.54E-07 | Armcx1 | armadillo repeat containing, X-linked 1 |
| ENSMUSG00000026464 | 1.18 | 2.27 | 1.92E-12 | Zc3h11a | zinc finger CCCH type containing 11A |
| ENSMUSG00000026077 | 1.18 | 2.27 | 1.62E-05 | Npas2 | neuronal PAS domain protein 2 |
| ENSMUSG00000015804 | 1.18 | 2.27 | 1.17E-09 | Med28 | mediator complex subunit 28 |
| ENSMUSG00000014609 | 1.18 | 2.27 | 2.83E-03 | Chrne | cholinergic receptor, nicotinic, epsilon polypeptide |
| ENSMUSG00000032440 | 1.18 | 2.27 | 3.28E-12 | Tgfbr2 | transforming growth factor, beta receptor II |
| ENSMUSG00000017778 | 1.18 | 2.27 | 1.94E-08 | Cox7c | cytochrome c oxidase subunit VIIc |
| ENSMUSG00000067365 | 1.18 | 2.27 | 1.12E-14 | Tmem128 | transmembrane protein 128 |
| ENSMUSG00000031590 | 1.18 | 2.27 | 4.29E-07 | Frg1 | FSHD region gene 1 |
| ENSMUSG00000027313 | 1.18 | 2.27 | 2.06E-03 | Chac1 | ChaC, cation transport regulator 1 |
| ENSMUSG00000025037 | 1.18 | 2.26 | 3.28E-06 | Maoa | monoamine oxidase A |
| ENSMUSG00000028557 | 1.18 | 2.26 | 8.72E-10 | Rnf11 | ring finger protein 11 |
| ENSMUSG00000025185 | 1.18 | 2.26 | 4.98E-04 | Loxl4 | lysyl oxidase-like 4 |
| ENSMUSG00000035109 | 1.18 | 2.26 | 1.76E-04 | Shc4 | SHC (Src homology 2 domain containing) family, member 4 |
| ENSMUSG00000000088 | 1.18 | 2.26 | 1.76E-05 | Cox5a | cytochrome c oxidase subunit Va |
| ENSMUSG00000004455 | 1.18 | 2.26 | 1.14E-04 | Ppp1cc | protein phosphatase 1, catalytic subunit, gamma isoform |
| ENSMUSG00000048752 | 1.18 | 2.26 | 6.41E-04 | Prss50 | protease, serine 50 |
| ENSMUSG00000001700 | 1.18 | 2.26 | 2.40E-08 | Gramd3 | GRAM domain containing 3 |
| ENSMUSG00000030323 | 1.18 | 2.26 | 3.96E-09 | Ift122 | intraflagellar transport 122 |
| ENSMUSG00000041361 | 1.18 | 2.26 | 5.61E-05 | Myzap | myocardial zonula adherens protein |
| ENSMUSG00000009470 | 1.18 | 2.26 | 7.66E-16 | Tnpo1 | transportin 1 |
| ENSMUSG00000039982 | 1.18 | 2.26 | 6.44E-17 | Dtx4 | deltex 4 homolog (Drosophila) |
| ENSMUSG00000084020 | 1.18 | 2.26 | 2.59E-05 | NA | NA |
| ENSMUSG00000027663 | 1.18 | 2.26 | 1.75E-08 | Zmat3 | zinc finger matrin type 3 |
| ENSMUSG00000029759 | 1.18 | 2.26 | 2.14E-10 | Pon3 | paraoxonase 3 |
| ENSMUSG00000068134 | 1.18 | 2.26 | 1.43E-08 | Zfp120 | zinc finger protein 120 |
| ENSMUSG00000020986 | 1.17 | 2.26 | 2.72E-18 | Sec23a | SEC23A (S. cerevisiae) |
| ENSMUSG00000042766 | 1.17 | 2.26 | 1.44E-04 | Trim46 | tripartite motif-containing 46 |
| ENSMUSG00000033295 | 1.17 | 2.26 | 4.14E-06 | Ptprf | protein tyrosine phosphatase, receptor type, F |
| ENSMUSG00000062545 | 1.17 | 2.26 | 1.56E-04 | Tlr12 | toll-like receptor 12 |
| ENSMUSG00000066613 | 1.17 | 2.26 | 4.76E-05 | Zfp932 | zinc finger protein 932 |
| ENSMUSG00000021171 | 1.17 | 2.26 | 8.46E-09 | Esyt2 | extended synaptotagmin-like protein 2 |
| ENSMUSG00000015656 | 1.17 | 2.25 | 3.92E-03 | Hspa8 | heat shock protein 8 |
| ENSMUSG00000028527 | 1.17 | 2.25 | 7.58E-05 | Ak4 | adenylate kinase 4 |
| ENSMUSG00000014786 | 1.17 | 2.25 | 7.37E-13 | Slc9a5 | solute carrier family 9 (sodium/hydrogen exchanger), member 5 |
| ENSMUSG00000029234 | 1.17 | 2.25 | 1.17E-10 | Tmem165 | transmembrane protein 165 |
| ENSMUSG00000048915 | 1.17 | 2.25 | 1.46E-04 | Efna5 | ephrin A5 |
| ENSMUSG00000051146 | 1.17 | 2.25 | 2.89E-03 | Camk2n2 | calcium/calmodulin-dependent protein kinase II inhibitor 2 |
| ENSMUSG00000040760 | 1.17 | 2.25 | 1.50E-08 | Appl1 | adaptor protein, phosphotyrosine interaction, PH domain and leucine zipper containing 1 |
| ENSMUSG00000033855 | 1.17 | 2.25 | 9.00E-05 | Ston1 | stonin 1 |
| ENSMUSG00000003847 | 1.17 | 2.25 | 5.86E-15 | Nfat5 | nuclear factor of activated T cells 5 |
| ENSMUSG00000061451 | 1.17 | 2.24 | 4.97E-03 | Tmem151a | transmembrane protein 151A |
| ENSMUSG00000081967 | 1.17 | 2.24 | 2.00E-05 | NA | NA |
| ENSMUSG00000054414 | 1.17 | 2.24 | 2.00E-10 | Slc30a7 | solute carrier family 30 (zinc transporter), member 7 |
| ENSMUSG00000033813 | 1.17 | 2.24 | 8.77E-07 | Tcea1 | transcription elongation factor A (SII) 1 |
| ENSMUSG00000023913 | 1.17 | 2.24 | 4.99E-09 | Pla2g7 | phospholipase A2, group VII (platelet-activating factor acetylhydrolase, plasma) |
| ENSMUSG00000083678 | 1.16 | 2.24 | 3.45E-08 | NA | NA |
| ENSMUSG00000019948 | 1.16 | 2.24 | 7.50E-08 | Actr6 | ARP6 actin-related protein 6 |
| ENSMUSG00000049791 | 1.16 | 2.24 | 9.40E-03 | Fzd4 | frizzled homolog 4 (Drosophila) |
| ENSMUSG00000031266 | 1.16 | 2.24 | 1.82E-07 | Gla | galactosidase, alpha |
| ENSMUSG00000019877 | 1.16 | 2.24 | 1.01E-10 | Serinc1 | serine incorporator 1 |
| ENSMUSG00000024621 | 1.16 | 2.24 | 9.32E-12 | Csf1r | colony stimulating factor 1 receptor |
| ENSMUSG00000041959 | 1.16 | 2.24 | 1.48E-05 | S100a10 | S100 calcium binding protein A10 (calpactin) |
| ENSMUSG00000085939 | 1.16 | 2.24 | 2.99E-07 | NA | NA |
| ENSMUSG00000026782 | 1.16 | 2.24 | 2.97E-06 | Abi2 | abl-interactor 2 |
| ENSMUSG00000051256 | 1.16 | 2.24 | 1.32E-07 | Jagn1 | jagunal homolog 1 (Drosophila) |
| ENSMUSG00000001270 | 1.16 | 2.23 | 2.69E-04 | Ckb | creatine kinase, brain |
| ENSMUSG00000020949 | 1.16 | 2.23 | 4.12E-04 | Fkbp3 | FK506 binding protein 3 |
| ENSMUSG00000036636 | 1.16 | 2.23 | 4.54E-14 | Clcn7 | chloride channel 7 |
| ENSMUSG00000028601 | 1.16 | 2.23 | 5.52E-06 | Echdc2 | enoyl Coenzyme A hydratase domain containing 2 |
| ENSMUSG00000033006 | 1.16 | 2.23 | 9.30E-03 | Sox10 | SRY (sex determining region Y)-box 10 |
| ENSMUSG00000051675 | 1.16 | 2.23 | 1.38E-13 | Trim32 | tripartite motif-containing 32 |
| ENSMUSG00000027340 | 1.16 | 2.23 | 1.26E-07 | Slc23a2 | solute carrier family 23 (nucleobase transporters), member 2 |
| ENSMUSG00000018500 | 1.16 | 2.23 | 2.57E-03 | Adora2b | adenosine A2b receptor |
| ENSMUSG00000062963 | 1.16 | 2.23 | 5.06E-09 | Ufc1 | ubiquitin-fold modifier conjugating enzyme 1 |
| ENSMUSG00000031709 | 1.16 | 2.23 | 2.87E-11 | Tbc1d9 | TBC1 domain family, member 9 |
| ENSMUSG00000041153 | 1.15 | 2.23 | 1.09E-07 | Osgin2 | oxidative stress induced growth inhibitor family member 2 |
| ENSMUSG00000030725 | 1.15 | 2.23 | 3.03E-06 | Lipt2 | lipoyl(octanoyl) transferase 2 (putative) |
| ENSMUSG00000038780 | 1.15 | 2.22 | 1.44E-18 | Smurf1 | SMAD specific E3 ubiquitin protein ligase 1 |
| ENSMUSG00000075702 | 1.15 | 2.22 | 1.60E-07 | Selm | selenoprotein M |
| ENSMUSG00000053477 | 1.15 | 2.22 | 1.35E-08 | Tcf4 | transcription factor 4 |
| ENSMUSG00000048537 | 1.15 | 2.22 | 1.66E-06 | Phldb1 | pleckstrin homology-like domain, family B, member 1 |
| ENSMUSG00000037552 | 1.15 | 2.22 | 6.29E-16 | Plekhg2 | pleckstrin homology domain containing, family G (with RhoGef domain) member 2 |
| ENSMUSG00000040093 | 1.15 | 2.22 | 5.23E-07 | Bmf | BCL2 modifying factor |
| ENSMUSG00000034037 | 1.15 | 2.22 | 6.95E-07 | Fgd5 | FYVE, RhoGEF and PH domain containing 5 |
| ENSMUSG00000032086 | 1.15 | 2.22 | 5.34E-08 | Bace1 | beta-site APP cleaving enzyme 1 |
| ENSMUSG00000047368 | 1.15 | 2.22 | 6.23E-07 | Abhd17b | abhydrolase domain containing 17B |
| ENSMUSG00000034848 | 1.15 | 2.22 | 2.12E-06 | Ttc21b | tetratricopeptide repeat domain 21B |
| ENSMUSG00000056515 | 1.15 | 2.22 | 2.09E-12 | Rab31 | RAB31, member RAS oncogene family |
| ENSMUSG00000078349 | 1.15 | 2.22 | 2.68E-06 | NA | NA |
| ENSMUSG00000060681 | 1.15 | 2.22 | 1.44E-07 | Slc9a6 | solute carrier family 9 (sodium/hydrogen exchanger), member 6 |
| ENSMUSG00000030188 | 1.15 | 2.22 | 6.06E-05 | Magohb | mago-nashi homolog B (Drosophila) |
| ENSMUSG00000017291 | 1.15 | 2.22 | 7.11E-06 | Taok1 | TAO kinase 1 |
| ENSMUSG00000002504 | 1.15 | 2.22 | 7.47E-09 | Slc9a3r2 | solute carrier family 9 (sodium/hydrogen exchanger), member 3 regulator 2 |
| ENSMUSG00000019659 | 1.15 | 2.21 | 8.36E-05 | Ccdc12 | coiled-coil domain containing 12 |
| ENSMUSG00000071793 | 1.15 | 2.21 | 2.70E-04 | 2610005L07Rik | cadherin 11 pseudogene |
| ENSMUSG00000048164 | 1.15 | 2.21 | 1.39E-05 | NA | NA |
| ENSMUSG00000085622 | 1.15 | 2.21 | 4.22E-04 | 3110056K07Rik | RIKEN cDNA 3110056K07 gene |
| ENSMUSG00000032841 | 1.15 | 2.21 | 9.25E-05 | Prr5l | proline rich 5 like |
| ENSMUSG00000023036 | 1.15 | 2.21 | 1.88E-07 | NA | NA |
| ENSMUSG00000022032 | 1.14 | 2.21 | 5.54E-06 | Scara5 | scavenger receptor class A, member 5 (putative) |
| ENSMUSG00000034863 | 1.14 | 2.21 | 5.38E-05 | Ano8 | anoctamin 8 |
| ENSMUSG00000030170 | 1.14 | 2.21 | 6.51E-06 | Wnt5b | wingless-related MMTV integration site 5B |
| ENSMUSG00000038777 | 1.14 | 2.21 | 5.25E-03 | Sema6c | sema domain, transmembrane domain (TM), and cytoplasmic domain, (semaphorin) 6C |
| ENSMUSG00000025920 | 1.14 | 2.21 | 9.19E-08 | Stau2 | staufen (RNA binding protein) homolog 2 (Drosophila) |
| ENSMUSG00000030647 | 1.14 | 2.21 | 6.95E-05 | Ndufc2 | NADH dehydrogenase (ubiquinone) 1, subcomplex unknown, 2 |
| ENSMUSG00000019978 | 1.14 | 2.21 | 8.81E-07 | Epb4.1l2 | erythrocyte protein band 4.1-like 2 |
| ENSMUSG00000068457 | 1.14 | 2.21 | 1.03E-05 | Uty | ubiquitously transcribed tetratricopeptide repeat gene, Y chromosome |
| ENSMUSG00000000605 | 1.14 | 2.21 | 7.81E-15 | Clcn4-2 | chloride channel 4-2 |
| ENSMUSG00000043154 | 1.14 | 2.21 | 3.08E-04 | Ppp2r3a | protein phosphatase 2, regulatory subunit B'', alpha |
| ENSMUSG00000057606 | 1.14 | 2.21 | 5.80E-04 | Colq | collagen-like tail subunit (single strand of homotrimer) of asymmetric acetylcholinesterase |
| ENSMUSG00000069045 | 1.14 | 2.20 | 7.24E-04 | Ddx3y | DEAD (Asp-Glu-Ala-Asp) box polypeptide 3, Y-linked |
| ENSMUSG00000078135 | 1.14 | 2.20 | 3.00E-04 | NA | NA |
| ENSMUSG00000050786 | 1.14 | 2.20 | 2.33E-07 | Ccdc126 | coiled-coil domain containing 126 |
| ENSMUSG00000032479 | 1.14 | 2.20 | 4.87E-08 | Map4 | microtubule-associated protein 4 |
| ENSMUSG00000081281 | 1.14 | 2.20 | 5.43E-06 | NA | NA |
| ENSMUSG00000022024 | 1.14 | 2.20 | 1.66E-08 | Sugt1 | SGT1, suppressor of G2 allele of SKP1 (S. cerevisiae) |
| ENSMUSG00000032198 | 1.14 | 2.20 | 5.72E-11 | Dock6 | dedicator of cytokinesis 6 |
| ENSMUSG00000054256 | 1.14 | 2.20 | 3.60E-05 | Msi1 | musashi RNA-binding protein 1 |
| ENSMUSG00000019796 | 1.14 | 2.20 | 4.50E-04 | Lrp11 | low density lipoprotein receptor-related protein 11 |
| ENSMUSG00000026986 | 1.14 | 2.20 | 3.32E-05 | Hnmt | histamine N-methyltransferase |
| ENSMUSG00000027963 | 1.14 | 2.20 | 3.63E-08 | Extl2 | exostoses (multiple)-like 2 |
| ENSMUSG00000021770 | 1.14 | 2.20 | 4.46E-06 | Samd8 | sterile alpha motif domain containing 8 |
| ENSMUSG00000045657 | 1.14 | 2.20 | 1.01E-05 | Pcdhb10 | protocadherin beta 10 |
| ENSMUSG00000001376 | 1.14 | 2.20 | 8.89E-05 | Ccdc132 | coiled-coil domain containing 132 |
| ENSMUSG00000038253 | 1.14 | 2.20 | 3.90E-03 | Hoxa5 | homeobox A5 |
| ENSMUSG00000006205 | 1.14 | 2.20 | 8.55E-06 | Htra1 | HtrA serine peptidase 1 |
| ENSMUSG00000015243 | 1.13 | 2.20 | 9.64E-05 | Abca1 | ATP-binding cassette, sub-family A (ABC1), member 1 |
| ENSMUSG00000055865 | 1.13 | 2.20 | 1.38E-03 | Fam19a3 | family with sequence similarity 19, member A3 |
| ENSMUSG00000020841 | 1.13 | 2.19 | 2.92E-07 | Cpd | carboxypeptidase D |
| ENSMUSG00000007867 | 1.13 | 2.19 | 2.73E-05 | Ift43 | intraflagellar transport 43 homolog (Chlamydomonas) |
| ENSMUSG00000079100 | 1.13 | 2.19 | 3.64E-10 | NA | NA |
| ENSMUSG00000062960 | 1.13 | 2.19 | 2.58E-06 | Kdr | kinase insert domain protein receptor |
| ENSMUSG00000043419 | 1.13 | 2.19 | 1.63E-03 | Chd3os | chromodomain helicase DNA binding protein 3, opposite strand |
| ENSMUSG00000000278 | 1.13 | 2.19 | 3.36E-11 | Scpep1 | serine carboxypeptidase 1 |
| ENSMUSG00000028883 | 1.13 | 2.19 | 3.35E-06 | Sema3a | sema domain, immunoglobulin domain (Ig), short basic domain, secreted, (semaphorin) 3A |
| ENSMUSG00000030057 | 1.13 | 2.19 | 2.17E-09 | Cnbp | cellular nucleic acid binding protein |
| ENSMUSG00000016946 | 1.13 | 2.19 | 7.85E-17 | Kctd5 | potassium channel tetramerisation domain containing 5 |
| ENSMUSG00000030122 | 1.13 | 2.19 | 2.31E-05 | Ptms | parathymosin |
| ENSMUSG00000058806 | 1.13 | 2.19 | 2.47E-03 | Col13a1 | collagen, type XIII, alpha 1 |
| ENSMUSG00000031137 | 1.13 | 2.19 | 1.08E-03 | Fgf13 | fibroblast growth factor 13 |
| ENSMUSG00000042810 | 1.13 | 2.19 | 4.99E-07 | Krba1 | KRAB-A domain containing 1 |
| ENSMUSG00000025903 | 1.13 | 2.19 | 1.38E-06 | Lypla1 | lysophospholipase 1 |
| ENSMUSG00000050234 | 1.13 | 2.19 | 1.51E-05 | Gja4 | gap junction protein, alpha 4 |
| ENSMUSG00000026896 | 1.13 | 2.19 | 6.01E-07 | Ifih1 | interferon induced with helicase C domain 1 |
| ENSMUSG00000043556 | 1.13 | 2.19 | 4.12E-05 | Fbxl7 | F-box and leucine-rich repeat protein 7 |
| ENSMUSG00000029605 | 1.13 | 2.19 | 2.15E-05 | Oas1b | 2'-5' oligoadenylate synthetase 1B |
| ENSMUSG00000039683 | 1.13 | 2.19 | 4.04E-03 | Sdk1 | sidekick homolog 1 (chicken) |
| ENSMUSG00000022758 | 1.13 | 2.18 | 2.81E-03 | P2rx6 | purinergic receptor P2X, ligand-gated ion channel, 6 |
| ENSMUSG00000060380 | 1.13 | 2.18 | 1.63E-04 | NA | NA |
| ENSMUSG00000073859 | 1.13 | 2.18 | 8.09E-03 | NA | NA |
| ENSMUSG00000023830 | 1.13 | 2.18 | 3.23E-06 | Igf2r | insulin-like growth factor 2 receptor |
| ENSMUSG00000060019 | 1.13 | 2.18 | 7.88E-04 | NA | NA |
| ENSMUSG00000028763 | 1.13 | 2.18 | 7.56E-05 | Hspg2 | perlecan (heparan sulfate proteoglycan 2) |
| ENSMUSG00000020829 | 1.13 | 2.18 | 4.11E-04 | Slc46a1 | solute carrier family 46, member 1 |
| ENSMUSG00000025407 | 1.13 | 2.18 | 2.19E-03 | Gli1 | GLI-Kruppel family member GLI1 |
| ENSMUSG00000029641 | 1.12 | 2.18 | 2.70E-03 | Rasl11a | RAS-like, family 11, member A |
| ENSMUSG00000051355 | 1.12 | 2.18 | 2.97E-09 | Commd1 | COMM domain containing 1 |
| ENSMUSG00000044645 | 1.12 | 2.18 | 1.79E-05 | Gm7334 | B-cell translocation gene 3 pseudogene |
| ENSMUSG00000046470 | 1.12 | 2.18 | 6.02E-04 | Sox18 | SRY (sex determining region Y)-box 18 |
| ENSMUSG00000019880 | 1.12 | 2.18 | 1.86E-04 | Rspo3 | R-spondin 3 homolog (Xenopus laevis) |
| ENSMUSG00000042814 | 1.12 | 2.18 | 3.23E-05 | Mcts2 | malignant T cell amplified sequence 2 |
| ENSMUSG00000090015 | 1.12 | 2.18 | 2.69E-03 | Gm15446 | predicted gene 15446 |
| ENSMUSG00000001774 | 1.12 | 2.18 | 7.14E-10 | Chordc1 | cysteine and histidine-rich domain (CHORD)-containing, zinc-binding protein 1 |
| ENSMUSG00000024349 | 1.12 | 2.18 | 7.45E-10 | Tmem173 | transmembrane protein 173 |
| ENSMUSG00000072889 | 1.12 | 2.17 | 3.23E-11 | Nfxl1 | nuclear transcription factor, X-box binding-like 1 |
| ENSMUSG00000074867 | 1.12 | 2.17 | 4.49E-04 | Zfp808 | zinc finger protein 80 |
| ENSMUSG00000027365 | 1.12 | 2.17 | 4.42E-08 | Trpm7 | transient receptor potential cation channel, subfamily M, member 7 |
| ENSMUSG00000063273 | 1.12 | 2.17 | 1.22E-13 | Naa15 | N(alpha)-acetyltransferase 15, NatA auxiliary subunit |
| ENSMUSG00000030555 | 1.12 | 2.17 | 1.11E-07 | Ttc23 | tetratricopeptide repeat domain 23 |
| ENSMUSG00000001891 | 1.12 | 2.17 | 2.32E-04 | Ugp2 | UDP-glucose pyrophosphorylase 2 |
| ENSMUSG00000026211 | 1.12 | 2.17 | 3.35E-05 | Obsl1 | obscurin-like 1 |
| ENSMUSG00000027534 | 1.12 | 2.17 | 1.35E-04 | Snx16 | sorting nexin 16 |
| ENSMUSG00000028603 | 1.12 | 2.17 | 1.69E-09 | Scp2 | sterol carrier protein 2, liver |
| ENSMUSG00000019929 | 1.12 | 2.17 | 3.28E-04 | Dcn | decorin |
| ENSMUSG00000040612 | 1.12 | 2.17 | 2.07E-05 | Ildr2 | immunoglobulin-like domain containing receptor 2 |
| ENSMUSG00000000958 | 1.12 | 2.17 | 7.03E-07 | Slc7a7 | solute carrier family 7 (cationic amino acid transporter, y+ system), member 7 |
| ENSMUSG00000019326 | 1.11 | 2.17 | 4.79E-04 | Aoc3 | amine oxidase, copper containing 3 |
| ENSMUSG00000027248 | 1.11 | 2.17 | 2.44E-05 | Pdia3 | protein disulfide isomerase associated 3 |
| ENSMUSG00000063316 | 1.11 | 2.17 | 3.58E-04 | NA | NA |
| ENSMUSG00000015575 | 1.11 | 2.17 | 5.77E-08 | Atp6v0e | ATPase, H+ transporting, lysosomal V0 subunit E |
| ENSMUSG00000024247 | 1.11 | 2.17 | 4.13E-04 | Pkdcc | protein kinase domain containing, cytoplasmic |
| ENSMUSG00000039976 | 1.11 | 2.16 | 1.47E-05 | Tbc1d16 | TBC1 domain family, member 16 |
| ENSMUSG00000051341 | 1.11 | 2.16 | 5.47E-07 | Zfp52 | zinc finger protein 52 |
| ENSMUSG00000031486 | 1.11 | 2.16 | 5.41E-06 | Gpr124 | G protein-coupled receptor 124 |
| ENSMUSG00000024486 | 1.11 | 2.16 | 2.77E-04 | Hbegf | heparin-binding EGF-like growth factor |
| ENSMUSG00000027309 | 1.11 | 2.16 | 7.09E-10 | 4930402H24Rik | RIKEN cDNA 4930402H24 gene |
| ENSMUSG00000047343 | 1.11 | 2.16 | 8.18E-03 | Mettl21c | methyltransferase like 21C |
| ENSMUSG00000079283 | 1.11 | 2.16 | 4.97E-04 | 2310009B15Rik | RIKEN cDNA 2310009B15 gene |
| ENSMUSG00000003752 | 1.11 | 2.16 | 1.46E-06 | Itpkc | inositol 1,4,5-trisphosphate 3-kinase C |
| ENSMUSG00000025616 | 1.11 | 2.16 | 1.23E-06 | Usp16 | ubiquitin specific peptidase 16 |
| ENSMUSG00000059839 | 1.11 | 2.16 | 5.65E-05 | Zfp874b | zinc finger protein 874b |
| ENSMUSG00000008305 | 1.11 | 2.16 | 3.64E-07 | Tle1 | transducin-like enhancer of split 1, homolog of Drosophila E(spl) |
| ENSMUSG00000066842 | 1.11 | 2.16 | 6.25E-03 | Hmcn1 | hemicentin 1 |
| ENSMUSG00000053931 | 1.11 | 2.16 | 1.20E-07 | Cnn3 | calponin 3, acidic |
| ENSMUSG00000047260 | 1.11 | 2.16 | 6.27E-05 | Emc6 | ER membrane protein complex subunit 6 |
| ENSMUSG00000022270 | 1.11 | 2.16 | 2.29E-04 | Fam134b | family with sequence similarity 134, member B |
| ENSMUSG00000015305 | 1.11 | 2.16 | 1.35E-06 | Sash1 | SAM and SH3 domain containing 1 |
| ENSMUSG00000022890 | 1.11 | 2.16 | 1.09E-04 | Atp5j | ATP synthase, H+ transporting, mitochondrial F0 complex, subunit F |
| ENSMUSG00000051000 | 1.11 | 2.16 | 6.22E-06 | Fam160a1 | family with sequence similarity 160, member A1 |
| ENSMUSG00000030717 | 1.11 | 2.16 | 4.07E-05 | Nupr1 | nuclear protein transcription regulator 1 |
| ENSMUSG00000038403 | 1.11 | 2.15 | 2.14E-04 | Hfe2 | hemochromatosis type 2 (juvenile) (human homolog) |
| ENSMUSG00000023008 | 1.11 | 2.15 | 1.05E-08 | Fmnl3 | formin-like 3 |
| ENSMUSG00000051674 | 1.11 | 2.15 | 3.61E-07 | Dcun1d4 | DCN1, defective in cullin neddylation 1, domain containing 4 (S. cerevisiae) |
| ENSMUSG00000027854 | 1.11 | 2.15 | 7.33E-21 | Sike1 | suppressor of IKBKE 1 |
| ENSMUSG00000036989 | 1.11 | 2.15 | 2.55E-12 | Trim3 | tripartite motif-containing 3 |
| ENSMUSG00000083621 | 1.11 | 2.15 | 2.74E-03 | Gm14586 | predicted gene 14586 |
| ENSMUSG00000041734 | 1.11 | 2.15 | 3.64E-08 | Kirrel | kin of IRRE like (Drosophila) |
| ENSMUSG00000025902 | 1.10 | 2.15 | 1.64E-04 | Sox17 | SRY (sex determining region Y)-box 17 |
| ENSMUSG00000029093 | 1.10 | 2.15 | 2.00E-06 | Sorcs2 | sortilin-related VPS10 domain containing receptor 2 |
| ENSMUSG00000035506 | 1.10 | 2.15 | 8.92E-03 | Slc12a8 | solute carrier family 12 (potassium/chloride transporters), member 8 |
| ENSMUSG00000079523 | 1.10 | 2.15 | 2.80E-03 | NA | NA |
| ENSMUSG00000019055 | 1.10 | 2.15 | 8.43E-13 | Plod1 | procollagen-lysine, 2-oxoglutarate 5-dioxygenase 1 |
| ENSMUSG00000032062 | 1.10 | 2.15 | 2.33E-05 | 2310030G06Rik | RIKEN cDNA 2310030G06 gene |
| ENSMUSG00000070047 | 1.10 | 2.15 | 9.77E-06 | Fat1 | FAT tumor suppressor homolog 1 (Drosophila) |
| ENSMUSG00000012483 | 1.10 | 2.15 | 1.79E-03 | Rpa3 | replication protein A3 |
| ENSMUSG00000010751 | 1.10 | 2.15 | 2.64E-06 | Tnfrsf22 | tumor necrosis factor receptor superfamily, member 22 |
| ENSMUSG00000028403 | 1.10 | 2.15 | 1.41E-08 | Zdhhc21 | zinc finger, DHHC domain containing 21 |
| ENSMUSG00000041440 | 1.10 | 2.15 | 2.23E-05 | Gk5 | glycerol kinase 5 (putative) |
| ENSMUSG00000031398 | 1.10 | 2.14 | 4.91E-08 | Plxna3 | plexin A3 |
| ENSMUSG00000001418 | 1.10 | 2.14 | 3.07E-10 | 0610031J06Rik | RIKEN cDNA 0610031J06 gene |
| ENSMUSG00000022389 | 1.10 | 2.14 | 7.37E-10 | Tef | thyrotroph embryonic factor |
| ENSMUSG00000025466 | 1.10 | 2.14 | 2.21E-05 | Fuom | fucose mutarotase |
| ENSMUSG00000027673 | 1.10 | 2.14 | 1.39E-03 | Ndufb5 | NADH dehydrogenase (ubiquinone) 1 beta subcomplex, 5 |
| ENSMUSG00000025610 | 1.10 | 2.14 | 2.59E-03 | Map3k7cl | Map3k7 C-terminal like |
| ENSMUSG00000026072 | 1.10 | 2.14 | 6.25E-09 | Il1r1 | interleukin 1 receptor, type I |
| ENSMUSG00000059708 | 1.10 | 2.14 | 5.09E-05 | Akap17b | A kinase (PRKA) anchor protein 17B |
| ENSMUSG00000023259 | 1.10 | 2.14 | 2.26E-05 | NA | NA |
| ENSMUSG00000032523 | 1.10 | 2.14 | 2.02E-03 | Hhatl | hedgehog acyltransferase-like |
| ENSMUSG00000060538 | 1.10 | 2.14 | 6.46E-05 | Tmem219 | transmembrane protein 219 |
| ENSMUSG00000033577 | 1.09 | 2.14 | 1.43E-13 | Myo6 | myosin VI |
| ENSMUSG00000031383 | 1.09 | 2.14 | 2.83E-03 | Dusp9 | dual specificity phosphatase 9 |
| ENSMUSG00000008167 | 1.09 | 2.14 | 8.85E-07 | Fbxw9 | F-box and WD-40 domain protein 9 |
| ENSMUSG00000055912 | 1.09 | 2.13 | 3.43E-04 | Tmem150a | transmembrane protein 150A |
| ENSMUSG00000042216 | 1.09 | 2.13 | 9.82E-07 | Sgsm1 | small G protein signaling modulator 1 |
| ENSMUSG00000087380 | 1.09 | 2.13 | 5.85E-03 | 2210408F21Rik | RIKEN cDNA 2210408F21 gene |
| ENSMUSG00000071337 | 1.09 | 2.13 | 7.54E-12 | Tia1 | cytotoxic granule-associated RNA binding protein 1 |
| ENSMUSG00000022052 | 1.09 | 2.13 | 1.64E-11 | Ppp2r2a | protein phosphatase 2, regulatory subunit B, alpha |
| ENSMUSG00000083692 | 1.09 | 2.13 | 2.12E-05 | NA | NA |
| ENSMUSG00000022901 | 1.09 | 2.13 | 9.22E-05 | Cd86 | CD86 antigen |
| ENSMUSG00000025888 | 1.09 | 2.13 | 5.90E-06 | Casp1 | caspase 1 |
| ENSMUSG00000048572 | 1.09 | 2.13 | 3.09E-04 | Tmem252 | transmembrane protein 252 |
| ENSMUSG00000045039 | 1.09 | 2.13 | 1.45E-06 | Megf8 | multiple EGF-like-domains 8 |
| ENSMUSG00000001946 | 1.09 | 2.13 | 1.17E-08 | Esam | endothelial cell-specific adhesion molecule |
| ENSMUSG00000038860 | 1.09 | 2.13 | 1.59E-04 | Garnl3 | GTPase activating RANGAP domain-like 3 |
| ENSMUSG00000019539 | 1.09 | 2.13 | 1.34E-06 | Rcn3 | reticulocalbin 3, EF-hand calcium binding domain |
| ENSMUSG00000026111 | 1.09 | 2.13 | 7.01E-16 | Unc50 | unc-50 homolog (C. elegans) |
| ENSMUSG00000046814 | 1.09 | 2.13 | 4.70E-03 | Gchfr | GTP cyclohydrolase I feedback regulator |
| ENSMUSG00000004945 | 1.09 | 2.12 | 3.84E-07 | Tmem242 | transmembrane protein 242 |
| ENSMUSG00000030123 | 1.09 | 2.12 | 1.62E-09 | Plxnd1 | plexin D1 |
| ENSMUSG00000022674 | 1.09 | 2.12 | 9.09E-07 | Ube2v2 | ubiquitin-conjugating enzyme E2 variant 2 |
| ENSMUSG00000039199 | 1.09 | 2.12 | 1.12E-08 | Zdhhc1 | zinc finger, DHHC domain containing 1 |
| ENSMUSG00000029122 | 1.09 | 2.12 | 1.18E-07 | Evc | Ellis van Creveld gene syndrome |
| ENSMUSG00000033998 | 1.09 | 2.12 | 8.82E-07 | Kcnk1 | potassium channel, subfamily K, member 1 |
| ENSMUSG00000027004 | 1.09 | 2.12 | 1.86E-03 | Frzb | frizzled-related protein |
| ENSMUSG00000047749 | 1.09 | 2.12 | 1.75E-04 | Zc3hav1l | zinc finger CCCH-type, antiviral 1-like |
| ENSMUSG00000041777 | 1.08 | 2.12 | 1.11E-04 | Cir1 | corepressor interacting with RBPJ, 1 |
| ENSMUSG00000040857 | 1.08 | 2.12 | 4.54E-04 | Erf | Ets2 repressor factor |
| ENSMUSG00000031596 | 1.08 | 2.12 | 1.22E-04 | Slc7a2 | solute carrier family 7 (cationic amino acid transporter, y+ system), member 2 |
| ENSMUSG00000029710 | 1.08 | 2.12 | 2.39E-07 | Ephb4 | Eph receptor B4 |
| ENSMUSG00000017418 | 1.08 | 2.12 | 9.55E-05 | Arl5b | ADP-ribosylation factor-like 5B |
| ENSMUSG00000021133 | 1.08 | 2.12 | 2.35E-09 | 4933426M11Rik | RIKEN cDNA 4933426M11 gene |
| ENSMUSG00000032883 | 1.08 | 2.12 | 1.68E-05 | Acsl3 | acyl-CoA synthetase long-chain family member 3 |
| ENSMUSG00000040287 | 1.08 | 2.12 | 3.88E-04 | Stac3 | SH3 and cysteine rich domain 3 |
| ENSMUSG00000022297 | 1.08 | 2.12 | 1.15E-04 | Fzd6 | frizzled homolog 6 (Drosophila) |
| ENSMUSG00000069835 | 1.08 | 2.12 | 5.84E-04 | Sat2 | spermidine/spermine N1-acetyl transferase 2 |
| ENSMUSG00000025855 | 1.08 | 2.12 | 7.09E-06 | Prkar1b | protein kinase, cAMP dependent regulatory, type I beta |
| ENSMUSG00000041303 | 1.08 | 2.12 | 4.49E-10 | Gtf3c3 | general transcription factor IIIC, polypeptide 3 |
| ENSMUSG00000028996 | 1.08 | 2.12 | 5.53E-03 | Rbp7 | retinol binding protein 7, cellular |
| ENSMUSG00000063611 | 1.08 | 2.12 | 7.81E-03 | NA | NA |
| ENSMUSG00000031012 | 1.08 | 2.12 | 2.76E-07 | Cask | calcium/calmodulin-dependent serine protein kinase (MAGUK family) |
| ENSMUSG00000034175 | 1.08 | 2.11 | 1.08E-04 | Rhbdd3 | rhomboid domain containing 3 |
| ENSMUSG00000084013 | 1.08 | 2.11 | 4.28E-05 | NA | NA |
| ENSMUSG00000079110 | 1.08 | 2.11 | 3.25E-11 | Capn3 | calpain 3 |
| ENSMUSG00000085037 | 1.08 | 2.11 | 7.72E-05 | 4933421O10Rik | RIKEN cDNA 4933421O10 gene |
| ENSMUSG00000001833 | 1.08 | 2.11 | 1.54E-03 | Sept7 | septin 7 |
| ENSMUSG00000028466 | 1.08 | 2.11 | 7.85E-09 | Creb3 | cAMP responsive element binding protein 3 |
| ENSMUSG00000026276 | 1.08 | 2.11 | 4.26E-08 | Sept2 | septin 2 |
| ENSMUSG00000028345 | 1.08 | 2.11 | 2.76E-09 | Tex10 | testis expressed gene 10 |
| ENSMUSG00000051223 | 1.08 | 2.11 | 1.03E-16 | Bzw1 | basic leucine zipper and W2 domains 1 |
| ENSMUSG00000032372 | 1.08 | 2.11 | 2.48E-04 | Plscr2 | phospholipid scramblase 2 |
| ENSMUSG00000041189 | 1.08 | 2.11 | 2.98E-05 | Chrnb1 | cholinergic receptor, nicotinic, beta polypeptide 1 (muscle) |
| ENSMUSG00000020859 | 1.08 | 2.11 | 2.48E-08 | Spag9 | sperm associated antigen 9 |
| ENSMUSG00000013921 | 1.08 | 2.11 | 7.36E-05 | Clip3 | CAP-GLY domain containing linker protein 3 |
| ENSMUSG00000004127 | 1.08 | 2.11 | 2.35E-09 | Trmt10a | tRNA methyltransferase 10A |
| ENSMUSG00000030098 | 1.08 | 2.11 | 9.06E-03 | Grip2 | glutamate receptor interacting protein 2 |
| ENSMUSG00000023495 | 1.08 | 2.11 | 1.47E-04 | Pcbp4 | poly(rC) binding protein 4 |
| ENSMUSG00000048581 | 1.08 | 2.11 | 2.98E-03 | E130311K13Rik | RIKEN cDNA E130311K13 gene |
| ENSMUSG00000027394 | 1.07 | 2.11 | 6.06E-11 | Ttl | tubulin tyrosine ligase |
| ENSMUSG00000025949 | 1.07 | 2.11 | 5.58E-08 | Pikfyve | phosphoinositide kinase, FYVE finger containing |
| ENSMUSG00000039208 | 1.07 | 2.10 | 2.15E-05 | Metrnl | meteorin, glial cell differentiation regulator-like |
| ENSMUSG00000027868 | 1.07 | 2.10 | 5.48E-07 | Tbx15 | T-box 15 |
| ENSMUSG00000040389 | 1.07 | 2.10 | 3.57E-07 | Wdr47 | WD repeat domain 47 |
| ENSMUSG00000041633 | 1.07 | 2.10 | 2.76E-05 | Kctd12b | potassium channel tetramerisation domain containing 12b |
| ENSMUSG00000038987 | 1.07 | 2.10 | 3.28E-03 | 1700019L03Rik | RIKEN cDNA 1700019L03 gene |
| ENSMUSG00000037636 | 1.07 | 2.10 | 7.17E-03 | Slc25a43 | solute carrier family 25, member 43 |
| ENSMUSG00000055943 | 1.07 | 2.10 | 5.07E-10 | Emc7 | ER membrane protein complex subunit 7 |
| ENSMUSG00000063652 | 1.07 | 2.10 | 1.08E-04 | Slc22a21 | solute carrier family 22 (organic cation transporter), member 21 |
| ENSMUSG00000020818 | 1.07 | 2.10 | 2.21E-08 | Mfsd11 | major facilitator superfamily domain containing 11 |
| ENSMUSG00000020889 | 1.07 | 2.10 | 4.90E-03 | Nr1d1 | nuclear receptor subfamily 1, group D, member 1 |
| ENSMUSG00000031532 | 1.07 | 2.10 | 4.86E-10 | Tmem66 | transmembrane protein 66 |
| ENSMUSG00000046675 | 1.07 | 2.10 | 3.59E-07 | Tmem251 | transmembrane protein 251 |
| ENSMUSG00000044328 | 1.07 | 2.10 | 4.69E-05 | Trp53i13 | transformation related protein 53 inducible protein 13 |
| ENSMUSG00000004071 | 1.07 | 2.10 | 5.29E-11 | Cdip1 | cell death inducing Trp53 target 1 |
| ENSMUSG00000037577 | 1.07 | 2.10 | 6.36E-03 | Ephx3 | epoxide hydrolase 3 |
| ENSMUSG00000062470 | 1.07 | 2.10 | 9.62E-03 | Fbxl12os | F-box and leucine-rich repeat protein 12, opposite strand |
| ENSMUSG00000026088 | 1.07 | 2.10 | 8.43E-13 | Mitd1 | MIT, microtubule interacting and transport, domain containing 1 |
| ENSMUSG00000018340 | 1.07 | 2.10 | 5.21E-09 | Anxa6 | annexin A6 |
| ENSMUSG00000026490 | 1.07 | 2.10 | 5.44E-10 | Cdc42bpa | CDC42 binding protein kinase alpha |
| ENSMUSG00000074280 | 1.07 | 2.09 | 9.01E-06 | NA | NA |
| ENSMUSG00000000120 | 1.07 | 2.09 | 3.55E-03 | Ngfr | nerve growth factor receptor (TNFR superfamily, member 16) |
| ENSMUSG00000083557 | 1.07 | 2.09 | 2.59E-04 | NA | NA |
| ENSMUSG00000087528 | 1.07 | 2.09 | 7.14E-07 | NA | NA |
| ENSMUSG00000076432 | 1.07 | 2.09 | 1.03E-08 | Ywhaq | tyrosine 3-monooxygenase/tryptophan 5-monooxygenase activation protein, theta polypeptide |
| ENSMUSG00000038668 | 1.06 | 2.09 | 2.19E-08 | Lpar1 | lysophosphatidic acid receptor 1 |
| ENSMUSG00000031312 | 1.06 | 2.09 | 4.31E-04 | Itgb1bp2 | integrin beta 1 binding protein 2 |
| ENSMUSG00000000686 | 1.06 | 2.09 | 8.60E-05 | Abhd15 | abhydrolase domain containing 15 |
| ENSMUSG00000009376 | 1.06 | 2.09 | 3.99E-05 | Met | met proto-oncogene |
| ENSMUSG00000071252 | 1.06 | 2.09 | 1.16E-03 | 2210408I21Rik | RIKEN cDNA 2210408I21 gene |
| ENSMUSG00000015501 | 1.06 | 2.09 | 1.31E-04 | Hivep2 | human immunodeficiency virus type I enhancer binding protein 2 |
| ENSMUSG00000034126 | 1.06 | 2.09 | 5.49E-07 | Pomt2 | protein-O-mannosyltransferase 2 |
| ENSMUSG00000036391 | 1.06 | 2.09 | 2.48E-03 | Sec24a | Sec24 related gene family, member A (S. cerevisiae) |
| ENSMUSG00000003623 | 1.06 | 2.09 | 2.50E-12 | Crot | carnitine O-octanoyltransferase |
| ENSMUSG00000032252 | 1.06 | 2.09 | 1.23E-06 | Glce | glucuronyl C5-epimerase |
| ENSMUSG00000031278 | 1.06 | 2.08 | 1.42E-04 | Acsl4 | acyl-CoA synthetase long-chain family member 4 |
| ENSMUSG00000021916 | 1.06 | 2.08 | 8.26E-08 | Glt8d1 | glycosyltransferase 8 domain containing 1 |
| ENSMUSG00000045827 | 1.06 | 2.08 | 2.52E-06 | Serpinb9 | serine (or cysteine) peptidase inhibitor, clade B, member 9 |
| ENSMUSG00000010122 | 1.06 | 2.08 | 7.46E-03 | Slc47a1 | solute carrier family 47, member 1 |
| ENSMUSG00000058254 | 1.06 | 2.08 | 2.80E-07 | Tspan7 | tetraspanin 7 |
| ENSMUSG00000021794 | 1.06 | 2.08 | 9.62E-12 | Glud1 | glutamate dehydrogenase 1 |
| ENSMUSG00000031712 | 1.06 | 2.08 | 4.09E-04 | Il15 | interleukin 15 |
| ENSMUSG00000030494 | 1.06 | 2.08 | 3.51E-03 | Rhpn2 | rhophilin, Rho GTPase binding protein 2 |
| ENSMUSG00000017692 | 1.06 | 2.08 | 1.30E-06 | Rhbdl3 | rhomboid, veinlet-like 3 (Drosophila) |
| ENSMUSG00000034845 | 1.06 | 2.08 | 5.91E-04 | Plvap | plasmalemma vesicle associated protein |
| ENSMUSG00000030660 | 1.06 | 2.08 | 9.37E-03 | Pik3c2a | phosphatidylinositol 3-kinase, C2 domain containing, alpha polypeptide |
| ENSMUSG00000055172 | 1.06 | 2.08 | 5.49E-07 | C1ra | complement component 1, r subcomponent A |
| ENSMUSG00000048234 | 1.06 | 2.08 | 6.47E-08 | Rnf149 | ring finger protein 149 |
| ENSMUSG00000027935 | 1.06 | 2.08 | 1.02E-04 | Rab13 | RAB13, member RAS oncogene family |
| ENSMUSG00000027894 | 1.06 | 2.08 | 8.25E-04 | Slc6a17 | solute carrier family 6 (neurotransmitter transporter), member 17 |
| ENSMUSG00000049881 | 1.05 | 2.08 | 1.12E-06 | 2810025M15Rik | RIKEN cDNA 2810025M15 gene |
| ENSMUSG00000029673 | 1.05 | 2.08 | 4.08E-07 | Auts2 | autism susceptibility candidate 2 |
| ENSMUSG00000004843 | 1.05 | 2.07 | 1.11E-07 | Chmp2b | charged multivesicular body protein 2B |
| ENSMUSG00000044938 | 1.05 | 2.07 | 4.71E-03 | Klhl31 | kelch-like 31 |
| ENSMUSG00000039376 | 1.05 | 2.07 | 8.56E-04 | Synpo2l | synaptopodin 2-like |
| ENSMUSG00000000881 | 1.05 | 2.07 | 9.23E-13 | Dlg3 | discs, large homolog 3 (Drosophila) |
| ENSMUSG00000031969 | 1.05 | 2.07 | 1.70E-08 | Acad8 | acyl-Coenzyme A dehydrogenase family, member 8 |
| ENSMUSG00000020722 | 1.05 | 2.07 | 7.98E-04 | Cacng1 | calcium channel, voltage-dependent, gamma subunit 1 |
| ENSMUSG00000025287 | 1.05 | 2.07 | 2.55E-09 | Acot9 | acyl-CoA thioesterase 9 |
| ENSMUSG00000066150 | 1.05 | 2.07 | 5.49E-20 | Slc31a1 | solute carrier family 31, member 1 |
| ENSMUSG00000030315 | 1.05 | 2.07 | 3.71E-07 | Vgll4 | vestigial like 4 (Drosophila) |
| ENSMUSG00000046909 | 1.05 | 2.07 | 6.11E-05 | Tefm | transcription elongation factor, mitochondrial |
| ENSMUSG00000027088 | 1.05 | 2.07 | 5.18E-06 | Phospho2 | phosphatase, orphan 2 |
| ENSMUSG00000079197 | 1.05 | 2.07 | 1.30E-03 | NA | NA |
| ENSMUSG00000029757 | 1.05 | 2.07 | 1.24E-03 | Dync1i1 | dynein cytoplasmic 1 intermediate chain 1 |
| ENSMUSG00000021594 | 1.05 | 2.07 | 1.12E-05 | Srd5a1 | steroid 5 alpha-reductase 1 |
| ENSMUSG00000048096 | 1.05 | 2.07 | 4.15E-04 | Lmod1 | leiomodin 1 (smooth muscle) |
| ENSMUSG00000006699 | 1.05 | 2.07 | 1.81E-09 | Cdc42 | cell division cycle 42 |
| ENSMUSG00000023147 | 1.05 | 2.07 | 1.07E-06 | Wrb | tryptophan rich basic protein |
| ENSMUSG00000050064 | 1.05 | 2.07 | 4.46E-05 | Zfp697 | zinc finger protein 697 |
| ENSMUSG00000030990 | 1.05 | 2.07 | 3.58E-08 | Pgap2 | post-GPI attachment to proteins 2 |
| ENSMUSG00000027384 | 1.05 | 2.07 | 1.42E-06 | Ndufaf5 | NADH dehydrogenase (ubiquinone) complex I, assembly factor 5 |
| ENSMUSG00000020424 | 1.05 | 2.06 | 3.94E-05 | Gatsl3 | GATS protein-like 3 |
| ENSMUSG00000025969 | 1.05 | 2.06 | 9.83E-06 | Nrp2 | neuropilin 2 |
| ENSMUSG00000022995 | 1.05 | 2.06 | 1.03E-04 | Enah | enabled homolog (Drosophila) |
| ENSMUSG00000089791 | 1.05 | 2.06 | 2.12E-04 | NA | NA |
| ENSMUSG00000021567 | 1.05 | 2.06 | 2.16E-06 | Nkd2 | naked cuticle 2 homolog (Drosophila) |
| ENSMUSG00000032192 | 1.05 | 2.06 | 1.03E-07 | Gnb5 | guanine nucleotide binding protein (G protein), beta 5 |
| ENSMUSG00000067586 | 1.05 | 2.06 | 6.75E-06 | S1pr3 | sphingosine-1-phosphate receptor 3 |
| ENSMUSG00000027428 | 1.04 | 2.06 | 7.80E-05 | Rbbp9 | retinoblastoma binding protein 9 |
| ENSMUSG00000034220 | 1.04 | 2.06 | 3.20E-10 | Gpc1 | glypican 1 |
| ENSMUSG00000031458 | 1.04 | 2.06 | 1.06E-03 | Coprs | coordinator of PRMT5, differentiation stimulator |
| ENSMUSG00000035173 | 1.04 | 2.06 | 1.46E-06 | A630007B06Rik | RIKEN cDNA A630007B06 gene |
| ENSMUSG00000026303 | 1.04 | 2.06 | 3.06E-04 | Mlph | melanophilin |
| ENSMUSG00000030393 | 1.04 | 2.06 | 1.56E-03 | Zik1 | zinc finger protein interacting with K protein 1 |
| ENSMUSG00000044562 | 1.04 | 2.06 | 6.91E-05 | Rasip1 | Ras interacting protein 1 |
| ENSMUSG00000068735 | 1.04 | 2.06 | 2.49E-06 | Trp53i11 | transformation related protein 53 inducible protein 11 |
| ENSMUSG00000018672 | 1.04 | 2.06 | 4.59E-03 | Copz2 | coatomer protein complex, subunit zeta 2 |
| ENSMUSG00000002007 | 1.04 | 2.06 | 1.82E-03 | Srpk3 | serine/arginine-rich protein specific kinase 3 |
| ENSMUSG00000033389 | 1.04 | 2.06 | 6.63E-04 | Arhgap44 | Rho GTPase activating protein 44 |
| ENSMUSG00000030621 | 1.04 | 2.05 | 1.22E-03 | Me3 | malic enzyme 3, NADP(+)-dependent, mitochondrial |
| ENSMUSG00000026930 | 1.04 | 2.05 | 2.07E-05 | Gpsm1 | G-protein signalling modulator 1 (AGS3-like, C. elegans) |
| ENSMUSG00000041308 | 1.04 | 2.05 | 1.58E-06 | Sntb2 | syntrophin, basic 2 |
| ENSMUSG00000059474 | 1.04 | 2.05 | 1.35E-08 | Mbtd1 | mbt domain containing 1 |
| ENSMUSG00000032366 | 1.04 | 2.05 | 4.94E-04 | Tpm1 | tropomyosin 1, alpha |
| ENSMUSG00000031838 | 1.04 | 2.05 | 6.06E-07 | Ifi30 | interferon gamma inducible protein 30 |
| ENSMUSG00000001751 | 1.03 | 2.05 | 2.11E-07 | Naglu | alpha-N-acetylglucosaminidase (Sanfilippo disease IIIB) |
| ENSMUSG00000022951 | 1.03 | 2.05 | 3.67E-05 | Rcan1 | regulator of calcineurin 1 |
| ENSMUSG00000073771 | 1.03 | 2.05 | 7.68E-07 | NA | NA |
| ENSMUSG00000032413 | 1.03 | 2.05 | 3.57E-06 | Rasa2 | RAS p21 protein activator 2 |
| ENSMUSG00000032114 | 1.03 | 2.05 | 8.01E-05 | Slc37a4 | solute carrier family 37 (glucose-6-phosphate transporter), member 4 |
| ENSMUSG00000029376 | 1.03 | 2.05 | 1.81E-04 | Mthfd2l | methylenetetrahydrofolate dehydrogenase (NADP+ dependent) 2-like |
| ENSMUSG00000025492 | 1.03 | 2.05 | 5.30E-05 | Ifitm3 | interferon induced transmembrane protein 3 |
| ENSMUSG00000008686 | 1.03 | 2.04 | 1.49E-03 | NA | NA |
| ENSMUSG00000025050 | 1.03 | 2.04 | 3.27E-05 | Pcgf6 | polycomb group ring finger 6 |
| ENSMUSG00000037405 | 1.03 | 2.04 | 5.58E-06 | Icam1 | intercellular adhesion molecule 1 |
| ENSMUSG00000020585 | 1.03 | 2.04 | 1.12E-03 | Laptm4a | lysosomal-associated protein transmembrane 4A |
| ENSMUSG00000046027 | 1.03 | 2.04 | 1.71E-07 | Stard5 | StAR-related lipid transfer (START) domain containing 5 |
| ENSMUSG00000087396 | 1.03 | 2.04 | 1.70E-04 | 4933407K13Rik | RIKEN cDNA 4933407K13 gene |
| ENSMUSG00000040423 | 1.03 | 2.04 | 7.53E-05 | Rc3h1 | RING CCCH (C3H) domains 1 |
| ENSMUSG00000020422 | 1.03 | 2.04 | 3.78E-05 | Tns3 | tensin 3 |
| ENSMUSG00000057147 | 1.03 | 2.04 | 2.86E-07 | Dph6 | diphthamine biosynthesis 6 |
| ENSMUSG00000059897 | 1.03 | 2.04 | 3.33E-03 | Zfp930 | zinc finger protein 930 |
| ENSMUSG00000031156 | 1.03 | 2.04 | 3.32E-13 | Slc35a2 | solute carrier family 35 (UDP-galactose transporter), member A2 |
| ENSMUSG00000029804 | 1.03 | 2.04 | 1.35E-06 | Herc3 | hect domain and RLD 3 |
| ENSMUSG00000021969 | 1.03 | 2.04 | 2.45E-09 | Zdhhc20 | zinc finger, DHHC domain containing 20 |
| ENSMUSG00000028249 | 1.03 | 2.04 | 1.30E-14 | Sdcbp | syndecan binding protein |
| ENSMUSG00000009076 | 1.03 | 2.04 | 1.98E-04 | Zmat5 | zinc finger, matrin type 5 |
| ENSMUSG00000052151 | 1.03 | 2.04 | 1.43E-04 | Ppap2c | phosphatidic acid phosphatase type 2C |
| ENSMUSG00000034327 | 1.03 | 2.04 | 4.57E-07 | Kctd9 | potassium channel tetramerisation domain containing 9 |
| ENSMUSG00000020023 | 1.03 | 2.04 | 1.01E-07 | Tmcc3 | transmembrane and coiled coil domains 3 |
| ENSMUSG00000057614 | 1.03 | 2.04 | 8.46E-04 | Gnai1 | guanine nucleotide binding protein (G protein), alpha inhibiting 1 |
| ENSMUSG00000062762 | 1.03 | 2.04 | 1.83E-07 | Ei24 | etoposide induced 2.4 mRNA |
| ENSMUSG00000056708 | 1.03 | 2.04 | 1.80E-07 | Ier5 | immediate early response 5 |
| ENSMUSG00000044583 | 1.03 | 2.04 | 6.82E-05 | Tlr7 | toll-like receptor 7 |
| ENSMUSG00000030447 | 1.02 | 2.03 | 1.60E-12 | Cyfip1 | cytoplasmic FMR1 interacting protein 1 |
| ENSMUSG00000059810 | 1.02 | 2.03 | 1.91E-11 | Rgs3 | regulator of G-protein signaling 3 |
| ENSMUSG00000049119 | 1.02 | 2.03 | 4.43E-06 | Fam110b | family with sequence similarity 110, member B |
| ENSMUSG00000087260 | 1.02 | 2.03 | 5.33E-03 | Lamtor5 | late endosomal/lysosomal adaptor, MAPK and MTOR activator 5 |
| ENSMUSG00000010358 | 1.02 | 2.03 | 1.88E-04 | Ifi35 | interferon-induced protein 35 |
| ENSMUSG00000048264 | 1.02 | 2.03 | 1.14E-03 | Dip2c | DIP2 disco-interacting protein 2 homolog C (Drosophila) |
| ENSMUSG00000060301 | 1.02 | 2.03 | 9.06E-11 | 2610008E11Rik | RIKEN cDNA 2610008E11 gene |
| ENSMUSG00000021578 | 1.02 | 2.03 | 2.30E-19 | Ccdc127 | coiled-coil domain containing 127 |
| ENSMUSG00000062464 | 1.02 | 2.03 | 6.41E-03 | Cyp4f37 | cytochrome P450, family 4, subfamily f, polypeptide 37 |
| ENSMUSG00000071660 | 1.02 | 2.03 | 1.24E-05 | Ttc9c | tetratricopeptide repeat domain 9C |
| ENSMUSG00000055723 | 1.02 | 2.03 | 6.21E-06 | Rras2 | related RAS viral (r-ras) oncogene homolog 2 |
| ENSMUSG00000050921 | 1.02 | 2.03 | 2.46E-04 | P2ry10 | purinergic receptor P2Y, G-protein coupled 10 |
| ENSMUSG00000025531 | 1.02 | 2.03 | 1.26E-05 | Chm | choroidermia |
| ENSMUSG00000028229 | 1.02 | 2.03 | 6.11E-04 | Rmdn1 | regulator of microtubule dynamics 1 |
| ENSMUSG00000025092 | 1.02 | 2.03 | 1.73E-04 | Hspa12a | heat shock protein 12A |
| ENSMUSG00000016758 | 1.02 | 2.03 | 8.73E-03 | Bik | BCL2-interacting killer |
| ENSMUSG00000006542 | 1.02 | 2.03 | 4.25E-03 | Prkag3 | protein kinase, AMP-activated, gamma 3 non-catatlytic subunit |
| ENSMUSG00000067203 | 1.02 | 2.03 | 5.18E-03 | NA | NA |
| ENSMUSG00000035469 | 1.02 | 2.03 | 1.86E-10 | Rcbtb1 | regulator of chromosome condensation (RCC1) and BTB (POZ) domain containing protein 1 |
| ENSMUSG00000056486 | 1.02 | 2.02 | 3.37E-04 | Chn1 | chimerin 1 |
| ENSMUSG00000075701 | 1.02 | 2.02 | 2.92E-10 | Vimp | VCP-interacting membrane protein |
| ENSMUSG00000032281 | 1.02 | 2.02 | 8.26E-04 | Acsbg1 | acyl-CoA synthetase bubblegum family member 1 |
| ENSMUSG00000021711 | 1.02 | 2.02 | 1.48E-12 | Trappc13 | trafficking protein particle complex 13 |
| ENSMUSG00000021456 | 1.02 | 2.02 | 2.55E-03 | Fbp2 | fructose bisphosphatase 2 |
| ENSMUSG00000026825 | 1.02 | 2.02 | 1.52E-04 | Dnm1 | dynamin 1 |
| ENSMUSG00000056220 | 1.02 | 2.02 | 2.06E-10 | Pla2g4a | phospholipase A2, group IVA (cytosolic, calcium-dependent) |
| ENSMUSG00000023021 | 1.02 | 2.02 | 2.70E-09 | Cers5 | ceramide synthase 5 |
| ENSMUSG00000045294 | 1.02 | 2.02 | 2.09E-03 | Insig1 | insulin induced gene 1 |
| ENSMUSG00000017830 | 1.02 | 2.02 | 7.28E-11 | Dhx58 | DEXH (Asp-Glu-X-His) box polypeptide 58 |
| ENSMUSG00000022607 | 1.02 | 2.02 | 1.94E-09 | Ptk2 | PTK2 protein tyrosine kinase 2 |
| ENSMUSG00000086466 | 1.01 | 2.02 | 6.86E-04 | NA | NA |
| ENSMUSG00000027698 | 1.01 | 2.02 | 2.65E-09 | Nceh1 | neutral cholesterol ester hydrolase 1 |
| ENSMUSG00000012405 | 1.01 | 2.02 | 1.11E-09 | NA | NA |
| ENSMUSG00000021076 | 1.01 | 2.02 | 4.86E-05 | Actr10 | ARP10 actin-related protein 10 |
| ENSMUSG00000035342 | 1.01 | 2.02 | 3.25E-06 | Lzts2 | leucine zipper, putative tumor suppressor 2 |
| ENSMUSG00000028028 | 1.01 | 2.02 | 3.80E-04 | Alpk1 | alpha-kinase 1 |
| ENSMUSG00000038179 | 1.01 | 2.02 | 1.27E-04 | Slamf7 | SLAM family member 7 |
| ENSMUSG00000031955 | 1.01 | 2.02 | 3.90E-06 | Bcar1 | breast cancer anti-estrogen resistance 1 |
| ENSMUSG00000079140 | 1.01 | 2.02 | 4.63E-04 | NA | NA |
| ENSMUSG00000025981 | 1.01 | 2.02 | 6.80E-05 | Coq10b | coenzyme Q10 homolog B (S. cerevisiae) |
| ENSMUSG00000024713 | 1.01 | 2.01 | 3.59E-08 | Pcsk5 | proprotein convertase subtilisin/kexin type 5 |
| ENSMUSG00000028164 | 1.01 | 2.01 | 2.00E-14 | Manba | mannosidase, beta A, lysosomal |
| ENSMUSG00000029684 | 1.01 | 2.01 | 2.09E-05 | Wasl | Wiskott-Aldrich syndrome-like (human) |
| ENSMUSG00000032179 | 1.01 | 2.01 | 2.44E-09 | Bmp5 | bone morphogenetic protein 5 |
| ENSMUSG00000068551 | 1.01 | 2.01 | 1.43E-05 | Zfp467 | zinc finger protein 467 |
| ENSMUSG00000027739 | 1.01 | 2.01 | 2.14E-12 | Rab33b | RAB33B, member of RAS oncogene family |
| ENSMUSG00000047415 | 1.01 | 2.01 | 6.19E-03 | Gpr68 | G protein-coupled receptor 68 |
| ENSMUSG00000018567 | 1.01 | 2.01 | 1.11E-06 | Gabarap | gamma-aminobutyric acid receptor associated protein |
| ENSMUSG00000034593 | 1.01 | 2.01 | 6.48E-09 | Myo5a | myosin VA |
| ENSMUSG00000000563 | 1.01 | 2.01 | 1.56E-06 | Atp5f1 | ATP synthase, H+ transporting, mitochondrial F0 complex, subunit B1 |
| ENSMUSG00000038205 | 1.01 | 2.01 | 5.69E-05 | Prkab2 | protein kinase, AMP-activated, beta 2 non-catalytic subunit |
| ENSMUSG00000056290 | 1.01 | 2.01 | 1.55E-03 | Ms4a4b | membrane-spanning 4-domains, subfamily A, member 4B |
| ENSMUSG00000021583 | 1.01 | 2.01 | 7.79E-15 | Erap1 | endoplasmic reticulum aminopeptidase 1 |
| ENSMUSG00000029649 | 1.01 | 2.01 | 1.84E-06 | Pomp | proteasome maturation protein |
| ENSMUSG00000042289 | 1.01 | 2.01 | 3.53E-11 | Hsd3b7 | hydroxy-delta-5-steroid dehydrogenase, 3 beta- and steroid delta-isomerase 7 |
| ENSMUSG00000030446 | 1.00 | 2.01 | 5.25E-03 | Zfp273 | zinc finger protein 273 |
| ENSMUSG00000042302 | 1.00 | 2.01 | 3.49E-07 | Ehbp1 | EH domain binding protein 1 |
| ENSMUSG00000054555 | 1.00 | 2.01 | 3.79E-04 | Adam12 | a disintegrin and metallopeptidase domain 12 (meltrin alpha) |
| ENSMUSG00000037997 | 1.00 | 2.00 | 3.92E-06 | Parp11 | poly (ADP-ribose) polymerase family, member 11 |
| ENSMUSG00000038991 | 1.00 | 2.00 | 9.99E-11 | Txndc5 | thioredoxin domain containing 5 |
| ENSMUSG00000087598 | 1.00 | 2.00 | 1.76E-05 | Zfp111 | zinc finger protein 111 |
| ENSMUSG00000034724 | 1.00 | 2.00 | 4.11E-10 | Cnot6l | CCR4-NOT transcription complex, subunit 6-like |
| ENSMUSG00000021497 | 1.00 | 2.00 | 3.41E-14 | Txndc15 | thioredoxin domain containing 15 |
| ENSMUSG00000021242 | 1.00 | 2.00 | 2.49E-09 | Npc2 | Niemann Pick type C2 |
| ENSMUSG00000086780 | 1.00 | 2.00 | 7.99E-03 | NA | NA |
| ENSMUSG00000058267 | 1.00 | 2.00 | 5.84E-09 | Mrps14 | mitochondrial ribosomal protein S14 |
| ENSMUSG00000079329 | -6.05 | -66.35 | 6.36E-78 | NA | NA |
| ENSMUSG00000083399 | -5.92 | -60.34 | 2.04E-43 | NA | NA |
| ENSMUSG00000058838 | -5.85 | -57.59 | 4.26E-38 | NA | NA |
| ENSMUSG00000048191 | -5.69 | -51.47 | 3.48E-112 | Muc6 | mucin 6, gastric |
| ENSMUSG00000082473 | -5.42 | -42.78 | 1.32E-20 | NA | NA |
| ENSMUSG00000056380 | -5.21 | -36.94 | 9.16E-24 | Gpr50 | G-protein-coupled receptor 50 |
| ENSMUSG00000068969 | -4.78 | -27.52 | 4.24E-57 | NA | NA |
| ENSMUSG00000082755 | -4.71 | -26.17 | 1.09E-12 | Gm8692 | predicted gene 8692 |
| ENSMUSG00000054385 | -4.68 | -25.72 | 1.44E-64 | Ceacam2 | carcinoembryonic antigen-related cell adhesion molecule 2 |
| ENSMUSG00000090101 | -4.58 | -23.90 | 4.28E-11 | Snhg9 | small nucleolar RNA host gene (non-protein coding) 9 |
| ENSMUSG00000081853 | -4.49 | -22.42 | 4.10E-41 | NA | NA |
| ENSMUSG00000076609 | -4.40 | -21.09 | 1.24E-17 | NA | NA |
| ENSMUSG00000050157 | -4.32 | -19.93 | 1.08E-44 | NA | NA |
| ENSMUSG00000056820 | -4.29 | -19.54 | 4.91E-24 | Tsnax | translin-associated factor X |
| ENSMUSG00000071292 | -4.28 | -19.37 | 4.11E-09 | NA | NA |
| ENSMUSG00000082080 | -4.18 | -18.11 | 1.02E-13 | NA | NA |
| ENSMUSG00000090243 | -4.14 | -17.67 | 4.23E-09 | NA | NA |
| ENSMUSG00000082658 | -4.14 | -17.61 | 4.03E-10 | NA | NA |
| ENSMUSG00000025400 | -4.14 | -17.61 | 4.71E-32 | Tac2 | tachykinin 2 |
| ENSMUSG00000035504 | -4.12 | -17.43 | 5.04E-47 | Reep6 | receptor accessory protein 6 |
| ENSMUSG00000085606 | -4.06 | -16.68 | 1.41E-20 | NA | NA |
| ENSMUSG00000031786 | -4.05 | -16.59 | 8.36E-40 | Ccdc135 | coiled-coil domain containing 135 |
| ENSMUSG00000085351 | -4.03 | -16.34 | 3.67E-08 | NA | NA |
| ENSMUSG00000081382 | -4.02 | -16.18 | 4.97E-37 | NA | NA |
| ENSMUSG00000070834 | -3.97 | -15.68 | 7.95E-08 | NA | NA |
| ENSMUSG00000084196 | -3.94 | -15.35 | 8.53E-07 | NA | NA |
| ENSMUSG00000046959 | -3.94 | -15.33 | 1.80E-75 | Slc26a1 | solute carrier family 26 (sulfate transporter), member 1 |
| ENSMUSG00000014030 | -3.91 | -15.07 | 1.42E-18 | Pax5 | paired box 5 |
| ENSMUSG00000050788 | -3.89 | -14.84 | 3.58E-14 | Olfr419 | olfactory receptor 419 |
| ENSMUSG00000052019 | -3.89 | -14.80 | 2.34E-16 | NA | NA |
| ENSMUSG00000053338 | -3.89 | -14.78 | 2.55E-36 | Tarm1 | T cell-interacting, activating receptor on myeloid cells 1 |
| ENSMUSG00000079707 | -3.88 | -14.76 | 6.83E-07 | NA | NA |
| ENSMUSG00000061991 | -3.86 | -14.54 | 7.58E-08 | Hist1h2af | histone cluster 1, H2af |
| ENSMUSG00000055609 | -3.86 | -14.48 | 4.47E-07 | Hba-x | hemoglobin X, alpha-like embryonic chain in Hba complex |
| ENSMUSG00000082701 | -3.85 | -14.42 | 3.35E-06 | NA | NA |
| ENSMUSG00000077394 | -3.81 | -14.05 | 4.17E-11 | NA | NA |
| ENSMUSG00000089937 | -3.81 | -14.02 | 2.78E-06 | NA | NA |
| ENSMUSG00000089417 | -3.80 | -13.94 | 1.34E-12 | NA | NA |
| ENSMUSG00000086691 | -3.80 | -13.92 | 2.35E-11 | NA | NA |
| ENSMUSG00000086377 | -3.78 | -13.78 | 5.05E-06 | 4930529C04Rik | zinc finger, BED domain containing 4 pseudogene |
| ENSMUSG00000072600 | -3.74 | -13.36 | 3.66E-12 | NA | NA |
| ENSMUSG00000086438 | -3.74 | -13.33 | 7.02E-21 | Asb17os | ankyrin repeat and SOCS box-containing 17, opposite strand |
| ENSMUSG00000047222 | -3.72 | -13.17 | 6.62E-18 | Ear11 | eosinophil-associated, ribonuclease A family, member 11 |
| ENSMUSG00000083253 | -3.72 | -13.16 | 2.35E-45 | NA | NA |
| ENSMUSG00000013415 | -3.69 | -12.91 | 3.80E-06 | Igf2bp1 | insulin-like growth factor 2 mRNA binding protein 1 |
| ENSMUSG00000023902 | -3.69 | -12.88 | 4.11E-08 | Zscan10 | zinc finger and SCAN domain containing 10 |
| ENSMUSG00000066002 | -3.69 | -12.86 | 1.11E-09 | NA | NA |
| ENSMUSG00000074562 | -3.68 | -12.86 | 2.37E-06 | NA | NA |
| ENSMUSG00000054909 | -3.68 | -12.85 | 1.54E-07 | Wbscr25 | Williams Beuren syndrome chromosome region 25 (human) |
| ENSMUSG00000059657 | -3.68 | -12.85 | 6.47E-11 | Stfa2l1 | stefin A2 like 1 |
| ENSMUSG00000064722 | -3.67 | -12.73 | 1.14E-05 | NA | NA |
| ENSMUSG00000084213 | -3.67 | -12.72 | 2.11E-07 | NA | NA |
| ENSMUSG00000003379 | -3.66 | -12.61 | 3.98E-31 | Cd79a | CD79A antigen (immunoglobulin-associated alpha) |
| ENSMUSG00000081046 | -3.66 | -12.61 | 8.51E-06 | NA | NA |
| ENSMUSG00000081383 | -3.64 | -12.44 | 1.08E-05 | NA | NA |
| ENSMUSG00000064437 | -3.64 | -12.43 | 2.52E-10 | Snord49b | small nucleolar RNA, C/D box 49B |
| ENSMUSG00000082220 | -3.61 | -12.20 | 1.98E-07 | NA | NA |
| ENSMUSG00000081926 | -3.60 | -12.11 | 7.18E-06 | NA | NA |
| ENSMUSG00000068105 | -3.60 | -12.10 | 5.16E-14 | Tnfrsf13c | tumor necrosis factor receptor superfamily, member 13c |
| ENSMUSG00000032556 | -3.58 | -11.92 | 1.14E-10 | Bfsp2 | beaded filament structural protein 2, phakinin |
| ENSMUSG00000083998 | -3.57 | -11.91 | 3.13E-05 | NA | NA |
| ENSMUSG00000079310 | -3.56 | -11.78 | 4.34E-85 | NA | NA |
| ENSMUSG00000041911 | -3.54 | -11.63 | 3.33E-06 | Dlx1 | distal-less homeobox 1 |
| ENSMUSG00000019146 | -3.51 | -11.38 | 1.81E-05 | Cacng2 | calcium channel, voltage-dependent, gamma subunit 2 |
| ENSMUSG00000067156 | -3.49 | -11.20 | 3.94E-05 | Gm7337 | DAZ interacting protein 1 pseudogene |
| ENSMUSG00000032226 | -3.48 | -11.13 | 3.67E-05 | Gcnt3 | glucosaminyl (N-acetyl) transferase 3, mucin type |
| ENSMUSG00000040592 | -3.47 | -11.09 | 1.91E-43 | Cd79b | CD79B antigen |
| ENSMUSG00000064281 | -3.44 | -10.88 | 1.31E-16 | NA | NA |
| ENSMUSG00000041841 | -3.44 | -10.84 | 2.84E-07 | NA | NA |
| ENSMUSG00000030413 | -3.42 | -10.71 | 1.92E-30 | Pglyrp1 | peptidoglycan recognition protein 1 |
| ENSMUSG00000055033 | -3.41 | -10.66 | 6.37E-05 | Olfr420 | olfactory receptor 420 |
| ENSMUSG00000081575 | -3.41 | -10.65 | 7.80E-05 | NA | NA |
| ENSMUSG00000028832 | -3.41 | -10.64 | 3.13E-29 | Stmn1 | stathmin 1 |
| ENSMUSG00000074122 | -3.41 | -10.61 | 5.90E-05 | NA | NA |
| ENSMUSG00000026815 | -3.40 | -10.58 | 1.00E-29 | Gfi1b | growth factor independent 1B |
| ENSMUSG00000000766 | -3.40 | -10.57 | 1.17E-05 | Oprm1 | opioid receptor, mu 1 |
| ENSMUSG00000027070 | -3.40 | -10.57 | 8.02E-06 | Lrp2 | low density lipoprotein receptor-related protein 2 |
| ENSMUSG00000032053 | -3.39 | -10.49 | 5.13E-20 | Pou2af1 | POU domain, class 2, associating factor 1 |
| ENSMUSG00000084292 | -3.36 | -10.28 | 9.84E-06 | NA | NA |
| ENSMUSG00000010505 | -3.36 | -10.27 | 4.47E-05 | Myt1 | myelin transcription factor 1 |
| ENSMUSG00000038218 | -3.36 | -10.25 | 1.82E-05 | NA | NA |
| ENSMUSG00000084184 | -3.35 | -10.20 | 5.68E-09 | NA | NA |
| ENSMUSG00000034384 | -3.35 | -10.17 | 5.25E-05 | Barhl2 | BarH-like 2 (Drosophila) |
| ENSMUSG00000047021 | -3.35 | -10.17 | 2.58E-05 | Ccdc108 | coiled-coil domain containing 108 |
| ENSMUSG00000021061 | -3.33 | -10.05 | 7.27E-25 | Sptb | spectrin beta, erythrocytic |
| ENSMUSG00000081985 | -3.32 | -10.01 | 1.70E-09 | NA | NA |
| ENSMUSG00000084893 | -3.32 | -9.95 | 7.32E-05 | NA | NA |
| ENSMUSG00000083186 | -3.31 | -9.91 | 1.28E-13 | NA | NA |
| ENSMUSG00000081933 | -3.30 | -9.83 | 5.17E-17 | NA | NA |
| ENSMUSG00000046179 | -3.30 | -9.82 | 1.75E-34 | E2f8 | E2F transcription factor 8 |
| ENSMUSG00000026073 | -3.29 | -9.79 | 1.25E-16 | Il1r2 | interleukin 1 receptor, type II |
| ENSMUSG00000083416 | -3.29 | -9.79 | 1.90E-29 | NA | NA |
| ENSMUSG00000007044 | -3.29 | -9.76 | 9.55E-28 | NA | NA |
| ENSMUSG00000086741 | -3.28 | -9.72 | 5.55E-19 | Gm15816 | predicted gene 15816 |
| ENSMUSG00000084814 | -3.28 | -9.70 | 5.04E-19 | NA | NA |
| ENSMUSG00000083549 | -3.27 | -9.67 | 2.39E-08 | NA | NA |
| ENSMUSG00000030000 | -3.27 | -9.65 | 3.37E-36 | Add2 | adducin 2 (beta) |
| ENSMUSG00000031543 | -3.27 | -9.64 | 5.50E-27 | Ank1 | ankyrin 1, erythroid |
| ENSMUSG00000081389 | -3.26 | -9.61 | 7.72E-06 | NA | NA |
| ENSMUSG00000020932 | -3.26 | -9.60 | 3.50E-33 | Gfap | glial fibrillary acidic protein |
| ENSMUSG00000081964 | -3.26 | -9.57 | 8.32E-05 | NA | NA |
| ENSMUSG00000037033 | -3.25 | -9.51 | 6.42E-11 | Clca4 | chloride channel calcium activated 4 |
| ENSMUSG00000060560 | -3.24 | -9.48 | 3.02E-05 | Ces4a | carboxylesterase 4A |
| ENSMUSG00000082797 | -3.24 | -9.44 | 1.47E-04 | NA | NA |
| ENSMUSG00000020018 | -3.24 | -9.42 | 1.01E-12 | Snrpf | small nuclear ribonucleoprotein polypeptide F |
| ENSMUSG00000060566 | -3.23 | -9.39 | 1.91E-04 | NA | NA |
| ENSMUSG00000054753 | -3.22 | -9.35 | 6.35E-05 | NA | NA |
| ENSMUSG00000047061 | -3.22 | -9.34 | 9.55E-05 | NA | NA |
| ENSMUSG00000087709 | -3.22 | -9.34 | 3.31E-04 | NA | NA |
| ENSMUSG00000002341 | -3.22 | -9.30 | 1.11E-05 | Ncan | neurocan |
| ENSMUSG00000025243 | -3.21 | -9.22 | 4.34E-06 | Slc6a20b | solute carrier family 6 (neurotransmitter transporter), member 20B |
| ENSMUSG00000049577 | -3.20 | -9.21 | 9.80E-25 | Zfpm1 | zinc finger protein, multitype 1 |
| ENSMUSG00000083438 | -3.19 | -9.12 | 2.42E-18 | NA | NA |
| ENSMUSG00000079259 | -3.18 | -9.09 | 1.91E-04 | Trim71 | tripartite motif-containing 71 |
| ENSMUSG00000005148 | -3.18 | -9.09 | 3.57E-25 | Klf5 | Kruppel-like factor 5 |
| ENSMUSG00000003469 | -3.18 | -9.08 | 1.09E-25 | Phyhip | phytanoyl-CoA hydroxylase interacting protein |
| ENSMUSG00000074203 | -3.18 | -9.04 | 4.34E-28 | NA | NA |
| ENSMUSG00000085823 | -3.18 | -9.03 | 3.14E-13 | NA | NA |
| ENSMUSG00000024064 | -3.17 | -9.03 | 3.02E-05 | Galnt14 | UDP-N-acetyl-alpha-D-galactosamine:polypeptide N-acetylgalactosaminyltransferase 14 |
| ENSMUSG00000086938 | -3.17 | -9.03 | 6.76E-07 | 4930481A15Rik | RIKEN cDNA 4930481A15 gene |
| ENSMUSG00000085363 | -3.17 | -9.01 | 6.28E-05 | NA | NA |
| ENSMUSG00000053310 | -3.17 | -8.98 | 1.55E-29 | Nrgn | neurogranin |
| ENSMUSG00000041831 | -3.16 | -8.95 | 6.46E-15 | NA | NA |
| ENSMUSG00000060275 | -3.16 | -8.93 | 1.16E-26 | Nrg2 | neuregulin 2 |
| ENSMUSG00000081086 | -3.16 | -8.92 | 4.08E-04 | NA | NA |
| ENSMUSG00000029819 | -3.15 | -8.87 | 6.65E-34 | Npy | neuropeptide Y |
| ENSMUSG00000031535 | -3.15 | -8.86 | 3.21E-04 | Dkk4 | dickkopf homolog 4 (Xenopus laevis) |
| ENSMUSG00000081552 | -3.15 | -8.86 | 3.08E-04 | NA | NA |
| ENSMUSG00000042750 | -3.15 | -8.85 | 3.81E-09 | Bex2 | brain expressed X-linked 2 |
| ENSMUSG00000042345 | -3.14 | -8.84 | 1.87E-30 | Ubash3a | ubiquitin associated and SH3 domain containing, A |
| ENSMUSG00000054428 | -3.14 | -8.81 | 1.95E-29 | Atpif1 | ATPase inhibitory factor 1 |
| ENSMUSG00000061540 | -3.13 | -8.78 | 3.02E-14 | Orm2 | orosomucoid 2 |
| ENSMUSG00000024034 | -3.13 | -8.78 | 1.05E-04 | Tmprss3 | transmembrane protease, serine 3 |
| ENSMUSG00000086761 | -3.13 | -8.77 | 2.95E-04 | NA | NA |
| ENSMUSG00000082530 | -3.12 | -8.69 | 8.93E-32 | NA | NA |
| ENSMUSG00000033450 | -3.12 | -8.68 | 3.07E-11 | Tagap | T cell activation Rho GTPase activating protein |
| ENSMUSG00000002324 | -3.12 | -8.68 | 9.22E-14 | Rec8 | REC8 homolog (yeast) |
| ENSMUSG00000042724 | -3.11 | -8.63 | 1.08E-21 | Map3k9 | mitogen-activated protein kinase kinase kinase 9 |
| ENSMUSG00000064540 | -3.11 | -8.61 | 5.54E-08 | NA | NA |
| ENSMUSG00000085006 | -3.10 | -8.56 | 7.11E-07 | NA | NA |
| ENSMUSG00000030792 | -3.10 | -8.56 | 2.50E-22 | Dkkl1 | dickkopf-like 1 |
| ENSMUSG00000066009 | -3.10 | -8.55 | 4.67E-04 | Gm13051 | predicted gene 13051 |
| ENSMUSG00000082117 | -3.09 | -8.52 | 6.15E-04 | NA | NA |
| ENSMUSG00000009350 | -3.09 | -8.51 | 1.45E-23 | Mpo | myeloperoxidase |
| ENSMUSG00000038997 | -3.09 | -8.50 | 5.53E-10 | Asb17 | ankyrin repeat and SOCS box-containing 17 |
| ENSMUSG00000080992 | -3.09 | -8.49 | 4.08E-04 | NA | NA |
| ENSMUSG00000086749 | -3.08 | -8.48 | 7.55E-06 | NA | NA |
| ENSMUSG00000072697 | -3.08 | -8.45 | 1.95E-09 | NA | NA |
| ENSMUSG00000081924 | -3.08 | -8.45 | 4.91E-05 | NA | NA |
| ENSMUSG00000082332 | -3.08 | -8.43 | 3.26E-26 | NA | NA |
| ENSMUSG00000000903 | -3.07 | -8.42 | 8.30E-16 | Vpreb3 | pre-B lymphocyte gene 3 |
| ENSMUSG00000089959 | -3.07 | -8.41 | 1.20E-06 | NA | NA |
| ENSMUSG00000076469 | -3.07 | -8.41 | 7.64E-07 | NA | NA |
| ENSMUSG00000035963 | -3.07 | -8.40 | 1.78E-09 | Odf3l2 | outer dense fiber of sperm tails 3-like 2 |
| ENSMUSG00000026822 | -3.07 | -8.39 | 6.65E-25 | Lcn2 | lipocalin 2 |
| ENSMUSG00000088307 | -3.07 | -8.39 | 3.24E-04 | NA | NA |
| ENSMUSG00000066392 | -3.07 | -8.39 | 2.90E-04 | Nrxn3 | neurexin III |
| ENSMUSG00000074561 | -3.07 | -8.38 | 2.19E-04 | NA | NA |
| ENSMUSG00000034764 | -3.07 | -8.38 | 4.79E-31 | NA | NA |
| ENSMUSG00000081744 | -3.07 | -8.37 | 2.90E-04 | NA | NA |
| ENSMUSG00000077340 | -3.06 | -8.36 | 4.49E-04 | NA | NA |
| ENSMUSG00000031933 | -3.06 | -8.33 | 4.16E-04 | Folr4 | folate receptor 4 (delta) |
| ENSMUSG00000071234 | -3.06 | -8.32 | 9.74E-08 | Syndig1l | synapse differentiation inducing 1 like |
| ENSMUSG00000067103 | -3.06 | -8.32 | 3.49E-04 | AY702103 | cDNA sequence AY702103 |
| ENSMUSG00000066389 | -3.06 | -8.31 | 1.50E-04 | NA | NA |
| ENSMUSG00000082336 | -3.05 | -8.30 | 2.41E-08 | NA | NA |
| ENSMUSG00000078500 | -3.04 | -8.25 | 5.82E-04 | NA | NA |
| ENSMUSG00000075296 | -3.04 | -8.24 | 2.11E-31 | NA | NA |
| ENSMUSG00000059305 | -3.04 | -8.24 | 7.44E-21 | Vpreb1 | pre-B lymphocyte gene 1 |
| ENSMUSG00000056656 | -3.03 | -8.16 | 2.34E-26 | Apol8 | apolipoprotein L 8 |
| ENSMUSG00000040935 | -3.03 | -8.14 | 3.37E-04 | Padi6 | peptidyl arginine deiminase, type VI |
| ENSMUSG00000074126 | -3.03 | -8.14 | 3.21E-04 | NA | NA |
| ENSMUSG00000050675 | -3.03 | -8.14 | 3.58E-27 | Gp1ba | glycoprotein 1b, alpha polypeptide |
| ENSMUSG00000021867 | -3.02 | -8.14 | 3.09E-04 | NA | NA |
| ENSMUSG00000084755 | -3.02 | -8.13 | 2.81E-13 | NA | NA |
| ENSMUSG00000060161 | -3.02 | -8.12 | 4.32E-04 | Klk1b7-ps | kallikrein 1-related peptidase b7, pseudogene |
| ENSMUSG00000034634 | -3.02 | -8.11 | 1.72E-14 | Ly6d | lymphocyte antigen 6 complex, locus D |
| ENSMUSG00000079188 | -3.02 | -8.09 | 2.65E-18 | NA | NA |
| ENSMUSG00000053228 | -3.01 | -8.05 | 4.97E-04 | Ceacam3 | carcinoembryonic antigen-related cell adhesion molecule 3 |
| ENSMUSG00000032221 | -3.00 | -8.02 | 5.03E-17 | Mns1 | meiosis-specific nuclear structural protein 1 |
| ENSMUSG00000049916 | -3.00 | -8.00 | 1.24E-25 | 2610318N02Rik | RIKEN cDNA 2610318N02 gene |
| ENSMUSG00000006574 | -3.00 | -7.99 | 1.14E-23 | Slc4a1 | solute carrier family 4 (anion exchanger), member 1 |
| ENSMUSG00000027401 | -3.00 | -7.99 | 8.22E-26 | Tgm3 | transglutaminase 3, E polypeptide |
| ENSMUSG00000079579 | -3.00 | -7.98 | 6.72E-04 | Gm6760 | predicted gene 6760 |
| ENSMUSG00000001076 | -3.00 | -7.97 | 4.41E-04 | C1ql4 | complement component 1, q subcomponent-like 4 |
| ENSMUSG00000041237 | -2.99 | -7.97 | 4.44E-22 | Pklr | pyruvate kinase liver and red blood cell |
| ENSMUSG00000085812 | -2.99 | -7.92 | 6.17E-05 | NA | NA |
| ENSMUSG00000039676 | -2.99 | -7.92 | 1.71E-06 | Capsl | calcyphosine-like |
| ENSMUSG00000040466 | -2.98 | -7.90 | 7.92E-31 | Blvrb | biliverdin reductase B (flavin reductase (NADPH)) |
| ENSMUSG00000049612 | -2.98 | -7.87 | 9.36E-10 | Omg | oligodendrocyte myelin glycoprotein |
| ENSMUSG00000017588 | -2.96 | -7.80 | 5.07E-04 | Krt27 | keratin 27 |
| ENSMUSG00000078939 | -2.96 | -7.80 | 7.45E-08 | NA | NA |
| ENSMUSG00000063816 | -2.96 | -7.77 | 2.35E-04 | NA | NA |
| ENSMUSG00000021056 | -2.95 | -7.75 | 5.22E-04 | Tex21 | testis expressed gene 21 |
| ENSMUSG00000084348 | -2.95 | -7.75 | 1.23E-07 | NA | NA |
| ENSMUSG00000004552 | -2.95 | -7.74 | 6.00E-33 | Ctse | cathepsin E |
| ENSMUSG00000083796 | -2.95 | -7.72 | 1.23E-05 | NA | NA |
| ENSMUSG00000087299 | -2.95 | -7.71 | 7.64E-20 | NA | NA |
| ENSMUSG00000082177 | -2.95 | -7.70 | 8.11E-04 | NA | NA |
| ENSMUSG00000070886 | -2.94 | -7.68 | 6.23E-04 | NA | NA |
| ENSMUSG00000079472 | -2.94 | -7.68 | 6.48E-12 | NA | NA |
| ENSMUSG00000024588 | -2.94 | -7.67 | 1.36E-44 | Fech | ferrochelatase |
| ENSMUSG00000059244 | -2.94 | -7.67 | 6.94E-04 | NA | NA |
| ENSMUSG00000069125 | -2.94 | -7.66 | 7.07E-04 | NA | NA |
| ENSMUSG00000021416 | -2.93 | -7.64 | 2.49E-08 | Eci3 | enoyl-Coenzyme A delta isomerase 3 |
| ENSMUSG00000021848 | -2.93 | -7.60 | 7.17E-04 | Otx2 | orthodenticle homolog 2 |
| ENSMUSG00000021792 | -2.93 | -7.60 | 7.20E-18 | Fam213a | family with sequence similarity 213, member A |
| ENSMUSG00000006389 | -2.92 | -7.59 | 4.68E-29 | Mpl | myeloproliferative leukemia virus oncogene |
| ENSMUSG00000006345 | -2.92 | -7.58 | 6.93E-13 | Ggt1 | gamma-glutamyltransferase 1 |
| ENSMUSG00000085298 | -2.92 | -7.58 | 4.41E-05 | F730035M05Rik | RIKEN cDNA F730035M05 gene |
| ENSMUSG00000048215 | -2.92 | -7.56 | 1.83E-07 | A630023P12Rik | RIKEN cDNA A630023P12 gene |
| ENSMUSG00000080708 | -2.92 | -7.55 | 3.00E-22 | NA | NA |
| ENSMUSG00000063804 | -2.92 | -7.54 | 6.43E-04 | Lin28b | lin-28 homolog B (C. elegans) |
| ENSMUSG00000048483 | -2.91 | -7.51 | 2.66E-04 | Zdhhc22 | zinc finger, DHHC-type containing 22 |
| ENSMUSG00000024223 | -2.91 | -7.51 | 4.34E-09 | Armc12 | armadillo repeat containing 12 |
| ENSMUSG00000043747 | -2.91 | -7.50 | 2.52E-04 | NA | NA |
| ENSMUSG00000003411 | -2.90 | -7.45 | 3.94E-05 | Rab3b | RAB3B, member RAS oncogene family |
| ENSMUSG00000001763 | -2.90 | -7.45 | 1.02E-27 | Tspan33 | tetraspanin 33 |
| ENSMUSG00000022099 | -2.90 | -7.45 | 1.27E-39 | Dmtn | dematin actin binding protein |
| ENSMUSG00000081498 | -2.90 | -7.44 | 1.30E-03 | NA | NA |
| ENSMUSG00000087187 | -2.89 | -7.44 | 7.48E-18 | NA | NA |
| ENSMUSG00000058579 | -2.89 | -7.39 | 2.83E-04 | Cela2a | chymotrypsin-like elastase family, member 2A |
| ENSMUSG00000016255 | -2.88 | -7.38 | 4.77E-25 | Tubb1 | tubulin, beta 1 class VI |
| ENSMUSG00000082467 | -2.88 | -7.38 | 2.64E-21 | NA | NA |
| ENSMUSG00000047586 | -2.88 | -7.38 | 8.13E-09 | Nccrp1 | non-specific cytotoxic cell receptor protein 1 homolog (zebrafish) |
| ENSMUSG00000082912 | -2.88 | -7.37 | 4.19E-12 | NA | NA |
| ENSMUSG00000005553 | -2.88 | -7.37 | 3.37E-13 | Atp4a | ATPase, H+/K+ exchanging, gastric, alpha polypeptide |
| ENSMUSG00000046378 | -2.88 | -7.37 | 4.92E-05 | Asphd1 | aspartate beta-hydroxylase domain containing 1 |
| ENSMUSG00000028307 | -2.87 | -7.33 | 4.20E-12 | Aldob | aldolase B, fructose-bisphosphate |
| ENSMUSG00000031170 | -2.87 | -7.33 | 3.24E-20 | Slc38a5 | solute carrier family 38, member 5 |
| ENSMUSG00000063646 | -2.87 | -7.32 | 5.45E-24 | Jakmip1 | janus kinase and microtubule interacting protein 1 |
| ENSMUSG00000084081 | -2.87 | -7.31 | 9.75E-14 | NA | NA |
| ENSMUSG00000085245 | -2.87 | -7.30 | 4.88E-04 | NA | NA |
| ENSMUSG00000088231 | -2.87 | -7.29 | 7.33E-04 | NA | NA |
| ENSMUSG00000028758 | -2.87 | -7.29 | 6.45E-36 | Kif17 | kinesin family member 17 |
| ENSMUSG00000089235 | -2.86 | -7.28 | 1.63E-06 | NA | NA |
| ENSMUSG00000020992 | -2.86 | -7.28 | 5.08E-05 | NA | NA |
| ENSMUSG00000085432 | -2.86 | -7.28 | 9.56E-04 | NA | NA |
| ENSMUSG00000083012 | -2.86 | -7.28 | 7.08E-30 | NA | NA |
| ENSMUSG00000086051 | -2.86 | -7.27 | 2.03E-05 | NA | NA |
| ENSMUSG00000032023 | -2.86 | -7.26 | 2.12E-14 | 4931429I11Rik | RIKEN cDNA 4931429I11 gene |
| ENSMUSG00000006567 | -2.86 | -7.26 | 1.14E-12 | Atp7b | ATPase, Cu++ transporting, beta polypeptide |
| ENSMUSG00000041842 | -2.86 | -7.25 | 1.40E-39 | Fhdc1 | FH2 domain containing 1 |
| ENSMUSG00000025927 | -2.86 | -7.24 | 1.40E-03 | Tfap2b | transcription factor AP-2 beta |
| ENSMUSG00000048665 | -2.85 | -7.19 | 4.84E-04 | NA | NA |
| ENSMUSG00000071475 | -2.85 | -7.19 | 1.71E-03 | NA | NA |
| ENSMUSG00000084325 | -2.84 | -7.18 | 4.17E-04 | NA | NA |
| ENSMUSG00000086001 | -2.84 | -7.17 | 6.69E-04 | NA | NA |
| ENSMUSG00000032864 | -2.84 | -7.17 | 6.29E-11 | Rag2 | recombination activating gene 2 |
| ENSMUSG00000025194 | -2.84 | -7.16 | 8.73E-04 | Abcc2 | ATP-binding cassette, sub-family C (CFTR/MRP), member 2 |
| ENSMUSG00000085668 | -2.84 | -7.16 | 1.39E-03 | NA | NA |
| ENSMUSG00000033569 | -2.84 | -7.15 | 5.10E-04 | Bai3 | brain-specific angiogenesis inhibitor 3 |
| ENSMUSG00000074037 | -2.84 | -7.14 | 1.59E-04 | Mc1r | melanocortin 1 receptor |
| ENSMUSG00000087313 | -2.83 | -7.13 | 1.84E-21 | NA | NA |
| ENSMUSG00000030897 | -2.83 | -7.10 | 2.77E-07 | Cnga4 | cyclic nucleotide gated channel alpha 4 |
| ENSMUSG00000021795 | -2.83 | -7.10 | 1.30E-03 | Sftpd | surfactant associated protein D |
| ENSMUSG00000039956 | -2.83 | -7.09 | 4.46E-19 | Mrap | melanocortin 2 receptor accessory protein |
| ENSMUSG00000087660 | -2.82 | -7.09 | 9.49E-04 | NA | NA |
| ENSMUSG00000037797 | -2.82 | -7.08 | 8.29E-04 | Adh4 | alcohol dehydrogenase 4 (class II), pi polypeptide |
| ENSMUSG00000087406 | -2.82 | -7.08 | 2.11E-07 | E130215H24Rik | RIKEN cDNA E130215H24 gene |
| ENSMUSG00000071235 | -2.82 | -7.07 | 7.36E-04 | Vrtn | vertebrae development associated |
| ENSMUSG00000070000 | -2.82 | -7.06 | 3.27E-28 | Fcho1 | FCH domain only 1 |
| ENSMUSG00000014859 | -2.82 | -7.06 | 3.92E-62 | E2f4 | E2F transcription factor 4 |
| ENSMUSG00000085347 | -2.82 | -7.06 | 2.41E-20 | NA | NA |
| ENSMUSG00000034456 | -2.82 | -7.04 | 3.16E-04 | Uroc1 | urocanase domain containing 1 |
| ENSMUSG00000040657 | -2.81 | -7.02 | 2.29E-10 | 1700063H04Rik | RIKEN cDNA 1700063H04 gene |
| ENSMUSG00000054999 | -2.81 | -7.02 | 3.24E-22 | Naaladl1 | N-acetylated alpha-linked acidic dipeptidase-like 1 |
| ENSMUSG00000027510 | -2.81 | -7.01 | 3.71E-21 | Rbm38 | RNA binding motif protein 38 |
| ENSMUSG00000074143 | -2.81 | -7.00 | 2.52E-06 | NA | NA |
| ENSMUSG00000083159 | -2.80 | -6.99 | 1.52E-06 | NA | NA |
| ENSMUSG00000083933 | -2.80 | -6.98 | 1.44E-03 | NA | NA |
| ENSMUSG00000037157 | -2.80 | -6.95 | 5.53E-04 | Il22ra1 | interleukin 22 receptor, alpha 1 |
| ENSMUSG00000001930 | -2.80 | -6.95 | 3.28E-23 | Vwf | Von Willebrand factor homolog |
| ENSMUSG00000030217 | -2.80 | -6.95 | 7.52E-26 | Art4 | ADP-ribosyltransferase 4 |
| ENSMUSG00000081656 | -2.79 | -6.94 | 1.42E-19 | NA | NA |
| ENSMUSG00000060777 | -2.79 | -6.93 | 1.58E-04 | NA | NA |
| ENSMUSG00000024747 | -2.79 | -6.93 | 1.85E-18 | Aldh1a7 | aldehyde dehydrogenase family 1, subfamily A7 |
| ENSMUSG00000061436 | -2.79 | -6.92 | 2.27E-13 | Hipk2 | homeodomain interacting protein kinase 2 |
| ENSMUSG00000045259 | -2.79 | -6.91 | 4.16E-05 | Klhdc9 | kelch domain containing 9 |
| ENSMUSG00000086060 | -2.79 | -6.91 | 1.61E-03 | NA | NA |
| ENSMUSG00000070692 | -2.79 | -6.91 | 5.94E-04 | NA | NA |
| ENSMUSG00000043760 | -2.79 | -6.90 | 7.18E-04 | Pkhd1 | polycystic kidney and hepatic disease 1 |
| ENSMUSG00000080892 | -2.78 | -6.87 | 1.31E-06 | NA | NA |
| ENSMUSG00000087405 | -2.78 | -6.85 | 1.67E-03 | NA | NA |
| ENSMUSG00000030069 | -2.77 | -6.83 | 6.65E-07 | Prok2 | prokineticin 2 |
| ENSMUSG00000086743 | -2.77 | -6.83 | 2.40E-03 | NA | NA |
| ENSMUSG00000074346 | -2.77 | -6.82 | 2.32E-03 | Kcnd3os | potassium voltage-gated channel, Shal-related family, member 3, opposite strand |
| ENSMUSG00000085314 | -2.77 | -6.82 | 1.32E-04 | NA | NA |
| ENSMUSG00000018907 | -2.77 | -6.81 | 2.07E-08 | Alox12e | arachidonate lipoxygenase, epidermal |
| ENSMUSG00000082991 | -2.77 | -6.81 | 1.73E-03 | NA | NA |
| ENSMUSG00000085476 | -2.77 | -6.80 | 2.32E-03 | NA | NA |
| ENSMUSG00000082429 | -2.76 | -6.80 | 1.07E-03 | NA | NA |
| ENSMUSG00000018983 | -2.76 | -6.79 | 1.74E-27 | E2f2 | E2F transcription factor 2 |
| ENSMUSG00000031893 | -2.76 | -6.79 | 1.19E-07 | Tsnaxip1 | translin-associated factor X (Tsnax) interacting protein 1 |
| ENSMUSG00000071318 | -2.76 | -6.77 | 1.48E-03 | NA | NA |
| ENSMUSG00000014453 | -2.76 | -6.77 | 3.54E-12 | Blk | B lymphoid kinase |
| ENSMUSG00000087129 | -2.76 | -6.75 | 6.45E-05 | NA | NA |
| ENSMUSG00000032093 | -2.75 | -6.74 | 7.08E-04 | Cd3e | CD3 antigen, epsilon polypeptide |
| ENSMUSG00000005800 | -2.75 | -6.74 | 2.97E-17 | Mmp8 | matrix metallopeptidase 8 |
| ENSMUSG00000073002 | -2.75 | -6.74 | 1.22E-03 | Vamp5 | vesicle-associated membrane protein 5 |
| ENSMUSG00000083385 | -2.75 | -6.74 | 2.19E-03 | NA | NA |
| ENSMUSG00000025270 | -2.75 | -6.73 | 1.15E-22 | Alas2 | aminolevulinic acid synthase 2, erythroid |
| ENSMUSG00000067526 | -2.75 | -6.73 | 1.70E-03 | Olfr1425 | olfactory receptor 1425 |
| ENSMUSG00000023995 | -2.75 | -6.71 | 1.45E-29 | Tspo2 | translocator protein 2 |
| ENSMUSG00000074199 | -2.75 | -6.70 | 1.46E-03 | Krtdap | keratinocyte differentiation associated protein |
| ENSMUSG00000058216 | -2.74 | -6.70 | 1.20E-24 | NA | NA |
| ENSMUSG00000038252 | -2.74 | -6.69 | 4.91E-46 | Ncapd2 | non-SMC condensin I complex, subunit D2 |
| ENSMUSG00000058045 | -2.74 | -6.69 | 1.30E-13 | NA | NA |
| ENSMUSG00000088356 | -2.74 | -6.68 | 9.71E-04 | NA | NA |
| ENSMUSG00000037979 | -2.74 | -6.68 | 7.98E-16 | Ccdc92 | coiled-coil domain containing 92 |
| ENSMUSG00000027907 | -2.74 | -6.68 | 6.67E-14 | NA | NA |
| ENSMUSG00000080957 | -2.74 | -6.68 | 9.85E-04 | NA | NA |
| ENSMUSG00000047495 | -2.74 | -6.68 | 3.36E-10 | Dlgap2 | discs, large (Drosophila) homolog-associated protein 2 |
| ENSMUSG00000066687 | -2.74 | -6.67 | 7.96E-12 | Zbtb16 | zinc finger and BTB domain containing 16 |
| ENSMUSG00000024206 | -2.74 | -6.66 | 4.03E-34 | Rfx2 | regulatory factor X, 2 (influences HLA class II expression) |
| ENSMUSG00000068601 | -2.73 | -6.65 | 5.17E-09 | NA | NA |
| ENSMUSG00000043286 | -2.73 | -6.65 | 1.61E-09 | Pnpla1 | patatin-like phospholipase domain containing 1 |
| ENSMUSG00000076468 | -2.73 | -6.63 | 6.53E-04 | NA | NA |
| ENSMUSG00000086765 | -2.73 | -6.63 | 3.57E-18 | Gm11827 | predicted gene 11827 |
| ENSMUSG00000056370 | -2.73 | -6.63 | 1.12E-06 | Sftpb | surfactant associated protein B |
| ENSMUSG00000068302 | -2.73 | -6.62 | 2.80E-03 | Noto | notochord homolog (Xenopus laevis) |
| ENSMUSG00000028393 | -2.72 | -6.59 | 1.37E-33 | Alad | aminolevulinate, delta-, dehydratase |
| ENSMUSG00000032591 | -2.72 | -6.59 | 5.38E-19 | Mst1 | macrophage stimulating 1 (hepatocyte growth factor-like) |
| ENSMUSG00000037001 | -2.72 | -6.58 | 1.29E-30 | Zfp39 | zinc finger protein 39 |
| ENSMUSG00000034918 | -2.71 | -6.57 | 1.79E-03 | Cdhr2 | cadherin-related family member 2 |
| ENSMUSG00000028716 | -2.71 | -6.56 | 1.18E-20 | Pdzk1ip1 | PDZK1 interacting protein 1 |
| ENSMUSG00000037124 | -2.71 | -6.55 | 1.67E-13 | Trim58 | tripartite motif-containing 58 |
| ENSMUSG00000020125 | -2.71 | -6.55 | 5.38E-17 | Elane | elastase, neutrophil expressed |
| ENSMUSG00000062859 | -2.71 | -6.55 | 3.97E-08 | Tcp11 | t-complex protein 11 |
| ENSMUSG00000061615 | -2.71 | -6.55 | 4.54E-04 | NA | NA |
| ENSMUSG00000087096 | -2.71 | -6.55 | 1.83E-03 | NA | NA |
| ENSMUSG00000035246 | -2.71 | -6.54 | 3.69E-25 | Pcyt1b | phosphate cytidylyltransferase 1, choline, beta isoform |
| ENSMUSG00000028906 | -2.70 | -6.52 | 1.22E-31 | Epb4.1 | erythrocyte protein band 4.1 |
| ENSMUSG00000070354 | -2.70 | -6.51 | 2.45E-23 | NA | NA |
| ENSMUSG00000028081 | -2.70 | -6.51 | 2.83E-32 | Rps3a1 | ribosomal protein S3A1 |
| ENSMUSG00000082458 | -2.70 | -6.50 | 1.26E-20 | NA | NA |
| ENSMUSG00000051378 | -2.70 | -6.50 | 1.88E-43 | Kif18b | kinesin family member 18B |
| ENSMUSG00000079173 | -2.70 | -6.49 | 1.14E-07 | Zan | zonadhesin |
| ENSMUSG00000083907 | -2.70 | -6.49 | 3.20E-20 | NA | NA |
| ENSMUSG00000066244 | -2.70 | -6.49 | 2.43E-03 | NA | NA |
| ENSMUSG00000076613 | -2.70 | -6.48 | 1.05E-03 | NA | NA |
| ENSMUSG00000024008 | -2.69 | -6.47 | 3.87E-08 | Cpne5 | copine V |
| ENSMUSG00000063162 | -2.69 | -6.46 | 1.93E-03 | NA | NA |
| ENSMUSG00000085315 | -2.69 | -6.44 | 1.35E-03 | NA | NA |
| ENSMUSG00000083200 | -2.69 | -6.43 | 1.66E-03 | NA | NA |
| ENSMUSG00000052303 | -2.68 | -6.43 | 2.92E-03 | Mrgpra6 | MAS-related GPR, member A6 |
| ENSMUSG00000032739 | -2.68 | -6.42 | 2.57E-23 | Pram1 | PML-RAR alpha-regulated adaptor molecule 1 |
| ENSMUSG00000082509 | -2.68 | -6.42 | 2.35E-10 | NA | NA |
| ENSMUSG00000026532 | -2.68 | -6.42 | 1.19E-16 | Spta1 | spectrin alpha, erythrocytic 1 |
| ENSMUSG00000000632 | -2.68 | -6.41 | 1.41E-04 | Sez6 | seizure related gene 6 |
| ENSMUSG00000073439 | -2.68 | -6.40 | 2.52E-03 | NA | NA |
| ENSMUSG00000082000 | -2.68 | -6.40 | 3.13E-03 | NA | NA |
| ENSMUSG00000049460 | -2.68 | -6.39 | 2.99E-16 | NA | NA |
| ENSMUSG00000015970 | -2.67 | -6.38 | 1.29E-32 | Chdh | choline dehydrogenase |
| ENSMUSG00000039533 | -2.67 | -6.38 | 6.58E-04 | Mmd2 | monocyte to macrophage differentiation-associated 2 |
| ENSMUSG00000090077 | -2.67 | -6.36 | 7.31E-05 | Lime1 | Lck interacting transmembrane adaptor 1 |
| ENSMUSG00000083285 | -2.67 | -6.36 | 2.95E-03 | NA | NA |
| ENSMUSG00000018168 | -2.67 | -6.36 | 3.20E-20 | Ikzf3 | IKAROS family zinc finger 3 |
| ENSMUSG00000027233 | -2.67 | -6.34 | 2.26E-04 | Patl2 | protein associated with topoisomerase II homolog 2 (yeast) |
| ENSMUSG00000083574 | -2.66 | -6.34 | 3.36E-03 | NA | NA |
| ENSMUSG00000059033 | -2.66 | -6.34 | 4.34E-06 | NA | NA |
| ENSMUSG00000023216 | -2.66 | -6.32 | 1.17E-15 | Epb4.2 | erythrocyte protein band 4.2 |
| ENSMUSG00000044165 | -2.66 | -6.32 | 1.39E-14 | Bcl2l15 | BCLl2-like 15 |
| ENSMUSG00000087131 | -2.66 | -6.31 | 1.15E-03 | NA | NA |
| ENSMUSG00000022218 | -2.66 | -6.31 | 1.81E-33 | Tgm1 | transglutaminase 1, K polypeptide |
| ENSMUSG00000033872 | -2.66 | -6.30 | 2.62E-03 | NA | NA |
| ENSMUSG00000074625 | -2.65 | -6.30 | 1.03E-03 | Arhgap40 | Rho GTPase activating protein 40 |
| ENSMUSG00000038745 | -2.65 | -6.30 | 1.25E-13 | Nlrp6 | NLR family, pyrin domain containing 6 |
| ENSMUSG00000055385 | -2.65 | -6.29 | 6.44E-15 | Rnf212 | ring finger protein 212 |
| ENSMUSG00000084012 | -2.65 | -6.28 | 2.92E-03 | NA | NA |
| ENSMUSG00000027360 | -2.65 | -6.28 | 1.81E-17 | Hdc | histidine decarboxylase |
| ENSMUSG00000053664 | -2.65 | -6.27 | 1.45E-27 | NA | NA |
| ENSMUSG00000067596 | -2.65 | -6.26 | 2.23E-03 | Krt74 | keratin 74 |
| ENSMUSG00000025014 | -2.64 | -6.25 | 1.77E-19 | Dntt | deoxynucleotidyltransferase, terminal |
| ENSMUSG00000031698 | -2.64 | -6.24 | 1.26E-20 | Mylk3 | myosin light chain kinase 3 |
| ENSMUSG00000020490 | -2.64 | -6.24 | 1.22E-16 | Btnl10 | butyrophilin-like 10 |
| ENSMUSG00000036264 | -2.64 | -6.23 | 9.80E-07 | Fstl4 | follistatin-like 4 |
| ENSMUSG00000029516 | -2.64 | -6.23 | 7.33E-25 | Cit | citron |
| ENSMUSG00000032131 | -2.64 | -6.22 | 6.68E-24 | Abcg4 | ATP-binding cassette, sub-family G (WHITE), member 4 |
| ENSMUSG00000087371 | -2.63 | -6.21 | 7.43E-09 | NA | NA |
| ENSMUSG00000005410 | -2.63 | -6.21 | 5.33E-38 | Mcm5 | minichromosome maintenance deficient 5, cell division cycle 46 (S. cerevisiae) |
| ENSMUSG00000037544 | -2.63 | -6.19 | 1.22E-19 | Dlgap5 | discs, large (Drosophila) homolog-associated protein 5 |
| ENSMUSG00000084183 | -2.63 | -6.19 | 1.94E-08 | NA | NA |
| ENSMUSG00000086046 | -2.63 | -6.18 | 4.26E-03 | 1700095A21Rik | RIKEN cDNA 1700095A21 gene |
| ENSMUSG00000075370 | -2.63 | -6.18 | 1.64E-15 | Igll1 | immunoglobulin lambda-like polypeptide 1 |
| ENSMUSG00000041859 | -2.63 | -6.17 | 2.41E-41 | Mcm3 | minichromosome maintenance deficient 3 (S. cerevisiae) |
| ENSMUSG00000020878 | -2.63 | -6.17 | 1.71E-05 | Lrrc46 | leucine rich repeat containing 46 |
| ENSMUSG00000066760 | -2.62 | -6.16 | 1.70E-03 | Psg16 | pregnancy specific glycoprotein 16 |
| ENSMUSG00000025023 | -2.62 | -6.16 | 4.24E-07 | NA | NA |
| ENSMUSG00000087581 | -2.62 | -6.15 | 3.35E-03 | LOC102635562 | uncharacterized LOC102635562 |
| ENSMUSG00000018924 | -2.62 | -6.14 | 2.92E-19 | Alox15 | arachidonate 15-lipoxygenase |
| ENSMUSG00000034427 | -2.62 | -6.14 | 1.61E-13 | NA | NA |
| ENSMUSG00000030724 | -2.61 | -6.13 | 9.76E-16 | Cd19 | CD19 antigen |
| ENSMUSG00000038871 | -2.61 | -6.12 | 7.04E-29 | Bpgm | 2,3-bisphosphoglycerate mutase |
| ENSMUSG00000087377 | -2.61 | -6.12 | 1.47E-10 | NA | NA |
| ENSMUSG00000040340 | -2.61 | -6.11 | 7.17E-04 | 1700019B03Rik | RIKEN cDNA 1700019B03 gene |
| ENSMUSG00000035638 | -2.61 | -6.11 | 1.80E-13 | Muc20 | mucin 20 |
| ENSMUSG00000081869 | -2.61 | -6.10 | 3.37E-03 | NA | NA |
| ENSMUSG00000051457 | -2.61 | -6.09 | 9.06E-51 | Spn | sialophorin |
| ENSMUSG00000000861 | -2.61 | -6.09 | 2.10E-25 | Bcl11a | B cell CLL/lymphoma 11A (zinc finger protein) |
| ENSMUSG00000077680 | -2.61 | -6.09 | 4.59E-03 | NA | NA |
| ENSMUSG00000062148 | -2.61 | -6.09 | 2.21E-20 | NA | NA |
| ENSMUSG00000089653 | -2.61 | -6.09 | 3.77E-03 | NA | NA |
| ENSMUSG00000052234 | -2.60 | -6.08 | 1.27E-17 | Epx | eosinophil peroxidase |
| ENSMUSG00000069917 | -2.60 | -6.08 | 4.35E-11 | NA | NA |
| ENSMUSG00000085579 | -2.60 | -6.08 | 1.54E-03 | NA | NA |
| ENSMUSG00000040009 | -2.60 | -6.07 | 1.31E-20 | Gnaz | guanine nucleotide binding protein, alpha z subunit |
| ENSMUSG00000075511 | -2.60 | -6.07 | 2.82E-11 | NA | NA |
| ENSMUSG00000073607 | -2.60 | -6.07 | 3.17E-03 | Gm10548 | ribosomal protein L29 pseudogene |
| ENSMUSG00000002068 | -2.60 | -6.06 | 7.87E-29 | Ccne1 | cyclin E1 |
| ENSMUSG00000082974 | -2.60 | -6.06 | 1.34E-03 | NA | NA |
| ENSMUSG00000036377 | -2.60 | -6.05 | 1.21E-15 | C530008M17Rik | RIKEN cDNA C530008M17 gene |
| ENSMUSG00000055298 | -2.59 | -6.04 | 3.26E-03 | Ctsj | cathepsin J |
| ENSMUSG00000063409 | -2.59 | -6.04 | 3.36E-04 | Lrrc43 | leucine rich repeat containing 43 |
| ENSMUSG00000073554 | -2.59 | -6.04 | 4.27E-14 | NA | NA |
| ENSMUSG00000086091 | -2.59 | -6.04 | 2.25E-03 | NA | NA |
| ENSMUSG00000085788 | -2.59 | -6.03 | 3.30E-03 | NA | NA |
| ENSMUSG00000020396 | -2.59 | -6.03 | 6.92E-16 | Nefh | neurofilament, heavy polypeptide |
| ENSMUSG00000082906 | -2.59 | -6.02 | 4.49E-15 | NA | NA |
| ENSMUSG00000081734 | -2.59 | -6.01 | 4.51E-03 | NA | NA |
| ENSMUSG00000059864 | -2.58 | -5.99 | 3.57E-04 | Olfr1393 | olfactory receptor 1393 |
| ENSMUSG00000029878 | -2.58 | -5.98 | 2.76E-03 | Dbpht2 | DNA binding protein with his-thr domain |
| ENSMUSG00000042489 | -2.58 | -5.97 | 4.42E-19 | Clspn | claspin |
| ENSMUSG00000084300 | -2.58 | -5.97 | 6.03E-06 | NA | NA |
| ENSMUSG00000082852 | -2.58 | -5.97 | 2.77E-03 | NA | NA |
| ENSMUSG00000078553 | -2.58 | -5.96 | 1.90E-03 | NA | NA |
| ENSMUSG00000071528 | -2.57 | -5.95 | 6.32E-07 | Usmg5 | upregulated during skeletal muscle growth 5 |
| ENSMUSG00000021675 | -2.57 | -5.95 | 1.09E-25 | F2rl2 | coagulation factor II (thrombin) receptor-like 2 |
| ENSMUSG00000029711 | -2.57 | -5.94 | 3.13E-03 | Epo | erythropoietin |
| ENSMUSG00000031162 | -2.57 | -5.94 | 3.71E-25 | Gata1 | GATA binding protein 1 |
| ENSMUSG00000037143 | -2.56 | -5.91 | 7.67E-04 | 4930529M08Rik | RIKEN cDNA 4930529M08 gene |
| ENSMUSG00000087687 | -2.56 | -5.91 | 2.43E-41 | Pet100 | PET100 homolog (S. cerevisiae) |
| ENSMUSG00000085915 | -2.56 | -5.90 | 2.92E-09 | NA | NA |
| ENSMUSG00000084338 | -2.56 | -5.90 | 2.35E-06 | NA | NA |
| ENSMUSG00000058211 | -2.56 | -5.90 | 4.02E-03 | NA | NA |
| ENSMUSG00000022311 | -2.56 | -5.90 | 1.95E-03 | Csmd3 | CUB and Sushi multiple domains 3 |
| ENSMUSG00000081233 | -2.56 | -5.89 | 1.15E-03 | NA | NA |
| ENSMUSG00000082688 | -2.56 | -5.88 | 4.57E-03 | NA | NA |
| ENSMUSG00000074779 | -2.55 | -5.87 | 7.96E-08 | NA | NA |
| ENSMUSG00000073006 | -2.55 | -5.87 | 4.50E-03 | Gm732 | predicted gene 732 |
| ENSMUSG00000074497 | -2.55 | -5.86 | 2.03E-08 | A430078G23Rik | RIKEN cDNA A430078G23 gene |
| ENSMUSG00000010154 | -2.55 | -5.86 | 2.39E-11 | Spire2 | spire homolog 2 (Drosophila) |
| ENSMUSG00000022824 | -2.55 | -5.86 | 7.60E-19 | Muc13 | mucin 13, epithelial transmembrane |
| ENSMUSG00000023993 | -2.55 | -5.85 | 2.52E-18 | Treml1 | triggering receptor expressed on myeloid cells-like 1 |
| ENSMUSG00000027072 | -2.55 | -5.85 | 2.02E-14 | Prg3 | proteoglycan 3 |
| ENSMUSG00000073414 | -2.55 | -5.84 | 3.21E-20 | G6b | immunoreceptor tyrosine-based inhibitory motif (ITIM) containing platelet receptor |
| ENSMUSG00000084126 | -2.55 | -5.84 | 4.54E-03 | NA | NA |
| ENSMUSG00000036718 | -2.54 | -5.83 | 2.50E-50 | Micall2 | MICAL-like 2 |
| ENSMUSG00000001156 | -2.54 | -5.83 | 4.30E-32 | Mxd1 | MAX dimerization protein 1 |
| ENSMUSG00000026355 | -2.54 | -5.83 | 1.59E-32 | Mcm6 | minichromosome maintenance deficient 6 (MIS5 homolog, S. pombe) (S. cerevisiae) |
| ENSMUSG00000085264 | -2.54 | -5.81 | 4.50E-03 | LOC102631779 | uncharacterized LOC102631779 |
| ENSMUSG00000019368 | -2.54 | -5.81 | 1.26E-03 | Sec14l4 | SEC14-like 4 (S. cerevisiae) |
| ENSMUSG00000004328 | -2.54 | -5.80 | 4.01E-09 | Hif3a | hypoxia inducible factor 3, alpha subunit |
| ENSMUSG00000030340 | -2.54 | -5.80 | 1.81E-22 | Scnn1a | sodium channel, nonvoltage-gated 1 alpha |
| ENSMUSG00000084303 | -2.54 | -5.80 | 1.33E-05 | NA | NA |
| ENSMUSG00000027168 | -2.54 | -5.80 | 3.75E-04 | Pax6 | paired box 6 |
| ENSMUSG00000027556 | -2.54 | -5.80 | 9.05E-20 | Car1 | carbonic anhydrase 1 |
| ENSMUSG00000073067 | -2.53 | -5.79 | 2.28E-13 | 9130019P16Rik | RIKEN cDNA 9130019P16 gene |
| ENSMUSG00000086324 | -2.53 | -5.79 | 3.06E-06 | LOC102639045 | uncharacterized LOC102639045 |
| ENSMUSG00000029423 | -2.53 | -5.79 | 5.45E-03 | Piwil1 | piwi-like RNA-mediated gene silencing 1 |
| ENSMUSG00000082122 | -2.53 | -5.79 | 6.11E-05 | NA | NA |
| ENSMUSG00000078091 | -2.53 | -5.79 | 2.10E-03 | NA | NA |
| ENSMUSG00000074354 | -2.53 | -5.78 | 1.07E-06 | Arhgap20os | Rho GTPase activating protein 20, opposite strand |
| ENSMUSG00000044456 | -2.53 | -5.78 | 3.76E-53 | Rin3 | Ras and Rab interactor 3 |
| ENSMUSG00000082046 | -2.53 | -5.78 | 3.25E-03 | NA | NA |
| ENSMUSG00000034209 | -2.53 | -5.78 | 9.75E-10 | Rasl10a | RAS-like, family 10, member A |
| ENSMUSG00000042333 | -2.53 | -5.78 | 7.92E-23 | Tnfrsf14 | tumor necrosis factor receptor superfamily, member 14 (herpesvirus entry mediator) |
| ENSMUSG00000058700 | -2.53 | -5.77 | 5.50E-03 | NA | NA |
| ENSMUSG00000083264 | -2.53 | -5.77 | 5.33E-03 | NA | NA |
| ENSMUSG00000031822 | -2.53 | -5.77 | 4.78E-12 | Gse1 | genetic suppressor element 1 |
| ENSMUSG00000022861 | -2.53 | -5.77 | 1.33E-15 | Dgkg | diacylglycerol kinase, gamma |
| ENSMUSG00000024184 | -2.53 | -5.76 | 5.90E-13 | Pdia2 | protein disulfide isomerase associated 2 |
| ENSMUSG00000033762 | -2.52 | -5.75 | 1.26E-28 | Recql4 | RecQ protein-like 4 |
| ENSMUSG00000087457 | -2.52 | -5.75 | 1.78E-03 | NA | NA |
| ENSMUSG00000045382 | -2.52 | -5.75 | 2.56E-47 | Cxcr4 | chemokine (C-X-C motif) receptor 4 |
| ENSMUSG00000085483 | -2.52 | -5.74 | 6.10E-07 | NA | NA |
| ENSMUSG00000010760 | -2.52 | -5.74 | 9.77E-04 | Phlda2 | pleckstrin homology-like domain, family A, member 2 |
| ENSMUSG00000059280 | -2.52 | -5.74 | 1.90E-08 | Vpreb2 | pre-B lymphocyte gene 2 |
| ENSMUSG00000036961 | -2.52 | -5.73 | 2.93E-03 | Wnt8b | wingless related MMTV integration site 8b |
| ENSMUSG00000074277 | -2.52 | -5.73 | 1.58E-31 | Phldb3 | pleckstrin homology-like domain, family B, member 3 |
| ENSMUSG00000042351 | -2.51 | -5.72 | 4.12E-26 | Grap2 | GRB2-related adaptor protein 2 |
| ENSMUSG00000021750 | -2.51 | -5.71 | 7.59E-13 | Fam107a | family with sequence similarity 107, member A |
| ENSMUSG00000018973 | -2.51 | -5.71 | 1.26E-03 | Hoxb1 | homeobox B1 |
| ENSMUSG00000035472 | -2.51 | -5.71 | 1.15E-17 | Slc25a21 | solute carrier family 25 (mitochondrial oxodicarboxylate carrier), member 21 |
| ENSMUSG00000019214 | -2.51 | -5.71 | 2.33E-32 | Chtf18 | CTF18, chromosome transmission fidelity factor 18 |
| ENSMUSG00000074575 | -2.51 | -5.70 | 4.13E-20 | Kcng1 | potassium voltage-gated channel, subfamily G, member 1 |
| ENSMUSG00000044927 | -2.51 | -5.70 | 2.35E-14 | H1fx | H1 histone family, member X |
| ENSMUSG00000034449 | -2.51 | -5.69 | 1.33E-23 | Dhrs11 | dehydrogenase/reductase (SDR family) member 11 |
| ENSMUSG00000072082 | -2.51 | -5.69 | 7.64E-32 | Ccnf | cyclin F |
| ENSMUSG00000069274 | -2.51 | -5.69 | 3.38E-04 | NA | NA |
| ENSMUSG00000039055 | -2.51 | -5.69 | 1.89E-27 | Eme1 | essential meiotic endonuclease 1 homolog 1 (S. pombe) |
| ENSMUSG00000040435 | -2.51 | -5.68 | 2.58E-32 | Ppp1r15a | protein phosphatase 1, regulatory (inhibitor) subunit 15A |
| ENSMUSG00000072629 | -2.51 | -5.68 | 1.35E-16 | NA | NA |
| ENSMUSG00000017718 | -2.50 | -5.67 | 3.80E-42 | Afmid | arylformamidase |
| ENSMUSG00000016552 | -2.50 | -5.67 | 6.86E-17 | Foxred2 | FAD-dependent oxidoreductase domain containing 2 |
| ENSMUSG00000037705 | -2.50 | -5.67 | 1.33E-05 | Tecta | tectorin alpha |
| ENSMUSG00000086820 | -2.50 | -5.66 | 1.70E-03 | NA | NA |
| ENSMUSG00000051054 | -2.50 | -5.66 | 1.43E-05 | NA | NA |
| ENSMUSG00000086446 | -2.50 | -5.65 | 1.08E-05 | Prkag2os1 | protein kinase, AMP-activated, gamma 2 non-catalytic subunit, opposite strand 1 |
| ENSMUSG00000046634 | -2.50 | -5.64 | 1.45E-14 | Pkd1l1 | polycystic kidney disease 1 like 1 |
| ENSMUSG00000022876 | -2.49 | -5.64 | 2.01E-13 | Samsn1 | SAM domain, SH3 domain and nuclear localization signals, 1 |
| ENSMUSG00000035459 | -2.49 | -5.63 | 3.37E-16 | Stab2 | stabilin 2 |
| ENSMUSG00000047324 | -2.49 | -5.63 | 6.93E-03 | 4931429P17Rik | RIKEN cDNA 4931429P17 gene |
| ENSMUSG00000086891 | -2.49 | -5.63 | 2.67E-03 | NA | NA |
| ENSMUSG00000048217 | -2.49 | -5.63 | 1.24E-03 | Nags | N-acetylglutamate synthase |
| ENSMUSG00000041872 | -2.49 | -5.63 | 2.80E-03 | Il17f | interleukin 17F |
| ENSMUSG00000087139 | -2.49 | -5.63 | 2.47E-03 | LOC102631682 | uncharacterized LOC102631682 |
| ENSMUSG00000046006 | -2.49 | -5.63 | 1.01E-16 | Gapt | Grb2-binding adaptor, transmembrane |
| ENSMUSG00000027330 | -2.49 | -5.62 | 4.96E-32 | Cdc25b | cell division cycle 25B |
| ENSMUSG00000060929 | -2.49 | -5.61 | 3.29E-10 | NA | NA |
| ENSMUSG00000017861 | -2.49 | -5.61 | 3.34E-30 | Mybl2 | myeloblastosis oncogene-like 2 |
| ENSMUSG00000085204 | -2.49 | -5.61 | 8.78E-05 | NA | NA |
| ENSMUSG00000032028 | -2.49 | -5.60 | 1.22E-14 | Nxpe2 | neurexophilin and PC-esterase domain family, member 2 |
| ENSMUSG00000080731 | -2.48 | -5.60 | 9.24E-04 | NA | NA |
| ENSMUSG00000024353 | -2.48 | -5.59 | 1.50E-14 | Mzb1 | marginal zone B and B1 cell-specific protein 1 |
| ENSMUSG00000063232 | -2.48 | -5.58 | 7.22E-06 | Serpina11 | serine (or cysteine) peptidase inhibitor, clade A (alpha-1 antiproteinase, antitrypsin), member 11 |
| ENSMUSG00000085671 | -2.48 | -5.58 | 3.71E-12 | NA | NA |
| ENSMUSG00000082660 | -2.48 | -5.57 | 2.31E-03 | NA | NA |
| ENSMUSG00000000320 | -2.48 | -5.57 | 8.87E-22 | Alox12 | arachidonate 12-lipoxygenase |
| ENSMUSG00000038473 | -2.48 | -5.56 | 7.40E-14 | Nos1ap | nitric oxide synthase 1 (neuronal) adaptor protein |
| ENSMUSG00000032269 | -2.47 | -5.55 | 4.24E-03 | Htr3a | 5-hydroxytryptamine (serotonin) receptor 3A |
| ENSMUSG00000082792 | -2.47 | -5.55 | 1.77E-03 | NA | NA |
| ENSMUSG00000035910 | -2.47 | -5.55 | 7.53E-03 | Dcdc2a | doublecortin domain containing 2a |
| ENSMUSG00000063398 | -2.47 | -5.55 | 8.83E-04 | NA | NA |
| ENSMUSG00000084064 | -2.47 | -5.54 | 1.08E-04 | NA | NA |
| ENSMUSG00000078925 | -2.47 | -5.54 | 7.27E-03 | NA | NA |
| ENSMUSG00000063903 | -2.47 | -5.54 | 3.04E-14 | Klk1 | kallikrein 1 |
| ENSMUSG00000076699 | -2.47 | -5.54 | 1.05E-04 | NA | NA |
| ENSMUSG00000019312 | -2.47 | -5.54 | 4.15E-06 | Grb7 | growth factor receptor bound protein 7 |
| ENSMUSG00000059434 | -2.47 | -5.53 | 4.46E-03 | Gckr | glucokinase regulatory protein |
| ENSMUSG00000036899 | -2.47 | -5.53 | 6.27E-06 | Trpv5 | transient receptor potential cation channel, subfamily V, member 5 |
| ENSMUSG00000060985 | -2.47 | -5.52 | 2.81E-03 | Tdrd5 | tudor domain containing 5 |
| ENSMUSG00000023015 | -2.46 | -5.52 | 2.87E-50 | Racgap1 | Rac GTPase-activating protein 1 |
| ENSMUSG00000050106 | -2.46 | -5.52 | 1.08E-22 | Tmc8 | transmembrane channel-like gene family 8 |
| ENSMUSG00000082145 | -2.46 | -5.52 | 2.17E-13 | NA | NA |
| ENSMUSG00000055717 | -2.46 | -5.51 | 7.63E-18 | Slain1 | SLAIN motif family, member 1 |
| ENSMUSG00000090030 | -2.46 | -5.51 | 6.93E-11 | NA | NA |
| ENSMUSG00000052212 | -2.46 | -5.51 | 1.45E-19 | Cd177 | CD177 antigen |
| ENSMUSG00000038893 | -2.46 | -5.51 | 3.84E-25 | Fam117a | family with sequence similarity 117, member A |
| ENSMUSG00000068876 | -2.46 | -5.50 | 9.14E-10 | Cgn | cingulin |
| ENSMUSG00000019982 | -2.46 | -5.50 | 1.45E-20 | Myb | myeloblastosis oncogene |
| ENSMUSG00000040231 | -2.46 | -5.50 | 6.11E-08 | Syngr4 | synaptogyrin 4 |
| ENSMUSG00000086705 | -2.46 | -5.50 | 4.70E-05 | NA | NA |
| ENSMUSG00000062046 | -2.46 | -5.50 | 3.54E-05 | 5730460C07Rik | RIKEN cDNA 5730460C07 gene |
| ENSMUSG00000001865 | -2.46 | -5.49 | 3.25E-12 | Cpa3 | carboxypeptidase A3, mast cell |
| ENSMUSG00000076004 | -2.46 | -5.49 | 9.57E-04 | NA | NA |
| ENSMUSG00000023443 | -2.46 | -5.49 | 5.68E-03 | Esx1 | extraembryonic, spermatogenesis, homeobox 1 |
| ENSMUSG00000028661 | -2.45 | -5.48 | 2.24E-10 | Epha8 | Eph receptor A8 |
| ENSMUSG00000081888 | -2.45 | -5.47 | 1.71E-15 | NA | NA |
| ENSMUSG00000085118 | -2.45 | -5.47 | 6.19E-12 | NA | NA |
| ENSMUSG00000085549 | -2.45 | -5.47 | 3.55E-03 | NA | NA |
| ENSMUSG00000081208 | -2.45 | -5.46 | 3.61E-20 | NA | NA |
| ENSMUSG00000086449 | -2.45 | -5.46 | 4.92E-03 | 9030204H09Rik | RIKEN cDNA 9030204H09 gene |
| ENSMUSG00000027715 | -2.45 | -5.46 | 3.22E-27 | Ccna2 | cyclin A2 |
| ENSMUSG00000050600 | -2.45 | -5.45 | 2.12E-12 | Zfp831 | zinc finger protein 831 |
| ENSMUSG00000074783 | -2.45 | -5.45 | 1.94E-04 | AU019990 | expressed sequence AU019990 |
| ENSMUSG00000082014 | -2.44 | -5.44 | 8.20E-03 | NA | NA |
| ENSMUSG00000031766 | -2.44 | -5.44 | 3.24E-05 | Slc12a3 | solute carrier family 12, member 3 |
| ENSMUSG00000079644 | -2.44 | -5.44 | 2.26E-03 | Gm1110 | predicted gene 1110 |
| ENSMUSG00000028544 | -2.44 | -5.43 | 7.24E-04 | Slc5a9 | solute carrier family 5 (sodium/glucose cotransporter), member 9 |
| ENSMUSG00000043110 | -2.44 | -5.43 | 1.07E-05 | Lrrn4 | leucine rich repeat neuronal 4 |
| ENSMUSG00000032484 | -2.44 | -5.43 | 1.03E-15 | Ngp | neutrophilic granule protein |
| ENSMUSG00000090115 | -2.44 | -5.43 | 3.51E-32 | Usp49 | ubiquitin specific peptidase 49 |
| ENSMUSG00000002033 | -2.44 | -5.42 | 4.72E-04 | Cd3g | CD3 antigen, gamma polypeptide |
| ENSMUSG00000031877 | -2.44 | -5.42 | 8.58E-26 | Ces2g | carboxylesterase 2G |
| ENSMUSG00000080775 | -2.44 | -5.41 | 5.72E-06 | NA | NA |
| ENSMUSG00000081310 | -2.43 | -5.40 | 8.53E-03 | NA | NA |
| ENSMUSG00000046541 | -2.43 | -5.40 | 1.64E-30 | Zfp526 | zinc finger protein 526 |
| ENSMUSG00000020788 | -2.43 | -5.40 | 3.63E-25 | Atp2a3 | ATPase, Ca++ transporting, ubiquitous |
| ENSMUSG00000083940 | -2.43 | -5.39 | 8.49E-03 | NA | NA |
| ENSMUSG00000030209 | -2.43 | -5.39 | 6.56E-03 | Grin2b | glutamate receptor, ionotropic, NMDA2B (epsilon 2) |
| ENSMUSG00000046213 | -2.43 | -5.39 | 1.21E-03 | Cym | chymosin |
| ENSMUSG00000083424 | -2.43 | -5.39 | 7.22E-03 | NA | NA |
| ENSMUSG00000028332 | -2.43 | -5.39 | 5.91E-17 | Hemgn | hemogen |
| ENSMUSG00000048080 | -2.43 | -5.39 | 7.85E-03 | Olfr731 | olfactory receptor 731 |
| ENSMUSG00000025396 | -2.43 | -5.38 | 6.38E-03 | Hsd17b6 | hydroxysteroid (17-beta) dehydrogenase 6 |
| ENSMUSG00000083338 | -2.43 | -5.38 | 3.74E-09 | NA | NA |
| ENSMUSG00000020892 | -2.43 | -5.38 | 1.06E-03 | Aloxe3 | arachidonate lipoxygenase 3 |
| ENSMUSG00000021553 | -2.43 | -5.38 | 3.81E-06 | Slc28a3 | solute carrier family 28 (sodium-coupled nucleoside transporter), member 3 |
| ENSMUSG00000076931 | -2.43 | -5.38 | 8.66E-06 | NA | NA |
| ENSMUSG00000073413 | -2.43 | -5.38 | 1.57E-07 | Ly6g6d | lymphocyte antigen 6 complex, locus G6D |
| ENSMUSG00000068399 | -2.43 | -5.37 | 8.84E-03 | NA | NA |
| ENSMUSG00000026614 | -2.43 | -5.37 | 5.51E-14 | Slc30a10 | solute carrier family 30, member 10 |
| ENSMUSG00000048148 | -2.42 | -5.37 | 3.02E-10 | Nwd1 | NACHT and WD repeat domain containing 1 |
| ENSMUSG00000086943 | -2.42 | -5.36 | 9.49E-05 | 4732414G09Rik | RIKEN cDNA 4732414G09 gene |
| ENSMUSG00000027562 | -2.42 | -5.35 | 2.76E-20 | Car2 | carbonic anhydrase 2 |
| ENSMUSG00000025889 | -2.42 | -5.35 | 1.37E-16 | Snca | synuclein, alpha |
| ENSMUSG00000029359 | -2.42 | -5.35 | 1.57E-17 | Tesc | tescalcin |
| ENSMUSG00000086617 | -2.42 | -5.34 | 8.36E-03 | NA | NA |
| ENSMUSG00000006711 | -2.42 | -5.33 | 1.20E-03 | D130043K22Rik | RIKEN cDNA D130043K22 gene |
| ENSMUSG00000030091 | -2.41 | -5.33 | 1.73E-16 | Nup210 | nucleoporin 210 |
| ENSMUSG00000051428 | -2.41 | -5.33 | 7.46E-12 | NA | NA |
| ENSMUSG00000039196 | -2.41 | -5.33 | 1.42E-11 | Orm1 | orosomucoid 1 |
| ENSMUSG00000002870 | -2.41 | -5.33 | 1.01E-31 | Mcm2 | minichromosome maintenance deficient 2 mitotin (S. cerevisiae) |
| ENSMUSG00000004948 | -2.41 | -5.33 | 1.25E-03 | Zp3 | zona pellucida glycoprotein 3 |
| ENSMUSG00000081647 | -2.41 | -5.33 | 9.18E-04 | NA | NA |
| ENSMUSG00000051839 | -2.41 | -5.32 | 1.54E-14 | Gypa | glycophorin A |
| ENSMUSG00000048824 | -2.41 | -5.32 | 3.45E-05 | NA | NA |
| ENSMUSG00000003410 | -2.41 | -5.32 | 3.71E-03 | Elavl3 | ELAV (embryonic lethal, abnormal vision, Drosophila)-like 3 (Hu antigen C) |
| ENSMUSG00000000157 | -2.41 | -5.31 | 1.88E-24 | Itgb2l | integrin beta 2-like |
| ENSMUSG00000053185 | -2.41 | -5.31 | 8.16E-04 | NA | NA |
| ENSMUSG00000024158 | -2.41 | -5.31 | 9.38E-40 | Hagh | hydroxyacyl glutathione hydrolase |
| ENSMUSG00000042761 | -2.41 | -5.31 | 1.51E-03 | Mrap2 | melanocortin 2 receptor accessory protein 2 |
| ENSMUSG00000017211 | -2.41 | -5.30 | 8.18E-03 | Gsdma2 | gasdermin A2 |
| ENSMUSG00000087636 | -2.41 | -5.30 | 9.28E-03 | NA | NA |
| ENSMUSG00000085355 | -2.41 | -5.30 | 5.59E-07 | NA | NA |
| ENSMUSG00000074923 | -2.41 | -5.30 | 7.24E-13 | Pak6 | p21 protein (Cdc42/Rac)-activated kinase 6 |
| ENSMUSG00000089643 | -2.40 | -5.30 | 9.02E-03 | NA | NA |
| ENSMUSG00000026579 | -2.40 | -5.30 | 1.08E-14 | F5 | coagulation factor V |
| ENSMUSG00000075025 | -2.40 | -5.30 | 2.36E-03 | Gm10804 | predicted gene 10804 |
| ENSMUSG00000074603 | -2.40 | -5.29 | 3.26E-03 | NA | NA |
| ENSMUSG00000050473 | -2.40 | -5.29 | 6.00E-19 | Slc35d3 | solute carrier family 35, member D3 |
| ENSMUSG00000061701 | -2.40 | -5.28 | 7.95E-03 | Fbxw20 | F-box and WD-40 domain protein 20 |
| ENSMUSG00000050108 | -2.40 | -5.28 | 1.74E-05 | Bpifc | BPI fold containing family C |
| ENSMUSG00000061242 | -2.40 | -5.28 | 8.42E-03 | NA | NA |
| ENSMUSG00000089932 | -2.40 | -5.26 | 8.58E-03 | NA | NA |
| ENSMUSG00000022818 | -2.40 | -5.26 | 8.95E-09 | Cyp2ab1 | cytochrome P450, family 2, subfamily ab, polypeptide 1 |
| ENSMUSG00000025573 | -2.40 | -5.26 | 2.06E-15 | 6030468B19Rik | RIKEN cDNA 6030468B19 gene |
| ENSMUSG00000060727 | -2.39 | -5.26 | 8.97E-18 | NA | NA |
| ENSMUSG00000082978 | -2.39 | -5.26 | 1.00E-06 | NA | NA |
| ENSMUSG00000076961 | -2.39 | -5.25 | 1.72E-03 | NA | NA |
| ENSMUSG00000045087 | -2.39 | -5.25 | 4.04E-06 | S1pr5 | sphingosine-1-phosphate receptor 5 |
| ENSMUSG00000036698 | -2.39 | -5.25 | 9.27E-20 | Ago2 | argonaute RISC catalytic subunit 2 |
| ENSMUSG00000023345 | -2.39 | -5.24 | 7.62E-20 | Poc1a | POC1 centriolar protein homolog A (Chlamydomonas) |
| ENSMUSG00000082279 | -2.39 | -5.24 | 2.88E-36 | NA | NA |
| ENSMUSG00000020295 | -2.39 | -5.23 | 2.44E-09 | Hbq1a | hemoglobin, theta 1A |
| ENSMUSG00000052040 | -2.39 | -5.23 | 3.93E-05 | Klf13 | Kruppel-like factor 13 |
| ENSMUSG00000049830 | -2.39 | -5.23 | 8.78E-03 | NA | NA |
| ENSMUSG00000065401 | -2.38 | -5.22 | 6.16E-05 | Mir144 | microRNA 144 |
| ENSMUSG00000084162 | -2.38 | -5.21 | 4.28E-03 | NA | NA |
| ENSMUSG00000081544 | -2.38 | -5.21 | 5.79E-12 | NA | NA |
| ENSMUSG00000074570 | -2.38 | -5.20 | 2.13E-36 | Cass4 | Cas scaffolding protein family member 4 |
| ENSMUSG00000088071 | -2.38 | -5.19 | 9.70E-03 | NA | NA |
| ENSMUSG00000051081 | -2.38 | -5.19 | 8.13E-03 | Gm4847 | predicted gene 4847 |
| ENSMUSG00000086615 | -2.38 | -5.19 | 8.66E-04 | NA | NA |
| ENSMUSG00000051497 | -2.37 | -5.19 | 2.21E-04 | Kcnj16 | potassium inwardly-rectifying channel, subfamily J, member 16 |
| ENSMUSG00000083046 | -2.37 | -5.18 | 6.54E-07 | NA | NA |
| ENSMUSG00000055159 | -2.37 | -5.18 | 9.48E-03 | 4930583K01Rik | RIKEN cDNA 4930583K01 gene |
| ENSMUSG00000020802 | -2.37 | -5.17 | 1.78E-26 | Ube2o | ubiquitin-conjugating enzyme E2O |
| ENSMUSG00000068101 | -2.37 | -5.17 | 3.64E-18 | Cenpm | centromere protein M |
| ENSMUSG00000081164 | -2.37 | -5.17 | 2.64E-13 | NA | NA |
| ENSMUSG00000045826 | -2.37 | -5.17 | 2.11E-20 | NA | NA |
| ENSMUSG00000082423 | -2.37 | -5.16 | 2.54E-03 | NA | NA |
| ENSMUSG00000085708 | -2.37 | -5.16 | 5.66E-03 | Gm16063 | predicted gene 16063 |
| ENSMUSG00000023903 | -2.37 | -5.16 | 1.52E-26 | Mmp25 | matrix metallopeptidase 25 |
| ENSMUSG00000063063 | -2.37 | -5.16 | 7.48E-03 | Ctnna2 | catenin (cadherin associated protein), alpha 2 |
| ENSMUSG00000025507 | -2.36 | -5.15 | 2.29E-29 | Lrdd | leucine-rich and death domain containing |
| ENSMUSG00000081380 | -2.36 | -5.15 | 4.14E-07 | NA | NA |
| ENSMUSG00000082971 | -2.36 | -5.14 | 3.72E-03 | NA | NA |
| ENSMUSG00000039452 | -2.36 | -5.14 | 4.20E-20 | Snx22 | sorting nexin 22 |
| ENSMUSG00000031391 | -2.36 | -5.14 | 1.93E-22 | L1cam | L1 cell adhesion molecule |
| ENSMUSG00000024670 | -2.36 | -5.14 | 1.25E-06 | Cd6 | CD6 antigen |
| ENSMUSG00000066639 | -2.36 | -5.14 | 5.20E-08 | NA | NA |
| ENSMUSG00000073093 | -2.36 | -5.13 | 4.14E-03 | NA | NA |
| ENSMUSG00000063796 | -2.36 | -5.13 | 4.76E-05 | Slc22a8 | solute carrier family 22 (organic anion transporter), member 8 |
| ENSMUSG00000087624 | -2.36 | -5.12 | 9.02E-13 | NA | NA |
| ENSMUSG00000070428 | -2.36 | -5.12 | 9.41E-03 | NA | NA |
| ENSMUSG00000069919 | -2.35 | -5.11 | 1.30E-10 | NA | NA |
| ENSMUSG00000086411 | -2.35 | -5.11 | 5.38E-03 | NA | NA |
| ENSMUSG00000022219 | -2.35 | -5.11 | 3.72E-11 | Cideb | cell death-inducing DNA fragmentation factor, alpha subunit-like effector B |
| ENSMUSG00000074569 | -2.35 | -5.10 | 2.19E-03 | Gcnt7 | glucosaminyl (N-acetyl) transferase family member 7 |
| ENSMUSG00000006398 | -2.35 | -5.10 | 6.09E-36 | Cdc20 | cell division cycle 20 |
| ENSMUSG00000087538 | -2.35 | -5.10 | 2.95E-03 | NA | NA |
| ENSMUSG00000029178 | -2.35 | -5.09 | 1.10E-12 | Klf3 | Kruppel-like factor 3 (basic) |
| ENSMUSG00000043800 | -2.35 | -5.09 | 1.19E-12 | NA | NA |
| ENSMUSG00000082341 | -2.35 | -5.09 | 4.14E-03 | NA | NA |
| ENSMUSG00000071226 | -2.35 | -5.09 | 2.92E-15 | Cecr2 | cat eye syndrome chromosome region, candidate 2 |
| ENSMUSG00000074662 | -2.35 | -5.08 | 1.79E-03 | NA | NA |
| ENSMUSG00000052403 | -2.35 | -5.08 | 8.00E-04 | NA | NA |
| ENSMUSG00000021069 | -2.34 | -5.08 | 4.03E-26 | Pygl | liver glycogen phosphorylase |
| ENSMUSG00000051582 | -2.34 | -5.08 | 9.98E-03 | Otud6a | OTU domain containing 6A |
| ENSMUSG00000081076 | -2.34 | -5.08 | 2.31E-26 | NA | NA |
| ENSMUSG00000028717 | -2.34 | -5.07 | 1.20E-19 | Tal1 | T cell acute lymphocytic leukemia 1 |
| ENSMUSG00000028843 | -2.34 | -5.06 | 2.35E-40 | Sh3bgrl3 | SH3 domain binding glutamic acid-rich protein-like 3 |
| ENSMUSG00000044794 | -2.34 | -5.06 | 1.87E-22 | NA | NA |
| ENSMUSG00000001228 | -2.34 | -5.06 | 2.53E-31 | Uhrf1 | ubiquitin-like, containing PHD and RING finger domains, 1 |
| ENSMUSG00000025330 | -2.34 | -5.06 | 8.61E-19 | Padi4 | peptidyl arginine deiminase, type IV |
| ENSMUSG00000036223 | -2.34 | -5.06 | 8.82E-15 | Ska1 | spindle and kinetochore associated complex subunit 1 |
| ENSMUSG00000035699 | -2.33 | -5.04 | 5.46E-03 | Slc51a | solute carrier family 51, alpha subunit |
| ENSMUSG00000020401 | -2.33 | -5.04 | 2.74E-15 | Fam71b | family with sequence similarity 71, member B |
| ENSMUSG00000068129 | -2.33 | -5.03 | 7.25E-14 | Cst7 | cystatin F (leukocystatin) |
| ENSMUSG00000027364 | -2.33 | -5.03 | 2.30E-04 | Usp50 | ubiquitin specific peptidase 50 |
| ENSMUSG00000028713 | -2.33 | -5.03 | 2.18E-33 | Cyp4b1 | cytochrome P450, family 4, subfamily b, polypeptide 1 |
| ENSMUSG00000030474 | -2.33 | -5.03 | 9.94E-20 | Siglece | sialic acid binding Ig-like lectin E |
| ENSMUSG00000018678 | -2.33 | -5.02 | 1.55E-42 | Sp2 | Sp2 transcription factor |
| ENSMUSG00000058550 | -2.33 | -5.02 | 9.88E-03 | Dppa4 | developmental pluripotency associated 4 |
| ENSMUSG00000028825 | -2.32 | -5.00 | 3.47E-14 | Rhd | Rh blood group, D antigen |
| ENSMUSG00000058626 | -2.32 | -5.00 | 1.23E-03 | Capn11 | calpain 11 |
| ENSMUSG00000082865 | -2.32 | -5.00 | 6.66E-03 | NA | NA |
| ENSMUSG00000045216 | -2.32 | -5.00 | 9.42E-55 | Hs6st1 | heparan sulfate 6-O-sulfotransferase 1 |
| ENSMUSG00000082345 | -2.32 | -4.99 | 4.19E-03 | NA | NA |
| ENSMUSG00000054169 | -2.31 | -4.97 | 5.38E-11 | Ceacam10 | carcinoembryonic antigen-related cell adhesion molecule 10 |
| ENSMUSG00000061353 | -2.31 | -4.97 | 1.01E-26 | Cxcl12 | chemokine (C-X-C motif) ligand 12 |
| ENSMUSG00000032259 | -2.31 | -4.96 | 2.07E-09 | Drd2 | dopamine receptor D2 |
| ENSMUSG00000034825 | -2.31 | -4.96 | 4.76E-13 | Nrip3 | nuclear receptor interacting protein 3 |
| ENSMUSG00000000486 | -2.31 | -4.96 | 1.37E-29 | Sept1 | septin 1 |
| ENSMUSG00000021221 | -2.31 | -4.96 | 9.36E-13 | Dpf3 | D4, zinc and double PHD fingers, family 3 |
| ENSMUSG00000001827 | -2.31 | -4.96 | 7.34E-04 | Folr1 | folate receptor 1 (adult) |
| ENSMUSG00000028386 | -2.31 | -4.95 | 9.27E-03 | Slc46a2 | solute carrier family 46, member 2 |
| ENSMUSG00000086236 | -2.31 | -4.95 | 2.53E-05 | 5830418P13Rik | RIKEN cDNA 5830418P13 gene |
| ENSMUSG00000079508 | -2.31 | -4.95 | 3.30E-06 | NA | NA |
| ENSMUSG00000037415 | -2.31 | -4.95 | 2.53E-19 | Ranbp10 | RAN binding protein 10 |
| ENSMUSG00000022375 | -2.31 | -4.95 | 4.11E-03 | Lrrc6 | leucine rich repeat containing 6 (testis) |
| ENSMUSG00000086660 | -2.31 | -4.95 | 9.86E-03 | NA | NA |
| ENSMUSG00000015053 | -2.31 | -4.95 | 1.67E-43 | Gata2 | GATA binding protein 2 |
| ENSMUSG00000024171 | -2.30 | -4.94 | 1.26E-04 | Prss28 | protease, serine 28 |
| ENSMUSG00000025701 | -2.30 | -4.94 | 3.27E-24 | Alox5 | arachidonate 5-lipoxygenase |
| ENSMUSG00000040809 | -2.30 | -4.93 | 9.30E-10 | Chil3 | chitinase-like 3 |
| ENSMUSG00000040432 | -2.30 | -4.93 | 3.24E-17 | Ltb4r2 | leukotriene B4 receptor 2 |
| ENSMUSG00000056091 | -2.30 | -4.93 | 7.40E-25 | St3gal5 | ST3 beta-galactoside alpha-2,3-sialyltransferase 5 |
| ENSMUSG00000020096 | -2.30 | -4.91 | 2.70E-03 | Tbata | thymus, brain and testes associated |
| ENSMUSG00000074272 | -2.30 | -4.91 | 7.90E-23 | Ceacam1 | carcinoembryonic antigen-related cell adhesion molecule 1 |
| ENSMUSG00000082596 | -2.29 | -4.90 | 2.17E-03 | NA | NA |
| ENSMUSG00000024730 | -2.29 | -4.90 | 8.82E-08 | Ms4a8a | membrane-spanning 4-domains, subfamily A, member 8A |
| ENSMUSG00000043670 | -2.29 | -4.89 | 3.77E-03 | Diras1 | DIRAS family, GTP-binding RAS-like 1 |
| ENSMUSG00000029866 | -2.29 | -4.89 | 2.32E-15 | Kel | Kell blood group |
| ENSMUSG00000085774 | -2.29 | -4.89 | 2.99E-03 | NA | NA |
| ENSMUSG00000041359 | -2.29 | -4.87 | 8.09E-03 | Tcl1 | T cell lymphoma breakpoint 1 |
| ENSMUSG00000070280 | -2.29 | -4.87 | 3.10E-03 | Slc22a14 | solute carrier family 22 (organic cation transporter), member 14 |
| ENSMUSG00000021697 | -2.28 | -4.87 | 1.13E-21 | Depdc1b | DEP domain containing 1B |
| ENSMUSG00000051811 | -2.28 | -4.87 | 1.46E-16 | Cox6b2 | cytochrome c oxidase subunit VIb polypeptide 2 |
| ENSMUSG00000045509 | -2.28 | -4.87 | 2.19E-03 | Gpr150 | G protein-coupled receptor 150 |
| ENSMUSG00000032202 | -2.28 | -4.87 | 4.96E-22 | Rab27a | RAB27A, member RAS oncogene family |
| ENSMUSG00000030711 | -2.28 | -4.87 | 2.84E-21 | Sult1a1 | sulfotransferase family 1A, phenol-preferring, member 1 |
| ENSMUSG00000061787 | -2.28 | -4.87 | 7.13E-17 | Rps17 | ribosomal protein S17 |
| ENSMUSG00000024180 | -2.28 | -4.86 | 1.78E-24 | Tmem8 | transmembrane protein 8 (five membrane-spanning domains) |
| ENSMUSG00000039236 | -2.28 | -4.86 | 2.38E-29 | Isg20 | interferon-stimulated protein |
| ENSMUSG00000088371 | -2.28 | -4.85 | 2.60E-05 | NA | NA |
| ENSMUSG00000052477 | -2.28 | -4.85 | 1.37E-05 | C130026I21Rik | RIKEN cDNA C130026I21 gene |
| ENSMUSG00000071658 | -2.28 | -4.85 | 3.68E-05 | Gng3 | guanine nucleotide binding protein (G protein), gamma 3 |
| ENSMUSG00000001225 | -2.28 | -4.85 | 3.00E-03 | Slc26a3 | solute carrier family 26, member 3 |
| ENSMUSG00000037012 | -2.28 | -4.84 | 5.55E-36 | Hk1 | hexokinase 1 |
| ENSMUSG00000056155 | -2.28 | -4.84 | 2.24E-04 | Nanos3 | nanos homolog 3 (Drosophila) |
| ENSMUSG00000032496 | -2.28 | -4.84 | 1.29E-14 | Ltf | lactotransferrin |
| ENSMUSG00000061482 | -2.27 | -4.84 | 6.28E-04 | NA | NA |
| ENSMUSG00000032561 | -2.27 | -4.84 | 6.00E-13 | Acpp | acid phosphatase, prostate |
| ENSMUSG00000042744 | -2.27 | -4.84 | 6.99E-11 | Gm15800 | predicted gene 15800 |
| ENSMUSG00000029730 | -2.27 | -4.83 | 1.50E-33 | Mcm7 | minichromosome maintenance deficient 7 (S. cerevisiae) |
| ENSMUSG00000023505 | -2.27 | -4.83 | 1.65E-22 | Cdca3 | cell division cycle associated 3 |
| ENSMUSG00000031980 | -2.27 | -4.83 | 9.21E-14 | Agt | angiotensinogen (serpin peptidase inhibitor, clade A, member 8) |
| ENSMUSG00000032589 | -2.27 | -4.82 | 2.22E-19 | Bsn | bassoon |
| ENSMUSG00000084862 | -2.27 | -4.82 | 2.40E-05 | NA | NA |
| ENSMUSG00000075184 | -2.27 | -4.81 | 3.76E-14 | F830002L21Rik | RIKEN cDNA F830002L21 gene |
| ENSMUSG00000037336 | -2.27 | -4.81 | 1.36E-15 | Mfsd2b | major facilitator superfamily domain containing 2B |
| ENSMUSG00000031444 | -2.27 | -4.81 | 4.52E-18 | F10 | coagulation factor X |
| ENSMUSG00000072618 | -2.27 | -4.81 | 3.16E-10 | NA | NA |
| ENSMUSG00000083995 | -2.27 | -4.81 | 9.82E-03 | NA | NA |
| ENSMUSG00000020020 | -2.26 | -4.80 | 5.32E-05 | Usp44 | ubiquitin specific peptidase 44 |
| ENSMUSG00000005202 | -2.26 | -4.80 | 6.29E-03 | Shbg | sex hormone binding globulin |
| ENSMUSG00000082931 | -2.26 | -4.80 | 8.50E-03 | NA | NA |
| ENSMUSG00000063177 | -2.26 | -4.80 | 7.76E-08 | Klk1b27 | kallikrein 1-related peptidase b27 |
| ENSMUSG00000031488 | -2.26 | -4.79 | 2.71E-20 | Rab11fip1 | RAB11 family interacting protein 1 (class I) |
| ENSMUSG00000069910 | -2.26 | -4.79 | 1.14E-22 | Spdl1 | spindle apparatus coiled-coil protein 1 |
| ENSMUSG00000021123 | -2.26 | -4.79 | 2.60E-11 | Rdh12 | retinol dehydrogenase 12 |
| ENSMUSG00000047139 | -2.26 | -4.79 | 8.82E-19 | Cd24a | CD24a antigen |
| ENSMUSG00000000182 | -2.26 | -4.79 | 1.23E-03 | Fgf23 | fibroblast growth factor 23 |
| ENSMUSG00000056399 | -2.26 | -4.78 | 6.13E-13 | Prss34 | protease, serine 34 |
| ENSMUSG00000078350 | -2.26 | -4.78 | 2.27E-38 | Smim1 | small integral membrane protein 1 |
| ENSMUSG00000028644 | -2.25 | -4.77 | 4.87E-12 | Ermap | erythroblast membrane-associated protein |
| ENSMUSG00000067316 | -2.25 | -4.77 | 5.35E-03 | NA | NA |
| ENSMUSG00000086587 | -2.25 | -4.77 | 1.36E-08 | NA | NA |
| ENSMUSG00000054733 | -2.25 | -4.77 | 1.75E-34 | Msra | methionine sulfoxide reductase A |
| ENSMUSG00000083289 | -2.25 | -4.77 | 3.93E-03 | NA | NA |
| ENSMUSG00000047842 | -2.25 | -4.76 | 3.41E-18 | Diras2 | DIRAS family, GTP-binding RAS-like 2 |
| ENSMUSG00000007659 | -2.25 | -4.75 | 1.36E-39 | Bcl2l1 | BCL2-like 1 |
| ENSMUSG00000060791 | -2.25 | -4.75 | 2.09E-21 | NA | NA |
| ENSMUSG00000024590 | -2.25 | -4.75 | 1.22E-40 | Lmnb1 | lamin B1 |
| ENSMUSG00000030220 | -2.25 | -4.75 | 2.74E-19 | Arhgdib | Rho, GDP dissociation inhibitor (GDI) beta |
| ENSMUSG00000084243 | -2.25 | -4.75 | 6.58E-03 | NA | NA |
| ENSMUSG00000022584 | -2.24 | -4.74 | 4.73E-13 | Ly6c2 | lymphocyte antigen 6 complex, locus C2 |
| ENSMUSG00000024867 | -2.24 | -4.74 | 7.28E-26 | Pip5k1b | phosphatidylinositol-4-phosphate 5-kinase, type 1 beta |
| ENSMUSG00000024533 | -2.24 | -4.74 | 8.08E-22 | Spire1 | spire homolog 1 (Drosophila) |
| ENSMUSG00000061742 | -2.24 | -4.73 | 6.45E-03 | Slc22a12 | solute carrier family 22 (organic anion/cation transporter), member 12 |
| ENSMUSG00000054161 | -2.24 | -4.73 | 1.53E-03 | Fam83e | family with sequence similarity 83, member E |
| ENSMUSG00000042678 | -2.24 | -4.73 | 1.71E-07 | Myo15 | myosin XV |
| ENSMUSG00000068397 | -2.24 | -4.73 | 7.32E-04 | NA | NA |
| ENSMUSG00000082623 | -2.24 | -4.73 | 3.13E-03 | NA | NA |
| ENSMUSG00000031860 | -2.24 | -4.73 | 8.68E-08 | Pbx4 | pre B cell leukemia homeobox 4 |
| ENSMUSG00000081453 | -2.24 | -4.73 | 1.23E-08 | NA | NA |
| ENSMUSG00000027469 | -2.24 | -4.72 | 1.84E-24 | Tpx2 | TPX2, microtubule-associated protein homolog (Xenopus laevis) |
| ENSMUSG00000020340 | -2.24 | -4.72 | 1.63E-23 | Cyfip2 | cytoplasmic FMR1 interacting protein 2 |
| ENSMUSG00000039809 | -2.24 | -4.72 | 9.30E-07 | Gabbr2 | gamma-aminobutyric acid (GABA) B receptor, 2 |
| ENSMUSG00000045019 | -2.24 | -4.72 | 1.04E-03 | Acer1 | alkaline ceramidase 1 |
| ENSMUSG00000066438 | -2.24 | -4.71 | 1.73E-04 | Plekhd1 | pleckstrin homology domain containing, family D (with coiled-coil domains) member 1 |
| ENSMUSG00000076726 | -2.24 | -4.71 | 5.97E-05 | NA | NA |
| ENSMUSG00000030336 | -2.23 | -4.71 | 4.06E-17 | Cd27 | CD27 antigen |
| ENSMUSG00000080778 | -2.23 | -4.70 | 6.68E-06 | NA | NA |
| ENSMUSG00000082645 | -2.23 | -4.70 | 6.81E-03 | NA | NA |
| ENSMUSG00000031738 | -2.23 | -4.70 | 9.09E-07 | Irx6 | Iroquois related homeobox 6 (Drosophila) |
| ENSMUSG00000034336 | -2.23 | -4.69 | 4.75E-03 | Ina | internexin neuronal intermediate filament protein, alpha |
| ENSMUSG00000028542 | -2.23 | -4.69 | 5.53E-20 | Slc6a9 | solute carrier family 6 (neurotransmitter transporter, glycine), member 9 |
| ENSMUSG00000081921 | -2.23 | -4.69 | 1.17E-08 | NA | NA |
| ENSMUSG00000085787 | -2.23 | -4.69 | 3.86E-17 | NA | NA |
| ENSMUSG00000002384 | -2.23 | -4.68 | 3.15E-05 | Bmp8b | bone morphogenetic protein 8b |
| ENSMUSG00000078652 | -2.23 | -4.68 | 5.83E-25 | Psme3 | proteaseome (prosome, macropain) activator subunit 3 (PA28 gamma, Ki) |
| ENSMUSG00000085030 | -2.22 | -4.67 | 1.22E-08 | NA | NA |
| ENSMUSG00000085085 | -2.22 | -4.67 | 5.61E-04 | NA | NA |
| ENSMUSG00000032021 | -2.22 | -4.67 | 2.48E-12 | Crtam | cytotoxic and regulatory T cell molecule |
| ENSMUSG00000024936 | -2.22 | -4.67 | 1.10E-04 | Kcnk7 | potassium channel, subfamily K, member 7 |
| ENSMUSG00000075240 | -2.22 | -4.67 | 6.02E-03 | NA | NA |
| ENSMUSG00000043794 | -2.22 | -4.66 | 2.92E-04 | NA | NA |
| ENSMUSG00000027867 | -2.22 | -4.65 | 1.05E-03 | Spag17 | sperm associated antigen 17 |
| ENSMUSG00000085715 | -2.22 | -4.65 | 4.10E-03 | Tsix | X (inactive)-specific transcript, opposite strand |
| ENSMUSG00000018654 | -2.21 | -4.64 | 7.26E-28 | Ikzf1 | IKAROS family zinc finger 1 |
| ENSMUSG00000034265 | -2.21 | -4.64 | 1.57E-47 | Zdhhc14 | zinc finger, DHHC domain containing 14 |
| ENSMUSG00000048185 | -2.21 | -4.64 | 3.66E-03 | NA | NA |
| ENSMUSG00000071068 | -2.21 | -4.63 | 3.26E-17 | Treml2 | triggering receptor expressed on myeloid cells-like 2 |
| ENSMUSG00000082815 | -2.21 | -4.63 | 8.87E-04 | NA | NA |
| ENSMUSG00000031004 | -2.21 | -4.63 | 9.00E-11 | Mki67 | antigen identified by monoclonal antibody Ki 67 |
| ENSMUSG00000020679 | -2.21 | -4.62 | 7.67E-03 | Hnf1b | HNF1 homeobox B |
| ENSMUSG00000055817 | -2.21 | -4.62 | 3.46E-32 | Mta3 | metastasis associated 3 |
| ENSMUSG00000085617 | -2.21 | -4.62 | 4.37E-04 | NA | NA |
| ENSMUSG00000046591 | -2.21 | -4.61 | 7.32E-16 | Ticrr | TOPBP1-interacting checkpoint and replication regulator |
| ENSMUSG00000002221 | -2.20 | -4.61 | 2.08E-19 | Paxip1 | PAX interacting (with transcription-activation domain) protein 1 |
| ENSMUSG00000054191 | -2.20 | -4.61 | 1.55E-11 | Klf1 | Kruppel-like factor 1 (erythroid) |
| ENSMUSG00000086673 | -2.20 | -4.61 | 8.12E-03 | NA | NA |
| ENSMUSG00000028528 | -2.20 | -4.60 | 1.60E-12 | Dnajc6 | DnaJ (Hsp40) homolog, subfamily C, member 6 |
| ENSMUSG00000001741 | -2.20 | -4.60 | 3.99E-23 | Il16 | interleukin 16 |
| ENSMUSG00000042417 | -2.20 | -4.60 | 1.86E-10 | Ccno | cyclin O |
| ENSMUSG00000074903 | -2.20 | -4.60 | 4.30E-22 | NA | NA |
| ENSMUSG00000068851 | -2.20 | -4.60 | 5.35E-05 | NA | NA |
| ENSMUSG00000038725 | -2.20 | -4.60 | 7.90E-12 | Pkhd1l1 | polycystic kidney and hepatic disease 1-like 1 |
| ENSMUSG00000064225 | -2.20 | -4.60 | 2.62E-06 | Paqr9 | progestin and adipoQ receptor family member IX |
| ENSMUSG00000056476 | -2.20 | -4.59 | 1.24E-06 | Med12l | mediator complex subunit 12-like |
| ENSMUSG00000001494 | -2.20 | -4.59 | 1.55E-16 | Sost | sclerostin |
| ENSMUSG00000051502 | -2.20 | -4.59 | 1.51E-24 | Ufsp1 | UFM1-specific peptidase 1 |
| ENSMUSG00000082465 | -2.20 | -4.59 | 1.28E-03 | NA | NA |
| ENSMUSG00000052397 | -2.20 | -4.59 | 3.79E-31 | Ezr | ezrin |
| ENSMUSG00000081715 | -2.20 | -4.58 | 1.71E-07 | NA | NA |
| ENSMUSG00000000617 | -2.19 | -4.58 | 2.50E-05 | Grm6 | glutamate receptor, metabotropic 6 |
| ENSMUSG00000020037 | -2.19 | -4.58 | 2.20E-03 | Rfx4 | regulatory factor X, 4 (influences HLA class II expression) |
| ENSMUSG00000082461 | -2.19 | -4.57 | 9.42E-14 | NA | NA |
| ENSMUSG00000073400 | -2.19 | -4.57 | 8.68E-14 | Trim10 | tripartite motif-containing 10 |
| ENSMUSG00000029607 | -2.19 | -4.57 | 1.21E-07 | Ankrd61 | ankyrin repeat domain 61 |
| ENSMUSG00000040761 | -2.19 | -4.56 | 2.12E-13 | Spen | SPEN homolog, transcriptional regulator (Drosophila) |
| ENSMUSG00000015950 | -2.19 | -4.56 | 1.26E-28 | Ncf1 | neutrophil cytosolic factor 1 |
| ENSMUSG00000028931 | -2.19 | -4.56 | 1.29E-53 | Kcnab2 | potassium voltage-gated channel, shaker-related subfamily, beta member 2 |
| ENSMUSG00000031785 | -2.19 | -4.56 | 1.00E-43 | Gpr56 | G protein-coupled receptor 56 |
| ENSMUSG00000033685 | -2.19 | -4.55 | 7.70E-26 | Ucp2 | uncoupling protein 2 (mitochondrial, proton carrier) |
| ENSMUSG00000025389 | -2.19 | -4.55 | 3.54E-07 | Mip | major intrinsic protein of eye lens fiber |
| ENSMUSG00000086425 | -2.18 | -4.54 | 1.16E-11 | F730016J06Rik | RIKEN cDNA F730016J06 gene |
| ENSMUSG00000030160 | -2.18 | -4.54 | 1.61E-03 | Tmem52b | transmembrane protein 52B |
| ENSMUSG00000001403 | -2.18 | -4.54 | 2.06E-19 | Ube2c | ubiquitin-conjugating enzyme E2C |
| ENSMUSG00000083890 | -2.18 | -4.54 | 1.82E-14 | NA | NA |
| ENSMUSG00000021182 | -2.18 | -4.54 | 3.80E-11 | Ccdc88c | coiled-coil domain containing 88C |
| ENSMUSG00000078487 | -2.18 | -4.54 | 2.95E-03 | Ankrd65 | ankyrin repeat domain 65 |
| ENSMUSG00000025557 | -2.18 | -4.54 | 9.27E-03 | Slc15a1 | solute carrier family 15 (oligopeptide transporter), member 1 |
| ENSMUSG00000050357 | -2.18 | -4.53 | 1.22E-15 | Rltpr | RGD motif, leucine rich repeats, tropomodulin domain and proline-rich containing |
| ENSMUSG00000030714 | -2.18 | -4.52 | 4.55E-27 | Ccdc101 | coiled-coil domain containing 101 |
| ENSMUSG00000086335 | -2.18 | -4.52 | 7.37E-05 | NA | NA |
| ENSMUSG00000019359 | -2.18 | -4.52 | 8.46E-17 | Gdpd2 | glycerophosphodiester phosphodiesterase domain containing 2 |
| ENSMUSG00000022377 | -2.17 | -4.51 | 1.68E-36 | Asap1 | ArfGAP with SH3 domain, ankyrin repeat and PH domain1 |
| ENSMUSG00000039158 | -2.17 | -4.51 | 1.24E-28 | Akna | AT-hook transcription factor |
| ENSMUSG00000069805 | -2.17 | -4.51 | 4.65E-20 | Fbp1 | fructose bisphosphatase 1 |
| ENSMUSG00000073288 | -2.17 | -4.51 | 2.46E-03 | NA | NA |
| ENSMUSG00000034041 | -2.17 | -4.51 | 6.54E-18 | Lyl1 | lymphoblastomic leukemia 1 |
| ENSMUSG00000083764 | -2.17 | -4.51 | 1.22E-16 | NA | NA |
| ENSMUSG00000086146 | -2.17 | -4.50 | 4.74E-05 | NA | NA |
| ENSMUSG00000029513 | -2.17 | -4.50 | 1.61E-54 | Prkab1 | protein kinase, AMP-activated, beta 1 non-catalytic subunit |
| ENSMUSG00000051527 | -2.17 | -4.50 | 9.25E-03 | Usp29 | ubiquitin specific peptidase 29 |
| ENSMUSG00000067371 | -2.17 | -4.50 | 4.32E-04 | NA | NA |
| ENSMUSG00000020235 | -2.17 | -4.49 | 7.16E-40 | Fzr1 | fizzy/cell division cycle 20 related 1 (Drosophila) |
| ENSMUSG00000038298 | -2.17 | -4.49 | 5.84E-08 | Pdzk1 | PDZ domain containing 1 |
| ENSMUSG00000015854 | -2.17 | -4.49 | 4.29E-11 | Cd5l | CD5 antigen-like |
| ENSMUSG00000056394 | -2.17 | -4.49 | 2.82E-45 | Lig1 | ligase I, DNA, ATP-dependent |
| ENSMUSG00000004099 | -2.17 | -4.49 | 3.11E-21 | Dnmt1 | DNA methyltransferase (cytosine-5) 1 |
| ENSMUSG00000081295 | -2.17 | -4.48 | 1.14E-23 | NA | NA |
| ENSMUSG00000087020 | -2.16 | -4.48 | 6.80E-06 | NA | NA |
| ENSMUSG00000032034 | -2.16 | -4.48 | 1.12E-10 | Kcnj5 | potassium inwardly-rectifying channel, subfamily J, member 5 |
| ENSMUSG00000032946 | -2.16 | -4.48 | 1.09E-15 | Rasgrp2 | RAS, guanyl releasing protein 2 |
| ENSMUSG00000081126 | -2.16 | -4.48 | 1.58E-19 | NA | NA |
| ENSMUSG00000047658 | -2.16 | -4.47 | 9.62E-03 | Gal3st3 | galactose-3-O-sulfotransferase 3 |
| ENSMUSG00000081325 | -2.16 | -4.47 | 2.41E-07 | NA | NA |
| ENSMUSG00000036770 | -2.16 | -4.47 | 7.00E-03 | 4933433C11Rik | RIKEN cDNA 4933433C11 gene |
| ENSMUSG00000027496 | -2.16 | -4.46 | 3.25E-21 | Aurka | aurora kinase A |
| ENSMUSG00000022875 | -2.16 | -4.46 | 5.95E-14 | Kng1 | kininogen 1 |
| ENSMUSG00000081952 | -2.16 | -4.46 | 1.73E-06 | NA | NA |
| ENSMUSG00000049115 | -2.15 | -4.45 | 4.81E-23 | Agtr1a | angiotensin II receptor, type 1a |
| ENSMUSG00000030303 | -2.15 | -4.44 | 3.41E-19 | Far2 | fatty acyl CoA reductase 2 |
| ENSMUSG00000042066 | -2.15 | -4.43 | 1.13E-13 | Tmcc2 | transmembrane and coiled-coil domains 2 |
| ENSMUSG00000089630 | -2.15 | -4.43 | 1.26E-03 | NA | NA |
| ENSMUSG00000049971 | -2.15 | -4.43 | 1.27E-12 | Glt1d1 | glycosyltransferase 1 domain containing 1 |
| ENSMUSG00000030825 | -2.15 | -4.43 | 2.41E-05 | Hsd17b14 | hydroxysteroid (17-beta) dehydrogenase 14 |
| ENSMUSG00000000731 | -2.15 | -4.43 | 8.24E-03 | Aire | autoimmune regulator (autoimmune polyendocrinopathy candidiasis ectodermal dystrophy) |
| ENSMUSG00000025163 | -2.15 | -4.43 | 8.54E-14 | Cd7 | CD7 antigen |
| ENSMUSG00000033688 | -2.15 | -4.42 | 2.25E-11 | 1300017J02Rik | RIKEN cDNA 1300017J02 gene |
| ENSMUSG00000073682 | -2.14 | -4.42 | 1.38E-03 | NA | NA |
| ENSMUSG00000085509 | -2.14 | -4.42 | 3.07E-04 | NA | NA |
| ENSMUSG00000025318 | -2.14 | -4.41 | 1.76E-07 | Jph3 | junctophilin 3 |
| ENSMUSG00000028333 | -2.14 | -4.41 | 7.38E-10 | Anp32b | acidic (leucine-rich) nuclear phosphoprotein 32 family, member B |
| ENSMUSG00000035576 | -2.14 | -4.41 | 1.17E-04 | L3mbtl1 | l(3)mbt-like (Drosophila) |
| ENSMUSG00000005672 | -2.14 | -4.41 | 2.92E-31 | Kit | kit oncogene |
| ENSMUSG00000082510 | -2.14 | -4.41 | 9.88E-03 | NA | NA |
| ENSMUSG00000089820 | -2.14 | -4.40 | 8.40E-04 | NA | NA |
| ENSMUSG00000005364 | -2.14 | -4.39 | 1.37E-06 | Il5ra | interleukin 5 receptor, alpha |
| ENSMUSG00000038618 | -2.13 | -4.39 | 1.58E-16 | Rassf7 | Ras association (RalGDS/AF-6) domain family (N-terminal) member 7 |
| ENSMUSG00000032586 | -2.13 | -4.39 | 9.14E-29 | Traip | TRAF-interacting protein |
| ENSMUSG00000027342 | -2.13 | -4.39 | 2.08E-22 | Pcna | proliferating cell nuclear antigen |
| ENSMUSG00000036752 | -2.13 | -4.39 | 1.01E-25 | Tubb4b | tubulin, beta 4B class IVB |
| ENSMUSG00000082203 | -2.13 | -4.38 | 7.53E-04 | NA | NA |
| ENSMUSG00000073529 | -2.13 | -4.38 | 2.75E-04 | NA | NA |
| ENSMUSG00000023926 | -2.13 | -4.37 | 3.39E-11 | Rhag | Rhesus blood group-associated A glycoprotein |
| ENSMUSG00000056724 | -2.13 | -4.37 | 3.29E-17 | Nbeal2 | neurobeachin-like 2 |
| ENSMUSG00000085692 | -2.13 | -4.37 | 2.09E-04 | NA | NA |
| ENSMUSG00000030854 | -2.13 | -4.37 | 3.21E-03 | Ptpn5 | protein tyrosine phosphatase, non-receptor type 5 |
| ENSMUSG00000003341 | -2.13 | -4.37 | 2.37E-04 | Atp8b3 | ATPase, class I, type 8B, member 3 |
| ENSMUSG00000006235 | -2.13 | -4.37 | 2.22E-11 | Epor | erythropoietin receptor |
| ENSMUSG00000028873 | -2.13 | -4.37 | 9.59E-23 | Cdca8 | cell division cycle associated 8 |
| ENSMUSG00000022683 | -2.13 | -4.36 | 1.24E-04 | Pla2g10 | phospholipase A2, group X |
| ENSMUSG00000079228 | -2.13 | -4.36 | 3.12E-06 | NA | NA |
| ENSMUSG00000040413 | -2.12 | -4.36 | 6.10E-03 | Timd2 | T cell immunoglobulin and mucin domain containing 2 |
| ENSMUSG00000025189 | -2.12 | -4.36 | 5.20E-07 | Cnnm1 | cyclin M1 |
| ENSMUSG00000029445 | -2.12 | -4.36 | 8.51E-03 | Hpd | 4-hydroxyphenylpyruvic acid dioxygenase |
| ENSMUSG00000086127 | -2.12 | -4.35 | 4.67E-04 | NA | NA |
| ENSMUSG00000030956 | -2.12 | -4.35 | 1.52E-33 | Fam53b | family with sequence similarity 53, member B |
| ENSMUSG00000027875 | -2.12 | -4.35 | 2.41E-14 | Hmgcs2 | 3-hydroxy-3-methylglutaryl-Coenzyme A synthase 2 |
| ENSMUSG00000050440 | -2.12 | -4.35 | 6.76E-03 | Hamp | hepcidin antimicrobial peptide |
| ENSMUSG00000020963 | -2.12 | -4.35 | 7.19E-15 | Tshr | thyroid stimulating hormone receptor |
| ENSMUSG00000087444 | -2.12 | -4.35 | 7.85E-03 | Gm5475 | predicted gene 5475 |
| ENSMUSG00000070985 | -2.12 | -4.34 | 7.56E-03 | Acnat1 | acyl-coenzyme A amino acid N-acyltransferase 1 |
| ENSMUSG00000084220 | -2.12 | -4.34 | 1.45E-10 | NA | NA |
| ENSMUSG00000026134 | -2.12 | -4.33 | 7.03E-36 | Prim2 | DNA primase, p58 subunit |
| ENSMUSG00000054342 | -2.11 | -4.33 | 4.95E-30 | Kcnn4 | potassium intermediate/small conductance calcium-activated channel, subfamily N, member 4 |
| ENSMUSG00000043036 | -2.11 | -4.33 | 5.18E-06 | Ccdc63 | coiled-coil domain containing 63 |
| ENSMUSG00000038578 | -2.11 | -4.33 | 2.42E-20 | Susd1 | sushi domain containing 1 |
| ENSMUSG00000022742 | -2.11 | -4.33 | 5.74E-24 | Cpox | coproporphyrinogen oxidase |
| ENSMUSG00000082101 | -2.11 | -4.33 | 7.72E-11 | Slfn14 | schlafen 14 |
| ENSMUSG00000061533 | -2.11 | -4.33 | 2.38E-19 | Cep128 | centrosomal protein 128 |
| ENSMUSG00000041544 | -2.11 | -4.32 | 9.16E-06 | Ptchd2 | patched domain containing 2 |
| ENSMUSG00000073423 | -2.11 | -4.32 | 4.83E-31 | Zfp414 | zinc finger protein 414 |
| ENSMUSG00000084953 | -2.11 | -4.32 | 9.90E-07 | NA | NA |
| ENSMUSG00000039232 | -2.11 | -4.32 | 2.53E-25 | Stx11 | syntaxin 11 |
| ENSMUSG00000030270 | -2.11 | -4.32 | 1.69E-03 | Cpne9 | copine family member IX |
| ENSMUSG00000028328 | -2.11 | -4.31 | 1.69E-21 | Tmod1 | tropomodulin 1 |
| ENSMUSG00000074491 | -2.11 | -4.31 | 2.81E-09 | Clec4g | C-type lectin domain family 4, member g |
| ENSMUSG00000089267 | -2.11 | -4.31 | 8.93E-04 | NA | NA |
| ENSMUSG00000022103 | -2.11 | -4.30 | 4.51E-19 | Gfra2 | glial cell line derived neurotrophic factor family receptor alpha 2 |
| ENSMUSG00000047953 | -2.10 | -4.30 | 7.41E-13 | Gp5 | glycoprotein 5 (platelet) |
| ENSMUSG00000047370 | -2.10 | -4.30 | 1.01E-08 | NA | NA |
| ENSMUSG00000055546 | -2.10 | -4.29 | 4.91E-18 | Timd4 | T cell immunoglobulin and mucin domain containing 4 |
| ENSMUSG00000020689 | -2.10 | -4.29 | 1.91E-28 | Itgb3 | integrin beta 3 |
| ENSMUSG00000037020 | -2.10 | -4.29 | 1.08E-28 | Wdr62 | WD repeat domain 62 |
| ENSMUSG00000086952 | -2.10 | -4.29 | 9.52E-04 | NA | NA |
| ENSMUSG00000027863 | -2.10 | -4.29 | 1.38E-11 | Cd2 | CD2 antigen |
| ENSMUSG00000087450 | -2.10 | -4.29 | 5.60E-03 | NA | NA |
| ENSMUSG00000048559 | -2.10 | -4.28 | 9.50E-03 | NA | NA |
| ENSMUSG00000087740 | -2.10 | -4.28 | 4.96E-04 | NA | NA |
| ENSMUSG00000017950 | -2.10 | -4.28 | 7.15E-04 | Hnf4a | hepatic nuclear factor 4, alpha |
| ENSMUSG00000015217 | -2.10 | -4.28 | 1.50E-14 | Hmgb3 | high mobility group box 3 |
| ENSMUSG00000032126 | -2.09 | -4.27 | 1.55E-16 | Hmbs | hydroxymethylbilane synthase |
| ENSMUSG00000056699 | -2.09 | -4.27 | 6.85E-03 | NA | NA |
| ENSMUSG00000018698 | -2.09 | -4.26 | 3.64E-05 | Lhx1 | LIM homeobox protein 1 |
| ENSMUSG00000051682 | -2.09 | -4.25 | 4.91E-15 | Treml4 | triggering receptor expressed on myeloid cells-like 4 |
| ENSMUSG00000028801 | -2.09 | -4.25 | 6.80E-03 | Stpg1 | sperm tail PG rich repeat containing 1 |
| ENSMUSG00000025580 | -2.08 | -4.24 | 1.70E-15 | NA | NA |
| ENSMUSG00000058546 | -2.08 | -4.24 | 2.18E-09 | NA | NA |
| ENSMUSG00000032514 | -2.08 | -4.24 | 1.34E-09 | Ttc21a | tetratricopeptide repeat domain 21A |
| ENSMUSG00000040412 | -2.08 | -4.23 | 5.87E-03 | 5330417C22Rik | RIKEN cDNA 5330417C22 gene |
| ENSMUSG00000036091 | -2.08 | -4.22 | 3.21E-08 | Hyal3 | hyaluronoglucosaminidase 3 |
| ENSMUSG00000056054 | -2.08 | -4.22 | 1.12E-07 | S100a8 | S100 calcium binding protein A8 (calgranulin A) |
| ENSMUSG00000020286 | -2.08 | -4.22 | 7.69E-03 | 1700093K21Rik | RIKEN cDNA 1700093K21 gene |
| ENSMUSG00000060429 | -2.08 | -4.21 | 3.53E-08 | Sntb1 | syntrophin, basic 1 |
| ENSMUSG00000030677 | -2.07 | -4.21 | 5.80E-29 | Kif22 | kinesin family member 22 |
| ENSMUSG00000000028 | -2.07 | -4.21 | 1.90E-17 | Cdc45 | cell division cycle 45 |
| ENSMUSG00000039814 | -2.07 | -4.21 | 1.48E-23 | Xkr5 | X Kell blood group precursor-related family, member 5 |
| ENSMUSG00000035632 | -2.07 | -4.21 | 5.10E-27 | Cnot3 | CCR4-NOT transcription complex, subunit 3 |
| ENSMUSG00000037221 | -2.07 | -4.21 | 8.39E-13 | Mospd3 | motile sperm domain containing 3 |
| ENSMUSG00000063239 | -2.07 | -4.20 | 3.54E-06 | Grm4 | glutamate receptor, metabotropic 4 |
| ENSMUSG00000048154 | -2.07 | -4.20 | 5.97E-13 | Kmt2d | lysine (K)-specific methyltransferase 2D |
| ENSMUSG00000027748 | -2.07 | -4.20 | 8.05E-04 | Trpc4 | transient receptor potential cation channel, subfamily C, member 4 |
| ENSMUSG00000024380 | -2.07 | -4.20 | 3.46E-37 | NA | NA |
| ENSMUSG00000017499 | -2.07 | -4.19 | 1.71E-17 | Cdc6 | cell division cycle 6 |
| ENSMUSG00000066364 | -2.07 | -4.19 | 3.28E-10 | Serpina3b | serine (or cysteine) peptidase inhibitor, clade A, member 3B |
| ENSMUSG00000013974 | -2.07 | -4.19 | 1.37E-11 | 1810033B17Rik | RIKEN cDNA 1810033B17 gene |
| ENSMUSG00000038267 | -2.07 | -4.19 | 1.86E-17 | Slc22a23 | solute carrier family 22, member 23 |
| ENSMUSG00000083161 | -2.07 | -4.19 | 4.22E-20 | NA | NA |
| ENSMUSG00000063856 | -2.06 | -4.18 | 1.74E-20 | Gpx1 | glutathione peroxidase 1 |
| ENSMUSG00000021258 | -2.06 | -4.18 | 1.07E-55 | Ccnk | cyclin K |
| ENSMUSG00000000706 | -2.06 | -4.18 | 1.22E-04 | Btn1a1 | butyrophilin, subfamily 1, member A1 |
| ENSMUSG00000008393 | -2.06 | -4.18 | 5.10E-14 | Carhsp1 | calcium regulated heat stable protein 1 |
| ENSMUSG00000030688 | -2.06 | -4.18 | 1.26E-25 | Stard10 | START domain containing 10 |
| ENSMUSG00000067613 | -2.06 | -4.17 | 2.26E-07 | 5430421N21Rik | RIKEN cDNA 5430421N21 gene |
| ENSMUSG00000074476 | -2.06 | -4.17 | 7.49E-17 | Spc24 | SPC24, NDC80 kinetochore complex component, homolog (S. cerevisiae) |
| ENSMUSG00000086453 | -2.06 | -4.17 | 2.81E-08 | NA | NA |
| ENSMUSG00000028684 | -2.06 | -4.17 | 2.89E-25 | Urod | uroporphyrinogen decarboxylase |
| ENSMUSG00000090174 | -2.06 | -4.17 | 4.22E-03 | NA | NA |
| ENSMUSG00000055805 | -2.06 | -4.17 | 3.89E-41 | Fmnl1 | formin-like 1 |
| ENSMUSG00000007080 | -2.06 | -4.16 | 8.13E-19 | Pole | polymerase (DNA directed), epsilon |
| ENSMUSG00000081746 | -2.06 | -4.16 | 6.89E-03 | NA | NA |
| ENSMUSG00000072589 | -2.06 | -4.16 | 1.33E-07 | NA | NA |
| ENSMUSG00000026450 | -2.06 | -4.16 | 5.42E-12 | Chit1 | chitinase 1 (chitotriosidase) |
| ENSMUSG00000024042 | -2.06 | -4.16 | 5.17E-21 | Sik1 | salt inducible kinase 1 |
| ENSMUSG00000040725 | -2.05 | -4.15 | 2.62E-45 | Hnrnpul1 | heterogeneous nuclear ribonucleoprotein U-like 1 |
| ENSMUSG00000030708 | -2.05 | -4.15 | 1.91E-10 | Dnajb13 | DnaJ (Hsp40) related, subfamily B, member 13 |
| ENSMUSG00000031452 | -2.05 | -4.15 | 9.59E-04 | 1700029H14Rik | RIKEN cDNA 1700029H14 gene |
| ENSMUSG00000050751 | -2.05 | -4.15 | 9.57E-12 | Pgbd5 | piggyBac transposable element derived 5 |
| ENSMUSG00000013766 | -2.05 | -4.15 | 1.77E-05 | Ly6g6e | lymphocyte antigen 6 complex, locus G6E |
| ENSMUSG00000083599 | -2.05 | -4.14 | 1.21E-28 | NA | NA |
| ENSMUSG00000053714 | -2.05 | -4.14 | 1.02E-04 | NA | NA |
| ENSMUSG00000069265 | -2.05 | -4.13 | 6.45E-03 | NA | NA |
| ENSMUSG00000043789 | -2.04 | -4.13 | 5.74E-06 | Vwce | von Willebrand factor C and EGF domains |
| ENSMUSG00000029860 | -2.04 | -4.13 | 8.38E-19 | Zyx | zyxin |
| ENSMUSG00000002658 | -2.04 | -4.12 | 1.95E-32 | Gtf2f1 | general transcription factor IIF, polypeptide 1 |
| ENSMUSG00000084055 | -2.04 | -4.12 | 2.46E-04 | NA | NA |
| ENSMUSG00000022945 | -2.04 | -4.12 | 6.90E-31 | Chaf1b | chromatin assembly factor 1, subunit B (p60) |
| ENSMUSG00000016028 | -2.04 | -4.12 | 2.51E-13 | Celsr1 | cadherin, EGF LAG seven-pass G-type receptor 1 (flamingo homolog, Drosophila) |
| ENSMUSG00000020163 | -2.04 | -4.12 | 1.58E-19 | Uqcr11 | ubiquinol-cytochrome c reductase, complex III subunit XI |
| ENSMUSG00000047798 | -2.04 | -4.12 | 2.41E-20 | Cd300lf | CD300 antigen like family member F |
| ENSMUSG00000028876 | -2.04 | -4.12 | 9.02E-03 | Epha10 | Eph receptor A10 |
| ENSMUSG00000073430 | -2.04 | -4.12 | 7.13E-07 | NA | NA |
| ENSMUSG00000086034 | -2.04 | -4.12 | 5.40E-03 | LOC102640117 | uncharacterized LOC102640117 |
| ENSMUSG00000010048 | -2.04 | -4.11 | 4.09E-19 | Ifrd2 | interferon-related developmental regulator 2 |
| ENSMUSG00000000411 | -2.04 | -4.11 | 5.94E-04 | Tssk3 | testis-specific serine kinase 3 |
| ENSMUSG00000006585 | -2.04 | -4.11 | 1.04E-11 | Cdt1 | chromatin licensing and DNA replication factor 1 |
| ENSMUSG00000075388 | -2.03 | -4.10 | 4.48E-03 | NA | NA |
| ENSMUSG00000081370 | -2.03 | -4.10 | 4.44E-24 | NA | NA |
| ENSMUSG00000024660 | -2.03 | -4.10 | 2.89E-23 | Incenp | inner centromere protein |
| ENSMUSG00000049539 | -2.03 | -4.09 | 2.76E-03 | Hist1h1a | histone cluster 1, H1a |
| ENSMUSG00000020185 | -2.03 | -4.09 | 5.56E-14 | E2f7 | E2F transcription factor 7 |
| ENSMUSG00000032572 | -2.03 | -4.09 | 2.60E-07 | Col6a4 | collagen, type VI, alpha 4 |
| ENSMUSG00000030769 | -2.03 | -4.09 | 4.84E-09 | Slc5a11 | solute carrier family 5 (sodium/glucose cotransporter), member 11 |
| ENSMUSG00000079729 | -2.03 | -4.09 | 5.14E-03 | NA | NA |
| ENSMUSG00000026198 | -2.03 | -4.09 | 1.68E-23 | Abcb6 | ATP-binding cassette, sub-family B (MDR/TAP), member 6 |
| ENSMUSG00000048445 | -2.03 | -4.09 | 5.63E-21 | Ccdc57 | coiled-coil domain containing 57 |
| ENSMUSG00000052730 | -2.03 | -4.09 | 6.85E-03 | Gm5111 | predicted gene 5111 |
| ENSMUSG00000028803 | -2.03 | -4.09 | 1.14E-27 | Nipal3 | NIPA-like domain containing 3 |
| ENSMUSG00000044867 | -2.03 | -4.08 | 4.49E-07 | NA | NA |
| ENSMUSG00000086421 | -2.03 | -4.08 | 3.84E-09 | NA | NA |
| ENSMUSG00000033220 | -2.03 | -4.08 | 5.75E-20 | Rac2 | RAS-related C3 botulinum substrate 2 |
| ENSMUSG00000016757 | -2.03 | -4.08 | 1.74E-27 | Ttll12 | tubulin tyrosine ligase-like family, member 12 |
| ENSMUSG00000028337 | -2.03 | -4.07 | 2.47E-26 | Coro2a | coronin, actin binding protein 2A |
| ENSMUSG00000083207 | -2.02 | -4.07 | 3.42E-07 | NA | NA |
| ENSMUSG00000061311 | -2.02 | -4.06 | 5.89E-06 | Rag1 | recombination activating gene 1 |
| ENSMUSG00000038943 | -2.02 | -4.06 | 1.45E-16 | Prc1 | protein regulator of cytokinesis 1 |
| ENSMUSG00000032397 | -2.02 | -4.06 | 8.95E-19 | Tipin | timeless interacting protein |
| ENSMUSG00000026354 | -2.02 | -4.06 | 2.13E-06 | Lct | lactase |
| ENSMUSG00000049092 | -2.02 | -4.06 | 1.14E-10 | Gpr137c | G protein-coupled receptor 137C |
| ENSMUSG00000032218 | -2.02 | -4.06 | 1.37E-18 | Ccnb2 | cyclin B2 |
| ENSMUSG00000022438 | -2.02 | -4.05 | 2.05E-23 | Parvb | parvin, beta |
| ENSMUSG00000029608 | -2.02 | -4.05 | 2.88E-05 | Rph3a | rabphilin 3A |
| ENSMUSG00000064120 | -2.02 | -4.05 | 1.77E-18 | Mocs1 | molybdenum cofactor synthesis 1 |
| ENSMUSG00000083218 | -2.02 | -4.05 | 4.99E-08 | NA | NA |
| ENSMUSG00000075226 | -2.02 | -4.05 | 1.14E-04 | NA | NA |
| ENSMUSG00000086606 | -2.02 | -4.04 | 4.77E-03 | NA | NA |
| ENSMUSG00000030994 | -2.02 | -4.04 | 1.85E-03 | D7Ertd443e | DNA segment, Chr 7, ERATO Doi 443, expressed |
| ENSMUSG00000005198 | -2.02 | -4.04 | 7.52E-15 | Polr2a | polymerase (RNA) II (DNA directed) polypeptide A |
| ENSMUSG00000035183 | -2.02 | -4.04 | 9.81E-19 | Slc24a5 | solute carrier family 24, member 5 |
| ENSMUSG00000032387 | -2.01 | -4.04 | 1.22E-25 | NA | NA |
| ENSMUSG00000068417 | -2.01 | -4.04 | 8.01E-16 | Pnp2 | purine-nucleoside phosphorylase 2 |
| ENSMUSG00000081911 | -2.01 | -4.04 | 1.71E-07 | NA | NA |
| ENSMUSG00000048699 | -2.01 | -4.04 | 1.00E-06 | 4732456N10Rik | RIKEN cDNA 4732456N10 gene |
| ENSMUSG00000027845 | -2.01 | -4.03 | 2.33E-21 | Dclre1b | DNA cross-link repair 1B, PSO2 homolog (S. cerevisiae) |
| ENSMUSG00000040828 | -2.01 | -4.03 | 3.05E-03 | Catsperd | catsper channel auxiliary subunit delta |
| ENSMUSG00000034278 | -2.01 | -4.03 | 4.39E-14 | Dnajc17 | DnaJ (Hsp40) homolog, subfamily C, member 17 |
| ENSMUSG00000037451 | -2.01 | -4.02 | 6.66E-08 | Slc22a20 | solute carrier family 22 (organic anion transporter), member 20 |
| ENSMUSG00000056738 | -2.01 | -4.02 | 5.80E-09 | A730036I17Rik | RIKEN cDNA A730036I17 gene |
| ENSMUSG00000022659 | -2.01 | -4.02 | 9.42E-15 | Gcsam | germinal center associated, signaling and motility |
| ENSMUSG00000071202 | -2.01 | -4.02 | 7.32E-06 | Ccdc78 | coiled-coil domain containing 78 |
| ENSMUSG00000035112 | -2.01 | -4.02 | 1.10E-10 | Wnk4 | WNK lysine deficient protein kinase 4 |
| ENSMUSG00000046432 | -2.01 | -4.02 | 5.16E-16 | Ngfrap1 | nerve growth factor receptor (TNFRSF16) associated protein 1 |
| ENSMUSG00000002835 | -2.01 | -4.02 | 1.09E-34 | Chaf1a | chromatin assembly factor 1, subunit A (p150) |
| ENSMUSG00000020732 | -2.01 | -4.02 | 1.03E-17 | Rab37 | RAB37, member of RAS oncogene family |
| ENSMUSG00000022422 | -2.01 | -4.01 | 7.38E-13 | Dscc1 | defective in sister chromatid cohesion 1 homolog (S. cerevisiae) |
| ENSMUSG00000024036 | -2.00 | -4.01 | 3.40E-23 | Slc37a1 | solute carrier family 37 (glycerol-3-phosphate transporter), member 1 |
| ENSMUSG00000037337 | -2.00 | -4.01 | 1.66E-38 | Map4k1 | mitogen-activated protein kinase kinase kinase kinase 1 |
| ENSMUSG00000042817 | -2.00 | -4.01 | 7.66E-18 | Flt3 | FMS-like tyrosine kinase 3 |
| ENSMUSG00000023048 | -2.00 | -4.00 | 1.28E-21 | Prr13 | proline rich 13 |
| ENSMUSG00000084756 | -2.00 | -4.00 | 4.73E-09 | NA | NA |
| ENSMUSG00000058435 | -2.00 | -4.00 | 2.95E-04 | Btnl4 | butyrophilin-like 4 |
| ENSMUSG00000029601 | -2.00 | -4.00 | 3.75E-10 | Iqcd | IQ motif containing D |
| ENSMUSG00000040557 | -2.00 | -3.99 | 1.78E-30 | Wbscr27 | Williams Beuren syndrome chromosome region 27 (human) |
| ENSMUSG00000025362 | -2.00 | -3.99 | 2.86E-09 | NA | NA |
| ENSMUSG00000070803 | -2.00 | -3.99 | 3.53E-11 | Cited4 | Cbp/p300-interacting transactivator, with Glu/Asp-rich carboxy-terminal domain, 4 |
| ENSMUSG00000040434 | -2.00 | -3.99 | 2.20E-05 | Gyltl1b | glycosyltransferase-like 1B |
| ENSMUSG00000032783 | -1.99 | -3.99 | 3.69E-25 | Troap | trophinin associated protein |
| ENSMUSG00000087326 | -1.99 | -3.98 | 3.38E-03 | NA | NA |
| ENSMUSG00000003585 | -1.99 | -3.98 | 2.18E-24 | Sec14l2 | SEC14-like 2 (S. cerevisiae) |
| ENSMUSG00000089429 | -1.99 | -3.98 | 7.99E-03 | NA | NA |
| ENSMUSG00000064147 | -1.99 | -3.98 | 6.60E-12 | Rab44 | RAB44, member RAS oncogene family |
| ENSMUSG00000059336 | -1.99 | -3.98 | 6.50E-15 | Slc14a1 | solute carrier family 14 (urea transporter), member 1 |
| ENSMUSG00000032741 | -1.99 | -3.97 | 2.17E-39 | Tpcn1 | two pore channel 1 |
| ENSMUSG00000058022 | -1.99 | -3.97 | 1.17E-08 | Adtrp | androgen dependent TFPI regulating protein |
| ENSMUSG00000035179 | -1.99 | -3.97 | 7.03E-05 | Ppp1r32 | protein phosphatase 1, regulatory subunit 32 |
| ENSMUSG00000024002 | -1.99 | -3.97 | 3.40E-22 | Brd4 | bromodomain containing 4 |
| ENSMUSG00000041323 | -1.99 | -3.97 | 8.72E-03 | Ak7 | adenylate kinase 7 |
| ENSMUSG00000015342 | -1.99 | -3.97 | 2.78E-09 | Xk | Kell blood group precursor (McLeod phenotype) homolog |
| ENSMUSG00000029614 | -1.99 | -3.97 | 1.11E-07 | Rpl6 | ribosomal protein L6 |
| ENSMUSG00000088211 | -1.99 | -3.96 | 7.00E-04 | NA | NA |
| ENSMUSG00000080829 | -1.98 | -3.96 | 1.33E-07 | NA | NA |
| ENSMUSG00000057863 | -1.98 | -3.96 | 2.16E-08 | NA | NA |
| ENSMUSG00000031756 | -1.98 | -3.95 | 3.28E-20 | Cenpn | centromere protein N |
| ENSMUSG00000054889 | -1.98 | -3.95 | 5.74E-09 | Dsp | desmoplakin |
| ENSMUSG00000020990 | -1.98 | -3.95 | 6.48E-12 | Cdkl1 | cyclin-dependent kinase-like 1 (CDC2-related kinase) |
| ENSMUSG00000021676 | -1.98 | -3.95 | 4.69E-16 | Iqgap2 | IQ motif containing GTPase activating protein 2 |
| ENSMUSG00000036959 | -1.98 | -3.94 | 2.26E-16 | Bcorl1 | BCL6 co-repressor-like 1 |
| ENSMUSG00000044352 | -1.98 | -3.94 | 7.28E-11 | Sowaha | sosondowah ankyrin repeat domain family member A |
| ENSMUSG00000025726 | -1.98 | -3.94 | 1.02E-03 | Slc28a1 | solute carrier family 28 (sodium-coupled nucleoside transporter), member 1 |
| ENSMUSG00000079334 | -1.98 | -3.94 | 4.66E-26 | Nat6 | N-acetyltransferase 6 |
| ENSMUSG00000062588 | -1.98 | -3.94 | 4.29E-08 | NA | NA |
| ENSMUSG00000085603 | -1.98 | -3.94 | 7.04E-06 | NA | NA |
| ENSMUSG00000082487 | -1.98 | -3.94 | 3.46E-03 | NA | NA |
| ENSMUSG00000072553 | -1.98 | -3.93 | 2.02E-03 | Gm525 | predicted gene 525 |
| ENSMUSG00000060216 | -1.98 | -3.93 | 5.70E-27 | Arrb2 | arrestin, beta 2 |
| ENSMUSG00000021313 | -1.97 | -3.93 | 8.22E-04 | Ryr2 | ryanodine receptor 2, cardiac |
| ENSMUSG00000020541 | -1.97 | -3.92 | 1.72E-22 | Tom1l1 | target of myb1-like 1 (chicken) |
| ENSMUSG00000008193 | -1.97 | -3.92 | 4.98E-15 | Spib | Spi-B transcription factor (Spi-1/PU.1 related) |
| ENSMUSG00000083043 | -1.97 | -3.92 | 1.71E-03 | NA | NA |
| ENSMUSG00000063952 | -1.97 | -3.92 | 7.68E-24 | Brpf3 | bromodomain and PHD finger containing, 3 |
| ENSMUSG00000047394 | -1.97 | -3.92 | 4.18E-07 | Odf3b | outer dense fiber of sperm tails 3B |
| ENSMUSG00000018659 | -1.97 | -3.92 | 3.11E-14 | Pnpo | pyridoxine 5'-phosphate oxidase |
| ENSMUSG00000002489 | -1.97 | -3.92 | 2.44E-12 | Tiam1 | T cell lymphoma invasion and metastasis 1 |
[truncated: 182,779 more chars]
