# Supplementary material for: Osteolytic cancer cells induce vascular/axon guidance processes in the bone/bone marrow stroma
Source: Oncotarget. 2018 Jun 22;9(48):28877–96. doi: 10.18632/oncotarget.25608 (PMC6034746; doi:10.18632/oncotarget.25608)
Supplement: Supplementary file 4 [file oncotarget-09-28877-s004.docx]

**Table S3.**

Up-regulated genes

|  | PC-3 xenografts | |  | MDA-MB231 xenografts | |  |  |  |
| --- | --- | --- | --- | --- | --- | --- | --- | --- |
| id | log2FoldChange | FC | padj | log2FoldChange | FC | padj | SYMBOL | GENENAME |
| ENSMUSG00000059256 | 4.32 | 19.98 | 3.91E-09 | 9.83 | 912.87 | 1.60E-61 | Gzmd | granzyme D |
| ENSMUSG00000022156 | 4.44 | 21.73 | 4.12E-08 | 8.61 | 389.40 | 1.92E-36 | Gzme | granzyme E |
| ENSMUSG00000047562 | 2.46 | 5.50 | 2.95E-03 | 8.50 | 362.39 | 1.52E-44 | Mmp10 | matrix metallopeptidase 10 |
| ENSMUSG00000062235 | 3.67 | 12.68 | 1.55E-05 | 7.25 | 152.74 | 2.88E-24 | NA | NA |
| ENSMUSG00000079186 | 3.37 | 10.31 | 4.54E-06 | 7.09 | 135.90 | 5.89E-31 | Gzmc | granzyme C |
| ENSMUSG00000068246 | 2.67 | 6.38 | 1.64E-05 | 6.81 | 112.15 | 8.78E-48 | Apol9b | apolipoprotein L 9b |
| ENSMUSG00000009185 | 2.46 | 5.51 | 1.88E-05 | 6.74 | 107.13 | 5.95E-50 | Ccl8 | chemokine (C-C motif) ligand 8 |
| ENSMUSG00000020581 | 4.31 | 19.79 | 8.96E-13 | 6.71 | 105.01 | 1.40E-41 | Agr2 | anterior gradient 2 |
| ENSMUSG00000089022 | 2.48 | 5.60 | 4.64E-04 | 6.64 | 99.78 | 1.39E-37 | NA | NA |
| ENSMUSG00000035352 | 2.82 | 7.07 | 3.24E-06 | 6.44 | 86.74 | 3.10E-42 | Ccl12 | chemokine (C-C motif) ligand 12 |
| ENSMUSG00000029675 | 1.83 | 3.56 | 6.16E-08 | 6.27 | 77.12 | 6.88E-118 | Eln | elastin |
| ENSMUSG00000026343 | 2.55 | 5.87 | 1.07E-03 | 6.23 | 75.23 | 2.37E-26 | Gpr39 | G protein-coupled receptor 39 |
| ENSMUSG00000006403 | 2.26 | 4.77 | 7.37E-14 | 5.85 | 57.87 | 1.43E-141 | Adamts4 | a disintegrin-like and metallopeptidase (reprolysin type) with thrombospondin type 1 motif, 4 |
| ENSMUSG00000090007 | 7.10 | 136.75 | 3.23E-32 | 5.74 | 53.49 | 4.36E-24 | NA | NA |
| ENSMUSG00000074978 | 2.71 | 6.54 | 1.47E-05 | 5.71 | 52.23 | 6.24E-31 | NA | NA |
| ENSMUSG00000048706 | 2.50 | 5.67 | 1.58E-06 | 5.53 | 46.36 | 1.39E-41 | Lurap1l | leucine rich adaptor protein 1-like |
| ENSMUSG00000031538 | 2.12 | 4.36 | 7.08E-09 | 5.39 | 41.95 | 6.07E-78 | Plat | plasminogen activator, tissue |
| ENSMUSG00000052504 | 1.06 | 2.09 | 4.11E-03 | 5.19 | 36.46 | 1.33E-79 | Epha3 | Eph receptor A3 |
| ENSMUSG00000075707 | 2.36 | 5.14 | 4.63E-04 | 5.18 | 36.36 | 2.36E-23 | Dio3 | deiodinase, iodothyronine type III |
| ENSMUSG00000065592 | 2.40 | 5.28 | 5.79E-03 | 4.97 | 31.30 | 1.75E-13 | NA | NA |
| ENSMUSG00000046182 | 2.06 | 4.17 | 5.42E-04 | 4.94 | 30.80 | 3.06E-30 | Gsg1l | GSG1-like |
| ENSMUSG00000040829 | 1.41 | 2.66 | 4.61E-05 | 4.86 | 29.00 | 1.47E-80 | Zmynd15 | zinc finger, MYND-type containing 15 |
| ENSMUSG00000081834 | 5.29 | 39.03 | 9.33E-12 | 4.73 | 26.49 | 4.09E-12 | NA | NA |
| ENSMUSG00000007888 | 1.48 | 2.79 | 4.39E-04 | 4.71 | 26.17 | 8.26E-47 | Crlf1 | cytokine receptor-like factor 1 |
| ENSMUSG00000026413 | 3.95 | 15.41 | 1.90E-10 | 4.65 | 25.15 | 9.16E-20 | Pkp1 | plakophilin 1 |
| ENSMUSG00000041482 | 1.72 | 3.30 | 2.05E-04 | 4.61 | 24.43 | 3.01E-37 | Piezo2 | piezo-type mechanosensitive ion channel component 2 |
| ENSMUSG00000004098 | 1.11 | 2.16 | 3.40E-03 | 4.56 | 23.56 | 4.54E-51 | Col5a3 | collagen, type V, alpha 3 |
| ENSMUSG00000089600 | 3.09 | 8.49 | 7.71E-04 | 4.55 | 23.35 | 1.61E-08 | NA | NA |
| ENSMUSG00000017724 | 2.19 | 4.56 | 5.16E-10 | 4.51 | 22.82 | 1.41E-64 | Etv4 | ets variant gene 4 (E1A enhancer binding protein, E1AF) |
| ENSMUSG00000022382 | 2.04 | 4.12 | 1.38E-05 | 4.51 | 22.78 | 4.11E-38 | Wnt7b | wingless-related MMTV integration site 7B |
| ENSMUSG00000027800 | 1.32 | 2.50 | 4.01E-03 | 4.41 | 21.27 | 1.74E-33 | Tm4sf1 | transmembrane 4 superfamily member 1 |
| ENSMUSG00000039209 | 1.42 | 2.68 | 1.58E-03 | 4.34 | 20.28 | 1.74E-40 | Rpl39l | ribosomal protein L39-like |
| ENSMUSG00000042834 | 1.15 | 2.21 | 6.99E-04 | 4.31 | 19.82 | 9.88E-57 | Nrep | neuronal regeneration related protein |
| ENSMUSG00000044303 | 1.56 | 2.95 | 9.50E-04 | 4.26 | 19.18 | 2.91E-36 | Cdkn2a | cyclin-dependent kinase inhibitor 2A |
| ENSMUSG00000052353 | 1.60 | 3.02 | 6.20E-04 | 4.25 | 19.07 | 2.01E-29 | 9930013L23Rik | RIKEN cDNA 9930013L23 gene |
| ENSMUSG00000029348 | 2.23 | 4.69 | 3.07E-06 | 4.24 | 18.96 | 3.99E-30 | Asphd2 | aspartate beta-hydroxylase domain containing 2 |
| ENSMUSG00000047261 | 2.24 | 4.71 | 1.05E-03 | 4.21 | 18.56 | 2.00E-15 | Gap43 | growth associated protein 43 |
| ENSMUSG00000032135 | 1.16 | 2.24 | 6.06E-05 | 4.13 | 17.45 | 7.14E-71 | Mcam | melanoma cell adhesion molecule |
| ENSMUSG00000004892 | 3.40 | 10.59 | 2.09E-12 | 4.12 | 17.43 | 1.43E-25 | Bcan | brevican |
| ENSMUSG00000001131 | 1.14 | 2.20 | 3.57E-03 | 4.09 | 16.98 | 1.23E-38 | Timp1 | tissue inhibitor of metalloproteinase 1 |
| ENSMUSG00000026548 | 1.14 | 2.20 | 5.03E-03 | 4.07 | 16.85 | 6.47E-41 | Slamf9 | SLAM family member 9 |
| ENSMUSG00000035131 | 2.37 | 5.15 | 1.48E-05 | 4.04 | 16.43 | 1.60E-21 | Brinp3 | bone morphogenetic protein/retinoic acid inducible neural specific 3 |
| ENSMUSG00000023905 | 1.01 | 2.02 | 9.61E-03 | 4.04 | 16.40 | 4.49E-42 | Tnfrsf12a | tumor necrosis factor receptor superfamily, member 12a |
| ENSMUSG00000075602 | 1.89 | 3.71 | 2.56E-05 | 4.02 | 16.23 | 1.19E-27 | Ly6a | lymphocyte antigen 6 complex, locus A |
| ENSMUSG00000038775 | 1.51 | 2.84 | 9.21E-03 | 4.00 | 16.02 | 2.01E-22 | Vill | villin-like |
| ENSMUSG00000065487 | 2.47 | 5.53 | 2.03E-03 | 3.99 | 15.90 | 3.46E-10 | NA | NA |
| ENSMUSG00000040703 | 1.87 | 3.66 | 5.52E-05 | 3.95 | 15.43 | 2.95E-26 | Cyp2s1 | cytochrome P450, family 2, subfamily s, polypeptide 1 |
| ENSMUSG00000020592 | 1.48 | 2.80 | 1.08E-08 | 3.86 | 14.51 | 7.20E-78 | Sdc1 | syndecan 1 |
| ENSMUSG00000026043 | 1.12 | 2.17 | 1.23E-03 | 3.85 | 14.39 | 1.18E-42 | Col3a1 | collagen, type III, alpha 1 |
| ENSMUSG00000074676 | 1.85 | 3.61 | 8.39E-07 | 3.83 | 14.22 | 4.38E-39 | Foxs1 | forkhead box S1 |
| ENSMUSG00000074971 | 1.29 | 2.44 | 9.88E-05 | 3.83 | 14.20 | 5.18E-47 | Fibin | fin bud initiation factor homolog (zebrafish) |
| ENSMUSG00000027670 | 2.09 | 4.27 | 2.04E-08 | 3.83 | 14.18 | 6.00E-38 | Ocstamp | osteoclast stimulatory transmembrane protein |
| ENSMUSG00000035165 | 1.05 | 2.07 | 9.28E-03 | 3.82 | 14.15 | 7.86E-43 | Kcne3 | potassium voltage-gated channel, Isk-related subfamily, gene 3 |
| ENSMUSG00000039405 | 1.01 | 2.01 | 1.74E-03 | 3.80 | 13.97 | 4.32E-51 | Prss23 | protease, serine 23 |
| ENSMUSG00000048126 | 1.12 | 2.17 | 2.10E-05 | 3.79 | 13.82 | 3.68E-71 | Col6a3 | collagen, type VI, alpha 3 |
| ENSMUSG00000001168 | 2.42 | 5.36 | 8.55E-03 | 3.78 | 13.77 | 6.91E-07 | Oas1h | 2'-5' oligoadenylate synthetase 1H |
| ENSMUSG00000084277 | 2.84 | 7.18 | 1.15E-04 | 3.77 | 13.66 | 8.75E-10 | NA | NA |
| ENSMUSG00000001435 | 1.78 | 3.43 | 4.17E-09 | 3.72 | 13.19 | 2.40E-53 | Col18a1 | collagen, type XVIII, alpha 1 |
| ENSMUSG00000031239 | 1.20 | 2.29 | 3.01E-04 | 3.70 | 12.97 | 5.20E-44 | Itm2a | integral membrane protein 2A |
| ENSMUSG00000036912 | 1.93 | 3.80 | 4.99E-03 | 3.69 | 12.89 | 9.24E-12 | Piwil4 | piwi-like RNA-mediated gene silencing 4 |
| ENSMUSG00000050288 | 2.43 | 5.38 | 1.17E-05 | 3.66 | 12.65 | 6.47E-16 | Fzd2 | frizzled homolog 2 (Drosophila) |
| ENSMUSG00000066861 | 1.70 | 3.26 | 2.68E-06 | 3.65 | 12.54 | 1.77E-37 | Oas1g | 2'-5' oligoadenylate synthetase 1G |
| ENSMUSG00000015852 | 1.97 | 3.92 | 3.48E-04 | 3.64 | 12.45 | 1.28E-16 | Fcrls | Fc receptor-like S, scavenger receptor |
| ENSMUSG00000032085 | 1.41 | 2.67 | 1.41E-07 | 3.60 | 12.11 | 1.62E-62 | Tagln | transgelin |
| ENSMUSG00000056481 | 1.89 | 3.71 | 5.29E-05 | 3.58 | 11.95 | 1.57E-20 | Cd248 | CD248 antigen, endosialin |
| ENSMUSG00000042734 | 1.41 | 2.67 | 6.64E-05 | 3.57 | 11.88 | 1.17E-39 | Ttc9 | tetratricopeptide repeat domain 9 |
| ENSMUSG00000054196 | 1.31 | 2.48 | 9.84E-05 | 3.56 | 11.83 | 1.29E-39 | Cthrc1 | collagen triple helix repeat containing 1 |
| ENSMUSG00000062380 | 1.84 | 3.59 | 9.70E-05 | 3.52 | 11.44 | 1.08E-23 | Tubb3 | tubulin, beta 3 class III |
| ENSMUSG00000000126 | 1.80 | 3.47 | 1.81E-04 | 3.51 | 11.36 | 1.79E-19 | Wnt9a | wingless-type MMTV integration site 9A |
| ENSMUSG00000018920 | 1.11 | 2.16 | 8.11E-04 | 3.50 | 11.34 | 6.56E-40 | Cxcl16 | chemokine (C-X-C motif) ligand 16 |
| ENSMUSG00000020186 | 1.15 | 2.21 | 1.31E-05 | 3.50 | 11.29 | 6.00E-65 | Csrp2 | cysteine and glycine-rich protein 2 |
| ENSMUSG00000034394 | 1.21 | 2.32 | 2.88E-03 | 3.48 | 11.12 | 1.42E-28 | Lif | leukemia inhibitory factor |
| ENSMUSG00000048450 | 1.25 | 2.39 | 2.93E-03 | 3.47 | 11.11 | 4.42E-28 | Msx1 | msh homeobox 1 |
| ENSMUSG00000031880 | 1.34 | 2.53 | 7.47E-04 | 3.45 | 10.96 | 1.67E-28 | Rrad | Ras-related associated with diabetes |
| ENSMUSG00000053318 | 1.53 | 2.88 | 5.78E-04 | 3.41 | 10.66 | 1.38E-23 | Slamf8 | SLAM family member 8 |
| ENSMUSG00000079105 | 2.15 | 4.44 | 5.86E-03 | 3.36 | 10.27 | 3.38E-08 | C7 | complement component 7 |
| ENSMUSG00000017652 | 1.10 | 2.15 | 8.33E-03 | 3.32 | 9.99 | 1.05E-25 | Cd40 | CD40 antigen |
| ENSMUSG00000088272 | 1.39 | 2.62 | 6.30E-03 | 3.31 | 9.89 | 1.50E-15 | NA | NA |
| ENSMUSG00000027656 | 1.29 | 2.44 | 7.40E-06 | 3.29 | 9.75 | 4.12E-46 | Wisp2 | WNT1 inducible signaling pathway protein 2 |
| ENSMUSG00000053647 | 1.58 | 2.99 | 9.37E-09 | 3.27 | 9.62 | 8.49E-62 | Gper1 | G protein-coupled estrogen receptor 1 |
| ENSMUSG00000062661 | 1.14 | 2.20 | 2.77E-03 | 3.26 | 9.58 | 1.44E-30 | Ncs1 | neuronal calcium sensor 1 |
| ENSMUSG00000042428 | 1.56 | 2.95 | 1.11E-07 | 3.22 | 9.30 | 3.26E-49 | Mgat3 | mannoside acetylglucosaminyltransferase 3 |
| ENSMUSG00000056174 | 1.93 | 3.81 | 6.35E-05 | 3.21 | 9.28 | 8.71E-16 | Col8a2 | collagen, type VIII, alpha 2 |
| ENSMUSG00000023411 | 1.61 | 3.05 | 5.60E-06 | 3.18 | 9.07 | 1.07E-28 | Nfatc4 | nuclear factor of activated T cells, cytoplasmic, calcineurin dependent 4 |
| ENSMUSG00000046856 | 3.20 | 9.19 | 2.77E-04 | 3.18 | 9.06 | 3.56E-05 | Gpr1 | G protein-coupled receptor 1 |
| ENSMUSG00000023886 | 1.40 | 2.64 | 3.32E-06 | 3.17 | 8.97 | 3.04E-38 | Smoc2 | SPARC related modular calcium binding 2 |
| ENSMUSG00000031503 | 1.18 | 2.27 | 1.86E-06 | 3.15 | 8.88 | 1.95E-55 | Col4a2 | collagen, type IV, alpha 2 |
| ENSMUSG00000041696 | 1.26 | 2.40 | 6.01E-05 | 3.15 | 8.88 | 1.40E-40 | Rasl12 | RAS-like, family 12 |
| ENSMUSG00000042116 | 1.47 | 2.78 | 2.29E-05 | 3.13 | 8.75 | 1.08E-29 | Vwa1 | von Willebrand factor A domain containing 1 |
| ENSMUSG00000030606 | 1.94 | 3.84 | 5.92E-07 | 3.12 | 8.70 | 6.38E-28 | Hapln3 | hyaluronan and proteoglycan link protein 3 |
| ENSMUSG00000032271 | 1.17 | 2.26 | 3.51E-03 | 3.11 | 8.61 | 1.34E-27 | Nnmt | nicotinamide N-methyltransferase |
| ENSMUSG00000064080 | 1.72 | 3.29 | 1.36E-04 | 3.11 | 8.61 | 8.49E-17 | Fbln2 | fibulin 2 |
| ENSMUSG00000021411 | 1.66 | 3.15 | 1.85E-07 | 3.09 | 8.51 | 2.47E-34 | Pxdc1 | PX domain containing 1 |
| ENSMUSG00000025272 | 1.62 | 3.07 | 7.21E-04 | 3.03 | 8.19 | 3.08E-17 | Tro | trophinin |
| ENSMUSG00000021095 | 2.59 | 6.00 | 1.85E-03 | 3.03 | 8.15 | 1.43E-05 | Gsc | goosecoid homeobox |
| ENSMUSG00000022440 | 1.48 | 2.80 | 1.15E-07 | 3.02 | 8.11 | 2.51E-40 | C1qtnf6 | C1q and tumor necrosis factor related protein 6 |
| ENSMUSG00000040690 | 1.71 | 3.27 | 3.01E-05 | 3.00 | 7.99 | 7.22E-19 | Col16a1 | collagen, type XVI, alpha 1 |
| ENSMUSG00000040875 | 1.40 | 2.65 | 3.44E-03 | 2.99 | 7.97 | 1.50E-16 | Osbpl10 | oxysterol binding protein-like 10 |
| ENSMUSG00000023885 | 1.18 | 2.27 | 8.01E-04 | 2.98 | 7.88 | 3.55E-25 | Thbs2 | thrombospondin 2 |
| ENSMUSG00000035783 | 1.33 | 2.51 | 1.66E-07 | 2.96 | 7.77 | 6.31E-47 | Acta2 | actin, alpha 2, smooth muscle, aorta |
| ENSMUSG00000015134 | 1.30 | 2.47 | 7.64E-04 | 2.96 | 7.76 | 7.97E-23 | Aldh1a3 | aldehyde dehydrogenase family 1, subfamily A3 |
| ENSMUSG00000040289 | 1.17 | 2.26 | 7.49E-03 | 2.94 | 7.66 | 4.61E-17 | Hey1 | hairy/enhancer-of-split related with YRPW motif 1 |
| ENSMUSG00000080242 | 1.22 | 2.34 | 4.32E-04 | 2.93 | 7.63 | 4.76E-25 | Atp6v0c-ps2 | ATPase, H+ transporting, lysosomal V0 subunit C, pseudogene 2 |
| ENSMUSG00000018930 | 1.58 | 3.00 | 6.96E-03 | 2.92 | 7.57 | 1.83E-10 | Ccl4 | chemokine (C-C motif) ligand 4 |
| ENSMUSG00000027978 | 1.84 | 3.59 | 7.41E-03 | 2.92 | 7.55 | 1.20E-07 | Prss12 | protease, serine 12 neurotrypsin (motopsin) |
| ENSMUSG00000021260 | 1.14 | 2.20 | 2.01E-03 | 2.91 | 7.52 | 4.47E-25 | Hhipl1 | hedgehog interacting protein-like 1 |
| ENSMUSG00000026837 | 1.04 | 2.05 | 6.39E-04 | 2.91 | 7.50 | 7.74E-32 | Col5a1 | collagen, type V, alpha 1 |
| ENSMUSG00000038932 | 1.95 | 3.87 | 9.81E-04 | 2.90 | 7.46 | 3.51E-10 | Tcfl5 | transcription factor-like 5 (basic helix-loop-helix) |
| ENSMUSG00000052485 | 2.13 | 4.39 | 8.12E-03 | 2.89 | 7.42 | 8.41E-06 | Tmem171 | transmembrane protein 171 |
| ENSMUSG00000079017 | 1.62 | 3.08 | 1.06E-05 | 2.86 | 7.27 | 2.14E-21 | Ifi27l2a | interferon, alpha-inducible protein 27 like 2A |
| ENSMUSG00000031274 | 2.33 | 5.04 | 6.46E-05 | 2.80 | 6.99 | 5.35E-09 | Col4a5 | collagen, type IV, alpha 5 |
| ENSMUSG00000027661 | 1.04 | 2.06 | 2.41E-03 | 2.80 | 6.98 | 2.59E-26 | Slc2a10 | solute carrier family 2 (facilitated glucose transporter), member 10 |
| ENSMUSG00000002847 | 1.39 | 2.62 | 2.08E-04 | 2.79 | 6.94 | 1.15E-21 | Pla1a | phospholipase A1 member A |
| ENSMUSG00000036995 | 1.42 | 2.68 | 8.53E-03 | 2.78 | 6.86 | 2.28E-10 | Asap3 | ArfGAP with SH3 domain, ankyrin repeat and PH domain 3 |
| ENSMUSG00000089547 | 3.75 | 13.47 | 1.00E-05 | 2.78 | 6.85 | 2.63E-04 | NA | NA |
| ENSMUSG00000020695 | 1.31 | 2.49 | 4.30E-06 | 2.77 | 6.82 | 1.20E-32 | Mrc2 | mannose receptor, C type 2 |
| ENSMUSG00000020099 | 1.29 | 2.45 | 2.60E-04 | 2.77 | 6.80 | 1.73E-21 | Unc5b | unc-5 homolog B (C. elegans) |
| ENSMUSG00000052026 | 1.52 | 2.86 | 1.29E-04 | 2.76 | 6.80 | 8.64E-19 | Slc6a7 | solute carrier family 6 (neurotransmitter transporter, L-proline), member 7 |
| ENSMUSG00000020241 | 1.20 | 2.31 | 1.78E-05 | 2.76 | 6.77 | 2.46E-33 | Col6a2 | collagen, type VI, alpha 2 |
| ENSMUSG00000072528 | 2.04 | 4.11 | 9.73E-03 | 2.75 | 6.74 | 1.87E-05 | NA | NA |
| ENSMUSG00000006369 | 1.76 | 3.39 | 2.80E-07 | 2.74 | 6.69 | 2.05E-22 | Fbln1 | fibulin 1 |
| ENSMUSG00000032997 | 1.88 | 3.67 | 7.83E-08 | 2.73 | 6.65 | 1.05E-21 | Chpf | chondroitin polymerizing factor |
| ENSMUSG00000032011 | 1.50 | 2.83 | 3.32E-05 | 2.73 | 6.64 | 3.42E-20 | Thy1 | thymus cell antigen 1, theta |
| ENSMUSG00000034675 | 1.25 | 2.38 | 3.06E-03 | 2.73 | 6.63 | 2.35E-15 | Dbn1 | drebrin 1 |
| ENSMUSG00000052951 | 1.36 | 2.56 | 1.34E-03 | 2.72 | 6.58 | 5.05E-18 | NA | NA |
| ENSMUSG00000021381 | 3.68 | 12.85 | 3.31E-05 | 2.72 | 6.57 | 7.21E-04 | Barx1 | BarH-like homeobox 1 |
| ENSMUSG00000047686 | 1.69 | 3.23 | 2.81E-03 | 2.71 | 6.55 | 5.33E-10 | Zcchc5 | zinc finger, CCHC domain containing 5 |
| ENSMUSG00000040164 | 2.05 | 4.13 | 2.09E-04 | 2.71 | 6.53 | 5.82E-10 | Kcns1 | K+ voltage-gated channel, subfamily S, 1 |
| ENSMUSG00000036459 | 1.49 | 2.82 | 3.55E-06 | 2.71 | 6.52 | 2.06E-26 | Wtip | WT1-interacting protein |
| ENSMUSG00000000957 | 1.40 | 2.64 | 5.91E-06 | 2.70 | 6.51 | 1.17E-26 | Mmp14 | matrix metallopeptidase 14 (membrane-inserted) |
| ENSMUSG00000028167 | 2.07 | 4.21 | 3.02E-05 | 2.70 | 6.51 | 2.51E-11 | Bdh2 | 3-hydroxybutyrate dehydrogenase, type 2 |
| ENSMUSG00000039476 | 2.01 | 4.04 | 8.11E-08 | 2.70 | 6.51 | 2.97E-19 | Prrx2 | paired related homeobox 2 |
| ENSMUSG00000020473 | 1.76 | 3.38 | 1.21E-05 | 2.69 | 6.45 | 6.50E-16 | Aebp1 | AE binding protein 1 |
| ENSMUSG00000036412 | 1.69 | 3.22 | 6.38E-04 | 2.69 | 6.44 | 2.34E-11 | Arsi | arylsulfatase i |
| ENSMUSG00000060240 | 2.86 | 7.26 | 1.22E-04 | 2.68 | 6.39 | 2.71E-05 | Cend1 | cell cycle exit and neuronal differentiation 1 |
| ENSMUSG00000000693 | 1.01 | 2.02 | 1.62E-04 | 2.66 | 6.34 | 1.77E-34 | Loxl3 | lysyl oxidase-like 3 |
| ENSMUSG00000032911 | 1.69 | 3.22 | 2.93E-06 | 2.66 | 6.34 | 2.40E-19 | Cspg4 | chondroitin sulfate proteoglycan 4 |
| ENSMUSG00000004267 | 1.28 | 2.42 | 2.81E-04 | 2.66 | 6.32 | 1.18E-21 | Eno2 | enolase 2, gamma neuronal |
| ENSMUSG00000030519 | 2.41 | 5.31 | 8.29E-05 | 2.66 | 6.30 | 1.44E-07 | Apba2 | amyloid beta (A4) precursor protein-binding, family A, member 2 |
| ENSMUSG00000023191 | 1.45 | 2.74 | 7.47E-07 | 2.65 | 6.26 | 1.81E-28 | Leprel2 | leprecan-like 2 |
| ENSMUSG00000057751 | 1.94 | 3.84 | 6.91E-04 | 2.62 | 6.15 | 5.20E-08 | Megf6 | multiple EGF-like-domains 6 |
| ENSMUSG00000029762 | 1.12 | 2.17 | 7.98E-03 | 2.62 | 6.14 | 7.62E-15 | Akr1b8 | aldo-keto reductase family 1, member B8 |
| ENSMUSG00000030281 | 1.75 | 3.37 | 5.13E-05 | 2.59 | 6.04 | 2.13E-13 | Il17rc | interleukin 17 receptor C |
| ENSMUSG00000041046 | 1.59 | 3.01 | 1.88E-03 | 2.58 | 5.99 | 3.23E-10 | Ramp3 | receptor (calcitonin) activity modifying protein 3 |
| ENSMUSG00000037813 | 1.52 | 2.86 | 2.77E-05 | 2.58 | 5.98 | 2.72E-20 | D630003M21Rik | RIKEN cDNA D630003M21 gene |
| ENSMUSG00000031740 | 1.42 | 2.68 | 2.33E-06 | 2.57 | 5.95 | 1.57E-25 | Mmp2 | matrix metallopeptidase 2 |
| ENSMUSG00000030772 | 1.18 | 2.26 | 6.81E-04 | 2.56 | 5.91 | 1.32E-19 | Dkk3 | dickkopf homolog 3 (Xenopus laevis) |
| ENSMUSG00000027603 | 1.25 | 2.38 | 2.68E-04 | 2.56 | 5.90 | 1.82E-25 | Ggt7 | gamma-glutamyltransferase 7 |
| ENSMUSG00000081520 | 2.18 | 4.52 | 3.09E-03 | 2.55 | 5.88 | 1.89E-05 | NA | NA |
| ENSMUSG00000072941 | 1.87 | 3.65 | 5.80E-03 | 2.54 | 5.84 | 1.01E-05 | Sod3 | superoxide dismutase 3, extracellular |
| ENSMUSG00000032925 | 1.38 | 2.60 | 3.56E-03 | 2.54 | 5.83 | 6.96E-11 | Itgbl1 | integrin, beta-like 1 |
| ENSMUSG00000026278 | 1.47 | 2.77 | 6.96E-05 | 2.54 | 5.82 | 1.07E-17 | Bok | BCL2-related ovarian killer protein |
| ENSMUSG00000038390 | 1.27 | 2.41 | 2.84E-05 | 2.53 | 5.78 | 3.81E-27 | Gpr162 | G protein-coupled receptor 162 |
| ENSMUSG00000059022 | 1.48 | 2.79 | 9.87E-04 | 2.52 | 5.73 | 1.29E-12 | Kcp | kielin/chordin-like protein |
| ENSMUSG00000021806 | 1.17 | 2.26 | 2.35E-04 | 2.51 | 5.69 | 4.99E-22 | Nid2 | nidogen 2 |
| ENSMUSG00000031595 | 1.60 | 3.03 | 3.30E-05 | 2.50 | 5.67 | 2.44E-15 | Pdgfrl | platelet-derived growth factor receptor-like |
| ENSMUSG00000010830 | 1.79 | 3.45 | 8.84E-08 | 2.49 | 5.61 | 7.65E-20 | Kdelr3 | KDEL (Lys-Asp-Glu-Leu) endoplasmic reticulum protein retention receptor 3 |
| ENSMUSG00000021253 | 1.37 | 2.58 | 5.27E-05 | 2.49 | 5.61 | 3.20E-19 | Tgfb3 | transforming growth factor, beta 3 |
| ENSMUSG00000024168 | 1.07 | 2.11 | 6.48E-04 | 2.49 | 5.61 | 6.90E-23 | Tmem204 | transmembrane protein 204 |
| ENSMUSG00000078200 | 1.42 | 2.67 | 6.79E-04 | 2.47 | 5.56 | 1.61E-16 | NA | NA |
| ENSMUSG00000040606 | 1.09 | 2.13 | 6.56E-04 | 2.47 | 5.55 | 4.53E-22 | Kazn | kazrin, periplakin interacting protein |
| ENSMUSG00000051048 | 1.22 | 2.33 | 2.62E-03 | 2.47 | 5.55 | 3.78E-14 | P4ha3 | procollagen-proline, 2-oxoglutarate 4-dioxygenase (proline 4-hydroxylase), alpha polypeptide III |
| ENSMUSG00000023232 | 1.21 | 2.31 | 5.08E-03 | 2.45 | 5.48 | 1.58E-12 | Serinc2 | serine incorporator 2 |
| ENSMUSG00000024440 | 1.12 | 2.17 | 6.15E-05 | 2.45 | 5.48 | 1.35E-30 | Pcdh12 | protocadherin 12 |
| ENSMUSG00000035274 | 1.36 | 2.56 | 5.96E-04 | 2.46 | 5.48 | 1.95E-14 | Tpbg | trophoblast glycoprotein |
| ENSMUSG00000005952 | 2.39 | 5.25 | 2.33E-03 | 2.45 | 5.47 | 1.91E-04 | Trpv1 | transient receptor potential cation channel, subfamily V, member 1 |
| ENSMUSG00000041801 | 1.50 | 2.84 | 1.31E-05 | 2.44 | 5.42 | 6.52E-18 | Phlda3 | pleckstrin homology-like domain, family A, member 3 |
| ENSMUSG00000035914 | 1.04 | 2.06 | 1.87E-03 | 2.42 | 5.35 | 6.63E-19 | Cd276 | CD276 antigen |
| ENSMUSG00000078794 | 1.73 | 3.32 | 3.26E-04 | 2.42 | 5.35 | 1.37E-09 | Dact3 | dapper homolog 3, antagonist of beta-catenin (xenopus) |
| ENSMUSG00000008136 | 1.15 | 2.22 | 1.09E-03 | 2.40 | 5.26 | 3.78E-17 | Fhl2 | four and a half LIM domains 2 |
| ENSMUSG00000083757 | 1.77 | 3.42 | 2.62E-04 | 2.40 | 5.26 | 3.25E-09 | NA | NA |
| ENSMUSG00000050505 | 2.36 | 5.14 | 6.06E-03 | 2.37 | 5.18 | 1.38E-03 | Pcdh20 | protocadherin 20 |
| ENSMUSG00000029126 | 1.24 | 2.36 | 2.32E-05 | 2.37 | 5.17 | 5.28E-24 | Nsg1 | neuron specific gene family member 1 |
| ENSMUSG00000072812 | 1.44 | 2.71 | 2.09E-03 | 2.37 | 5.16 | 8.73E-10 | Ahnak2 | AHNAK nucleoprotein 2 |
| ENSMUSG00000004473 | 1.21 | 2.32 | 6.32E-05 | 2.36 | 5.12 | 1.64E-21 | Clec11a | C-type lectin domain family 11, member a |
| ENSMUSG00000035279 | 1.58 | 2.98 | 3.47E-03 | 2.35 | 5.09 | 2.07E-07 | Ssc5d | scavenger receptor cysteine rich domain containing (5 domains) |
| ENSMUSG00000049939 | 1.46 | 2.74 | 4.95E-03 | 2.35 | 5.09 | 1.30E-09 | Lrrc4 | leucine rich repeat containing 4 |
| ENSMUSG00000038400 | 1.75 | 3.36 | 9.15E-09 | 2.35 | 5.08 | 2.02E-21 | Pmepa1 | prostate transmembrane protein, androgen induced 1 |
| ENSMUSG00000031253 | 1.24 | 2.36 | 1.19E-04 | 2.33 | 5.03 | 9.28E-19 | Srpx2 | sushi-repeat-containing protein, X-linked 2 |
| ENSMUSG00000006651 | 1.52 | 2.86 | 1.81E-05 | 2.32 | 4.99 | 2.46E-16 | Aplp1 | amyloid beta (A4) precursor-like protein 1 |
| ENSMUSG00000046031 | 1.31 | 2.47 | 9.73E-03 | 2.31 | 4.96 | 3.87E-09 | Fam26f | family with sequence similarity 26, member F |
| ENSMUSG00000085988 | 1.25 | 2.39 | 2.11E-03 | 2.31 | 4.96 | 1.78E-13 | NA | NA |
| ENSMUSG00000034161 | 1.68 | 3.20 | 5.68E-06 | 2.31 | 4.95 | 1.21E-15 | Scx | scleraxis |
| ENSMUSG00000029581 | 1.31 | 2.47 | 9.80E-04 | 2.29 | 4.90 | 2.27E-12 | Fscn1 | fascin homolog 1, actin bundling protein (Strongylocentrotus purpuratus) |
| ENSMUSG00000034040 | 1.39 | 2.63 | 4.18E-04 | 2.29 | 4.89 | 5.43E-13 | Wbscr17 | Williams-Beuren syndrome chromosome region 17 homolog (human) |
| ENSMUSG00000040323 | 1.42 | 2.68 | 4.66E-05 | 2.28 | 4.86 | 9.19E-16 | NA | NA |
| ENSMUSG00000075012 | 1.66 | 3.16 | 5.81E-04 | 2.27 | 4.83 | 4.36E-09 | Fjx1 | four jointed box 1 (Drosophila) |
| ENSMUSG00000055254 | 1.66 | 3.16 | 1.95E-04 | 2.26 | 4.79 | 4.69E-10 | Ntrk2 | neurotrophic tyrosine kinase, receptor, type 2 |
| ENSMUSG00000027314 | 1.12 | 2.17 | 9.65E-05 | 2.26 | 4.78 | 6.68E-24 | Dll4 | delta-like 4 (Drosophila) |
| ENSMUSG00000043895 | 1.29 | 2.44 | 3.02E-05 | 2.25 | 4.76 | 1.07E-19 | S1pr2 | sphingosine-1-phosphate receptor 2 |
| ENSMUSG00000028111 | 1.35 | 2.54 | 1.53E-03 | 2.23 | 4.70 | 2.48E-10 | Ctsk | cathepsin K |
| ENSMUSG00000054675 | 1.52 | 2.88 | 2.69E-08 | 2.23 | 4.70 | 1.50E-23 | Tmem119 | transmembrane protein 119 |
| ENSMUSG00000028597 | 1.44 | 2.72 | 6.20E-06 | 2.21 | 4.64 | 9.17E-18 | Gpx7 | glutathione peroxidase 7 |
| ENSMUSG00000070867 | 1.51 | 2.86 | 1.81E-04 | 2.21 | 4.62 | 2.02E-11 | Trabd2b | TraB domain containing 2B |
| ENSMUSG00000023972 | 1.31 | 2.49 | 2.44E-05 | 2.20 | 4.60 | 5.66E-18 | Ptk7 | PTK7 protein tyrosine kinase 7 |
| ENSMUSG00000028214 | 1.11 | 2.16 | 5.47E-03 | 2.20 | 4.60 | 3.88E-12 | Gem | GTP binding protein (gene overexpressed in skeletal muscle) |
| ENSMUSG00000030470 | 1.47 | 2.77 | 1.89E-03 | 2.20 | 4.60 | 2.19E-08 | Csrp3 | cysteine and glycine-rich protein 3 |
| ENSMUSG00000007039 | 1.65 | 3.14 | 3.90E-08 | 2.19 | 4.57 | 1.30E-19 | Ddah2 | dimethylarginine dimethylaminohydrolase 2 |
| ENSMUSG00000033595 | 1.99 | 3.98 | 2.81E-03 | 2.19 | 4.57 | 9.25E-05 | Lgi3 | leucine-rich repeat LGI family, member 3 |
| ENSMUSG00000032491 | 1.54 | 2.91 | 1.29E-05 | 2.18 | 4.55 | 5.67E-15 | Nradd | neurotrophin receptor associated death domain |
| ENSMUSG00000001555 | 1.75 | 3.37 | 1.14E-07 | 2.18 | 4.54 | 1.27E-15 | Fkbp10 | FK506 binding protein 10 |
| ENSMUSG00000028108 | 1.43 | 2.70 | 3.51E-08 | 2.18 | 4.53 | 7.91E-25 | Ecm1 | extracellular matrix protein 1 |
| ENSMUSG00000032122 | 1.14 | 2.20 | 6.52E-06 | 2.18 | 4.52 | 2.71E-26 | Slc37a2 | solute carrier family 37 (glycerol-3-phosphate transporter), member 2 |
| ENSMUSG00000062694 | 1.34 | 2.53 | 6.44E-04 | 2.17 | 4.49 | 4.78E-12 | Cav3 | caveolin 3 |
| ENSMUSG00000041559 | 1.61 | 3.06 | 2.00E-03 | 2.16 | 4.46 | 9.58E-07 | Fmod | fibromodulin |
| ENSMUSG00000012017 | 1.60 | 3.02 | 6.64E-05 | 2.15 | 4.44 | 1.15E-10 | Scarf2 | scavenger receptor class F, member 2 |
| ENSMUSG00000020811 | 1.37 | 2.58 | 2.47E-05 | 2.15 | 4.43 | 9.57E-19 | Wscd1 | WSC domain containing 1 |
| ENSMUSG00000001473 | 1.87 | 3.67 | 7.01E-10 | 2.14 | 4.41 | 7.06E-18 | Tubb6 | tubulin, beta 6 class V |
| ENSMUSG00000078570 | 1.75 | 3.36 | 7.68E-05 | 2.13 | 4.38 | 5.91E-09 | 1110065P20Rik | RIKEN cDNA 1110065P20 gene |
| ENSMUSG00000032584 | 1.45 | 2.73 | 2.14E-08 | 2.13 | 4.37 | 1.52E-24 | Mst1r | macrophage stimulating 1 receptor (c-met-related tyrosine kinase) |
| ENSMUSG00000069806 | 1.31 | 2.47 | 1.95E-04 | 2.11 | 4.33 | 7.87E-14 | NA | NA |
| ENSMUSG00000040280 | 2.02 | 4.06 | 3.12E-06 | 2.11 | 4.32 | 4.18E-09 | Ndufa4l2 | NADH dehydrogenase (ubiquinone) 1 alpha subcomplex, 4-like 2 |
| ENSMUSG00000006576 | 1.31 | 2.48 | 7.27E-05 | 2.11 | 4.31 | 2.92E-15 | Slc4a3 | solute carrier family 4 (anion exchanger), member 3 |
| ENSMUSG00000017734 | 1.34 | 2.52 | 3.56E-07 | 2.10 | 4.28 | 2.89E-23 | Dbndd2 | dysbindin (dystrobrevin binding protein 1) domain containing 2 |
| ENSMUSG00000050663 | 2.71 | 6.53 | 8.02E-04 | 2.10 | 4.28 | 3.33E-03 | Trhde | TRH-degrading enzyme |
| ENSMUSG00000050022 | 1.05 | 2.07 | 2.89E-03 | 2.09 | 4.27 | 1.20E-13 | Amz1 | archaelysin family metallopeptidase 1 |
| ENSMUSG00000018906 | 1.18 | 2.26 | 1.73E-04 | 2.09 | 4.26 | 3.37E-16 | P4ha2 | procollagen-proline, 2-oxoglutarate 4-dioxygenase (proline 4-hydroxylase), alpha II polypeptide |
| ENSMUSG00000032291 | 2.25 | 4.76 | 5.89E-04 | 2.09 | 4.26 | 1.43E-04 | Crabp1 | cellular retinoic acid binding protein I |
| ENSMUSG00000002059 | 1.27 | 2.41 | 1.77E-06 | 2.09 | 4.25 | 8.39E-23 | Rab34 | RAB34, member of RAS oncogene family |
| ENSMUSG00000036564 | 1.41 | 2.66 | 1.35E-04 | 2.09 | 4.25 | 3.56E-12 | Ndrg4 | N-myc downstream regulated gene 4 |
| ENSMUSG00000042828 | 1.31 | 2.48 | 3.91E-04 | 2.08 | 4.24 | 7.65E-12 | Trim72 | tripartite motif-containing 72 |
| ENSMUSG00000078532 | 1.42 | 2.68 | 2.93E-06 | 2.09 | 4.24 | 3.78E-19 | Nkain1 | Na+/K+ transporting ATPase interacting 1 |
| ENSMUSG00000007872 | 1.52 | 2.87 | 6.82E-06 | 2.08 | 4.22 | 1.16E-13 | Id3 | inhibitor of DNA binding 3 |
| ENSMUSG00000028978 | 1.46 | 2.75 | 1.67E-03 | 2.08 | 4.22 | 6.12E-08 | Nos3 | nitric oxide synthase 3, endothelial cell |
| ENSMUSG00000022817 | 1.01 | 2.01 | 1.59E-05 | 2.08 | 4.21 | 7.95E-28 | Itgb5 | integrin beta 5 |
| ENSMUSG00000025348 | 1.33 | 2.52 | 5.81E-05 | 2.07 | 4.21 | 2.07E-14 | Itga7 | integrin alpha 7 |
| ENSMUSG00000001507 | 1.11 | 2.16 | 1.66E-04 | 2.07 | 4.20 | 1.91E-18 | Itga3 | integrin alpha 3 |
| ENSMUSG00000029576 | 1.57 | 2.97 | 7.86E-03 | 2.07 | 4.19 | 2.06E-05 | Radil | Ras association and DIL domains |
| ENSMUSG00000029718 | 1.73 | 3.33 | 9.27E-09 | 2.07 | 4.19 | 4.46E-17 | Pcolce | procollagen C-endopeptidase enhancer protein |
| ENSMUSG00000042254 | 2.79 | 6.90 | 4.16E-04 | 2.07 | 4.19 | 3.60E-03 | Cilp | cartilage intermediate layer protein, nucleotide pyrophosphohydrolase |
| ENSMUSG00000030499 | 1.52 | 2.87 | 1.14E-05 | 2.06 | 4.16 | 4.61E-13 | Kctd15 | potassium channel tetramerisation domain containing 15 |
| ENSMUSG00000046491 | 1.62 | 3.07 | 5.65E-04 | 2.06 | 4.16 | 6.55E-08 | C1qtnf2 | C1q and tumor necrosis factor related protein 2 |
| ENSMUSG00000032725 | 1.29 | 2.44 | 1.30E-03 | 2.05 | 4.15 | 1.74E-10 | Folr2 | folate receptor 2 (fetal) |
| ENSMUSG00000022098 | 1.31 | 2.49 | 2.78E-06 | 2.05 | 4.14 | 5.03E-19 | Bmp1 | bone morphogenetic protein 1 |
| ENSMUSG00000045275 | 1.31 | 2.48 | 8.68E-03 | 2.05 | 4.14 | 1.56E-07 | Lca5l | Leber congenital amaurosis 5-like |
| ENSMUSG00000083679 | 1.96 | 3.88 | 1.93E-06 | 2.05 | 4.14 | 2.52E-09 | NA | NA |
| ENSMUSG00000082223 | 1.94 | 3.84 | 1.27E-04 | 2.04 | 4.10 | 9.45E-07 | NA | NA |
| ENSMUSG00000021268 | 1.21 | 2.31 | 6.00E-03 | 2.03 | 4.08 | 2.08E-08 | Meg3 | maternally expressed 3 |
| ENSMUSG00000022340 | 1.37 | 2.59 | 1.68E-03 | 2.02 | 4.06 | 2.70E-09 | Sybu | syntabulin (syntaxin-interacting) |
| ENSMUSG00000045672 | 1.52 | 2.86 | 2.49E-03 | 2.02 | 4.06 | 1.65E-06 | Col27a1 | collagen, type XXVII, alpha 1 |
| ENSMUSG00000029061 | 1.65 | 3.13 | 3.84E-07 | 2.02 | 4.05 | 4.13E-14 | Mmp23 | matrix metallopeptidase 23 |
| ENSMUSG00000043099 | 1.16 | 2.24 | 3.28E-03 | 2.01 | 4.04 | 2.08E-10 | Hic1 | hypermethylated in cancer 1 |
| ENSMUSG00000028040 | 2.06 | 4.16 | 6.46E-05 | 2.01 | 4.02 | 2.58E-06 | Efna4 | ephrin A4 |
| ENSMUSG00000032334 | 1.31 | 2.48 | 1.74E-04 | 2.00 | 4.00 | 4.61E-12 | Loxl1 | lysyl oxidase-like 1 |
| ENSMUSG00000041845 | 1.43 | 2.69 | 1.78E-03 | 2.00 | 4.00 | 1.05E-07 | Rhod | ras homolog gene family, member D |
| ENSMUSG00000038264 | 1.10 | 2.15 | 1.53E-04 | 2.00 | 3.99 | 6.55E-17 | Sema7a | sema domain, immunoglobulin domain (Ig), and GPI membrane anchor, (semaphorin) 7A |
| ENSMUSG00000025478 | 1.59 | 3.01 | 8.36E-05 | 1.99 | 3.98 | 2.64E-10 | Dpysl4 | dihydropyrimidinase-like 4 |
| ENSMUSG00000033542 | 1.00 | 2.00 | 2.39E-03 | 1.98 | 3.94 | 2.28E-14 | Arhgef5 | Rho guanine nucleotide exchange factor (GEF) 5 |
| ENSMUSG00000034684 | 1.24 | 2.36 | 1.25E-05 | 1.98 | 3.94 | 7.92E-18 | Sema3f | sema domain, immunoglobulin domain (Ig), short basic domain, secreted, (semaphorin) 3F |
| ENSMUSG00000061100 | 1.94 | 3.84 | 8.48E-03 | 1.97 | 3.93 | 1.48E-03 | Retnla | resistin like alpha |
| ENSMUSG00000079507 | 1.32 | 2.49 | 9.93E-04 | 1.97 | 3.93 | 2.99E-09 | H2-Q1 | histocompatibility 2, Q region locus 1 |
| ENSMUSG00000005958 | 1.87 | 3.66 | 1.61E-07 | 1.97 | 3.91 | 2.01E-11 | Ephb3 | Eph receptor B3 |
| ENSMUSG00000006931 | 1.77 | 3.41 | 8.85E-08 | 1.97 | 3.91 | 5.33E-13 | Leprel4 | leprecan-like 4 |
| ENSMUSG00000019467 | 1.09 | 2.14 | 1.15E-04 | 1.96 | 3.90 | 1.79E-17 | Arhgef25 | Rho guanine nucleotide exchange factor (GEF) 25 |
| ENSMUSG00000030598 | 1.43 | 2.70 | 1.92E-05 | 1.97 | 3.90 | 4.59E-14 | Fbxo17 | F-box protein 17 |
| ENSMUSG00000025875 | 1.12 | 2.17 | 1.68E-04 | 1.96 | 3.89 | 2.34E-16 | Tspan17 | tetraspanin 17 |
| ENSMUSG00000027224 | 1.92 | 3.79 | 1.45E-03 | 1.96 | 3.89 | 1.19E-04 | Duoxa1 | dual oxidase maturation factor 1 |
| ENSMUSG00000036206 | 1.07 | 2.10 | 7.57E-05 | 1.96 | 3.89 | 2.32E-19 | Sh3bp4 | SH3-domain binding protein 4 |
| ENSMUSG00000012889 | 1.33 | 2.52 | 5.61E-06 | 1.95 | 3.87 | 4.24E-16 | Podnl1 | podocan-like 1 |
| ENSMUSG00000020388 | 1.27 | 2.41 | 3.03E-05 | 1.95 | 3.85 | 2.03E-15 | Pdlim4 | PDZ and LIM domain 4 |
| ENSMUSG00000052957 | 1.60 | 3.02 | 1.46E-03 | 1.94 | 3.84 | 4.52E-06 | Gas1 | growth arrest specific 1 |
| ENSMUSG00000059430 | 1.10 | 2.14 | 1.16E-03 | 1.93 | 3.82 | 1.01E-13 | Actg2 | actin, gamma 2, smooth muscle, enteric |
| ENSMUSG00000028047 | 1.74 | 3.35 | 2.17E-04 | 1.93 | 3.81 | 1.31E-06 | Thbs3 | thrombospondin 3 |
| ENSMUSG00000038552 | 1.52 | 2.88 | 3.23E-05 | 1.92 | 3.78 | 1.22E-10 | Fndc4 | fibronectin type III domain containing 4 |
| ENSMUSG00000048616 | 1.48 | 2.78 | 5.84E-03 | 1.92 | 3.78 | 7.88E-06 | Nog | noggin |
| ENSMUSG00000080115 | 1.57 | 2.97 | 5.15E-03 | 1.92 | 3.78 | 3.50E-05 | LOC100504608 | protein FAM119B-like |
| ENSMUSG00000000031 | 1.04 | 2.06 | 9.88E-03 | 1.90 | 3.74 | 8.81E-09 | NA | NA |
| ENSMUSG00000027221 | 1.33 | 2.52 | 4.01E-04 | 1.90 | 3.73 | 3.34E-10 | Chst1 | carbohydrate (keratan sulfate Gal-6) sulfotransferase 1 |
| ENSMUSG00000034685 | 1.73 | 3.31 | 9.92E-04 | 1.89 | 3.72 | 1.97E-05 | Fam171a2 | family with sequence similarity 171, member A2 |
| ENSMUSG00000035172 | 1.90 | 3.74 | 4.01E-06 | 1.89 | 3.71 | 4.29E-08 | Plekhh3 | pleckstrin homology domain containing, family H (with MyTH4 domain) member 3 |
| ENSMUSG00000010307 | 1.12 | 2.18 | 2.19E-05 | 1.89 | 3.70 | 1.05E-18 | Tmem86a | transmembrane protein 86A |
| ENSMUSG00000063060 | 1.04 | 2.05 | 3.83E-03 | 1.89 | 3.70 | 1.20E-11 | Sox7 | SRY (sex determining region Y)-box 7 |
| ENSMUSG00000028789 | 1.25 | 2.38 | 1.40E-04 | 1.89 | 3.69 | 7.38E-13 | Adc | arginine decarboxylase |
| ENSMUSG00000020435 | 1.26 | 2.39 | 6.31E-03 | 1.87 | 3.66 | 1.61E-07 | Osbp2 | oxysterol binding protein 2 |
| ENSMUSG00000070436 | 1.01 | 2.01 | 2.09E-05 | 1.86 | 3.64 | 9.45E-22 | Serpinh1 | serine (or cysteine) peptidase inhibitor, clade H, member 1 |
| ENSMUSG00000002266 | 2.12 | 4.36 | 9.01E-03 | 1.86 | 3.63 | 7.88E-03 | Zim1 | zinc finger, imprinted 1 |
| ENSMUSG00000027848 | 1.09 | 2.13 | 9.96E-05 | 1.86 | 3.63 | 7.14E-16 | Olfml3 | olfactomedin-like 3 |
| ENSMUSG00000067653 | 1.23 | 2.34 | 4.66E-03 | 1.86 | 3.63 | 2.47E-07 | Ankrd23 | ankyrin repeat domain 23 |
| ENSMUSG00000049097 | 1.78 | 3.44 | 8.35E-03 | 1.85 | 3.61 | 9.54E-04 | Ankrd34a | ankyrin repeat domain 34A |
| ENSMUSG00000020937 | 1.43 | 2.69 | 2.93E-06 | 1.85 | 3.60 | 5.76E-15 | Plcd3 | phospholipase C, delta 3 |
| ENSMUSG00000074916 | 1.09 | 2.13 | 2.65E-03 | 1.85 | 3.60 | 3.40E-10 | Chst14 | carbohydrate (N-acetylgalactosamine 4-0) sulfotransferase 14 |
| ENSMUSG00000042436 | 1.36 | 2.57 | 4.00E-03 | 1.85 | 3.59 | 2.83E-06 | Mfap4 | microfibrillar-associated protein 4 |
| ENSMUSG00000061848 | 1.36 | 2.57 | 4.42E-04 | 1.85 | 3.59 | 1.12E-08 | NA | NA |
| ENSMUSG00000019558 | 1.03 | 2.04 | 1.63E-03 | 1.84 | 3.58 | 5.94E-12 | Slc6a8 | solute carrier family 6 (neurotransmitter transporter, creatine), member 8 |
| ENSMUSG00000025402 | 1.30 | 2.47 | 1.30E-03 | 1.84 | 3.58 | 3.48E-08 | Nab2 | Ngfi-A binding protein 2 |
| ENSMUSG00000036545 | 1.07 | 2.10 | 8.41E-06 | 1.84 | 3.58 | 8.52E-21 | Adamts2 | a disintegrin-like and metallopeptidase (reprolysin type) with thrombospondin type 1 motif, 2 |
| ENSMUSG00000038128 | 1.31 | 2.48 | 2.20E-03 | 1.84 | 3.58 | 1.31E-08 | Camk4 | calcium/calmodulin-dependent protein kinase IV |
| ENSMUSG00000007207 | 1.24 | 2.36 | 6.09E-05 | 1.84 | 3.57 | 1.14E-13 | Stx1a | syntaxin 1A (brain) |
| ENSMUSG00000070424 | 1.29 | 2.44 | 2.43E-05 | 1.83 | 3.56 | 9.11E-15 | Art5 | ADP-ribosyltransferase 5 |
| ENSMUSG00000039699 | 1.39 | 2.61 | 3.11E-03 | 1.83 | 3.55 | 1.40E-06 | Batf2 | basic leucine zipper transcription factor, ATF-like 2 |
| ENSMUSG00000044134 | 1.54 | 2.91 | 1.62E-07 | 1.83 | 3.55 | 1.87E-14 | Fam109a | family with sequence similarity 109, member A |
| ENSMUSG00000042429 | 1.50 | 2.83 | 6.30E-03 | 1.82 | 3.53 | 2.63E-05 | Adora1 | adenosine A1 receptor |
| ENSMUSG00000047676 | 2.01 | 4.02 | 5.61E-06 | 1.82 | 3.53 | 1.18E-06 | NA | NA |
| ENSMUSG00000036957 | 1.72 | 3.29 | 6.34E-05 | 1.82 | 3.52 | 2.14E-07 | Lrfn3 | leucine rich repeat and fibronectin type III domain containing 3 |
| ENSMUSG00000037962 | 1.99 | 3.96 | 4.02E-03 | 1.82 | 3.52 | 2.38E-03 | Fam101a | family with sequence similarity 101, member A |
| ENSMUSG00000006958 | 1.67 | 3.19 | 6.40E-08 | 1.81 | 3.50 | 8.44E-14 | Chrd | chordin |
| ENSMUSG00000032180 | 1.36 | 2.57 | 1.72E-04 | 1.81 | 3.50 | 1.49E-09 | Tmed1 | transmembrane emp24 domain containing 1 |
| ENSMUSG00000029070 | 1.52 | 2.88 | 4.06E-07 | 1.80 | 3.49 | 4.85E-13 | Mxra8 | matrix-remodelling associated 8 |
| ENSMUSG00000030605 | 1.76 | 3.39 | 1.73E-05 | 1.80 | 3.49 | 1.81E-07 | Mfge8 | milk fat globule-EGF factor 8 protein |
| ENSMUSG00000042804 | 1.03 | 2.05 | 2.25E-03 | 1.80 | 3.49 | 7.11E-11 | Gpr153 | G protein-coupled receptor 153 |
| ENSMUSG00000014303 | 1.34 | 2.53 | 2.05E-04 | 1.80 | 3.48 | 1.59E-09 | Glis2 | GLIS family zinc finger 2 |
| ENSMUSG00000028445 | 1.98 | 3.94 | 3.64E-07 | 1.79 | 3.47 | 2.11E-08 | Enho | energy homeostasis associated |
| ENSMUSG00000031070 | 2.06 | 4.17 | 8.70E-05 | 1.79 | 3.47 | 6.21E-05 | Mrgprf | MAS-related GPR, member F |
| ENSMUSG00000029096 | 1.40 | 2.64 | 3.75E-05 | 1.79 | 3.45 | 2.27E-10 | Htra3 | HtrA serine peptidase 3 |
| ENSMUSG00000027611 | 1.18 | 2.26 | 2.96E-04 | 1.78 | 3.44 | 5.16E-12 | Procr | protein C receptor, endothelial |
| ENSMUSG00000050212 | 1.42 | 2.68 | 4.92E-04 | 1.77 | 3.42 | 1.92E-07 | Eva1b | eva-1 homolog B (C. elegans) |
| ENSMUSG00000056271 | 1.58 | 3.00 | 2.17E-03 | 1.77 | 3.42 | 1.63E-05 | Lman1l | lectin, mannose-binding 1 like |
| ENSMUSG00000034930 | 1.31 | 2.48 | 7.78E-03 | 1.76 | 3.40 | 9.93E-06 | Rtkn | rhotekin |
| ENSMUSG00000046761 | 1.24 | 2.36 | 1.03E-03 | 1.76 | 3.39 | 2.25E-09 | Fam83h | family with sequence similarity 83, member H |
| ENSMUSG00000000901 | 1.27 | 2.41 | 4.32E-05 | 1.76 | 3.38 | 2.27E-12 | Mmp11 | matrix metallopeptidase 11 |
| ENSMUSG00000015085 | 1.21 | 2.31 | 3.69E-03 | 1.74 | 3.35 | 7.40E-08 | Entpd2 | ectonucleoside triphosphate diphosphohydrolase 2 |
| ENSMUSG00000022131 | 1.10 | 2.15 | 1.63E-05 | 1.73 | 3.32 | 4.19E-17 | Gpr180 | G protein-coupled receptor 180 |
| ENSMUSG00000003070 | 1.41 | 2.66 | 3.49E-03 | 1.72 | 3.31 | 8.72E-06 | Efna2 | ephrin A2 |
| ENSMUSG00000001020 | 1.12 | 2.17 | 1.57E-05 | 1.72 | 3.30 | 4.55E-16 | S100a4 | S100 calcium binding protein A4 |
| ENSMUSG00000001348 | 1.44 | 2.72 | 4.02E-06 | 1.72 | 3.30 | 3.44E-11 | Acp5 | acid phosphatase 5, tartrate resistant |
| ENSMUSG00000049556 | 2.53 | 5.76 | 9.61E-07 | 1.71 | 3.28 | 1.04E-04 | Lingo1 | leucine rich repeat and Ig domain containing 1 |
| ENSMUSG00000050248 | 1.20 | 2.29 | 9.56E-06 | 1.71 | 3.28 | 2.05E-15 | Evc2 | Ellis van Creveld syndrome 2 |
| ENSMUSG00000060572 | 1.39 | 2.63 | 4.31E-06 | 1.71 | 3.28 | 6.07E-12 | Mfap2 | microfibrillar-associated protein 2 |
| ENSMUSG00000051790 | 1.01 | 2.01 | 5.17E-03 | 1.71 | 3.27 | 8.56E-09 | Nlgn2 | neuroligin 2 |
| ENSMUSG00000033722 | 1.42 | 2.67 | 4.85E-06 | 1.70 | 3.25 | 1.21E-11 | BC034090 | cDNA sequence BC034090 |
| ENSMUSG00000037735 | 1.95 | 3.86 | 7.91E-03 | 1.70 | 3.25 | 5.68E-03 | NA | NA |
| ENSMUSG00000050910 | 1.24 | 2.36 | 3.07E-07 | 1.70 | 3.25 | 4.56E-18 | Cdr2l | cerebellar degeneration-related protein 2-like |
| ENSMUSG00000022199 | 1.27 | 2.42 | 5.21E-04 | 1.69 | 3.24 | 2.28E-08 | Slc22a17 | solute carrier family 22 (organic cation transporter), member 17 |
| ENSMUSG00000032649 | 1.60 | 3.03 | 3.41E-03 | 1.69 | 3.23 | 2.74E-04 | Colgalt2 | collagen beta(1-O)galactosyltransferase 2 |
| ENSMUSG00000025511 | 1.69 | 3.22 | 8.93E-08 | 1.68 | 3.21 | 1.17E-10 | Tspan4 | tetraspanin 4 |
| ENSMUSG00000049580 | 1.43 | 2.69 | 3.48E-05 | 1.68 | 3.21 | 4.18E-09 | Tsku | tsukushi |
| ENSMUSG00000051703 | 1.23 | 2.34 | 5.02E-03 | 1.68 | 3.21 | 4.06E-07 | Tmem198 | transmembrane protein 198 |
| ENSMUSG00000007594 | 1.84 | 3.59 | 3.85E-04 | 1.67 | 3.19 | 1.07E-04 | Hapln4 | hyaluronan and proteoglycan link protein 4 |
| ENSMUSG00000025140 | 1.40 | 2.64 | 1.65E-05 | 1.67 | 3.18 | 3.20E-10 | Pycr1 | pyrroline-5-carboxylate reductase 1 |
| ENSMUSG00000037347 | 1.63 | 3.10 | 2.19E-04 | 1.67 | 3.18 | 5.91E-06 | Chst7 | carbohydrate (N-acetylglucosamino) sulfotransferase 7 |
| ENSMUSG00000037686 | 1.97 | 3.93 | 6.42E-10 | 1.66 | 3.17 | 6.26E-11 | Aspg | asparaginase homolog (S. cerevisiae) |
| ENSMUSG00000032363 | 1.09 | 2.12 | 7.29E-04 | 1.66 | 3.16 | 2.30E-10 | Adamts7 | a disintegrin-like and metallopeptidase (reprolysin type) with thrombospondin type 1 motif, 7 |
| ENSMUSG00000020902 | 1.66 | 3.17 | 2.39E-05 | 1.65 | 3.15 | 5.29E-07 | Ntn1 | netrin 1 |
| ENSMUSG00000048779 | 1.40 | 2.64 | 1.93E-04 | 1.65 | 3.15 | 1.19E-07 | P2ry6 | pyrimidinergic receptor P2Y, G-protein coupled, 6 |
| ENSMUSG00000023266 | 1.47 | 2.78 | 9.16E-04 | 1.64 | 3.13 | 3.97E-06 | Frs3 | fibroblast growth factor receptor substrate 3 |
| ENSMUSG00000073674 | 1.26 | 2.40 | 4.53E-04 | 1.65 | 3.13 | 2.45E-08 | NA | NA |
| ENSMUSG00000045045 | 1.43 | 2.69 | 2.90E-04 | 1.64 | 3.12 | 5.55E-07 | Lrfn4 | leucine rich repeat and fibronectin type III domain containing 4 |
| ENSMUSG00000001657 | 1.80 | 3.48 | 1.66E-03 | 1.63 | 3.09 | 5.76E-04 | Hoxc8 | homeobox C8 |
| ENSMUSG00000020086 | 1.21 | 2.31 | 9.46E-04 | 1.63 | 3.09 | 1.67E-08 | H2afy2 | H2A histone family, member Y2 |
| ENSMUSG00000022525 | 1.05 | 2.06 | 9.66E-03 | 1.63 | 3.09 | 3.52E-07 | Hrasls | HRAS-like suppressor |
| ENSMUSG00000078311 | 1.87 | 3.65 | 8.52E-05 | 1.63 | 3.09 | 5.92E-05 | NA | NA |
| ENSMUSG00000002308 | 1.23 | 2.35 | 6.97E-05 | 1.62 | 3.08 | 1.42E-11 | Cd320 | CD320 antigen |
| ENSMUSG00000020282 | 1.13 | 2.19 | 7.38E-05 | 1.62 | 3.08 | 4.95E-12 | Rhbdf1 | rhomboid family 1 (Drosophila) |
| ENSMUSG00000029769 | 1.36 | 2.56 | 2.07E-03 | 1.62 | 3.08 | 5.42E-06 | Ccdc136 | coiled-coil domain containing 136 |
| ENSMUSG00000080002 | 1.26 | 2.39 | 5.29E-06 | 1.62 | 3.08 | 7.91E-13 | NA | NA |
| ENSMUSG00000067924 | 1.17 | 2.25 | 7.84E-06 | 1.60 | 3.04 | 8.11E-15 | NA | NA |
| ENSMUSG00000070371 | 1.19 | 2.27 | 3.88E-04 | 1.60 | 3.03 | 2.46E-10 | Prss36 | protease, serine 36 |
| ENSMUSG00000003355 | 1.49 | 2.82 | 1.77E-04 | 1.60 | 3.02 | 1.62E-06 | Fkbp11 | FK506 binding protein 11 |
| ENSMUSG00000031169 | 1.05 | 2.07 | 8.91E-04 | 1.59 | 3.02 | 4.94E-10 | Porcn | porcupine homolog (Drosophila) |
| ENSMUSG00000073418 | 1.13 | 2.19 | 7.94E-05 | 1.60 | 3.02 | 1.83E-11 | C4b | complement component 4B (Chido blood group) |
| ENSMUSG00000015094 | 1.68 | 3.21 | 1.12E-09 | 1.59 | 3.01 | 2.31E-12 | Npdc1 | neural proliferation, differentiation and control 1 |
| ENSMUSG00000002228 | 1.39 | 2.62 | 3.13E-05 | 1.59 | 3.00 | 6.09E-09 | Ppm1j | protein phosphatase 1J |
| ENSMUSG00000043068 | 1.83 | 3.57 | 2.11E-03 | 1.59 | 3.00 | 1.32E-03 | Fam89a | family with sequence similarity 89, member A |
| ENSMUSG00000020810 | 1.49 | 2.81 | 8.20E-04 | 1.57 | 2.98 | 3.11E-05 | Cygb | cytoglobin |
| ENSMUSG00000024736 | 1.42 | 2.68 | 2.12E-05 | 1.58 | 2.98 | 1.12E-08 | Tmem132a | transmembrane protein 132A |
| ENSMUSG00000026796 | 1.15 | 2.22 | 1.04E-03 | 1.58 | 2.98 | 7.11E-08 | Fam129b | family with sequence similarity 129, member B |
| ENSMUSG00000036862 | 1.06 | 2.09 | 6.75E-04 | 1.58 | 2.98 | 7.69E-10 | Dchs1 | dachsous 1 (Drosophila) |
| ENSMUSG00000024909 | 1.47 | 2.78 | 2.16E-06 | 1.57 | 2.97 | 1.31E-09 | Efemp2 | epidermal growth factor-containing fibulin-like extracellular matrix protein 2 |
| ENSMUSG00000048807 | 1.31 | 2.48 | 4.52E-05 | 1.57 | 2.97 | 2.72E-09 | Slc35e4 | solute carrier family 35, member E4 |
| ENSMUSG00000028885 | 1.12 | 2.17 | 1.55E-03 | 1.56 | 2.96 | 1.59E-08 | Smpdl3b | sphingomyelin phosphodiesterase, acid-like 3B |
| ENSMUSG00000031790 | 1.45 | 2.74 | 2.18E-04 | 1.56 | 2.96 | 1.92E-06 | Mmp15 | matrix metallopeptidase 15 |
| ENSMUSG00000045930 | 1.26 | 2.40 | 1.25E-04 | 1.57 | 2.96 | 6.46E-09 | Clec14a | C-type lectin domain family 14, member a |
| ENSMUSG00000040605 | 1.04 | 2.06 | 1.55E-03 | 1.56 | 2.95 | 1.50E-09 | Bace2 | beta-site APP-cleaving enzyme 2 |
| ENSMUSG00000020312 | 1.43 | 2.69 | 2.71E-07 | 1.55 | 2.94 | 6.64E-12 | Shc2 | SHC (Src homology 2 domain containing) transforming protein 2 |
| ENSMUSG00000073556 | 1.17 | 2.26 | 1.36E-04 | 1.55 | 2.93 | 1.76E-11 | NA | NA |
| ENSMUSG00000038600 | 1.62 | 3.07 | 8.55E-03 | 1.55 | 2.92 | 2.48E-03 | Atp6v0a4 | ATPase, H+ transporting, lysosomal V0 subunit A4 |
| ENSMUSG00000073889 | 1.33 | 2.51 | 4.09E-06 | 1.54 | 2.91 | 5.95E-11 | Il11ra1 | interleukin 11 receptor, alpha chain 1 |
| ENSMUSG00000026223 | 1.20 | 2.30 | 3.41E-05 | 1.53 | 2.89 | 2.33E-10 | Itm2c | integral membrane protein 2C |
| ENSMUSG00000033256 | 1.64 | 3.13 | 4.50E-10 | 1.53 | 2.89 | 2.83E-13 | Shf | Src homology 2 domain containing F |
| ENSMUSG00000028641 | 1.18 | 2.27 | 1.60E-05 | 1.52 | 2.87 | 1.72E-11 | Lepre1 | leprecan 1 |
| ENSMUSG00000031851 | 1.09 | 2.12 | 1.35E-05 | 1.52 | 2.87 | 4.26E-14 | Ntpcr | nucleoside-triphosphatase, cancer-related |
| ENSMUSG00000048782 | 1.46 | 2.74 | 1.69E-04 | 1.52 | 2.86 | 2.53E-06 | Insc | inscuteable homolog (Drosophila) |
| ENSMUSG00000049521 | 1.35 | 2.54 | 3.01E-05 | 1.51 | 2.86 | 1.70E-08 | Cdc42ep1 | CDC42 effector protein (Rho GTPase binding) 1 |
| ENSMUSG00000056590 | 1.19 | 2.29 | 5.71E-03 | 1.51 | 2.85 | 4.42E-06 | NA | NA |
| ENSMUSG00000007783 | 1.67 | 3.19 | 1.94E-09 | 1.51 | 2.84 | 2.43E-11 | Cpt1c | carnitine palmitoyltransferase 1c |
| ENSMUSG00000022947 | 1.23 | 2.35 | 6.09E-05 | 1.50 | 2.83 | 1.04E-09 | Cbr3 | carbonyl reductase 3 |
| ENSMUSG00000000094 | 1.35 | 2.55 | 7.73E-04 | 1.49 | 2.81 | 4.97E-06 | Tbx4 | T-box 4 |
| ENSMUSG00000032431 | 1.12 | 2.18 | 3.16E-05 | 1.49 | 2.81 | 2.57E-11 | Crtap | cartilage associated protein |
| ENSMUSG00000030351 | 1.65 | 3.14 | 2.07E-08 | 1.48 | 2.79 | 2.69E-10 | Tspan11 | tetraspanin 11 |
| ENSMUSG00000030796 | 1.37 | 2.59 | 1.50E-06 | 1.48 | 2.79 | 2.35E-10 | Tead2 | TEA domain family member 2 |
| ENSMUSG00000051043 | 1.29 | 2.44 | 9.19E-05 | 1.48 | 2.78 | 6.11E-08 | Gprc5c | G protein-coupled receptor, family C, group 5, member C |
| ENSMUSG00000003534 | 1.39 | 2.61 | 1.75E-04 | 1.47 | 2.77 | 1.86E-06 | Ddr1 | discoidin domain receptor family, member 1 |
| ENSMUSG00000040488 | 1.47 | 2.78 | 2.50E-04 | 1.47 | 2.77 | 1.80E-05 | Ltbp4 | latent transforming growth factor beta binding protein 4 |
| ENSMUSG00000003380 | 1.52 | 2.87 | 4.69E-06 | 1.47 | 2.76 | 1.44E-07 | Rabac1 | Rab acceptor 1 (prenylated) |
| ENSMUSG00000025484 | 1.05 | 2.08 | 1.07E-03 | 1.46 | 2.75 | 2.47E-08 | Bet1l | blocked early in transport 1 homolog (S. cerevisiae)-like |
| ENSMUSG00000069920 | 1.13 | 2.19 | 5.12E-04 | 1.46 | 2.74 | 3.43E-08 | B3gnt9 | UDP-GlcNAc:betaGal beta-1,3-N-acetylglucosaminyltransferase 9 |
| ENSMUSG00000073368 | 1.46 | 2.75 | 2.00E-03 | 1.45 | 2.74 | 2.00E-04 | NA | NA |
| ENSMUSG00000035314 | 1.34 | 2.53 | 1.25E-06 | 1.45 | 2.73 | 6.14E-11 | Gdpd5 | glycerophosphodiester phosphodiesterase domain containing 5 |
| ENSMUSG00000021464 | 1.12 | 2.17 | 4.83E-03 | 1.44 | 2.72 | 9.21E-06 | Ror2 | receptor tyrosine kinase-like orphan receptor 2 |
| ENSMUSG00000036904 | 1.95 | 3.85 | 2.03E-04 | 1.44 | 2.72 | 1.51E-03 | Fzd8 | frizzled homolog 8 (Drosophila) |
| ENSMUSG00000026890 | 1.11 | 2.16 | 9.51E-03 | 1.43 | 2.70 | 2.51E-05 | Lhx6 | LIM homeobox protein 6 |
| ENSMUSG00000027932 | 1.50 | 2.82 | 6.72E-06 | 1.43 | 2.70 | 1.38E-07 | Slc27a3 | solute carrier family 27 (fatty acid transporter), member 3 |
| ENSMUSG00000022203 | 2.01 | 4.02 | 1.14E-13 | 1.43 | 2.69 | 2.24E-10 | Efs | embryonal Fyn-associated substrate |
| ENSMUSG00000035041 | 1.14 | 2.21 | 7.78E-03 | 1.42 | 2.69 | 3.79E-05 | Creb3l3 | cAMP responsive element binding protein 3-like 3 |
| ENSMUSG00000022548 | 1.36 | 2.57 | 3.16E-03 | 1.42 | 2.67 | 3.20E-04 | Apod | apolipoprotein D |
| ENSMUSG00000053646 | 1.41 | 2.65 | 3.42E-05 | 1.42 | 2.67 | 5.91E-07 | Plxnb1 | plexin B1 |
| ENSMUSG00000075389 | 1.07 | 2.09 | 5.61E-03 | 1.42 | 2.67 | 1.37E-06 | NA | NA |
| ENSMUSG00000024990 | 1.42 | 2.68 | 2.76E-04 | 1.41 | 2.66 | 1.95E-05 | Rbp4 | retinol binding protein 4, plasma |
| ENSMUSG00000038260 | 1.16 | 2.24 | 4.66E-05 | 1.41 | 2.66 | 1.50E-09 | Trpm4 | transient receptor potential cation channel, subfamily M, member 4 |
| ENSMUSG00000006378 | 1.38 | 2.61 | 1.80E-03 | 1.40 | 2.65 | 1.04E-04 | NA | NA |
| ENSMUSG00000023992 | 1.19 | 2.28 | 9.74E-03 | 1.41 | 2.65 | 2.64E-04 | Trem2 | triggering receptor expressed on myeloid cells 2 |
| ENSMUSG00000026785 | 1.05 | 2.07 | 2.04E-06 | 1.41 | 2.65 | 1.46E-15 | Pkn3 | protein kinase N3 |
| ENSMUSG00000027954 | 1.21 | 2.31 | 2.79E-04 | 1.41 | 2.65 | 2.91E-07 | Efna1 | ephrin A1 |
| ENSMUSG00000031841 | 1.36 | 2.56 | 3.51E-06 | 1.41 | 2.65 | 7.26E-09 | Cdh13 | cadherin 13 |
| ENSMUSG00000006435 | 1.33 | 2.52 | 5.66E-04 | 1.40 | 2.64 | 1.65E-05 | Neurl1a | neuralized homolog 1A (Drosophila) |
| ENSMUSG00000033327 | 1.95 | 3.87 | 1.05E-04 | 1.40 | 2.64 | 1.45E-03 | Tnxb | tenascin XB |
| ENSMUSG00000060284 | 1.61 | 3.05 | 5.64E-06 | 1.40 | 2.64 | 2.71E-06 | Sp7 | Sp7 transcription factor 7 |
| ENSMUSG00000038765 | 1.33 | 2.52 | 6.99E-03 | 1.40 | 2.63 | 5.17E-04 | Lmx1b | LIM homeobox transcription factor 1 beta |
| ENSMUSG00000066705 | 1.02 | 2.03 | 8.83E-04 | 1.40 | 2.63 | 2.96E-08 | Fxyd6 | FXYD domain-containing ion transport regulator 6 |
| ENSMUSG00000024346 | 1.20 | 2.29 | 8.44E-05 | 1.39 | 2.62 | 3.76E-08 | Pfdn1 | prefoldin 1 |
| ENSMUSG00000040563 | 1.34 | 2.53 | 8.84E-04 | 1.39 | 2.62 | 3.18E-05 | BC018242 | cDNA sequence BC018242 |
| ENSMUSG00000086409 | 1.84 | 3.59 | 2.83E-05 | 1.39 | 2.62 | 1.77E-04 | NA | NA |
| ENSMUSG00000090210 | 1.31 | 2.47 | 7.74E-08 | 1.39 | 2.62 | 2.24E-12 | Itga10 | integrin, alpha 10 |
| ENSMUSG00000037254 | 1.68 | 3.21 | 3.55E-03 | 1.38 | 2.61 | 4.62E-03 | Itih2 | inter-alpha trypsin inhibitor, heavy chain 2 |
| ENSMUSG00000031958 | 1.05 | 2.06 | 2.63E-03 | 1.38 | 2.60 | 9.76E-07 | Ldhd | lactate dehydrogenase D |
| ENSMUSG00000037977 | 1.52 | 2.87 | 2.56E-03 | 1.38 | 2.60 | 1.35E-03 | 6430571L13Rik | RIKEN cDNA 6430571L13 gene |
| ENSMUSG00000054474 | 1.03 | 2.04 | 4.77E-03 | 1.38 | 2.60 | 1.36E-06 | Thnsl2 | threonine synthase-like 2 (bacterial) |
| ENSMUSG00000021763 | 1.59 | 3.01 | 3.85E-03 | 1.37 | 2.59 | 2.88E-03 | BC067074 | cDNA sequence BC067074 |
| ENSMUSG00000032968 | 1.78 | 3.44 | 2.08E-04 | 1.38 | 2.59 | 8.23E-04 | Inha | inhibin alpha |
| ENSMUSG00000041120 | 1.61 | 3.06 | 4.83E-05 | 1.37 | 2.59 | 5.09E-05 | Nbl1 | neuroblastoma, suppression of tumorigenicity 1 |
| ENSMUSG00000000753 | 1.40 | 2.64 | 1.31E-05 | 1.37 | 2.58 | 4.17E-07 | Serpinf1 | serine (or cysteine) peptidase inhibitor, clade F, member 1 |
| ENSMUSG00000003352 | 1.76 | 3.38 | 3.90E-08 | 1.36 | 2.58 | 3.11E-07 | Cacnb3 | calcium channel, voltage-dependent, beta 3 subunit |
| ENSMUSG00000037032 | 1.63 | 3.10 | 3.51E-08 | 1.37 | 2.58 | 2.15E-08 | Apbb1 | amyloid beta (A4) precursor protein-binding, family B, member 1 |
| ENSMUSG00000039461 | 1.05 | 2.07 | 2.16E-04 | 1.37 | 2.58 | 3.75E-09 | Tcta | T cell leukemia translocation altered gene |
| ENSMUSG00000026697 | 2.30 | 4.92 | 1.25E-06 | 1.36 | 2.57 | 9.45E-04 | Myoc | myocilin |
| ENSMUSG00000066607 | 1.35 | 2.55 | 1.01E-05 | 1.36 | 2.57 | 4.12E-08 | 6030419C18Rik | RIKEN cDNA 6030419C18 gene |
| ENSMUSG00000067925 | 1.09 | 2.13 | 5.28E-04 | 1.36 | 2.57 | 8.53E-08 | NA | NA |
| ENSMUSG00000004105 | 1.12 | 2.17 | 1.29E-03 | 1.36 | 2.56 | 3.29E-06 | Angptl2 | angiopoietin-like 2 |
| ENSMUSG00000039646 | 1.09 | 2.13 | 1.63E-04 | 1.36 | 2.56 | 1.91E-08 | Vasn | vasorin |
| ENSMUSG00000074796 | 1.19 | 2.28 | 9.78E-03 | 1.35 | 2.56 | 2.10E-04 | Slc4a11 | solute carrier family 4, sodium bicarbonate transporter-like, member 11 |
| ENSMUSG00000021506 | 1.39 | 2.62 | 2.89E-04 | 1.35 | 2.55 | 2.53E-05 | Pitx1 | paired-like homeodomain transcription factor 1 |
| ENSMUSG00000038319 | 1.51 | 2.85 | 2.45E-04 | 1.35 | 2.55 | 7.76E-05 | Kcnh2 | potassium voltage-gated channel, subfamily H (eag-related), member 2 |
| ENSMUSG00000025738 | 1.42 | 2.68 | 1.12E-05 | 1.34 | 2.53 | 2.45E-07 | Fbxl16 | F-box and leucine-rich repeat protein 16 |
| ENSMUSG00000025150 | 1.76 | 3.39 | 9.26E-08 | 1.32 | 2.50 | 1.74E-06 | Cbr2 | carbonyl reductase 2 |
| ENSMUSG00000029161 | 1.34 | 2.53 | 7.68E-04 | 1.32 | 2.50 | 9.22E-05 | Cgref1 | cell growth regulator with EF hand domain 1 |
| ENSMUSG00000039908 | 1.25 | 2.38 | 5.00E-06 | 1.32 | 2.50 | 1.32E-09 | Slc26a11 | solute carrier family 26, member 11 |
| ENSMUSG00000041420 | 1.26 | 2.40 | 3.18E-05 | 1.32 | 2.50 | 1.23E-07 | Meis3 | Meis homeobox 3 |
| ENSMUSG00000042821 | 1.37 | 2.58 | 7.61E-05 | 1.32 | 2.50 | 5.48E-06 | Snai1 | snail homolog 1 (Drosophila) |
| ENSMUSG00000017737 | 1.01 | 2.02 | 2.58E-07 | 1.31 | 2.48 | 5.28E-16 | Mmp9 | matrix metallopeptidase 9 |
| ENSMUSG00000020773 | 1.40 | 2.64 | 1.96E-06 | 1.31 | 2.48 | 8.91E-08 | Trim47 | tripartite motif-containing 47 |
| ENSMUSG00000050390 | 1.00 | 2.00 | 7.04E-03 | 1.31 | 2.47 | 2.37E-05 | C77080 | expressed sequence C77080 |
| ENSMUSG00000014776 | 1.04 | 2.06 | 1.06E-03 | 1.30 | 2.46 | 6.01E-07 | Nol3 | nucleolar protein 3 (apoptosis repressor with CARD domain) |
| ENSMUSG00000016995 | 1.78 | 3.43 | 6.41E-04 | 1.30 | 2.46 | 4.40E-03 | Matn4 | matrilin 4 |
| ENSMUSG00000034412 | 1.13 | 2.19 | 1.16E-05 | 1.30 | 2.46 | 7.14E-10 | Tbc1d10a | TBC1 domain family, member 10a |
| ENSMUSG00000043501 | 2.27 | 4.82 | 3.61E-05 | 1.30 | 2.46 | 6.93E-03 | Lgals2 | lectin, galactose-binding, soluble 2 |
| ENSMUSG00000015312 | 1.05 | 2.07 | 2.46E-03 | 1.28 | 2.43 | 7.37E-06 | Gadd45b | growth arrest and DNA-damage-inducible 45 beta |
| ENSMUSG00000034463 | 1.05 | 2.06 | 3.07E-04 | 1.28 | 2.43 | 1.15E-07 | Scara3 | scavenger receptor class A, member 3 |
| ENSMUSG00000074861 | 1.41 | 2.66 | 1.79E-03 | 1.28 | 2.42 | 3.64E-04 | NA | NA |
| ENSMUSG00000030804 | 1.03 | 2.05 | 3.31E-04 | 1.27 | 2.41 | 9.94E-08 | NA | NA |
| ENSMUSG00000038086 | 1.27 | 2.41 | 7.75E-04 | 1.27 | 2.41 | 5.76E-05 | Hspb2 | heat shock protein 2 |
| ENSMUSG00000047945 | 1.05 | 2.07 | 9.88E-05 | 1.27 | 2.41 | 1.35E-08 | Marcksl1 | MARCKS-like 1 |
| ENSMUSG00000008206 | 1.14 | 2.21 | 5.02E-05 | 1.26 | 2.40 | 1.75E-08 | Cers4 | ceramide synthase 4 |
| ENSMUSG00000002393 | 1.24 | 2.36 | 5.22E-03 | 1.26 | 2.39 | 7.74E-04 | Nr2f6 | nuclear receptor subfamily 2, group F, member 6 |
| ENSMUSG00000050105 | 1.40 | 2.63 | 1.16E-08 | 1.26 | 2.39 | 9.21E-11 | Grrp1 | glycine/arginine rich protein 1 |
| ENSMUSG00000070577 | 1.24 | 2.36 | 9.85E-03 | 1.25 | 2.38 | 1.53E-03 | Gm572 | predicted gene 572 |
| ENSMUSG00000002105 | 1.19 | 2.27 | 2.08E-08 | 1.25 | 2.37 | 4.98E-13 | Slc39a13 | solute carrier family 39 (metal ion transporter), member 13 |
| ENSMUSG00000018012 | 1.51 | 2.85 | 1.63E-05 | 1.25 | 2.37 | 1.48E-05 | Rac3 | RAS-related C3 botulinum substrate 3 |
| ENSMUSG00000032606 | 1.07 | 2.10 | 1.16E-03 | 1.24 | 2.37 | 3.41E-06 | Nicn1 | nicolin 1 |
| ENSMUSG00000024793 | 1.41 | 2.65 | 4.99E-03 | 1.24 | 2.36 | 3.61E-03 | Tnfrsf25 | tumor necrosis factor receptor superfamily, member 25 |
| ENSMUSG00000009035 | 1.05 | 2.08 | 2.36E-04 | 1.23 | 2.35 | 2.77E-07 | Tmem184b | transmembrane protein 184b |
| ENSMUSG00000022215 | 1.29 | 2.45 | 2.41E-03 | 1.22 | 2.33 | 7.38E-04 | Fitm1 | fat storage-inducing transmembrane protein 1 |
| ENSMUSG00000025978 | 1.31 | 2.49 | 1.69E-05 | 1.22 | 2.33 | 1.70E-06 | Rftn2 | raftlin family member 2 |
| ENSMUSG00000029312 | 1.00 | 2.00 | 4.25E-04 | 1.22 | 2.32 | 9.74E-08 | Klhl8 | kelch-like 8 |
| ENSMUSG00000000154 | 1.62 | 3.08 | 2.21E-04 | 1.21 | 2.31 | 7.35E-04 | Slc22a18 | solute carrier family 22 (organic cation transporter), member 18 |
| ENSMUSG00000031808 | 1.11 | 2.16 | 2.32E-05 | 1.21 | 2.31 | 2.85E-08 | Slc27a1 | solute carrier family 27 (fatty acid transporter), member 1 |
| ENSMUSG00000068396 | 1.50 | 2.84 | 4.21E-04 | 1.20 | 2.30 | 1.12E-03 | NA | NA |
| ENSMUSG00000024173 | 2.64 | 6.25 | 2.82E-07 | 1.19 | 2.29 | 9.47E-03 | Tpsab1 | tryptase alpha/beta 1 |
| ENSMUSG00000034659 | 1.11 | 2.15 | 1.43E-04 | 1.19 | 2.29 | 1.00E-06 | Tmem109 | transmembrane protein 109 |
| ENSMUSG00000039759 | 1.00 | 2.00 | 1.43E-04 | 1.20 | 2.29 | 1.75E-08 | Thap3 | THAP domain containing, apoptosis associated protein 3 |
| ENSMUSG00000074170 | 1.03 | 2.04 | 2.93E-07 | 1.20 | 2.29 | 5.13E-14 | Plekhf1 | pleckstrin homology domain containing, family F (with FYVE domain) member 1 |
| ENSMUSG00000002985 | 1.08 | 2.11 | 7.30E-04 | 1.19 | 2.28 | 1.01E-05 | Apoe | apolipoprotein E |
| ENSMUSG00000046892 | 1.63 | 3.09 | 4.56E-04 | 1.19 | 2.28 | 2.99E-03 | NA | NA |
| ENSMUSG00000054793 | 1.21 | 2.32 | 6.80E-03 | 1.19 | 2.28 | 1.00E-03 | Cadm4 | cell adhesion molecule 4 |
| ENSMUSG00000082076 | 1.42 | 2.67 | 5.31E-04 | 1.19 | 2.28 | 5.90E-04 | NA | NA |
| ENSMUSG00000014609 | 1.41 | 2.66 | 2.91E-03 | 1.18 | 2.27 | 2.83E-03 | Chrne | cholinergic receptor, nicotinic, epsilon polypeptide |
| ENSMUSG00000027313 | 1.44 | 2.71 | 1.22E-03 | 1.18 | 2.27 | 2.06E-03 | Chac1 | ChaC, cation transport regulator 1 |
| ENSMUSG00000057777 | 1.29 | 2.45 | 1.39E-03 | 1.19 | 2.27 | 4.17E-04 | Mab21l2 | mab-21-like 2 (C. elegans) |
| ENSMUSG00000025185 | 1.51 | 2.85 | 1.37E-04 | 1.18 | 2.26 | 4.98E-04 | Loxl4 | lysyl oxidase-like 4 |
| ENSMUSG00000042766 | 1.52 | 2.87 | 3.22E-05 | 1.17 | 2.26 | 1.44E-04 | Trim46 | tripartite motif-containing 46 |
| ENSMUSG00000048752 | 1.54 | 2.91 | 1.85E-04 | 1.18 | 2.26 | 6.41E-04 | Prss50 | protease, serine 50 |
| ENSMUSG00000061451 | 1.56 | 2.94 | 1.51E-03 | 1.17 | 2.24 | 4.97E-03 | Tmem151a | transmembrane protein 151A |
| ENSMUSG00000085939 | 1.10 | 2.14 | 4.72E-05 | 1.16 | 2.24 | 2.99E-07 | NA | NA |
| ENSMUSG00000001270 | 1.63 | 3.09 | 1.12E-05 | 1.16 | 2.23 | 2.69E-04 | Ckb | creatine kinase, brain |
| ENSMUSG00000028601 | 1.18 | 2.26 | 1.54E-04 | 1.16 | 2.23 | 5.52E-06 | Echdc2 | enoyl Coenzyme A hydratase domain containing 2 |
| ENSMUSG00000002504 | 1.02 | 2.03 | 2.47E-05 | 1.15 | 2.22 | 7.47E-09 | Slc9a3r2 | solute carrier family 9 (sodium/hydrogen exchanger), member 3 regulator 2 |
| ENSMUSG00000048537 | 1.17 | 2.25 | 4.61E-05 | 1.15 | 2.22 | 1.66E-06 | Phldb1 | pleckstrin homology-like domain, family B, member 1 |
| ENSMUSG00000075702 | 1.33 | 2.51 | 4.60E-07 | 1.15 | 2.22 | 1.60E-07 | Selm | selenoprotein M |
| ENSMUSG00000019659 | 1.08 | 2.12 | 1.74E-03 | 1.15 | 2.21 | 8.36E-05 | Ccdc12 | coiled-coil domain containing 12 |
| ENSMUSG00000030170 | 1.35 | 2.54 | 1.08E-05 | 1.14 | 2.21 | 6.51E-06 | Wnt5b | wingless-related MMTV integration site 5B |
| ENSMUSG00000034863 | 1.02 | 2.03 | 2.60E-03 | 1.14 | 2.21 | 5.38E-05 | Ano8 | anoctamin 8 |
| ENSMUSG00000038777 | 1.63 | 3.10 | 4.88E-04 | 1.14 | 2.21 | 5.25E-03 | Sema6c | sema domain, transmembrane domain (TM), and cytoplasmic domain, (semaphorin) 6C |
| ENSMUSG00000006205 | 1.21 | 2.32 | 5.83E-05 | 1.14 | 2.20 | 8.55E-06 | Htra1 | HtrA serine peptidase 1 |
| ENSMUSG00000030122 | 1.11 | 2.15 | 4.45E-04 | 1.13 | 2.19 | 2.31E-05 | Ptms | parathymosin |
| ENSMUSG00000050234 | 1.17 | 2.25 | 2.61E-04 | 1.13 | 2.19 | 1.51E-05 | Gja4 | gap junction protein, alpha 4 |
| ENSMUSG00000058806 | 1.26 | 2.39 | 3.99E-03 | 1.13 | 2.19 | 2.47E-03 | Col13a1 | collagen, type XIII, alpha 1 |
| ENSMUSG00000020829 | 1.78 | 3.44 | 1.92E-06 | 1.13 | 2.18 | 4.11E-04 | Slc46a1 | solute carrier family 46, member 1 |
| ENSMUSG00000025407 | 1.79 | 3.46 | 2.45E-05 | 1.13 | 2.18 | 2.19E-03 | Gli1 | GLI-Kruppel family member GLI1 |
| ENSMUSG00000028763 | 1.11 | 2.16 | 9.46E-04 | 1.13 | 2.18 | 7.56E-05 | Hspg2 | perlecan (heparan sulfate proteoglycan 2) |
| ENSMUSG00000029641 | 1.44 | 2.71 | 1.26E-03 | 1.12 | 2.18 | 2.70E-03 | Rasl11a | RAS-like, family 11, member A |
| ENSMUSG00000046470 | 1.60 | 3.03 | 2.64E-05 | 1.12 | 2.18 | 6.02E-04 | Sox18 | SRY (sex determining region Y)-box 18 |
| ENSMUSG00000060019 | 1.51 | 2.85 | 1.04E-04 | 1.13 | 2.18 | 7.88E-04 | NA | NA |
| ENSMUSG00000024247 | 1.75 | 3.35 | 2.06E-06 | 1.11 | 2.17 | 4.13E-04 | Pkdcc | protein kinase domain containing, cytoplasmic |
| ENSMUSG00000026211 | 1.21 | 2.31 | 1.50E-04 | 1.12 | 2.17 | 3.35E-05 | Obsl1 | obscurin-like 1 |
| ENSMUSG00000029093 | 1.03 | 2.04 | 2.81E-04 | 1.10 | 2.15 | 2.00E-06 | Sorcs2 | sortilin-related VPS10 domain containing receptor 2 |
| ENSMUSG00000008167 | 1.35 | 2.55 | 6.84E-07 | 1.09 | 2.14 | 8.85E-07 | Fbxw9 | F-box and WD-40 domain protein 9 |
| ENSMUSG00000023259 | 1.13 | 2.19 | 3.84E-04 | 1.10 | 2.14 | 2.26E-05 | NA | NA |
| ENSMUSG00000025466 | 1.05 | 2.07 | 9.93E-04 | 1.10 | 2.14 | 2.21E-05 | Fuom | fucose mutarotase |
| ENSMUSG00000025610 | 1.14 | 2.21 | 8.41E-03 | 1.10 | 2.14 | 2.59E-03 | Map3k7cl | Map3k7 C-terminal like |
| ENSMUSG00000032523 | 1.44 | 2.70 | 4.82E-04 | 1.10 | 2.14 | 2.02E-03 | Hhatl | hedgehog acyltransferase-like |
| ENSMUSG00000019539 | 1.54 | 2.90 | 1.15E-08 | 1.09 | 2.13 | 1.34E-06 | Rcn3 | reticulocalbin 3, EF-hand calcium binding domain |
| ENSMUSG00000055912 | 1.52 | 2.87 | 2.17E-05 | 1.09 | 2.13 | 3.43E-04 | Tmem150a | transmembrane protein 150A |
| ENSMUSG00000027004 | 1.42 | 2.68 | 4.79E-04 | 1.09 | 2.12 | 1.86E-03 | Frzb | frizzled-related protein |
| ENSMUSG00000029122 | 1.02 | 2.03 | 4.20E-05 | 1.09 | 2.12 | 1.18E-07 | Evc | Ellis van Creveld gene syndrome |
| ENSMUSG00000029710 | 1.02 | 2.03 | 5.10E-05 | 1.08 | 2.12 | 2.39E-07 | Ephb4 | Eph receptor B4 |
| ENSMUSG00000033998 | 1.13 | 2.18 | 3.24E-05 | 1.09 | 2.12 | 8.82E-07 | Kcnk1 | potassium channel, subfamily K, member 1 |
| ENSMUSG00000040287 | 1.19 | 2.27 | 9.68E-04 | 1.08 | 2.12 | 3.88E-04 | Stac3 | SH3 and cysteine rich domain 3 |
| ENSMUSG00000023495 | 1.28 | 2.42 | 1.30E-04 | 1.08 | 2.11 | 1.47E-04 | Pcbp4 | poly(rC) binding protein 4 |
| ENSMUSG00000034175 | 1.20 | 2.30 | 3.06E-04 | 1.08 | 2.11 | 1.08E-04 | Rhbdd3 | rhomboid domain containing 3 |
| ENSMUSG00000037577 | 1.67 | 3.19 | 2.99E-04 | 1.07 | 2.10 | 6.36E-03 | Ephx3 | epoxide hydrolase 3 |
| ENSMUSG00000039208 | 1.58 | 2.98 | 1.30E-07 | 1.07 | 2.10 | 2.15E-05 | Metrnl | meteorin, glial cell differentiation regulator-like |
| ENSMUSG00000044328 | 1.33 | 2.51 | 2.24E-05 | 1.07 | 2.10 | 4.69E-05 | Trp53i13 | transformation related protein 53 inducible protein 13 |
| ENSMUSG00000074280 | 1.11 | 2.15 | 1.15E-04 | 1.07 | 2.09 | 9.01E-06 | NA | NA |
| ENSMUSG00000027894 | 1.02 | 2.03 | 9.80E-03 | 1.06 | 2.08 | 8.25E-04 | Slc6a17 | solute carrier family 6 (neurotransmitter transporter), member 17 |
| ENSMUSG00000034845 | 1.05 | 2.07 | 3.66E-03 | 1.06 | 2.08 | 5.91E-04 | Plvap | plasmalemma vesicle associated protein |
| ENSMUSG00000020722 | 1.30 | 2.47 | 3.66E-04 | 1.05 | 2.07 | 7.98E-04 | Cacng1 | calcium channel, voltage-dependent, gamma subunit 1 |
| ENSMUSG00000029757 | 1.35 | 2.55 | 5.42E-04 | 1.05 | 2.07 | 1.24E-03 | Dync1i1 | dynein cytoplasmic 1 intermediate chain 1 |
| ENSMUSG00000033389 | 1.17 | 2.24 | 1.61E-03 | 1.04 | 2.06 | 6.63E-04 | Arhgap44 | Rho GTPase activating protein 44 |
| ENSMUSG00000034220 | 1.25 | 2.38 | 6.09E-10 | 1.04 | 2.06 | 3.20E-10 | Gpc1 | glypican 1 |
| ENSMUSG00000001751 | 1.20 | 2.30 | 5.74E-07 | 1.03 | 2.05 | 2.11E-07 | Naglu | alpha-N-acetylglucosaminidase (Sanfilippo disease IIIB) |
| ENSMUSG00000026930 | 1.08 | 2.11 | 2.09E-04 | 1.04 | 2.05 | 2.07E-05 | Gpsm1 | G-protein signalling modulator 1 (AGS3-like, C. elegans) |
| ENSMUSG00000030621 | 1.05 | 2.07 | 5.90E-03 | 1.04 | 2.05 | 1.22E-03 | Me3 | malic enzyme 3, NADP(+)-dependent, mitochondrial |
| ENSMUSG00000021456 | 1.08 | 2.11 | 7.13E-03 | 1.02 | 2.02 | 2.55E-03 | Fbp2 | fructose bisphosphatase 2 |
| ENSMUSG00000026825 | 1.49 | 2.81 | 2.25E-06 | 1.02 | 2.02 | 1.52E-04 | Dnm1 | dynamin 1 |
| ENSMUSG00000031955 | 1.17 | 2.25 | 7.97E-06 | 1.01 | 2.02 | 3.90E-06 | Bcar1 | breast cancer anti-estrogen resistance 1 |
| ENSMUSG00000068551 | 1.02 | 2.02 | 2.77E-04 | 1.01 | 2.01 | 1.43E-05 | Zfp467 | zinc finger protein 467 |

Down-regulated genes

|  | PC-3 xenografts | |  | MDA-MB231 xenografts | |  |  |  |
| --- | --- | --- | --- | --- | --- | --- | --- | --- |
| id | log2FoldChange | FC | padj | log2FoldChange | FC | padj | SYMBOL | GENENAME |
| ENSMUSG00000076609 | -1.82 | -3.53 | 1.80E-03 | -4.40 | -21.09 | 1.24E-17 | NA | NA |
| ENSMUSG00000050157 | -1.18 | -2.26 | 1.77E-03 | -4.32 | -19.93 | 1.08E-44 | NA | NA |
| ENSMUSG00000085606 | -1.88 | -3.68 | 1.33E-04 | -4.06 | -16.68 | 1.41E-20 | NA | NA |
| ENSMUSG00000031786 | -1.33 | -2.52 | 6.88E-05 | -4.05 | -16.59 | 8.36E-40 | Ccdc135 | coiled-coil domain containing 135 |
| ENSMUSG00000052019 | -1.81 | -3.49 | 1.46E-03 | -3.89 | -14.80 | 2.34E-16 | NA | NA |
| ENSMUSG00000086438 | -1.33 | -2.51 | 3.77E-03 | -3.74 | -13.33 | 7.02E-21 | Asb17os | ankyrin repeat and SOCS box-containing 17, opposite strand |
| ENSMUSG00000032556 | -1.69 | -3.22 | 9.20E-03 | -3.58 | -11.92 | 1.14E-10 | Bfsp2 | beaded filament structural protein 2, phakinin |
| ENSMUSG00000083186 | -2.47 | -5.53 | 7.30E-06 | -3.31 | -9.91 | 1.28E-13 | NA | NA |
| ENSMUSG00000046179 | -1.21 | -2.32 | 2.26E-04 | -3.30 | -9.82 | 1.75E-34 | E2f8 | E2F transcription factor 8 |
| ENSMUSG00000086741 | -1.44 | -2.72 | 1.17E-03 | -3.28 | -9.72 | 5.55E-19 | Gm15816 | predicted gene 15816 |
| ENSMUSG00000083438 | -1.87 | -3.65 | 4.36E-05 | -3.19 | -9.12 | 2.42E-18 | NA | NA |
| ENSMUSG00000086749 | -2.81 | -7.03 | 1.36E-03 | -3.08 | -8.48 | 7.55E-06 | NA | NA |
| ENSMUSG00000032221 | -1.53 | -2.90 | 3.98E-04 | -3.00 | -8.02 | 5.03E-17 | Mns1 | meiosis-specific nuclear structural protein 1 |
| ENSMUSG00000079472 | -1.68 | -3.20 | 1.60E-03 | -2.94 | -7.68 | 6.48E-12 | NA | NA |
| ENSMUSG00000021792 | -1.87 | -3.67 | 5.15E-06 | -2.93 | -7.60 | 7.20E-18 | Fam213a | family with sequence similarity 213, member A |
| ENSMUSG00000028307 | -1.31 | -2.48 | 9.69E-03 | -2.87 | -7.33 | 4.20E-12 | Aldob | aldolase B, fructose-bisphosphate |
| ENSMUSG00000084081 | -2.02 | -4.07 | 1.96E-05 | -2.87 | -7.31 | 9.75E-14 | NA | NA |
| ENSMUSG00000032864 | -1.68 | -3.21 | 2.06E-03 | -2.84 | -7.17 | 6.29E-11 | Rag2 | recombination activating gene 2 |
| ENSMUSG00000087313 | -1.33 | -2.52 | 2.27E-04 | -2.83 | -7.13 | 1.84E-21 | NA | NA |
| ENSMUSG00000005800 | -1.10 | -2.14 | 6.08E-03 | -2.75 | -6.74 | 2.97E-17 | Mmp8 | matrix metallopeptidase 8 |
| ENSMUSG00000037001 | -1.26 | -2.39 | 1.43E-05 | -2.72 | -6.58 | 1.29E-30 | Zfp39 | zinc finger protein 39 |
| ENSMUSG00000037124 | -1.21 | -2.31 | 7.14E-03 | -2.71 | -6.55 | 1.67E-13 | Trim58 | tripartite motif-containing 58 |
| ENSMUSG00000035246 | -1.05 | -2.08 | 9.86E-04 | -2.71 | -6.54 | 3.69E-25 | Pcyt1b | phosphate cytidylyltransferase 1, choline, beta isoform |
| ENSMUSG00000028906 | -1.06 | -2.09 | 1.71E-04 | -2.70 | -6.52 | 1.22E-31 | Epb4.1 | erythrocyte protein band 4.1 |
| ENSMUSG00000082458 | -1.04 | -2.05 | 3.52E-03 | -2.70 | -6.50 | 1.26E-20 | NA | NA |
| ENSMUSG00000026532 | -1.51 | -2.85 | 1.10E-04 | -2.68 | -6.42 | 1.19E-16 | Spta1 | spectrin alpha, erythrocytic 1 |
| ENSMUSG00000049460 | -1.30 | -2.47 | 1.06E-03 | -2.68 | -6.39 | 2.99E-16 | NA | NA |
| ENSMUSG00000020490 | -1.14 | -2.21 | 3.37E-03 | -2.64 | -6.24 | 1.22E-16 | Btnl10 | butyrophilin-like 10 |
| ENSMUSG00000037544 | -1.38 | -2.60 | 8.87E-05 | -2.63 | -6.19 | 1.22E-19 | Dlgap5 | discs, large (Drosophila) homolog-associated protein 5 |
| ENSMUSG00000036377 | -1.20 | -2.30 | 2.36E-03 | -2.60 | -6.05 | 1.21E-15 | C530008M17Rik | RIKEN cDNA C530008M17 gene |
| ENSMUSG00000059864 | -2.99 | -7.94 | 1.05E-03 | -2.58 | -5.99 | 3.57E-04 | Olfr1393 | olfactory receptor 1393 |
| ENSMUSG00000042489 | -1.59 | -3.01 | 5.79E-06 | -2.58 | -5.97 | 4.42E-19 | Clspn | claspin |
| ENSMUSG00000071528 | -1.71 | -3.27 | 5.96E-03 | -2.57 | -5.95 | 6.32E-07 | Usmg5 | upregulated during skeletal muscle growth 5 |
| ENSMUSG00000073067 | -1.14 | -2.20 | 7.46E-03 | -2.53 | -5.79 | 2.28E-13 | 9130019P16Rik | RIKEN cDNA 9130019P16 gene |
| ENSMUSG00000022876 | -1.67 | -3.18 | 4.67E-05 | -2.49 | -5.64 | 2.01E-13 | Samsn1 | SAM domain, SH3 domain and nuclear localization signals, 1 |
| ENSMUSG00000032028 | -1.79 | -3.45 | 4.41E-06 | -2.49 | -5.60 | 1.22E-14 | Nxpe2 | neurexophilin and PC-esterase domain family, member 2 |
| ENSMUSG00000082145 | -2.06 | -4.18 | 7.45E-07 | -2.46 | -5.52 | 2.17E-13 | NA | NA |
| ENSMUSG00000019982 | -1.03 | -2.04 | 1.55E-03 | -2.46 | -5.50 | 1.45E-20 | Myb | myeloblastosis oncogene |
| ENSMUSG00000027715 | -1.02 | -2.03 | 2.22E-04 | -2.45 | -5.46 | 3.22E-27 | Ccna2 | cyclin A2 |
| ENSMUSG00000028332 | -1.08 | -2.12 | 2.30E-03 | -2.43 | -5.39 | 5.91E-17 | Hemgn | hemogen |
| ENSMUSG00000083338 | -1.44 | -2.71 | 5.11E-03 | -2.43 | -5.38 | 3.74E-09 | NA | NA |
| ENSMUSG00000026614 | -1.44 | -2.71 | 3.13E-04 | -2.43 | -5.37 | 5.51E-14 | Slc30a10 | solute carrier family 30, member 10 |
| ENSMUSG00000051839 | -1.21 | -2.31 | 1.51E-03 | -2.41 | -5.32 | 1.54E-14 | Gypa | glycophorin A |
| ENSMUSG00000026579 | -1.12 | -2.17 | 3.27E-03 | -2.40 | -5.30 | 1.08E-14 | F5 | coagulation factor V |
| ENSMUSG00000051497 | -2.85 | -7.20 | 9.67E-04 | -2.37 | -5.19 | 2.21E-04 | Kcnj16 | potassium inwardly-rectifying channel, subfamily J, member 16 |
| ENSMUSG00000029178 | -1.64 | -3.12 | 3.48E-05 | -2.35 | -5.09 | 1.10E-12 | Klf3 | Kruppel-like factor 3 (basic) |
| ENSMUSG00000071226 | -1.15 | -2.22 | 1.55E-03 | -2.35 | -5.09 | 2.92E-15 | Cecr2 | cat eye syndrome chromosome region, candidate 2 |
| ENSMUSG00000074662 | -2.40 | -5.27 | 9.01E-03 | -2.35 | -5.08 | 1.79E-03 | NA | NA |
| ENSMUSG00000036223 | -1.22 | -2.33 | 9.15E-04 | -2.34 | -5.06 | 8.82E-15 | Ska1 | spindle and kinetochore associated complex subunit 1 |
| ENSMUSG00000034825 | -1.59 | -3.02 | 4.61E-05 | -2.31 | -4.96 | 4.76E-13 | Nrip3 | nuclear receptor interacting protein 3 |
| ENSMUSG00000032561 | -1.08 | -2.11 | 5.60E-03 | -2.27 | -4.84 | 6.00E-13 | Acpp | acid phosphatase, prostate |
| ENSMUSG00000084862 | -1.76 | -3.40 | 8.10E-03 | -2.27 | -4.82 | 2.40E-05 | NA | NA |
| ENSMUSG00000047139 | -1.11 | -2.16 | 3.62E-04 | -2.26 | -4.79 | 8.82E-19 | Cd24a | CD24a antigen |
| ENSMUSG00000069910 | -1.24 | -2.36 | 1.35E-05 | -2.26 | -4.79 | 1.14E-22 | Spdl1 | spindle apparatus coiled-coil protein 1 |
| ENSMUSG00000086587 | -1.70 | -3.25 | 1.05E-03 | -2.25 | -4.77 | 1.36E-08 | NA | NA |
| ENSMUSG00000024533 | -1.37 | -2.59 | 1.61E-06 | -2.24 | -4.74 | 8.08E-22 | Spire1 | spire homolog 1 (Drosophila) |
| ENSMUSG00000027469 | -1.01 | -2.01 | 1.72E-04 | -2.24 | -4.72 | 1.84E-24 | Tpx2 | TPX2, microtubule-associated protein homolog (Xenopus laevis) |
| ENSMUSG00000039809 | -2.19 | -4.55 | 2.69E-04 | -2.24 | -4.72 | 9.30E-07 | Gabbr2 | gamma-aminobutyric acid (GABA) B receptor, 2 |
| ENSMUSG00000085085 | -2.20 | -4.60 | 9.87E-03 | -2.22 | -4.67 | 5.61E-04 | NA | NA |
| ENSMUSG00000031004 | -2.11 | -4.31 | 2.35E-07 | -2.21 | -4.63 | 9.00E-11 | Mki67 | antigen identified by monoclonal antibody Ki 67 |
| ENSMUSG00000028528 | -1.02 | -2.02 | 8.31E-03 | -2.20 | -4.60 | 1.60E-12 | Dnajc6 | DnaJ (Hsp40) homolog, subfamily C, member 6 |
| ENSMUSG00000038725 | -1.30 | -2.46 | 8.89E-04 | -2.20 | -4.60 | 7.90E-12 | Pkhd1l1 | polycystic kidney and hepatic disease 1-like 1 |
| ENSMUSG00000074903 | -1.36 | -2.57 | 1.31E-06 | -2.20 | -4.60 | 4.30E-22 | NA | NA |
| ENSMUSG00000081952 | -1.90 | -3.74 | 5.26E-04 | -2.16 | -4.46 | 1.73E-06 | NA | NA |
| ENSMUSG00000028333 | -1.65 | -3.15 | 6.88E-05 | -2.14 | -4.41 | 7.38E-10 | Anp32b | acidic (leucine-rich) nuclear phosphoprotein 32 family, member B |
| ENSMUSG00000023926 | -1.27 | -2.41 | 1.05E-03 | -2.13 | -4.37 | 3.39E-11 | Rhag | Rhesus blood group-associated A glycoprotein |
| ENSMUSG00000061533 | -1.11 | -2.16 | 1.15E-04 | -2.11 | -4.33 | 2.38E-19 | Cep128 | centrosomal protein 128 |
| ENSMUSG00000082101 | -1.16 | -2.23 | 3.42E-03 | -2.11 | -4.33 | 7.72E-11 | Slfn14 | schlafen 14 |
| ENSMUSG00000084953 | -1.52 | -2.87 | 3.11E-03 | -2.11 | -4.32 | 9.90E-07 | NA | NA |
| ENSMUSG00000028328 | -1.08 | -2.11 | 6.95E-05 | -2.11 | -4.31 | 1.69E-21 | Tmod1 | tropomodulin 1 |
| ENSMUSG00000060429 | -1.82 | -3.53 | 8.14E-05 | -2.08 | -4.21 | 3.53E-08 | Sntb1 | syntrophin, basic 1 |
| ENSMUSG00000086453 | -1.53 | -2.90 | 1.81E-03 | -2.06 | -4.17 | 2.81E-08 | NA | NA |
| ENSMUSG00000020185 | -1.04 | -2.05 | 1.76E-03 | -2.03 | -4.09 | 5.56E-14 | E2f7 | E2F transcription factor 7 |
| ENSMUSG00000032397 | -1.06 | -2.08 | 1.48E-04 | -2.02 | -4.06 | 8.95E-19 | Tipin | timeless interacting protein |
| ENSMUSG00000038943 | -1.06 | -2.08 | 3.93E-04 | -2.02 | -4.06 | 1.45E-16 | Prc1 | protein regulator of cytokinesis 1 |
| ENSMUSG00000049092 | -1.49 | -2.81 | 3.97E-04 | -2.02 | -4.06 | 1.14E-10 | Gpr137c | G protein-coupled receptor 137C |
| ENSMUSG00000022659 | -1.17 | -2.24 | 2.75E-04 | -2.01 | -4.02 | 9.42E-15 | Gcsam | germinal center associated, signaling and motility |
| ENSMUSG00000022422 | -1.24 | -2.36 | 3.47E-04 | -2.01 | -4.01 | 7.38E-13 | Dscc1 | defective in sister chromatid cohesion 1 homolog (S. cerevisiae) |
| ENSMUSG00000084756 | -1.33 | -2.52 | 2.52E-03 | -2.00 | -4.00 | 4.73E-09 | NA | NA |
| ENSMUSG00000015342 | -1.34 | -2.54 | 8.77E-04 | -1.99 | -3.97 | 2.78E-09 | Xk | Kell blood group precursor (McLeod phenotype) homolog |
| ENSMUSG00000020990 | -1.09 | -2.13 | 2.60E-03 | -1.98 | -3.95 | 6.48E-12 | Cdkl1 | cyclin-dependent kinase-like 1 (CDC2-related kinase) |
| ENSMUSG00000021676 | -1.33 | -2.52 | 8.12E-06 | -1.98 | -3.95 | 4.69E-16 | Iqgap2 | IQ motif containing GTPase activating protein 2 |
| ENSMUSG00000054889 | -1.11 | -2.16 | 8.13E-03 | -1.98 | -3.95 | 5.74E-09 | Dsp | desmoplakin |
| ENSMUSG00000062588 | -1.81 | -3.52 | 2.70E-05 | -1.98 | -3.94 | 4.29E-08 | NA | NA |
| ENSMUSG00000020541 | -1.41 | -2.66 | 1.89E-08 | -1.97 | -3.92 | 1.72E-22 | Tom1l1 | target of myb1-like 1 (chicken) |
| ENSMUSG00000040170 | -1.51 | -2.84 | 1.31E-05 | -1.97 | -3.91 | 3.88E-12 | Fmo2 | flavin containing monooxygenase 2 |
| ENSMUSG00000034206 | -1.15 | -2.22 | 5.95E-05 | -1.94 | -3.85 | 7.32E-17 | Polq | polymerase (DNA directed), theta |
| ENSMUSG00000034566 | -1.43 | -2.70 | 3.53E-03 | -1.94 | -3.84 | 2.44E-06 | Atp5h | ATP synthase, H+ transporting, mitochondrial F0 complex, subunit D |
| ENSMUSG00000017146 | -1.35 | -2.56 | 2.38E-04 | -1.90 | -3.74 | 4.64E-10 | Brca1 | breast cancer 1 |
| ENSMUSG00000037922 | -1.48 | -2.80 | 5.07E-04 | -1.90 | -3.73 | 1.74E-08 | Bank1 | B cell scaffold protein with ankyrin repeats 1 |
| ENSMUSG00000044468 | -1.22 | -2.33 | 3.28E-04 | -1.87 | -3.66 | 3.40E-11 | Fam46c | family with sequence similarity 46, member C |
| ENSMUSG00000048327 | -1.49 | -2.81 | 1.42E-08 | -1.86 | -3.64 | 1.99E-18 | Ckap2l | cytoskeleton associated protein 2-like |
| ENSMUSG00000026672 | -1.08 | -2.11 | 1.67E-04 | -1.86 | -3.62 | 3.18E-15 | Optn | optineurin |
| ENSMUSG00000034311 | -1.05 | -2.06 | 6.58E-04 | -1.86 | -3.62 | 1.83E-13 | Kif4 | kinesin family member 4 |
| ENSMUSG00000019996 | -1.24 | -2.36 | 4.14E-05 | -1.84 | -3.59 | 4.50E-15 | Map7 | microtubule-associated protein 7 |
| ENSMUSG00000032350 | -1.00 | -2.00 | 1.37E-04 | -1.85 | -3.59 | 1.13E-17 | Gclc | glutamate-cysteine ligase, catalytic subunit |
| ENSMUSG00000020493 | -1.69 | -3.23 | 1.41E-07 | -1.84 | -3.58 | 2.42E-12 | Prr11 | proline rich 11 |
| ENSMUSG00000032411 | -1.02 | -2.03 | 5.32E-04 | -1.82 | -3.54 | 4.30E-14 | Tfdp2 | transcription factor Dp 2 |
| ENSMUSG00000023169 | -1.08 | -2.11 | 5.65E-03 | -1.82 | -3.53 | 1.29E-08 | Slc38a1 | solute carrier family 38, member 1 |
| ENSMUSG00000036390 | -1.20 | -2.30 | 1.16E-04 | -1.79 | -3.45 | 3.29E-12 | Gadd45a | growth arrest and DNA-damage-inducible 45 alpha |
| ENSMUSG00000022673 | -1.02 | -2.02 | 1.76E-06 | -1.78 | -3.44 | 7.77E-25 | Mcm4 | minichromosome maintenance deficient 4 homolog (S. cerevisiae) |
| ENSMUSG00000023940 | -1.31 | -2.48 | 1.15E-04 | -1.78 | -3.44 | 1.94E-10 | Sgol1 | shugoshin-like 1 (S. pombe) |
| ENSMUSG00000026605 | -1.78 | -3.43 | 9.18E-07 | -1.78 | -3.44 | 3.33E-09 | Cenpf | centromere protein F |
| ENSMUSG00000083367 | -1.26 | -2.39 | 1.56E-05 | -1.78 | -3.44 | 1.10E-13 | NA | NA |
| ENSMUSG00000041147 | -1.21 | -2.32 | 8.19E-05 | -1.77 | -3.42 | 1.94E-12 | Brca2 | breast cancer 2 |
| ENSMUSG00000022100 | -1.24 | -2.36 | 1.75E-04 | -1.77 | -3.40 | 1.27E-10 | Xpo7 | exportin 7 |
| ENSMUSG00000020124 | -1.21 | -2.32 | 2.47E-05 | -1.76 | -3.39 | 1.24E-13 | Usp15 | ubiquitin specific peptidase 15 |
| ENSMUSG00000048489 | -1.51 | -2.84 | 4.51E-04 | -1.76 | -3.39 | 8.83E-07 | 8430408G22Rik | RIKEN cDNA 8430408G22 gene |
| ENSMUSG00000015749 | -1.46 | -2.76 | 4.41E-06 | -1.75 | -3.37 | 3.34E-11 | Anp32e | acidic (leucine-rich) nuclear phosphoprotein 32 family, member E |
| ENSMUSG00000026028 | -1.41 | -2.67 | 7.83E-04 | -1.75 | -3.37 | 7.73E-07 | Trak2 | trafficking protein, kinesin binding 2 |
| ENSMUSG00000026348 | -1.09 | -2.12 | 7.46E-03 | -1.75 | -3.37 | 6.58E-08 | Acmsd | amino carboxymuconate semialdehyde decarboxylase |
| ENSMUSG00000034997 | -2.32 | -4.99 | 2.24E-04 | -1.74 | -3.35 | 3.62E-04 | Htr2a | 5-hydroxytryptamine (serotonin) receptor 2A |
| ENSMUSG00000044966 | -2.09 | -4.25 | 2.20E-04 | -1.74 | -3.35 | 1.04E-04 | Fbxo48 | F-box protein 48 |
| ENSMUSG00000027962 | -1.51 | -2.86 | 1.66E-07 | -1.71 | -3.28 | 8.41E-13 | Vcam1 | vascular cell adhesion molecule 1 |
| ENSMUSG00000053965 | -1.49 | -2.80 | 2.38E-03 | -1.72 | -3.28 | 3.28E-05 | Pde5a | phosphodiesterase 5A, cGMP-specific |
| ENSMUSG00000034906 | -1.19 | -2.29 | 4.09E-05 | -1.70 | -3.26 | 1.04E-12 | Ncaph | non-SMC condensin I complex, subunit H |
| ENSMUSG00000083834 | -2.36 | -5.12 | 9.31E-04 | -1.68 | -3.20 | 3.26E-03 | NA | NA |
| ENSMUSG00000049493 | -1.04 | -2.05 | 2.24E-04 | -1.67 | -3.19 | 1.57E-13 | Pls1 | plastin 1 (I-isoform) |
| ENSMUSG00000030154 | -1.55 | -2.92 | 6.57E-05 | -1.67 | -3.18 | 2.31E-08 | Klrb1f | killer cell lectin-like receptor subfamily B member 1F |
| ENSMUSG00000033952 | -1.54 | -2.90 | 2.91E-05 | -1.67 | -3.18 | 5.07E-08 | Aspm | asp (abnormal spindle)-like, microcephaly associated (Drosophila) |
| ENSMUSG00000029414 | -1.01 | -2.02 | 4.42E-04 | -1.66 | -3.16 | 1.77E-12 | Kntc1 | kinetochore associated 1 |
| ENSMUSG00000027242 | -1.15 | -2.22 | 1.60E-04 | -1.65 | -3.15 | 4.09E-11 | Wdr76 | WD repeat domain 76 |
| ENSMUSG00000020914 | -1.22 | -2.34 | 9.15E-06 | -1.65 | -3.14 | 4.29E-13 | Top2a | topoisomerase (DNA) II alpha |
| ENSMUSG00000035024 | -1.09 | -2.13 | 1.59E-04 | -1.65 | -3.13 | 4.12E-12 | Ncapd3 | non-SMC condensin II complex, subunit D3 |
| ENSMUSG00000026196 | -1.06 | -2.08 | 4.11E-04 | -1.64 | -3.11 | 1.47E-11 | Bard1 | BRCA1 associated RING domain 1 |
| ENSMUSG00000004110 | -1.56 | -2.95 | 8.05E-04 | -1.63 | -3.10 | 9.62E-07 | Cacna1e | calcium channel, voltage-dependent, R type, alpha 1E subunit |
| ENSMUSG00000023883 | -1.04 | -2.05 | 5.10E-04 | -1.63 | -3.10 | 4.02E-11 | Phf10 | PHD finger protein 10 |
| ENSMUSG00000021118 | -1.16 | -2.23 | 7.42E-05 | -1.63 | -3.09 | 1.35E-12 | Plek2 | pleckstrin 2 |
| ENSMUSG00000022026 | -1.22 | -2.33 | 5.85E-04 | -1.62 | -3.07 | 4.55E-08 | Olfm4 | olfactomedin 4 |
| ENSMUSG00000037313 | -1.21 | -2.32 | 1.47E-04 | -1.62 | -3.07 | 1.09E-09 | Tacc3 | transforming, acidic coiled-coil containing protein 3 |
| ENSMUSG00000081289 | -1.04 | -2.06 | 4.40E-03 | -1.62 | -3.07 | 4.85E-08 | NA | NA |
| ENSMUSG00000028718 | -1.42 | -2.67 | 6.94E-06 | -1.61 | -3.06 | 3.81E-10 | Stil | Scl/Tal1 interrupting locus |
| ENSMUSG00000030528 | -1.71 | -3.27 | 1.35E-05 | -1.61 | -3.06 | 9.19E-07 | Blm | Bloom syndrome, RecQ helicase-like |
| ENSMUSG00000032854 | -1.25 | -2.39 | 2.22E-03 | -1.61 | -3.05 | 2.04E-06 | Ugt8a | UDP galactosyltransferase 8A |
| ENSMUSG00000007480 | -1.86 | -3.62 | 4.22E-04 | -1.60 | -3.04 | 1.38E-05 | Mc5r | melanocortin 5 receptor |
| ENSMUSG00000029613 | -1.06 | -2.09 | 3.07E-05 | -1.60 | -3.04 | 2.09E-14 | Eif2ak1 | eukaryotic translation initiation factor 2 alpha kinase 1 |
| ENSMUSG00000039187 | -1.38 | -2.60 | 2.68E-04 | -1.60 | -3.04 | 2.46E-07 | Fanci | Fanconi anemia, complementation group I |
| ENSMUSG00000004880 | -1.03 | -2.04 | 9.27E-04 | -1.59 | -3.00 | 7.05E-10 | Lbr | lamin B receptor |
| ENSMUSG00000047534 | -2.16 | -4.47 | 9.38E-07 | -1.58 | -3.00 | 1.91E-05 | Mis18bp1 | MIS18 binding protein 1 |
| ENSMUSG00000026463 | -1.25 | -2.38 | 2.96E-04 | -1.58 | -2.99 | 4.75E-08 | Atp2b4 | ATPase, Ca++ transporting, plasma membrane 4 |
| ENSMUSG00000031583 | -1.92 | -3.78 | 2.20E-09 | -1.57 | -2.97 | 1.27E-09 | Wrn | Werner syndrome homolog (human) |
| ENSMUSG00000039396 | -1.49 | -2.80 | 6.06E-05 | -1.56 | -2.95 | 4.44E-07 | Neil3 | nei like 3 (E. coli) |
| ENSMUSG00000041498 | -1.26 | -2.39 | 2.42E-04 | -1.56 | -2.95 | 3.64E-08 | Kif14 | kinesin family member 14 |
| ENSMUSG00000061607 | -1.35 | -2.55 | 2.72E-05 | -1.55 | -2.94 | 6.39E-09 | Mdc1 | mediator of DNA damage checkpoint 1 |
| ENSMUSG00000040693 | -1.91 | -3.76 | 1.04E-03 | -1.55 | -2.93 | 1.86E-03 | Slco4c1 | solute carrier organic anion transporter family, member 4C1 |
| ENSMUSG00000051910 | -1.32 | -2.50 | 3.25E-05 | -1.55 | -2.92 | 4.62E-09 | Sox6 | SRY (sex determining region Y)-box 6 |
| ENSMUSG00000029227 | -1.27 | -2.42 | 4.21E-05 | -1.54 | -2.90 | 2.79E-09 | Fip1l1 | FIP1 like 1 (S. cerevisiae) |
| ENSMUSG00000064367 | -1.46 | -2.76 | 7.29E-04 | -1.54 | -2.90 | 3.07E-05 | ND5 | NADH dehydrogenase subunit 5 |
| ENSMUSG00000070733 | -1.00 | -2.00 | 3.63E-03 | -1.54 | -2.90 | 7.33E-08 | Fryl | furry homolog-like (Drosophila) |
| ENSMUSG00000075569 | -2.63 | -6.19 | 1.13E-03 | -1.53 | -2.90 | 9.01E-03 | Rsph10b | radial spoke head 10 homolog B (Chlamydomonas) |
| ENSMUSG00000082674 | -1.94 | -3.83 | 1.00E-05 | -1.54 | -2.90 | 1.50E-05 | NA | NA |
| ENSMUSG00000078973 | -2.03 | -4.07 | 3.96E-04 | -1.53 | -2.89 | 5.75E-04 | NA | NA |
| ENSMUSG00000050243 | -2.06 | -4.18 | 1.62E-06 | -1.53 | -2.88 | 1.70E-05 | NA | NA |
| ENSMUSG00000057399 | -1.59 | -3.02 | 3.03E-03 | -1.52 | -2.87 | 4.08E-04 | NA | NA |
| ENSMUSG00000073542 | -1.09 | -2.13 | 2.72E-05 | -1.52 | -2.87 | 5.48E-13 | Cep76 | centrosomal protein 76 |
| ENSMUSG00000089686 | -1.55 | -2.94 | 4.89E-03 | -1.52 | -2.87 | 6.56E-04 | NA | NA |
| ENSMUSG00000044229 | -1.23 | -2.35 | 6.86E-05 | -1.51 | -2.85 | 4.35E-10 | Nxpe4 | neurexophilin and PC-esterase domain family, member 4 |
| ENSMUSG00000026683 | -1.36 | -2.57 | 5.59E-05 | -1.49 | -2.82 | 1.04E-07 | Nuf2 | NUF2, NDC80 kinetochore complex component, homolog (S. cerevisiae) |
| ENSMUSG00000066800 | -1.43 | -2.69 | 1.76E-03 | -1.50 | -2.82 | 1.05E-04 | Rnasel | ribonuclease L (2', 5'-oligoisoadenylate synthetase-dependent) |
| ENSMUSG00000014361 | -1.15 | -2.22 | 4.37E-04 | -1.49 | -2.81 | 3.63E-08 | Mertk | c-mer proto-oncogene tyrosine kinase |
| ENSMUSG00000054720 | -1.03 | -2.04 | 2.44E-08 | -1.49 | -2.81 | 5.63E-24 | Lrrc8c | leucine rich repeat containing 8 family, member C |
| ENSMUSG00000037474 | -1.06 | -2.09 | 4.83E-04 | -1.49 | -2.80 | 2.98E-09 | Dtl | denticleless homolog (Drosophila) |
| ENSMUSG00000067367 | -1.20 | -2.29 | 2.91E-04 | -1.48 | -2.80 | 6.54E-08 | Lyar | Ly1 antibody reactive clone |
| ENSMUSG00000024228 | -1.03 | -2.04 | 4.83E-03 | -1.46 | -2.76 | 2.52E-07 | Nudt12 | nudix (nucleoside diphosphate linked moiety X)-type motif 12 |
| ENSMUSG00000037572 | -1.31 | -2.47 | 9.61E-07 | -1.46 | -2.76 | 2.04E-11 | Wdhd1 | WD repeat and HMG-box DNA binding protein 1 |
| ENSMUSG00000030254 | -1.48 | -2.78 | 8.93E-08 | -1.46 | -2.75 | 4.52E-11 | Rad18 | RAD18 homolog (S. cerevisiae) |
| ENSMUSG00000034023 | -1.28 | -2.43 | 1.25E-05 | -1.46 | -2.75 | 8.70E-10 | Fancd2 | Fanconi anemia, complementation group D2 |
| ENSMUSG00000063810 | -1.68 | -3.21 | 1.37E-05 | -1.45 | -2.74 | 3.20E-06 | Alms1 | Alstrom syndrome 1 |
| ENSMUSG00000022148 | -1.31 | -2.47 | 2.19E-05 | -1.45 | -2.73 | 1.50E-08 | Fyb | FYN binding protein |
| ENSMUSG00000044211 | -2.36 | -5.13 | 5.61E-06 | -1.45 | -2.73 | 1.23E-03 | NA | NA |
| ENSMUSG00000021754 | -1.38 | -2.60 | 2.16E-06 | -1.44 | -2.72 | 1.74E-09 | Map3k1 | mitogen-activated protein kinase kinase kinase 1 |
| ENSMUSG00000024524 | -1.60 | -3.04 | 5.93E-03 | -1.44 | -2.72 | 5.31E-04 | Gnal | guanine nucleotide binding protein, alpha stimulating, olfactory type |
| ENSMUSG00000027508 | -1.20 | -2.30 | 8.12E-05 | -1.44 | -2.71 | 1.28E-08 | Pag1 | phosphoprotein associated with glycosphingolipid microdomains 1 |
| ENSMUSG00000030142 | -1.35 | -2.54 | 2.81E-04 | -1.44 | -2.71 | 2.75E-06 | Clec4e | C-type lectin domain family 4, member e |
| ENSMUSG00000082988 | -1.14 | -2.20 | 1.01E-03 | -1.44 | -2.71 | 5.38E-07 | NA | NA |
| ENSMUSG00000002297 | -1.20 | -2.30 | 1.21E-04 | -1.43 | -2.69 | 3.38E-08 | Dbf4 | DBF4 homolog (S. cerevisiae) |
| ENSMUSG00000086651 | -1.29 | -2.45 | 8.73E-05 | -1.43 | -2.69 | 8.20E-08 | NA | NA |
| ENSMUSG00000011960 | -1.50 | -2.83 | 7.57E-03 | -1.41 | -2.66 | 3.31E-03 | Ccnt1 | cyclin T1 |
| ENSMUSG00000027505 | -2.18 | -4.54 | 2.98E-03 | -1.41 | -2.66 | 5.38E-03 | Fam209 | family with sequence similarity 209 |
| ENSMUSG00000028132 | -1.61 | -3.05 | 4.03E-06 | -1.41 | -2.66 | 1.19E-06 | Tmem56 | transmembrane protein 56 |
| ENSMUSG00000026915 | -1.13 | -2.20 | 3.48E-05 | -1.41 | -2.65 | 2.04E-10 | Strbp | spermatid perinuclear RNA binding protein |
| ENSMUSG00000031647 | -1.54 | -2.91 | 1.65E-05 | -1.39 | -2.63 | 2.37E-06 | Mfap3l | microfibrillar-associated protein 3-like |
| ENSMUSG00000090215 | -2.36 | -5.15 | 1.09E-07 | -1.39 | -2.63 | 1.14E-05 | Trim34b | tripartite motif-containing 34B |
| ENSMUSG00000024680 | -1.56 | -2.95 | 3.00E-03 | -1.39 | -2.62 | 4.60E-04 | Ms4a2 | membrane-spanning 4-domains, subfamily A, member 2 |
| ENSMUSG00000058624 | -1.67 | -3.18 | 1.30E-06 | -1.38 | -2.61 | 1.88E-06 | Gda | guanine deaminase |
| ENSMUSG00000035248 | -1.27 | -2.42 | 5.31E-05 | -1.37 | -2.59 | 1.96E-07 | Zcchc6 | zinc finger, CCHC domain containing 6 |
| ENSMUSG00000035367 | -1.02 | -2.03 | 5.76E-04 | -1.37 | -2.59 | 1.24E-08 | Rmi1 | RMI1, RecQ mediated genome instability 1, homolog (S. cerevisiae) |
| ENSMUSG00000035455 | -1.11 | -2.15 | 8.97E-06 | -1.38 | -2.59 | 8.71E-12 | Fignl1 | fidgetin-like 1 |
| ENSMUSG00000053846 | -1.02 | -2.03 | 4.85E-03 | -1.37 | -2.59 | 3.80E-06 | Lipg | lipase, endothelial |
| ENSMUSG00000084113 | -1.14 | -2.20 | 8.31E-03 | -1.37 | -2.59 | 1.38E-04 | NA | NA |
| ENSMUSG00000041219 | -1.20 | -2.30 | 4.01E-05 | -1.37 | -2.58 | 1.80E-08 | Arhgap11a | Rho GTPase activating protein 11A |
| ENSMUSG00000081865 | -1.22 | -2.32 | 6.33E-05 | -1.36 | -2.57 | 9.85E-09 | NA | NA |
| ENSMUSG00000022021 | -1.44 | -2.72 | 6.25E-07 | -1.36 | -2.56 | 7.83E-09 | Diap3 | diaphanous homolog 3 (Drosophila) |
| ENSMUSG00000027306 | -1.33 | -2.51 | 2.23E-05 | -1.35 | -2.56 | 2.22E-07 | Nusap1 | nucleolar and spindle associated protein 1 |
| ENSMUSG00000027692 | -1.35 | -2.54 | 6.86E-04 | -1.35 | -2.55 | 5.39E-05 | Tnik | TRAF2 and NCK interacting kinase |
| ENSMUSG00000073905 | -1.54 | -2.92 | 7.95E-06 | -1.35 | -2.55 | 1.89E-06 | NA | NA |
| ENSMUSG00000052572 | -1.64 | -3.11 | 2.00E-05 | -1.35 | -2.54 | 1.18E-05 | Dlg2 | discs, large homolog 2 (Drosophila) |
| ENSMUSG00000051235 | -1.50 | -2.83 | 7.28E-06 | -1.34 | -2.53 | 6.84E-07 | Gen1 | Gen homolog 1, endonuclease (Drosophila) |
| ENSMUSG00000006678 | -1.73 | -3.31 | 1.10E-08 | -1.33 | -2.52 | 6.16E-08 | Pola1 | polymerase (DNA directed), alpha 1 |
| ENSMUSG00000081152 | -1.15 | -2.22 | 1.90E-04 | -1.33 | -2.52 | 6.83E-08 | NA | NA |
| ENSMUSG00000085687 | -1.65 | -3.14 | 6.92E-04 | -1.34 | -2.52 | 8.23E-04 | NA | NA |
| ENSMUSG00000000804 | -1.37 | -2.58 | 3.94E-05 | -1.32 | -2.50 | 2.30E-06 | Usp32 | ubiquitin specific peptidase 32 |
| ENSMUSG00000019961 | -1.11 | -2.16 | 1.59E-04 | -1.32 | -2.50 | 7.76E-08 | Tmpo | thymopoietin |
| ENSMUSG00000032555 | -1.17 | -2.25 | 2.82E-05 | -1.32 | -2.50 | 1.18E-08 | Topbp1 | topoisomerase (DNA) II binding protein 1 |
| ENSMUSG00000056025 | -1.04 | -2.05 | 7.95E-03 | -1.32 | -2.49 | 4.49E-05 | Clca1 | chloride channel calcium activated 1 |
| ENSMUSG00000035492 | -1.56 | -2.94 | 8.78E-06 | -1.31 | -2.48 | 9.09E-06 | NA | NA |
| ENSMUSG00000038379 | -1.48 | -2.80 | 8.79E-07 | -1.31 | -2.47 | 1.15E-07 | Ttk | Ttk protein kinase |
| ENSMUSG00000087118 | -2.43 | -5.40 | 2.04E-04 | -1.28 | -2.44 | 1.26E-03 | NA | NA |
| ENSMUSG00000022710 | -1.24 | -2.36 | 7.68E-07 | -1.28 | -2.42 | 9.17E-10 | Usp7 | ubiquitin specific peptidase 7 |
| ENSMUSG00000028394 | -1.03 | -2.04 | 1.58E-04 | -1.26 | -2.40 | 2.02E-08 | Pole3 | polymerase (DNA directed), epsilon 3 (p17 subunit) |
| ENSMUSG00000022512 | -1.19 | -2.28 | 9.99E-04 | -1.26 | -2.39 | 2.92E-05 | Cldn1 | claudin 1 |
| ENSMUSG00000051316 | -1.47 | -2.76 | 3.30E-04 | -1.25 | -2.38 | 2.65E-04 | Taf7 | TAF7 RNA polymerase II, TATA box binding protein (TBP)-associated factor |
| ENSMUSG00000027699 | -1.70 | -3.24 | 3.09E-07 | -1.24 | -2.37 | 6.79E-06 | Ect2 | ect2 oncogene |
| ENSMUSG00000034574 | -1.46 | -2.76 | 2.52E-07 | -1.24 | -2.37 | 1.27E-07 | Daam1 | dishevelled associated activator of morphogenesis 1 |
| ENSMUSG00000025899 | -2.13 | -4.36 | 7.82E-08 | -1.24 | -2.36 | 1.23E-04 | Alkbh8 | alkB, alkylation repair homolog 8 (E. coli) |
| ENSMUSG00000027843 | -1.23 | -2.35 | 4.08E-04 | -1.24 | -2.36 | 2.16E-05 | Ptpn22 | protein tyrosine phosphatase, non-receptor type 22 (lymphoid) |
| ENSMUSG00000032915 | -1.21 | -2.32 | 1.54E-03 | -1.24 | -2.36 | 4.64E-05 | Emr4 | EGF-like module containing, mucin-like, hormone receptor-like sequence 4 |
| ENSMUSG00000033596 | -1.03 | -2.04 | 6.45E-06 | -1.24 | -2.36 | 2.85E-11 | Rfwd3 | ring finger and WD repeat domain 3 |
| ENSMUSG00000035021 | -1.15 | -2.22 | 1.03E-03 | -1.24 | -2.36 | 2.66E-05 | Baz1a | bromodomain adjacent to zinc finger domain 1A |
| ENSMUSG00000022899 | -1.23 | -2.35 | 2.64E-03 | -1.23 | -2.35 | 1.15E-04 | Slc15a2 | solute carrier family 15 (H+/peptide transporter), member 2 |
| ENSMUSG00000017550 | -1.92 | -3.78 | 5.75E-06 | -1.23 | -2.34 | 6.58E-04 | Atad5 | ATPase family, AAA domain containing 5 |
| ENSMUSG00000069913 | -1.88 | -3.67 | 3.51E-06 | -1.21 | -2.32 | 3.12E-04 | NA | NA |
| ENSMUSG00000030978 | -1.04 | -2.06 | 2.66E-08 | -1.21 | -2.31 | 3.18E-15 | Rrm1 | ribonucleotide reductase M1 |
| ENSMUSG00000006715 | -1.01 | -2.01 | 4.70E-04 | -1.20 | -2.29 | 4.87E-07 | Gmnn | geminin |
| ENSMUSG00000066057 | -1.78 | -3.44 | 3.81E-06 | -1.20 | -2.29 | 3.60E-05 | Gm1976 | predicted gene 1976 |
| ENSMUSG00000006169 | -1.06 | -2.08 | 7.08E-05 | -1.18 | -2.27 | 1.12E-07 | Clint1 | clathrin interactor 1 |
| ENSMUSG00000036825 | -1.06 | -2.09 | 1.19E-04 | -1.18 | -2.27 | 2.23E-07 | Ssx2ip | synovial sarcoma, X breakpoint 2 interacting protein |
| ENSMUSG00000022314 | -1.53 | -2.90 | 1.60E-06 | -1.18 | -2.26 | 1.30E-05 | Rad21 | RAD21 homolog (S. pombe) |
| ENSMUSG00000034329 | -1.45 | -2.72 | 1.13E-05 | -1.17 | -2.26 | 9.59E-06 | Brip1 | BRCA1 interacting protein C-terminal helicase 1 |
| ENSMUSG00000054641 | -1.34 | -2.54 | 6.03E-08 | -1.17 | -2.26 | 7.53E-09 | Mmrn1 | multimerin 1 |
| ENSMUSG00000057808 | -1.29 | -2.44 | 4.94E-05 | -1.18 | -2.26 | 5.66E-06 | NA | NA |
| ENSMUSG00000042029 | -1.83 | -3.57 | 5.23E-06 | -1.17 | -2.25 | 6.91E-04 | Ncapg2 | non-SMC condensin II complex, subunit G2 |
| ENSMUSG00000083545 | -1.22 | -2.34 | 4.27E-03 | -1.17 | -2.25 | 5.57E-04 | NA | NA |
| ENSMUSG00000022364 | -1.06 | -2.08 | 9.57E-04 | -1.16 | -2.23 | 1.26E-05 | Tbc1d31 | TBC1 domain family, member 31 |
| ENSMUSG00000024290 | -1.24 | -2.36 | 1.08E-05 | -1.16 | -2.23 | 7.13E-07 | Rock1 | Rho-associated coiled-coil containing protein kinase 1 |
| ENSMUSG00000026069 | -1.18 | -2.26 | 9.31E-04 | -1.15 | -2.23 | 8.57E-05 | Il1rl1 | interleukin 1 receptor-like 1 |
| ENSMUSG00000026357 | -1.68 | -3.20 | 5.79E-06 | -1.15 | -2.23 | 2.40E-04 | Rgs18 | regulator of G-protein signaling 18 |
| ENSMUSG00000034192 | -1.09 | -2.12 | 7.64E-03 | -1.16 | -2.23 | 7.25E-04 | Lsm3 | LSM3 homolog, U6 small nuclear RNA associated (S. cerevisiae) |
| ENSMUSG00000045328 | -1.81 | -3.51 | 1.31E-05 | -1.15 | -2.22 | 1.27E-03 | Cenpe | centromere protein E |
| ENSMUSG00000079108 | -2.02 | -4.05 | 3.17E-05 | -1.15 | -2.22 | 2.54E-03 | NA | NA |
| ENSMUSG00000032261 | -1.15 | -2.22 | 1.31E-04 | -1.15 | -2.21 | 2.14E-06 | Sh3bgrl2 | SH3 domain binding glutamic acid-rich protein like 2 |
| ENSMUSG00000020641 | -1.27 | -2.40 | 1.15E-07 | -1.13 | -2.20 | 1.04E-08 | Rsad2 | radical S-adenosyl methionine domain containing 2 |
| ENSMUSG00000024135 | -1.04 | -2.05 | 3.73E-06 | -1.14 | -2.20 | 2.16E-10 | Srbd1 | S1 RNA binding domain 1 |
| ENSMUSG00000024283 | -1.35 | -2.55 | 5.32E-05 | -1.14 | -2.20 | 5.45E-05 | Wac | WW domain containing adaptor with coiled-coil |
| ENSMUSG00000059288 | -1.15 | -2.22 | 3.33E-07 | -1.14 | -2.20 | 3.02E-10 | Cdyl | chromodomain protein, Y chromosome-like |
| ENSMUSG00000070729 | -1.28 | -2.43 | 7.56E-07 | -1.13 | -2.20 | 1.60E-07 | NA | NA |
| ENSMUSG00000022105 | -2.20 | -4.61 | 9.26E-08 | -1.13 | -2.19 | 1.35E-03 | Rb1 | retinoblastoma 1 |
| ENSMUSG00000038587 | -1.59 | -3.02 | 2.52E-07 | -1.13 | -2.19 | 1.04E-05 | Akap12 | A kinase (PRKA) anchor protein (gravin) 12 |
| ENSMUSG00000044749 | -1.56 | -2.94 | 3.49E-03 | -1.13 | -2.19 | 5.81E-03 | Abca6 | ATP-binding cassette, sub-family A (ABC1), member 6 |
| ENSMUSG00000051220 | -1.38 | -2.61 | 7.10E-06 | -1.13 | -2.18 | 6.94E-06 | Ercc6l | excision repair cross-complementing rodent repair deficiency complementation group 6 like |
| ENSMUSG00000041997 | -1.24 | -2.36 | 6.82E-08 | -1.12 | -2.17 | 3.13E-09 | Tlk1 | tousled-like kinase 1 |
| ENSMUSG00000031196 | -1.10 | -2.15 | 1.29E-03 | -1.11 | -2.16 | 2.66E-05 | F8 | coagulation factor VIII |
| ENSMUSG00000033107 | -1.48 | -2.79 | 2.68E-03 | -1.11 | -2.15 | 6.58E-03 | Rnf125 | ring finger protein 125 |
| ENSMUSG00000012443 | -1.18 | -2.26 | 2.35E-04 | -1.09 | -2.13 | 5.71E-05 | Kif11 | kinesin family member 11 |
| ENSMUSG00000034801 | -1.09 | -2.13 | 9.70E-05 | -1.09 | -2.13 | 3.10E-06 | Sos2 | son of sevenless homolog 2 (Drosophila) |
| ENSMUSG00000042167 | -1.33 | -2.52 | 2.22E-04 | -1.09 | -2.12 | 3.35E-04 | Papd4 | PAP associated domain containing 4 |
| ENSMUSG00000024513 | -1.08 | -2.12 | 9.81E-04 | -1.08 | -2.11 | 1.07E-04 | Mbd2 | methyl-CpG binding domain protein 2 |
| ENSMUSG00000027115 | -1.74 | -3.33 | 2.21E-06 | -1.08 | -2.11 | 4.06E-04 | Kif18a | kinesin family member 18A |
| ENSMUSG00000039697 | -2.30 | -4.92 | 1.63E-07 | -1.08 | -2.11 | 4.33E-03 | Ncoa7 | nuclear receptor coactivator 7 |
| ENSMUSG00000078933 | -1.53 | -2.89 | 3.04E-04 | -1.07 | -2.10 | 9.88E-04 | NA | NA |
| ENSMUSG00000021520 | -1.39 | -2.62 | 6.65E-04 | -1.06 | -2.09 | 2.33E-03 | Uqcrb | ubiquinol-cytochrome c reductase binding protein |
| ENSMUSG00000024474 | -1.16 | -2.23 | 1.12E-04 | -1.07 | -2.09 | 2.53E-05 | Ik | IK cytokine |
| ENSMUSG00000021908 | -1.23 | -2.34 | 9.86E-06 | -1.06 | -2.08 | 5.07E-06 | NA | NA |
| ENSMUSG00000036371 | -1.20 | -2.30 | 2.57E-04 | -1.05 | -2.07 | 1.71E-04 | Serbp1 | serpine1 mRNA binding protein 1 |
| ENSMUSG00000083819 | -1.58 | -3.00 | 8.77E-04 | -1.04 | -2.06 | 5.41E-03 | NA | NA |
| ENSMUSG00000026039 | -1.22 | -2.32 | 9.91E-05 | -1.03 | -2.05 | 2.62E-05 | Sgol2 | shugoshin-like 2 (S. pombe) |
| ENSMUSG00000031245 | -1.36 | -2.57 | 6.08E-04 | -1.02 | -2.03 | 2.74E-03 | Hmgn5 | high-mobility group nucleosome binding domain 5 |
| ENSMUSG00000090124 | -1.20 | -2.29 | 2.26E-03 | -1.02 | -2.03 | 1.79E-03 | Ugt1a7c | UDP glucuronosyltransferase 1 family, polypeptide A7C |
| ENSMUSG00000029246 | -1.24 | -2.36 | 8.57E-10 | -1.01 | -2.01 | 1.55E-10 | Ppat | phosphoribosyl pyrophosphate amidotransferase |
| ENSMUSG00000029623 | -1.05 | -2.07 | 3.84E-04 | -1.01 | -2.01 | 5.35E-05 | Pdap1 | PDGFA associated protein 1 |
| ENSMUSG00000073492 | -1.69 | -3.22 | 3.32E-04 | -1.01 | -2.01 | 5.74E-03 | NA | NA |
| ENSMUSG00000003882 | -1.44 | -2.72 | 4.02E-08 | -1.00 | -2.00 | 4.24E-07 | Il7r | interleukin 7 receptor |
| ENSMUSG00000020330 | -1.07 | -2.10 | 1.15E-04 | -1.00 | -2.00 | 1.75E-05 | Hmmr | hyaluronan mediated motility receptor (RHAMM) |
| ENSMUSG00000020900 | -1.03 | -2.04 | 1.22E-03 | -1.00 | -2.00 | 2.08E-04 | Myh10 | myosin, heavy polypeptide 10, non-muscle |
| ENSMUSG00000028212 | -2.00 | -4.00 | 2.87E-07 | -1.00 | -2.00 | 2.36E-03 | Ccne2 | cyclin E2 |
