# Supplementary material for: Osteolytic cancer cells induce vascular/axon guidance processes in the bone/bone marrow stroma
Source: Oncotarget. 2018 Jun 22;9(48):28877–96. doi: 10.18632/oncotarget.25608 (PMC6034746; doi:10.18632/oncotarget.25608)
Supplement: Supplementary file 5 [file oncotarget-09-28877-s005.docx]

**Table S4.**

| **gene id** | **gene short name** | **description** | **ensembl gene ID** | **human ensembl gene ID** |
| --- | --- | --- | --- | --- |
| ENSG00000234017 | RP11-214N15.5 |  | NA | NA |
| ENSG00000237350 | RP11-800K23.2 |  | NA | NA |
| ENSG00000187866 | FAM122A | family with sequence similarity 122A [Source:HGNC Symbol;Acc:23490] | ENSMUSG00000074922 | ENSG00000187866 |
| ENSG00000207264 | RNU6-15 | RNA, U6 small nuclear 15 [Source:HGNC Symbol;Acc:34259] | NA | NA |
| ENSG00000113140 | SPARC | secreted protein, acidic, cysteine-rich (osteonectin) [Source:HGNC Symbol;Acc:11219] | ENSMUSG00000018593 | ENSG00000113140 |
| ENSG00000201432 | Y_RNA | Y RNA [Source:RFAM;Acc:RF00019] | NA | NA |
| ENSG00000182944 | EWSR1 | Ewing sarcoma breakpoint region 1 [Source:HGNC Symbol;Acc:3508] | ENSMUSG00000009079 | ENSG00000182944 |
| ENSG00000169045 | HNRNPH1 | heterogeneous nuclear ribonucleoprotein H1 (H) [Source:HGNC Symbol;Acc:5041] | ENSMUSG00000007850 | ENSG00000169045 |
| ENSG00000231684 | RP11-416K24.2 |  | NA | NA |
| ENSG00000233137 | RP11-220I1.1 |  | NA | NA |
| ENSG00000132475 | H3F3B | H3 histone, family 3B (H3.3B) [Source:HGNC Symbol;Acc:4765] | ENSMUSG00000016559 | ENSG00000132475 |
| ENSG00000213553 | RPLP0P6 | ribosomal protein, large, P0 pseudogene 6 [Source:HGNC Symbol;Acc:36404] | NA | NA |
| ENSG00000168066 | SF1 | splicing factor 1 [Source:HGNC Symbol;Acc:12950] | ENSMUSG00000024949 | ENSG00000168066 |
| ENSG00000120727 | PAIP2 | poly(A) binding protein interacting protein 2 [Source:HGNC Symbol;Acc:17970] | ENSMUSG00000037058 | ENSG00000120727 |
| ENSG00000055070 | C1orf144 | chromosome 1 open reading frame 144 [Source:HGNC Symbol;Acc:30232] | ENSMUSG00000040842 | ENSG00000055070 |
| ENSG00000233045 | AC097523.1 |  | NA | NA |
| ENSG00000108654 | DDX5 | DEAD (Asp-Glu-Ala-Asp) box polypeptide 5 [Source:HGNC Symbol;Acc:2746] | ENSMUSG00000020719 | ENSG00000108654 |
| ENSG00000112081 | SRSF3 | serine/arginine-rich splicing factor 3 [Source:HGNC Symbol;Acc:10785] | ENSMUSG00000078134 | ENSG00000112081 |
| ENSG00000127824 | TUBA4A | tubulin, alpha 4a [Source:HGNC Symbol;Acc:12407] | ENSMUSG00000026202 | ENSG00000127824 |
| ENSG00000134371 | CDC73 | cell division cycle 73, Paf1/RNA polymerase II complex component, homolog (S. cerevisiae) [Source:HGNC Symbol;Acc:16783] | ENSMUSG00000026361 | ENSG00000134371 |
| ENSG00000164924 | YWHAZ | tyrosine 3-monooxygenase/tryptophan 5-monooxygenase activation protein, zeta polypeptide [Source:HGNC Symbol;Acc:12855] | ENSMUSG00000022285 | ENSG00000164924 |
| ENSG00000085231 | TAF9 | TAF9 RNA polymerase II, TATA box binding protein (TBP)-associated factor, 32kDa [Source:HGNC Symbol;Acc:11542] | ENSMUSG00000052293 | ENSG00000085231 |
| ENSG00000091656 | ZFHX4 | zinc finger homeobox 4 [Source:HGNC Symbol;Acc:30939] | ENSMUSG00000025255 | ENSG00000091656 |
| ENSG00000188529 | SRSF10 | serine/arginine-rich splicing factor 10 [Source:HGNC Symbol;Acc:16713] | NA | NA |
| ENSG00000228169 | PPIAP19 | peptidylprolyl isomerase A (cyclophilin A) pseudogene 19 [Source:HGNC Symbol;Acc:31663] | NA | NA |
| ENSG00000198668 | CALM1 | calmodulin 1 (phosphorylase kinase, delta) [Source:HGNC Symbol;Acc:1442] | NA | NA |
| ENSG00000228285 | LYPLA2P1 | lysophospholipase II pseudogene 1 [Source:HGNC Symbol;Acc:21069] | NA | NA |
| ENSG00000006451 | RALA | v-ral simian leukemia viral oncogene homolog A (ras related) [Source:HGNC Symbol;Acc:9839] | ENSMUSG00000008859 | ENSG00000006451 |
| ENSG00000137747 | TMPRSS13 | transmembrane protease, serine 13 [Source:HGNC Symbol;Acc:29808] | ENSMUSG00000037129 | ENSG00000137747 |
| ENSG00000185670 | ZBTB3 | zinc finger and BTB domain containing 3 [Source:HGNC Symbol;Acc:22918] | ENSMUSG00000071661 | ENSG00000185670 |
| ENSG00000117713 | ARID1A | AT rich interactive domain 1A (SWI-like) [Source:HGNC Symbol;Acc:11110] | ENSMUSG00000007880 | ENSG00000117713 |
| ENSG00000238057 | ZEB2-AS1 | ZEB2 antisense RNA 1 (non-protein coding) [Source:HGNC Symbol;Acc:37149] | NA | NA |
| ENSG00000120705 | ETF1 | eukaryotic translation termination factor 1 [Source:HGNC Symbol;Acc:3477] | ENSMUSG00000024360 | ENSG00000120705 |
| ENSG00000225051 | HMGB3P22 | high mobility group box 3 pseudogene 22 [Source:HGNC Symbol;Acc:39314] | NA | NA |
| ENSG00000172432 | GTPBP2 | GTP binding protein 2 [Source:HGNC Symbol;Acc:4670] | ENSMUSG00000023952 | ENSG00000172432 |
| ENSG00000004534 | RBM6 | RNA binding motif protein 6 [Source:HGNC Symbol;Acc:9903] | ENSMUSG00000032582 | ENSG00000004534 |
| ENSG00000177301 | KCNA2 | potassium voltage-gated channel, shaker-related subfamily, member 2 [Source:HGNC Symbol;Acc:6220] | ENSMUSG00000040724 | ENSG00000177301 |
| ENSG00000163466 | ARPC2 | actin related protein 2/3 complex, subunit 2, 34kDa [Source:HGNC Symbol;Acc:705] | ENSMUSG00000006304 | ENSG00000163466 |
| ENSG00000140406 | MESDC1 | mesoderm development candidate 1 [Source:HGNC Symbol;Acc:13519] | ENSMUSG00000070462 | ENSG00000140406 |
| ENSG00000048828 | FAM120A | family with sequence similarity 120A [Source:HGNC Symbol;Acc:13247] | ENSMUSG00000038014 | ENSG00000048828 |
| ENSG00000067560 | RHOA | ras homolog gene family, member A [Source:HGNC Symbol;Acc:667] | ENSMUSG00000007815 | ENSG00000067560 |
| ENSG00000167978 | SRRM2 | serine/arginine repetitive matrix 2 [Source:HGNC Symbol;Acc:16639] | ENSMUSG00000039218 | ENSG00000167978 |
| ENSG00000239306 | RBM14 | RNA binding motif protein 14 [Source:HGNC Symbol;Acc:14219] | ENSMUSG00000006456 | ENSG00000239306 |
| ENSG00000259133 | RP11-1085N6.3 |  | NA | NA |
| ENSG00000009307 | CSDE1 | cold shock domain containing E1, RNA-binding [Source:HGNC Symbol;Acc:29905] | ENSMUSG00000068823 | ENSG00000009307 |
| ENSG00000169554 | ZEB2 | zinc finger E-box binding homeobox 2 [Source:HGNC Symbol;Acc:14881] | ENSMUSG00000026872 | ENSG00000169554 |
| ENSG00000145416 | 03/01/12 | membrane-associated ring finger (C3HC4) 1 [Source:HGNC Symbol;Acc:26077] | ENSMUSG00000036469 | ENSG00000145416 |
| ENSG00000162734 | PEA15 | phosphoprotein enriched in astrocytes 15 [Source:HGNC Symbol;Acc:8822] | ENSMUSG00000013698 | ENSG00000162734 |
| ENSG00000123144 | C19orf43 | chromosome 19 open reading frame 43 [Source:HGNC Symbol;Acc:28424] | ENSMUSG00000041203 | ENSG00000123144 |
| ENSG00000136451 | VEZF1 | vascular endothelial zinc finger 1 [Source:HGNC Symbol;Acc:12949] | ENSMUSG00000018377 | ENSG00000136451 |
| ENSG00000140092 | FBLN5 | fibulin 5 [Source:HGNC Symbol;Acc:3602] | ENSMUSG00000021186 | ENSG00000140092 |
| ENSG00000180818 | HOXC10 | homeobox C10 [Source:HGNC Symbol;Acc:5122] | ENSMUSG00000022484 | ENSG00000180818 |
| ENSG00000127995 | CASD1 | CAS1 domain containing 1 [Source:HGNC Symbol;Acc:16014] | ENSMUSG00000015189 | ENSG00000127995 |
| ENSG00000119772 | DNMT3A | DNA (cytosine-5-)-methyltransferase 3 alpha [Source:HGNC Symbol;Acc:2978] | ENSMUSG00000020661 | ENSG00000119772 |
| ENSG00000126746 | ZNF384 | zinc finger protein 384 [Source:HGNC Symbol;Acc:11955] | ENSMUSG00000038346 | ENSG00000126746 |
| ENSG00000169057 | MECP2 | methyl CpG binding protein 2 (Rett syndrome) [Source:HGNC Symbol;Acc:6990] | ENSMUSG00000031393 | ENSG00000169057 |
| ENSG00000234337 | AC026462.1 |  | NA | NA |
| ENSG00000100888 | CHD8 | chromodomain helicase DNA binding protein 8 [Source:HGNC Symbol;Acc:20153] | ENSMUSG00000053754 | ENSG00000100888 |
| ENSG00000109846 | CRYAB | crystallin, alpha B [Source:HGNC Symbol;Acc:2389] | ENSMUSG00000032060 | ENSG00000109846 |
| ENSG00000198369 | SPRED2 | sprouty-related, EVH1 domain containing 2 [Source:HGNC Symbol;Acc:17722] | ENSMUSG00000045671 | ENSG00000198369 |
| ENSG00000162923 | WDR26 | WD repeat domain 26 [Source:HGNC Symbol;Acc:21208] | ENSMUSG00000038733 | ENSG00000162923 |
| ENSG00000114942 | EEF1B2 | eukaryotic translation elongation factor 1 beta 2 [Source:HGNC Symbol;Acc:3208] | ENSMUSG00000025967 | ENSG00000114942 |
| ENSG00000008441 | NFIX | nuclear factor I/X (CCAAT-binding transcription factor) [Source:HGNC Symbol;Acc:7788] | ENSMUSG00000001911 | ENSG00000008441 |
| ENSG00000177820 | AC004917.1 |  | NA | NA |
| ENSG00000119042 | SATB2 | SATB homeobox 2 [Source:HGNC Symbol;Acc:21637] | ENSMUSG00000038331 | ENSG00000119042 |
| ENSG00000134986 | C5orf13 | chromosome 5 open reading frame 13 [Source:HGNC Symbol;Acc:16834] | ENSMUSG00000042834 | ENSG00000134986 |
| ENSG00000108443 | RPS6KB1 | ribosomal protein S6 kinase, 70kDa, polypeptide 1 [Source:HGNC Symbol;Acc:10436] | ENSMUSG00000020516 | ENSG00000108443 |
| ENSG00000111341 | MGP | matrix Gla protein [Source:HGNC Symbol;Acc:7060] | ENSMUSG00000030218 | ENSG00000111341 |
| ENSG00000110497 | AMBRA1 | autophagy/beclin-1 regulator 1 [Source:HGNC Symbol;Acc:25990] | ENSMUSG00000040506 | ENSG00000110497 |
| ENSG00000126351 | THRA | thyroid hormone receptor, alpha [Source:HGNC Symbol;Acc:11796] | ENSMUSG00000058756 | ENSG00000126351 |
| ENSG00000237433 | AC097639.8 |  | NA | NA |
| ENSG00000130723 | PRRC2B | proline-rich coiled-coil 2B [Source:HGNC Symbol;Acc:28121] | ENSMUSG00000039262 | ENSG00000130723 |
| ENSG00000142676 | RPL11 | ribosomal protein L11 [Source:HGNC Symbol;Acc:10301] | NA | NA |
| ENSG00000118689 | FOXO3 | forkhead box O3 [Source:HGNC Symbol;Acc:3821] | ENSMUSG00000048756 | ENSG00000118689 |
| ENSG00000114098 | ARMC8 | armadillo repeat containing 8 [Source:HGNC Symbol;Acc:24999] | ENSMUSG00000032468 | ENSG00000114098 |
| ENSG00000133858 | ZFC3H1 | zinc finger, C3H1-type containing [Source:HGNC Symbol;Acc:28328] | ENSMUSG00000034163 | ENSG00000133858 |
| ENSG00000120708 | TGFBI | transforming growth factor, beta-induced, 68kDa [Source:HGNC Symbol;Acc:11771] | ENSMUSG00000035493 | ENSG00000120708 |
| ENSG00000125107 | CNOT1 | CCR4-NOT transcription complex, subunit 1 [Source:HGNC Symbol;Acc:7877] | ENSMUSG00000036550 | ENSG00000125107 |
| ENSG00000169446 | MMGT1 | membrane magnesium transporter 1 [Source:HGNC Symbol;Acc:28100] | ENSMUSG00000061273 | ENSG00000169446 |
| ENSG00000130402 | ACTN4 | actinin, alpha 4 [Source:HGNC Symbol;Acc:166] | ENSMUSG00000054808 | ENSG00000130402 |
| ENSG00000147130 | ZMYM3 | zinc finger, MYM-type 3 [Source:HGNC Symbol;Acc:13054] | ENSMUSG00000031310 | ENSG00000147130 |
| ENSG00000177732 | SOX12 | SRY (sex determining region Y)-box 12 [Source:HGNC Symbol;Acc:11198] | ENSMUSG00000051817 | ENSG00000177732 |
| ENSG00000076108 | BAZ2A | bromodomain adjacent to zinc finger domain, 2A [Source:HGNC Symbol;Acc:962] | ENSMUSG00000040054 | ENSG00000076108 |
| ENSG00000109685 | WHSC1 | Wolf-Hirschhorn syndrome candidate 1 [Source:HGNC Symbol;Acc:12766] | ENSMUSG00000057406 | ENSG00000109685 |
| ENSG00000100462 | PRMT5 | protein arginine methyltransferase 5 [Source:HGNC Symbol;Acc:10894] | ENSMUSG00000023110 | ENSG00000100462 |
| ENSG00000119596 | YLPM1 | YLP motif containing 1 [Source:HGNC Symbol;Acc:17798] | ENSMUSG00000021244 | ENSG00000119596 |
| ENSG00000183779 | ZNF703 | zinc finger protein 703 [Source:HGNC Symbol;Acc:25883] | ENSMUSG00000085795 | ENSG00000183779 |
| ENSG00000163412 | EIF4E3 | eukaryotic translation initiation factor 4E family member 3 [Source:HGNC Symbol;Acc:31837] | ENSMUSG00000030068 | ENSG00000163412 |
| ENSG00000112983 | BRD8 | bromodomain containing 8 [Source:HGNC Symbol;Acc:19874] | ENSMUSG00000003778 | ENSG00000112983 |
| ENSG00000102974 | CTCF | CCCTC-binding factor (zinc finger protein) [Source:HGNC Symbol;Acc:13723] | ENSMUSG00000005698 | ENSG00000102974 |
| ENSG00000164944 | KIAA1429 | KIAA1429 [Source:HGNC Symbol;Acc:24500] | ENSMUSG00000040720 | ENSG00000164944 |
| ENSG00000218227 | RP11-889L3.1 |  | NA | NA |
| ENSG00000143106 | PSMA5 | proteasome (prosome, macropain) subunit, alpha type, 5 [Source:HGNC Symbol;Acc:9534] | NA | NA |
| ENSG00000223973 | AC068491.3 |  | NA | NA |
| ENSG00000077782 | FGFR1 | fibroblast growth factor receptor 1 [Source:HGNC Symbol;Acc:3688] | ENSMUSG00000031565 | ENSG00000077782 |
| ENSG00000185591 | SP1 | Sp1 transcription factor [Source:HGNC Symbol;Acc:11205] | ENSMUSG00000001280 | ENSG00000185591 |
| ENSG00000143952 | VPS54 | vacuolar protein sorting 54 homolog (S. cerevisiae) [Source:HGNC Symbol;Acc:18652] | ENSMUSG00000020128 | ENSG00000143952 |
| ENSG00000233247 | GS1-257G1.1 |  | NA | NA |
| ENSG00000143761 | ARF1 | ADP-ribosylation factor 1 [Source:HGNC Symbol;Acc:652] | ENSMUSG00000048076 | ENSG00000143761 |
| ENSG00000067369 | TP53BP1 | tumor protein p53 binding protein 1 [Source:HGNC Symbol;Acc:11999] | ENSMUSG00000043909 | ENSG00000067369 |
| ENSG00000126457 | PRMT1 | protein arginine methyltransferase 1 [Source:HGNC Symbol;Acc:5187] | ENSMUSG00000052429 | ENSG00000126457 |
| ENSG00000196428 | TSC22D2 | TSC22 domain family, member 2 [Source:HGNC Symbol;Acc:29095] | ENSMUSG00000027806 | ENSG00000196428 |
| ENSG00000157514 | TSC22D3 | TSC22 domain family, member 3 [Source:HGNC Symbol;Acc:3051] | ENSMUSG00000031431 | ENSG00000157514 |
| ENSG00000120458 | C11orf61 | chromosome 11 open reading frame 61 [Source:HGNC Symbol;Acc:26266] | ENSMUSG00000042138 | ENSG00000120458 |
| ENSG00000139620 | C12orf41 | chromosome 12 open reading frame 41 [Source:HGNC Symbol;Acc:26024] | ENSMUSG00000022992 | ENSG00000139620 |
| ENSG00000173153 | ESRRA | estrogen-related receptor alpha [Source:HGNC Symbol;Acc:3471] | ENSMUSG00000024955 | ENSG00000173153 |
| ENSG00000167193 | CRK | v-crk sarcoma virus CT10 oncogene homolog (avian) [Source:HGNC Symbol;Acc:2362] | ENSMUSG00000017776 | ENSG00000167193 |
| ENSG00000104904 | OAZ1 | ornithine decarboxylase antizyme 1 [Source:HGNC Symbol;Acc:8095] | ENSMUSG00000035242 | ENSG00000104904 |
| ENSG00000155511 | GRIA1 | glutamate receptor, ionotropic, AMPA 1 [Source:HGNC Symbol;Acc:4571] | ENSMUSG00000020524 | ENSG00000155511 |
| ENSG00000126705 | AHDC1 | AT hook, DNA binding motif, containing 1 [Source:HGNC Symbol;Acc:25230] | ENSMUSG00000037692 | ENSG00000126705 |
| ENSG00000198925 | ATG9A | ATG9 autophagy related 9 homolog A (S. cerevisiae) [Source:HGNC Symbol;Acc:22408] | ENSMUSG00000033124 | ENSG00000198925 |
| ENSG00000128708 | HAT1 | histone acetyltransferase 1 [Source:HGNC Symbol;Acc:4821] | ENSMUSG00000027018 | ENSG00000128708 |
| ENSG00000110047 | EHD1 | EH-domain containing 1 [Source:HGNC Symbol;Acc:3242] | ENSMUSG00000024772 | ENSG00000110047 |
| ENSG00000115966 | ATF2 | activating transcription factor 2 [Source:HGNC Symbol;Acc:784] | ENSMUSG00000027104 | ENSG00000115966 |
| ENSG00000140367 | UBE2Q2 | ubiquitin-conjugating enzyme E2Q family member 2 [Source:HGNC Symbol;Acc:19248] | ENSMUSG00000045291 | ENSG00000140367 |
| ENSG00000166398 | KIAA0355 | KIAA0355 [Source:HGNC Symbol;Acc:29016] | ENSMUSG00000066571 | ENSG00000166398 |
| ENSG00000214655 | KIAA0913 | KIAA0913 [Source:HGNC Symbol;Acc:23528] | ENSMUSG00000021819 | ENSG00000214655 |
| ENSG00000154582 | TCEB1 | transcription elongation factor B (SIII), polypeptide 1 (15kDa, elongin C) [Source:HGNC Symbol;Acc:11617] | ENSMUSG00000079658 | ENSG00000154582 |
| ENSG00000139990 | DCAF5 | DDB1 and CUL4 associated factor 5 [Source:HGNC Symbol;Acc:20224] | ENSMUSG00000049106 | ENSG00000139990 |
| ENSG00000100105 | PATZ1 | POZ (BTB) and AT hook containing zinc finger 1 [Source:HGNC Symbol;Acc:13071] | ENSMUSG00000020453 | ENSG00000100105 |
| ENSG00000125352 | RNF113A | ring finger protein 113A [Source:HGNC Symbol;Acc:12974] | ENSMUSG00000036537 | ENSG00000125352 |
| ENSG00000123685 | BATF3 | basic leucine zipper transcription factor, ATF-like 3 [Source:HGNC Symbol;Acc:28915] | ENSMUSG00000026630 | ENSG00000123685 |
| ENSG00000099385 | BCL7C | B-cell CLL/lymphoma 7C [Source:HGNC Symbol;Acc:1006] | ENSMUSG00000030814 | ENSG00000099385 |
| ENSG00000105996 | HOXA2 | homeobox A2 [Source:HGNC Symbol;Acc:5103] | ENSMUSG00000014704 | ENSG00000105996 |
| ENSG00000067048 | DDX3Y | DEAD (Asp-Glu-Ala-Asp) box polypeptide 3, Y-linked [Source:HGNC Symbol;Acc:2699] | ENSMUSG00000069045 | ENSG00000067048 |
| ENSG00000167258 | CDK12 | cyclin-dependent kinase 12 [Source:HGNC Symbol;Acc:24224] | ENSMUSG00000003119 | ENSG00000167258 |
| ENSG00000224861 | YBX1P1 | Y box binding protein 1 pseudogene 1 [Source:HGNC Symbol;Acc:8015] | NA | NA |
| ENSG00000175826 | CTDNEP1 | CTD nuclear envelope phosphatase 1 [Source:HGNC Symbol;Acc:19085] | ENSMUSG00000018559 | ENSG00000175826 |
| ENSG00000236370 | RP11-574K11.16 |  | NA | NA |
| ENSG00000115414 | FN1 | fibronectin 1 [Source:HGNC Symbol;Acc:3778] | ENSMUSG00000026193 | ENSG00000115414 |
| ENSG00000250182 | CTD-2165H16.1 |  | NA | NA |
| ENSG00000237493 | RP11-603J24.7 |  | NA | NA |
| ENSG00000259102 | CTD-2552B11.2 |  | NA | NA |
| ENSG00000136802 | LRRC8A | leucine rich repeat containing 8 family, member A [Source:HGNC Symbol;Acc:19027] | ENSMUSG00000007476 | ENSG00000136802 |
| ENSG00000055130 | CUL1 | cullin 1 [Source:HGNC Symbol;Acc:2551] | ENSMUSG00000029686 | ENSG00000055130 |
| ENSG00000168658 | VWA3B | von Willebrand factor A domain containing 3B [Source:HGNC Symbol;Acc:28385] | ENSMUSG00000050122 | ENSG00000168658 |
| ENSG00000179933 | C14orf119 | chromosome 14 open reading frame 119 [Source:HGNC Symbol;Acc:20270] | ENSMUSG00000040822 | ENSG00000179933 |
| ENSG00000087085 | ACHE | acetylcholinesterase [Source:HGNC Symbol;Acc:108] | ENSMUSG00000023328 | ENSG00000087085 |
| ENSG00000188483 | IER5L | immediate early response 5-like [Source:HGNC Symbol;Acc:23679] | ENSMUSG00000089762 | ENSG00000188483 |
| ENSG00000075624 | ACTB | actin, beta [Source:HGNC Symbol;Acc:132] | ENSMUSG00000029580 | ENSG00000075624 |
| ENSG00000161180 | CCDC116 | coiled-coil domain containing 116 [Source:HGNC Symbol;Acc:26688] | ENSMUSG00000022768 | ENSG00000161180 |
| ENSG00000105672 | ETV2 | ets variant 2 [Source:HGNC Symbol;Acc:3491] | ENSMUSG00000006311 | ENSG00000105672 |
| ENSG00000110911 | SLC11A2 | solute carrier family 11 (proton-coupled divalent metal ion transporters), member 2 [Source:HGNC Symbol;Acc:10908] | ENSMUSG00000023030 | ENSG00000110911 |
| ENSG00000136504 | KAT7 | K(lysine) acetyltransferase 7 [Source:HGNC Symbol;Acc:17016] | ENSMUSG00000038909 | ENSG00000136504 |
| ENSG00000175592 | FOSL1 | FOS-like antigen 1 [Source:HGNC Symbol;Acc:13718] | ENSMUSG00000024912 | ENSG00000175592 |
| ENSG00000238109 | AC004893.10 |  | NA | NA |
| ENSG00000115942 | ORC2 | origin recognition complex, subunit 2 [Source:HGNC Symbol;Acc:8488] | ENSMUSG00000026037 | ENSG00000115942 |
| ENSG00000144567 | FAM134A | family with sequence similarity 134, member A [Source:HGNC Symbol;Acc:28450] | ENSMUSG00000049339 | ENSG00000144567 |
| ENSG00000139318 | DUSP6 | dual specificity phosphatase 6 [Source:HGNC Symbol;Acc:3072] | ENSMUSG00000019960 | ENSG00000139318 |
| ENSG00000167797 | CDK2AP2 | cyclin-dependent kinase 2 associated protein 2 [Source:HGNC Symbol;Acc:30833] | ENSMUSG00000024856 | ENSG00000167797 |
| ENSG00000092964 | DPYSL2 | dihydropyrimidinase-like 2 [Source:HGNC Symbol;Acc:3014] | ENSMUSG00000022048 | ENSG00000092964 |
| ENSG00000188042 | ARL4C | ADP-ribosylation factor-like 4C [Source:HGNC Symbol;Acc:698] | ENSMUSG00000049866 | ENSG00000188042 |
| ENSG00000107581 | EIF3A | eukaryotic translation initiation factor 3, subunit A [Source:HGNC Symbol;Acc:3271] | ENSMUSG00000024991 | ENSG00000107581 |
| ENSG00000128606 | LRRC17 | leucine rich repeat containing 17 [Source:HGNC Symbol;Acc:16895] | ENSMUSG00000039883 | ENSG00000128606 |
| ENSG00000257103 | LSM14A | LSM14A, SCD6 homolog A (S. cerevisiae) [Source:HGNC Symbol;Acc:24489] | ENSMUSG00000066568 | ENSG00000257103 |
| ENSG00000135932 | CAB39 | calcium binding protein 39 [Source:HGNC Symbol;Acc:20292] | ENSMUSG00000036707 | ENSG00000135932 |
| ENSG00000113441 | LNPEP | leucyl/cystinyl aminopeptidase [Source:HGNC Symbol;Acc:6656] | ENSMUSG00000023845 | ENSG00000113441 |
| ENSG00000170265 | ZNF282 | zinc finger protein 282 [Source:HGNC Symbol;Acc:13076] | ENSMUSG00000025821 | ENSG00000170265 |
| ENSG00000163939 | PBRM1 | polybromo 1 [Source:HGNC Symbol;Acc:30064] | ENSMUSG00000042323 | ENSG00000163939 |
| ENSG00000243746 | EEF1A1P10 | eukaryotic translation elongation factor 1 alpha 1 pseudogene 10 [Source:HGNC Symbol;Acc:3205] | NA | NA |
| ENSG00000197635 | DPP4 | dipeptidyl-peptidase 4 [Source:HGNC Symbol;Acc:3009] | ENSMUSG00000035000 | ENSG00000197635 |
| ENSG00000223647 | AL133249.1 |  | NA | NA |
| ENSG00000144136 | SLC20A1 | solute carrier family 20 (phosphate transporter), member 1 [Source:HGNC Symbol;Acc:10946] | ENSMUSG00000027397 | ENSG00000144136 |
| ENSG00000250492 | CTD-2120M21.1 |  | NA | NA |
| ENSG00000164080 | RAD54L2 | RAD54-like 2 (S. cerevisiae) [Source:HGNC Symbol;Acc:29123] | ENSMUSG00000040661 | ENSG00000164080 |
| ENSG00000220157 | HNRNPA1P12 | heterogeneous nuclear ribonucleoprotein A1 pseudogene 12 [Source:HGNC Symbol;Acc:39130] | NA | NA |
| ENSG00000117394 | SLC2A1 | solute carrier family 2 (facilitated glucose transporter), member 1 [Source:HGNC Symbol;Acc:11005] | ENSMUSG00000028645 | ENSG00000117394 |
| ENSG00000151532 | VTI1A | vesicle transport through interaction with t-SNAREs homolog 1A (yeast) [Source:HGNC Symbol;Acc:17792] | ENSMUSG00000024983 | ENSG00000151532 |
| ENSG00000158966 | CACHD1 | cache domain containing 1 [Source:HGNC Symbol;Acc:29314] | ENSMUSG00000028532 | ENSG00000158966 |
| ENSG00000125347 | IRF1 | interferon regulatory factor 1 [Source:HGNC Symbol;Acc:6116] | ENSMUSG00000018899 | ENSG00000125347 |
| ENSG00000197208 | SLC22A4 | solute carrier family 22 (organic cation/ergothioneine transporter), member 4 [Source:HGNC Symbol;Acc:10968] | ENSMUSG00000020334 | ENSG00000197208 |
| ENSG00000104221 | BRF2 | BRF2, subunit of RNA polymerase III transcription initiation factor, BRF1-like [Source:HGNC Symbol;Acc:17298] | ENSMUSG00000031487 | ENSG00000104221 |
| ENSG00000101447 | FAM83D | family with sequence similarity 83, member D [Source:HGNC Symbol;Acc:16122] | ENSMUSG00000027654 | ENSG00000101447 |
| ENSG00000141905 | NFIC | nuclear factor I/C (CCAAT-binding transcription factor) [Source:HGNC Symbol;Acc:7786] | ENSMUSG00000055053 | ENSG00000141905 |
| ENSG00000106128 | GHRHR | growth hormone releasing hormone receptor [Source:HGNC Symbol;Acc:4266] | ENSMUSG00000004654 | ENSG00000106128 |
| ENSG00000167196 | FBXO22 | F-box protein 22 [Source:HGNC Symbol;Acc:13593] | ENSMUSG00000032309 | ENSG00000167196 |
| ENSG00000251667 | RP11-844P9.3 |  | NA | NA |
| ENSG00000134318 | ROCK2 | Rho-associated, coiled-coil containing protein kinase 2 [Source:HGNC Symbol;Acc:10252] | ENSMUSG00000020580 | ENSG00000134318 |
| ENSG00000163902 | RPN1 | ribophorin I [Source:HGNC Symbol;Acc:10381] | ENSMUSG00000030062 | ENSG00000163902 |
| ENSG00000093167 | LRRFIP2 | leucine rich repeat (in FLII) interacting protein 2 [Source:HGNC Symbol;Acc:6703] | ENSMUSG00000032497 | ENSG00000093167 |
| ENSG00000168286 | THAP11 | THAP domain containing 11 [Source:HGNC Symbol;Acc:23194] | ENSMUSG00000036442 | ENSG00000168286 |
| ENSG00000126602 | TRAP1 | TNF receptor-associated protein 1 [Source:HGNC Symbol;Acc:16264] | ENSMUSG00000005981 | ENSG00000126602 |
| ENSG00000254598 | CSNK2A1P | casein kinase 2, alpha 1 polypeptide pseudogene [Source:HGNC Symbol;Acc:2458] | NA | NA |
| ENSG00000078043 | PIAS2 | protein inhibitor of activated STAT, 2 [Source:HGNC Symbol;Acc:17311] | ENSMUSG00000025423 | ENSG00000078043 |
| ENSG00000119906 | FAM178A | family with sequence similarity 178, member A [Source:HGNC Symbol;Acc:17814] | ENSMUSG00000036097 | ENSG00000119906 |
| ENSG00000111237 | VPS29 | vacuolar protein sorting 29 homolog (S. cerevisiae) [Source:HGNC Symbol;Acc:14340] | ENSMUSG00000029462 | ENSG00000111237 |
| ENSG00000258972 | NDUFB8P1 | NADH dehydrogenase (ubiquinone) 1 beta subcomplex, 8, pseudogene 1 [Source:HGNC Symbol;Acc:20023] | NA | NA |
| ENSG00000091527 | CDV3 | CDV3 homolog (mouse) [Source:HGNC Symbol;Acc:26928] | ENSMUSG00000032803 | ENSG00000091527 |
| ENSG00000197063 | MAFG | v-maf musculoaponeurotic fibrosarcoma oncogene homolog G (avian) [Source:HGNC Symbol;Acc:6781] | ENSMUSG00000051510 | ENSG00000197063 |
| ENSG00000164309 | CMYA5 | cardiomyopathy associated 5 [Source:HGNC Symbol;Acc:14305] | ENSMUSG00000047419 | ENSG00000164309 |
| ENSG00000164692 | COL1A2 | collagen, type I, alpha 2 [Source:HGNC Symbol;Acc:2198] | ENSMUSG00000029661 | ENSG00000164692 |
| ENSG00000186642 | PDE2A | phosphodiesterase 2A, cGMP-stimulated [Source:HGNC Symbol;Acc:8777] | ENSMUSG00000030653 | ENSG00000186642 |
| ENSG00000010803 | SCMH1 | sex comb on midleg homolog 1 (Drosophila) [Source:HGNC Symbol;Acc:19003] | ENSMUSG00000000085 | ENSG00000010803 |
| ENSG00000101605 | MYOM1 | myomesin 1, 185kDa [Source:HGNC Symbol;Acc:7613] | ENSMUSG00000024049 | ENSG00000101605 |
| ENSG00000108518 | PFN1 | profilin 1 [Source:HGNC Symbol;Acc:8881] | ENSMUSG00000018293 | ENSG00000108518 |
| ENSG00000084693 | AGBL5 | ATP/GTP binding protein-like 5 [Source:HGNC Symbol;Acc:26147] | ENSMUSG00000029165 | ENSG00000084693 |
| ENSG00000120656 | TAF12 | TAF12 RNA polymerase II, TATA box binding protein (TBP)-associated factor, 20kDa [Source:HGNC Symbol;Acc:11545] | ENSMUSG00000028899 | ENSG00000120656 |
| ENSG00000182541 | LIMK2 | LIM domain kinase 2 [Source:HGNC Symbol;Acc:6614] | ENSMUSG00000020451 | ENSG00000182541 |
| ENSG00000033050 | ABCF2 | ATP-binding cassette, sub-family F (GCN20), member 2 [Source:HGNC Symbol;Acc:71] | ENSMUSG00000028953 | ENSG00000033050 |
| ENSG00000101911 | PRPS2 | phosphoribosyl pyrophosphate synthetase 2 [Source:HGNC Symbol;Acc:9465] | ENSMUSG00000025742 | ENSG00000101911 |
| ENSG00000227164 | AL354993.1 | Cell growth-inhibiting protein 7HCG1784586Uncharacterized protein [Source:UniProtKB/TrEMBL;Acc:B1H0U8] | NA | NA |
| ENSG00000131242 | RAB11FIP4 | RAB11 family interacting protein 4 (class II) [Source:HGNC Symbol;Acc:30267] | ENSMUSG00000017639 | ENSG00000131242 |
| ENSG00000237285 | HNRNPA1P2 | heterogeneous nuclear ribonucleoprotein A1 pseudogene 2 [Source:HGNC Symbol;Acc:13958] | NA | NA |
| ENSG00000182872 | RBM10 | RNA binding motif protein 10 [Source:HGNC Symbol;Acc:9896] | ENSMUSG00000031060 | ENSG00000182872 |
| ENSG00000017427 | IGF1 | insulin-like growth factor 1 (somatomedin C) [Source:HGNC Symbol;Acc:5464] | ENSMUSG00000020053 | ENSG00000017427 |
| ENSG00000136848 | DAB2IP | DAB2 interacting protein [Source:HGNC Symbol;Acc:17294] | ENSMUSG00000026883 | ENSG00000136848 |
| ENSG00000255773 | RP11-566K11.1 |  | NA | NA |
| ENSG00000183337 | BCOR | BCL6 corepressor [Source:HGNC Symbol;Acc:20893] | ENSMUSG00000040363 | ENSG00000183337 |
| ENSG00000231381 | RNF2P1 | ring finger protein 2 pseudogene 1 [Source:HGNC Symbol;Acc:33987] | NA | NA |
| ENSG00000168765 | GSTM4 | glutathione S-transferase mu 4 [Source:HGNC Symbol;Acc:4636] | ENSMUSG00000027890 | ENSG00000168765 |
| ENSG00000091157 | WDR7 | WD repeat domain 7 [Source:HGNC Symbol;Acc:13490] | ENSMUSG00000040560 | ENSG00000091157 |
| ENSG00000089048 | ESF1 | ESF1, nucleolar pre-rRNA processing protein, homolog (S. cerevisiae) [Source:HGNC Symbol;Acc:15898] | ENSMUSG00000045624 | ENSG00000089048 |
| ENSG00000214653 | HNRNPA3P3 | heterogeneous nuclear ribonucleoprotein A3 pseudogene 3 [Source:HGNC Symbol;Acc:39772] | NA | NA |
| ENSG00000129911 | KLF16 | Kruppel-like factor 16 [Source:HGNC Symbol;Acc:16857] | ENSMUSG00000035397 | ENSG00000129911 |
| ENSG00000052841 | TTC17 | tetratricopeptide repeat domain 17 [Source:HGNC Symbol;Acc:25596] | ENSMUSG00000027194 | ENSG00000052841 |
| ENSG00000180694 | TMEM64 | transmembrane protein 64 [Source:HGNC Symbol;Acc:25441] | ENSMUSG00000043252 | ENSG00000180694 |
| ENSG00000188693 | CTB-161K23.1 |  | NA | NA |
| ENSG00000140326 | CDAN1 | congenital dyserythropoietic anemia, type I [Source:HGNC Symbol;Acc:1713] | ENSMUSG00000027284 | ENSG00000140326 |
| ENSG00000114405 | C3orf14 | chromosome 3 open reading frame 14 [Source:HGNC Symbol;Acc:25024] | ENSMUSG00000033111 | ENSG00000114405 |
| ENSG00000044459 | CNTLN | centlein, centrosomal protein [Source:HGNC Symbol;Acc:23432] | ENSMUSG00000038070 | ENSG00000044459 |
| ENSG00000213420 | GPC2 | glypican 2 [Source:HGNC Symbol;Acc:4450] | ENSMUSG00000029510 | ENSG00000213420 |
| ENSG00000131263 | RLIM | ring finger protein, LIM domain interacting [Source:HGNC Symbol;Acc:13429] | ENSMUSG00000056537 | ENSG00000131263 |
| ENSG00000069329 | VPS35 | vacuolar protein sorting 35 homolog (S. cerevisiae) [Source:HGNC Symbol;Acc:13487] | ENSMUSG00000031696 | ENSG00000069329 |
| ENSG00000215895 | RP11-334L9.1 |  | NA | NA |
| ENSG00000106819 | ASPN | asporin [Source:HGNC Symbol;Acc:14872] | ENSMUSG00000021388 | ENSG00000106819 |
| ENSG00000197724 | PHF2 | PHD finger protein 2 [Source:HGNC Symbol;Acc:8920] | ENSMUSG00000038025 | ENSG00000197724 |
| ENSG00000242265 | PEG10 | paternally expressed 10 [Source:HGNC Symbol;Acc:14005] | NA | NA |
| ENSG00000178075 | GRAMD1C | GRAM domain containing 1C [Source:HGNC Symbol;Acc:25252] | ENSMUSG00000036292 | ENSG00000178075 |
| ENSG00000187091 | PLCD1 | phospholipase C, delta 1 [Source:HGNC Symbol;Acc:9060] | ENSMUSG00000010660 | ENSG00000187091 |
| ENSG00000184574 | LPAR5 | lysophosphatidic acid receptor 5 [Source:HGNC Symbol;Acc:13307] | ENSMUSG00000067714 | ENSG00000184574 |
| ENSG00000127083 | OMD | osteomodulin [Source:HGNC Symbol;Acc:8134] | ENSMUSG00000048368 | ENSG00000127083 |
| ENSG00000103994 | ZFP106 | zinc finger protein 106 homolog (mouse) [Source:HGNC Symbol;Acc:23240] | ENSMUSG00000027288 | ENSG00000103994 |
| ENSG00000158545 | ZC3H18 | zinc finger CCCH-type containing 18 [Source:HGNC Symbol;Acc:25091] | ENSMUSG00000017478 | ENSG00000158545 |
| ENSG00000179930 | ZNF648 | zinc finger protein 648 [Source:HGNC Symbol;Acc:18190] | ENSMUSG00000066797 | ENSG00000179930 |
| ENSG00000227391 | SALL1P1 | sal-like 1 (Drosophila) pseudogene 1 [Source:HGNC Symbol;Acc:10525] | NA | NA |
| ENSG00000185085 | INTS5 | integrator complex subunit 5 [Source:HGNC Symbol;Acc:29352] | ENSMUSG00000071652 | ENSG00000185085 |
| ENSG00000148814 | LRRC27 | leucine rich repeat containing 27 [Source:HGNC Symbol;Acc:29346] | ENSMUSG00000015980 | ENSG00000148814 |
| ENSG00000149573 | MPZL2 | myelin protein zero-like 2 [Source:HGNC Symbol;Acc:3496] | ENSMUSG00000032092 | ENSG00000149573 |
| ENSG00000163873 | GRIK3 | glutamate receptor, ionotropic, kainate 3 [Source:HGNC Symbol;Acc:4581] | ENSMUSG00000001985 | ENSG00000163873 |
| ENSG00000251347 | IRF5P1 | interferon regulatory factor 5 pseudogene 1 [Source:HGNC Symbol;Acc:41981] | NA | NA |
| ENSG00000108511 | HOXB6 | homeobox B6 [Source:HGNC Symbol;Acc:5117] | ENSMUSG00000000690 | ENSG00000108511 |
| ENSG00000139044 | B4GALNT3 | beta-1,4-N-acetyl-galactosaminyl transferase 3 [Source:HGNC Symbol;Acc:24137] | ENSMUSG00000041372 | ENSG00000139044 |
| ENSG00000186448 | ZNF197 | zinc finger protein 197 [Source:HGNC Symbol;Acc:12988] | NA | NA |
| ENSG00000173227 | SYT12 | synaptotagmin XII [Source:HGNC Symbol;Acc:18381] | ENSMUSG00000049303 | ENSG00000173227 |
| ENSG00000155275 | METTL19 | methyltransferase like 19 [Source:HGNC Symbol;Acc:26653] | ENSMUSG00000029097 | ENSG00000155275 |
