# Supplementary material for: Osteolytic cancer cells induce vascular/axon guidance processes in the bone/bone marrow stroma
Source: Oncotarget. 2018 Jun 22;9(48):28877–96. doi: 10.18632/oncotarget.25608 (PMC6034746; doi:10.18632/oncotarget.25608)
Supplement: Supplementary file 6 [file oncotarget-09-28877-s006.docx]

**Table S5.**

| id | log2FoldChange.y | FC | padj | SYMBOL | GENENAME |
| --- | --- | --- | --- | --- | --- |
| ENSMUSG00000049723 | 7.33 | 160.40 | 3.20E-40 | Mmp12 | matrix metallopeptidase 12 |
| ENSMUSG00000029816 | 7.19 | 145.57 | 1.98E-93 | Gpnmb | glycoprotein (transmembrane) nmb |
| ENSMUSG00000084893 | 3.39 | 10.48 | 1.56E-05 | NA | NA |
| ENSMUSG00000043613 | 3.24 | 9.43 | 6.29E-07 | Mmp3 | matrix metallopeptidase 3 |
| ENSMUSG00000003545 | 3.15 | 8.86 | 4.60E-05 | Fosb | FBJ osteosarcoma oncogene B |
| ENSMUSG00000058914 | 3.09 | 8.49 | 2.32E-09 | C1qtnf3 | C1q and tumor necrosis factor related protein 3 |
| ENSMUSG00000042254 | 2.92 | 7.56 | 3.29E-08 | Cilp | cartilage intermediate layer protein, nucleotide pyrophosphohydrolase |
| ENSMUSG00000006014 | 2.83 | 7.12 | 1.48E-03 | Prg4 | proteoglycan 4 (megakaryocyte stimulating factor, articular superficial zone protein) |
| ENSMUSG00000059412 | 2.79 | 6.94 | 4.26E-03 | Fxyd2 | FXYD domain-containing ion transport regulator 2 |
| ENSMUSG00000025776 | 2.76 | 6.77 | 6.37E-05 | Crispld1 | cysteine-rich secretory protein LCCL domain containing 1 |
| ENSMUSG00000075047 | 2.75 | 6.74 | 1.41E-05 | NA | NA |
| ENSMUSG00000085128 | 2.69 | 6.46 | 1.85E-03 | NA | NA |
| ENSMUSG00000031849 | 2.65 | 6.28 | 5.85E-04 | Comp | cartilage oligomeric matrix protein |
| ENSMUSG00000032487 | 2.41 | 5.30 | 2.66E-03 | Ptgs2 | prostaglandin-endoperoxide synthase 2 |
| ENSMUSG00000037362 | 2.35 | 5.11 | 6.09E-04 | Nov | nephroblastoma overexpressed gene |
| ENSMUSG00000019932 | 2.27 | 4.82 | 5.85E-04 | Kera | keratocan |
| ENSMUSG00000041577 | 2.21 | 4.63 | 1.56E-06 | Prelp | proline arginine-rich end leucine-rich repeat |
| ENSMUSG00000043122 | 2.14 | 4.40 | 3.99E-03 | A530016L24Rik | RIKEN cDNA A530016L24 gene |
| ENSMUSG00000062083 | 2.13 | 4.38 | 2.94E-04 | NA | NA |
| ENSMUSG00000021950 | 2.12 | 4.35 | 1.07E-05 | Anxa8 | annexin A8 |
| ENSMUSG00000086596 | 2.11 | 4.31 | 1.72E-04 | Susd5 | sushi domain containing 5 |
| ENSMUSG00000027996 | 2.04 | 4.11 | 3.13E-03 | Sfrp2 | secreted frizzled-related protein 2 |
| ENSMUSG00000057751 | 1.98 | 3.96 | 5.83E-04 | Megf6 | multiple EGF-like-domains 6 |
| ENSMUSG00000026051 | 1.97 | 3.92 | 2.42E-04 | 1500015O10Rik | RIKEN cDNA 1500015O10 gene |
| ENSMUSG00000027386 | 1.96 | 3.89 | 3.93E-04 | Fbln7 | fibulin 7 |
| ENSMUSG00000020467 | 1.95 | 3.86 | 5.43E-04 | Efemp1 | epidermal growth factor-containing fibulin-like extracellular matrix protein 1 |
| ENSMUSG00000020363 | 1.94 | 3.84 | 1.41E-05 | Gfpt2 | glutamine fructose-6-phosphate transaminase 2 |
| ENSMUSG00000041559 | 1.92 | 3.78 | 1.00E-06 | Fmod | fibromodulin |
| ENSMUSG00000072941 | 1.92 | 3.77 | 8.48E-04 | Sod3 | superoxide dismutase 3, extracellular |
| ENSMUSG00000039062 | 1.90 | 3.73 | 2.91E-08 | Anpep | alanyl (membrane) aminopeptidase |
| ENSMUSG00000037868 | 1.86 | 3.63 | 1.10E-03 | Egr2 | early growth response 2 |
| ENSMUSG00000039691 | 1.81 | 3.52 | 3.13E-03 | Tspan10 | tetraspanin 10 |
| ENSMUSG00000030789 | 1.76 | 3.39 | 5.22E-12 | Itgax | integrin alpha X |
| ENSMUSG00000037411 | 1.76 | 3.38 | 4.68E-03 | Serpine1 | serine (or cysteine) peptidase inhibitor, clade E, member 1 |
| ENSMUSG00000027859 | 1.73 | 3.33 | 4.36E-03 | Ngf | nerve growth factor |
| ENSMUSG00000017400 | 1.70 | 3.26 | 1.17E-04 | Stac2 | SH3 and cysteine rich domain 2 |
| ENSMUSG00000071984 | 1.69 | 3.23 | 2.59E-03 | Fndc1 | fibronectin type III domain containing 1 |
| ENSMUSG00000084183 | 1.68 | 3.21 | 4.48E-03 | NA | NA |
| ENSMUSG00000015396 | 1.68 | 3.20 | 1.09E-06 | Cd83 | CD83 antigen |
| ENSMUSG00000026697 | 1.62 | 3.07 | 1.66E-04 | Myoc | myocilin |
| ENSMUSG00000072812 | 1.60 | 3.03 | 6.47E-05 | Ahnak2 | AHNAK nucleoprotein 2 |
| ENSMUSG00000045672 | 1.56 | 2.95 | 2.73E-03 | Col27a1 | collagen, type XXVII, alpha 1 |
| ENSMUSG00000028583 | 1.54 | 2.91 | 2.26E-04 | NA | NA |
| ENSMUSG00000028047 | 1.54 | 2.91 | 5.08E-04 | Thbs3 | thrombospondin 3 |
| ENSMUSG00000056174 | 1.51 | 2.85 | 4.49E-03 | Col8a2 | collagen, type VIII, alpha 2 |
| ENSMUSG00000022665 | 1.43 | 2.70 | 1.09E-06 | Ccdc80 | coiled-coil domain containing 80 |
| ENSMUSG00000040552 | 1.38 | 2.60 | 1.27E-04 | C3ar1 | complement component 3a receptor 1 |
| ENSMUSG00000035095 | 1.38 | 2.60 | 1.00E-05 | Fam167a | family with sequence similarity 167, member A |
| ENSMUSG00000021943 | 1.38 | 2.60 | 2.19E-05 | Gdf10 | growth differentiation factor 10 |
| ENSMUSG00000023992 | 1.36 | 2.56 | 4.49E-03 | Trem2 | triggering receptor expressed on myeloid cells 2 |
| ENSMUSG00000075585 | 1.35 | 2.55 | 1.71E-05 | NA | NA |
| ENSMUSG00000028111 | 1.33 | 2.51 | 8.48E-04 | Ctsk | cathepsin K |
| ENSMUSG00000083679 | 1.30 | 2.46 | 6.90E-04 | NA | NA |
| ENSMUSG00000030605 | 1.29 | 2.45 | 6.83E-04 | Mfge8 | milk fat globule-EGF factor 8 protein |
| ENSMUSG00000059824 | 1.29 | 2.44 | 4.68E-03 | Dbp | D site albumin promoter binding protein |
| ENSMUSG00000029581 | 1.26 | 2.40 | 2.39E-04 | Fscn1 | fascin homolog 1, actin bundling protein (Strongylocentrotus purpuratus) |
| ENSMUSG00000044646 | 1.25 | 2.39 | 3.83E-04 | Zbtb7c | zinc finger and BTB domain containing 7C |
| ENSMUSG00000023349 | 1.23 | 2.35 | 1.26E-03 | Clec4n | C-type lectin domain family 4, member n |
| ENSMUSG00000019929 | 1.22 | 2.32 | 6.23E-07 | Dcn | decorin |
| ENSMUSG00000020473 | 1.21 | 2.31 | 3.70E-03 | Aebp1 | AE binding protein 1 |
| ENSMUSG00000001270 | 1.19 | 2.28 | 1.17E-04 | Ckb | creatine kinase, brain |
| ENSMUSG00000045294 | 1.17 | 2.25 | 8.48E-04 | Insig1 | insulin induced gene 1 |
| ENSMUSG00000037624 | 1.12 | 2.17 | 3.62E-04 | Kcnk2 | potassium channel, subfamily K, member 2 |
| ENSMUSG00000056214 | 1.11 | 2.16 | 1.41E-05 | Pard6g | par-6 family cell polarity regulator gamma |
| ENSMUSG00000044337 | 1.09 | 2.13 | 4.32E-03 | Ackr3 | atypical chemokine receptor 3 |
| ENSMUSG00000025854 | 1.05 | 2.07 | 2.39E-04 | Fam20c | family with sequence similarity 20, member C |
| ENSMUSG00000047712 | 1.01 | 2.02 | 6.47E-05 | Ust | uronyl-2-sulfotransferase |
| ENSMUSG00000000093 | 1.00 | 2.01 | 2.50E-03 | Tbx2 | T-box 2 |
