# Supplementary material for: Osteolytic cancer cells induce vascular/axon guidance processes in the bone/bone marrow stroma
Source: Oncotarget. 2018 Jun 22;9(48):28877–96. doi: 10.18632/oncotarget.25608 (PMC6034746; doi:10.18632/oncotarget.25608)
Supplement: Supplementary file 7 [file oncotarget-09-28877-s007.docx]

**Table S6.**

| **Gene name** | **TaqMan probe** |
| --- | --- |
| 18S | Hs99999901_s1 |
| Acta2 | Mm01546133_m1 |
| ACTA2 | Hs00426835_g1 |
| ACTB | Hs99999903_m1 |
| Actb | Mm02619580_g1 |
| Ang1 | Mm00456503_m1 |
| B2M | Hs99999907_m1 |
| B2m | Mm00437762_m1 |
| Cdc42 | Mm01194005_g1 |
| Cfl2 | Mm00483282_g1 |
| Chordin | Mm00438203_m1 |
| Csf1 | Mm00432686_m1 |
| Cspg4/Ng2 | Mm00507257_m1 |
| Dkk-1 | Mm00438422_m1 |
| Dkk-2 | Mm00445025_m1 |
| Dkk-3 | Mm00443800_m1 |
| Ecmn | Mm00497495_m1 |
| Efna1 | Mm01212795_m1 |
| Efna2 | Mm00433011_m1 |
| Efna4 | Mm00433013_m1 |
| Efna5 | Mm01237700_m1 |
| Efnb1 | Mm00438666_m1 |
| Efnb2 | Mm00438670_m1 |
| Eln | Mm00514670_m1 |
| Eng (Cd105) | Mm00468252_m1 |
| Epha4 | Mm00433056_m1 |
| Ephb1 | Mm00557955_m1 |
| Ephb2 | Mm01181021_m1 |
| Ephb3 | Mm00802553_m1 |
| Ephb4 | Mm01201157_m1 |
| Frzb | Mm00441378_m1 |
| Gap43 | Mm00500404_m1 |
| GAPDH | Hs99999905_m1 |
| Gnai1 | Mm01165301_m1 |
| HPRT1 | Hs_99999909_m1 |
| Hprt1 | Mm00446968_m1 |
| Itgav/Cd51 | Mm00434506_m1 |
| Itgb1 | Mm01253230_m1 |
| Lamb1 | Mm00801853_m1 |
| Ly6a | Mm04337234_mH |
| Met | Mm01156972_m1 |
| Nfat5 | Mm00467257_m1 |
| Nfatc4 | Mm00452375_m1 |
| Ngef | Mm00451232_m1 |
| Nog | Mm01297833_s1 |
| Nrp1 | Mm00435379_m1 |
| Ntn1 | Mm00500896_m1 |
| Ntn4 | Mm00480462_m1 |
| Pak3 | Mm01332263_m1 |
| Pdgfrb | Mm01298578_m1 |
| Pecam1 (Cd31) | Mm01246167_m1 |
| Plvap | Mm00453379_m1 |
| Plxna3 | Mm00501170_m1 |
| Plxnb1 | Mm00555359_m1 |
| Plxnb3 | Mm00502216_m1 |
| Ppp3cb | Mm00920265_m1 |
| Ppp3cb | Mm00920265_m1 |
| Ptk2 | Mm00433209_m1 |
| Ptprc/Cd45 | Mm01293577_m1 |
| Rac3 | Mm00652427_g1 |
| Rasa1 | Mm00520858_m1 |
| Rgs3 | Mm01267574_m1 |
| Rhod | Mm00455907_m1 |
| Rnd1 | Mm00553835_m1 |
| Robo1 | Mm00803879_m1 |
| Sema3a | Mm00436469_m1 |
| Sema3b | Mm00436477_m1 |
| Sema3c | Mm00443121_m1 |
| Sema3f | Mm00441325_m1 |
| Sema4c | Mm01229764_m1 |
| Sema4g | Mm00442518_m1 |
| Sema6c | Mm00441345_m1 |
| Sema7a | Mm00441361_m1 |
| Slit2 | Mm01216521_m1 |
| Slit3 | Ms_01326992_m1 |
| Sost | Mm00470479_m1 |
| Tie2 (Tek) | Mm00443243_m1 |
| Tm4sf19 | Mm01344503_m1 |
| Tnfsf11 (Rankl) | Mm00441906_m1 |
| Tubb3 | Mm00727586_s1 |
| Unc5c | Mm00494093_m1 |
| Uncb5 | Mm00504054_m1 |
| Vegfr1 (Flt1) | Mm00438980_m1 |
| Vegfr2 (Kdr) | Mm01222421_m1 |
| Vegfr3 (Flt4) | Mm01292604_m1 |
